# The global, regional, and national patterns of change in the burden of nonmalignant upper gastrointestinal diseases from 1990 to 2019 and the forecast for the next decade

**DOI:** 10.1097/JS9.0000000000001902

**Published:** 2024-07-03

**Authors:** Zihao Bai, Hao Wang, Chong Shen, Jia An, Zhaocong Yang, Xuming Mo

**Affiliations:** a Nanjing Children’s Hospital, Clinical Teaching Hospital of Medical School, Nanjing University, Nanjing, China; b Department of Cardiothoracic Surgery, Children’s Hospital of Nanjing Medical University, Nanjing, China

**Keywords:** disability-adjusted life years, gastritis and duodenitis, gastroesophageal reflux disease, health inequality, peptic ulcer disease

## Abstract

**Background:**

Nonmalignant upper gastrointestinal diseases, including peptic ulcer disease (PUD), gastritis and duodenitis (GD), and gastroesophageal reflux disease (GERD), significantly challenge global healthcare. These conditions not only impact patient health but also highlight socioeconomic development issues and healthcare system accessibility and efficiency. Utilizing the Global Burden of Disease database, this study aims to comprehensively assess the global burden of PUD, GD, and GERD, examining their association with the sociodemographic index (SDI).

**Methods:**

Employing data from the Global Burden of Disease 2019 database, this study analyzed the disability-adjusted life years for PUD, GD, and GERD. We integrated the SDI with the inequality slope index and concentration index for an international health inequality analysis, assessing disparities in the burden of these nonmalignant upper gastrointestinal diseases. Decomposition analysis was conducted to determine the effects of population growth, aging, and epidemiological change on disease burden. Frontier analysis was performed to identify potential improvement areas and disparities among countries by development status. Disease time trends were depicted using a Joinpoint regression model, and the Bayesian age-period-cohort model projected the disease burden up to 2030.

**Results:**

Between 1990 and 2019, the age-standardized disability-adjusted life years rates for nonmalignant upper gastrointestinal diseases declined. However, global geographic heterogeneity remained evident and closely linked to the SDI. Notably, low-SDI countries experienced higher disease burdens. Population growth and aging emerged as principal contributors to the increasing disease burden. Despite development levels, many countries have considerable potential for reducing the burden of these diseases. Furthermore, significant variations in the time trends of nonmalignant upper gastrointestinal diseases were observed among countries and regions with different SDI levels, a pattern expected to continue through 2030.

**Conclusion:**

Nonmalignant upper gastrointestinal diseases demonstrate notable heterogeneity across age, sex, and geography, with the disparities most marked in underdeveloped regions or countries. Consequently, it is imperative to focus research on policy development and to enact prevention and treatment strategies tailored to high-risk groups. This targeted approach is essential for effectively mitigating the disease burden.

## Introduction

Highlights

- The study uses the 2019 Global Burden of Disease (GBD) study to analyze the global burden of nonmalignant upper gastrointestinal diseases, linking it with the sociodemographic index (SDI).
- Findings show higher disease burdens in low-SDI countries, as analyzed through health inequality indices.
- Population growth and aging are identified as the main factors increasing the disease burden across nations.
- Future projections using Bayesian age-period-cohort (BAPC) model indicate persistent geographic and temporal disparities in disease burden related to SDI levels through 2030.
- The study highlights the need for targeted prevention and treatment strategies for high-risk groups to reduce the global disease burden.

Nonmalignant upper gastrointestinal diseases, including peptic ulcer disease (PUD), gastritis and duodenitis (GD), and gastroesophageal reflux disease (GERD), are hallmarked by chronic inflammation and tissue damage. These conditions pose substantial challenges to patient health and strain the global healthcare system^1,2^. Taking PUD as an example, its lifetime prevalence is about 5–10%, and the annual incidence rate is 0.1–0.3%^3^. These diseases are highly prevalent worldwide and can cause symptoms such as chronic pain and malnutrition, significantly affecting patients’ quality of life^4^. Additionally, the economic burden posed by nonmalignant upper gastrointestinal diseases cannot be overlooked. They not only escalate direct medical costs, including diagnosis, treatment, and hospitalization fees but also result in indirect economic losses, such as labor loss and reduced productivity^5^.

The high disease burden of these nonmalignant conditions often correlates with low socioeconomic development and limited healthcare access and efficiency^6,7^. Contrarily, recent research indicates that higher socioeconomic regions or countries might also experience a disproportionately high burden of these diseases^8,9^. This disparity suggests a complex interplay of etiological factors such as lifestyle changes, widespread *Helicobacter pylori* infection, and genetic susceptibility, which collectively contribute to the prevalence and progression of these conditions. Accordingly, tracking the trends of nonmalignant upper gastrointestinal diseases across various countries is vital for identifying risk factors, optimizing resource allocation, and mitigating the disease burden and its complications.

GBD 2019 studies are esteemed as an instrumental resource in epidemiological research. They facilitate classification and comparisons grounded on the SDI, offering a foundational framework for investigations in disease epidemiology, planning, implementation, and policy development. These studies play a crucial role in enhancing the judicious distribution of health resources^10^. Nonetheless, prior GBD 2019-based research on nonmalignant upper gastrointestinal diseases has predominantly concentrated on isolated diseases, thereby omitting a comprehensive examination of nonmalignant upper gastrointestinal diseases as a collective category.

This research examines the burden of three nonmalignant upper gastrointestinal diseases – PUD, GD, and GERD – and their prevalence across various age and sex demographics. Additionally, employing health equity analysis techniques endorsed by the WHO, this study undertakes a cross-national analysis of inequality to determine if disparities related to the SDI exist in the disease burden among countries. It evaluates the magnitude of these disparities and their evolution over time and forecasts their potential global implications in the future.

## Methods

### Data source

Data for this study were sourced from the GBD 2019 database, accessible through the GBD results tool on the Institute for Health Metrics and Evaluation (IHME) website (http://ghdx.healthdata.org/)^11^. The GBD 2019 estimation process amalgamates various data sources, including censuses, household surveys, civil registration and vital statistics systems, disease registries, health service utilization records, air pollution monitoring data, satellite imagery, disease notifications, and more. This comprehensive data collection stems from systematic literature reviews, government, and international organization website searches, report analyses, and evaluations of primary data sources like population and health surveys. Additionally, the dataset includes contributions from GBD collaborators^12^.

In this research, disability-adjusted life years (DALYs) estimates, along with their 95% uncertainty intervals (UI) for three nonmalignant upper gastrointestinal diseases – PUD, GD, and GERD – were extracted from the GBD 2019 database. DALYs are calculated by adding years of life lost due to disease and years lived with disability, thereby comprehensively measuring both the fatal and nonfatal burdens of disease^11^. Years of life lost are determined by multiplying the number of deaths caused by each disease by the standard expected remaining years of life, while years lived with disability quantify the years lived with disability caused by the disease. DALYs offer a comprehensive reflection of the overall impact of diseases on population health by combining both fatal and nonfatal health losses, thus providing a unified measure of health burden.

Furthermore, the study employed the SDI, a composite measure that evaluates the sociodemographic status of a country or region based on its average income, educational attainment, and fertility rates^13^. Each country or region has a corresponding SDI value, ranging from 0 to 1, with higher values indicating higher levels of sociodemographic development. By stratifying the SDI values of 204 countries or regions, different levels of development can be distinguished. In this study, the SDI is used to reflect the differences in the burden of nonmalignant upper gastrointestinal diseases across various socioeconomic backgrounds, providing a basis for cross-country health inequality analysis.

### Cross-country inequality analysis

The inequality slope index and the concentration index are standardized indicators for measuring absolute and relative gradient inequalities, respectively. They quantify inequality in the burden distribution of nonmalignant upper gastrointestinal diseases among countries^14^. The inequality slope index is obtained through regression analysis, relating a country’s DALYs rates to its SDI relative position, defined by the population’s midpoint in a cumulative distribution ranked by SDI. Heteroscedasticity is examined using a weighted regression model. The concentration index is calculated by numerically integrating the area under the Lorenz curve, aligning the cumulative proportion of DALYs with the population’s cumulative distribution sorted by SDI^15^.

### Decomposition analysis

To elucidate the principal factors influencing the changes in the burden of nonmalignant upper gastrointestinal diseases between 1990 and 2019, a decomposition analysis was performed. This analysis sought to quantify the separate impacts of population growth, aging, and epidemiological change. The methodology entailed assessing the contribution of each factor in isolation, with the other two factors remaining fixed^16^.

### Statistics

DALYs rates are presented as estimates per 100 000 population, accompanied by their 95% UI. The analyses and visualizations in this study were conducted using the World Health Organization Health Equity Assessment Toolkit along with R software (version 4.3.2).

### Reporting standards

This study adhered to the Strengthening the Reporting of Cohort Studies in Surgery (STROCSS, Supplemental Digital Content 1, http://links.lww.com/JS9/D13) guidelines for observational studies in its protocol development, execution, and reporting^17^.

## Result

### Peptic ulcer disease

**Figure 1:**
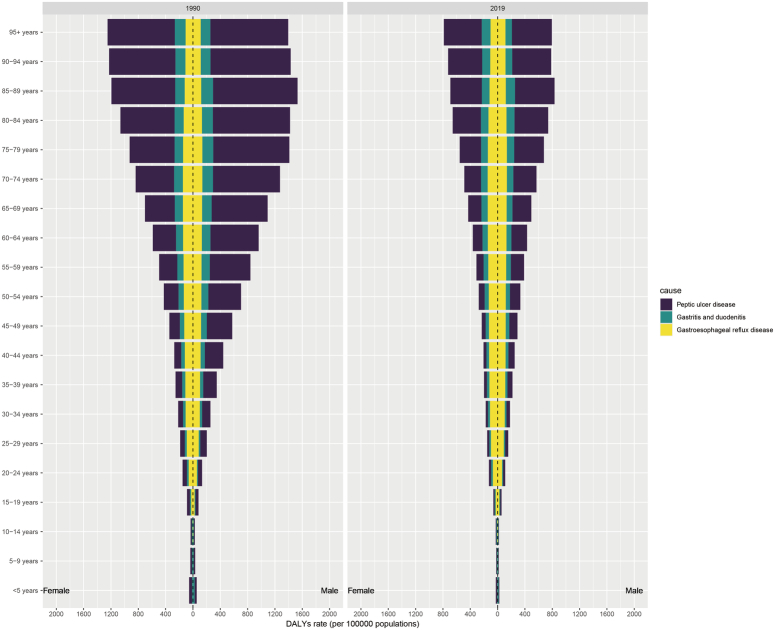
The distribution of global DALYs rates for PUD, GD, and GERD by age and sex in 1990 and 2019. The left panel displays the distribution for the year 1990, whereas the right panel delineates the distribution for 2019. The color-coded bars represent DALYs rates for PUD (purple), GD (green), and GERD (yellow) across various age groups. The bars are bifurcated to reflect males (on the right side of the dashed line) and females (on the left side of the dashed line). DALYs, disability-adjusted life years; GD, gastritis and duodenitis; GERD, gastroesophageal reflux disease; PUD, peptic ulcer disease.

**Figure 2:**
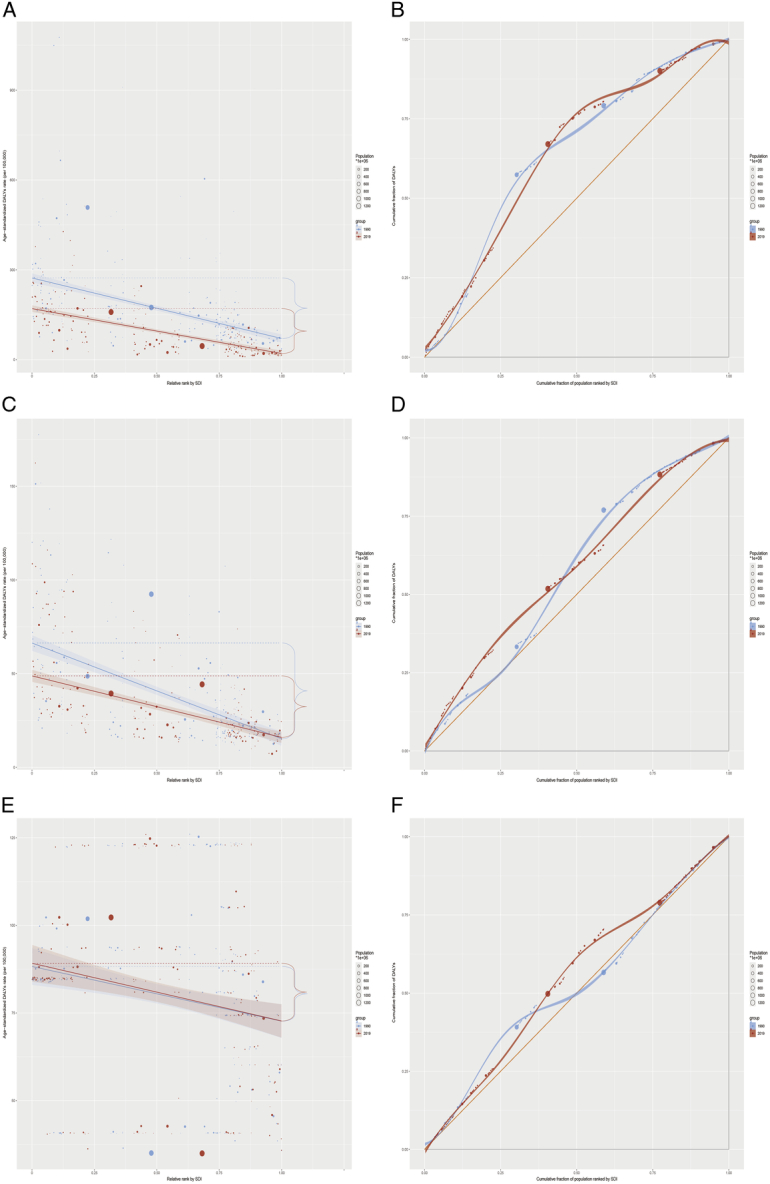
The inequality slope index and concentration index for DALYs of PUD (A–B), GD (C–D), and GERD (E–F) worldwide in 1990 and 2019. Panels A, C, and E illustrate the inequality slope index, depicting the relationship between SDI and age-standardized DALYs rates for each condition, with points representing individual countries sized by population. Panels B, D, and F present the concentration index, which quantifies relative inequalities by integrating the area under the Lorenz curve, aligning DALYs distribution with population distribution by SDI. Blue represents data from 1990, and red represents data from 2019. DALYs, disability-adjusted life years; GD, gastritis and duodenitis; GERD, gastroesophageal reflux disease; PUD, peptic ulcer disease; SDI, sociodemographic index.

In 2019, significant differences in the age-standardized disability-adjusted life years rates (ASDR) of PUD across 204 countries were noted (Supplementary Table S1, Supplemental Digital Content 2, http://links.lww.com/JS9/D14). Kiribati exhibited the highest ASDR at 512.71 (95% UI: 362.22–701.02), while Israel reported the lowest at 9.88 (95% UI: 8.43–11.84). In 1990, the highest DALYs rates were observed in the age group of 95 years and above, with males showing higher rates across all age groups compared to females. By 2019, despite a decrease in DALYs rates, the highest rates persisted in the age group of 95 years and above, and males continued to exhibit higher rates than females across all age groups (Fig. 1 and Supplementary Table S2, Supplemental Digital Content 3, http://links.lww.com/JS9/D15). The study also revealed significant absolute and relative inequalities in PUD burden associated with SDI, where low-SDI regions faced a disproportionately high burden. The inequality slope index indicated a reduction in the DALYs rate gap between the highest and lowest SDI countries, from −202.2 (95% CI: −232.9 to −171.6) in 1990 to −150.0 (95% CI: −170.2 to −129.8) in 2019. The concentration index changed slightly from −28.7 (95% CI: −33.4 to −22.8) in 1990 to −28.8 (95% CI: −33.1 to −24.4) by 2019, suggesting persistent inequalities (Fig. 2A–B and Supplementary Table S3–S4, Supplemental Digital Content 4, http://links.lww.com/JS9/D16, Supplemental Digital Content 5, http://links.lww.com/JS9/D17).

**Figure 3:**
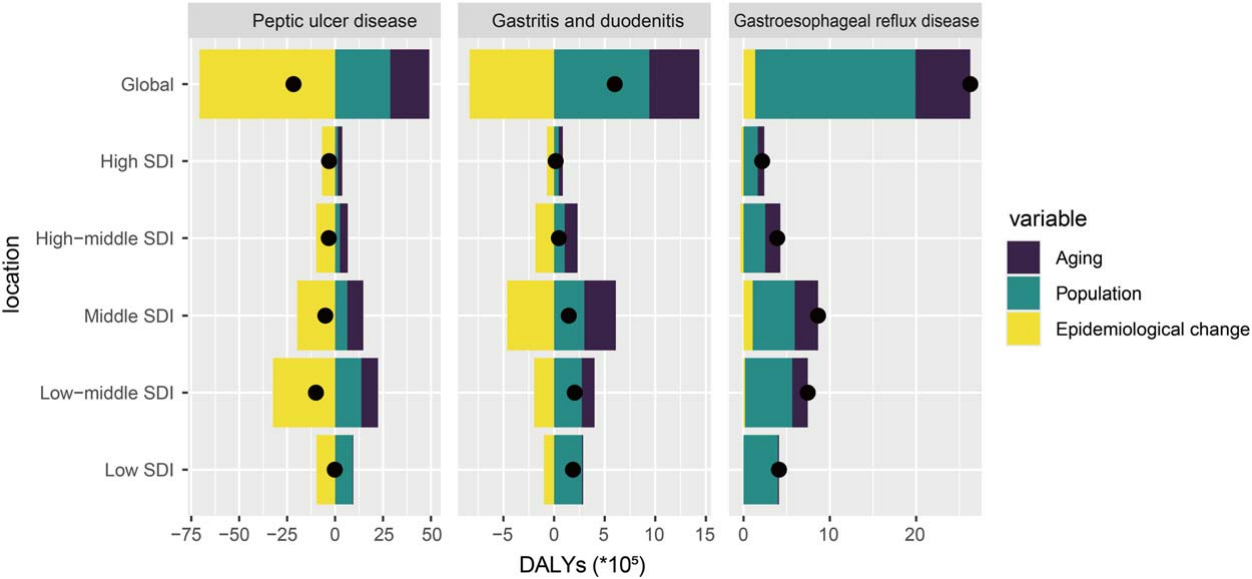
Changes in PUD, GD, and GERD DALYs according to population-level determinants of aging, population growth, and epidemiological change from 1990 to 2019 at the global level and by SDI quintile. The black dot represents the overall value of change contributed by all three components. For each component, the magnitude of a positive value indicates a corresponding increase in DALYs attributed to that component, while the magnitude of a negative value indicates a corresponding decrease in DALYs attributed to the related component. DALYs, disability-adjusted life years; GD, gastritis and duodenitis; GERD, gastroesophageal reflux disease; PUD, peptic ulcer disease; SDI, sociodemographic index.

Through decomposition analysis of the original DALYs for PUD, this study evaluated the effects of aging, population growth, and epidemiological change on PUD from 1990 to 2019 (Supplementary Table S5, Supplemental Digital Content 6, http://links.lww.com/JS9/D18). Globally, and across all SDI regions, DALYs for PUD demonstrated a declining trend, most notably within the low-middle-SDI regions where the decrease was pronounced (Fig. 3). On a global scale, the contributions of population growth and aging to the disease burden increase were −94.08 and −132.57%, respectively (Supplementary Table S5, Supplemental Digital Content 6, http://links.lww.com/JS9/D18). Aging’s impact varied across different SDI regions, with a significant −5278.29% in the low-SDI regions, followed by −136.49, −123.78, −74.51, and −43.54% in low-middle, middle, high-middle, and high-SDI regions, respectively. The adverse effect of aging on disease burden diminishes with higher SDI levels. Epidemiological changes led to a global increase in disease burden, notably in low-SDI regions, where the rise was 5601.26%.

**Figure 4:**
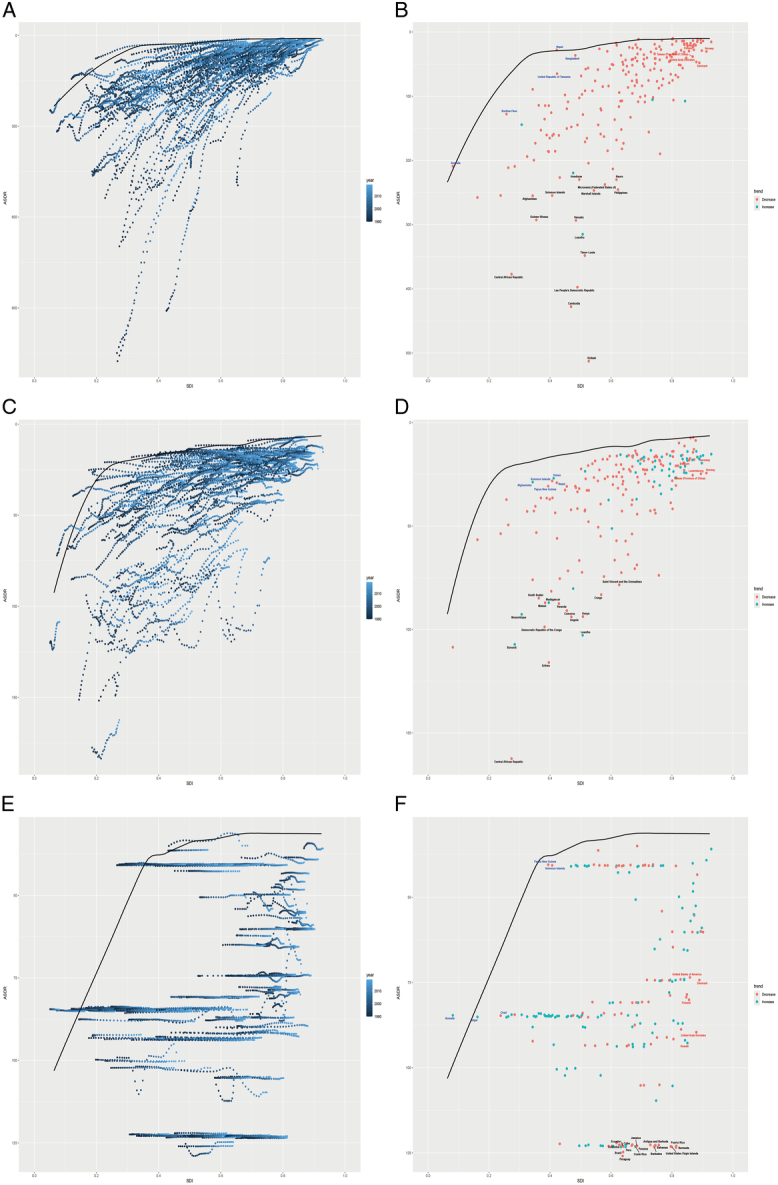
Frontier analysis based on the age-standardized rates of DALYs for PUD (A–B), GD (C–D), and GERD (E–F), and SDI over the decades (1990–2019) and specifically in 2019. Panels A, C, and E illustrate the frontier analysis based on ASDR and SDI from 1990 to 2019. The color scale ranges from dark blue (1990) to light blue (2019). The solid black line delineates the frontier. Panels B, D, and F illustrate the frontier analysis based on ASDR and SDI in 2019. The solid black line delineates the frontier, and the dots represent countries and territories. The top 15 countries and territories with the largest effective difference are labeled in black. Examples of frontier countries with low SDI (<0.5) and low effective differences are labeled in blue, and examples of countries and territories with high SDI (>0.85) and relatively high effective difference are labeled in red. Red dots indicate a decrease in ASDR, while blue dots indicate an increase in ASDR from 1990 to 2019. ASDR, age-standardized disability-adjusted life years rates; DALYs, disability-adjusted life years; GD, gastritis and duodenitis; GERD, gastroesophageal reflux disease; PUD, peptic ulcer disease; SDI, sociodemographic index.

Utilizing data spanning from 1990 to 2019 and employing ASDR alongside the SDI, this study performed frontier analysis to assess the potential improvement space for the burden of PUD relative to national development levels (Supplementary Table S6, Supplemental Digital Content 7, http://links.lww.com/JS9/D19). Taking national development into account, the top 15 countries with the most significant actual potential for improvement (effective difference range: 489.23–201.73) were identified as Kiribati, Cambodia, Lao People’s Democratic Republic, Timor-Leste, Central African Republic, Lesotho, Vanuatu, Guinea-Bissau, Philippines, Marshall Islands, Solomon Islands, Afghanistan, Micronesia (Federated States of), Nauru, and Honduras. Frontier nations with low SDI (<0.5) include Somalia, Burkina Faso, the United Republic of Tanzania, Bangladesh, and Nepal. Notably, countries and regions with a high SDI (>0.85) yet demonstrating substantial room for improvement given their development stage are Finland, Norway, Taiwan (Province of China), United Arab Emirates, and Denmark (Fig. 4A–B).

**Figure 5:**
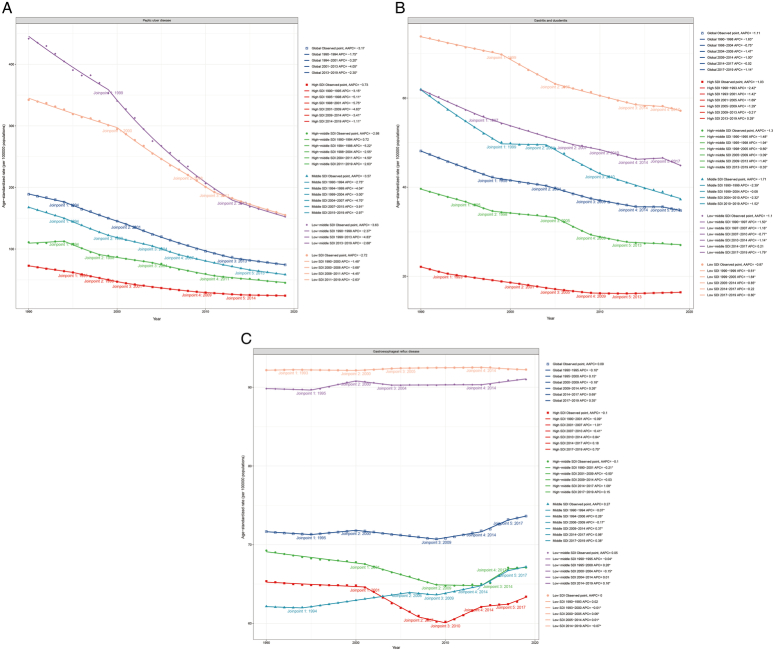
Joinpoint regression analysis of the age-standardized DALYs rates for PUD (A), GD (B), and GERD (C) at the global level and by SDI quintile from 1990 to 2019. Each panel demonstrates the temporal trends of DALYs rates with specific joinpoints indicating significant changes in trends. The analysis includes global data and different SDI quintiles, represented by various colored lines, showing the APC and AAPC across different locations. AAPC, average annual percentage change; APC, annual percentage change; DALYs, disability-adjusted life years; GD, gastritis and duodenitis; GERD, gastroesophageal reflux disease; PUD, peptic ulcer disease; SDI, sociodemographic index.

The Joinpoint regression model was employed to examine the temporal trends of PUD ASDR both globally and across different SDI regions, revealing notable differences in ASDR trends among the SDI regions (Fig. 5A). Globally, the PUD burden exhibited a significant declining trend, characterized by a gradual decrease from 1990 to 1994 [annual percentage change (APC)=−1.75], followed by an accelerated decline from 1994 to 2001 (APC=−3.20) and 2001 to 2013 (APC=−4.05), and subsequently, a slowdown in the rate of decline from 2013 to 2019 (APC=−2.30). Except for the high-middle-SDI region, all regions displayed a pattern of initial acceleration and subsequent deceleration in the decline rate. Specifically, the high-middle-SDI regions experienced an initial increase from 1990 to 1994 (APC=0.72), then observed declines at various stages: 1994–1998 (APC=−5.22), 1998–2004 (APC=−2.55), 2004–2011 (APC=−4.50), and 2011–2019 (APC=−2.63).

**Figure 6:**
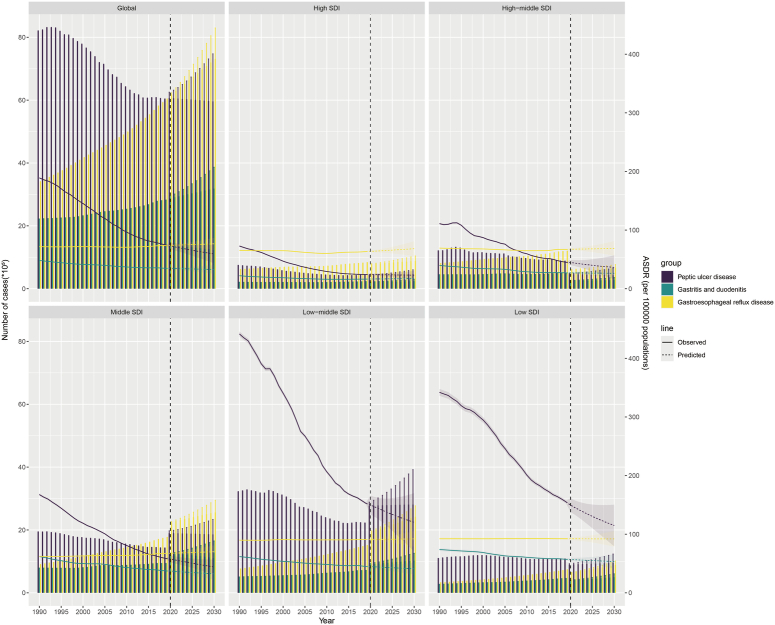
Temporal trends of the number of DALYs and age-standardized DALYs rates for PUD, GD, and GERD at the global level and by SDI quintile from 1990 to 2030. The solid line represents the observed age-standardized DALYs rate, and the dashed line represents the age-standardized DALYs rate predicted by the BAPC model. BAPC, Bayesian age-period-cohort; DALYs, disability-adjusted life years; GD, gastritis and duodenitis; GERD, gastroesophageal reflux disease; PUD, peptic ulcer disease; SDI, sociodemographic index.

Predictions based on the SDI indicate significant heterogeneity in the global PUD ASDR (Fig. 6 and Supplementary Table S9–10, Supplemental Digital Content 10, http://links.lww.com/JS9/D22 http://links.lww.com/JS9/D23, Supplemental Digital Content 11, http://links.lww.com/JS9/D23). The BAPC model forecasts an 11.7% decrease in total global DALYs for PUD, approximately 5 959 128 years, from 2019 to 2030. Within this timeframe, the largest decline in DALYs is anticipated in the high-middle-SDI regions, with a 51.5% reduction. Conversely, an increase in total DALYs is expected in the high-SDI, middle-SDI, and low-middle-SDI regions over the next decade, with the middle-SDI regions seeing the most substantial growth at 30.2%. Moreover, the model predicts a 20.3% decrease in PUD ASDR per 100 000 individuals, from 74.40 in 2019 to 59.31 by 2030. Between 2019 and 2030, the steepest drop in ASDR is forecasted for low-SDI regions (−25.9%), while high-SDI regions are expected to experience the smallest reduction in PUD ASDR during this period (−4.2%).

### Gastritis and duodenitis

In 2019, notable variations in the ASDR for GD were recorded across 204 countries (Supplementary Table S1, Supplemental Digital Content 2, http://links.lww.com/JS9/D14), with the Central African Republic registering the highest ASDR at 162.44 (95% UI: 100.26–272.14) and Japan the lowest at 7.2 (95% UI: 4.68–10.53). In 1990, the highest ASDR was seen in individuals aged 95 and above, with females aged 5–9 to 30–34 years, as well as those aged 90–94 and 95 and above, experiencing higher DALYs rates than males. By 2019, the age group of 85–89 years showed the most significant DALYs rates, and beyond the age groups identified in 1990, females aged 35–39 to 70–74 years also displayed higher DALYs rates than males (Fig. 1 and Supplementary Table S2, Supplemental Digital Content 3, http://links.lww.com/JS9/D15). Regarding GD’s burden, pronounced absolute and relative inequalities were linked to the SDI, particularly in low-middle-SDI regions, which faced an excessively high burden. The inequality slope index revealed a narrowing in the DALYs rate disparity between the highest and lowest SDI countries, from −51.1 (95% CI: −59.3 to −42.9) in 1990 to −32.9 (95% CI: −39.0 to −26.7) in 2019. Meanwhile, the concentration index shifted from −14.8 (95% CI: −10.6 to −19.0) in 1990 to −16.3 (95% CI: −12.9 to −19.7) by 2019 (Fig. 2C–D and Supplementary Table S3–S4, Supplemental Digital Content 4, http://links.lww.com/JS9/D16, Supplemental Digital Content 5).

A decomposition analysis of the original DALYs was conducted to evaluate the distinct effects of aging, population growth, and epidemiological change on GD from 1990 to 2019 (Supplementary Table S5, Supplemental Digital Content 6, http://links.lww.com/JS9/D18). Overall, DALYs for GD exhibited a rising trend globally and in all SDI regions, with the most notable increase observed in low-middle-SDI regions (Fig. 3). On a global scale, population growth and aging contributed to 156.94 and 82.50% of the increase in disease burden, respectively (Supplementary Table S5, Supplemental Digital Content 6, http://links.lww.com/JS9/D18). The influence of population growth was most pronounced in high-SDI regions at 289.31%, followed by high-middle-SDI, middle-SDI, low-SDI, and low-middle-SDI regions at 219.40, 204.83, 148.13, and 133.73%, respectively. Aging had a more substantial impact in higher SDI regions, particularly in high-middle-SDI (269.79%), high-SDI (252.48%), middle-SDI (214.96%), low-middle-SDI (62.39%), and low-SDI (5.52%) regions. Conversely, epidemiological changes resulted in a decrease in the global disease burden for most regions, with the most significant reduction seen in the high-middle-SDI regions (−389.19%).

Using data from 1990 to 2019 and conducting a frontier analysis based on ASDR and SDI, this study explores the improvement potential in the disease burden of GD, considering the level of national development (Supplementary Table S7, Supplemental Digital Content 8, http://links.lww.com/JS9/D20). The analysis identifies 15 countries with the most significant potential for actual improvement in GD burden, with an effective difference range of 141.62–66.23. These countries, including the Central African Republic, Eritrea, Lesotho, Burundi, Democratic Republic of the Congo, Kenya, Angola, Comoros, Rwanda, Mozambique, Congo, Madagascar, Malawi, Saint Vincent and the Grenadines, and South Sudan, exhibit higher GD ASDR compared to others under similar sociodemographic conditions. Frontier countries with low SDI (<0.5) are Afghanistan, Yemen, Solomon Islands, Papua New Guinea, and Nepal, indicating a significant need for targeted health interventions. Conversely, countries and territories like Norway, Taiwan (Province of China), Luxembourg, Belgium, and Germany, despite having high SDI (>0.85), show relatively high effective differences at their development level, suggesting room for improvement in managing GD burden (Fig. 4C–D).

The Joinpoint regression model was utilized to examine the temporal patterns of GD disease burden globally and across SDI regions, uncovering notable differences among regions (Fig. 5B). Globally, GD’s ASDR exhibited a consistent downward trend (APC from 1990 to 1998=−1.63; 1998 to 2004=−0.75; 2004 to 2009=−1.47; 2009 to 2014=−1.00; 2014 to 2017=−0.02; 2017 to 2019=−1.14). Excluding the high-SDI and low-middle-SDI regions, all regions demonstrated a continual decline in GD’s disease burden. In high-SDI regions, an initial decreasing trend in GD burden was noted (APC from 1990 to 1993=−2.42; 1993 to 2001=−1.42; 2001 to 2005=−1.69; 2005 to 2009=−1.29; 2009 to 2013=−0.21), followed by an increase from 2013 to 2019 (APC=0.29). Conversely, the disease burden in low-middle-SDI regions displayed a more complex pattern, initially decreasing (APC from 1990 to 1997=−1.50; 1997 to 2007=−1.16; 2007 to 2010=−0.77; 2010 to 2014=−1.14), then increasing from 2014 to 2017 (APC=0.21), and again decreasing from 2017 to 2019 (APC=−1.79).

Predictions based on the SDI reveal significant heterogeneity in the global GD ASDR (Fig. 6 and Supplementary Table S9–10, Supplemental Digital Content 10, http://links.lww.com/JS9/D22, Supplemental Digital Content 11, http://links.lww.com/JS9/D23). The BAPC model forecasts a 13.2% increase in total global DALYs for GD, approximately 3 186 281 years, from 2019 to 2030, with the most substantial growth projected in low-middle-SDI regions (44.9%). Conversely, total DALYs for high-middle-SDI regions are anticipated to decline in the next decade (−44.2%). The model also estimates a 5.5% reduction in GD’s ASDR per 100 000, from 34.78 in 2019 to 32.86 by 2030. The most notable decrease in ASDR from 2019 to 2030 is expected in middle-SDI regions (−12.9%), while an increase is predicted for high-SDI regions over the same period (5.3%).

### Gastroesophageal reflux disease

In 2019, notable disparities in GERD’s ASDR were recorded across 204 countries, with Paraguay registering the highest ASDR at 125.91 (95% UI: 65.11–223.26) and China the lowest at 34.94 (95% UI: 17.73–63.02) (Supplementary Table S1, Supplemental Digital Content 2, http://links.lww.com/JS9/D14). In 1990, the peak DALYs rates were seen in individuals aged 70–74 years, and except for those aged 85–89, 90–94, and 95 years and older, females experienced higher DALYs rates than males. By 2019, despite reductions in DALYs rates, the highest DALYs rates persisted in the 70–74 age group, and females, excluding the aforementioned age groups, continued to exhibit higher DALYs rates than males (Fig. 1 and Supplementary Table S2, Supplemental Digital Content 3, http://links.lww.com/JS9/D15). GERD showed both absolute and relative inequalities in relation to the SDI, with low-SDI countries shouldering a comparatively larger burden. The slope index revealed that the disparity in DALYs rates between the highest and lowest SDI countries widened slightly from −15.6 (95% CI: −27.9 to −3.3) in 1990 to −16.5 (95% CI: −28.8 to −4.2) in 2019. The concentration index increased from −5.0 (95% CI: −1.9 to −8.1) in 1990 to −10.1 (95% CI: −7.4 to −12.8) by 2019 (Fig. 2E–F and Supplementary Table S3-4, Supplemental Digital Content 4, http://links.lww.com/JS9/D16, Supplemental Digital Content 5).

Utilizing data from 1990 to 2019, this study conducted a decomposition analysis of the original DALYs to assess the influence of aging, population growth, and epidemiological change on the disease burden of GERD (Supplementary Table S5, Supplemental Digital Content 6, http://links.lww.com/JS9/D18). Globally, and across all SDI regions, GERD DALYs exhibited an upward trend, most notably in the middle-SDI regions (Fig. 3). At the global scale, population growth and aging were responsible for 70.65 and 24.22% of the GERD burden increase, respectively (Supplementary Table S5, Supplemental Digital Content 6, http://links.lww.com/JS9/D18). Population growth’s impact was especially significant in low-SDI regions, reaching 96.89%; in high-SDI, low-middle-SDI, high-middle-SDI, and middle-SDI regions, the contributions were 75.98, 73.55, 64.09, and 56.44%, respectively. Aging’s contribution was predominantly observed in higher SDI regions [high-middle-SDI (45.01%), high-SDI (35.34%), middle-SDI (31.35%), low-middle-SDI (24.27%), low-SDI (3.16%)]. Epidemiological changes contributed to an increase of 12.21% in middle-SDI regions and 2.18% in low-middle-SDI regions, whereas they resulted in a decrease of 11.32% in high-SDI, 9.10% in high-middle-SDI, and 0.06% in low-SDI regions.

Utilizing data from 1990 to 2019 and based on ASDR and SDI, this study aims to delineate the potential for improvement in GERD’s disease burden, factoring in national development levels (Supplementary Table S8, Supplemental Digital Content 9, http://links.lww.com/JS9/D21). According to national development, the top 15 countries with the most significant potential for improvement, with an effective difference range of 94.06–91.44, comprise Paraguay, Brazil, Bermuda, Peru, Barbados, United States Virgin Islands, Ecuador, Bahamas, Colombia, Costa Rica, Antigua and Barbuda, Puerto Rico, Jamaica, Panama, and Cuba. These nations exhibit notably higher GERD ASDR than others under comparable sociodemographic conditions. Frontier countries with a low SDI (<0.5) are Somalia, Niger, Chad, Papua New Guinea, and Solomon Islands. Conversely, countries like Kuwait, the United Arab Emirates, Finland, Denmark, and the United States of America, despite possessing a high SDI (>0.85), demonstrate considerable potential for improvement given their developmental stage (Fig. 4E–F).

The Joinpoint regression model was utilized to assess the temporal trends of GERD ASDR both globally and across different SDI regions, uncovering significant differences among these regions over time (Fig. 5C). Globally, the GERD disease burden exhibited an initial decrease from 1990 to 1995 (APC=−0.10), a slight increase from 1995 to 2000 (APC=0.15), a subsequent decrease from 2000 to 2009 (APC=−0.18), and has been on a consistent rise since 2009, particularly from 2009 to 2014 (APC=0.26), 2014 to 2017 (APC=0.69), and 2017 to 2019 (APC=0.35). The trends in middle-SDI, low-middle-SDI, and low-SDI regions mirrored the global pattern, showing an initial decrease, a rise, another decrease, and a sustained increase after that. Conversely, high-SDI and high-middle-SDI regions displayed a continuous decline followed by a persistent increase [(high-SDI: 1990−2001 APC=−0.09; 2001−2007 APC=−1.01; 2007−2010 APC=−0.41; 2010−2014 APC=0.84; 2014−2017 APC=0.18; 2017−2019 APC=0.70), (high−middle-SDI: 1990−2001 APC=−0.21; 2001−2009 APC=−0.50; 2009−2014 APC=−0.03; 2014−2017 APC=1.08; 2017−2019 APC=0.15)].

Global projections for GERD ASDR, segmented by the SDI, indicate substantial variation across different SDI regions (Fig. 6 and Supplementary Table S9–S10, Supplemental Digital Content 10, http://links.lww.com/JS9/D22, Supplemental Digital Content 11, http://links.lww.com/JS9/D23). According to the BAPC model, from 2019 to 2030, the total global DALYs for GERD are expected to rise by 21.3% to 7 311 204 years, with the steepest increase projected for low-middle-SDI regions at 61.0%. In contrast, high-middle-SDI regions are anticipated to see a significant reduction in total DALYs by 45.5% within the next decade. Moreover, the model forecasts a 3.9% increase in the GERD ASDR per 100 000 people, from 73.63 in 2019 to 76.53 by 2030. The most substantial increase in ASDR from 2019 to 2030, amounting to 7.6%, is predicted for high-SDI regions. Concurrently, a slight decline of 0.2% in GERD ASDR is expected in the low-SDI regions.

## Discussion

This research utilized data from the GBD study 2019 for an extensive and current evaluation. It detailed the disease burden of PUD, GD, and GERD, segmented by age, sex, SDI, and geographical areas. Additionally, it examined cross-national disparities and forecasted future disease burdens. Diverging from earlier studies that mainly concentrated on incidence or mortality, this analysis employs DALYs to measure disease burden, thus providing a novel viewpoint for a thorough comprehension of these impacts of nonmalignant upper gastrointestinal diseases.

Since 1990, there has been a downward trend in the ASDRs for global nonmalignant upper gastrointestinal diseases, yet the patterns of these diseases have evolved significantly in relation to age and sex. Specifically focusing on GD, the primary burden shifted from the over-95 age group in 1990 to the 85–89 age group by 2019. This change in age distribution highlights the potential impact of health interventions targeted at older populations, suggesting that conditions previously prevalent in higher age groups are now appearing earlier, indicative of the effectiveness of preventative and early intervention strategies. Additionally, the study observed a trend where, despite a general predominance of these diseases in men, the burden in certain female age groups has increasingly exceeded that of their male counterparts from 1990 to 2019. Factors such as sex differences in accessing and utilizing medical services, amplified by cultural and social barriers faced by women, might have contributed to this trend. This disparity is particularly noticeable among older women and may influence disease prevention, early detection, and treatment efforts. This observation is supported by research, including a study from Tanzania^18^. Enhanced societal recognition of women’s health needs has led to stronger research and public health initiatives for diseases affecting women, suggesting that the impact of these conditions on women might have been previously underappreciated. Moreover, the past decades have seen a rise in awareness and intervention against unhealthy lifestyle choices among men, like poor diet, stress, inactivity, smoking, and excessive alcohol use. Concurrently, risk behaviors among women have also seen an uptick^19–22^.

The impact of age distribution and population aging on the burden of nonmalignant upper gastrointestinal diseases must be considered. As the global population ages, significant regional differences in the aging process profoundly impact disease incidence and burden. The incidence of these diseases is closely related to age, and aging not only increases the disease burden among the elderly but also affects the overall burden by altering the age distribution of disease onset. Notably, the rate of aging varies globally, potentially leading to an imbalance in disease burden across regions. High-SDI regions typically have a longer life expectancy and a faster aging process, resulting in a more significant burden of non-malignant upper gastrointestinal diseases among the elderly. Conversely, although low-SDI regions experience a relatively slower rate of aging, insufficient health resources, and limited health services result in poor disease management and treatment outcomes among the elderly, maintaining a high disease burden.

Globally, differences in diet and lifestyle across regions significantly impact the burden of nonmalignant upper gastrointestinal diseases. For example, the rising obesity rate is a crucial risk factor for GERD. According to the global obesity atlas, obesity rates are significantly correlated with national income levels, and regions with high obesity rates overlap with those with high GERD prevalence^23^. Obesity can lead to increased intra-abdominal pressure, resulting in an elevated gastroesophageal pressure gradient and decreased lower esophageal sphincter pressure, thereby triggering GERD. Furthermore, the availability and adherence to proton pump inhibitors (PPIs) also significantly impact the burden of PUD. The widespread use of PPIs has significantly reduced the incidence and recurrence rates of PUD. However, in low-SDI countries, limited medical resources and poor drug adherence hinder regular PPI use. Consequently, health systems often struggle to afford the consistent use of PPIs, and patients find it challenging to adhere to long-term treatment, resulting in a persistently high burden of PUD^24^.

The SDI disparities significantly influence the geographical distribution of the burden of nonmalignant upper gastrointestinal diseases, with countries classified as low-middle-SDI and middle-SDI experiencing the greatest impact. In countries with low SDI, the relatively lower disease burden may be attributed to factors such as inadequate epidemiological data coverage, limited diagnostic facilities, incomplete disease screening mechanisms, and constrained healthcare resources^25^. PUD exhibits the most substantial health inequality among these conditions, though its disparity has notably decreased from 1990 to 2019. Conversely, GERD presents the least pronounced health inequality, which has remained relatively unchanged over recent decades. GERD’s unique risk factors, including modern lifestyle elements like increasing obesity rates, may contribute to persistently stable health inequality^26,27^. Additionally, the long-term management of GERD, involving sustained lifestyle modifications and ongoing medication, poses particular challenges for individuals with lower socioeconomic status, potentially worsening health disparities.

PUD registers the highest ASDR among nonmalignant upper gastrointestinal diseases, a phenomenon significantly linked to the worldwide rise in *H. pylori* infections, notably in low-income and middle-income countries^28,29^. Studies highlight that *H. pylori*’s annual recurrence rate in developing countries reaches 13%, a figure considerably higher than the 2.7% observed in developed nations^30^. Contributing factors include poor dietary hygiene among low-income populations, inadequate health regulation, and insufficient increases in prevention and control budgets^31^. Additionally, the recurring nature and prolonged treatment demands of peptic ulcers diminish patients’ life quality, further intensifying the disease burden^32^. Despite a general decline in the burden of nonmalignant upper gastrointestinal diseases globally and across all SDI regions, dissecting DALYs data uncovers distinct insights. The burden increase in GD and GERD is chiefly propelled by aging and population growth, with epidemiological change exerting a negative influence. In contrast, PUD’s trend diverges as epidemiological changes significantly contribute to its burden escalation. The impact of diet and lifestyle factors is evident not only globally but also within each SDI region. Therefore, public health interventions targeting these factors, such as controlling obesity, improving the accessibility and adherence to PPIs, and strengthening the prevention and control of *H. pylori* infection, are crucial for reducing the burden of nonmalignant upper gastrointestinal diseases.

This study has significant implications for policy-making and prevention and treatment strategies. Firstly, policymakers should develop differentiated health policies based on the specific needs of countries with different SDI levels. In low-SDI countries, priority should be given to enhancing disease screening and diagnostic capabilities, especially in resource-poor areas, to ensure early detection and intervention, thereby reducing disease progression and complications. Additionally, strengthening public health education and raising awareness of nonmalignant upper gastrointestinal diseases and their risk factors is crucial for prevention and early treatment. In high-SDI countries, policies should focus on managing lifestyle-related risk factors. Specific measures include promoting healthy eating, reducing smoking, and limiting excessive alcohol consumption to control the prevalence of obesity and *H. pylori* infection. Furthermore, targeted intervention programs should be strengthened, especially by providing accessible and affordable healthcare services for high-risk populations to improve treatment adherence and outcomes. Global health policies must also fully consider sex differences to ensure that women have fair access to healthcare services. Particularly in certain cultural and social contexts, women may face more barriers to accessing health services, making it essential to develop and implement targeted policies to eliminate these barriers.

Utilizing ASDR and SDI data, we carried out a frontier analysis at the national level. This analysis highlights significant room for enhancement in managing the burden of nonmalignant upper gastrointestinal diseases. Remarkably, low-SDI countries, including Somalia, Nepal, Solomon Islands, and Papua New Guinea, have demonstrated outstanding management of these diseases’ burden. Despite their resource constraints, these nations stand out as exemplary cases, showcasing effective strategies for improving health outcomes in contexts with limited resources. On the flip side, high-SDI countries such as Finland, Norway, and Denmark have underperformed relative to their developmental status in addressing the burden of nonmalignant upper gastrointestinal diseases. Factors like geographical location and dietary habits may play a role in this discrepancy. However, this indicates a pressing need for enhanced optimization and reforms in health policy formulation and execution in these countries.

Employing the BAPC model, this research forecasts the global trajectory of nonmalignant upper gastrointestinal diseases from 2019 to 2030. The findings suggest a general decline in the global ASDR for these diseases, yet the DALYs exhibit varying trends. Notably, the burden of PUD is projected to decrease by 11.7%, while GERD’s burden is expected to increase by 21.3% and the burden of GD by 13.2%. Additionally, the impact of these diseases displays significant regional disparities across different SDI categories. While the overall burden across all SDI regions is anticipated to decline, high-SDI regions will see a minimal decrease in PUD ASDR, with expected increases in ASDR for GD and GERD. The faster aging process in the high-SDI regions may significantly contribute to this phenomenon. Despite relatively effective global health policies for PUD and GD, the persistently high burden of GERD underscores the inadequacy of current strategies targeting GERD, highlighting the urgent need for enhanced approaches^33–36^.

Despite the significant burden posed by non-malignant upper gastrointestinal diseases, there is optimism regarding the potential impact of various initiatives on reducing this burden in the future. Such initiatives include the WHO’s ‘Global Action Plan for the Prevention and Control of Non-Communicable Diseases,’ a hand hygiene program in Spain targeted at children, and the innovative use of artificial intelligence in the prevention and treatment of gastrointestinal diseases. These efforts are anticipated to contribute positively to alleviating the disease burden^37–39^.

Furthermore, interventions targeting high-risk groups are crucial. Health education and early screening for the elderly and low-income groups should be strengthened, convenient medical services should be provided, and effective prevention and treatment strategies should be implemented. In high-development countries, promoting public health programs that encourage balanced diets and increased physical activity can effectively reduce the burden of nonmalignant upper gastrointestinal diseases. In low-development countries, continuing to strengthen *H. pylori* infection control measures is a significant step towards reducing the incidence of nonmalignant upper gastrointestinal diseases. Through these targeted interventions, the disease burden on high-risk groups can be effectively reduced, and the overall public health level can be improved.

This study presents the most comprehensive update on the global burden of nonmalignant upper gastrointestinal diseases over the last three decades, encompassing data from 204 countries and regions and illuminating previously unexplored aspects. Nonetheless, it faces several constraints. Firstly, despite the extensive data compilation, some estimates rely on projections or sparse data from adjacent countries, particularly in regions with low SDI. Secondly, the scope of nonmalignant upper gastrointestinal diseases in the GBD 2019 is confined to those with adequate global epidemiological data for burden assessment. Thirdly, methodological variations could influence estimate accuracy, and our analysis only partially mitigates this concern. Fourthly, disability definitions in this study are based on simplified disease descriptions, overlooking comorbidities and disease complexities, suggesting a need for further refinement and investigation. In conclusion, our findings highlight the critical need for enhanced disease screening, prevention, intervention strategies, and healthcare services, particularly in low-SDI regions. Future research should prioritize the acquisition of higher-quality epidemiological data and the application of uniform methods and definitions to refine the accuracy and dependability of global burden assessments for nonmalignant upper gastrointestinal diseases.

## Conclusion

Nonmalignant upper gastrointestinal diseases continue to be a significant component of the global disease burden, affecting past and future health landscapes. Despite a decrease in the global burden of these diseases over recent decades, notable regional disparities persist. The burden remains particularly high in low-SDI countries, where targeted interventions are urgently needed. The variation in disease burden across different sexes, and age groups underscores the importance of tailoring prevention and treatment strategies to meet the specific needs of these populations. To effectively manage future challenges posed by population growth and aging, public health interventions must account for these disparities, ensuring that resources are judiciously allocated and measures are precisely directed. Strengthening global and regional collaboration, enhancing the availability and accessibility of diagnostic, treatment, and prevention services, and crafting specialized health interventions for high-risk groups are essential steps toward mitigating the impact of nonmalignant upper gastrointestinal diseases.

## Ethical approval

Not applicable. This study involved the analysis of publicly available data from the Global Burden of Disease (GBD) study, which does not require ethical approval.

## Consent

Not applicable. This research utilized de-identified data from the GBD database, eliminating the need for individual patient or volunteer consent.

## Source of funding

Not applicable.

## Author contribution

Z.H.B. and H.W. conceived and designed the research. Z.H.B. analyzed data. C.S. and J.A. prepared figures. Z.H.B., H.W., and C.S. drafted the paper. Z.C.Y. and X.M.M. edited and revised the manuscript. All authors read and approved the final version of the manuscript.

## Conflicts of interest disclosure

The authors declare no conflicts of interest.

## Research registration unique identifying number (UIN)

Not applicable.

## Guarantor

Z.H.B. takes responsibility for the integrity of the work as a whole, from inception to published article.

## Data availability statement

The data used in this study can be derived from the GBD 2019 https://ghdx.healthdata.org/gbd-2019).

## Provenance and peer review

Not commissioned, externally peer-reviewed.

## Supplementary Material
