# Supplementary material for: The global, regional, and national patterns of change in the burden of nonmalignant upper gastrointestinal diseases from 1990 to 2019 and the forecast for the next decade
Source: Int J Surg. 2024 Jul 3;111(1):80–92. doi: 10.1097/JS9.0000000000001902 (PMC11745775; doi:10.1097/JS9.0000000000001902)
Supplement: Supplementary file 2 [file js9-111-0080-s002.pdf]

Table S1. Age-standardized DALYs rates of PUD, GD and GERD across all countries and territori

Location	PUD	GD
Afghanistan	255.08 (351.83 to 173.23)	28.78 (41.46 to 19.56)
Albania	19.73 (25.59 to 14.65)	24.16 (36.3 to 15.14)
Algeria	53.26 (68.01 to 40.99)	16.3 (23.92 to 10.36)
American Samoa	99.41 (119.73 to 80.21)	24.83 (35.77 to 16.81)
Andorra	11.93 (15.25 to 8.97)	16.33 (24.36 to 10.27)
Angola	147.89 (208.04 to 98.47)	93.92 (130.33 to 62.16)
Antigua and Barbuda	33.99 (40.86 to 27.75)	42.23 (56.82 to 31.09)
Argentina	35.36 (41.26 to 30.44)	18.48 (26.66 to 12.56)
Armenia	108.64 (128.77 to 90.49)	18.54 (25.22 to 13.23)
Australia	14 (16.19 to 11.95)	19.37 (29.19 to 12.24)
Austria	18.1 (20.67 to 15.59)	17.43 (25.84 to 11.35)
Azerbaijan	66.45 (87.63 to 52.12)	14.94 (21.8 to 9.58)
Bahamas	65.88 (84.79 to 50.71)	38.78 (52.43 to 28.22)
Bahrain	76.88 (93.54 to 62.41)	16.61 (23.67 to 11.26)
Bangladesh	36.49 (47.42 to 25.73)	30.78 (44.61 to 19.94)
Barbados	57.2 (71.38 to 45.87)	28.59 (40.3 to 19.63)
Belarus	49.1 (64.41 to 37.13)	18.52 (27 to 12.13)
Belgium	21.43 (24.65 to 18.52)	18.38 (27.62 to 11.54)
Belize	50.38 (60.84 to 40.85)	51.21 (67.97 to 37.41)
Benin	173.31 (230.9 to 128.16)	40.45 (55.23 to 28.56)
Bermuda	31.53 (39.97 to 24.78)	30.41 (43.91 to 20.71)
Bhutan	150.32 (280.11 to 77.12)	30.84 (43.42 to 20.75)
Bolivia (Plurinational State of)	119.39 (158.29 to 86.62)	54.95 (72.41 to 40.36)
Bosnia and Herzegovina	40.22 (50.33 to 31.95)	22.87 (33.17 to 14.72)
Botswana	151.98 (220.64 to 105.9)	72.54 (97.7 to 52.32)
Brazil	50.64 (54.72 to 46.87)	28.35 (39.28 to 20.41)
Brunei Darussalam	51.25 (60.24 to 43.5)	9.87 (13.66 to 6.93)
Bulgaria	59.61 (75.66 to 45.86)	34.46 (46.15 to 24.48)
Burkina Faso	127.84 (198.82 to 80.38)	37.23 (52.57 to 25.36)
Burundi	209.35 (354.13 to 118.82)	107.22 (159.26 to 68.96)
Cabo Verde	56.13 (67.76 to 45.9)	20.81 (29.38 to 14.04)
Cambodia	427.74 (562.93 to 330.42)	42.15 (56.79 to 29.37)
Cameroon	144.77 (203.6 to 92.03)	33.48 (46 to 22.99)
Canada	19.12 (22.04 to 16.42)	17.89 (25.7 to 12.05)
Central African Republic	377.18 (573.09 to 228.76)	162.44 (272.14 to 100.26)
Chad	254.67 (332.12 to 191.04)	53.7 (71.86 to 38.16)
Chile	22.73 (26.19 to 19.78)	18.13 (25.81 to 12.3)
China	45.34 (53.74 to 38.74)	44.27 (57.06 to 33.63)
Colombia	36.45 (47.89 to 27.87)	20.3 (26.93 to 15.1)
Comoros	119.54 (181.32 to 63.78)	90.92 (129.12 to 61.39)
Congo	121.71 (173.44 to 82.76)	83.19 (115.64 to 56.02)
Cook Islands	27.37 (33.95 to 22.24)	20.1 (30.11 to 12.72)
Costa Rica	40.2 (53.45 to 29.91)	13.65 (19.76 to 9.3)
Croatia	45.31 (57.83 to 35.81)	19.94 (27.89 to 13.72)
Cuba	43.23 (55.16 to 34.06)	39.74 (54.13 to 28.06)
Cyprus	19.79 (25.81 to 16.34)	11.34 (17.06 to 7.12)
Czechia	51.56 (62.98 to 41)	21.63 (31.47 to 14.29)
Côte d'Ivoire	161.4 (211.83 to 118.67)	36.42 (49.53 to 25.95)
Democratic People's Republic of Korea	144.72 (202.76 to 87.86)	50.57 (72.42 to 34.78)
Democratic Republic of the Congo	135.74 (188.39 to 92.5)	98.76 (135.45 to 66.53)
Denmark	47.94 (55.6 to 41.09)	17.62 (25.11 to 11.73)
Djibouti	108.87 (187.15 to 48.18)	71.7 (100.33 to 46.44)
Dominica	59.4 (76.59 to 45.12)	42.64 (58.08 to 31)
Dominican Republic	84.12 (113.34 to 55.85)	37.44 (52.88 to 25.96)
Ecuador	59.82 (78.04 to 46.46)	34.87 (45.64 to 25.96)
Egypt	62.27 (94.15 to 35.42)	15.83 (23.07 to 10.29)

El Salvador	85.07 (110.6 to 64.08)	17.72 (24.5 to 12.73)
Equatorial Guinea	78.41 (122.23 to 45.49)	68.79 (94.83 to 46.68)
Eritrea	186.56 (258.88 to 133.57)	115.97 (165.69 to 73.04)
Estonia	56.25 (73.15 to 42.9)	17.62 (25.75 to 11.54)
Eswatini	162.89 (233.76 to 108.63)	74.43 (100.23 to 54.52)
Ethiopia	89.3 (131.13 to 49.96)	76 (105.82 to 49.29)
Fiji	132.89 (172.69 to 101.69)	23.57 (34.18 to 15.54)
Finland	29.88 (35.24 to 25.65)	18.15 (24.97 to 12.73)
France	14.23 (16.27 to 12.33)	15.71 (23.87 to 9.69)
Gabon	86.42 (124.35 to 58.24)	63.79 (86.2 to 44.06)
Gambia	165.29 (220.24 to 118.95)	37.07 (49.98 to 25.68)
Georgia	83.89 (103.8 to 66.6)	17.37 (24.21 to 11.91)
Germany	28.98 (33.26 to 25.56)	19.61 (27.79 to 13.31)
Ghana	58.7 (76.23 to 44.1)	28.2 (40.07 to 19.53)
Greece	36.64 (42.03 to 31.55)	15.99 (23.98 to 9.89)
Greenland	189.82 (242.69 to 148.55)	18.65 (26.97 to 12.91)
Grenada	76.21 (90.67 to 63.61)	53.86 (68.89 to 41.23)
Guam	33.82 (41.75 to 26.68)	20.92 (31.56 to 12.99)
Guatemala	204.32 (258.92 to 160)	47.93 (60.81 to 37.64)
Guinea	193.97 (263.74 to 130.1)	42.94 (60.17 to 28.27)
Guinea-Bissau	292.77 (391.48 to 213.77)	53.18 (71.9 to 38.71)
Guyana	168.26 (220.76 to 126.37)	57.13 (74.79 to 42.93)
Haiti	226.71 (315.36 to 154.76)	75.44 (104.02 to 52.71)
Honduras	229.8 (301.77 to 166.69)	66.67 (92.71 to 46.12)
Hungary	79.25 (96.38 to 64.43)	25.75 (36.4 to 17.63)
Iceland	13.52 (15.88 to 11.4)	14.39 (20.97 to 9.38)
India	158.99 (189.45 to 134.9)	39.4 (55.25 to 27.56)
Indonesia	23.92 (27.45 to 19.6)	22.65 (33.66 to 14.41)
Iran (Islamic Republic of)	42.48 (47.09 to 36.91)	21.24 (30.77 to 14.14)
Iraq	25.12 (31.17 to 19.29)	16.76 (24.53 to 10.98)
Ireland	21.59 (25.49 to 18.19)	18.08 (26.59 to 12.07)
Israel	9.88 (11.84 to 8.43)	16.48 (24.51 to 10.3)
Italy	11.06 (12.15 to 9.8)	13.97 (20.07 to 9.43)
Jamaica	74.96 (95.35 to 57.34)	37.02 (51.67 to 26.45)
Japan	22.71 (25.75 to 19.88)	7.2 (10.53 to 4.68)
Jordan	31.07 (37.28 to 25.22)	13.43 (20.19 to 8.49)
Kazakhstan	75.53 (91.21 to 62.9)	17.64 (24.85 to 11.75)
Kenya	128.65 (188.91 to 75.73)	93.79 (127.09 to 62.69)
Kiribati	512.71 (701.02 to 362.22)	32.78 (45.19 to 22.58)
Kuwait	21.84 (27.74 to 17.33)	14.01 (21.14 to 8.82)
Kyrgyzstan	57.47 (68.62 to 47.92)	16.25 (23.95 to 10.43)
Lao People's Democratic Republic	397.46 (545.23 to 282.9)	41.59 (58.32 to 27.38)
Latvia	70.36 (88.19 to 56.51)	20.89 (29.34 to 14.31)
Lebanon	42.35 (66.39 to 25.19)	16.66 (24.66 to 10.78)
Lesotho	315.04 (427.66 to 224.71)	102.81 (137.59 to 71.9)
Liberia	152.8 (217.63 to 100.32)	36.44 (50.91 to 25.04)
Libya	53.49 (76.15 to 35.49)	16.85 (24.65 to 11.02)
Lithuania	107.64 (137.12 to 84.79)	21.12 (29.58 to 14.52)
Luxembourg	16.77 (19.82 to 13.96)	24.75 (37.41 to 15.65)
Madagascar	114.8 (153.01 to 80.67)	87.05 (116.62 to 58.69)
Malawi	114.71 (165.25 to 71.3)	87.11 (118.47 to 62.43)
Malaysia	104.8 (131.77 to 78.93)	19.1 (28.02 to 12.2)
Maldives	26.33 (31.76 to 21.55)	22.86 (34.97 to 13.93)
Mali	211.39 (321.76 to 125.03)	49.49 (69.68 to 33.41)
Malta	19.48 (23.12 to 16.28)	28.11 (41.17 to 18.82)
Marshall Islands	246.77 (345.24 to 162.32)	26.57 (37.54 to 17.37)
Mauritania	102.66 (137.14 to 69.96)	31.93 (45.17 to 22.31)
Mauritius	49.55 (61.97 to 38.91)	22.65 (31.45 to 16.04)

Mexico	66.07 (76.29 to 56.15)	32.34 (39.15 to 27.29)
Micronesia (Federated States of)	237.53 (337.8 to 148.65)	27.18 (37.98 to 17.9)
Monaco	14.03 (17.31 to 10.8)	15.87 (23.87 to 9.82)
Mongolia	213.32 (289.88 to 161.84)	29.99 (40.66 to 21.6)
Montenegro	46.14 (56.2 to 37.89)	22.54 (33.93 to 14.01)
Morocco	76.22 (94.52 to 57.95)	18.57 (26.56 to 12.36)
Mozambique	144.33 (208.87 to 97.72)	92.7 (134.95 to 60.89)
Myanmar	165.5 (226.29 to 129.51)	32.79 (45.92 to 22.65)
Namibia	136.52 (190.35 to 99.07)	66.51 (88.62 to 47.48)
Nauru	229.91 (314.87 to 161.9)	26.07 (37.16 to 17.28)
Nepal	27.97 (39.19 to 21.27)	29.08 (43.25 to 18.68)
Netherlands	15.15 (17.49 to 13.03)	16.6 (24.64 to 10.52)
New Zealand	18.32 (21.05 to 15.73)	23.04 (33.01 to 15.3)
Nicaragua	57.75 (74.74 to 46.52)	27.21 (34.61 to 20.62)
Niger	257.77 (384.56 to 150.3)	56.62 (82.36 to 36.45)
Nigeria	171.11 (301.72 to 97.14)	42.23 (63.78 to 28.47)
Niue	109.12 (141.81 to 80.34)	22.88 (33.11 to 15.05)
North Macedonia	41.38 (51.35 to 33.14)	21.42 (31.97 to 13.42)
Northern Mariana Islands	72.98 (87.05 to 59.45)	20.83 (30.83 to 13.21)
Norway	30.39 (35.47 to 26.96)	24.42 (36.52 to 16.03)
Oman	39.72 (48.96 to 32.46)	15.27 (22.89 to 9.45)
Pakistan	98.1 (132.19 to 74.53)	32.61 (46.51 to 21.87)
Palau	101.99 (133.54 to 64)	31.06 (43.02 to 21.53)
Palestine	30.16 (35.69 to 25.18)	17.74 (26.49 to 11.52)
Panama	24.37 (32.66 to 18.27)	15.56 (21.85 to 10.97)
Papua New Guinea	185.91 (243.91 to 142.86)	30.74 (44.75 to 19.93)
Paraguay	46.82 (62.3 to 35.63)	29.74 (43.52 to 19.53)
Peru	43.65 (59.3 to 31.6)	57.27 (76.33 to 42.18)
Philippines	245.46 (295.16 to 203.15)	31.72 (43.3 to 22.83)
Poland	75.36 (88.73 to 62.9)	41.72 (58.3 to 28.58)
Portugal	19.11 (22.19 to 16.4)	16.65 (24.84 to 10.57)
Puerto Rico	14.51 (18.83 to 11.06)	26.63 (40.07 to 16.85)
Qatar	28.54 (36.12 to 22.48)	16.03 (23.07 to 10.8)
Republic of Korea	20.7 (24.21 to 17.17)	8.66 (12.44 to 5.83)
Republic of Moldova	101.32 (121.02 to 83.36)	19.86 (29.56 to 12.48)
Romania	43.35 (52.7 to 34.95)	30.33 (42.05 to 21.61)
Russian Federation	106.1 (122.43 to 91.55)	23.67 (33.04 to 16.96)
Rwanda	161.08 (256.27 to 105.23)	87.49 (121.61 to 60.57)
Saint Kitts and Nevis	41.39 (53.33 to 30.97)	43.79 (58.98 to 31.22)
Saint Lucia	51.7 (63.82 to 41.91)	37.41 (51.38 to 26.34)
Saint Vincent and the Grenadines	94.34 (115.37 to 75.65)	78.45 (99.24 to 61.58)
Samoa	173.41 (225.62 to 130.22)	22.47 (32.41 to 14.68)
San Marino	12.61 (17.46 to 8.83)	16.73 (24.79 to 10.66)
Sao Tome and Principe	68.41 (111.11 to 48.06)	22.41 (32.24 to 15.25)
Saudi Arabia	34.19 (44.8 to 26.57)	13.88 (20.43 to 8.87)
Senegal	160.48 (234.31 to 96.4)	36.52 (50.82 to 24.12)
Serbia	83 (102.41 to 66.58)	23.11 (32.48 to 16.25)
Seychelles	155.87 (186.97 to 126.2)	28.27 (38.93 to 19.5)
Sierra Leone	199.65 (282.76 to 131.59)	42.77 (58.52 to 29.31)
Singapore	17.4 (20.42 to 14.36)	7.43 (11.24 to 4.74)
Slovakia	67.15 (88.38 to 50.91)	26.76 (37.76 to 18.54)
Slovenia	31.34 (42.59 to 23.8)	24.19 (35.36 to 15.55)
Solomon Islands	254.69 (332.14 to 194.32)	28.21 (40.26 to 18.82)
Somalia	208.97 (354.41 to 106.88)	108.6 (178.44 to 64.8)
South Africa	83.84 (93.63 to 74.18)	70.63 (87.57 to 57.09)
South Sudan	114.26 (211 to 61.82)	84.87 (126.24 to 54.65)
Spain	12.9 (14.83 to 11.18)	16.57 (24.27 to 10.27)
Sri Lanka	11.17 (14.98 to 8.5)	28.93 (40.36 to 20.42)

Sudan	91.51 (143.97 to 52.72)	23.55 (34.47 to 15.59)
Suriname	107.23 (131.43 to 85.93)	42.8 (57.05 to 31.97)
Sweden	27.71 (31.04 to 24.46)	12.92 (18.88 to 8.38)
Switzerland	15.44 (18.82 to 12.61)	15.27 (22.05 to 10.12)
Syrian Arab Republic	20.77 (27.32 to 15.42)	16.14 (23.58 to 10.48)
Taiwan (Province of China)	38.31 (49.08 to 29.52)	25.45 (38.2 to 15.61)
Tajikistan	91.66 (118.53 to 72.6)	19.91 (28.11 to 13.69)
Thailand	34.11 (46.45 to 25.72)	26.34 (37.7 to 17.99)
Timor-Leste	348.16 (521.47 to 228.91)	42.77 (66.2 to 26.93)
Togo	185.76 (249.64 to 128.38)	43.63 (60.36 to 30.48)
Tokelau	116.3 (148.81 to 87.01)	24.06 (34.37 to 15.94)
Tonga	182.04 (233.31 to 136.79)	26.24 (38.06 to 17.28)
Trinidad and Tobago	87.49 (118.97 to 62.4)	73.85 (95.5 to 55.33)
Tunisia	42.36 (58.64 to 30.51)	16.06 (23.71 to 10.5)
Turkey	25.87 (31.33 to 21.12)	16 (23.7 to 10.14)
Turkmenistan	95.68 (121.5 to 73.53)	16.16 (23.59 to 10.65)
Tuvalu	185.03 (255.98 to 128.29)	26.14 (36.77 to 17.33)
Uganda	125.63 (201.97 to 89.1)	81.52 (111.76 to 56.07)
Ukraine	105.56 (130.06 to 85.76)	22.68 (32.84 to 15.47)
United Arab Emirates	46.69 (64.96 to 32.83)	13.93 (20.7 to 8.9)
United Kingdom	40.31 (43.58 to 37.36)	24.35 (33.93 to 17)
United Republic of Tanzania	64.99 (97.62 to 37.55)	56 (76.02 to 39.09)
United States of America	20.8 (23.03 to 18.77)	17.32 (24.36 to 11.58)
United States Virgin Islands	38.96 (48.98 to 30.08)	32.2 (45.73 to 22.27)
Uruguay	26.99 (31.84 to 22.57)	17.85 (25.36 to 11.96)
Uzbekistan	107.62 (128.4 to 88.46)	15.53 (23.18 to 9.67)
Vanuatu	293.42 (406.35 to 209.17)	31.46 (44.93 to 21.33)
Venezuela (Bolivarian Republic of)	51.22 (69.42 to 38.23)	26.1 (34.02 to 19.52)
Viet Nam	17.11 (22.23 to 13.05)	20.44 (30.85 to 13.03)
Yemen	159.2 (235.82 to 109.32)	26.92 (38.06 to 18.36)
Zambia	97.22 (135.26 to 73.75)	72.38 (96.29 to 52.45)
Zimbabwe	219.55 (309.66 to 121.51)	80.27 (112.23 to 53.22)

ies in 2019.

GRED
92.21 (167.08 to 47.65)
50.67 (92.11 to 25.97)
93.66 (169.01 to 48.63)
40.5 (74.2 to 20.66)
60.04 (109.03 to 30.75)
84.83 (151.86 to 44.07)
122.96 (217.33 to 63.84)
105.14 (187.94 to 54.26)
80.92 (147.23 to 41.33)
65.36 (118.9 to 33.27)
78.52 (140.19 to 40.18)
80.87 (146.52 to 41.37)
123.09 (217.48 to 64.04)
91.14 (164.49 to 46.92)
100.18 (179.08 to 52.01)
123.24 (218.35 to 64.16)
87.13 (156.14 to 44.48)
65.6 (117.89 to 33.14)
123 (218.39 to 63.83)
85.06 (152.23 to 43.87)
123.42 (218.38 to 64.09)
100.27 (182.11 to 51.81)
122.87 (218.07 to 63.88)
74.24 (133.88 to 37.97)
84.1 (150.21 to 43.77)
124.74 (222.06 to 64.6)
49.83 (90.21 to 25.58)
74.47 (134.13 to 38.12)
85.21 (152 to 44.09)
84.44 (150.92 to 43.59)
85.18 (152.87 to 43.99)
40.92 (74.92 to 20.81)
84.82 (151.72 to 43.83)
55.3 (100.28 to 28.34)
84.43 (150.61 to 43.87)
84.71 (151.35 to 43.81)
105.03 (187.75 to 53.95)
34.94 (63.02 to 17.73)
123.23 (218.22 to 63.88)
85.1 (152.4 to 44.13)
84.62 (150.79 to 43.94)
40.72 (74.13 to 20.95)
123.1 (218.52 to 64.09)
74.25 (133.59 to 37.85)
122.85 (217.61 to 64.23)
60.23 (108.84 to 30.83)
74.09 (133.37 to 38.08)
84.61 (151.08 to 44.1)
36.24 (65.15 to 18.5)
84.51 (150.49 to 43.89)
74.28 (132.3 to 38.37)
84.86 (150.7 to 43.81)
122.64 (217.36 to 63.77)
123.03 (217.58 to 63.96)
123.09 (217.87 to 63.94)
93.37 (168.4 to 48.28)

122.96 (217.11 to 63.99)
84.6 (151.22 to 43.77)
84.62 (151.77 to 43.68)
87 (155.83 to 44.46)
84.01 (149.39 to 43.55)
88.06 (158.29 to 45.23)
40.52 (74.46 to 20.47)
80.06 (143.9 to 41.03)
53.16 (95.68 to 26.92)
84.65 (151.12 to 43.69)
84.84 (151.64 to 43.81)
80.73 (146.78 to 41.35)
58.97 (105.58 to 29.97)
85.17 (152.31 to 44.29)
78.79 (144.24 to 40.5)
62.32 (112.36 to 32.08)
122.67 (217.15 to 63.77)
40.79 (74.4 to 20.73)
122.65 (216.19 to 63.88)
85.02 (152.12 to 44.2)
84.94 (151.8 to 44.25)
122.06 (216.09 to 63.61)
122.34 (216.22 to 63.92)
122.89 (217.2 to 63.78)
77.94 (139.24 to 39.82)
48.26 (87.58 to 24.27)
102.26 (184.31 to 53.1)
42.69 (76.51 to 21.62)
88.01 (158.39 to 44.53)
93.1 (168.51 to 48.02)
60.19 (109.23 to 30.52)
64.63 (116.78 to 32.81)
74.71 (134.16 to 38.33)
122.96 (217.35 to 64.16)
45.89 (83.33 to 23.33)
93.09 (168.08 to 48.03)
80.7 (145.69 to 41.32)
88.02 (158.02 to 45.12)
40.72 (74.69 to 20.81)
92.75 (168.06 to 48.03)
81.01 (146.37 to 41.34)
40.85 (74.8 to 20.91)
87.02 (155.7 to 44.59)
93.84 (169.69 to 48.6)
83.97 (149.43 to 43.64)
84.12 (149.83 to 43.73)
93.07 (168.06 to 48.21)
91.96 (165.83 to 47.19)
60 (109.14 to 30.51)
84.91 (151.91 to 43.73)
84.8 (151.79 to 43.98)
40.77 (75.49 to 20.94)
40.6 (74.48 to 20.61)
84.97 (152.49 to 43.95)
60.1 (109.14 to 30.69)
40.43 (74.34 to 20.77)
85.2 (152.01 to 44.23)
40.66 (74.71 to 20.69)

122.74 (220.76 to 64.16)
40.66 (74.38 to 20.78)
60.22 (108.75 to 30.58)
80.89 (147.09 to 41.37)
74.39 (134.22 to 38.05)
93.56 (169.11 to 48.18)
84.46 (150.74 to 43.93)
40.92 (74.95 to 21)
84.45 (151.24 to 43.85)
40.75 (74.28 to 20.76)
100.46 (181.31 to 51.93)
43.35 (78.99 to 22.02)
74.01 (133.73 to 37.79)
122.9 (217.16 to 64.02)
85.11 (152.15 to 44.32)
88.18 (158.46 to 45.28)
40.58 (74.63 to 20.91)
74.33 (134.38 to 38.31)
40.66 (74.69 to 20.55)
39.1 (71.24 to 19.98)
91.09 (165.1 to 46.6)
102.28 (185.09 to 52.44)
40.41 (73.88 to 20.58)
93.14 (168.18 to 48.33)
122.93 (217.39 to 64.06)
40.43 (73.6 to 20.54)
125.91 (223.26 to 65.11)
123.26 (218.57 to 64.08)
42.73 (76.63 to 21.68)
105.34 (185.83 to 53.64)
65.16 (115.74 to 33.15)
122.96 (217.75 to 63.89)
88.75 (160.37 to 45.71)
52.51 (94.45 to 26.67)
86.99 (154.88 to 44.31)
74.51 (135 to 38.09)
86.19 (157.49 to 43.95)
84.81 (152.08 to 43.99)
122.76 (217.32 to 63.87)
122.59 (216.58 to 63.82)
122.64 (217.49 to 63.87)
40.68 (74.96 to 20.66)
60.33 (109.56 to 30.73)
85.05 (151.63 to 44)
91.6 (164.95 to 47.6)
84.91 (151.92 to 44.04)
74.33 (134.55 to 38.08)
40.7 (74.85 to 20.85)
84.85 (151.72 to 43.9)
56.94 (101.88 to 29.19)
74.33 (134.35 to 38.13)
74.25 (133.8 to 37.97)
40.62 (74.24 to 20.59)
84.62 (150.69 to 43.81)
87.46 (156.38 to 45.04)
84.09 (149.57 to 43.5)
54.06 (97.4 to 27.5)
40.85 (74.64 to 20.85)

93.45 (169.37 to 48.38)
122.52 (216.27 to 63.63)
55 (99.92 to 28.02)
35.84 (65.01 to 18.35)
93.73 (169.47 to 48.28)
40.02 (71.77 to 20.29)
80.75 (145.44 to 41.31)
41.01 (75.29 to 21.03)
40.71 (74.91 to 20.8)
85.21 (151.9 to 44.05)
40.68 (74.66 to 20.68)
40.82 (74.4 to 20.78)
122.68 (217.85 to 63.84)
93.89 (169.17 to 48.29)
109.67 (198.02 to 57.2)
80.93 (146.86 to 41.25)
40.65 (73.97 to 20.79)
84.93 (151.64 to 44.11)
89.79 (163.81 to 46.08)
89.59 (161.86 to 46.3)
79.31 (142.97 to 40.78)
84.85 (151.18 to 43.98)
73.5 (131.47 to 37.17)
123.2 (218.49 to 63.97)
105.19 (187.87 to 54.32)
80.83 (145.85 to 41.4)
40.59 (73.86 to 20.78)
123.03 (217.04 to 63.74)
41.08 (75.32 to 21.03)
93.27 (169.35 to 47.96)
84.55 (150.77 to 43.64)
84.63 (150.81 to 43.91)
