# Supplementary material for: The global, regional, and national patterns of change in the burden of nonmalignant upper gastrointestinal diseases from 1990 to 2019 and the forecast for the next decade
Source: Int J Surg. 2024 Jul 3;111(1):80–92. doi: 10.1097/JS9.0000000000001902 (PMC11745775; doi:10.1097/JS9.0000000000001902)
Supplement: Supplementary file 3 [file js9-111-0080-s003.pdf]

Table S2. DALYs rates of PUD, GD and GERD globally in 1990 and 2019, categorized by age and sex.

Sex	Age	Cause	Year	Val	Upper	Lower
Male	<5 years	Peptic ulcer disease	1990	30.288671	58.709329	15.874139
Female	<5 years	Peptic ulcer disease	1990	42.623708	65.701212	22.432866
Male	5-9 years	Peptic ulcer disease	1990	19.886621	26.686848	15.204016
Female	5-9 years	Peptic ulcer disease	1990	23.504304	30.758453	15.957523
Male	10-14 years	Peptic ulcer disease	1990	13.700841	17.011055	11.346037
Female	10-14 years	Peptic ulcer disease	1990	17.528798	21.654558	13.681213
Male	15-19 years	Peptic ulcer disease	1990	44.128071	51.938987	37.518818
Female	15-19 years	Peptic ulcer disease	1990	42.843027	54.288085	33.61409
Male	20-24 years	Peptic ulcer disease	1990	60.270645	72.043425	52.602295
Female	20-24 years	Peptic ulcer disease	1990	63.213254	79.71117	47.955365
Male	25-29 years	Peptic ulcer disease	1990	95.320927	109.79314	83.251945
Female	25-29 years	Peptic ulcer disease	1990	67.836293	85.873286	51.681274
Male	30-34 years	Peptic ulcer disease	1990	121.47113	138.02979	105.61195
Female	30-34 years	Peptic ulcer disease	1990	71.330591	88.307535	55.489103
Male	35-39 years	Peptic ulcer disease	1990	197.66156	223.49138	169.38042
Female	35-39 years	Peptic ulcer disease	1990	97.736127	122.11498	78.748825
Male	40-44 years	Peptic ulcer disease	1990	268.59253	305.0032	231.40075
Female	40-44 years	Peptic ulcer disease	1990	101.72722	123.51388	85.396986
Male	45-49 years	Peptic ulcer disease	1990	371.85452	418.01893	320.05231
Female	45-49 years	Peptic ulcer disease	1990	153.33834	181.09732	131.06577
Male	50-54 years	Peptic ulcer disease	1990	479.28336	537.42691	417.13578
Female	50-54 years	Peptic ulcer disease	1990	214.59913	254.90644	179.05473
Male	55-59 years	Peptic ulcer disease	1990	594.94304	660.97133	523.48454
Female	55-59 years	Peptic ulcer disease	1990	265.91526	318.62238	228.39945
Male	60-64 years	Peptic ulcer disease	1990	706.19423	786.44827	621.45445
Female	60-64 years	Peptic ulcer disease	1990	340.86107	396.8618	291.09297
Male	65-69 years	Peptic ulcer disease	1990	818.71771	905.44991	723.18391
Female	65-69 years	Peptic ulcer disease	1990	435.42172	504.99198	373.45982
Male	70-74 years	Peptic ulcer disease	1990	980.5464	1083.5873	870.94622
Female	70-74 years	Peptic ulcer disease	1990	563.53158	648.78839	485.92181
Male	75-79 years	Peptic ulcer disease	1990	1113.1962	1223.3713	997.6084
Female	75-79 years	Peptic ulcer disease	1990	658.3446	737.83015	581.64021
Male	80-84 years	Peptic ulcer disease	1990	1129.6673	1238.1938	1000.8363
Female	80-84 years	Peptic ulcer disease	1990	792.53734	887.49246	696.48528
Male	85-89 years	Peptic ulcer disease	1990	1235.4692	1349.1252	1098.5468
Female	85-89 years	Peptic ulcer disease	1990	933.12141	1050.675	803.21839
Male	90-94 years	Peptic ulcer disease	1990	1173.8585	1291.463	995.99682
Female	90-94 years	Peptic ulcer disease	1990	971.17788	1109.5603	798.10807
Male	95+ years	Peptic ulcer disease	1990	1139.3327	1265.1244	929.68668
Female	95+ years	Peptic ulcer disease	1990	984.72127	1134.5108	759.33049
Male	<5 years	Gastritis and duodenitis	1990	23.898839	34.61455	14.608166
Female	<5 years	Gastritis and duodenitis	1990	12.923732	19.87898	7.7944755
Male	5-9 years	Gastritis and duodenitis	1990	12.265663	19.611122	7.1528531
Female	5-9 years	Gastritis and duodenitis	1990	13.914104	22.037027	7.8528711
Male	10-14 years	Gastritis and duodenitis	1990	9.9355396	17.744593	5.4100587
Female	10-14 years	Gastritis and duodenitis	1990	12.716783	22.462942	7.1419849
Male	15-19 years	Gastritis and duodenitis	1990	13.337228	21.109507	8.2961207
Female	15-19 years	Gastritis and duodenitis	1990	18.367216	32.167659	10.305807
Male	20-24 years	Gastritis and duodenitis	1990	16.024416	24.373417	10.517315
Female	20-24 years	Gastritis and duodenitis	1990	26.052076	41.656026	15.517082
Male	25-29 years	Gastritis and duodenitis	1990	23.748522	34.550431	15.674949
Female	25-29 years	Gastritis and duodenitis	1990	29.186631	47.369868	16.13102
Male	30-34 years	Gastritis and duodenitis	1990	34.31108	50.775913	22.566536
Female	30-34 years	Gastritis and duodenitis	1990	37.50323	62.923352	21.634174
Male	35-39 years	Gastritis and duodenitis	1990	47.208286	69.295772	31.993606
Female	35-39 years	Gastritis and duodenitis	1990	45.927647	72.315019	27.196967

Male	40-44 years	Gastritis and duodenitis	1990	63.562135	93.659056	43.211361
Female	40-44 years	Gastritis and duodenitis	1990	52.207013	87.478351	29.906171
Male	45-49 years	Gastritis and duodenitis	1990	83.987095	124.66139	57.831602
Female	45-49 years	Gastritis and duodenitis	1990	64.579315	107.18414	38.846224
Male	50-54 years	Gastritis and duodenitis	1990	101.60386	146.66719	70.478741
Female	50-54 years	Gastritis and duodenitis	1990	77.971771	122.26209	48.576227
Male	55-59 years	Gastritis and duodenitis	1990	120.83606	175.29152	84.443179
Female	55-59 years	Gastritis and duodenitis	1990	91.805541	142.1963	58.60678
Male	60-64 years	Gastritis and duodenitis	1990	125.07492	178.9976	85.493342
Female	60-64 years	Gastritis and duodenitis	1990	102.86105	152.03874	68.542194
Male	65-69 years	Gastritis and duodenitis	1990	140.49532	194.60621	96.746594
Female	65-69 years	Gastritis and duodenitis	1990	119.81463	171.09287	81.346826
Male	70-74 years	Gastritis and duodenitis	1990	156.29367	221.26264	106.95511
Female	70-74 years	Gastritis and duodenitis	1990	128.26524	177.57621	89.870158
Male	75-79 years	Gastritis and duodenitis	1990	160.39857	220.87012	113.99867
Female	75-79 years	Gastritis and duodenitis	1990	124.89108	168.14367	91.736655
Male	80-84 years	Gastritis and duodenitis	1990	159.05696	221.47311	116.61461
Female	80-84 years	Gastritis and duodenitis	1990	134.3899	174.27046	100.07654
Male	85-89 years	Gastritis and duodenitis	1990	173.72533	237.44833	133.79903
Female	85-89 years	Gastritis and duodenitis	1990	139.66259	176.80917	105.02435
Male	90-94 years	Gastritis and duodenitis	1990	142.54113	183.90039	111.94164
Female	90-94 years	Gastritis and duodenitis	1990	146.5472	178.64477	115.50171
Male	95+ years	Gastritis and duodenitis	1990	141.91854	179.86945	105.67139
Female	95+ years	Gastritis and duodenitis	1990	157.61541	189.63961	122.25993
Male	<5 years	Gastroesophageal reflux disease	1990	0	0	0
Female	<5 years	Gastroesophageal reflux disease	1990	0	0	0
Male	5-9 years	Gastroesophageal reflux disease	1990	0.1797567	0.3807813	0.0672393
Female	5-9 years	Gastroesophageal reflux disease	1990	0.186185	0.3920885	0.0713774
Male	10-14 years	Gastroesophageal reflux disease	1990	4.0478191	7.7076784	1.8039268
Female	10-14 years	Gastroesophageal reflux disease	1990	4.3886558	8.1964573	1.9405837
Male	15-19 years	Gastroesophageal reflux disease	1990	23.362214	44.774802	11.372992
Female	15-19 years	Gastroesophageal reflux disease	1990	25.430539	48.353555	12.457237
Male	20-24 years	Gastroesophageal reflux disease	1990	57.498268	112.747	28.038136
Female	20-24 years	Gastroesophageal reflux disease	1990	62.123488	121.37434	30.777548
Male	25-29 years	Gastroesophageal reflux disease	1990	83.539855	156.60743	39.156855
Female	25-29 years	Gastroesophageal reflux disease	1990	89.713435	167.50334	42.086398
Male	30-34 years	Gastroesophageal reflux disease	1990	99.470407	187.05781	46.65066
Female	30-34 years	Gastroesophageal reflux disease	1990	106.88463	196.97503	50.1042
Male	35-39 years	Gastroesophageal reflux disease	1990	103.18123	195.05473	47.476569
Female	35-39 years	Gastroesophageal reflux disease	1990	110.74459	207.86062	50.820049
Male	40-44 years	Gastroesophageal reflux disease	1990	111.114	200.59872	52.604476
Female	40-44 years	Gastroesophageal reflux disease	1990	118.58788	214.82796	56.095319
Male	45-49 years	Gastroesophageal reflux disease	1990	118.96426	223.42422	57.642183
Female	45-49 years	Gastroesophageal reflux disease	1990	126.58389	234.31549	62.323203
Male	50-54 years	Gastroesophageal reflux disease	1990	122.80593	226.53269	59.88356
Female	50-54 years	Gastroesophageal reflux disease	1990	132.95442	242.94726	65.811965
Male	55-59 years	Gastroesophageal reflux disease	1990	125.12169	237.1057	58.65533
Female	55-59 years	Gastroesophageal reflux disease	1990	136.54113	257.30746	63.823671
Male	60-64 years	Gastroesophageal reflux disease	1990	130.24921	246.44062	61.585682
Female	60-64 years	Gastroesophageal reflux disease	1990	143.5789	273.22506	68.235914
Male	65-69 years	Gastroesophageal reflux disease	1990	133.16447	246.28304	66.423221
Female	65-69 years	Gastroesophageal reflux disease	1990	145.98602	270.7596	73.160542
Male	70-74 years	Gastroesophageal reflux disease	1990	137.17031	247.03742	68.21077
Female	70-74 years	Gastroesophageal reflux disease	1990	146.72996	265.71681	72.202785
Male	75-79 years	Gastroesophageal reflux disease	1990	136.88999	244.64924	69.268498
Female	75-79 years	Gastroesophageal reflux disease	1990	143.51795	259.29766	72.703068
Male	80-84 years	Gastroesophageal reflux disease	1990	132.26452	237.26997	66.097775
Female	80-84 years	Gastroesophageal reflux disease	1990	134.39225	241.52903	67.422497

Male	85-89 years	Gastroesophageal reflux disease	1990	120.79139	220.07075	59.255843
Female	85-89 years	Gastroesophageal reflux disease	1990	119.93134	219.55803	59.39552
Male	90-94 years	Gastroesophageal reflux disease	1990	113.94537	202.23191	57.152243
Female	90-94 years	Gastroesophageal reflux disease	1990	109.29508	197.83448	55.145393
Male	95+ years	Gastroesophageal reflux disease	1990	112.95679	199.4938	55.324937
Female	95+ years	Gastroesophageal reflux disease	1990	107.20612	192.22594	52.369397
Male	<5 years	Peptic ulcer disease	2019	9.0056911	22.166927	4.4216469
Female	<5 years	Peptic ulcer disease	2019	18.904055	34.450776	11.722525
Male	5-9 years	Peptic ulcer disease	2019	7.1477839	9.618523	5.1893112
Female	5-9 years	Peptic ulcer disease	2019	9.8102312	13.660031	7.1000932
Male	10-14 years	Peptic ulcer disease	2019	6.0921449	7.5312892	4.8433421
Female	10-14 years	Peptic ulcer disease	2019	10.792764	13.569121	8.5043205
Male	15-19 years	Peptic ulcer disease	2019	19.879398	23.809186	16.595845
Female	15-19 years	Peptic ulcer disease	2019	18.3812	23.273726	14.729431
Male	20-24 years	Peptic ulcer disease	2019	31.701858	37.327629	26.8194
Female	20-24 years	Peptic ulcer disease	2019	32.117612	39.441135	25.979284
Male	25-29 years	Peptic ulcer disease	2019	45.218198	53.125579	39.597866
Female	25-29 years	Peptic ulcer disease	2019	30.595123	36.996738	25.455257
Male	30-34 years	Peptic ulcer disease	2019	50.257041	58.264846	44.559219
Female	30-34 years	Peptic ulcer disease	2019	32.222595	38.154877	26.870955
Male	35-39 years	Peptic ulcer disease	2019	73.356473	86.467543	64.51683
Female	35-39 years	Peptic ulcer disease	2019	42.842582	51.407395	35.99791
Male	40-44 years	Peptic ulcer disease	2019	91.210813	105.79658	79.48152
Female	40-44 years	Peptic ulcer disease	2019	43.375875	51.56452	36.970418
Male	45-49 years	Peptic ulcer disease	2019	120.28386	141.01652	104.77351
Female	45-49 years	Peptic ulcer disease	2019	57.266169	68.127725	48.89929
Male	50-54 years	Peptic ulcer disease	2019	151.39514	172.20213	133.31176
Female	50-54 years	Peptic ulcer disease	2019	86.331587	103.18252	70.819849
Male	55-59 years	Peptic ulcer disease	2019	194.3844	221.452	172.58565
Female	55-59 years	Peptic ulcer disease	2019	104.4982	121.16768	89.657148
Male	60-64 years	Peptic ulcer disease	2019	229.87943	259.60334	207.02977
Female	60-64 years	Peptic ulcer disease	2019	142.06729	162.06247	124.8485
Male	65-69 years	Peptic ulcer disease	2019	276.62922	313.33546	246.94159
Female	65-69 years	Peptic ulcer disease	2019	193.3444	220.46096	170.75615
Male	70-74 years	Peptic ulcer disease	2019	338.84863	382.86206	305.3914
Female	70-74 years	Peptic ulcer disease	2019	244.98408	274.81625	217.18514
Male	75-79 years	Peptic ulcer disease	2019	435.0232	491.71445	393.94545
Female	75-79 years	Peptic ulcer disease	2019	313.21324	348.84593	281.08428
Male	80-84 years	Peptic ulcer disease	2019	493.3108	557.87709	442.70175
Female	80-84 years	Peptic ulcer disease	2019	413.55357	461.02046	357.78012
Male	85-89 years	Peptic ulcer disease	2019	577.96226	648.37592	510.99665
Female	85-89 years	Peptic ulcer disease	2019	460.22408	522.04563	379.73172
Male	90-94 years	Peptic ulcer disease	2019	570.16425	640.49955	478.47355
Female	90-94 years	Peptic ulcer disease	2019	498.93457	572.4799	394.06482
Male	95+ years	Peptic ulcer disease	2019	584.85428	670.98897	463.76517
Female	95+ years	Peptic ulcer disease	2019	554.83888	655.10031	420.15967
Male	<5 years	Gastritis and duodenitis	2019	15.743562	24.592954	10.060783
Female	<5 years	Gastritis and duodenitis	2019	6.7693222	10.969255	3.9716503
Male	5-9 years	Gastritis and duodenitis	2019	9.0883398	15.047741	4.8968051
Female	5-9 years	Gastritis and duodenitis	2019	10.451682	17.117942	5.8464676
Male	10-14 years	Gastritis and duodenitis	2019	8.9463389	16.403896	4.6916109
Female	10-14 years	Gastritis and duodenitis	2019	11.747212	21.385195	6.2547311
Male	15-19 years	Gastritis and duodenitis	2019	11.928617	19.861887	7.1410256
Female	15-19 years	Gastritis and duodenitis	2019	16.092809	28.232078	9.1140724
Male	20-24 years	Gastritis and duodenitis	2019	14.748839	23.204725	9.1160295
Female	20-24 years	Gastritis and duodenitis	2019	25.438793	42.478095	14.525817
Male	25-29 years	Gastritis and duodenitis	2019	20.047242	30.158791	13.059115
Female	25-29 years	Gastritis and duodenitis	2019	25.018176	42.654633	13.36259

Male	30-34 years	Gastritis and duodenitis	2019	26.111106	39.252997	17.056038
Female	30-34 years	Gastritis and duodenitis	2019	30.086499	52.366222	16.679459
Male	35-39 years	Gastritis and duodenitis	2019	32.357833	49.217928	21.028185
Female	35-39 years	Gastritis and duodenitis	2019	36.493325	59.827196	20.735446
Male	40-44 years	Gastritis and duodenitis	2019	41.596372	65.363334	27.331501
Female	40-44 years	Gastritis and duodenitis	2019	38.767991	68.99441	21.312226
Male	45-49 years	Gastritis and duodenitis	2019	52.929475	81.846557	35.295649
Female	45-49 years	Gastritis and duodenitis	2019	48.927914	84.807812	27.988063
Male	50-54 years	Gastritis and duodenitis	2019	61.458782	90.709422	41.70122
Female	50-54 years	Gastritis and duodenitis	2019	60.492805	99.381645	35.33323
Male	55-59 years	Gastritis and duodenitis	2019	68.507836	99.566311	47.519497
Female	55-59 years	Gastritis and duodenitis	2019	68.664211	111.51655	41.123124
Male	60-64 years	Gastritis and duodenitis	2019	72.20167	101.39515	51.479727
Female	60-64 years	Gastritis and duodenitis	2019	78.259934	120.5201	48.904811
Male	65-69 years	Gastritis and duodenitis	2019	85.185497	112.54744	61.943796
Female	65-69 years	Gastritis and duodenitis	2019	93.298421	136.29642	62.862651
Male	70-74 years	Gastritis and duodenitis	2019	94.551208	123.95779	69.066639
Female	70-74 years	Gastritis and duodenitis	2019	97.359732	135.94066	68.559882
Male	75-79 years	Gastritis and duodenitis	2019	107.30809	135.7348	80.276333
Female	75-79 years	Gastritis and duodenitis	2019	99.774192	134.70479	76.11748
Male	80-84 years	Gastritis and duodenitis	2019	117.86922	142.59709	86.036363
Female	80-84 years	Gastritis and duodenitis	2019	110.64813	139.14669	87.011553
Male	85-89 years	Gastritis and duodenitis	2019	135.80851	161.04689	105.42387
Female	85-89 years	Gastritis and duodenitis	2019	113.17245	136.40028	91.438404
Male	90-94 years	Gastritis and duodenitis	2019	97.837336	114.57638	79.529145
Female	90-94 years	Gastritis and duodenitis	2019	118.13159	139.74649	94.939673
Male	95+ years	Gastritis and duodenitis	2019	94.429983	113.86077	74.903708
Female	95+ years	Gastritis and duodenitis	2019	128.7681	152.7357	100.98512
Male	<5 years	Gastroesophageal reflux disease	2019	0	0	0
Female	<5 years	Gastroesophageal reflux disease	2019	0	0	0
Male	5-9 years	Gastroesophageal reflux disease	2019	0.1817973	0.3813353	0.0697633
Female	5-9 years	Gastroesophageal reflux disease	2019	0.1884152	0.4025644	0.0705532
Male	10-14 years	Gastroesophageal reflux disease	2019	4.2097754	7.9808135	1.8656958
Female	10-14 years	Gastroesophageal reflux disease	2019	4.5198056	8.4513103	1.9774742
Male	15-19 years	Gastroesophageal reflux disease	2019	25.446872	48.87607	12.370372
Female	15-19 years	Gastroesophageal reflux disease	2019	27.400677	51.842923	13.365726
Male	20-24 years	Gastroesophageal reflux disease	2019	64.304672	125.97534	31.364979
Female	20-24 years	Gastroesophageal reflux disease	2019	69.055204	134.31119	34.570322
Male	25-29 years	Gastroesophageal reflux disease	2019	90.154224	169.05403	42.185196
Female	25-29 years	Gastroesophageal reflux disease	2019	96.289941	179.26588	45.074139
Male	30-34 years	Gastroesophageal reflux disease	2019	104.08569	195.56547	48.818138
Female	30-34 years	Gastroesophageal reflux disease	2019	110.71064	205.74073	51.666285
Male	35-39 years	Gastroesophageal reflux disease	2019	111.69244	210.50893	51.408901
Female	35-39 years	Gastroesophageal reflux disease	2019	119.22878	223.33194	55.043781
Male	40-44 years	Gastroesophageal reflux disease	2019	116.33742	209.20528	55.059215
Female	40-44 years	Gastroesophageal reflux disease	2019	123.8903	222.49654	58.418927
Male	45-49 years	Gastroesophageal reflux disease	2019	117.78458	220.46385	57.609215
Female	45-49 years	Gastroesophageal reflux disease	2019	125.62903	230.53884	61.910615
Male	50-54 years	Gastroesophageal reflux disease	2019	119.57303	219.93761	58.690702
Female	50-54 years	Gastroesophageal reflux disease	2019	128.57943	235.54646	63.192703
Male	55-59 years	Gastroesophageal reflux disease	2019	124.70648	235.99815	58.09899
Female	55-59 years	Gastroesophageal reflux disease	2019	136.25295	257.14026	64.174242
Male	60-64 years	Gastroesophageal reflux disease	2019	129.374	245.36448	61.325683
Female	60-64 years	Gastroesophageal reflux disease	2019	142.34702	270.04744	67.977547
Male	65-69 years	Gastroesophageal reflux disease	2019	131.47712	242.61168	65.414819
Female	65-69 years	Gastroesophageal reflux disease	2019	143.30709	265.56012	71.888181
Male	70-74 years	Gastroesophageal reflux disease	2019	135.35541	242.92998	67.433794
Female	70-74 years	Gastroesophageal reflux disease	2019	144.53248	261.23718	71.620181

Male	75-79 years	Gastroesophageal reflux disease	2019	135.24026	243.22672	68.689099
Female	75-79 years	Gastroesophageal reflux disease	2019	141.39491	255.34037	71.612686
Male	80-84 years	Gastroesophageal reflux disease	2019	128.90615	231.39997	64.475892
Female	80-84 years	Gastroesophageal reflux disease	2019	133.08646	239.29486	67.16273
Male	85-89 years	Gastroesophageal reflux disease	2019	119.02504	218.17336	58.559323
Female	85-89 years	Gastroesophageal reflux disease	2019	117.39949	215.45028	58.124458
Male	90-94 years	Gastroesophageal reflux disease	2019	115.34052	207.80572	57.654205
Female	90-94 years	Gastroesophageal reflux disease	2019	107.2211	192.38891	54.002774
Male	95+ years	Gastroesophageal reflux disease	2019	114.50686	202.26908	55.727147
Female	95+ years	Gastroesophageal reflux disease	2019	102.77622	180.58928	50.178942
