# Supplementary material for: The global, regional, and national patterns of change in the burden of nonmalignant upper gastrointestinal diseases from 1990 to 2019 and the forecast for the next decade
Source: Int J Surg. 2024 Jul 3;111(1):80–92. doi: 10.1097/JS9.0000000000001902 (PMC11745775; doi:10.1097/JS9.0000000000001902)
Supplement: Supplementary file 4 [file js9-111-0080-s004.pdf]

Table S3.Age-standardized DALYs rates of PUD, GD, and GERD with inequality slope index analysis, catego

Region	Cause	Year	lm
All included	Gastroesophageal reflux disease	1990	-10.6874621
All included	Peptic ulcer disease	1990	-253.6992163
All included	Gastritis and duodenitis	1990	-60.19740551
All included	Gastroesophageal reflux disease	2019	-12.71301684
All included	Peptic ulcer disease	2019	-170.6956461
All included	Gastritis and duodenitis	2019	-44.97620873
Low SDI	Gastroesophageal reflux disease	1990	-8.804225666
Low SDI	Peptic ulcer disease	1990	23.31999095
Low SDI	Gastritis and duodenitis	1990	-13.74757345
Low SDI	Gastroesophageal reflux disease	2019	7.958598513
Low SDI	Peptic ulcer disease	2019	-125.7228211
Low SDI	Gastritis and duodenitis	2019	-38.72338817
Low-middle SDI	Gastroesophageal reflux disease	1990	8.013667496
Low-middle SDI	Peptic ulcer disease	1990	-134.9918451
Low-middle SDI	Gastritis and duodenitis	1990	-17.17604428
Low-middle SDI	Gastroesophageal reflux disease	2019	13.81908494
Low-middle SDI	Peptic ulcer disease	2019	-79.23291611
Low-middle SDI	Gastritis and duodenitis	2019	-18.56434466
Middle SDI	Peptic ulcer disease	1990	9.797754877
Middle SDI	Gastritis and duodenitis	1990	-22.54407941
Middle SDI	Gastroesophageal reflux disease	1990	13.5302064
Middle SDI	Peptic ulcer disease	2019	-83.73125618
Middle SDI	Gastritis and duodenitis	2019	-14.66946656
Middle SDI	Gastroesophageal reflux disease	2019	-7.299711843
High-middle SDI	Peptic ulcer disease	1990	-32.57661339
High-middle SDI	Gastritis and duodenitis	1990	-4.43589045
High-middle SDI	Gastroesophageal reflux disease	1990	-8.5217599
High-middle SDI	Peptic ulcer disease	2019	-19.28415399
High-middle SDI	Gastritis and duodenitis	2019	2.514601077
High-middle SDI	Gastroesophageal reflux disease	2019	9.902543305
High SDI	Peptic ulcer disease	1990	-65.09052366
High SDI	Gastritis and duodenitis	1990	-3.155541197
High SDI	Gastroesophageal reflux disease	1990	-26.36638896
High SDI	Peptic ulcer disease	2019	-19.1820915
High SDI	Gastritis and duodenitis	2019	-4.360727846
High SDI	Gastroesophageal reflux disease	2019	-28.82770917
Eastern Sub-Saharan Africa	Gastroesophageal reflux disease	1990	0.470917191
Eastern Sub-Saharan Africa	Peptic ulcer disease	1990	-66.74140895
Eastern Sub-Saharan Africa	Gastritis and duodenitis	1990	-18.12151403
Eastern Sub-Saharan Africa	Gastroesophageal reflux disease	2019	0.783609353
Eastern Sub-Saharan Africa	Peptic ulcer disease	2019	-74.69532177
Eastern Sub-Saharan Africa	Gastritis and duodenitis	2019	-25.1839564
Western Sub-Saharan Africa	Peptic ulcer disease	1990	-100.6869407
Western Sub-Saharan Africa	Gastritis and duodenitis	1990	-21.42678694
Western Sub-Saharan Africa	Gastroesophageal reflux disease	1990	0.669990461
Western Sub-Saharan Africa	Peptic ulcer disease	2019	-181.5569034
Western Sub-Saharan Africa	Gastritis and duodenitis	2019	-26.77910837
Western Sub-Saharan Africa	Gastroesophageal reflux disease	2019	1.164967234
Central Sub-Saharan Africa	Peptic ulcer disease	1990	-178.0461593
Central Sub-Saharan Africa	Gastritis and duodenitis	1990	-55.36671427
Central Sub-Saharan Africa	Gastroesophageal reflux disease	1990	0.299895675
Central Sub-Saharan Africa	Peptic ulcer disease	2019	-240.3642517
Central Sub-Saharan Africa	Gastritis and duodenitis	2019	-81.89790702
Central Sub-Saharan Africa	Gastroesophageal reflux disease	2019	0.233952977
North Africa and Middle East	Peptic ulcer disease	1990	-118.3409673
North Africa and Middle East	Gastritis and duodenitis	1990	-7.377409681

North Africa and Middle East	Gastroesophageal reflux disease	1990	-0.775075896
North Africa and Middle East	Peptic ulcer disease	2019	-100.0158768
North Africa and Middle East	Gastritis and duodenitis	2019	-9.313207329
North Africa and Middle East	Gastroesophageal reflux disease	2019	-0.448781491
Oceania	Peptic ulcer disease	1990	-318.1197812
Oceania	Gastritis and duodenitis	1990	-9.462677068
Oceania	Gastroesophageal reflux disease	1990	0.170198656
Oceania	Peptic ulcer disease	2019	-284.1105684
Oceania	Gastritis and duodenitis	2019	-14.79892372
Oceania	Gastroesophageal reflux disease	2019	0.272034507
South Asia	Gastroesophageal reflux disease	1990	2.385452112
South Asia	Peptic ulcer disease	1990	408.6239686
South Asia	Gastritis and duodenitis	1990	16.79167612
South Asia	Gastroesophageal reflux disease	2019	2.309229789
South Asia	Peptic ulcer disease	2019	161.369348
South Asia	Gastritis and duodenitis	2019	15.97340062
Caribbean	Peptic ulcer disease	1990	-248.6095388
Caribbean	Gastritis and duodenitis	1990	-31.7655808
Caribbean	Gastroesophageal reflux disease	1990	0.695801406
Caribbean	Peptic ulcer disease	2019	-174.42915
Caribbean	Gastritis and duodenitis	2019	-37.90554631
Caribbean	Gastroesophageal reflux disease	2019	0.755936579
Southeast Asia	Peptic ulcer disease	1990	-529.4416857
Southeast Asia	Gastritis and duodenitis	1990	-22.56305316
Southeast Asia	Gastroesophageal reflux disease	1990	0.244555677
Southeast Asia	Peptic ulcer disease	2019	-219.8577427
Southeast Asia	Gastritis and duodenitis	2019	-12.15164255
Southeast Asia	Gastroesophageal reflux disease	2019	-0.034714356
Southern Sub-Saharan Africa	Peptic ulcer disease	1990	-141.7263102
Southern Sub-Saharan Africa	Gastritis and duodenitis	1990	-3.243226682
Southern Sub-Saharan Africa	Gastroesophageal reflux disease	1990	4.605979561
Southern Sub-Saharan Africa	Peptic ulcer disease	2019	-282.559691
Southern Sub-Saharan Africa	Gastritis and duodenitis	2019	-24.77771274
Southern Sub-Saharan Africa	Gastroesophageal reflux disease	2019	6.316254969
Central Latin America	Peptic ulcer disease	1990	-258.2094603
Central Latin America	Gastritis and duodenitis	1990	-24.35231308
Central Latin America	Gastroesophageal reflux disease	1990	0.298170876
Central Latin America	Peptic ulcer disease	2019	-114.6900265
Central Latin America	Gastritis and duodenitis	2019	-25.54784773
Central Latin America	Gastroesophageal reflux disease	2019	0.088883179
Central Asia	Peptic ulcer disease	1990	-148.8162755
Central Asia	Gastritis and duodenitis	1990	-10.88721388
Central Asia	Gastroesophageal reflux disease	1990	0.2067617
Central Asia	Peptic ulcer disease	2019	-45.04894998
Central Asia	Gastritis and duodenitis	2019	-5.790933417
Central Asia	Gastroesophageal reflux disease	2019	-0.112693153
East Asia	Peptic ulcer disease	1990	-32.84769662
East Asia	Gastritis and duodenitis	1990	-21.99866827
East Asia	Gastroesophageal reflux disease	1990	2.12925775
East Asia	Peptic ulcer disease	2019	-108.26882
East Asia	Gastritis and duodenitis	2019	-25.54950147
East Asia	Gastroesophageal reflux disease	2019	3.838940304
Andean Latin America	Peptic ulcer disease	1990	-154.7094433
Andean Latin America	Gastritis and duodenitis	1990	-28.46095851
Andean Latin America	Gastroesophageal reflux disease	1990	0.607592679
Andean Latin America	Peptic ulcer disease	2019	-109.8368476
Andean Latin America	Gastritis and duodenitis	2019	9.408880911
Andean Latin America	Gastroesophageal reflux disease	2019	0.587327121

Central Europe	Peptic ulcer disease	1990	11.47329813
Central Europe	Gastritis and duodenitis	1990	-2.838382249
Central Europe	Gastroesophageal reflux disease	1990	7.808523613
Central Europe	Peptic ulcer disease	2019	14.72726774
Central Europe	Gastritis and duodenitis	2019	1.544967428
Central Europe	Gastroesophageal reflux disease	2019	12.252536
Eastern Europe	Gastroesophageal reflux disease	1990	-1.400350407
Eastern Europe	Peptic ulcer disease	1990	-18.77268433
Eastern Europe	Gastritis and duodenitis	1990	4.751599187
Eastern Europe	Gastroesophageal reflux disease	2019	0.717671942
Eastern Europe	Peptic ulcer disease	2019	-12.34664237
Eastern Europe	Gastritis and duodenitis	2019	-0.510321594
Southern Latin America	Gastroesophageal reflux disease	1990	-0.266224875
Southern Latin America	Peptic ulcer disease	1990	18.50273853
Southern Latin America	Gastritis and duodenitis	1990	7.114210105
Southern Latin America	Gastroesophageal reflux disease	2019	-0.190456581
Southern Latin America	Peptic ulcer disease	2019	-6.154788202
Southern Latin America	Gastritis and duodenitis	2019	0.278388693
Western Europe	Gastroesophageal reflux disease	1990	-11.62311856
Western Europe	Peptic ulcer disease	1990	-20.80564459
Western Europe	Gastritis and duodenitis	1990	-1.307138794
Western Europe	Gastroesophageal reflux disease	2019	-15.67259726
Western Europe	Peptic ulcer disease	2019	4.111462716
Western Europe	Gastritis and duodenitis	2019	0.695633478
High-income North America	Peptic ulcer disease	1990	-452.5540086
High-income North America	Gastritis and duodenitis	1990	-14.80824804
High-income North America	Gastroesophageal reflux disease	1990	-7.937429715
High-income North America	Peptic ulcer disease	2019	-176.4563548
High-income North America	Gastritis and duodenitis	2019	-0.761883357
High-income North America	Gastroesophageal reflux disease	2019	-7.931481529
High-income Asia Pacific	Peptic ulcer disease	1990	-235.1399336
High-income Asia Pacific	Gastritis and duodenitis	1990	-25.54069854
High-income Asia Pacific	Gastroesophageal reflux disease	1990	-8.23714549
High-income Asia Pacific	Peptic ulcer disease	2019	-17.36244527
High-income Asia Pacific	Gastritis and duodenitis	2019	-0.218935055
High-income Asia Pacific	Gastroesophageal reflux disease	2019	-1.816654381
Sub-Saharan Africa	Gastroesophageal reflux disease	1990	0.92751423
Sub-Saharan Africa	Peptic ulcer disease	1990	-96.85952218
Sub-Saharan Africa	Gastritis and duodenitis	1990	-11.70758328
Sub-Saharan Africa	Gastroesophageal reflux disease	2019	0.360251009
Sub-Saharan Africa	Peptic ulcer disease	2019	-114.9212828
Sub-Saharan Africa	Gastritis and duodenitis	2019	-15.82658607
North Africa and Middle East	Peptic ulcer disease	1990	-118.3409673
North Africa and Middle East	Gastritis and duodenitis	1990	-7.377409681
North Africa and Middle East	Gastroesophageal reflux disease	1990	-0.775075896
North Africa and Middle East	Peptic ulcer disease	2019	-100.0158768
North Africa and Middle East	Gastritis and duodenitis	2019	-9.313207329
North Africa and Middle East	Gastroesophageal reflux disease	2019	-0.448781491
Southeast Asia, east Asia, and Oceania	Peptic ulcer disease	1990	-321.5386322
Southeast Asia, east Asia, and Oceania	Gastritis and duodenitis	1990	-15.01823673
Southeast Asia, east Asia, and Oceania	Gastroesophageal reflux disease	1990	0.450683741
Southeast Asia, east Asia, and Oceania	Peptic ulcer disease	2019	-177.3155493
Southeast Asia, east Asia, and Oceania	Gastritis and duodenitis	2019	-6.875019005
Southeast Asia, east Asia, and Oceania	Gastroesophageal reflux disease	2019	-0.145228088
South Asia	Gastroesophageal reflux disease	1990	2.385452112
South Asia	Peptic ulcer disease	1990	408.6239686
South Asia	Gastritis and duodenitis	1990	16.79167612
South Asia	Gastroesophageal reflux disease	2019	2.309229789

South Asia	Peptic ulcer disease	2019	161.369348
South Asia	Gastritis and duodenitis	2019	15.97340062
Latin America and Caribbean	Peptic ulcer disease	1990	-180.5184924
Latin America and Caribbean	Gastritis and duodenitis	1990	-19.41004096
Latin America and Caribbean	Gastroesophageal reflux disease	1990	-3.572814067
Latin America and Caribbean	Peptic ulcer disease	2019	-83.20996154
Latin America and Caribbean	Gastritis and duodenitis	2019	-13.56166501
Latin America and Caribbean	Gastroesophageal reflux disease	2019	-3.544219623
Central Europe, eastern Europe, and central Asia	Peptic ulcer disease	1990	-56.97653099
Central Europe, eastern Europe, and central Asia	Gastritis and duodenitis	1990	-0.42530892
Central Europe, eastern Europe, and central Asia	Gastroesophageal reflux disease	1990	1.904571072
Central Europe, eastern Europe, and central Asia	Peptic ulcer disease	2019	-28.88361709
Central Europe, eastern Europe, and central Asia	Gastritis and duodenitis	2019	3.71929641
Central Europe, eastern Europe, and central Asia	Gastroesophageal reflux disease	2019	4.395537064
High income	Gastroesophageal reflux disease	1990	-10.28608233
High income	Peptic ulcer disease	1990	-84.90633851
High income	Gastritis and duodenitis	1990	-4.014551757
High income	Gastroesophageal reflux disease	2019	-15.31853739
High income	Peptic ulcer disease	2019	-20.73559266
High income	Gastritis and duodenitis	2019	-1.170935955

orized by all included, SDI, and GBD regions.

lm_lower	lm_upper	ncvTest_p	rlm	rlm_lower	rlm_upper
-21.75596509	0.38104089	0.035195027	-15.60649787	-27.88336527	-3.329630466
-313.8088018	-193.5896308	1.23E-13	-202.2236747	-232.8847322	-171.5626172
-70.75557031	-49.63924072	6.12E-16	-51.1039636	-59.31457143	-42.89335578
-23.54016426	-1.885869422	0.435201482	-16.48928423	-28.81955673	-4.159011735
-197.526057	-143.8652352	4.70E-15	-149.9988901	-170.1952294	-129.8025507
-53.19065544	-36.76176201	1.86E-18	-32.8516455	-39.0041	-26.69919101
-23.78378439	6.175333062	1.53E-08	-0.609724586	-1.390471896	0.171022724
-100.5268431	147.166825	0.125790794	-1.444443624	-126.0741498	123.1852625
-55.76025755	28.26511065	0.611306436	-10.43158414	-51.4129416	30.54977333
-11.89399003	27.81118705	0.001184698	0.597125585	-0.25335163	1.4476028
-217.2759376	-34.16970457	0.566897627	-115.4363307	-210.2468537	-20.62580757
-84.51285314	7.066076802	0.189658849	-32.7495775	-76.33533409	10.8361791
-13.09466874	29.12200373	0.163175849	12.11243472	-13.98742816	38.21229761
-314.5324633	44.54877312	0.060350342	-83.96608491	-232.208291	64.27612115
-38.26263248	3.910543916	0.619144377	-17.81370263	-37.85824948	2.230844221
-9.406406902	37.04457679	0.53175254	16.99826128	-9.550210479	43.54673304
-168.5505516	10.08471934	0.115193246	-54.73512922	-135.3769557	25.90669723
-37.94144413	0.812754811	0.450444457	-18.00920111	-36.22806519	0.209662978
-88.57968112	108.1751909	0.497055812	-6.334705946	-85.60035761	72.93094572
-48.44044349	3.352284669	3.49E-05	-11.56692562	-36.28282639	13.14897514
-14.73815638	41.79856918	0.183700132	13.5302064	-14.73815638	41.79856918
-146.9229542	-20.53955818	0.02697606	-71.88011463	-131.1057072	-12.65452201
-37.21100974	7.872076617	0.321303247	-13.6596469	-36.76266311	9.443369319
-46.6588981	32.05947442	0.761272975	-7.299711843	-46.6588981	32.05947442
-124.0765446	58.92331781	0.163650405	-43.38764996	-103.4899456	16.71464564
-22.76218553	13.89040463	0.187675454	-0.812754942	-11.98881752	10.36330764
-39.17996096	22.13644116	0.442613205	-14.01702508	-51.83533245	23.80128229
-65.99286649	27.4245585	0.961779017	-21.17522798	-61.75666167	19.40620572
-11.09783876	16.12704091	0.325464629	1.385671803	-8.372935688	11.14427929
-23.39705112	43.20213773	0.311089498	10.78506513	-29.1983356	50.76846587
-94.62853974	-35.55250758	0.01262862	-57.3086443	-81.63247389	-32.98481471
-9.595433855	3.284351461	0.028930685	-1.540881076	-6.575106059	3.493343907
-42.76601116	-9.966766757	0.02881266	-24.52962228	-39.77792222	-9.281322343
-35.05884886	-3.305334143	0.012884444	-14.26206922	-27.96867435	-0.555464097
-8.794360346	0.072904653	0.796932423	-4.549484824	-9.34237541	0.243405762
-43.79346564	-13.8619527	0.034930149	-28.91872203	-42.43439474	-15.40304933
-1.784938164	2.726772545	0.866908648	0.322594993	-0.199054752	0.844244738
-218.8944212	85.41160333	0.623115903	-70.25420812	-255.9574708	115.4490546
-60.07016925	23.8271412	0.532499156	-11.9897485	-54.93875843	30.95926143
-1.313371017	2.880589722	0.834115854	0.540540393	0.134184768	0.946896017
-138.0669942	-11.32364938	0.482285347	-76.63879146	-148.7699624	-4.507620489
-49.14715747	-1.220755323	0.602713427	-23.40892634	-48.60404151	1.786188842
-217.8850132	16.51113181	0.7890971	-73.92215141	-152.0007116	4.156408757
-40.89506632	-1.958507559	0.943911298	-21.38953939	-43.79815193	1.019073159
-0.593818692	1.933799615	0.017102788	0.17153024	-0.30852147	0.651581951
-263.8447955	-99.26901135	0.648529093	-191.3500586	-258.7502887	-123.9498285
-39.48223565	-14.07598109	0.810206087	-26.92207628	-40.54900761	-13.29514495
-0.169247127	2.499181595	0.00048754	0.351373174	-0.050949363	0.753695711
-307.1773778	-48.91494093	0.51634391	-178.0461593	-307.1773778	-48.91494093
-97.63188453	-13.10154402	0.688909831	-55.36671427	-97.63188453	-13.10154402
-0.10087114	0.700662491	0.803254536	0.391655523	0.276787517	0.506523529
-370.2407614	-110.487742	0.096141761	-280.6630027	-335.6120693	-225.7139361
-114.3861096	-49.40970442	0.275194315	-84.36076305	-119.9742641	-48.74726201
-0.002844982	0.470750935	0.478780564	0.20548516	0.132720287	0.278250033
-226.4544558	-10.22747874	0.004138392	-81.79840249	-158.7525207	-4.844284318
-10.85948543	-3.895333937	0.742501399	-7.371708304	-10.54806076	-4.195355852

-4.246444787	2.696292995	0.047788796	-1.548901601	-3.228258705	0.130455503
-159.2213872	-40.81036639	9.79E-05	-59.99154057	-86.69985191	-33.28322924
-13.06268758	-5.563727075	0.001732438	-8.034978375	-11.83961476	-4.230341995
-6.09187558	5.194312598	0.019469705	-1.77893934	-3.721290622	0.163411942
-699.9895636	63.75000111	0.749853188	-307.5936867	-611.5037222	-3.683651245
-20.99226742	2.066913284	0.689644217	-9.284426305	-21.14571923	2.576866624
-0.098248905	0.438646218	0.755179344	0.169227602	-0.105799859	0.444255063
-668.8990096	100.6778728	0.371129817	-268.6600775	-558.3866556	21.06650066
-26.58409337	-3.013754065	0.601161004	-15.33470488	-26.03775298	-4.631656783
-0.154902655	0.698971668	0.391440192	0.313470454	-0.131010498	0.757951406
-3.183448109	7.954352333	0.643001202	3.101184008	0.328489221	5.873878795
-431.8854751	1249.133412	0.342330964	408.6239686	-431.8854751	1249.133412
-25.56581501	59.14916725	0.56902455	16.79167612	-25.56581501	59.14916725
-2.121284685	6.739744264	0.509931822	2.637170355	-2.072659786	7.347000496
-66.08363575	388.8223318	0.596501983	161.369348	-66.08363575	388.8223318
9.652535302	22.29426594	0.529267411	16.93263697	11.36594974	22.4993242
-395.1792027	-102.0398749	0.000542677	-145.3618698	-237.9987499	-52.72498964
-69.27737532	5.746213723	0.992520498	-35.87456888	-69.56228519	-2.186852574
0.134985257	1.256617555	0.104351232	0.653298511	0.106868092	1.199728931
-240.3213694	-108.5369306	0.010215095	-169.3425612	-235.3747993	-103.3103231
-65.24328951	-10.56780311	0.787098516	-43.21069518	-63.43890445	-22.98248591
0.165932489	1.34594067	0.459156586	0.710808427	0.068142482	1.353474372
-968.9753465	-89.90802495	0.180588187	-534.7316445	-1009.785764	-59.67752528
-42.85762208	-2.268484239	0.267377806	-22.56305316	-42.85762208	-2.268484239
-0.709329518	1.198440873	0.449941563	0.089043663	-0.217662121	0.395749448
-393.562241	-46.15324441	0.105290769	-230.4261969	-425.9927279	-34.85966594
-21.58285783	-2.720427282	0.148937583	-12.6852211	-23.24913765	-2.12130455
-1.058877745	0.989449032	0.950142472	-0.052486829	-0.379136261	0.274162603
-331.3203956	47.86777533	0.778040511	-141.7263102	-331.3203956	47.86777533
-63.38995371	56.90350034	0.637771827	-3.400826456	-80.58456354	73.78291063
1.708366529	7.503592592	0.674153895	4.605979561	1.708366529	7.503592592
-592.4050041	27.28562217	0.502595879	-235.8466947	-417.2107005	-54.48268885
-87.98249289	38.42706741	0.513995268	-15.99116535	-48.93098574	16.94865504
3.119372832	9.513137106	0.37799947	7.511046163	5.580068598	9.442023728
-418.5430768	-97.87584373	0.058125897	-285.2624038	-328.4224822	-242.1023255
-54.1814918	5.476865638	0.292018036	-20.84255211	-61.4723798	19.78727558
-0.020957937	0.617299688	0.273921777	0.254061479	-0.063923626	0.572046584
-228.7813475	-0.59870545	0.113939548	-114.6900265	-228.7813475	-0.59870545
-52.35390021	1.25820475	0.133022908	-21.51671325	-46.38115218	3.347725674
-0.235527491	0.413293849	0.993273523	0.083420681	-0.308474652	0.475316013
-280.1895912	-17.44295973	0.044762517	-75.75713502	-141.4813509	-10.03291915
-22.04954517	0.275117408	0.053721662	-4.983281413	-10.36214931	0.395586485
-0.09270355	0.506226951	0.419382386	0.209311887	-0.134685821	0.553309596
-148.2249828	58.1270828	0.038153035	-15.41563308	-78.54972461	47.71845844
-15.73468754	4.152820703	0.078168601	-2.494310944	-7.993315497	3.004693609
-0.34249499	0.117108684	0.644724776	-0.112693153	-0.34249499	0.117108684
-108.1475956	42.45220238	0.999674184	-32.84748143	-196.1401794	130.4452166
-125.6143172	81.61698061	0.999674184	-21.99896438	-246.6961962	202.6982675
-3.51908179	7.77759729	0.999674184	2.129273892	-10.11951548	14.37806327
-214.4674592	-2.07018075	0.998919747	-108.2680471	-340.5705496	124.0344555
-39.99053531	-11.10846763	0.998919747	-25.54960657	-57.1384134	6.039200269
-3.513513227	11.19139383	0.998919747	3.838993813	-12.24401222	19.92199984
-516.8842065	207.46532	0.928232879	-154.7094433	-516.8842065	207.46532
-47.33282647	-9.589090545	0.928232879	-28.46095851	-47.33282647	-9.589090545
-0.034842085	1.250027443	0.928232879	0.607592679	-0.034842085	1.250027443
-220.4148085	0.741113356	0.697615635	-109.8368476	-220.4148085	0.741113356
-63.00225694	81.82001876	0.697615635	9.408880911	-63.00225694	81.82001876
0.304937136	0.869717106	0.697615635	0.587327121	0.304937136	0.869717106

-30.98996763	53.93656389	0.320880025	12.90304849	-27.19043063	52.9965276
-15.61000995	9.933245448	0.952324532	-3.356620956	-9.112485306	2.399243394
-8.986158377	24.6032056	0.437240089	0.039006719	-0.294525885	0.372539323
-17.31111806	46.76565353	0.722463505	18.71229086	-19.10857385	56.53315556
-9.043863993	12.13379885	0.418073583	0.373837733	-7.426154752	8.173830217
-5.909182392	30.41425439	0.65905561	-0.051403616	-0.41408386	0.311276629
-9.286793111	6.486092297	0.812407707	-1.688530399	-7.653361886	4.276301089
-134.9221326	97.37676394	0.138820028	27.25416631	-40.06655999	94.5748926
-3.839956671	13.34315505	0.639071067	5.221458168	-4.09285524	14.53577158
-3.419251682	4.854595566	0.360749016	-0.06006512	-1.09168019	0.971549949
-63.08307368	38.38978894	0.982262163	-12.34664237	-63.08307368	38.38978894
-4.827067863	3.806424675	0.946759378	-0.510321594	-4.827067863	3.806424675
-0.412513028	-0.119936722	0.855318483	-0.266224875	-0.412513028	-0.119936722
0.465723374	36.53975368	0.855318483	18.50273853	0.465723374	36.53975368
-1.532384378	15.76080459	0.855318483	7.114210105	-1.532384378	15.76080459
-0.250804816	-0.130108345	0.84472292	-0.190456581	-0.250804816	-0.130108345
-33.57890221	21.26932581	0.84472292	-6.154788202	-33.57890221	21.26932581
-1.085930125	1.64270751	0.84472292	0.278388693	-1.085930125	1.64270751
-27.76914297	4.522905856	0.349612693	-11.06926358	-30.33993094	8.201403788
-50.54859593	8.937306752	0.594976049	-21.53142967	-59.95282201	16.88996266
-6.943458876	4.329181287	0.394304581	-1.356547137	-5.857288819	3.144194546
-31.90628469	0.56109017	0.534942382	-15.24637519	-35.672006	5.179255622
-10.04580479	18.26873022	0.799510848	3.93396508	-9.050449349	16.91837951
-4.961863447	6.353130403	0.389737817	1.348837601	-2.401332375	5.099007577
-1006.56389	101.4558726	0.939249963	-452.5540086	-1006.56389	101.4558726
-15.2018973	-14.41459877	0.939249963	-14.80824804	-15.2018973	-14.41459877
-67.31143578	51.43657635	0.939249963	-7.937429715	-67.31143578	51.43657635
-386.3150013	33.40229161	0.937155771	-176.4563548	-386.3150013	33.40229161
-3.056776765	1.533010051	0.937155771	-0.761883357	-3.056776765	1.533010051
-42.45764671	26.59468366	0.937155771	-7.931481529	-42.45764671	26.59468366
-400.6489961	-69.63087105	0.746567421	-235.1399336	-400.6489961	-69.63087105
-108.0213625	56.93996548	0.467777934	-25.54069854	-108.0213625	56.93996548
-28.08872819	11.61443721	0.920185128	-8.23714549	-28.08872819	11.61443721
-65.631781	30.90689047	0.244746455	-17.36244527	-65.631781	30.90689047
-4.460982404	4.023112293	0.495136768	-0.218935055	-4.460982404	4.023112293
-17.63219497	13.99888621	0.765961309	-1.816654381	-17.63219497	13.99888621
-0.012653693	1.867682152	0.042456482	0.400529174	0.001321258	0.79973709
-173.2893088	-20.42973554	0.102526611	-88.9430304	-158.2537911	-19.63226968
-46.32606234	22.91089578	0.264761769	-8.296714119	-42.55249229	25.95906405
-0.584472859	1.304974878	0.03300561	0.009275587	-0.365788264	0.384339437
-172.184539	-57.65802654	0.225123199	-107.723571	-156.7844884	-58.66265365
-44.22337646	12.57020432	0.077005405	-11.20957039	-38.2072728	15.78813203
-226.4544558	-10.22747874	0.004138392	-81.79840249	-158.7525207	-4.844284318
-10.85948543	-3.895333937	0.742501399	-7.371708304	-10.54806076	-4.195355852
-4.246444787	2.696292995	0.047788796	-1.548901601	-3.228258705	0.130455503
-159.2213872	-40.81036639	9.79E-05	-59.99154057	-86.69985191	-33.28322924
-13.06268758	-5.563727075	0.001732438	-8.034978375	-11.83961476	-4.230341995
-6.09187558	5.194312598	0.019469705	-1.77893934	-3.721290622	0.163411942
-501.0445307	-142.0327336	0.003956491	-284.6587176	-459.2703901	-110.0470451
-25.88683689	-4.149636565	0.052124065	-13.10174634	-22.54387973	-3.65961295
-0.576000659	1.477368142	0.208598314	0.057583832	-0.089994584	0.205162247
-256.7595812	-97.87151741	0.008476907	-162.9747038	-223.674002	-102.2754055
-12.48444203	-1.265595982	0.118602618	-5.385660979	-9.782624097	-0.988697862
-1.217368254	0.926912078	0.66006599	-0.05570512	-0.244069882	0.132659641
-3.183448109	7.954352333	0.643001202	3.101184008	0.328489221	5.873878795
-431.8854751	1249.133412	0.342330964	408.6239686	-431.8854751	1249.133412
-25.56581501	59.14916725	0.56902455	16.79167612	-25.56581501	59.14916725
-2.121284685	6.739744264	0.509931822	2.637170355	-2.072659786	7.347000496

-66.08363575	388.8223318	0.596501983	161.369348	-66.08363575	388.8223318
9.652535302	22.29426594	0.529267411	16.93263697	11.36594974	22.4993242
-255.831546	-105.2054387	1.18E-05	-140.3574582	-204.9732969	-75.74161947
-35.84885287	-2.971229058	0.881454662	-22.10083148	-35.57494396	-8.626718993
-7.686691962	0.541063829	9.66E-05	0.288321171	-0.053117178	0.62975952
-119.8715479	-46.54837514	3.12E-05	-57.36936091	-85.12829199	-29.61042982
-27.53785148	0.414521463	0.305843724	-13.57037219	-27.64289221	0.50214783
-7.660762086	0.572322841	0.00020159	-0.000496731	-0.311482834	0.310489373
-116.9377966	2.98473464	0.000975321	-35.24855409	-70.67198668	0.174878513
-9.363384239	8.512766399	0.330793737	0.975595617	-4.069643807	6.020835041
-9.815393792	13.62453594	0.527666799	-0.054566776	-8.69101192	8.581878368
-69.45297187	11.68573769	0.033248888	-19.07498761	-51.47405454	13.32407933
-2.875791932	10.31438475	0.979392778	3.959259486	-0.918236569	8.83675554
-5.808569383	14.59964351	0.912967469	2.243491089	-5.34448197	9.831464148
-21.87005605	1.297891394	0.537631286	-10.37343933	-22.44936539	1.702486739
-160.3661636	-9.446513454	3.44E-05	-38.3695756	-62.82654487	-13.91260633
-11.32830557	3.299202052	0.011152031	-1.909440279	-7.488331118	3.669450561
-25.28455473	-5.352520047	0.951254005	-15.58785374	-26.78579839	-4.389909088
-50.13073964	8.659554326	8.66E-07	-2.285982372	-11.85438945	7.282424706
-5.823342315	3.481470405	0.838972318	-0.406616353	-3.946409849	3.133177143
