# Supplementary material for: The global, regional, and national patterns of change in the burden of nonmalignant upper gastrointestinal diseases from 1990 to 2019 and the forecast for the next decade
Source: Int J Surg. 2024 Jul 3;111(1):80–92. doi: 10.1097/JS9.0000000000001902 (PMC11745775; doi:10.1097/JS9.0000000000001902)
Supplement: Supplementary file 5 [file js9-111-0080-s005.pdf]

Table S4. Age-standardized DALYs rates of PUD, GD, and GERD with concentration index analysis, categorized b

Region	Cause	Year	Concentration_index
All included	Gastritis and duodenitis	1990	-0.14789958
All included	Gastritis and duodenitis	2019	-0.16283376
All included	Gastroesophageal reflux disease	1990	-0.05009993
All included	Gastroesophageal reflux disease	2019	-0.10102571
All included	Peptic ulcer disease	1990	-0.28600799
All included	Peptic ulcer disease	2019	-0.28791318
Low SDI	Gastritis and duodenitis	1990	-0.06310932
Low SDI	Gastritis and duodenitis	2019	-0.13712083
Low SDI	Gastroesophageal reflux disease	1990	0.00525659
Low SDI	Gastroesophageal reflux disease	2019	0.03664635
Low SDI	Peptic ulcer disease	1990	-0.06216427
Low SDI	Peptic ulcer disease	2019	-0.13107779
Low-middle SDI	Gastritis and duodenitis	1990	-0.02584809
Low-middle SDI	Gastritis and duodenitis	2019	-0.03871955
Low-middle SDI	Gastroesophageal reflux disease	1990	0.02126572
Low-middle SDI	Gastroesophageal reflux disease	2019	0.03909672
Low-middle SDI	Peptic ulcer disease	1990	-0.04431759
Low-middle SDI	Peptic ulcer disease	2019	0.0256964
Middle SDI	Gastritis and duodenitis	1990	-0.07204982
Middle SDI	Gastritis and duodenitis	2019	0.10974108
Middle SDI	Gastroesophageal reflux disease	1990	0.14723669
Middle SDI	Gastroesophageal reflux disease	2019	-0.20987812
Middle SDI	Peptic ulcer disease	1990	0.03566561
Middle SDI	Peptic ulcer disease	2019	-0.13874694
High-middle SDI	Gastritis and duodenitis	1990	-0.00766463
High-middle SDI	Gastritis and duodenitis	2019	0.03616601
High-middle SDI	Gastroesophageal reflux disease	1990	-0.01939156
High-middle SDI	Gastroesophageal reflux disease	2019	0.00683069
High-middle SDI	Peptic ulcer disease	1990	0.01888584
High-middle SDI	Peptic ulcer disease	2019	0.12730977
High SDI	Gastritis and duodenitis	1990	-0.1038738
High SDI	Gastritis and duodenitis	2019	-0.06630964
High SDI	Gastroesophageal reflux disease	1990	-0.04122466
High SDI	Gastroesophageal reflux disease	2019	-0.06416298
High SDI	Peptic ulcer disease	1990	-0.13210514
High SDI	Peptic ulcer disease	2019	-0.01116945
Eastern Sub-Saharan Africa	Gastritis and duodenitis	1990	-0.07124326
Eastern Sub-Saharan Africa	Gastritis and duodenitis	2019	-0.02947483
Eastern Sub-Saharan Africa	Gastroesophageal reflux disease	1990	-0.00081555
Eastern Sub-Saharan Africa	Gastroesophageal reflux disease	2019	0.00010502
Eastern Sub-Saharan Africa	Peptic ulcer disease	1990	-0.11242763
Eastern Sub-Saharan Africa	Peptic ulcer disease	2019	-0.05764305
Western Sub-Saharan Africa	Gastritis and duodenitis	1990	-0.04274093
Western Sub-Saharan Africa	Gastritis and duodenitis	2019	-0.04158907
Western Sub-Saharan Africa	Gastroesophageal reflux disease	1990	0.00403883
Western Sub-Saharan Africa	Gastroesophageal reflux disease	2019	0.00697565
Western Sub-Saharan Africa	Peptic ulcer disease	1990	-0.04175233
Western Sub-Saharan Africa	Peptic ulcer disease	2019	-0.08304774
Central Sub-Saharan Africa	Gastritis and duodenitis	1990	-0.03701131
Central Sub-Saharan Africa	Gastritis and duodenitis	2019	-0.04567099
Central Sub-Saharan Africa	Gastroesophageal reflux disease	1990	-0.00035139
Central Sub-Saharan Africa	Gastroesophageal reflux disease	2019	0.00067486
Central Sub-Saharan Africa	Peptic ulcer disease	1990	-0.0926479
Central Sub-Saharan Africa	Peptic ulcer disease	2019	-0.06354502
North Africa and Middle East	Gastritis and duodenitis	1990	-0.05685651
North Africa and Middle East	Gastritis and duodenitis	2019	-0.09273786

North Africa and Middle East	Gastroesophageal reflux disease	1990	0.01015923
North Africa and Middle East	Gastroesophageal reflux disease	2019	0.01285145
North Africa and Middle East	Peptic ulcer disease	1990	-0.19852828
North Africa and Middle East	Peptic ulcer disease	2019	-0.36077394
Oceania	Gastritis and duodenitis	1990	-0.01702248
Oceania	Gastritis and duodenitis	2019	-0.03002388
Oceania	Gastroesophageal reflux disease	1990	0.00087524
Oceania	Gastroesophageal reflux disease	2019	0.00072316
Oceania	Peptic ulcer disease	1990	-0.02516723
Oceania	Peptic ulcer disease	2019	-3.09E-05
South Asia	Gastritis and duodenitis	1990	0.02130459
South Asia	Gastritis and duodenitis	2019	0.03620556
South Asia	Gastroesophageal reflux disease	1990	0.00183213
South Asia	Gastroesophageal reflux disease	2019	0.00140896
South Asia	Peptic ulcer disease	1990	0.08582344
South Asia	Peptic ulcer disease	2019	0.11079084
Caribbean	Gastritis and duodenitis	1990	-0.15714145
Caribbean	Gastritis and duodenitis	2019	-0.1465066
Caribbean	Gastroesophageal reflux disease	1990	0.00058916
Caribbean	Gastroesophageal reflux disease	2019	0.00083415
Caribbean	Peptic ulcer disease	1990	-0.36459152
Caribbean	Peptic ulcer disease	2019	-0.35022724
Southeast Asia	Gastritis and duodenitis	1990	-0.03719454
Southeast Asia	Gastritis and duodenitis	2019	-0.05261744
Southeast Asia	Gastroesophageal reflux disease	1990	0.00105754
Southeast Asia	Gastroesophageal reflux disease	2019	0.0013797
Southeast Asia	Peptic ulcer disease	1990	-0.17662708
Southeast Asia	Peptic ulcer disease	2019	-0.3370005
Southern Sub-Saharan Africa	Gastritis and duodenitis	1990	0.01295901
Southern Sub-Saharan Africa	Gastritis and duodenitis	2019	-0.02711487
Southern Sub-Saharan Africa	Gastroesophageal reflux disease	1990	0.00739189
Southern Sub-Saharan Africa	Gastroesophageal reflux disease	2019	0.00704729
Southern Sub-Saharan Africa	Peptic ulcer disease	1990	-0.12247364
Southern Sub-Saharan Africa	Peptic ulcer disease	2019	-0.22335957
Central Latin America	Gastritis and duodenitis	1990	0.01639359
Central Latin America	Gastritis and duodenitis	2019	-0.04612909
Central Latin America	Gastroesophageal reflux disease	1990	8.27E-05
Central Latin America	Gastroesophageal reflux disease	2019	-0.00034028
Central Latin America	Peptic ulcer disease	1990	-0.14998902
Central Latin America	Peptic ulcer disease	2019	-0.17395197
Central Asia	Gastritis and duodenitis	1990	-0.01604568
Central Asia	Gastritis and duodenitis	2019	-0.02073119
Central Asia	Gastroesophageal reflux disease	1990	8.07E-05
Central Asia	Gastroesophageal reflux disease	2019	-0.00026804
Central Asia	Peptic ulcer disease	1990	-0.09663013
Central Asia	Peptic ulcer disease	2019	-0.06045742
East Asia	Gastritis and duodenitis	1990	-0.00370215
East Asia	Gastritis and duodenitis	2019	-0.00924148
East Asia	Gastroesophageal reflux disease	1990	0.00095542
East Asia	Gastroesophageal reflux disease	2019	0.00163716
East Asia	Peptic ulcer disease	1990	-0.00315768
East Asia	Peptic ulcer disease	2019	-0.03936997
Andean Latin America	Gastritis and duodenitis	1990	-0.07555544
Andean Latin America	Gastritis and duodenitis	2019	0.04929671
Andean Latin America	Gastroesophageal reflux disease	1990	0.00056734
Andean Latin America	Gastroesophageal reflux disease	2019	0.00061298
Andean Latin America	Peptic ulcer disease	1990	-0.07426497
Andean Latin America	Peptic ulcer disease	2019	-0.21063466

Central Europe	Gastritis and duodenitis	1990	-0.00065956
Central Europe	Gastritis and duodenitis	2019	0.03225325
Central Europe	Gastroesophageal reflux disease	1990	0.02543019
Central Europe	Gastroesophageal reflux disease	2019	0.04809081
Central Europe	Peptic ulcer disease	1990	0.04637474
Central Europe	Peptic ulcer disease	2019	0.06011117
Eastern Europe	Gastritis and duodenitis	1990	0.03088565
Eastern Europe	Gastritis and duodenitis	2019	0.01065078
Eastern Europe	Gastroesophageal reflux disease	1990	-0.00747767
Eastern Europe	Gastroesophageal reflux disease	2019	-0.00594914
Eastern Europe	Peptic ulcer disease	1990	0.04211877
Eastern Europe	Peptic ulcer disease	2019	0.00843993
Southern Latin America	Gastritis and duodenitis	1990	0.04653634
Southern Latin America	Gastritis and duodenitis	2019	-0.00211929
Southern Latin America	Gastroesophageal reflux disease	1990	-0.0003523
Southern Latin America	Gastroesophageal reflux disease	2019	-0.00023204
Southern Latin America	Peptic ulcer disease	1990	0.04188328
Southern Latin America	Peptic ulcer disease	2019	-0.06655972
Western Europe	Gastritis and duodenitis	1990	-0.00980231
Western Europe	Gastritis and duodenitis	2019	0.04760271
Western Europe	Gastroesophageal reflux disease	1990	-0.02424695
Western Europe	Gastroesophageal reflux disease	2019	-0.02313454
Western Europe	Peptic ulcer disease	1990	0.02663864
Western Europe	Peptic ulcer disease	2019	0.13989326
High-income North America	Gastritis and duodenitis	1990	-0.02295621
High-income North America	Gastritis and duodenitis	2019	0.00292054
High-income North America	Gastroesophageal reflux disease	1990	-0.03073003
High-income North America	Gastroesophageal reflux disease	2019	-0.02286657
High-income North America	Peptic ulcer disease	1990	0.00276238
High-income North America	Peptic ulcer disease	2019	-0.00860917
High-income Asia Pacific	Gastritis and duodenitis	1990	-0.31071409
High-income Asia Pacific	Gastritis and duodenitis	2019	0.03732185
High-income Asia Pacific	Gastroesophageal reflux disease	1990	-0.02932527
High-income Asia Pacific	Gastroesophageal reflux disease	2019	0.02113496
High-income Asia Pacific	Peptic ulcer disease	1990	-0.19582723
High-income Asia Pacific	Peptic ulcer disease	2019	-0.01457433
Sub-Saharan Africa	Gastritis and duodenitis	1990	-0.07963815
Sub-Saharan Africa	Gastritis and duodenitis	2019	-0.07952785
Sub-Saharan Africa	Gastroesophageal reflux disease	1990	0.00411518
Sub-Saharan Africa	Gastroesophageal reflux disease	2019	0.00479629
Sub-Saharan Africa	Peptic ulcer disease	1990	-0.07635326
Sub-Saharan Africa	Peptic ulcer disease	2019	-0.04874283
North Africa and Middle East	Gastritis and duodenitis	1990	-0.05685651
North Africa and Middle East	Gastritis and duodenitis	2019	-0.09273786
North Africa and Middle East	Gastroesophageal reflux disease	1990	0.01015923
North Africa and Middle East	Gastroesophageal reflux disease	2019	0.01285145
North Africa and Middle East	Peptic ulcer disease	1990	-0.19852828
North Africa and Middle East	Peptic ulcer disease	2019	-0.36077394
Southeast Asia, east Asia, and Oceania	Gastritis and duodenitis	1990	-0.08180057
Southeast Asia, east Asia, and Oceania	Gastritis and duodenitis	2019	0.05123411
Southeast Asia, east Asia, and Oceania	Gastroesophageal reflux disease	1990	0.01901116
Southeast Asia, east Asia, and Oceania	Gastroesophageal reflux disease	2019	-0.02569818
Southeast Asia, east Asia, and Oceania	Peptic ulcer disease	1990	-0.08338609
Southeast Asia, east Asia, and Oceania	Peptic ulcer disease	2019	-0.22969036
South Asia	Gastritis and duodenitis	1990	0.02130459
South Asia	Gastritis and duodenitis	2019	0.03620556
South Asia	Gastroesophageal reflux disease	1990	0.00183213
South Asia	Gastroesophageal reflux disease	2019	0.00140896

South Asia	Peptic ulcer disease	1990	0.08582344
South Asia	Peptic ulcer disease	2019	0.11079084
Latin America and Caribbean	Gastritis and duodenitis	1990	-0.05162079
Latin America and Caribbean	Gastritis and duodenitis	2019	-0.0638555
Latin America and Caribbean	Gastroesophageal reflux disease	1990	-0.01628224
Latin America and Caribbean	Gastroesophageal reflux disease	2019	-0.01408388
Latin America and Caribbean	Peptic ulcer disease	1990	-0.15834846
Latin America and Caribbean	Peptic ulcer disease	2019	-0.18600901
Central Europe, eastern Europe, and central Asia	Gastritis and duodenitis	1990	-0.009517
Central Europe, eastern Europe, and central Asia	Gastritis and duodenitis	2019	0.05053715
Central Europe, eastern Europe, and central Asia	Gastroesophageal reflux disease	1990	0.00910429
Central Europe, eastern Europe, and central Asia	Gastroesophageal reflux disease	2019	0.01026362
Central Europe, eastern Europe, and central Asia	Peptic ulcer disease	1990	-0.00990068
Central Europe, eastern Europe, and central Asia	Peptic ulcer disease	2019	0.01129165
High income	Gastritis and duodenitis	1990	-0.07116142
High income	Gastritis and duodenitis	2019	-0.04401269
High income	Gastroesophageal reflux disease	1990	-0.03307023
High income	Gastroesophageal reflux disease	2019	-0.04919638
High income	Peptic ulcer disease	1990	-0.06455196
High income	Peptic ulcer disease	2019	0.0578545

y all included, SDI, and GBD regions.

SE	P	uci	lci
0.02145055	0	-0.105856502	-0.189942658
0.01731041	0	-0.128905356	-0.196762164
0.01589932	0.0019	-0.018937263	-0.081262597
0.01357872	0	-0.074411419	-0.127640001
0.02426373	0	-0.238451079	-0.333564901
0.02221985	0	-0.244362274	-0.331464086
0.05768939	0.2824	0.049961884	-0.176180524
0.04222772	0.0028	-0.054354499	-0.219887161
0.01123976	0.6433	0.02728652	-0.01677334
0.00891667	3.00E-04	0.054123023	0.019169677
0.04272582	0.1557	0.021578337	-0.145906877
0.03825852	0.0017	-0.056091091	-0.206064489
0.0214999	0.2362	0.016291714	-0.067987894
0.02455234	0.1225	0.009403036	-0.086842136
0.01231422	0.0917	0.045401591	-0.002870151
0.0104862	6.00E-04	0.059649672	0.018543768
0.02418972	0.0742	0.003094261	-0.091729441
0.02643986	0.3368	0.077518526	-0.026125726
0.03657265	0.056	-0.000367426	-0.143732214
0.02361512	0	0.156026715	0.063455445
0.04401641	0.0018	0.233508854	0.060964526
0.03746351	0	-0.13644964	-0.2833066
0.04599121	0.4427	0.125808382	-0.054477162
0.06375505	0.0357	-0.013787042	-0.263706838
0.03441281	0.8247	0.059784478	-0.075113738
0.02828903	0.2075	0.091612509	-0.019280489
0.01702056	0.2605	0.013968738	-0.052751858
0.01939072	0.7262	0.044836501	-0.031175121
0.0281123	0.5051	0.073985948	-0.036214268
0.05311895	0.0207	0.231422912	0.023196628
0.03179111	0.0023	-0.041563224	-0.166184376
0.02665442	0.0175	-0.014066977	-0.118552303
0.02302645	0.0816	0.003907182	-0.086356502
0.01667429	5.00E-04	-0.031481372	-0.096844588
0.03213966	2.00E-04	-0.069111406	-0.195098874
0.03788618	0.7698	0.063087463	-0.085426363
0.03910459	0.0916	0.005401736	-0.147888256
0.02664641	0.2887	0.022752134	-0.081701794
0.00298489	0.789	0.005034834	-0.006665934
0.00298674	0.9725	0.00595903	-0.00574899
0.05828942	0.0759	0.001819633	-0.226674893
0.05010878	0.2707	0.040570159	-0.155856259
0.02256034	0.0753	0.001477336	-0.086959196
0.01831016	0.0364	-0.005701156	-0.077476984
0.00212317	0.0742	0.008200243	-0.000122583
0.001814	0.0013	0.01053109	0.00342021
0.02959129	0.1763	0.016246598	-0.099751258
0.02747086	0.0077	-0.029204854	-0.136890626
0.02265297	0.1776	0.007388511	-0.081411131
0.02618625	0.1561	0.00565406	-0.09699604
0.00049195	0.5145	0.000612832	-0.001315612
0.00019219	0.0246	0.001051552	0.000298168
0.03809129	0.0718	-0.017988972	-0.167306828
0.07276311	0.4318	0.079070676	-0.206160716
0.0183733	0.006	-0.020844842	-0.092868178
0.02147129	4.00E-04	-0.050654132	-0.134821588

0.0060089	0.1072	0.021936674	-0.001618214
0.00816135	0.1318	0.028847696	-0.003144796
0.06098527	0.0042	-0.078997151	-0.318059409
0.07977334	2.00E-04	-0.204418194	-0.517129686
0.00957346	0.0944	0.001741502	-0.035786462
0.00488547	0	-0.020448359	-0.039599401
0.00022723	0.0014	0.001320611	0.000429869
0.00012033	0	0.000959007	0.000487313
0.04114941	0.5494	0.055485614	-0.105820074
0.02594915	0.9991	0.050829414	-0.050891254
0.02366142	0.4343	0.067680973	-0.025071793
0.00518208	0.006	0.046362437	0.026048683
0.00182354	0.3891	0.005406268	-0.001742008
0.00126752	0.3474	0.003893299	-0.001075379
0.03301532	0.0804	0.150533467	0.021113413
0.03281813	0.0432	0.175114375	0.046467305
0.0600807	0.0181	-0.039383278	-0.274899622
0.03451439	5.00E-04	-0.078858396	-0.214154804
0.00036539	0.1253	0.001305324	-0.000127004
0.00025535	0.0045	0.001334636	0.000333664
0.07104764	1.00E-04	-0.225338146	-0.503844894
0.04874201	0	-0.2546929	-0.44576158
0.06283823	0.5659	0.085968391	-0.160357471
0.03196262	0.128	0.010029295	-0.115264175
0.00340668	0.762	0.007734633	-0.005619553
0.00345294	0.6971	0.008147462	-0.005388062
0.20872635	0.4155	0.232476566	-0.585730726
0.1709159	0.0743	-0.002005336	-0.671995664
0.02211269	0.5893	0.056299882	-0.030381862
0.01421156	0.1291	0.000739788	-0.054969528
0.00072196	5.00E-04	0.008806932	0.005976848
0.00092428	0.0016	0.008858879	0.005235701
0.03070725	0.0163	-0.06228743	-0.18265985
0.04200479	0.006	-0.141030182	-0.305688958
0.06517283	0.8086	0.144132337	-0.111345157
0.06729695	0.5151	0.085772932	-0.178031112
0.00029322	0.786	0.000657441	-0.000491981
0.00030756	0.3051	0.000262538	-0.000943098
0.0632767	0.0496	-0.025966688	-0.274011352
0.12315089	0.2007	0.067423774	-0.415327714
0.04371305	0.7244	0.069631898	-0.101723258
0.03514723	0.5738	0.048157381	-0.089619761
0.00031028	0.8022	0.000688889	-0.000527409
0.00020451	0.2313	0.0001328	-0.00066888
0.062197	0.1642	0.02527599	-0.21853625
0.06115517	0.3558	0.059406713	-0.180321553
0.01618624	0.8569	0.02802288	-0.03542718
0.00464991	0.2968	-0.000127656	-0.018355304
0.00228401	0.7478	0.00543208	-0.00352124
0.00297794	0.68	0.007473922	-0.004199602
0.0060961	0.6957	0.008790676	-0.015106036
0.0320742	0.4352	0.023495462	-0.102235402
0.03026922	0.2426	-0.016227769	-0.134883111
0.08813496	0.6753	0.222041232	-0.123447812
0.00045099	0.4276	0.00145128	-0.0003166
0.00014134	0.1443	0.000890006	0.000335954
0.1598457	0.7231	0.239032602	-0.387562542
0.10917053	0.3044	0.003339579	-0.424608899

0.06858731	0.9925	0.133771568	-0.135090688
0.04287255	0.4677	0.116283448	-0.051776948
0.02955691	0.4079	0.083361734	-0.032501354
0.02718125	0.1045	0.10136606	-0.00518444
0.02442191	0.0841	0.094241684	-0.001492204
0.04217544	0.1818	0.142775032	-0.022552692
0.00659899	0.0054	0.04381967	0.01795163
0.0106859	0.3647	0.031595144	-0.010293584
0.00227295	0.0217	-0.003022688	-0.011932652
0.00259029	0.0701	-0.000872172	-0.011026108
0.02253032	0.1205	0.086278197	-0.002040657
0.02560437	0.755	0.058624495	-0.041744635
0.02188737	0.2799	0.089435585	0.003637095
0.00451918	0.7208	0.006738303	-0.010976883
9.16E-05	0.162	-0.000172744	-0.000531856
3.49E-05	0.095	-0.000163636	-0.000300444
0.01691349	0.2443	0.07503372	0.00873284
0.0529473	0.4278	0.037216988	-0.170336428
0.01951166	0.6204	0.028440544	-0.048045164
0.02103063	0.0338	0.088822745	0.006382675
0.02266994	0.2964	0.020186132	-0.068680032
0.02240591	0.313	0.020781044	-0.067050124
0.02672767	0.3298	0.079024873	-0.025747593
0.05019557	0.0107	0.238276577	0.041509943
4.54E-05	0.0013	-0.022867324	-0.023045096
0.00039273	0.0851	0.003690291	0.002150789
0.0024391	0.0504	-0.025949394	-0.035510666
0.00143271	0.0398	-0.020058458	-0.025674682
0.03395022	0.9483	0.069304811	-0.063780051
0.03020877	0.8233	0.050600019	-0.067818359
0.06012699	0.0355	-0.19286519	-0.42856299
0.01176855	0.0867	0.060388208	0.014255492
0.00515736	0.0296	-0.019216844	-0.039433696
0.0176125	0.353	0.05565546	-0.01338554
0.01810395	0.0084	-0.160343488	-0.231310972
0.02600247	0.6316	0.036390511	-0.065539171
0.03221263	0.0174	-0.016501395	-0.142774905
0.03021749	0.0117	-0.02030157	-0.13875413
0.00151556	0.0094	0.007085678	0.001144682
0.00145679	0.002	0.007651598	0.001940982
0.0279361	0.009	-0.021598504	-0.131108016
0.02982523	0.1093	0.009714621	-0.107200281
0.0183733	0.006	-0.020844842	-0.092868178
0.02147129	4.00E-04	-0.050654132	-0.134821588
0.0060089	0.1072	0.021936674	-0.001618214
0.00816135	0.1318	0.028847696	-0.003144796
0.06098527	0.0042	-0.078997151	-0.318059409
0.07977334	2.00E-04	-0.204418194	-0.517129686
0.02711719	0.005	-0.028650878	-0.134950262
0.01871539	0.01	0.087916274	0.014551946
0.0059477	0.0031	0.030668652	0.007353668
0.00606474	2.00E-04	-0.01381129	-0.03758507
0.06412674	0.2028	0.04230232	-0.2090745
0.08129238	0.0081	-0.070357295	-0.389023425
0.02366142	0.4343	0.067680973	-0.025071793
0.00518208	0.006	0.046362437	0.026048683
0.00182354	0.3891	0.005406268	-0.001742008
0.00126752	0.3474	0.003893299	-0.001075379

0.03301532	0.0804	0.150533467	0.021113413
0.03281813	0.0432	0.175114375	0.046467305
0.02871259	0.0811	0.004655886	-0.107897466
0.03595195	0.0847	0.006610322	-0.134321322
0.00385413	2.00E-04	-0.008728145	-0.023836335
0.00382431	8.00E-04	-0.006588232	-0.021579528
0.03965947	3.00E-04	-0.080615899	-0.236081021
0.05785993	0.0029	-0.072603547	-0.299414473
0.04058517	0.8164	0.070029933	-0.089063933
0.02938676	0.0969	0.1081352	-0.0070609
0.01071665	0.403	0.030108924	-0.011900344
0.010612	0.342	0.03106314	-0.0105359
0.02280226	0.6676	0.03479175	-0.05459311
0.03057625	0.7148	0.0712211	-0.0486378
0.03636361	0.0594	0.000111256	-0.142434096
0.02816053	0.1282	0.011181949	-0.099207329
0.02312426	0.1627	0.01225332	-0.07839378
0.01686819	0.0065	-0.016134728	-0.082258032
0.0326677	0.0571	-0.000523268	-0.128580652
0.03341141	0.0933	0.123340864	-0.007631864
