# Supplementary material for: The global, regional, and national patterns of change in the burden of nonmalignant upper gastrointestinal diseases from 1990 to 2019 and the forecast for the next decade
Source: Int J Surg. 2024 Jul 3;111(1):80–92. doi: 10.1097/JS9.0000000000001902 (PMC11745775; doi:10.1097/JS9.0000000000001902)
Supplement: Supplementary file 6 [file js9-111-0080-s006.pdf]

Table S5. DALYs of PUD, GD, and GERD with decomposition analysis, categorized by global and SD

Location	Cause	Overll difference	Aging	Population
Global	Peptic ulcer disease	-2166554.136	2038397.28	2872283.581
Low-middle SDI	Peptic ulcer disease	-998887.8346	873812.849	1363359.076
Low SDI	Peptic ulcer disease	-17261.84421	38488.57383	911129.6279
Middle SDI	Peptic ulcer disease	-510060.1955	838729.6115	631329.1162
High-middle SDI	Peptic ulcer disease	-328508.4567	414509.9069	244756.5213
High SDI	Peptic ulcer disease	-311537.4586	235773.004	135637.5215
Global	Gastritis and duodenitis	599203.6265	494324.5828	940362.5556
Low-middle SDI	Gastritis and duodenitis	203830.6963	127169.445	272589.2698
Low SDI	Gastritis and duodenitis	186453.9685	10294.12258	276192.3587
High-middle SDI	Gastritis and duodenitis	47287.13555	127576.6729	103748.2894
Middle SDI	Gastritis and duodenitis	145204.8363	312137.8824	297426.1248
High SDI	Gastritis and duodenitis	15848.8781	40014.79227	45853.13567
Global	Gastroesophageal reflux disease	2626124.851	636013.7417	1855332.77
Low SDI	Gastroesophageal reflux disease	410767.2274	13000.74294	398005.3987
Low-middle SDI	Gastroesophageal reflux disease	743888.1695	180554.3572	547116.1585
Middle SDI	Gastroesophageal reflux disease	863346.0516	270688.6374	487258.7533
High-middle SDI	Gastroesophageal reflux disease	390605.5062	175829.6891	250319.7439
High SDI	Gastroesophageal reflux disease	215831.5762	76284.00802	163985.6184

I regions.

Epidemiological change	Percent change of aging	Percent change of population
-7077234.997	-94.08476099	-132.5738201
-3236059.76	-87.47857554	-136.4877045
-966880.0459	-222.9690719	-5278.286703
-1980118.923	-164.437378	-123.7754135
-987774.8849	-126.1793718	-74.50539439
-682947.9842	-75.68046713	-43.53811004
-835483.5119	82.49692775	156.9353913
-195928.0185	62.38974174	133.7331789
-100032.5128	5.520999453	148.1289784
-184037.8268	269.7915014	219.400664
-464359.1708	214.9638334	204.8321064
-70019.04985	252.4771282	289.3147098
134778.3395	24.21871685	70.64906945
-238.9142574	3.164990309	96.89317262
16217.65372	24.27170704	73.54817309
105398.661	31.35343434	56.4384064
-35543.92682	45.01464682	64.08505256
-24438.05024	35.34422969	75.97851126

Percent change of epidemiological change
326.658581
323.9662801
5601.255775
388.2127915
300.6847662
219.2185772
-139.432319
-96.12292064
-53.64997783
-389.1921654
-319.7959397
-441.791838
5.132213705
-0.058162931
2.180119861
12.20815926
-9.09969938
-11.32274094
