# Supplementary material for: The global, regional, and national patterns of change in the burden of nonmalignant upper gastrointestinal diseases from 1990 to 2019 and the forecast for the next decade
Source: Int J Surg. 2024 Jul 3;111(1):80–92. doi: 10.1097/JS9.0000000000001902 (PMC11745775; doi:10.1097/JS9.0000000000001902)
Supplement: Supplementary file 7 [file js9-111-0080-s007.pdf]

Table S6. Age-standardized DALYs rates of PUD with frontier analysis across all countries and

Location	SDI	Age-standardized DALYs	Frontier DALYs
Afghanistan	0.343	255.08(173.23 to 351.83)	33.99
Albania	0.681	19.73(14.65 to 25.59)	12.64
Algeria	0.652	53.26(40.99 to 68.01)	14.97
American Samoa	0.712	99.41(80.21 to 119.73)	11.67
Andorra	0.894	11.93(8.97 to 15.25)	9.93
Angola	0.47	147.89(98.47 to 208.04)	28.28
Antigua and Barbuda	0.743	33.99(27.75 to 40.86)	11.5
Argentina	0.708	35.36(30.44 to 41.26)	11.61
Armenia	0.689	108.64(90.49 to 128.77)	12.22
Australia	0.839	14(11.95 to 16.19)	9.85
Austria	0.849	18.1(15.59 to 20.67)	9.85
Azerbaijan	0.683	66.45(52.12 to 87.63)	12.3
Bahamas	0.796	65.88(50.71 to 84.79)	11.23
Bahrain	0.751	76.88(62.41 to 93.54)	12.53
Bangladesh	0.483	36.49(25.73 to 47.42)	28.15
Barbados	0.742	57.2(45.87 to 71.38)	11.69
Belarus	0.745	49.1(37.13 to 64.41)	11.69
Belgium	0.851	21.43(18.52 to 24.65)	9.94
Belize	0.603	50.38(40.85 to 60.84)	17.72
Benin	0.352	173.31(128.16 to 230.9)	34.17
Bermuda	0.813	31.53(24.78 to 39.97)	9.94
Bhutan	0.455	150.32(77.12 to 280.11)	28.39
Bolivia (Plurinational State of)	0.566	119.39(86.62 to 158.29)	19.29
Bosnia and Herzegovina	0.718	40.22(31.95 to 50.33)	11.61
Botswana	0.634	151.98(105.9 to 220.64)	15.72
Brazil	0.64	50.64(46.87 to 54.72)	15.28
Brunei Darussalam	0.823	51.25(43.5 to 60.24)	9.84
Bulgaria	0.764	59.61(45.86 to 75.66)	11.56
Burkina Faso	0.257	127.84(80.38 to 198.82)	107.83
Burundi	0.284	209.35(118.82 to 354.13)	46.97
Cabo Verde	0.525	56.13(45.9 to 67.76)	24.68
Cambodia	0.469	427.74(330.42 to 562.93)	28.04
Cameroon	0.49	144.77(92.03 to 203.6)	28.31
Canada	0.873	19.12(16.42 to 22.04)	9.86
Central African Republic	0.274	377.18(228.76 to 573.09)	72.5
Chad	0.238	254.67(191.04 to 332.12)	85.53
Chile	0.759	22.73(19.78 to 26.19)	11.61
China	0.686	45.34(38.74 to 53.74)	11.97
Colombia	0.633	36.45(27.87 to 47.89)	15.51
Comoros	0.455	119.54(63.78 to 181.32)	28.13
Congo	0.568	121.71(82.76 to 173.44)	21.13
Cook Islands	0.764	27.37(22.24 to 33.95)	11.65
Costa Rica	0.68	40.2(29.91 to 53.45)	12.24
Croatia	0.794	45.31(35.81 to 57.83)	11.35
Cuba	0.668	43.23(34.06 to 55.16)	12.48
Cyprus	0.841	19.79(16.34 to 25.81)	10.14
Czechia	0.828	51.56(41 to 62.98)	9.84
Democratic People's Republic of Korea	0.558	144.72(87.86 to 202.76)	20.43
Democratic Republic of the Congo	0.382	135.74(92.5 to 188.39)	30.64
Denmark	0.89	47.94(41.09 to 55.6)	9.84
Djibouti	0.459	108.87(48.18 to 187.15)	28.04
Dominica	0.729	59.4(45.12 to 76.59)	11.52
Dominican Republic	0.592	84.12(55.85 to 113.34)	18.3
Ecuador	0.64	59.82(46.46 to 78.04)	15.17
Egypt	0.658	62.27(35.42 to 94.15)	13.96
El Salvador	0.573	85.07(64.08 to 110.6)	19.03

Equatorial Guinea	0.685	78.41(45.49 to 122.23)	11.88
Eritrea	0.396	186.56(133.57 to 258.88)	31.53
Estonia	0.835	56.25(42.9 to 73.15)	10
Eswatini	0.577	162.89(108.63 to 233.76)	19.08
Ethiopia	0.343	89.3(49.96 to 131.13)	32.98
Fiji	0.664	132.89(101.69 to 172.69)	12.85
Finland	0.856	29.88(25.65 to 35.24)	9.93
France	0.834	14.23(12.33 to 16.27)	10.51
Gabon	0.656	86.42(58.24 to 124.35)	15.02
Gambia	0.399	165.29(118.95 to 220.24)	30.04
Georgia	0.702	83.89(66.6 to 103.8)	11.66
Germany	0.898	28.98(25.56 to 33.26)	10.68
Ghana	0.557	58.7(44.1 to 76.23)	20.13
Greece	0.794	36.64(31.55 to 42.03)	10.92
Greenland	0.761	189.82(148.55 to 242.69)	11.68
Grenada	0.669	76.21(63.61 to 90.67)	12.42
Guam	0.813	33.82(26.68 to 41.75)	9.9
Guatemala	0.526	204.32(160 to 258.92)	25.44
Guinea	0.325	193.97(130.1 to 263.74)	35.2
Guinea-Bissau	0.355	292.77(213.77 to 391.48)	32.37
Guyana	0.618	168.26(126.37 to 220.76)	17.28
Haiti	0.432	226.71(154.76 to 315.36)	28.15
Honduras	0.496	229.8(166.69 to 301.77)	28.06
Hungary	0.791	79.25(64.43 to 96.38)	11.36
Iceland	0.869	13.52(11.4 to 15.88)	9.87
India	0.566	158.99(134.9 to 189.45)	20.17
Indonesia	0.66	23.92(19.6 to 27.45)	14.96
Iran (Islamic Republic of)	0.67	42.48(36.91 to 47.09)	12.91
Iraq	0.671	25.12(19.29 to 31.17)	12.63
Ireland	0.867	21.59(18.19 to 25.49)	9.94
Israel	0.803	9.88(8.43 to 11.84)	9.83
Italy	0.801	11.06(9.8 to 12.15)	10.16
Jamaica	0.684	74.96(57.34 to 95.35)	11.65
Japan	0.87	22.71(19.88 to 25.75)	9.83
Jordan	0.731	31.07(25.22 to 37.28)	12.03
Kazakhstan	0.723	75.53(62.9 to 91.21)	11.61
Kenya	0.508	128.65(75.73 to 188.91)	27.53
Kiribati	0.527	512.71(362.22 to 701.02)	23.48
Kuwait	0.851	21.84(17.33 to 27.74)	9.89
Kyrgyzstan	0.596	57.47(47.92 to 68.62)	17.91
Lao People's Democratic Republic	0.49	397.46(282.9 to 545.23)	28.16
Latvia	0.82	70.36(56.51 to 88.19)	9.88
Lebanon	0.708	42.35(25.19 to 66.39)	11.94
Lesotho	0.507	315.04(224.71 to 427.66)	27.54
Liberia	0.37	152.8(100.32 to 217.63)	31.76
Libya	0.709	53.49(35.49 to 76.15)	11.71
Lithuania	0.843	107.64(84.79 to 137.12)	10.04
Luxembourg	0.895	16.77(13.96 to 19.82)	10.5
Madagascar	0.396	114.8(80.67 to 153.01)	32.09
Malawi	0.384	114.71(71.3 to 165.25)	31.34
Malaysia	0.737	104.8(78.93 to 131.77)	11.91
Maldives	0.562	26.33(21.55 to 31.76)	19.66
Mali	0.263	211.39(125.03 to 321.76)	54.41
Malta	0.801	19.48(16.28 to 23.12)	10.11
Marshall Islands	0.544	246.77(162.32 to 345.24)	22.11
Mauritania	0.496	102.66(69.96 to 137.14)	27.93
Mauritius	0.705	49.55(38.91 to 61.97)	11.9
Mexico	0.649	66.07(56.15 to 76.29)	15.23

Micronesia (Federated States of)	0.58	237.53(148.65 to 337.8)	19.01
Monaco	0.902	14.03(10.8 to 17.31)	9.91
Mongolia	0.606	213.32(161.84 to 289.88)	17.82
Montenegro	0.791	46.14(37.89 to 56.2)	11.49
Morocco	0.548	76.22(57.95 to 94.52)	22.79
Mozambique	0.307	144.33(97.72 to 208.87)	43.9
Myanmar	0.521	165.5(129.51 to 226.29)	26.67
Namibia	0.612	136.52(99.07 to 190.35)	16.6
Nauru	0.618	229.91(161.9 to 314.87)	17.42
Nepal	0.422	27.97(21.27 to 39.19)	27.97
Netherlands	0.883	15.15(13.03 to 17.49)	10.66
New Zealand	0.84	18.32(15.73 to 21.05)	9.94
Nicaragua	0.517	57.75(46.52 to 74.74)	27.3
Niger	0.162	257.77(150.3 to 384.56)	127.31
Nigeria	0.515	171.11(97.14 to 301.72)	25.38
Niue	0.711	109.12(80.34 to 141.81)	11.6
North Macedonia	0.744	41.38(33.14 to 51.35)	11.56
Northern Mariana Islands	0.771	72.98(59.45 to 87.05)	11.5
Norway	0.913	30.39(26.96 to 35.47)	9.83
Oman	0.783	39.72(32.46 to 48.96)	11.64
Pakistan	0.449	98.1(74.53 to 132.19)	28
Palau	0.738	101.99(64 to 133.54)	11.56
Palestine	0.588	30.16(25.18 to 35.69)	18.69
Panama	0.686	24.37(18.27 to 32.66)	11.71
Papua New Guinea	0.394	185.91(142.86 to 243.91)	31.05
Paraguay	0.638	46.82(35.63 to 62.3)	15.5
Peru	0.648	43.65(31.6 to 59.3)	15.03
Philippines	0.623	245.46(203.15 to 295.16)	15.9
Poland	0.802	75.36(62.9 to 88.73)	10.25
Portugal	0.743	19.11(16.4 to 22.19)	11.58
Puerto Rico	0.814	14.51(11.06 to 18.83)	9.99
Qatar	0.83	28.54(22.48 to 36.12)	10.32
Republic of Korea	0.878	20.7(17.17 to 24.21)	10.3
Republic of Moldova	0.696	101.32(83.36 to 121.02)	11.62
Romania	0.76	43.35(34.95 to 52.7)	11.51
Russian Federation	0.805	106.1(91.55 to 122.43)	9.84
Rwanda	0.429	161.08(105.23 to 256.27)	28.14
Saint Kitts and Nevis	0.746	41.39(30.97 to 53.33)	11.89
Saint Lucia	0.67	51.7(41.91 to 63.82)	12.51
Saint Vincent and the Grenadines	0.627	94.34(75.65 to 115.37)	16.08
Samoa	0.641	173.41(130.22 to 225.62)	15.19
San Marino	0.884	12.61(8.83 to 17.46)	10.24
Sao Tome and Principe	0.502	68.41(48.06 to 111.11)	27.76
Saudi Arabia	0.805	34.19(26.57 to 44.8)	10.17
Senegal	0.389	160.48(96.4 to 234.31)	30.63
Serbia	0.767	83(66.58 to 102.41)	11.82
Seychelles	0.724	155.87(126.2 to 186.97)	11.79
Sierra Leone	0.347	199.65(131.59 to 282.76)	33.76
Singapore	0.861	17.4(14.36 to 20.42)	9.9
Slovakia	0.812	67.15(50.91 to 88.38)	10.16
Slovenia	0.84	31.34(23.8 to 42.59)	10.01
Solomon Islands	0.407	254.69(194.32 to 332.14)	31.89
Somalia	0.081	208.97(106.88 to 354.41)	208.97
South Africa	0.678	83.84(74.18 to 93.63)	11.96
South Sudan	0.363	114.26(61.82 to 211)	33.65
Spain	0.767	12.9(11.18 to 14.83)	11.64
Sri Lanka	0.69	11.17(8.5 to 14.98)	11.17
Sudan	0.515	91.51(52.72 to 143.97)	26.7

Suriname	0.636	107.23(85.93 to 131.43)	15.39
Sweden	0.872	27.71(24.46 to 31.04)	9.84
Switzerland	0.929	15.44(12.61 to 18.82)	9.96
Syrian Arab Republic	0.619	20.77(15.42 to 27.32)	16.63
Taiwan (Province of China)	0.868	38.31(29.52 to 49.08)	9.84
Tajikistan	0.539	91.66(72.6 to 118.53)	23.33
Thailand	0.687	34.11(25.72 to 46.45)	11.61
Timor-Leste	0.514	348.16(228.91 to 521.47)	26.59
Togo	0.417	185.76(128.38 to 249.64)	28.97
Tokelau	0.626	116.3(87.01 to 148.81)	15.65
Tonga	0.636	182.04(136.79 to 233.31)	14.61
Trinidad and Tobago	0.757	87.49(62.4 to 118.97)	11.53
Tunisia	0.672	42.36(30.51 to 58.64)	12.32
Turkey	0.748	25.87(21.12 to 31.33)	11.68
Turkmenistan	0.67	95.68(73.53 to 121.5)	13.35
Tuvalu	0.589	185.03(128.29 to 255.98)	18.68
Uganda	0.404	125.63(89.1 to 201.97)	31.17
Ukraine	0.736	105.56(85.76 to 130.06)	11.77
United Arab Emirates	0.88	46.69(32.83 to 64.96)	9.86
United Kingdom	0.847	40.31(37.36 to 43.58)	10.04
United Republic of Tanzania	0.423	64.99(37.55 to 97.62)	28.09
United States of America	0.859	20.8(18.77 to 23.03)	9.94
United States Virgin Islands	0.799	38.96(30.08 to 48.98)	9.99
Uruguay	0.697	26.99(22.57 to 31.84)	11.59
Uzbekistan	0.631	107.62(88.46 to 128.4)	15.45
Vanuatu	0.485	293.42(209.17 to 406.35)	28.13
Venezuela (Bolivarian Republic of)	0.607	51.22(38.23 to 69.42)	17.85
Viet Nam	0.617	17.11(13.05 to 22.23)	16.57
Yemen	0.412	159.2(109.32 to 235.82)	29.66
Zambia	0.505	97.22(73.75 to 135.26)	27.23
Zimbabwe	0.476	219.55(121.51 to 309.66)	28.38

l territories.

Effective difference	Effective difference rank (Age-standardized DALYs rank)
221.09	193 (195)
7.09	20 (22)
38.28	87 (85)
87.75	134 (127)
1.99	8 (4)
119.61	154 (156)
22.49	53 (50)
23.75	55 (53)
96.42	144 (137)
4.15	14 (8)
8.26	23 (17)
54.15	103 (100)
54.65	104 (98)
64.35	112 (110)
8.34	24 (55)
45.51	96 (89)
37.41	84 (77)
11.49	34 (27)
32.66	76 (79)
139.14	169 (171)
21.58	52 (48)
121.93	158 (157)
100.1	147 (144)
28.61	62 (61)
136.27	166 (158)
35.36	81 (80)
41.41	92 (82)
48.05	99 (94)
20.01	48 (148)
162.38	180 (183)
31.45	74 (87)
399.7	203 (203)
116.46	153 (155)
9.26	27 (20)
304.68	200 (201)
169.14	184 (193)
11.12	32 (31)
33.37	77 (72)
20.94	50 (54)
91.4	137 (145)
100.58	149 (146)
15.71	42 (38)
27.96	59 (60)
33.96	79 (71)
30.75	72 (68)
9.65	29 (23)
41.72	93 (83)
124.29	159 (154)
105.11	152 (151)
38.09	86 (76)
80.83	130 (138)
47.87	98 (93)
65.83	115 (116)
44.66	95 (95)
48.31	100 (96)
66.04	116 (117)

66.53	117 (111)
155.03	175 (177)
46.25	97 (88)
143.81	170 (166)
56.32	105 (120)
120.04	156 (150)
19.94	47 (43)
3.72	11 (10)
71.4	123 (118)
135.25	165 (167)
72.24	125 (115)
18.3	45 (42)
38.57	88 (92)
25.72	58 (56)
178.14	185 (178)
63.79	110 (108)
23.92	56 (49)
178.88	186 (181)
158.77	179 (179)
260.4	197 (197)
150.98	173 (169)
198.57	189 (187)
201.73	190 (188)
67.89	118 (112)
3.65	10 (7)
138.82	167 (161)
8.96	26 (32)
29.57	66 (67)
12.49	37 (34)
11.65	35 (28)
0.04	4 (1)
0.9	6 (2)
63.31	109 (105)
12.87	39 (30)
19.04	46 (46)
63.92	111 (107)
101.12	151 (149)
489.23	204 (204)
11.95	36 (29)
39.56	90 (90)
369.3	202 (202)
60.47	107 (103)
30.42	70 (65)
287.5	199 (199)
121.04	157 (159)
41.78	94 (86)
97.6	146 (136)
6.27	18 (14)
82.71	132 (142)
83.37	133 (141)
92.89	140 (131)
6.67	19 (36)
156.98	177 (184)
9.36	28 (21)
224.66	195 (192)
74.73	126 (130)
37.66	85 (78)
50.83	101 (99)

218.52	192 (190)
4.12	12 (9)
195.5	188 (185)
34.65	80 (73)
53.43	102 (109)
100.43	148 (153)
138.82	168 (168)
119.92	155 (152)
212.49	191 (189)
0	2 (40)
4.49	15 (12)
8.38	25 (18)
30.45	71 (91)
130.46	162 (196)
145.72	172 (170)
97.52	145 (139)
29.82	67 (63)
61.48	108 (104)
20.56	49 (45)
28.08	60 (59)
70.1	121 (126)
90.43	136 (129)
11.46	33 (44)
12.66	38 (33)
154.85	174 (176)
31.31	73 (75)
28.61	63 (70)
229.56	196 (191)
65.11	114 (106)
7.52	22 (19)
4.52	16 (11)
18.21	44 (41)
10.39	30 (24)
89.71	135 (128)
31.84	75 (69)
96.26	143 (133)
132.94	164 (164)
29.5	65 (64)
39.19	89 (84)
78.26	128 (123)
158.21	178 (172)
2.37	9 (5)
40.66	91 (102)
24.02	57 (52)
129.85	161 (163)
71.19	122 (113)
144.08	171 (160)
165.89	181 (180)
7.5	21 (16)
56.99	106 (101)
21.34	51 (47)
222.8	194 (194)
0	2 (182)
71.87	124 (114)
80.61	129 (140)
1.26	7 (6)
0	2 (3)
64.81	113 (121)

91.84	138 (134)
17.87	43 (39)
5.48	17 (13)
4.14	13 (25)
28.47	61 (57)
68.32	119 (122)
22.5	54 (51)
321.57	201 (200)
156.8	176 (175)
100.65	150 (143)
167.42	183 (173)
75.96	127 (119)
30.03	68 (66)
14.19	40 (35)
82.34	131 (124)
166.35	182 (174)
94.46	142 (147)
93.79	141 (132)
36.83	82 (74)
30.27	69 (62)
36.89	83 (97)
10.87	31 (26)
28.97	64 (58)
15.4	41 (37)
92.17	139 (135)
265.29	198 (198)
33.37	78 (81)
0.54	5 (15)
129.54	160 (162)
69.99	120 (125)
191.17	187 (186)
