# Supplementary material for: The global, regional, and national patterns of change in the burden of nonmalignant upper gastrointestinal diseases from 1990 to 2019 and the forecast for the next decade
Source: Int J Surg. 2024 Jul 3;111(1):80–92. doi: 10.1097/JS9.0000000000001902 (PMC11745775; doi:10.1097/JS9.0000000000001902)
Supplement: Supplementary file 8 [file js9-111-0080-s008.pdf]

Table S7. Age-standardized DALYs rates of GD with frontier analysis across all countries and t

Location	SDI	Age-standardized DALYs	Frontier DALYs
Afghanistan	0.343	28.78(19.56 to 41.46)	18.21
Albania	0.681	24.16(15.14 to 36.3)	11.53
Algeria	0.652	16.3(10.36 to 23.92)	11.53
American Samoa	0.712	24.83(16.81 to 35.77)	8.7
Andorra	0.894	16.33(10.27 to 24.36)	6.88
Angola	0.47	93.92(62.16 to 130.33)	15.19
Antigua and Barbuda	0.743	42.23(31.09 to 56.82)	8.35
Argentina	0.708	18.48(12.56 to 26.66)	8.78
Armenia	0.689	18.54(13.23 to 25.22)	10.59
Australia	0.839	19.37(12.24 to 29.19)	7.46
Austria	0.849	17.43(11.35 to 25.84)	7.1
Azerbaijan	0.683	14.94(9.58 to 21.8)	11.56
Bahamas	0.796	38.78(28.22 to 52.43)	7.96
Bahrain	0.751	16.61(11.26 to 23.67)	8.28
Bangladesh	0.483	30.78(19.94 to 44.61)	15.08
Barbados	0.742	28.59(19.63 to 40.3)	8.53
Belarus	0.745	18.52(12.13 to 27)	8.62
Belgium	0.851	18.38(11.54 to 27.62)	7.01
Belize	0.603	51.21(37.41 to 67.97)	11.53
Benin	0.352	40.45(28.56 to 55.23)	19.67
Bermuda	0.813	30.41(20.71 to 43.91)	7.89
Bhutan	0.455	30.84(20.75 to 43.42)	15.43
Bolivia (Plurinational State of)	0.566	54.95(40.36 to 72.41)	13.29
Bosnia and Herzegovina	0.718	22.87(14.72 to 33.17)	8.76
Botswana	0.634	72.54(52.32 to 97.7)	11.51
Brazil	0.64	28.35(20.41 to 39.28)	11.55
Brunei Darussalam	0.823	9.87(6.93 to 13.66)	7.65
Bulgaria	0.764	34.46(24.48 to 46.15)	8.26
Burkina Faso	0.257	37.23(25.36 to 52.57)	20.83
Burundi	0.284	107.22(68.96 to 159.26)	20.82
Cabo Verde	0.525	20.81(14.04 to 29.38)	13.43
Cambodia	0.469	42.15(29.37 to 56.79)	15.07
Cameroon	0.49	33.48(22.99 to 46)	14.97
Canada	0.873	17.89(12.05 to 25.7)	6.88
Central African Republic	0.274	162.44(100.26 to 272.14)	20.82
Chad	0.238	53.7(38.16 to 71.86)	22.73
Chile	0.759	18.13(12.3 to 25.81)	8.27
China	0.686	44.27(33.63 to 57.06)	11.52
Colombia	0.633	20.3(15.1 to 26.93)	11.56
Comoros	0.455	90.92(61.39 to 129.12)	15.1
Congo	0.568	83.19(56.02 to 115.64)	11.66
Cook Islands	0.764	20.1(12.72 to 30.11)	8.25
Costa Rica	0.68	13.65(9.3 to 19.76)	11.52
Croatia	0.794	19.94(13.72 to 27.89)	7.98
Cuba	0.668	39.74(28.06 to 54.13)	11.53
Cyprus	0.841	11.34(7.12 to 17.06)	7.3
Czechia	0.828	21.63(14.29 to 31.47)	7.53
Democratic People's Republic of Korea	0.558	50.57(34.78 to 72.42)	12.9
Democratic Republic of the Congo	0.382	98.76(66.53 to 135.45)	18.13
Denmark	0.89	17.62(11.73 to 25.11)	6.88
Djibouti	0.459	71.7(46.44 to 100.33)	15.08
Dominica	0.729	42.64(31 to 58.08)	8.7
Dominican Republic	0.592	37.44(25.96 to 52.88)	11.51
Ecuador	0.64	34.87(25.96 to 45.64)	11.55
Egypt	0.658	15.83(10.29 to 23.07)	11.51
El Salvador	0.573	17.72(12.73 to 24.5)	11.56

Equatorial Guinea	0.685	68.79(46.68 to 94.83)	11.52
Eritrea	0.396	115.97(73.04 to 165.69)	17.18
Estonia	0.835	17.62(11.54 to 25.75)	7.5
Eswatini	0.577	74.43(54.52 to 100.23)	11.54
Ethiopia	0.343	76(49.29 to 105.82)	18.58
Fiji	0.664	23.57(15.54 to 34.18)	11.52
Finland	0.856	18.15(12.73 to 24.97)	6.92
France	0.834	15.71(9.69 to 23.87)	7.57
Gabon	0.656	63.79(44.06 to 86.2)	11.52
Gambia	0.399	37.07(25.68 to 49.98)	16.72
Georgia	0.702	17.37(11.91 to 24.21)	8.96
Germany	0.898	19.61(13.31 to 27.79)	6.88
Ghana	0.557	28.2(19.53 to 40.07)	13.21
Greece	0.794	15.99(9.89 to 23.98)	8.13
Greenland	0.761	18.65(12.91 to 26.97)	8.27
Grenada	0.669	53.86(41.23 to 68.89)	11.53
Guam	0.813	20.92(12.99 to 31.56)	7.84
Guatemala	0.526	47.93(37.64 to 60.81)	13.52
Guinea	0.325	42.94(28.27 to 60.17)	18.37
Guinea-Bissau	0.355	53.18(38.71 to 71.9)	18.6
Guyana	0.618	57.13(42.93 to 74.79)	11.59
Haiti	0.432	75.44(52.71 to 104.02)	15.53
Honduras	0.496	66.67(46.12 to 92.71)	13.94
Hungary	0.791	25.75(17.63 to 36.4)	8.24
Iceland	0.869	14.39(9.38 to 20.97)	6.88
India	0.566	39.4(27.56 to 55.25)	12.59
Indonesia	0.66	22.65(14.41 to 33.66)	11.55
Iran (Islamic Republic of)	0.67	21.24(14.14 to 30.77)	11.53
Iraq	0.671	16.76(10.98 to 24.53)	11.54
Ireland	0.867	18.08(12.07 to 26.59)	6.88
Israel	0.803	16.48(10.3 to 24.51)	7.88
Italy	0.801	13.97(9.43 to 20.07)	7.92
Jamaica	0.684	37.02(26.45 to 51.67)	11.51
Japan	0.87	7.2(4.68 to 10.53)	6.89
Jordan	0.731	13.43(8.49 to 20.19)	8.77
Kazakhstan	0.723	17.64(11.75 to 24.85)	8.75
Kenya	0.508	93.79(62.69 to 127.09)	13.89
Kiribati	0.527	32.78(22.58 to 45.19)	13.53
Kuwait	0.851	14.01(8.82 to 21.14)	7.06
Kyrgyzstan	0.596	16.25(10.43 to 23.95)	11.52
Lao People's Democratic Republic	0.49	41.59(27.38 to 58.32)	14.87
Latvia	0.82	20.89(14.31 to 29.34)	7.9
Lebanon	0.708	16.66(10.78 to 24.66)	8.74
Lesotho	0.507	102.81(71.9 to 137.59)	13.76
Liberia	0.37	36.44(25.04 to 50.91)	17.65
Libya	0.709	16.85(11.02 to 24.65)	8.79
Lithuania	0.843	21.12(14.52 to 29.58)	7.23
Luxembourg	0.895	24.75(15.65 to 37.41)	6.88
Madagascar	0.396	87.05(58.69 to 116.62)	17.18
Malawi	0.384	87.11(62.43 to 118.47)	18.12
Malaysia	0.737	19.1(12.2 to 28.02)	8.66
Maldives	0.562	22.86(13.93 to 34.97)	13.05
Mali	0.263	49.49(33.41 to 69.68)	20.81
Malta	0.801	28.11(18.82 to 41.17)	7.85
Marshall Islands	0.544	26.57(17.37 to 37.54)	13.1
Mauritania	0.496	31.93(22.31 to 45.17)	14.57
Mauritius	0.705	22.65(16.04 to 31.45)	8.79
Mexico	0.649	32.34(27.29 to 39.15)	11.55

Micronesia (Federated States of)	0.58	27.18(17.9 to 37.98)	11.56
Monaco	0.902	15.87(9.82 to 23.87)	6.88
Mongolia	0.606	29.99(21.6 to 40.66)	11.53
Montenegro	0.791	22.54(14.01 to 33.93)	8.23
Morocco	0.548	18.57(12.36 to 26.56)	12.61
Mozambique	0.307	92.7(60.89 to 134.95)	20.81
Myanmar	0.521	32.79(22.65 to 45.92)	13.39
Namibia	0.612	66.51(47.48 to 88.62)	11.54
Nauru	0.618	26.07(17.28 to 37.16)	11.53
Nepal	0.422	29.08(18.68 to 43.25)	15.56
Netherlands	0.883	16.6(10.52 to 24.64)	6.88
New Zealand	0.84	23.04(15.3 to 33.01)	7.42
Nicaragua	0.517	27.21(20.62 to 34.61)	13.53
Niger	0.162	56.62(36.45 to 82.36)	36.63
Nigeria	0.515	42.23(28.47 to 63.78)	13.52
Niue	0.711	22.88(15.05 to 33.11)	8.8
North Macedonia	0.744	21.42(13.42 to 31.97)	8.36
Northern Mariana Islands	0.771	20.83(13.21 to 30.83)	8.25
Norway	0.913	24.42(16.03 to 36.52)	6.88
Oman	0.783	15.27(9.45 to 22.89)	8.2
Pakistan	0.449	32.61(21.87 to 46.51)	15.46
Palau	0.738	31.06(21.53 to 43.02)	8.73
Palestine	0.588	17.74(11.52 to 26.49)	11.53
Panama	0.686	15.56(10.97 to 21.85)	11.52
Papua New Guinea	0.394	30.74(19.93 to 44.75)	17.3
Paraguay	0.638	29.74(19.53 to 43.52)	11.52
Peru	0.648	57.27(42.18 to 76.33)	11.51
Philippines	0.623	31.72(22.83 to 43.3)	11.52
Poland	0.802	41.72(28.58 to 58.3)	7.85
Portugal	0.743	16.65(10.57 to 24.84)	8.71
Puerto Rico	0.814	26.63(16.85 to 40.07)	7.8
Qatar	0.83	16.03(10.8 to 23.07)	7.52
Republic of Korea	0.878	8.66(5.83 to 12.44)	6.88
Republic of Moldova	0.696	19.86(12.48 to 29.56)	10.98
Romania	0.76	30.33(21.61 to 42.05)	8.27
Russian Federation	0.805	23.67(16.96 to 33.04)	7.86
Rwanda	0.429	87.49(60.57 to 121.61)	15.59
Saint Kitts and Nevis	0.746	43.79(31.22 to 58.98)	8.7
Saint Lucia	0.67	37.41(26.34 to 51.38)	11.52
Saint Vincent and the Grenadines	0.627	78.45(61.58 to 99.24)	11.53
Samoa	0.641	22.47(14.68 to 32.41)	11.53
San Marino	0.884	16.73(10.66 to 24.79)	6.88
Sao Tome and Principe	0.502	22.41(15.25 to 32.24)	14.15
Saudi Arabia	0.805	13.88(8.87 to 20.43)	7.86
Senegal	0.389	36.52(24.12 to 50.82)	17.71
Serbia	0.767	23.11(16.25 to 32.48)	8.26
Seychelles	0.724	28.27(19.5 to 38.93)	8.81
Sierra Leone	0.347	42.77(29.31 to 58.52)	18.89
Singapore	0.861	7.43(4.74 to 11.24)	6.92
Slovakia	0.812	26.76(18.54 to 37.76)	7.79
Slovenia	0.84	24.19(15.55 to 35.36)	7.33
Solomon Islands	0.407	28.21(18.82 to 40.26)	15.54
Somalia	0.081	108.6(64.8 to 178.44)	75.14
South Africa	0.678	70.63(57.09 to 87.57)	11.53
South Sudan	0.363	84.87(54.65 to 126.24)	18.64
Spain	0.767	16.57(10.27 to 24.27)	8.26
Sri Lanka	0.69	28.93(20.42 to 40.36)	11.1
Sudan	0.515	23.55(15.59 to 34.47)	13.54

Suriname	0.636	42.8(31.97 to 57.05)	11.57
Sweden	0.872	12.92(8.38 to 18.88)	6.88
Switzerland	0.929	15.27(10.12 to 22.05)	6.88
Syrian Arab Republic	0.619	16.14(10.48 to 23.58)	11.51
Taiwan (Province of China)	0.868	25.45(15.61 to 38.2)	6.88
Tajikistan	0.539	19.91(13.69 to 28.11)	13.08
Thailand	0.687	26.34(17.99 to 37.7)	11.53
Timor-Leste	0.514	42.77(26.93 to 66.2)	13.53
Togo	0.417	43.63(30.48 to 60.36)	15.58
Tokelau	0.626	24.06(15.94 to 34.37)	11.54
Tonga	0.636	26.24(17.28 to 38.06)	11.59
Trinidad and Tobago	0.757	73.85(55.33 to 95.5)	8.27
Tunisia	0.672	16.06(10.5 to 23.71)	11.52
Turkey	0.748	16(10.14 to 23.7)	8.5
Turkmenistan	0.67	16.16(10.65 to 23.59)	11.54
Tuvalu	0.589	26.14(17.33 to 36.77)	11.54
Uganda	0.404	81.52(56.07 to 111.76)	16.66
Ukraine	0.736	22.68(15.47 to 32.84)	8.7
United Arab Emirates	0.88	13.93(8.9 to 20.7)	6.88
United Kingdom	0.847	24.35(17 to 33.93)	7.11
United Republic of Tanzania	0.423	56(39.09 to 76.02)	15.49
United States of America	0.859	17.32(11.58 to 24.36)	6.92
United States Virgin Islands	0.799	32.2(22.27 to 45.73)	7.96
Uruguay	0.697	17.85(11.96 to 25.36)	10.24
Uzbekistan	0.631	15.53(9.67 to 23.18)	11.52
Vanuatu	0.485	31.46(21.33 to 44.93)	15.05
Venezuela (Bolivarian Republic of)	0.607	26.1(19.52 to 34.02)	11.53
Viet Nam	0.617	20.44(13.03 to 30.85)	11.52
Yemen	0.412	26.92(18.36 to 38.06)	15.7
Zambia	0.505	72.38(52.45 to 96.29)	13.56
Zimbabwe	0.476	80.27(53.22 to 112.23)	15.02

territories.

Effective difference	Effective difference rank (Age-standardized DALYs rank)
10.57	64 (116)
12.63	79 (91)
4.76	16 (29)
16.13	108 (96)
9.45	50 (30)
78.73	198 (198)
33.88	164 (154)
9.7	51 (54)
7.95	35 (56)
11.91	74 (60)
10.33	60 (42)
3.38	6 (14)
30.83	158 (146)
8.33	40 (34)
15.7	106 (124)
20.06	132 (115)
9.9	57 (55)
11.37	72 (53)
39.68	170 (166)
20.78	137 (149)
22.52	141 (122)
15.41	103 (125)
41.66	172 (170)
14.11	94 (83)
61.02	185 (182)
16.79	111 (114)
2.22	5 (4)
26.2	149 (136)
16.4	109 (143)
86.4	201 (201)
7.37	28 (68)
27.08	152 (152)
18.5	122 (135)
11.01	67 (49)
141.62	204 (204)
30.97	159 (168)
9.86	56 (51)
32.75	161 (162)
8.74	45 (66)
75.82	197 (195)
71.53	194 (190)
11.86	73 (65)
2.12	4 (8)
11.96	75 (64)
28.21	154 (148)
4.04	8 (5)
14.1	93 (75)
37.66	169 (165)
80.63	200 (199)
10.75	65 (43)
56.62	179 (180)
33.94	165 (155)
25.93	148 (145)
23.32	142 (137)
4.32	10 (20)
6.15	22 (46)

57.28	180 (178)
98.79	203 (203)
10.12	59 (44)
62.89	186 (184)
57.42	181 (186)
12.05	76 (88)
11.23	71 (52)
8.14	37 (19)
52.27	176 (175)
20.35	135 (142)
8.41	42 (41)
12.73	81 (61)
14.99	102 (111)
7.86	32 (22)
10.37	61 (58)
42.32	173 (169)
13.08	84 (71)
34.41	166 (163)
24.57	145 (159)
34.58	167 (167)
45.54	174 (173)
59.91	184 (185)
52.73	177 (177)
17.51	116 (98)
7.51	30 (13)
26.81	151 (147)
11.1	68 (80)
9.71	52 (73)
5.23	17 (38)
11.2	69 (50)
8.59	44 (31)
6.05	21 (11)
25.51	146 (141)
0.31	1 (1)
4.67	14 (7)
8.89	47 (45)
79.9	199 (197)
19.25	128 (133)
6.95	25 (12)
4.73	15 (28)
26.72	150 (150)
12.99	82 (70)
7.92	33 (36)
89.04	202 (200)
18.79	124 (139)
8.06	36 (39)
13.89	90 (72)
17.88	119 (95)
69.87	193 (192)
68.99	192 (193)
10.45	63 (59)
9.81	54 (82)
28.67	155 (164)
20.26	134 (110)
13.47	86 (104)
17.37	115 (129)
13.87	89 (79)
20.79	138 (131)

15.62	104 (108)
9	49 (21)
18.46	121 (120)
14.31	95 (78)
5.96	18 (57)
71.88	195 (196)
19.4	129 (134)
54.96	178 (176)
14.54	96 (99)
13.52	87 (118)
9.72	53 (33)
15.62	105 (85)
13.68	88 (109)
19.99	131 (172)
28.7	156 (153)
14.08	92 (84)
13.07	83 (74)
12.58	78 (69)
17.54	117 (94)
7.07	27 (16)
17.14	113 (132)
22.33	140 (126)
6.22	23 (47)
4.04	9 (18)
13.44	85 (123)
18.23	120 (119)
45.75	175 (174)
20.2	133 (128)
33.86	163 (151)
7.94	34 (35)
18.83	126 (105)
8.5	43 (24)
1.78	3 (3)
8.88	46 (62)
22.06	139 (121)
15.81	107 (89)
71.9	196 (194)
35.09	168 (161)
25.9	147 (144)
66.92	191 (187)
10.94	66 (77)
9.85	55 (37)
8.26	38 (76)
6.02	19 (9)
18.82	125 (140)
14.85	101 (86)
19.45	130 (113)
23.88	143 (156)
0.51	2 (2)
18.96	127 (106)
16.86	112 (92)
12.67	80 (112)
33.46	162 (202)
59.1	183 (179)
66.23	190 (191)
8.31	39 (32)
17.83	118 (117)
10.02	58 (87)

31.23	160 (158)
6.04	20 (6)
8.38	41 (15)
4.62	13 (26)
18.57	123 (97)
6.83	24 (63)
14.81	100 (103)
29.23	157 (157)
28.05	153 (160)
12.52	77 (90)
14.66	99 (102)
65.58	189 (183)
4.53	11 (25)
7.49	29 (23)
4.62	12 (27)
14.6	98 (101)
64.86	187 (189)
13.97	91 (81)
7.05	26 (10)
17.24	114 (93)
40.51	171 (171)
10.4	62 (40)
24.25	144 (130)
7.61	31 (48)
4.01	7 (17)
16.4	110 (127)
14.57	97 (100)
8.92	48 (67)
11.22	70 (107)
58.82	182 (181)
65.24	188 (188)
