# Supplementary material for: The global, regional, and national patterns of change in the burden of nonmalignant upper gastrointestinal diseases from 1990 to 2019 and the forecast for the next decade
Source: Int J Surg. 2024 Jul 3;111(1):80–92. doi: 10.1097/JS9.0000000000001902 (PMC11745775; doi:10.1097/JS9.0000000000001902)
Supplement: Supplementary file 9 [file js9-111-0080-s009.pdf]

Table S8. Age-standardized DALYs rates of GERD with frontier analysis across all countries and territories.

Location	SDI	Age-standardized DALYs	Frontier DALYs	Effective difference
Afghanistan	0.343	92.21(47.65 to 167.08)	40.39	51.82
Albania	0.681	50.67(25.97 to 92.11)	31.4	19.27
Algeria	0.652	93.66(48.63 to 169.01)	32.02	61.64
American Samoa	0.712	40.5(20.66 to 74.2)	31.39	9.12
Andorra	0.894	60.04(30.75 to 109.03)	31.43	28.61
Angola	0.47	84.83(44.07 to 151.86)	34.32	50.51
Antigua and Barbuda	0.743	122.96(63.84 to 217.33)	31.29	91.67
Argentina	0.708	105.14(54.26 to 187.94)	31.4	73.74
Armenia	0.689	80.92(41.33 to 147.23)	31.41	49.52
Australia	0.839	65.36(33.27 to 118.9)	31.41	33.95
Austria	0.849	78.52(40.18 to 140.19)	31.38	47.14
Azerbaijan	0.683	80.87(41.37 to 146.52)	31.38	49.5
Bahamas	0.796	123.09(64.04 to 217.48)	31.33	91.77
Bahrain	0.751	91.14(46.92 to 164.49)	31.4	59.74
Bangladesh	0.483	100.18(52.01 to 179.08)	33.88	66.3
Barbados	0.742	123.24(64.16 to 218.35)	31.44	91.8
Belarus	0.745	87.13(44.48 to 156.14)	31.44	55.7
Belgium	0.851	65.6(33.14 to 117.89)	31.51	34.1
Belize	0.603	123(63.83 to 218.39)	32.31	90.69
Benin	0.352	85.06(43.87 to 152.23)	40.36	44.7
Bermuda	0.813	123.42(64.09 to 218.38)	31.4	92.02
Bhutan	0.455	100.27(51.81 to 182.11)	34.53	65.74
Bolivia (Plurinational State of)	0.566	122.87(63.88 to 218.07)	33.41	89.46
Bosnia and Herzegovina	0.718	74.24(37.97 to 133.88)	31.47	42.77
Botswana	0.634	84.1(43.77 to 150.21)	31.73	52.37
Brazil	0.64	124.74(64.6 to 222.06)	31.88	92.86
Brunei Darussalam	0.823	49.83(25.58 to 90.21)	31.36	18.47
Bulgaria	0.764	74.47(38.12 to 134.13)	31.4	43.07
Burkina Faso	0.257	85.21(44.09 to 152)	83.76	1.44
Burundi	0.284	84.44(43.59 to 150.92)	40.65	43.79
Cabo Verde	0.525	85.18(43.99 to 152.87)	33.41	51.76
Cambodia	0.469	40.92(20.81 to 74.92)	33.82	7.09
Cameroon	0.49	84.82(43.83 to 151.72)	33.86	50.96
Canada	0.873	55.3(28.34 to 100.28)	31.28	24.02
Central African Republic	0.274	84.43(43.87 to 150.61)	41.14	43.29
Chad	0.238	84.71(43.81 to 151.35)	83.82	0.89
Chile	0.759	105.03(53.95 to 187.75)	31.29	73.74
China	0.686	34.94(17.73 to 63.02)	31.47	3.46
Colombia	0.633	123.23(63.88 to 218.22)	31.5	91.73
Comoros	0.455	85.1(44.13 to 152.4)	34.47	50.63
Congo	0.568	84.62(43.94 to 150.79)	33.47	51.15
Cook Islands	0.764	40.72(20.95 to 74.13)	31.37	9.34
Costa Rica	0.68	123.1(64.09 to 218.52)	31.4	91.7
Croatia	0.794	74.25(37.85 to 133.59)	31.47	42.77
Cuba	0.668	122.85(64.23 to 217.61)	31.41	91.44
Cyprus	0.841	60.23(30.83 to 108.84)	31.35	28.88
Czechia	0.828	74.09(38.08 to 133.37)	31.34	42.75
Democratic People's Republic of Korea	0.558	36.24(18.5 to 65.15)	33.42	2.82
Democratic Republic of the Congo	0.382	84.51(43.89 to 150.49)	40.38	44.13
Denmark	0.89	74.28(38.37 to 132.3)	31.36	42.92
Djibouti	0.459	84.86(43.81 to 150.7)	34	50.86
Dominica	0.729	122.64(63.77 to 217.36)	31.3	91.35
Dominican Republic	0.592	123.03(63.96 to 217.58)	33.26	89.77
Ecuador	0.64	123.09(63.94 to 217.87)	31.32	91.77
Egypt	0.658	93.37(48.28 to 168.4)	31.38	61.99
El Salvador	0.573	122.96(63.99 to 217.11)	33.37	89.58

Equatorial Guinea	0.685	84.6(43.77 to 151.22)	31.4	53.2
Eritrea	0.396	84.62(43.68 to 151.77)	40.27	44.36
Estonia	0.835	87(44.46 to 155.83)	31.28	55.72
Eswatini	0.577	84.01(43.55 to 149.39)	33.38	50.63
Ethiopia	0.343	88.06(45.23 to 158.29)	40.39	47.67
Fiji	0.664	40.52(20.47 to 74.46)	31.51	9.01
Finland	0.856	80.06(41.03 to 143.9)	31.33	48.73
France	0.834	53.16(26.92 to 95.68)	31.45	21.7
Gabon	0.656	84.65(43.69 to 151.12)	31.29	53.36
Gambia	0.399	84.84(43.81 to 151.64)	40.32	44.52
Georgia	0.702	80.73(41.35 to 146.78)	31.43	49.3
Germany	0.898	58.97(29.97 to 105.58)	31.34	27.63
Ghana	0.557	85.17(44.29 to 152.31)	33.43	51.73
Greece	0.794	78.79(40.5 to 144.24)	31.34	47.45
Greenland	0.761	62.32(32.08 to 112.36)	31.41	30.91
Grenada	0.669	122.67(63.77 to 217.15)	31.38	91.29
Guam	0.813	40.79(20.73 to 74.4)	31.4	9.39
Guatemala	0.526	122.65(63.88 to 216.19)	33.43	89.22
Guinea	0.325	85.02(44.2 to 152.12)	40.4	44.62
Guinea-Bissau	0.355	84.94(44.25 to 151.8)	40.37	44.58
Guyana	0.618	122.06(63.61 to 216.09)	32	90.06
Haiti	0.432	122.34(63.92 to 216.22)	40.33	82.01
Honduras	0.496	122.89(63.78 to 217.2)	33.96	88.93
Hungary	0.791	77.94(39.82 to 139.24)	31.26	46.68
Iceland	0.869	48.26(24.27 to 87.58)	31.42	16.84
India	0.566	102.26(53.1 to 184.31)	33.39	68.87
Indonesia	0.66	42.69(21.62 to 76.51)	31.4	11.29
Iran (Islamic Republic of)	0.67	88.01(44.53 to 158.39)	31.42	56.58
Iraq	0.671	93.1(48.02 to 168.51)	31.42	61.68
Ireland	0.867	60.19(30.52 to 109.23)	31.27	28.92
Israel	0.803	64.63(32.81 to 116.78)	31.38	33.26
Italy	0.801	74.71(38.33 to 134.16)	31.46	43.26
Jamaica	0.684	122.96(64.16 to 217.35)	31.43	91.53
Japan	0.87	45.89(23.33 to 83.33)	31.43	14.46
Jordan	0.731	93.09(48.03 to 168.08)	31.39	61.7
Kazakhstan	0.723	80.7(41.32 to 145.69)	31.35	49.35
Kenya	0.508	88.02(45.12 to 158.02)	33.43	54.59
Kiribati	0.527	40.72(20.81 to 74.69)	33.44	7.28
Kuwait	0.851	92.75(48.03 to 168.06)	31.4	61.35
Kyrgyzstan	0.596	81.01(41.34 to 146.37)	33.28	47.73
Lao People's Democratic Republic	0.49	40.85(20.91 to 74.8)	33.9	6.95
Latvia	0.82	87.02(44.59 to 155.7)	31.31	55.72
Lebanon	0.708	93.84(48.6 to 169.69)	31.4	62.44
Lesotho	0.507	83.97(43.64 to 149.43)	33.45	50.52
Liberia	0.37	84.12(43.73 to 149.83)	40.33	43.79
Libya	0.709	93.07(48.21 to 168.06)	31.38	61.69
Lithuania	0.843	91.96(47.19 to 165.83)	31.44	60.52
Luxembourg	0.895	60(30.51 to 109.14)	31.41	28.59
Madagascar	0.396	84.91(43.73 to 151.91)	40.28	44.63
Malawi	0.384	84.8(43.98 to 151.79)	40.32	44.48
Malaysia	0.737	40.77(20.94 to 75.49)	31.33	9.44
Maldives	0.562	40.6(20.61 to 74.48)	33.44	7.16
Mali	0.263	84.97(43.95 to 152.49)	83.71	1.26
Malta	0.801	60.1(30.69 to 109.14)	31.48	28.63
Marshall Islands	0.544	40.43(20.77 to 74.34)	33.44	6.99
Mauritania	0.496	85.2(44.23 to 152.01)	33.94	51.26
Mauritius	0.705	40.66(20.69 to 74.71)	31.52	9.14
Mexico	0.649	122.74(64.16 to 220.76)	32.31	90.43

Micronesia (Federated States of)	0.58	40.66(20.78 to 74.38)	33.37	7.29
Monaco	0.902	60.22(30.58 to 108.75)	31.39	28.83
Mongolia	0.606	80.89(41.37 to 147.09)	33.25	47.64
Montenegro	0.791	74.39(38.05 to 134.22)	31.31	43.08
Morocco	0.548	93.56(48.18 to 169.11)	33.39	60.17
Mozambique	0.307	84.46(43.93 to 150.74)	40.54	43.92
Myanmar	0.521	40.92(21 to 74.95)	33.42	7.5
Namibia	0.612	84.45(43.85 to 151.24)	32.54	51.91
Nauru	0.618	40.75(20.76 to 74.28)	31.52	9.23
Nepal	0.422	100.46(51.93 to 181.31)	40.34	60.12
Netherlands	0.883	43.35(22.02 to 78.99)	31.35	12
New Zealand	0.84	74.01(37.79 to 133.73)	31.38	42.63
Nicaragua	0.517	122.9(64.02 to 217.16)	33.42	89.47
Niger	0.162	85.11(44.32 to 152.15)	84.33	0.78
Nigeria	0.515	88.18(45.28 to 158.46)	33.44	54.74
Niue	0.711	40.58(20.91 to 74.63)	31.4	9.18
North Macedonia	0.744	74.33(38.31 to 134.38)	31.33	43
Northern Mariana Islands	0.771	40.66(20.55 to 74.69)	31.41	9.25
Norway	0.913	39.1(19.98 to 71.24)	31.34	7.76
Oman	0.783	91.09(46.6 to 165.1)	31.4	59.68
Pakistan	0.449	102.28(52.44 to 185.09)	35.68	66.61
Palau	0.738	40.41(20.58 to 73.88)	31.4	9.01
Palestine	0.588	93.14(48.33 to 168.18)	33.33	59.81
Panama	0.686	122.93(64.06 to 217.39)	31.45	91.48
Papua New Guinea	0.394	40.43(20.54 to 73.6)	40.31	0.12
Paraguay	0.638	125.91(65.11 to 223.26)	31.85	94.06
Peru	0.648	123.26(64.08 to 218.57)	31.32	91.93
Philippines	0.623	42.73(21.68 to 76.63)	32.33	10.4
Poland	0.802	105.34(53.64 to 185.83)	31.26	74.08
Portugal	0.743	65.16(33.15 to 115.74)	31.35	33.8
Puerto Rico	0.814	122.96(63.89 to 217.75)	31.41	91.55
Qatar	0.83	88.75(45.71 to 160.37)	31.44	57.31
Republic of Korea	0.878	52.51(26.67 to 94.45)	31.41	21.1
Republic of Moldova	0.696	86.99(44.31 to 154.88)	31.3	55.69
Romania	0.76	74.51(38.09 to 135)	31.41	43.1
Russian Federation	0.805	86.19(43.95 to 157.49)	31.27	54.91
Rwanda	0.429	84.81(43.99 to 152.08)	40.32	44.49
Saint Kitts and Nevis	0.746	122.76(63.87 to 217.32)	31.42	91.34
Saint Lucia	0.67	122.59(63.82 to 216.58)	31.34	91.25
Saint Vincent and the Grenadines	0.627	122.64(63.87 to 217.49)	32.24	90.4
Samoa	0.641	40.68(20.66 to 74.96)	32.87	7.81
San Marino	0.884	60.33(30.73 to 109.56)	31.42	28.91
Sao Tome and Principe	0.502	85.05(44 to 151.63)	33.52	51.53
Saudi Arabia	0.805	91.6(47.6 to 164.95)	31.42	60.18
Senegal	0.389	84.91(44.04 to 151.92)	40.18	44.73
Serbia	0.767	74.33(38.08 to 134.55)	31.43	42.89
Seychelles	0.724	40.7(20.85 to 74.85)	31.42	9.29
Sierra Leone	0.347	84.85(43.9 to 151.72)	40.38	44.47
Singapore	0.861	56.94(29.19 to 101.88)	31.42	25.52
Slovakia	0.812	74.33(38.13 to 134.35)	31.4	42.92
Slovenia	0.84	74.25(37.97 to 133.8)	31.23	43.02
Solomon Islands	0.407	40.62(20.59 to 74.24)	40.33	0.29
Somalia	0.081	84.62(43.81 to 150.69)	84.47	0.15
South Africa	0.678	87.46(45.04 to 156.38)	31.36	56.1
South Sudan	0.363	84.09(43.5 to 149.57)	40.32	43.76
Spain	0.767	54.06(27.5 to 97.4)	31.36	22.7
Sri Lanka	0.69	40.85(20.85 to 74.64)	31.41	9.44
Sudan	0.515	93.45(48.38 to 169.37)	33.46	59.98

Suriname	0.636	122.52(63.63 to 216.27)	31.47	91.05
Sweden	0.872	55(28.02 to 99.92)	31.4	23.6
Switzerland	0.929	35.84(18.35 to 65.01)	31.34	4.5
Syrian Arab Republic	0.619	93.73(48.28 to 169.47)	31.83	61.9
Taiwan (Province of China)	0.868	40.02(20.29 to 71.77)	31.41	8.61
Tajikistan	0.539	80.75(41.31 to 145.44)	33.4	47.35
Thailand	0.687	41.01(21.03 to 75.29)	31.29	9.72
Timor-Leste	0.514	40.71(20.8 to 74.91)	33.44	7.27
Togo	0.417	85.21(44.05 to 151.9)	40.3	44.91
Tokelau	0.626	40.68(20.68 to 74.66)	31.49	9.19
Tonga	0.636	40.82(20.78 to 74.4)	32.5	8.33
Trinidad and Tobago	0.757	122.68(63.84 to 217.85)	31.43	91.25
Tunisia	0.672	93.89(48.29 to 169.17)	31.39	62.5
Turkey	0.748	109.67(57.2 to 198.02)	31.28	78.38
Turkmenistan	0.67	80.93(41.25 to 146.86)	31.36	49.56
Tuvalu	0.589	40.65(20.79 to 73.97)	33.37	7.28
Uganda	0.404	84.93(44.11 to 151.64)	40.32	44.61
Ukraine	0.736	89.79(46.08 to 163.81)	31.28	58.51
United Arab Emirates	0.88	89.59(46.3 to 161.86)	31.29	58.3
United Kingdom	0.847	79.31(40.78 to 142.97)	31.24	48.07
United Republic of Tanzania	0.423	84.85(43.98 to 151.18)	40.32	44.53
United States of America	0.859	73.5(37.17 to 131.47)	31.33	42.17
United States Virgin Islands	0.799	123.2(63.97 to 218.49)	31.41	91.79
Uruguay	0.697	105.19(54.32 to 187.87)	31.42	73.77
Uzbekistan	0.631	80.83(41.4 to 145.85)	32.55	48.28
Vanuatu	0.485	40.59(20.78 to 73.86)	33.77	6.82
Venezuela (Bolivarian Republic of)	0.607	123.03(63.74 to 217.04)	32.55	90.48
Viet Nam	0.617	41.08(21.03 to 75.32)	32.47	8.61
Yemen	0.412	93.27(47.96 to 169.35)	40.35	52.92
Zambia	0.505	84.55(43.64 to 150.77)	33.46	51.09
Zimbabwe	0.476	84.63(43.91 to 150.81)	33.92	50.71

Effective difference rank (Age-standardized DALYs rank)
128 (148)
46 (41)
155 (158)
28 (9)
55 (50)
115 (111)
194 (191)
168 (168)
113 (86)
64 (59)
101 (76)
112 (84)
197 (196)
147 (145)
164 (162)
200 (200)
138 (135)
65 (60)
183 (192)
97 (123)
202 (202)
163 (163)
175 (184)
69 (64)
130 (92)
203 (203)
45 (40)
76 (72)
7 (129)
83 (95)
127 (127)
14 (31)
121 (110)
51 (46)
80 (94)
5 (107)
167 (167)
9 (1)
196 (199)
118 (124)
123 (102)
35 (23)
195 (197)
70 (65)
190 (183)
58 (54)
68 (63)
8 (3)
85 (98)
72 (67)
120 (115)
189 (177)
178 (193)
198 (195)
160 (155)
177 (189)

132 (100)
87 (104)
140 (133)
117 (90)
105 (139)
27 (10)
109 (79)
48 (43)
133 (106)
91 (112)
110 (81)
53 (48)
126 (126)
103 (77)
61 (56)
187 (179)
36 (27)
174 (178)
95 (121)
93 (119)
179 (172)
172 (173)
173 (185)
100 (75)
44 (39)
166 (165)
41 (35)
142 (137)
156 (152)
60 (52)
62 (57)
79 (74)
192 (190)
43 (38)
158 (151)
111 (80)
134 (138)
18 (24)
154 (149)
106 (88)
12 (29)
139 (134)
161 (160)
116 (89)
82 (93)
157 (150)
153 (147)
54 (49)
96 (116)
89 (108)
38 (26)
15 (13)
6 (120)
56 (51)
13 (7)
124 (128)
29 (17)
181 (181)

19 (16)
57 (53)
104 (85)
77 (71)
151 (157)
84 (97)
20 (32)
129 (96)
32 (25)
150 (164)
42 (37)
67 (62)
176 (186)
4 (125)
135 (140)
30 (11)
74 (70)
33 (18)
21 (4)
146 (144)
165 (166)
26 (6)
148 (153)
191 (187)
1 (8)
204 (204)
201 (201)
40 (36)
170 (170)
63 (58)
193 (188)
143 (141)
47 (42)
137 (132)
78 (73)
136 (131)
90 (109)
188 (182)
186 (175)
180 (176)
22 (19)
59 (55)
125 (122)
152 (146)
98 (117)
71 (69)
34 (21)
88 (113)
52 (47)
73 (68)
75 (66)
3 (14)
2 (103)
141 (136)
81 (91)
49 (44)
37 (30)
149 (156)

184 (174)
50 (45)
10 (2)
159 (159)
24 (5)
102 (82)
39 (33)
16 (22)
99 (130)
31 (20)
23 (28)
185 (180)
162 (161)
171 (171)
114 (87)
17 (15)
94 (118)
145 (143)
144 (142)
107 (78)
92 (114)
66 (61)
199 (198)
169 (169)
108 (83)
11 (12)
182 (194)
25 (34)
131 (154)
122 (99)
119 (105)
