# Supplementary material for: The global, regional, and national patterns of change in the burden of nonmalignant upper gastrointestinal diseases from 1990 to 2019 and the forecast for the next decade
Source: Int J Surg. 2024 Jul 3;111(1):80–92. doi: 10.1097/JS9.0000000000001902 (PMC11745775; doi:10.1097/JS9.0000000000001902)
Supplement: Supplementary file 10 [file js9-111-0080-s010.pdf]

Table S9. All age DALYs number of PUD, GD, and GERD from 1990 to 2019 and projected to 2030, categorized b

Location	Cause	Sex	Pred_val	Pred_low	Pred_up	Year
Global	Peptic ulcer disease	Male	5067073.227	5060835.45	5073311.004	1990
Global	Peptic ulcer disease	Male	5092300.709	5086049.278	5098552.14	1991
Global	Peptic ulcer disease	Male	5123744.168	5117473.636	5130014.701	1992
Global	Peptic ulcer disease	Male	5144489.127	5138205.914	5150772.339	1993
Global	Peptic ulcer disease	Male	5140994.144	5134713.103	5147275.186	1994
Global	Peptic ulcer disease	Male	5065914.86	5059679.931	5072149.788	1995
Global	Peptic ulcer disease	Male	4976586.141	4970406.502	4982765.779	1996
Global	Peptic ulcer disease	Male	4927492.775	4921343.739	4933641.81	1997
Global	Peptic ulcer disease	Male	4844110.55	4838013.83	4850207.27	1998
Global	Peptic ulcer disease	Male	4772420.425	4766369.034	4778471.816	1999
Global	Peptic ulcer disease	Male	4703545.035	4697537.525	4709552.545	2000
Global	Peptic ulcer disease	Male	4604629.615	4598685.701	4610573.529	2001
Global	Peptic ulcer disease	Male	4519022.162	4513133.839	4524910.486	2002
Global	Peptic ulcer disease	Male	4413409.777	4407590.767	4419228.788	2003
Global	Peptic ulcer disease	Male	4296798.355	4291056.846	4302539.863	2004
Global	Peptic ulcer disease	Male	4254088.542	4248375.685	4259801.399	2005
Global	Peptic ulcer disease	Male	4130290.572	4124661.574	4135919.569	2006
Global	Peptic ulcer disease	Male	4043607.93	4038038.402	4049177.458	2007
Global	Peptic ulcer disease	Male	4005817.927	4000274.525	4011361.329	2008
Global	Peptic ulcer disease	Male	3889143.629	3883681.681	3894605.576	2009
Global	Peptic ulcer disease	Male	3807271.555	3801867.496	3812675.614	2010
Global	Peptic ulcer disease	Male	3732758.178	3727407.351	3738109.005	2011
Global	Peptic ulcer disease	Male	3667752.596	3662448.647	3673056.545	2012
Global	Peptic ulcer disease	Male	3628910.056	3623634.316	3634185.796	2013
Global	Peptic ulcer disease	Male	3557283.103	3552059.757	3562506.448	2014
Global	Peptic ulcer disease	Male	3538163.679	3532954.385	3543372.973	2015
Global	Peptic ulcer disease	Male	3536183.274	3530975.413	3541391.136	2016
Global	Peptic ulcer disease	Male	3515917.728	3510724.834	3521110.622	2017
Global	Peptic ulcer disease	Male	3488177.657	3483005.109	3493350.205	2018
Global	Peptic ulcer disease	Male	3468018.496	3462858.689	3473178.304	2019
Global	Peptic ulcer disease	Male	3485272.599	3386629.78	3583915.418	2020
Global	Peptic ulcer disease	Male	3468533.384	3331678.071	3605388.697	2021
Global	Peptic ulcer disease	Male	3450320.144	3263787.295	3636852.992	2022
Global	Peptic ulcer disease	Male	3432217.62	3187612.819	3676822.421	2023
Global	Peptic ulcer disease	Male	3415104.479	3105688.79	3724520.168	2024
Global	Peptic ulcer disease	Male	3397798.176	3017956.247	3777640.105	2025
Global	Peptic ulcer disease	Male	3379530.447	2924606.68	3834454.214	2026
Global	Peptic ulcer disease	Male	3359839.893	2825902.32	3893777.465	2027
Global	Peptic ulcer disease	Male	3339867.798	2723297.468	3956438.128	2028
Global	Peptic ulcer disease	Male	3320272.172	2617635.783	4022908.561	2029
Global	Peptic ulcer disease	Male	3300128.606	2508436.251	4091820.962	2030
Global	Peptic ulcer disease	Female	3129068.117	3124166.364	3133969.871	1990
Global	Peptic ulcer disease	Female	3134524.081	3129619.38	3139428.783	1991
Global	Peptic ulcer disease	Female	3179244.936	3174305.507	3184184.364	1992
Global	Peptic ulcer disease	Female	3161643.197	3156717.486	3166568.908	1993
Global	Peptic ulcer disease	Female	3149181.692	3144265.727	3154097.658	1994
Global	Peptic ulcer disease	Female	3118948.234	3114055.967	3123840.502	1995
Global	Peptic ulcer disease	Female	3097489.923	3092614.541	3102365.305	1996
Global	Peptic ulcer disease	Female	3136396.677	3131490.736	3141302.618	1997
Global	Peptic ulcer disease	Female	3109988.154	3105102.935	3114873.372	1998
Global	Peptic ulcer disease	Female	3085643.283	3080777.24	3090509.327	1999
Global	Peptic ulcer disease	Female	3071601.401	3066746.449	3076456.354	2000
Global	Peptic ulcer disease	Female	3044225.235	3039391.997	3049058.472	2001
Global	Peptic ulcer disease	Female	3010733.117	3005926.586	3015539.648	2002
Global	Peptic ulcer disease	Female	2964005.854	2959236.82	2968774.887	2003
Global	Peptic ulcer disease	Female	2892257.173	2887546.291	2896968.054	2004
Global	Peptic ulcer disease	Female	2880965.246	2876263.579	2885666.913	2005
Global	Peptic ulcer disease	Female	2824373.15	2819717.95	2829028.35	2006
Global	Peptic ulcer disease	Female	2758847.841	2754247.032	2763448.65	2007
Global	Peptic ulcer disease	Female	2716880.164	2712314.531	2721445.796	2008

Global	Peptic ulcer disease	Female	2637455.347	2632957.039	2641953.655	2009
Global	Peptic ulcer disease	Female	2610621.445	2606146.108	2615096.782	2010
Global	Peptic ulcer disease	Female	2579449.519	2575001.018	2583898.019	2011
Global	Peptic ulcer disease	Female	2532623.94	2528216.062	2537031.817	2012
Global	Peptic ulcer disease	Female	2532621.66	2528213.781	2537029.539	2013
Global	Peptic ulcer disease	Female	2507111.311	2502725.703	2511496.92	2014
Global	Peptic ulcer disease	Female	2519707.654	2515311.002	2524104.305	2015
Global	Peptic ulcer disease	Female	2537015.618	2532603.861	2541427.374	2016
Global	Peptic ulcer disease	Female	2559823.009	2555391.434	2564254.584	2017
Global	Peptic ulcer disease	Female	2560021.324	2555589.388	2564453.259	2018
Global	Peptic ulcer disease	Female	2561491.694	2557057.02	2565926.369	2019
Global	Peptic ulcer disease	Female	2580890.9	2491375.146	2670406.654	2020
Global	Peptic ulcer disease	Female	2590433.772	2468201.334	2712666.211	2021
Global	Peptic ulcer disease	Female	2598394.26	2433341.178	2763447.341	2022
Global	Peptic ulcer disease	Female	2606063.23	2390351.286	2821775.175	2023
Global	Peptic ulcer disease	Female	2614530.418	2341515.808	2887545.028	2024
Global	Peptic ulcer disease	Female	2623198.626	2287028.898	2959368.355	2025
Global	Peptic ulcer disease	Female	2631267.353	2226820.933	3035713.773	2026
Global	Peptic ulcer disease	Female	2638074.963	2160803.084	3115346.841	2027
Global	Peptic ulcer disease	Female	2644611.29	2090122.369	3199100.212	2028
Global	Peptic ulcer disease	Female	2651801.216	2015665.717	3287936.715	2029
Global	Peptic ulcer disease	Female	2658999.755	1937031.547	3380967.963	2030
Global	Peptic ulcer disease	Both	8196141.344	8185001.813	8207280.875	1990
Global	Peptic ulcer disease	Both	8226824.79	8215668.658	8237980.922	1991
Global	Peptic ulcer disease	Both	8302989.104	8291779.143	8314199.064	1992
Global	Peptic ulcer disease	Both	8306132.324	8294923.4	8317341.247	1993
Global	Peptic ulcer disease	Both	8290175.837	8278978.83	8301372.844	1994
Global	Peptic ulcer disease	Both	8184863.094	8173735.898	8195990.29	1995
Global	Peptic ulcer disease	Both	8074076.064	8063021.043	8085131.084	1996
Global	Peptic ulcer disease	Both	8063889.452	8052834.476	8074944.428	1997
Global	Peptic ulcer disease	Both	7954098.704	7943116.765	7965080.642	1998
Global	Peptic ulcer disease	Both	7858063.708	7847146.274	7868981.143	1999
Global	Peptic ulcer disease	Both	7775146.436	7764283.974	7786008.898	2000
Global	Peptic ulcer disease	Both	7648854.85	7638077.699	7659632.001	2001
Global	Peptic ulcer disease	Both	7529755.28	7519060.425	7540450.134	2002
Global	Peptic ulcer disease	Both	7377415.631	7366827.587	7388003.675	2003
Global	Peptic ulcer disease	Both	7189055.527	7178603.138	7199507.917	2004
Global	Peptic ulcer disease	Both	7135053.788	7124639.263	7145468.313	2005
Global	Peptic ulcer disease	Both	6954663.721	6944379.524	6964947.919	2006
Global	Peptic ulcer disease	Both	6802455.772	6792285.435	6812626.109	2007
Global	Peptic ulcer disease	Both	6722698.09	6712589.056	6732807.125	2008
Global	Peptic ulcer disease	Both	6526598.976	6516638.72	6536559.232	2009
Global	Peptic ulcer disease	Both	6417893	6408013.604	6427772.396	2010
Global	Peptic ulcer disease	Both	6312207.697	6302408.369	6322007.024	2011
Global	Peptic ulcer disease	Both	6200376.536	6190664.709	6210088.363	2012
Global	Peptic ulcer disease	Both	6161531.716	6151848.097	6171215.335	2013
Global	Peptic ulcer disease	Both	6064394.414	6054785.46	6074003.368	2014
Global	Peptic ulcer disease	Both	6057871.333	6048265.387	6067477.278	2015
Global	Peptic ulcer disease	Both	6073198.892	6063579.274	6082818.51	2016
Global	Peptic ulcer disease	Both	6075740.737	6066116.268	6085365.207	2017
Global	Peptic ulcer disease	Both	6048198.98	6038594.497	6057803.464	2018
Global	Peptic ulcer disease	Both	6029510.191	6019915.708	6039104.673	2019
Global	Peptic ulcer disease	Both	6066163.499	5878004.926	6254322.072	2020
Global	Peptic ulcer disease	Both	6058967.157	5799879.405	6318054.908	2021
Global	Peptic ulcer disease	Both	6048714.403	5697128.474	6400300.333	2022
Global	Peptic ulcer disease	Both	6038280.85	5577964.105	6498597.596	2023
Global	Peptic ulcer disease	Both	6029634.897	5447204.597	6612065.196	2024
Global	Peptic ulcer disease	Both	6020996.802	5304985.144	6737008.459	2025
Global	Peptic ulcer disease	Both	6010797.8	5151427.613	6870167.987	2026
Global	Peptic ulcer disease	Both	5997914.856	4986705.404	7009124.307	2027
Global	Peptic ulcer disease	Both	5984479.088	4813419.836	7155538.34	2028
Global	Peptic ulcer disease	Both	5972073.388	4633301.5	7310845.275	2029

Global	Peptic ulcer disease	Both	5959128.362	4445467.798	7472788.925	2030
High SDI	Peptic ulcer disease	Male	428700.089	426890.2837	430509.8942	1990
High SDI	Peptic ulcer disease	Male	420277.0677	418492.4613	422061.6741	1991
High SDI	Peptic ulcer disease	Male	413170.0491	411401.138	414938.9603	1992
High SDI	Peptic ulcer disease	Male	409805.6286	408044.1275	411567.1297	1993
High SDI	Peptic ulcer disease	Male	401856.7186	400112.7211	403600.7161	1994
High SDI	Peptic ulcer disease	Male	394554.4246	392826.5813	396282.2678	1995
High SDI	Peptic ulcer disease	Male	380041.263	378345.957	381736.569	1996
High SDI	Peptic ulcer disease	Male	365531.6824	363869.5224	367193.8424	1997
High SDI	Peptic ulcer disease	Male	354144.1508	352508.4214	355779.8802	1998
High SDI	Peptic ulcer disease	Male	341302.5856	339697.1801	342907.9911	1999
High SDI	Peptic ulcer disease	Male	325913.7769	324345.5495	327482.0042	2000
High SDI	Peptic ulcer disease	Male	313816.9116	312278.5443	315355.2789	2001
High SDI	Peptic ulcer disease	Male	305616.0005	304098.1935	307133.8076	2002
High SDI	Peptic ulcer disease	Male	298051.5975	296552.9839	299550.2111	2003
High SDI	Peptic ulcer disease	Male	286927.3445	285457.4369	288397.252	2004
High SDI	Peptic ulcer disease	Male	281200.4326	279745.4881	282655.3771	2005
High SDI	Peptic ulcer disease	Male	273227.7378	271793.9294	274661.5463	2006
High SDI	Peptic ulcer disease	Male	266656.3219	265240.1793	268072.4646	2007
High SDI	Peptic ulcer disease	Male	261007.713	259606.9602	262408.4658	2008
High SDI	Peptic ulcer disease	Male	255297.2088	253912.1671	256682.2505	2009
High SDI	Peptic ulcer disease	Male	250036.8835	248666.5043	251407.2627	2010
High SDI	Peptic ulcer disease	Male	246214.7479	244855.1027	247574.3931	2011
High SDI	Peptic ulcer disease	Male	242270.3964	240921.8832	243618.9096	2012
High SDI	Peptic ulcer disease	Male	239702.0103	238360.7711	241043.2495	2013
High SDI	Peptic ulcer disease	Male	236686.8112	235354.1545	238019.4679	2014
High SDI	Peptic ulcer disease	Male	237855.4204	236519.3238	239191.5169	2015
High SDI	Peptic ulcer disease	Male	238981.4973	237642.0101	240320.9846	2016
High SDI	Peptic ulcer disease	Male	239287.9797	237947.4836	240628.4758	2017
High SDI	Peptic ulcer disease	Male	242065.4938	240716.5553	243414.4323	2018
High SDI	Peptic ulcer disease	Male	243363.8544	242002.6605	244725.0483	2019
High SDI	Peptic ulcer disease	Male	237385.048	230040.814	244729.2819	2020
High SDI	Peptic ulcer disease	Male	239085.62	228124.052	250047.1881	2021
High SDI	Peptic ulcer disease	Male	240546.0839	224882.6637	256209.5041	2022
High SDI	Peptic ulcer disease	Male	242025.21	220816.2774	263234.1425	2023
High SDI	Peptic ulcer disease	Male	243666.4236	216170.1511	271162.696	2024
High SDI	Peptic ulcer disease	Male	245346.3103	210888.1477	279804.4729	2025
High SDI	Peptic ulcer disease	Male	246894.6088	204882.5836	288906.634	2026
High SDI	Peptic ulcer disease	Male	248225.1095	198132.7357	298317.4833	2027
High SDI	Peptic ulcer disease	Male	249526.5785	190826.9061	308226.2508	2028
High SDI	Peptic ulcer disease	Male	250918.7448	183067.5458	318769.9438	2029
High SDI	Peptic ulcer disease	Male	252311.502	174789.3732	329833.6307	2030
High SDI	Peptic ulcer disease	Female	314106.4424	312558.6536	315654.2313	1990
High SDI	Peptic ulcer disease	Female	310069.3802	308539.3303	311599.4302	1991
High SDI	Peptic ulcer disease	Female	305931.0581	304411.8727	307450.2436	1992
High SDI	Peptic ulcer disease	Female	305869.0955	304350.152	307388.0391	1993
High SDI	Peptic ulcer disease	Female	300238.5463	298733.9925	301743.1002	1994
High SDI	Peptic ulcer disease	Female	297000.8999	295504.6397	298497.1601	1995
High SDI	Peptic ulcer disease	Female	288904.5023	287429.1278	290379.8768	1996
High SDI	Peptic ulcer disease	Female	280217.1598	278764.5277	281669.792	1997
High SDI	Peptic ulcer disease	Female	271049.8036	269621.5327	272478.0745	1998
High SDI	Peptic ulcer disease	Female	261397.3709	259995.1924	262799.5493	1999
High SDI	Peptic ulcer disease	Female	249397.0617	248028.0473	250766.076	2000
High SDI	Peptic ulcer disease	Female	240204.0912	238861.0038	241547.1787	2001
High SDI	Peptic ulcer disease	Female	233411.3927	232087.7971	234734.9884	2002
High SDI	Peptic ulcer disease	Female	227353.6044	226047.6342	228659.5746	2003
High SDI	Peptic ulcer disease	Female	218307.9005	217028.7514	219587.0497	2004
High SDI	Peptic ulcer disease	Female	212436.7175	211175.243	213698.1921	2005
High SDI	Peptic ulcer disease	Female	205905.6944	204664.1871	207147.2016	2006
High SDI	Peptic ulcer disease	Female	200685.6747	199460.3662	201910.9833	2007
High SDI	Peptic ulcer disease	Female	196513.2626	195301.0646	197725.4606	2008
High SDI	Peptic ulcer disease	Female	191375.6627	190179.7798	192571.5456	2009

High SDI	Peptic ulcer disease	Female	187214.0393	186031.5499	188396.5286	2010
High SDI	Peptic ulcer disease	Female	184956.9281	183781.7765	186132.0798	2011
High SDI	Peptic ulcer disease	Female	182347.3728	181180.7384	183514.0071	2012
High SDI	Peptic ulcer disease	Female	180220.4699	179060.7893	181380.1506	2013
High SDI	Peptic ulcer disease	Female	177419.4268	176268.9307	178569.9228	2014
High SDI	Peptic ulcer disease	Female	178336.9348	177183.2699	179490.5998	2015
High SDI	Peptic ulcer disease	Female	180112.4869	178952.7871	181272.1867	2016
High SDI	Peptic ulcer disease	Female	181444.7887	180280.6018	182608.9757	2017
High SDI	Peptic ulcer disease	Female	185006.9096	183830.3381	186183.4812	2018
High SDI	Peptic ulcer disease	Female	187937.0359	186742.0968	189131.9751	2019
High SDI	Peptic ulcer disease	Female	183040.8342	177255.886	188825.7825	2020
High SDI	Peptic ulcer disease	Female	185360.4644	176868.381	193852.5478	2021
High SDI	Peptic ulcer disease	Female	187526.9685	175479.9348	199574.0021	2022
High SDI	Peptic ulcer disease	Female	189827.8973	173538.4322	206117.3624	2023
High SDI	Peptic ulcer disease	Female	192406.355	171242.1769	213570.5331	2024
High SDI	Peptic ulcer disease	Female	195142.4175	168504.7154	221780.1195	2025
High SDI	Peptic ulcer disease	Female	197924.6045	165259.8905	230589.3185	2026
High SDI	Peptic ulcer disease	Female	200673.6085	161461.7972	239885.4197	2027
High SDI	Peptic ulcer disease	Female	203564.0488	157256.7035	249871.3941	2028
High SDI	Peptic ulcer disease	Female	206687.5364	152696.4047	260678.6682	2029
High SDI	Peptic ulcer disease	Female	209959.4388	147691.6888	272227.1889	2030
High SDI	Peptic ulcer disease	Both	742806.5314	739448.9373	746164.1255	1990
High SDI	Peptic ulcer disease	Both	730346.4479	727031.7916	733661.1043	1991
High SDI	Peptic ulcer disease	Both	719101.1073	715813.0108	722389.2038	1992
High SDI	Peptic ulcer disease	Both	715674.7242	712394.2796	718955.1688	1993
High SDI	Peptic ulcer disease	Both	702095.2649	698846.7136	705343.8162	1994
High SDI	Peptic ulcer disease	Both	691555.3245	688331.221	694779.4279	1995
High SDI	Peptic ulcer disease	Both	668945.7653	665775.0848	672116.4458	1996
High SDI	Peptic ulcer disease	Both	645748.8422	642634.0501	648863.6343	1997
High SDI	Peptic ulcer disease	Both	625193.9543	622129.954	628257.9547	1998
High SDI	Peptic ulcer disease	Both	602699.9565	599692.3725	605707.5404	1999
High SDI	Peptic ulcer disease	Both	575310.8385	572373.5968	578248.0803	2000
High SDI	Peptic ulcer disease	Both	554021.0028	551139.5481	556902.4576	2001
High SDI	Peptic ulcer disease	Both	539027.3933	536185.9906	541868.7959	2002
High SDI	Peptic ulcer disease	Both	525405.2019	522600.6181	528209.7857	2003
High SDI	Peptic ulcer disease	Both	505235.245	502486.1883	507984.3017	2004
High SDI	Peptic ulcer disease	Both	493637.1501	490920.7311	496353.5691	2005
High SDI	Peptic ulcer disease	Both	479133.4322	476458.1165	481808.7479	2006
High SDI	Peptic ulcer disease	Both	467341.9967	464700.5455	469983.4478	2007
High SDI	Peptic ulcer disease	Both	457520.9756	454908.0248	460133.9264	2008
High SDI	Peptic ulcer disease	Both	446672.8715	444091.9469	449253.7961	2009
High SDI	Peptic ulcer disease	Both	437250.9228	434698.0542	439803.7913	2010
High SDI	Peptic ulcer disease	Both	431171.6761	428636.8792	433706.4729	2011
High SDI	Peptic ulcer disease	Both	424617.7691	422102.6216	427132.9166	2012
High SDI	Peptic ulcer disease	Both	419922.4802	417421.5603	422423.4	2013
High SDI	Peptic ulcer disease	Both	414106.2379	411623.0852	416589.3907	2014
High SDI	Peptic ulcer disease	Both	416192.3552	413702.5937	418682.1167	2015
High SDI	Peptic ulcer disease	Both	419093.9843	416594.7972	421593.1714	2016
High SDI	Peptic ulcer disease	Both	420732.7684	418228.0854	423237.4515	2017
High SDI	Peptic ulcer disease	Both	427072.4034	424546.8933	429597.9135	2018
High SDI	Peptic ulcer disease	Both	431300.8903	428744.7573	433857.0234	2019
High SDI	Peptic ulcer disease	Both	420425.8822	407296.7	433555.0644	2020
High SDI	Peptic ulcer disease	Both	424446.0844	404992.433	443899.7359	2021
High SDI	Peptic ulcer disease	Both	428073.0524	400362.5986	455783.5062	2022
High SDI	Peptic ulcer disease	Both	431853.1073	394354.7096	469351.505	2023
High SDI	Peptic ulcer disease	Both	436072.7786	387412.3281	484733.2291	2024
High SDI	Peptic ulcer disease	Both	440488.7278	379392.8631	501584.5924	2025
High SDI	Peptic ulcer disease	Both	444819.2133	370142.4741	519495.9525	2026
High SDI	Peptic ulcer disease	Both	448898.718	359594.5329	538202.903	2027
High SDI	Peptic ulcer disease	Both	453090.6272	348083.6096	558097.6449	2028
High SDI	Peptic ulcer disease	Both	457606.2812	335763.9505	579448.6119	2029
High SDI	Peptic ulcer disease	Both	462270.9408	322481.062	602060.8196	2030

High-middle SDI	Peptic ulcer disease	Male	834237.6821	831707.249	836768.1153	1990
High-middle SDI	Peptic ulcer disease	Male	839480.4735	836944.5945	842016.3524	1991
High-middle SDI	Peptic ulcer disease	Male	869316.2517	866735.6552	871896.8483	1992
High-middle SDI	Peptic ulcer disease	Male	911375.1208	908732.7357	914017.506	1993
High-middle SDI	Peptic ulcer disease	Male	933248.0068	930574.0113	935922.0023	1994
High-middle SDI	Peptic ulcer disease	Male	914303.9498	911657.325	916950.5745	1995
High-middle SDI	Peptic ulcer disease	Male	866387.3997	863811.2869	868963.5125	1996
High-middle SDI	Peptic ulcer disease	Male	821661.3222	819152.7768	824169.8676	1997
High-middle SDI	Peptic ulcer disease	Male	793824.0597	791358.5121	796289.6074	1998
High-middle SDI	Peptic ulcer disease	Male	799369.2693	796895.1074	801843.4312	1999
High-middle SDI	Peptic ulcer disease	Male	796806.5883	794336.3947	799276.782	2000
High-middle SDI	Peptic ulcer disease	Male	780270.2251	777825.8839	782714.5663	2001
High-middle SDI	Peptic ulcer disease	Male	771691.0852	769260.2556	774121.9147	2002
High-middle SDI	Peptic ulcer disease	Male	767076.7442	764653.217	769500.2714	2003
High-middle SDI	Peptic ulcer disease	Male	755105.6538	752701.1696	757510.1379	2004
High-middle SDI	Peptic ulcer disease	Male	756826.553	754419.2873	759233.8188	2005
High-middle SDI	Peptic ulcer disease	Male	708985.4454	706655.7766	711315.1141	2006
High-middle SDI	Peptic ulcer disease	Male	682947.9567	680661.6086	685234.3048	2007
High-middle SDI	Peptic ulcer disease	Male	675090.4405	672817.3337	677363.5473	2008
High-middle SDI	Peptic ulcer disease	Male	648252.9698	646025.6879	650480.2517	2009
High-middle SDI	Peptic ulcer disease	Male	641760.2358	639544.1704	643976.3012	2010
High-middle SDI	Peptic ulcer disease	Male	619590.318	617413.0065	621767.6296	2011
High-middle SDI	Peptic ulcer disease	Male	609660.6266	607500.886	611820.3672	2012
High-middle SDI	Peptic ulcer disease	Male	599715.1032	597573.1225	601857.0839	2013
High-middle SDI	Peptic ulcer disease	Male	597198.6551	595061.1918	599336.1184	2014
High-middle SDI	Peptic ulcer disease	Male	605247.7517	603095.8547	607399.6487	2015
High-middle SDI	Peptic ulcer disease	Male	600304.6523	598161.5502	602447.7544	2016
High-middle SDI	Peptic ulcer disease	Male	580380.9255	578273.7654	582488.0857	2017
High-middle SDI	Peptic ulcer disease	Male	567955.5701	565871.1371	570040.003	2018
High-middle SDI	Peptic ulcer disease	Male	563776.91	561697.2972	565856.5228	2019
High-middle SDI	Peptic ulcer disease	Male	284036.9511	269871.8578	298202.0444	2020
High-middle SDI	Peptic ulcer disease	Male	280825.5188	258041.9096	303609.128	2021
High-middle SDI	Peptic ulcer disease	Male	277535.349	243938.6105	311132.0875	2022
High-middle SDI	Peptic ulcer disease	Male	274503.0737	228558.8185	320447.3288	2023
High-middle SDI	Peptic ulcer disease	Male	271731.7694	212194.1621	331269.3766	2024
High-middle SDI	Peptic ulcer disease	Male	268867.2482	194726.9192	343007.5772	2025
High-middle SDI	Peptic ulcer disease	Male	265646.9397	176181.3919	355112.4876	2026
High-middle SDI	Peptic ulcer disease	Male	262327.0501	156923.2064	367730.8938	2027
High-middle SDI	Peptic ulcer disease	Male	259227.9519	137252.49	381203.4138	2028
High-middle SDI	Peptic ulcer disease	Male	256340.6695	117190.8391	395490.4999	2029
High-middle SDI	Peptic ulcer disease	Male	253335.2258	96615.41773	410055.0339	2030
High-middle SDI	Peptic ulcer disease	Female	370208.5685	368525.1231	371892.0139	1990
High-middle SDI	Peptic ulcer disease	Female	369013.076	367336.1627	370689.9893	1991
High-middle SDI	Peptic ulcer disease	Female	371556.8754	369874.4304	373239.3204	1992
High-middle SDI	Peptic ulcer disease	Female	380360.2742	378657.8963	382062.6521	1993
High-middle SDI	Peptic ulcer disease	Female	385038.9591	383326.0961	386751.822	1994
High-middle SDI	Peptic ulcer disease	Female	377301.6207	375606.236	378997.0054	1995
High-middle SDI	Peptic ulcer disease	Female	364495.5104	362829.4341	366161.5867	1996
High-middle SDI	Peptic ulcer disease	Female	354713.7815	353070.3831	356357.1798	1997
High-middle SDI	Peptic ulcer disease	Female	346657.1285	345032.6255	348281.6315	1998
High-middle SDI	Peptic ulcer disease	Female	347583.7665	345957.0928	349210.4401	1999
High-middle SDI	Peptic ulcer disease	Female	348148.8621	346520.8642	349776.8601	2000
High-middle SDI	Peptic ulcer disease	Female	347399.1997	345772.971	349025.4283	2001
High-middle SDI	Peptic ulcer disease	Female	349248.9331	347618.3472	350879.519	2002
High-middle SDI	Peptic ulcer disease	Female	350909.8266	349275.2902	352544.363	2003
High-middle SDI	Peptic ulcer disease	Female	347808.8575	346181.606	349436.109	2004
High-middle SDI	Peptic ulcer disease	Female	348759.6017	347130.0747	350389.1287	2005
High-middle SDI	Peptic ulcer disease	Female	335844.1263	334245.332	337442.9206	2006
High-middle SDI	Peptic ulcer disease	Female	329384.726	327801.5044	330967.9476	2007
High-middle SDI	Peptic ulcer disease	Female	326037.2561	324462.1612	327612.3509	2008
High-middle SDI	Peptic ulcer disease	Female	320177.9413	318617.1996	321738.6829	2009
High-middle SDI	Peptic ulcer disease	Female	319110.4578	317552.3249	320668.5907	2010

High-middle SDI	Peptic ulcer disease	Female	315090.7227	313542.5181	316638.9273	2011
High-middle SDI	Peptic ulcer disease	Female	312781.5549	311239.0695	314324.0404	2012
High-middle SDI	Peptic ulcer disease	Female	312279.3728	310738.1196	313820.6261	2013
High-middle SDI	Peptic ulcer disease	Female	313200.4754	311656.8874	314744.0633	2014
High-middle SDI	Peptic ulcer disease	Female	318622.1154	317065.0375	320179.1933	2015
High-middle SDI	Peptic ulcer disease	Female	319139.6699	317581.2348	320698.1049	2016
High-middle SDI	Peptic ulcer disease	Female	316097.3808	314546.4054	317648.3562	2017
High-middle SDI	Peptic ulcer disease	Female	313282.4833	311738.164	314826.8025	2018
High-middle SDI	Peptic ulcer disease	Female	312212.7332	310667.1555	313758.3109	2019
High-middle SDI	Peptic ulcer disease	Female	178304.0036	170806.2515	185801.7557	2020
High-middle SDI	Peptic ulcer disease	Female	177574.5365	166890.617	188258.4561	2021
High-middle SDI	Peptic ulcer disease	Female	176675.1749	161851.3674	191498.9823	2022
High-middle SDI	Peptic ulcer disease	Female	175854.908	156190.6836	195519.1324	2023
High-middle SDI	Peptic ulcer disease	Female	175254.7762	150162.2235	200347.329	2024
High-middle SDI	Peptic ulcer disease	Female	174711.9347	143690.7941	205733.0752	2025
High-middle SDI	Peptic ulcer disease	Female	174076.5847	136721.4563	211431.7131	2026
High-middle SDI	Peptic ulcer disease	Female	173340.6581	129306.2462	217375.07	2027
High-middle SDI	Peptic ulcer disease	Female	172690.4922	121621.372	223759.6125	2028
High-middle SDI	Peptic ulcer disease	Female	172227.6477	113744.0524	230711.2431	2029
High-middle SDI	Peptic ulcer disease	Female	171801.7279	105572.8124	238030.6434	2030
High-middle SDI	Peptic ulcer disease	Both	1204446.251	1200232.372	1208660.129	1990
High-middle SDI	Peptic ulcer disease	Both	1208493.549	1204280.757	1212706.342	1991
High-middle SDI	Peptic ulcer disease	Both	1240873.127	1236610.086	1245136.169	1992
High-middle SDI	Peptic ulcer disease	Both	1291735.395	1287390.632	1296080.158	1993
High-middle SDI	Peptic ulcer disease	Both	1318286.966	1313900.107	1322673.824	1994
High-middle SDI	Peptic ulcer disease	Both	1291605.571	1287263.561	1295947.58	1995
High-middle SDI	Peptic ulcer disease	Both	1230882.91	1226640.721	1235125.099	1996
High-middle SDI	Peptic ulcer disease	Both	1176375.104	1172223.16	1180527.047	1997
High-middle SDI	Peptic ulcer disease	Both	1140481.188	1136391.138	1144571.239	1998
High-middle SDI	Peptic ulcer disease	Both	1146953.036	1142852.2	1151053.871	1999
High-middle SDI	Peptic ulcer disease	Both	1144955.45	1140857.259	1149053.642	2000
High-middle SDI	Peptic ulcer disease	Both	1127669.425	1123598.855	1131739.995	2001
High-middle SDI	Peptic ulcer disease	Both	1120940.018	1116878.603	1125001.434	2002
High-middle SDI	Peptic ulcer disease	Both	1117986.571	1113928.507	1122044.634	2003
High-middle SDI	Peptic ulcer disease	Both	1102914.511	1098882.776	1106946.247	2004
High-middle SDI	Peptic ulcer disease	Both	1105586.155	1101549.362	1109622.947	2005
High-middle SDI	Peptic ulcer disease	Both	1044829.572	1040901.109	1048758.035	2006
High-middle SDI	Peptic ulcer disease	Both	1012332.683	1008463.113	1016202.252	2007
High-middle SDI	Peptic ulcer disease	Both	1001127.697	997279.4949	1004975.898	2008
High-middle SDI	Peptic ulcer disease	Both	968430.911	964642.8875	972218.9345	2009
High-middle SDI	Peptic ulcer disease	Both	960870.6936	957096.4953	964644.8919	2010
High-middle SDI	Peptic ulcer disease	Both	934681.0407	930955.5246	938406.5569	2011
High-middle SDI	Peptic ulcer disease	Both	922442.1816	918739.9555	926144.4076	2012
High-middle SDI	Peptic ulcer disease	Both	911994.476	908311.2421	915677.71	2013
High-middle SDI	Peptic ulcer disease	Both	910399.1305	906718.0792	914080.1817	2014
High-middle SDI	Peptic ulcer disease	Both	923869.8671	920160.8922	927578.8421	2015
High-middle SDI	Peptic ulcer disease	Both	919444.3222	915742.785	923145.8593	2016
High-middle SDI	Peptic ulcer disease	Both	896478.3063	892820.1708	900136.4419	2017
High-middle SDI	Peptic ulcer disease	Both	881238.0533	877609.3011	884866.8055	2018
High-middle SDI	Peptic ulcer disease	Both	875989.6432	872364.4527	879614.8337	2019
High-middle SDI	Peptic ulcer disease	Both	462340.9547	440678.1092	484003.8001	2020
High-middle SDI	Peptic ulcer disease	Both	458400.0553	424932.5266	491867.5841	2021
High-middle SDI	Peptic ulcer disease	Both	454210.5239	405789.9779	502631.0698	2022
High-middle SDI	Peptic ulcer disease	Both	450357.9817	384749.5022	515966.4612	2023
High-middle SDI	Peptic ulcer disease	Both	446986.5456	362356.3856	531616.7056	2024
High-middle SDI	Peptic ulcer disease	Both	443579.1828	338417.7133	548740.6524	2025
High-middle SDI	Peptic ulcer disease	Both	439723.5244	312902.8481	566544.2007	2026
High-middle SDI	Peptic ulcer disease	Both	435667.7082	286229.4526	585105.9638	2027
High-middle SDI	Peptic ulcer disease	Both	431918.4441	258873.8619	604963.0263	2028
High-middle SDI	Peptic ulcer disease	Both	428568.3172	230934.8914	626201.743	2029
High-middle SDI	Peptic ulcer disease	Both	425136.9537	202188.2301	648085.6773	2030
Middle SDI	Peptic ulcer disease	Male	1219629.799	1216570.941	1222688.658	1990

Middle SDI	Peptic ulcer disease	Male	1221411.995	1218352.719	1224471.271	1991
Middle SDI	Peptic ulcer disease	Male	1219328.136	1216271.83	1222384.443	1992
Middle SDI	Peptic ulcer disease	Male	1210208.137	1207163.362	1213252.912	1993
Middle SDI	Peptic ulcer disease	Male	1201033.269	1198000.12	1204066.418	1994
Middle SDI	Peptic ulcer disease	Male	1186921.655	1183906.459	1189936.852	1995
Middle SDI	Peptic ulcer disease	Male	1171965.655	1168969.592	1174961.717	1996
Middle SDI	Peptic ulcer disease	Male	1145770.966	1142808.692	1148733.239	1997
Middle SDI	Peptic ulcer disease	Male	1121179.923	1118249.718	1124110.127	1998
Middle SDI	Peptic ulcer disease	Male	1105064.998	1102155.999	1107973.996	1999
Middle SDI	Peptic ulcer disease	Male	1096521.872	1093624.18	1099419.565	2000
Middle SDI	Peptic ulcer disease	Male	1082551.114	1079672.002	1085430.225	2001
Middle SDI	Peptic ulcer disease	Male	1079973.524	1077097.846	1082849.203	2002
Middle SDI	Peptic ulcer disease	Male	1073562.711	1070695.599	1076429.822	2003
Middle SDI	Peptic ulcer disease	Male	1066080.149	1063223.074	1068937.224	2004
Middle SDI	Peptic ulcer disease	Male	1054506.122	1051664.66	1057347.584	2005
Middle SDI	Peptic ulcer disease	Male	1027100.875	1024296.706	1029905.043	2006
Middle SDI	Peptic ulcer disease	Male	1005390.469	1002616.198	1008164.74	2007
Middle SDI	Peptic ulcer disease	Male	1001396.77	998628.0402	1004165.499	2008
Middle SDI	Peptic ulcer disease	Male	986118.7013	983371.2557	988866.1469	2009
Middle SDI	Peptic ulcer disease	Male	972277.8571	969549.8281	975005.8861	2010
Middle SDI	Peptic ulcer disease	Male	960239.9744	957528.9472	962951.0017	2011
Middle SDI	Peptic ulcer disease	Male	943241.0173	940554.1795	945927.8551	2012
Middle SDI	Peptic ulcer disease	Male	929726.4789	927059.0228	932393.935	2013
Middle SDI	Peptic ulcer disease	Male	911052.4021	908411.9492	913692.855	2014
Middle SDI	Peptic ulcer disease	Male	898293.0679	895671.2124	900914.9234	2015
Middle SDI	Peptic ulcer disease	Male	897002.8026	894382.828	899622.7773	2016
Middle SDI	Peptic ulcer disease	Male	887463.8844	884857.9014	890069.8674	2017
Middle SDI	Peptic ulcer disease	Male	875381.7246	872793.2138	877970.2354	2018
Middle SDI	Peptic ulcer disease	Male	869789.9109	867207.5831	872372.2388	2019
Middle SDI	Peptic ulcer disease	Male	1153275.443	1111623.562	1194927.324	2020
Middle SDI	Peptic ulcer disease	Male	1146061.473	1093092.752	1199030.195	2021
Middle SDI	Peptic ulcer disease	Male	1137672.372	1069809.927	1205534.816	2022
Middle SDI	Peptic ulcer disease	Male	1129120.423	1043649.332	1214591.513	2023
Middle SDI	Peptic ulcer disease	Male	1121084.84	1015820.816	1226348.863	2024
Middle SDI	Peptic ulcer disease	Male	1112884.409	986082.1907	1239686.628	2025
Middle SDI	Peptic ulcer disease	Male	1103963.382	954303.8521	1253622.912	2026
Middle SDI	Peptic ulcer disease	Male	1093987.887	920480.8463	1267494.927	2027
Middle SDI	Peptic ulcer disease	Male	1083739.686	885478.1675	1282001.205	2028
Middle SDI	Peptic ulcer disease	Male	1073756.368	849842.847	1297669.89	2029
Middle SDI	Peptic ulcer disease	Male	1063481.493	813217.6842	1313745.301	2030
Middle SDI	Peptic ulcer disease	Female	716517.3708	714173.1627	718861.579	1990
Middle SDI	Peptic ulcer disease	Female	711854.2066	709519.3298	714189.0835	1991
Middle SDI	Peptic ulcer disease	Female	718780.6225	716434.6876	721126.5573	1992
Middle SDI	Peptic ulcer disease	Female	706799.0534	704472.8526	709125.2543	1993
Middle SDI	Peptic ulcer disease	Female	703000.1995	700680.307	705320.0919	1994
Middle SDI	Peptic ulcer disease	Female	692221.7188	689919.7676	694523.67	1995
Middle SDI	Peptic ulcer disease	Female	676565.4959	674289.8378	678841.154	1996
Middle SDI	Peptic ulcer disease	Female	671085.9015	668819.5194	673352.2835	1997
Middle SDI	Peptic ulcer disease	Female	664206.8797	661952.1875	666461.5718	1998
Middle SDI	Peptic ulcer disease	Female	659995.223	657747.721	662242.725	1999
Middle SDI	Peptic ulcer disease	Female	659223.6518	656977.468	661469.8356	2000
Middle SDI	Peptic ulcer disease	Female	653582.6359	651346.1183	655819.1534	2001
Middle SDI	Peptic ulcer disease	Female	653008.371	650772.84	655243.9021	2002
Middle SDI	Peptic ulcer disease	Female	648756.4475	646528.2299	650984.6652	2003
Middle SDI	Peptic ulcer disease	Female	641784.6367	639568.4656	644000.8078	2004
Middle SDI	Peptic ulcer disease	Female	632511.5769	630311.5445	634711.6094	2005
Middle SDI	Peptic ulcer disease	Female	616982.4761	614809.7324	619155.2198	2006
Middle SDI	Peptic ulcer disease	Female	601828.9787	599683.1951	603974.7624	2007
Middle SDI	Peptic ulcer disease	Female	593468.8305	591338.067	595599.5941	2008
Middle SDI	Peptic ulcer disease	Female	583438.4971	581325.894	585551.1001	2009
Middle SDI	Peptic ulcer disease	Female	577655.9134	575553.846	579757.9808	2010
Middle SDI	Peptic ulcer disease	Female	570836.1332	568746.5655	572925.701	2011

Middle SDI	Peptic ulcer disease	Female	562041.1039	559967.7652	564114.4427	2012
Middle SDI	Peptic ulcer disease	Female	560537.9271	558467.3652	562608.489	2013
Middle SDI	Peptic ulcer disease	Female	551742.2489	549688.0384	553796.4595	2014
Middle SDI	Peptic ulcer disease	Female	545670.7198	543627.8554	547713.5841	2015
Middle SDI	Peptic ulcer disease	Female	548434.136	546386.0739	550482.198	2016
Middle SDI	Peptic ulcer disease	Female	552089.4992	550034.5752	554144.4232	2017
Middle SDI	Peptic ulcer disease	Female	553431.3138	551373.5464	555489.0812	2018
Middle SDI	Peptic ulcer disease	Female	556176.1927	554111.4187	558240.9667	2019
Middle SDI	Peptic ulcer disease	Female	744869.4507	712664.7918	777074.1095	2020
Middle SDI	Peptic ulcer disease	Female	750218.643	708393.479	792043.807	2021
Middle SDI	Peptic ulcer disease	Female	754876.5333	700257.3295	809495.7372	2022
Middle SDI	Peptic ulcer disease	Female	759534.6099	689536.8291	829532.3908	2023
Middle SDI	Peptic ulcer disease	Female	764738.8408	677111.9288	852365.7528	2024
Middle SDI	Peptic ulcer disease	Female	769982.1975	662761.0808	877203.3142	2025
Middle SDI	Peptic ulcer disease	Female	774869.5617	646374.5833	903364.5401	2026
Middle SDI	Peptic ulcer disease	Female	779183.2984	627939.3445	930427.2523	2027
Middle SDI	Peptic ulcer disease	Female	783463.5855	607996.3423	958930.8286	2028
Middle SDI	Peptic ulcer disease	Female	788149.2365	586906.7947	989391.6783	2029
Middle SDI	Peptic ulcer disease	Female	792740.9542	564301.4045	1021180.504	2030
Middle SDI	Peptic ulcer disease	Both	1936147.17	1930744.103	1941550.237	1990
Middle SDI	Peptic ulcer disease	Both	1933266.201	1927872.048	1938660.354	1991
Middle SDI	Peptic ulcer disease	Both	1938108.759	1932706.517	1943511	1992
Middle SDI	Peptic ulcer disease	Both	1917007.19	1911636.214	1922378.166	1993
Middle SDI	Peptic ulcer disease	Both	1904033.469	1898680.427	1909386.51	1994
Middle SDI	Peptic ulcer disease	Both	1879143.374	1873826.227	1884460.522	1995
Middle SDI	Peptic ulcer disease	Both	1848531.151	1843259.43	1853802.871	1996
Middle SDI	Peptic ulcer disease	Both	1816856.867	1811628.212	1822085.523	1997
Middle SDI	Peptic ulcer disease	Both	1785386.802	1780201.906	1790571.699	1998
Middle SDI	Peptic ulcer disease	Both	1765060.221	1759903.72	1770216.721	1999
Middle SDI	Peptic ulcer disease	Both	1755745.524	1750601.648	1760889.4	2000
Middle SDI	Peptic ulcer disease	Both	1736133.75	1731018.12	1741249.379	2001
Middle SDI	Peptic ulcer disease	Both	1732981.895	1727870.686	1738093.105	2002
Middle SDI	Peptic ulcer disease	Both	1722319.158	1717223.829	1727414.487	2003
Middle SDI	Peptic ulcer disease	Both	1707864.786	1702791.54	1712938.032	2004
Middle SDI	Peptic ulcer disease	Both	1687017.699	1681976.204	1692059.194	2005
Middle SDI	Peptic ulcer disease	Both	1644083.351	1639106.438	1649060.263	2006
Middle SDI	Peptic ulcer disease	Both	1607219.447	1602299.393	1612139.502	2007
Middle SDI	Peptic ulcer disease	Both	1594865.6	1589966.107	1599765.093	2008
Middle SDI	Peptic ulcer disease	Both	1569557.198	1564697.15	1574417.247	2009
Middle SDI	Peptic ulcer disease	Both	1549933.77	1545103.674	1554763.867	2010
Middle SDI	Peptic ulcer disease	Both	1531076.108	1526275.513	1535876.703	2011
Middle SDI	Peptic ulcer disease	Both	1505282.121	1500521.945	1510042.298	2012
Middle SDI	Peptic ulcer disease	Both	1490264.406	1485526.388	1495002.424	2013
Middle SDI	Peptic ulcer disease	Both	1462794.651	1458099.988	1467489.315	2014
Middle SDI	Peptic ulcer disease	Both	1443963.788	1439299.068	1448628.508	2015
Middle SDI	Peptic ulcer disease	Both	1445436.939	1440768.902	1450104.975	2016
Middle SDI	Peptic ulcer disease	Both	1439553.384	1434892.477	1444214.291	2017
Middle SDI	Peptic ulcer disease	Both	1428813.038	1424166.76	1433459.317	2018
Middle SDI	Peptic ulcer disease	Both	1425966.104	1421319.002	1430613.205	2019
Middle SDI	Peptic ulcer disease	Both	1898144.894	1824288.354	1972001.434	2020
Middle SDI	Peptic ulcer disease	Both	1896280.116	1801486.231	1991074.002	2021
Middle SDI	Peptic ulcer disease	Both	1892548.905	1770067.257	2015030.553	2022
Middle SDI	Peptic ulcer disease	Both	1888655.032	1733186.161	2044123.904	2023
Middle SDI	Peptic ulcer disease	Both	1885823.68	1692932.745	2078714.616	2024
Middle SDI	Peptic ulcer disease	Both	1882866.607	1648843.272	2116889.942	2025
Middle SDI	Peptic ulcer disease	Both	1878832.944	1600678.435	2156987.452	2026
Middle SDI	Peptic ulcer disease	Both	1873171.185	1548420.191	2197922.18	2027
Middle SDI	Peptic ulcer disease	Both	1867203.272	1493474.51	2240932.034	2028
Middle SDI	Peptic ulcer disease	Both	1861905.605	1436749.642	2287061.568	2029
Middle SDI	Peptic ulcer disease	Both	1856222.447	1377519.089	2334925.805	2030
Low-middle SDI	Peptic ulcer disease	Male	1950521.259	1946651.418	1954391.101	1990
Low-middle SDI	Peptic ulcer disease	Male	1968075.425	1964189.917	1971960.934	1991

Low-middle SDI	Peptic ulcer disease	Male	1971996.283	1968107.011	1975885.555	1992
Low-middle SDI	Peptic ulcer disease	Male	1957535.199	1953660.262	1961410.137	1993
Low-middle SDI	Peptic ulcer disease	Male	1941660.281	1937801.15	1945519.412	1994
Low-middle SDI	Peptic ulcer disease	Male	1907344.708	1903519.902	1911169.515	1995
Low-middle SDI	Peptic ulcer disease	Male	1891184.339	1887375.799	1894992.879	1996
Low-middle SDI	Peptic ulcer disease	Male	1917519.846	1913684.849	1921354.844	1997
Low-middle SDI	Peptic ulcer disease	Male	1894083.156	1890271.708	1897894.605	1998
Low-middle SDI	Peptic ulcer disease	Male	1844688.955	1840927.61	1848450.3	1999
Low-middle SDI	Peptic ulcer disease	Male	1801899.006	1798181.623	1805616.39	2000
Low-middle SDI	Peptic ulcer disease	Male	1756417.106	1752747.029	1760087.183	2001
Low-middle SDI	Peptic ulcer disease	Male	1702568.793	1698955.52	1706182.066	2002
Low-middle SDI	Peptic ulcer disease	Male	1621139.635	1617613.995	1624665.276	2003
Low-middle SDI	Peptic ulcer disease	Male	1549881.584	1546434.466	1553328.702	2004
Low-middle SDI	Peptic ulcer disease	Male	1535576.541	1532145.413	1539007.669	2005
Low-middle SDI	Peptic ulcer disease	Male	1503131.589	1499736.981	1506526.196	2006
Low-middle SDI	Peptic ulcer disease	Male	1460875.883	1457529.436	1464222.33	2007
Low-middle SDI	Peptic ulcer disease	Male	1450339.703	1447005.371	1453674.035	2008
Low-middle SDI	Peptic ulcer disease	Male	1388253.047	1384991.036	1391515.059	2009
Low-middle SDI	Peptic ulcer disease	Male	1345461.31	1342250.073	1348672.547	2010
Low-middle SDI	Peptic ulcer disease	Male	1315391.795	1312216.724	1318566.866	2011
Low-middle SDI	Peptic ulcer disease	Male	1286321.003	1283181.307	1289460.698	2012
Low-middle SDI	Peptic ulcer disease	Male	1266812.927	1263697.215	1269928.64	2013
Low-middle SDI	Peptic ulcer disease	Male	1229088.895	1226020.018	1232157.771	2014
Low-middle SDI	Peptic ulcer disease	Male	1209306.91	1206262.874	1212350.946	2015
Low-middle SDI	Peptic ulcer disease	Male	1213826.04	1210776.276	1216875.804	2016
Low-middle SDI	Peptic ulcer disease	Male	1219272.105	1216215.478	1222328.732	2017
Low-middle SDI	Peptic ulcer disease	Male	1213278.929	1210229.713	1216328.144	2018
Low-middle SDI	Peptic ulcer disease	Male	1204512.717	1201472.347	1207553.087	2019
Low-middle SDI	Peptic ulcer disease	Male	1514509.255	1453350.812	1575667.698	2020
Low-middle SDI	Peptic ulcer disease	Male	1516022.85	1426344.878	1605700.822	2021
Low-middle SDI	Peptic ulcer disease	Male	1516857.033	1390081.764	1643632.302	2022
Low-middle SDI	Peptic ulcer disease	Male	1517312.197	1347062.035	1687562.36	2023
Low-middle SDI	Peptic ulcer disease	Male	1517858.727	1298840.339	1736877.115	2024
Low-middle SDI	Peptic ulcer disease	Male	1518318.835	1245904.267	1790733.402	2025
Low-middle SDI	Peptic ulcer disease	Male	1518454.8	1188588.181	1848321.419	2026
Low-middle SDI	Peptic ulcer disease	Male	1517844.535	1126964.139	1908724.931	2027
Low-middle SDI	Peptic ulcer disease	Male	1516689.594	1061508.809	1971870.38	2028
Low-middle SDI	Peptic ulcer disease	Male	1515360.99	992707.7061	2038014.274	2029
Low-middle SDI	Peptic ulcer disease	Male	1513744.305	920655.5818	2106833.028	2030
Low-middle SDI	Peptic ulcer disease	Female	1265314.831	1262198.126	1268431.537	1990
Low-middle SDI	Peptic ulcer disease	Female	1273200.158	1270075.027	1276325.289	1991
Low-middle SDI	Peptic ulcer disease	Female	1301242.838	1298083.561	1304402.115	1992
Low-middle SDI	Peptic ulcer disease	Female	1285724.992	1282584.652	1288865.332	1993
Low-middle SDI	Peptic ulcer disease	Female	1276309.455	1273180.671	1279438.239	1994
Low-middle SDI	Peptic ulcer disease	Female	1267385.184	1264267.386	1270502.982	1995
Low-middle SDI	Peptic ulcer disease	Female	1279315.819	1276183.363	1282448.274	1996
Low-middle SDI	Peptic ulcer disease	Female	1334844.183	1331644.356	1338044.01	1997
Low-middle SDI	Peptic ulcer disease	Female	1326410.709	1323221.024	1329600.394	1998
Low-middle SDI	Peptic ulcer disease	Female	1311524.388	1308352.679	1314696.098	1999
Low-middle SDI	Peptic ulcer disease	Female	1307305.643	1304139.043	1310472.243	2000
Low-middle SDI	Peptic ulcer disease	Female	1289936.356	1286790.903	1293081.809	2001
Low-middle SDI	Peptic ulcer disease	Female	1260519.698	1257410.389	1263629.007	2002
Low-middle SDI	Peptic ulcer disease	Female	1224545.557	1221481.018	1227610.096	2003
Low-middle SDI	Peptic ulcer disease	Female	1175297.053	1172294.885	1178299.22	2004
Low-middle SDI	Peptic ulcer disease	Female	1175431.332	1172428.994	1178433.669	2005
Low-middle SDI	Peptic ulcer disease	Female	1159473.299	1156491.447	1162455.151	2006
Low-middle SDI	Peptic ulcer disease	Female	1129770.881	1126827.542	1132714.22	2007
Low-middle SDI	Peptic ulcer disease	Female	1105474.852	1102563.397	1108386.308	2008
Low-middle SDI	Peptic ulcer disease	Female	1052472.319	1049631.665	1055312.972	2009
Low-middle SDI	Peptic ulcer disease	Female	1043393.217	1040564.868	1046221.567	2010
Low-middle SDI	Peptic ulcer disease	Female	1033049.879	1030235.61	1035864.148	2011
Low-middle SDI	Peptic ulcer disease	Female	998103.6602	995337.5027	1000869.818	2012

Low-middle SDI	Peptic ulcer disease	Female	1004030.662	1001256.287	1006805.037	2013
Low-middle SDI	Peptic ulcer disease	Female	981093.8755	978351.4246	983836.3265	2014
Low-middle SDI	Peptic ulcer disease	Female	993834.0478	991073.787	996594.3087	2015
Low-middle SDI	Peptic ulcer disease	Female	1003541.841	1000768.096	1006315.587	2016
Low-middle SDI	Peptic ulcer disease	Female	1018668.606	1015873.987	1021463.225	2017
Low-middle SDI	Peptic ulcer disease	Female	1015355.721	1012565.511	1018145.93	2018
Low-middle SDI	Peptic ulcer disease	Female	1012368.924	1009581.385	1015156.463	2019
Low-middle SDI	Peptic ulcer disease	Female	1253014.364	1194252.129	1311776.599	2020
Low-middle SDI	Peptic ulcer disease	Female	1258421.74	1176071.572	1340771.908	2021
Low-middle SDI	Peptic ulcer disease	Female	1263007.169	1149741.642	1376272.696	2022
Low-middle SDI	Peptic ulcer disease	Female	1267203.75	1117409.028	1416998.472	2023
Low-middle SDI	Peptic ulcer disease	Female	1271639.523	1080579.933	1462699.113	2024
Low-middle SDI	Peptic ulcer disease	Female	1276226.538	1039704.045	1512749.032	2025
Low-middle SDI	Peptic ulcer disease	Female	1280601.927	994887.7858	1566316.069	2026
Low-middle SDI	Peptic ulcer disease	Female	1284405.943	946162.5379	1622649.347	2027
Low-middle SDI	Peptic ulcer disease	Female	1288007.012	894039.72	1681974.304	2028
Low-middle SDI	Peptic ulcer disease	Female	1291847.767	838956.9836	1744738.55	2029
Low-middle SDI	Peptic ulcer disease	Female	1295770.756	780883.0968	1810658.414	2030
Low-middle SDI	Peptic ulcer disease	Both	3215836.091	3208849.543	3222822.638	1990
Low-middle SDI	Peptic ulcer disease	Both	3241275.583	3234264.944	3248286.223	1991
Low-middle SDI	Peptic ulcer disease	Both	3273239.121	3266190.572	3280287.67	1992
Low-middle SDI	Peptic ulcer disease	Both	3243260.192	3236244.914	3250275.469	1993
Low-middle SDI	Peptic ulcer disease	Both	3217969.736	3210981.821	3224957.651	1994
Low-middle SDI	Peptic ulcer disease	Both	3174729.892	3167787.288	3181672.496	1995
Low-middle SDI	Peptic ulcer disease	Both	3170500.158	3163559.163	3177441.153	1996
Low-middle SDI	Peptic ulcer disease	Both	3252364.029	3245329.205	3259398.854	1997
Low-middle SDI	Peptic ulcer disease	Both	3220493.865	3213492.731	3227494.999	1998
Low-middle SDI	Peptic ulcer disease	Both	3156213.344	3149280.289	3163146.398	1999
Low-middle SDI	Peptic ulcer disease	Both	3109204.649	3102320.666	3116088.633	2000
Low-middle SDI	Peptic ulcer disease	Both	3046353.462	3039537.932	3053168.992	2001
Low-middle SDI	Peptic ulcer disease	Both	2963088.491	2956365.91	2969811.073	2002
Low-middle SDI	Peptic ulcer disease	Both	2845685.192	2839095.013	2852275.372	2003
Low-middle SDI	Peptic ulcer disease	Both	2725178.636	2718729.351	2731627.922	2004
Low-middle SDI	Peptic ulcer disease	Both	2711007.873	2704574.408	2717441.338	2005
Low-middle SDI	Peptic ulcer disease	Both	2662604.888	2656228.428	2668981.347	2006
Low-middle SDI	Peptic ulcer disease	Both	2590646.764	2584356.978	2596936.55	2007
Low-middle SDI	Peptic ulcer disease	Both	2555814.555	2549568.768	2562060.343	2008
Low-middle SDI	Peptic ulcer disease	Both	2440725.366	2434622.701	2446828.031	2009
Low-middle SDI	Peptic ulcer disease	Both	2388854.527	2382814.94	2394894.115	2010
Low-middle SDI	Peptic ulcer disease	Both	2348441.674	2342452.334	2354431.014	2011
Low-middle SDI	Peptic ulcer disease	Both	2284424.663	2278518.809	2290330.516	2012
Low-middle SDI	Peptic ulcer disease	Both	2270843.589	2264953.502	2276733.677	2013
Low-middle SDI	Peptic ulcer disease	Both	2210182.77	2204371.443	2215994.098	2014
Low-middle SDI	Peptic ulcer disease	Both	2203140.958	2197336.661	2208945.254	2015
Low-middle SDI	Peptic ulcer disease	Both	2217367.881	2211544.371	2223191.391	2016
Low-middle SDI	Peptic ulcer disease	Both	2237940.711	2232089.465	2243791.957	2017
Low-middle SDI	Peptic ulcer disease	Both	2228634.649	2222795.224	2234474.074	2018
Low-middle SDI	Peptic ulcer disease	Both	2216881.641	2211053.732	2222709.55	2019
Low-middle SDI	Peptic ulcer disease	Both	2767523.619	2647602.94	2887444.297	2020
Low-middle SDI	Peptic ulcer disease	Both	2774444.59	2602416.451	2946472.73	2021
Low-middle SDI	Peptic ulcer disease	Both	2779864.202	2539823.406	3019904.998	2022
Low-middle SDI	Peptic ulcer disease	Both	2784515.948	2464471.063	3104560.832	2023
Low-middle SDI	Peptic ulcer disease	Both	2789498.25	2379420.272	3199576.228	2024
Low-middle SDI	Peptic ulcer disease	Both	2794545.373	2285608.312	3303482.434	2025
Low-middle SDI	Peptic ulcer disease	Both	2799056.727	2183475.966	3414637.488	2026
Low-middle SDI	Peptic ulcer disease	Both	2802250.477	2073126.677	3531374.278	2027
Low-middle SDI	Peptic ulcer disease	Both	2804696.606	1955548.529	3653844.684	2028
Low-middle SDI	Peptic ulcer disease	Both	2807208.757	1831664.69	3782752.824	2029
Low-middle SDI	Peptic ulcer disease	Both	2809515.06	1701538.679	3917491.442	2030
Low SDI	Peptic ulcer disease	Male	631493.9237	629295.5547	633692.2927	1990
Low SDI	Peptic ulcer disease	Male	640633.862	638425.241	642842.4831	1991
Low SDI	Peptic ulcer disease	Male	647518.9163	645298.6995	649739.1331	1992

Low SDI	Peptic ulcer disease	Male	653018.4399	650788.8129	655248.067	1993
Low SDI	Peptic ulcer disease	Male	660573.5074	658331.0244	662815.9903	1994
Low SDI	Peptic ulcer disease	Male	660232.2072	657990.3554	662474.0591	1995
Low SDI	Peptic ulcer disease	Male	664678.27	662428.8344	666927.7057	1996
Low SDI	Peptic ulcer disease	Male	674617.7646	672351.44	676884.0893	1997
Low SDI	Peptic ulcer disease	Male	678435.8389	676163.0658	680708.6119	1998
Low SDI	Peptic ulcer disease	Male	679548.655	677273.9705	681823.3395	1999
Low SDI	Peptic ulcer disease	Male	679968.2632	677692.8884	682243.6379	2000
Low SDI	Peptic ulcer disease	Male	669297.5511	667040.3097	671554.7926	2001
Low SDI	Peptic ulcer disease	Male	656972.6194	654736.4775	659208.7613	2002
Low SDI	Peptic ulcer disease	Male	651177.3618	648951.2035	653403.5201	2003
Low SDI	Peptic ulcer disease	Male	636671.3017	634470.2949	638872.3085	2004
Low SDI	Peptic ulcer disease	Male	623596.4912	621418.3942	625774.5882	2005
Low SDI	Peptic ulcer disease	Male	615791.7633	613627.4648	617956.0619	2006
Low SDI	Peptic ulcer disease	Male	625493.0289	623311.5918	627674.4661	2007
Low SDI	Peptic ulcer disease	Male	615599.2126	613435.2568	617763.1684	2008
Low SDI	Peptic ulcer disease	Male	608915.0517	606763.0353	611067.0681	2009
Low SDI	Peptic ulcer disease	Male	595615.7996	593487.6472	597743.952	2010
Low SDI	Peptic ulcer disease	Male	589182.1045	587065.5854	591298.6235	2011
Low SDI	Peptic ulcer disease	Male	584219.9635	582112.5025	586327.4245	2012
Low SDI	Peptic ulcer disease	Male	590571.951	588452.9734	592690.9286	2013
Low SDI	Peptic ulcer disease	Male	581162.437	579060.5728	583264.3011	2014
Low SDI	Peptic ulcer disease	Male	585213.6623	583104.3535	587322.971	2015
Low SDI	Peptic ulcer disease	Male	583848.7312	581741.8123	585955.6501	2016
Low SDI	Peptic ulcer disease	Male	587307.8288	585194.5644	589421.0932	2017
Low SDI	Peptic ulcer disease	Male	587164.6402	585051.3084	589277.972	2018
Low SDI	Peptic ulcer disease	Male	584296.7997	582182.5791	586411.0202	2019
Low SDI	Peptic ulcer disease	Male	488673.0727	474679.4561	502666.6893	2020
Low SDI	Peptic ulcer disease	Male	489795.4038	468657.0891	510933.7184	2021
Low SDI	Peptic ulcer disease	Male	490939.5507	460600.4709	521278.6305	2022
Low SDI	Peptic ulcer disease	Male	492213.8416	451107.108	533320.5752	2023
Low SDI	Peptic ulcer disease	Male	493677.0492	440464.6611	546889.4373	2024
Low SDI	Peptic ulcer disease	Male	495261.7401	428735.3006	561788.1795	2025
Low SDI	Peptic ulcer disease	Male	496912.6553	415975.7201	577849.5905	2026
Low SDI	Peptic ulcer disease	Male	498536.5962	402179.8656	594893.3269	2027
Low SDI	Peptic ulcer disease	Male	500198.2633	387450.0479	612946.4787	2028
Low SDI	Peptic ulcer disease	Male	501922.6434	371831.678	632013.6089	2029
Low SDI	Peptic ulcer disease	Male	503638.0052	355288.6923	651987.3181	2030
Low SDI	Peptic ulcer disease	Female	461519.1929	459639.9606	463398.4251	1990
Low SDI	Peptic ulcer disease	Female	469076.2888	467185.8819	470966.6957	1991
Low SDI	Peptic ulcer disease	Female	480311.0183	478398.327	482223.7097	1992
Low SDI	Peptic ulcer disease	Female	481369.4248	479454.6474	483284.2022	1993
Low SDI	Peptic ulcer disease	Female	483051.473	481133.4058	484969.5403	1994
Low SDI	Peptic ulcer disease	Female	483453.9831	481535.1528	485372.8133	1995
Low SDI	Peptic ulcer disease	Female	486870.3291	484944.6835	488795.9747	1996
Low SDI	Peptic ulcer disease	Female	494183.6016	492243.4325	496123.7707	1997
Low SDI	Peptic ulcer disease	Female	500320.9382	498368.6578	502273.2185	1998
Low SDI	Peptic ulcer disease	Female	503711.2809	501752.3208	505670.241	1999
Low SDI	Peptic ulcer disease	Female	506136.8297	504173.1181	508100.5413	2000
Low SDI	Peptic ulcer disease	Female	511669.2963	509694.8156	513643.777	2001
Low SDI	Peptic ulcer disease	Female	513144.3592	511167.0345	515121.684	2002
Low SDI	Peptic ulcer disease	Female	511036.6944	509063.4951	513009.8937	2003
Low SDI	Peptic ulcer disease	Female	507791.7553	505824.8866	509758.624	2004
Low SDI	Peptic ulcer disease	Female	510182.1669	508210.631	512153.7028	2005
Low SDI	Peptic ulcer disease	Female	504804.2172	502843.1838	506765.2505	2006
Low SDI	Peptic ulcer disease	Female	495941.9313	493998.3255	497885.5372	2007
Low SDI	Peptic ulcer disease	Female	493923.9153	491984.3107	495863.5199	2008
Low SDI	Peptic ulcer disease	Female	488656.2051	486727.079	490585.3312	2009
Low SDI	Peptic ulcer disease	Female	481953.3366	480037.5897	483869.0835	2010
Low SDI	Peptic ulcer disease	Female	474245.2638	472345.0254	476145.5022	2011
Low SDI	Peptic ulcer disease	Female	476105.3706	474201.3915	478009.3496	2012
Low SDI	Peptic ulcer disease	Female	474218.2352	472318.0874	476118.383	2013

Low SDI	Peptic ulcer disease	Female	482355.087	480438.5512	484271.6228	2014
Low SDI	Peptic ulcer disease	Female	481961.3539	480045.5642	483877.1435	2015
Low SDI	Peptic ulcer disease	Female	484455.13	482534.2913	486375.9687	2016
Low SDI	Peptic ulcer disease	Female	490168.6943	488236.4501	492100.9385	2017
Low SDI	Peptic ulcer disease	Female	491527.9736	489592.6548	493463.2924	2018
Low SDI	Peptic ulcer disease	Female	491408.6279	489469.3497	493347.9061	2019
Low SDI	Peptic ulcer disease	Female	411192.754	397713.7363	424671.7718	2020
Low SDI	Peptic ulcer disease	Female	414703.3185	395186.9723	434219.6648	2021
Low SDI	Peptic ulcer disease	Female	418179.5911	390799.6916	445559.4907	2022
Low SDI	Peptic ulcer disease	Female	421785.9359	385094.1181	458477.7537	2023
Low SDI	Peptic ulcer disease	Female	425659.0411	378376.6363	472941.4459	2024
Low SDI	Peptic ulcer disease	Female	429646.2505	370599.8173	488692.6838	2025
Low SDI	Peptic ulcer disease	Female	433636.0176	361748.4289	505523.6064	2026
Low SDI	Peptic ulcer disease	Female	437515.7098	351786.6343	523244.7853	2027
Low SDI	Peptic ulcer disease	Female	441389.4785	340836.386	541942.5711	2028
Low SDI	Peptic ulcer disease	Female	445358.1213	328986.3092	561729.9334	2029
Low SDI	Peptic ulcer disease	Female	449317.2013	316160.1928	582474.2098	2030
Low SDI	Peptic ulcer disease	Both	1093013.117	1088935.515	1097090.718	1990
Low SDI	Peptic ulcer disease	Both	1109710.151	1105611.123	1113809.179	1991
Low SDI	Peptic ulcer disease	Both	1127829.935	1123697.026	1131962.843	1992
Low SDI	Peptic ulcer disease	Both	1134387.865	1130243.46	1138532.269	1993
Low SDI	Peptic ulcer disease	Both	1143624.98	1139464.43	1147785.531	1994
Low SDI	Peptic ulcer disease	Both	1143686.19	1139525.508	1147846.872	1995
Low SDI	Peptic ulcer disease	Both	1151548.599	1147373.518	1155723.68	1996
Low SDI	Peptic ulcer disease	Both	1168801.366	1164594.872	1173007.86	1997
Low SDI	Peptic ulcer disease	Both	1178756.777	1174531.724	1182981.83	1998
Low SDI	Peptic ulcer disease	Both	1183259.936	1179026.291	1187493.58	1999
Low SDI	Peptic ulcer disease	Both	1186105.093	1181866.007	1190344.179	2000
Low SDI	Peptic ulcer disease	Both	1180966.847	1176735.125	1185198.57	2001
Low SDI	Peptic ulcer disease	Both	1170116.979	1165903.512	1174330.445	2002
Low SDI	Peptic ulcer disease	Both	1162214.056	1158014.699	1166413.414	2003
Low SDI	Peptic ulcer disease	Both	1144463.057	1140295.182	1148630.932	2004
Low SDI	Peptic ulcer disease	Both	1133778.658	1129629.025	1137928.291	2005
Low SDI	Peptic ulcer disease	Both	1120595.98	1116470.649	1124721.312	2006
Low SDI	Peptic ulcer disease	Both	1121434.96	1117309.917	1125560.003	2007
Low SDI	Peptic ulcer disease	Both	1109523.128	1105419.567	1113626.688	2008
Low SDI	Peptic ulcer disease	Both	1097571.257	1093490.114	1101652.399	2009
Low SDI	Peptic ulcer disease	Both	1077569.136	1073525.237	1081613.035	2010
Low SDI	Peptic ulcer disease	Both	1063427.368	1059410.611	1067444.126	2011
Low SDI	Peptic ulcer disease	Both	1060325.334	1056313.894	1064336.774	2012
Low SDI	Peptic ulcer disease	Both	1064790.186	1060771.061	1068809.312	2013
Low SDI	Peptic ulcer disease	Both	1063517.524	1059499.124	1067535.924	2014
Low SDI	Peptic ulcer disease	Both	1067175.016	1063149.918	1071200.114	2015
Low SDI	Peptic ulcer disease	Both	1068303.861	1064276.104	1072331.619	2016
Low SDI	Peptic ulcer disease	Both	1077476.523	1073431.014	1081522.032	2017
Low SDI	Peptic ulcer disease	Both	1078692.614	1074643.963	1082741.264	2018
Low SDI	Peptic ulcer disease	Both	1075705.428	1071651.929	1079758.926	2019
Low SDI	Peptic ulcer disease	Both	899865.8268	872393.1924	927338.4611	2020
Low SDI	Peptic ulcer disease	Both	904498.7223	863844.0614	945153.3833	2021
Low SDI	Peptic ulcer disease	Both	909119.1418	851400.1624	966838.1212	2022
Low SDI	Peptic ulcer disease	Both	913999.7775	836201.2261	991798.3289	2023
Low SDI	Peptic ulcer disease	Both	919336.0903	818841.2974	1019830.883	2024
Low SDI	Peptic ulcer disease	Both	924907.9906	799335.1179	1050480.863	2025
Low SDI	Peptic ulcer disease	Both	930548.6729	777724.1489	1083373.197	2026
Low SDI	Peptic ulcer disease	Both	936052.3061	753966.4999	1118138.112	2027
Low SDI	Peptic ulcer disease	Both	941587.7418	728286.4339	1154889.05	2028
Low SDI	Peptic ulcer disease	Both	947280.7647	700817.9872	1193743.542	2029
Low SDI	Peptic ulcer disease	Both	952955.2065	671448.8852	1234461.528	2030
Global	Gastritis and duodenitis	Male	1144997.492	1142036.349	1147958.634	1990
Global	Gastritis and duodenitis	Male	1150886.727	1147923.409	1153850.045	1991
Global	Gastritis and duodenitis	Male	1154036.244	1151069.363	1157003.125	1992
Global	Gastritis and duodenitis	Male	1155677.132	1152708.183	1158646.082	1993

Global	Gastritis and duodenitis	Male	1158398.631	1155426.261	1161371	1994
Global	Gastritis and duodenitis	Male	1161183.727	1158207.832	1164159.622	1995
Global	Gastritis and duodenitis	Male	1160734.319	1157759.047	1163709.592	1996
Global	Gastritis and duodenitis	Male	1158845.858	1155873.041	1161818.674	1997
Global	Gastritis and duodenitis	Male	1161186.844	1158211.01	1164162.678	1998
Global	Gastritis and duodenitis	Male	1166954.854	1163971.589	1169938.12	1999
Global	Gastritis and duodenitis	Male	1178238.824	1175241.084	1181236.564	2000
Global	Gastritis and duodenitis	Male	1186574.746	1183566.358	1189583.134	2001
Global	Gastritis and duodenitis	Male	1199343.633	1196318.987	1202368.279	2002
Global	Gastritis and duodenitis	Male	1211755.883	1208715.503	1214796.264	2003
Global	Gastritis and duodenitis	Male	1222196.025	1219142.47	1225249.579	2004
Global	Gastritis and duodenitis	Male	1228222.46	1225161.339	1231283.581	2005
Global	Gastritis and duodenitis	Male	1230640.147	1227576.037	1233704.257	2006
Global	Gastritis and duodenitis	Male	1229151.642	1226089.437	1232213.847	2007
Global	Gastritis and duodenitis	Male	1233008.882	1229941.863	1236075.901	2008
Global	Gastritis and duodenitis	Male	1235734.076	1232663.654	1238804.498	2009
Global	Gastritis and duodenitis	Male	1240067.337	1236991.506	1243143.169	2010
Global	Gastritis and duodenitis	Male	1248960.809	1245873.897	1252047.72	2011
Global	Gastritis and duodenitis	Male	1259708.11	1256607.857	1262808.363	2012
Global	Gastritis and duodenitis	Male	1273049.625	1269932.887	1276166.364	2013
Global	Gastritis and duodenitis	Male	1282984.462	1279855.483	1286113.441	2014
Global	Gastritis and duodenitis	Male	1298195.866	1295048.219	1301343.513	2015
Global	Gastritis and duodenitis	Male	1322082.933	1318906.195	1325259.671	2016
Global	Gastritis and duodenitis	Male	1341100.459	1337900.781	1344300.136	2017
Global	Gastritis and duodenitis	Male	1341495.286	1338294.705	1344695.867	2018
Global	Gastritis and duodenitis	Male	1344051.927	1340843.069	1347260.785	2019
Global	Gastritis and duodenitis	Male	1364725.101	1332763.041	1396687.16	2020
Global	Gastritis and duodenitis	Male	1372452.352	1326408.194	1418496.51	2021
Global	Gastritis and duodenitis	Male	1379669.699	1315283.363	1444056.034	2022
Global	Gastritis and duodenitis	Male	1386685.998	1300676.285	1472695.711	2023
Global	Gastritis and duodenitis	Male	1393732.971	1283304.98	1504160.962	2024
Global	Gastritis and duodenitis	Male	1400735.937	1263374.913	1538096.96	2025
Global	Gastritis and duodenitis	Male	1407552.297	1241020.986	1574083.609	2026
Global	Gastritis and duodenitis	Male	1413980.33	1216240.641	1611720.018	2027
Global	Gastritis and duodenitis	Male	1420197.847	1189297.37	1651098.323	2028
Global	Gastritis and duodenitis	Male	1426427.459	1160427.019	1692427.9	2029
Global	Gastritis and duodenitis	Male	1432570.787	1129578.541	1735563.034	2030
Global	Gastritis and duodenitis	Female	1070692.462	1067827.788	1073557.135	1990
Global	Gastritis and duodenitis	Female	1071709.604	1068846.665	1074572.543	1991
Global	Gastritis and duodenitis	Female	1074339	1071473.009	1077204.99	1992
Global	Gastritis and duodenitis	Female	1074796.1	1071929.54	1077662.661	1993
Global	Gastritis and duodenitis	Female	1080714.657	1077840.22	1083589.094	1994
Global	Gastritis and duodenitis	Female	1085646.708	1082765.735	1088527.68	1995
Global	Gastritis and duodenitis	Female	1091113.134	1088224.917	1094001.351	1996
Global	Gastritis and duodenitis	Female	1100654.379	1097753.517	1103555.24	1997
Global	Gastritis and duodenitis	Female	1109478.168	1106565.651	1112390.685	1998
Global	Gastritis and duodenitis	Female	1121247.624	1118319.628	1124175.62	1999
Global	Gastritis and duodenitis	Female	1137369.66	1134420.588	1140318.731	2000
Global	Gastritis and duodenitis	Female	1151558.072	1148590.575	1154525.569	2001
Global	Gastritis and duodenitis	Female	1168966.865	1165976.909	1171956.822	2002
Global	Gastritis and duodenitis	Female	1187500.387	1184486.696	1190514.079	2003
Global	Gastritis and duodenitis	Female	1203919.365	1200884.802	1206953.928	2004
Global	Gastritis and duodenitis	Female	1218206.596	1215154.001	1221259.19	2005
Global	Gastritis and duodenitis	Female	1229597.242	1226530.369	1232664.114	2006
Global	Gastritis and duodenitis	Female	1239046.964	1235968.306	1242125.622	2007
Global	Gastritis and duodenitis	Female	1254116.864	1251019.474	1257214.253	2008
Global	Gastritis and duodenitis	Female	1269465.001	1266348.637	1272581.365	2009
Global	Gastritis and duodenitis	Female	1285574.443	1282438.283	1288710.603	2010
Global	Gastritis and duodenitis	Female	1300561.61	1297407.146	1303716.075	2011
Global	Gastritis and duodenitis	Female	1313569.313	1310399.046	1316739.58	2012
Global	Gastritis and duodenitis	Female	1330973.303	1327782.012	1334164.594	2013
Global	Gastritis and duodenitis	Female	1348809.357	1345596.65	1352022.064	2014

Global	Gastritis and duodenitis	Female	1371064.775	1367825.539	1374304.01	2015
Global	Gastritis and duodenitis	Female	1404225.21	1400946.864	1407503.556	2016
Global	Gastritis and duodenitis	Female	1438749.447	1435430.876	1442068.018	2017
Global	Gastritis and duodenitis	Female	1456766.028	1453426.278	1460105.779	2018
Global	Gastritis and duodenitis	Female	1470954.651	1467595.929	1474313.372	2019
Global	Gastritis and duodenitis	Female	1511366.077	1468371.425	1554360.73	2020
Global	Gastritis and duodenitis	Female	1536242.561	1477352.89	1595132.232	2021
Global	Gastritis and duodenitis	Female	1560640.807	1480687.729	1640593.885	2022
Global	Gastritis and duodenitis	Female	1584807.131	1479519.479	1690094.783	2023
Global	Gastritis and duodenitis	Female	1609195.841	1474757.171	1743634.512	2024
Global	Gastritis and duodenitis	Female	1633730.244	1466585.829	1800874.659	2025
Global	Gastritis and duodenitis	Female	1658074.742	1454943.599	1861205.885	2026
Global	Gastritis and duodenitis	Female	1682005.829	1439782.438	1924229.22	2027
Global	Gastritis and duodenitis	Female	1705781.405	1421397.873	1990164.936	2028
Global	Gastritis and duodenitis	Female	1729733.858	1400070.971	2059396.744	2029
Global	Gastritis and duodenitis	Female	1753710.507	1375660.285	2131760.728	2030
Global	Gastritis and duodenitis	Both	2215689.954	2209864.138	2221515.77	1990
Global	Gastritis and duodenitis	Both	2222596.332	2216770.075	2228422.589	1991
Global	Gastritis and duodenitis	Both	2228375.243	2222542.372	2234208.115	1992
Global	Gastritis and duodenitis	Both	2230473.233	2224637.723	2236308.742	1993
Global	Gastritis and duodenitis	Both	2239113.288	2233266.481	2244960.095	1994
Global	Gastritis and duodenitis	Both	2246830.435	2240973.567	2252687.302	1995
Global	Gastritis and duodenitis	Both	2251847.454	2245983.964	2257710.944	1996
Global	Gastritis and duodenitis	Both	2259500.236	2253626.558	2265373.915	1997
Global	Gastritis and duodenitis	Both	2270665.012	2264776.661	2276553.363	1998
Global	Gastritis and duodenitis	Both	2288202.478	2282291.217	2294113.739	1999
Global	Gastritis and duodenitis	Both	2315608.483	2309661.672	2321555.294	2000
Global	Gastritis and duodenitis	Both	2338132.818	2332156.933	2344108.703	2001
Global	Gastritis and duodenitis	Both	2368310.498	2362295.896	2374325.101	2002
Global	Gastritis and duodenitis	Both	2399256.271	2393202.198	2405310.343	2003
Global	Gastritis and duodenitis	Both	2426115.389	2420027.273	2432203.506	2004
Global	Gastritis and duodenitis	Both	2446429.056	2440315.34	2452542.771	2005
Global	Gastritis and duodenitis	Both	2460237.388	2454106.405	2466368.371	2006
Global	Gastritis and duodenitis	Both	2468198.606	2462057.742	2474339.47	2007
Global	Gastritis and duodenitis	Both	2487125.745	2480961.337	2493290.154	2008
Global	Gastritis and duodenitis	Both	2505199.077	2499012.292	2511385.863	2009
Global	Gastritis and duodenitis	Both	2525641.78	2519429.789	2531853.772	2010
Global	Gastritis and duodenitis	Both	2549522.419	2543281.043	2555763.795	2011
Global	Gastritis and duodenitis	Both	2573277.423	2567006.902	2579547.943	2012
Global	Gastritis and duodenitis	Both	2604022.928	2597714.898	2610330.958	2013
Global	Gastritis and duodenitis	Both	2631793.819	2625452.133	2638135.504	2014
Global	Gastritis and duodenitis	Both	2669260.641	2662873.758	2675647.523	2015
Global	Gastritis and duodenitis	Both	2726308.143	2719853.059	2732763.227	2016
Global	Gastritis and duodenitis	Both	2779849.906	2773331.657	2786368.155	2017
Global	Gastritis and duodenitis	Both	2798261.314	2791720.983	2804801.645	2018
Global	Gastritis and duodenitis	Both	2815006.578	2808438.999	2821574.157	2019
Global	Gastritis and duodenitis	Both	2876091.178	2801134.466	2951047.89	2020
Global	Gastritis and duodenitis	Both	2908694.913	2803761.084	3013628.743	2021
Global	Gastritis and duodenitis	Both	2940310.505	2795971.092	3084649.919	2022
Global	Gastritis and duodenitis	Both	2971493.129	2780195.764	3162790.494	2023
Global	Gastritis and duodenitis	Both	3002928.813	2758062.151	3247795.474	2024
Global	Gastritis and duodenitis	Both	3034466.181	2729960.742	3338971.619	2025
Global	Gastritis and duodenitis	Both	3065627.039	2695964.585	3435289.493	2026
Global	Gastritis and duodenitis	Both	3095986.158	2656023.079	3535949.238	2027
Global	Gastritis and duodenitis	Both	3125979.251	2610695.243	3641263.259	2028
Global	Gastritis and duodenitis	Both	3156161.317	2560497.99	3751824.644	2029
Global	Gastritis and duodenitis	Both	3186281.294	2505238.826	3867323.762	2030
High SDI	Gastritis and duodenitis	Male	87046.09216	86242.71253	87849.4718	1990
High SDI	Gastritis and duodenitis	Male	86489.62808	85703.76082	87275.49535	1991
High SDI	Gastritis and duodenitis	Male	85791.36318	85010.74393	86571.98244	1992
High SDI	Gastritis and duodenitis	Male	85437.647	84658.81926	86216.47474	1993
High SDI	Gastritis and duodenitis	Male	85254.57405	84476.72494	86032.42316	1994

High SDI	Gastritis and duodenitis	Male	85085.97347	84309.0262	85862.92074	1995
High SDI	Gastritis and duodenitis	Male	84552.60843	83778.35652	85326.86034	1996
High SDI	Gastritis and duodenitis	Male	84290.29451	83517.39451	85063.19451	1997
High SDI	Gastritis and duodenitis	Male	84146.216	83373.89787	84918.53413	1998
High SDI	Gastritis and duodenitis	Male	83945.87994	83174.32642	84717.43346	1999
High SDI	Gastritis and duodenitis	Male	84136.82084	83364.21757	84909.42411	2000
High SDI	Gastritis and duodenitis	Male	83953.93204	83182.23494	84725.62915	2001
High SDI	Gastritis and duodenitis	Male	83958.76938	83187.02164	84730.51712	2002
High SDI	Gastritis and duodenitis	Male	83732.74411	82962.11576	84503.37246	2003
High SDI	Gastritis and duodenitis	Male	83167.43906	82399.62253	83935.25558	2004
High SDI	Gastritis and duodenitis	Male	83058.84454	82291.56881	83826.12028	2005
High SDI	Gastritis and duodenitis	Male	82949.2047	82182.51895	83715.89046	2006
High SDI	Gastritis and duodenitis	Male	82771.12317	82005.41412	83536.83221	2007
High SDI	Gastritis and duodenitis	Male	82735.86128	81970.4292	83501.29335	2008
High SDI	Gastritis and duodenitis	Male	82911.30992	82145.05741	83677.56242	2009
High SDI	Gastritis and duodenitis	Male	83353.01308	82584.62292	84121.40324	2010
High SDI	Gastritis and duodenitis	Male	84023.26817	83251.60066	84794.93568	2011
High SDI	Gastritis and duodenitis	Male	84891.00242	84115.10769	85666.89715	2012
High SDI	Gastritis and duodenitis	Male	86031.84766	85250.39261	86813.30272	2013
High SDI	Gastritis and duodenitis	Male	87017.71065	86231.40073	87804.02057	2014
High SDI	Gastritis and duodenitis	Male	88255.20041	87462.80244	89047.59839	2015
High SDI	Gastritis and duodenitis	Male	89704.8591	88905.42928	90504.28891	2016
High SDI	Gastritis and duodenitis	Male	90987.55533	90182.02286	91793.08779	2017
High SDI	Gastritis and duodenitis	Male	92170.66877	91357.88484	92983.45271	2018
High SDI	Gastritis and duodenitis	Male	93235.04612	92403.04793	94067.04431	2019
High SDI	Gastritis and duodenitis	Male	88932.22305	86633.79931	91230.64678	2020
High SDI	Gastritis and duodenitis	Male	89955.63945	86731.60828	93179.67063	2021
High SDI	Gastritis and duodenitis	Male	90939.82391	86500.84159	95378.80624	2022
High SDI	Gastritis and duodenitis	Male	91909.83724	86024.16864	97795.50584	2023
High SDI	Gastritis and duodenitis	Male	92887.16715	85352.11	100422.2243	2024
High SDI	Gastritis and duodenitis	Male	93865.90755	84495.5606	103236.2545	2025
High SDI	Gastritis and duodenitis	Male	94822.47145	83447.80624	106197.1367	2026
High SDI	Gastritis and duodenitis	Male	95738.99346	82203.24867	109274.7382	2027
High SDI	Gastritis and duodenitis	Male	96634.87778	80785.28244	112484.4731	2028
High SDI	Gastritis and duodenitis	Male	97529.22834	79212.77554	115845.6811	2029
High SDI	Gastritis and duodenitis	Male	98416.748	77482.84075	119350.6553	2030
High SDI	Gastritis and duodenitis	Female	120441.456	119487.4515	121395.4606	1990
High SDI	Gastritis and duodenitis	Female	118583.514	117644.2284	119522.7996	1991
High SDI	Gastritis and duodenitis	Female	116976.9985	116045.5162	117908.4808	1992
High SDI	Gastritis and duodenitis	Female	115958.4549	115031.2906	116885.6192	1993
High SDI	Gastritis and duodenitis	Female	115395.159	114470.4004	116319.9175	1994
High SDI	Gastritis and duodenitis	Female	115578.9749	114653.5281	116504.4218	1995
High SDI	Gastritis and duodenitis	Female	115863.9943	114937.4073	116790.5813	1996
High SDI	Gastritis and duodenitis	Female	116151.8444	115224.065	117079.6239	1997
High SDI	Gastritis and duodenitis	Female	116559.6545	115630.1384	117489.1707	1998
High SDI	Gastritis and duodenitis	Female	116883.3348	115952.403	117814.2667	1999
High SDI	Gastritis and duodenitis	Female	116660.808	115730.8027	117590.8133	2000
High SDI	Gastritis and duodenitis	Female	116127.3278	115199.5533	117055.1024	2001
High SDI	Gastritis and duodenitis	Female	115285.9977	114361.7297	116210.2656	2002
High SDI	Gastritis and duodenitis	Female	114398.3578	113477.7883	115318.9273	2003
High SDI	Gastritis and duodenitis	Female	113393.3741	112476.9989	114309.7494	2004
High SDI	Gastritis and duodenitis	Female	113152.8016	112237.4294	114068.1737	2005
High SDI	Gastritis and duodenitis	Female	113074.9297	112159.8801	113989.9793	2006
High SDI	Gastritis and duodenitis	Female	113114.4494	112199.2327	114029.666	2007
High SDI	Gastritis and duodenitis	Female	113426.8775	112510.3554	114343.3996	2008
High SDI	Gastritis and duodenitis	Female	113878.3609	112959.9462	114796.7756	2009
High SDI	Gastritis and duodenitis	Female	114741.5492	113819.5473	115663.551	2010
High SDI	Gastritis and duodenitis	Female	116075.7794	115148.2612	117003.2976	2011
High SDI	Gastritis and duodenitis	Female	117612.1814	116678.34	118546.0228	2012
High SDI	Gastritis and duodenitis	Female	119150.2019	118210.0488	120090.3549	2013
High SDI	Gastritis and duodenitis	Female	120707.5615	119761.0256	121654.0974	2014
High SDI	Gastritis and duodenitis	Female	122559.6403	121605.5673	123513.7134	2015

High SDI	Gastritis and duodenitis	Female	124498.309	123536.4252	125460.1927	2016
High SDI	Gastritis and duodenitis	Female	126319.5606	125350.3843	127288.7368	2017
High SDI	Gastritis and duodenitis	Female	128163.671	127186.1661	129141.1759	2018
High SDI	Gastritis and duodenitis	Female	130103.6588	129111.8136	131095.5039	2019
High SDI	Gastritis and duodenitis	Female	124853.236	120807.1496	128899.3225	2020
High SDI	Gastritis and duodenitis	Female	126471.2434	121001.1795	131941.3073	2021
High SDI	Gastritis and duodenitis	Female	128048.8177	120693.092	135404.5433	2022
High SDI	Gastritis and duodenitis	Female	129615.2858	119996.9755	139233.5961	2023
High SDI	Gastritis and duodenitis	Female	131190.4185	118980.5623	143400.2748	2024
High SDI	Gastritis and duodenitis	Female	132768.3077	117666.4532	147870.1622	2025
High SDI	Gastritis and duodenitis	Female	134324.4751	116054.2346	152594.7156	2026
High SDI	Gastritis and duodenitis	Female	135840.7857	114142.6503	157538.9212	2027
High SDI	Gastritis and duodenitis	Female	137335.5786	111956.7258	162714.4313	2028
High SDI	Gastritis and duodenitis	Female	138822.3612	109512.4421	168132.2803	2029
High SDI	Gastritis and duodenitis	Female	140296.0744	106808.9772	173783.1715	2030
High SDI	Gastritis and duodenitis	Both	207487.5482	205730.164	209244.9324	1990
High SDI	Gastritis and duodenitis	Both	205073.1421	203347.9892	206798.295	1991
High SDI	Gastritis and duodenitis	Both	202768.3617	201056.2601	204480.4632	1992
High SDI	Gastritis and duodenitis	Both	201396.1019	199690.1099	203102.0939	1993
High SDI	Gastritis and duodenitis	Both	200649.733	198947.1254	202352.3407	1994
High SDI	Gastritis and duodenitis	Both	200664.9484	198962.5543	202367.3426	1995
High SDI	Gastritis and duodenitis	Both	200416.6028	198715.7638	202117.4417	1996
High SDI	Gastritis and duodenitis	Both	200442.1389	198741.4595	202142.8184	1997
High SDI	Gastritis and duodenitis	Both	200705.8705	199004.0362	202407.7048	1998
High SDI	Gastritis and duodenitis	Both	200829.2148	199126.7294	202531.7001	1999
High SDI	Gastritis and duodenitis	Both	200797.6288	199095.0203	202500.2374	2000
High SDI	Gastritis and duodenitis	Both	200081.2599	198381.7882	201780.7315	2001
High SDI	Gastritis and duodenitis	Both	199244.7671	197548.7514	200940.7828	2002
High SDI	Gastritis and duodenitis	Both	198131.1019	196439.9041	199822.2997	2003
High SDI	Gastritis and duodenitis	Both	196560.8132	194876.6214	198245.005	2004
High SDI	Gastritis and duodenitis	Both	196211.6461	194528.9982	197894.294	2005
High SDI	Gastritis and duodenitis	Both	196024.1344	194342.3991	197705.8698	2006
High SDI	Gastritis and duodenitis	Both	195885.5725	194204.6468	197566.4983	2007
High SDI	Gastritis and duodenitis	Both	196162.7388	194480.7846	197844.693	2008
High SDI	Gastritis and duodenitis	Both	196789.6708	195105.0037	198474.338	2009
High SDI	Gastritis and duodenitis	Both	198094.5623	196404.1703	199784.9543	2010
High SDI	Gastritis and duodenitis	Both	200099.0476	198399.8619	201798.2333	2011
High SDI	Gastritis and duodenitis	Both	202503.1838	200793.4477	204212.9199	2012
High SDI	Gastritis and duodenitis	Both	205182.0495	203460.4414	206903.6576	2013
High SDI	Gastritis and duodenitis	Both	207725.2722	205992.4263	209458.118	2014
High SDI	Gastritis and duodenitis	Both	210814.8407	209068.3697	212561.3118	2015
High SDI	Gastritis and duodenitis	Both	214203.1681	212441.8545	215964.4816	2016
High SDI	Gastritis and duodenitis	Both	217307.1159	215532.4072	219081.8246	2017
High SDI	Gastritis and duodenitis	Both	220334.3398	218544.0509	222124.6286	2018
High SDI	Gastritis and duodenitis	Both	223338.7049	221514.8616	225162.5483	2019
High SDI	Gastritis and duodenitis	Both	213785.4591	207440.9489	220129.9693	2020
High SDI	Gastritis and duodenitis	Both	216426.8828	207732.7878	225120.9779	2021
High SDI	Gastritis and duodenitis	Both	218988.6416	207193.9336	230783.3495	2022
High SDI	Gastritis and duodenitis	Both	221525.1231	206021.1441	237029.102	2023
High SDI	Gastritis and duodenitis	Both	224077.5857	204332.6723	243822.4991	2024
High SDI	Gastritis and duodenitis	Both	226634.2153	202162.0138	251106.4167	2025
High SDI	Gastritis and duodenitis	Both	229146.9466	199502.0409	258791.8522	2026
High SDI	Gastritis and duodenitis	Both	231579.7792	196345.8989	266813.6594	2027
High SDI	Gastritis and duodenitis	Both	233970.4563	192742.0083	275198.9044	2028
High SDI	Gastritis and duodenitis	Both	236351.5895	188725.2176	283977.9614	2029
High SDI	Gastritis and duodenitis	Both	238712.8224	184291.818	293133.8268	2030
High-middle SDI	Gastritis and duodenitis	Male	220074.8448	218782.8683	221366.8213	1990
High-middle SDI	Gastritis and duodenitis	Male	220476.8628	219200.7254	221753.0003	1991
High-middle SDI	Gastritis and duodenitis	Male	221344.9891	220066.7013	222623.2769	1992
High-middle SDI	Gastritis and duodenitis	Male	222034.0312	220754.2483	223313.8142	1993
High-middle SDI	Gastritis and duodenitis	Male	222636.1322	221354.7343	223917.5301	1994
High-middle SDI	Gastritis and duodenitis	Male	222381.3444	221100.6744	223662.0144	1995

High-middle SDI	Gastritis and duodenitis	Male	220435.7299	219160.9009	221710.559	1996
High-middle SDI	Gastritis and duodenitis	Male	217894.3429	216627.2005	219161.4854	1997
High-middle SDI	Gastritis and duodenitis	Male	216848.6579	215584.701	218112.6148	1998
High-middle SDI	Gastritis and duodenitis	Male	218140.4446	216872.5926	219408.2967	1999
High-middle SDI	Gastritis and duodenitis	Male	220653.661	219378.2844	221929.0376	2000
High-middle SDI	Gastritis and duodenitis	Male	222274.8148	220994.6472	223554.9825	2001
High-middle SDI	Gastritis and duodenitis	Male	224687.34	223400.0072	225974.6728	2002
High-middle SDI	Gastritis and duodenitis	Male	227045.7918	225751.4286	228340.1551	2003
High-middle SDI	Gastritis and duodenitis	Male	228585.1294	227286.1255	229884.1333	2004
High-middle SDI	Gastritis and duodenitis	Male	228612.1012	227312.9312	229911.2712	2005
High-middle SDI	Gastritis and duodenitis	Male	224522.0277	223235.0872	225808.9682	2006
High-middle SDI	Gastritis and duodenitis	Male	219243.8134	217972.6886	220514.9381	2007
High-middle SDI	Gastritis and duodenitis	Male	215383.1745	214123.662	216642.687	2008
High-middle SDI	Gastritis and duodenitis	Male	211592.931	210344.9353	212840.9266	2009
High-middle SDI	Gastritis and duodenitis	Male	210866.9139	209621.1445	212112.6833	2010
High-middle SDI	Gastritis and duodenitis	Male	210946.4666	209700.4383	212192.4949	2011
High-middle SDI	Gastritis and duodenitis	Male	211950.9593	210701.8129	213200.1056	2012
High-middle SDI	Gastritis and duodenitis	Male	213164.7875	211911.9458	214417.6291	2013
High-middle SDI	Gastritis and duodenitis	Male	214529.1232	213272.1426	215786.1037	2014
High-middle SDI	Gastritis and duodenitis	Male	216648.5263	215385.0425	217912.0101	2015
High-middle SDI	Gastritis and duodenitis	Male	219371.3865	218099.4416	220643.3313	2016
High-middle SDI	Gastritis and duodenitis	Male	221102.7081	219825.2049	222380.2112	2017
High-middle SDI	Gastritis and duodenitis	Male	221459.8802	220181.1624	222738.598	2018
High-middle SDI	Gastritis and duodenitis	Male	222443.8994	221145.6427	223742.156	2019
High-middle SDI	Gastritis and duodenitis	Male	111227.9837	108328.4891	114127.4782	2020
High-middle SDI	Gastritis and duodenitis	Male	111548.7904	106778.1228	116319.4581	2021
High-middle SDI	Gastritis and duodenitis	Male	111819.2961	104670.174	118968.4183	2022
High-middle SDI	Gastritis and duodenitis	Male	112085.7676	102171.749	121999.7861	2023
High-middle SDI	Gastritis and duodenitis	Male	112373.7107	99358.20979	125389.2116	2024
High-middle SDI	Gastritis and duodenitis	Male	112655.6113	96230.27718	129080.9455	2025
High-middle SDI	Gastritis and duodenitis	Male	112898.3785	92792.88176	133003.8753	2026
High-middle SDI	Gastritis and duodenitis	Male	113097.113	89067.46955	137126.7565	2027
High-middle SDI	Gastritis and duodenitis	Male	113291.7563	85102.88405	141480.6286	2028
High-middle SDI	Gastritis and duodenitis	Male	113499.832	80919.27602	146080.3879	2029
High-middle SDI	Gastritis and duodenitis	Male	113696.1897	76500.69201	150891.6875	2030
High-middle SDI	Gastritis and duodenitis	Female	223443.8153	222141.1911	224746.4396	1990
High-middle SDI	Gastritis and duodenitis	Female	222892.6188	221602.3216	224182.916	1991
High-middle SDI	Gastritis and duodenitis	Female	222360.9936	221072.7718	223649.2153	1992
High-middle SDI	Gastritis and duodenitis	Female	222503.5213	221215.0176	223792.0249	1993
High-middle SDI	Gastritis and duodenitis	Female	222976.0515	221686.3053	224265.7976	1994
High-middle SDI	Gastritis and duodenitis	Female	223131.4989	221841.3518	224421.6461	1995
High-middle SDI	Gastritis and duodenitis	Female	223288.8225	221998.243	224579.402	1996
High-middle SDI	Gastritis and duodenitis	Female	223811.1902	222519.0904	225103.29	1997
High-middle SDI	Gastritis and duodenitis	Female	224486.1224	223192.0329	225780.212	1998
High-middle SDI	Gastritis and duodenitis	Female	226502.4863	225202.4829	227802.4898	1999
High-middle SDI	Gastritis and duodenitis	Female	229043.4877	227736.0484	230350.9271	2000
High-middle SDI	Gastritis and duodenitis	Female	231621.1224	230306.1575	232936.0873	2001
High-middle SDI	Gastritis and duodenitis	Female	235272.5208	233946.9446	236598.097	2002
High-middle SDI	Gastritis and duodenitis	Female	239051.3222	237714.7787	240387.8657	2003
High-middle SDI	Gastritis and duodenitis	Female	242282.3875	240936.5042	243628.2708	2004
High-middle SDI	Gastritis and duodenitis	Female	243918.2029	242567.6401	245268.7658	2005
High-middle SDI	Gastritis and duodenitis	Female	243588.2769	242238.7393	244937.8145	2006
High-middle SDI	Gastritis and duodenitis	Female	241918.6899	240573.9979	243263.382	2007
High-middle SDI	Gastritis and duodenitis	Female	240280.1151	238940.1371	241620.093	2008
High-middle SDI	Gastritis and duodenitis	Female	239520.9169	238183.1251	240858.7087	2009
High-middle SDI	Gastritis and duodenitis	Female	240046.2265	238706.9311	241385.5219	2010
High-middle SDI	Gastritis and duodenitis	Female	241232.1745	239889.4757	242574.8734	2011
High-middle SDI	Gastritis and duodenitis	Female	242552.0223	241205.5419	243898.5028	2012
High-middle SDI	Gastritis and duodenitis	Female	244537.9262	243185.7944	245890.058	2013
High-middle SDI	Gastritis and duodenitis	Female	247213.4019	245853.6811	248573.1226	2014
High-middle SDI	Gastritis and duodenitis	Female	250695.5067	249325.9365	252065.077	2015
High-middle SDI	Gastritis and duodenitis	Female	256543.6757	255157.6831	257929.6683	2016

High-middle SDI	Gastritis and duodenitis	Female	262537.2455	261134.6626	263939.8285	2017
High-middle SDI	Gastritis and duodenitis	Female	265785.9992	264374.1685	267197.8298	2018
High-middle SDI	Gastritis and duodenitis	Female	268436.5329	267007.5196	269865.5461	2019
High-middle SDI	Gastritis and duodenitis	Female	143355.7297	139279.2217	147432.2376	2020
High-middle SDI	Gastritis and duodenitis	Female	145113.6531	138871.621	151355.6851	2021
High-middle SDI	Gastritis and duodenitis	Female	146798.4446	137736.53	155860.3591	2022
High-middle SDI	Gastritis and duodenitis	Female	148458.8612	136059.0642	160858.6583	2023
High-middle SDI	Gastritis and duodenitis	Female	150143.7915	133941.0129	166346.5701	2024
High-middle SDI	Gastritis and duodenitis	Female	151842.4654	131396.3591	172288.5716	2025
High-middle SDI	Gastritis and duodenitis	Female	153517.0773	128428.5609	178605.5937	2026
High-middle SDI	Gastritis and duodenitis	Female	155136.4039	125035.1096	185237.6982	2027
High-middle SDI	Gastritis and duodenitis	Female	156739.0894	121260.0284	192218.1505	2028
High-middle SDI	Gastritis and duodenitis	Female	158365.1231	117133.7116	199596.5345	2029
High-middle SDI	Gastritis and duodenitis	Female	159999.4939	112638.5753	207360.4125	2030
High-middle SDI	Gastritis and duodenitis	Both	443518.6601	440924.0593	446113.2609	1990
High-middle SDI	Gastritis and duodenitis	Both	443369.4816	440803.047	445935.9163	1991
High-middle SDI	Gastritis and duodenitis	Both	443705.9827	441139.4731	446272.4922	1992
High-middle SDI	Gastritis and duodenitis	Both	444537.5525	441969.2659	447105.8391	1993
High-middle SDI	Gastritis and duodenitis	Both	445612.1837	443041.0396	448183.3278	1994
High-middle SDI	Gastritis and duodenitis	Both	445512.8434	442942.0262	448083.6605	1995
High-middle SDI	Gastritis and duodenitis	Both	443724.5524	441159.1438	446289.961	1996
High-middle SDI	Gastritis and duodenitis	Both	441705.5331	439146.2909	444264.7753	1997
High-middle SDI	Gastritis and duodenitis	Both	441334.7803	438776.7339	443892.8268	1998
High-middle SDI	Gastritis and duodenitis	Both	444642.931	442075.0755	447210.7865	1999
High-middle SDI	Gastritis and duodenitis	Both	449697.1487	447114.3328	452279.9647	2000
High-middle SDI	Gastritis and duodenitis	Both	453895.9372	451300.8047	456491.0698	2001
High-middle SDI	Gastritis and duodenitis	Both	459959.8608	457346.9517	462572.7698	2002
High-middle SDI	Gastritis and duodenitis	Both	466097.114	463466.2073	468728.0208	2003
High-middle SDI	Gastritis and duodenitis	Both	470867.5169	468222.6297	473512.4041	2004
High-middle SDI	Gastritis and duodenitis	Both	472530.3041	469880.5712	475180.037	2005
High-middle SDI	Gastritis and duodenitis	Both	468110.3047	465473.8265	470746.7828	2006
High-middle SDI	Gastritis and duodenitis	Both	461162.5033	458546.6865	463778.3201	2007
High-middle SDI	Gastritis and duodenitis	Both	455663.2896	453063.7992	458262.78	2008
High-middle SDI	Gastritis and duodenitis	Both	451113.8479	448528.0605	453699.6353	2009
High-middle SDI	Gastritis and duodenitis	Both	450913.1404	448328.0756	453498.2052	2010
High-middle SDI	Gastritis and duodenitis	Both	452178.6411	449589.914	454767.3682	2011
High-middle SDI	Gastritis and duodenitis	Both	454502.9816	451907.3548	457098.6084	2012
High-middle SDI	Gastritis and duodenitis	Both	457702.7137	455097.7403	460307.6871	2013
High-middle SDI	Gastritis and duodenitis	Both	461742.525	459125.8238	464359.2263	2014
High-middle SDI	Gastritis and duodenitis	Both	467344.0331	464710.979	469977.0872	2015
High-middle SDI	Gastritis and duodenitis	Both	475915.0622	473257.1247	478572.9996	2016
High-middle SDI	Gastritis and duodenitis	Both	483639.9536	480959.8675	486320.0397	2017
High-middle SDI	Gastritis and duodenitis	Both	487245.8794	484555.3309	489936.4279	2018
High-middle SDI	Gastritis and duodenitis	Both	490880.4322	488153.1623	493607.7021	2019
High-middle SDI	Gastritis and duodenitis	Both	254583.7133	247607.7109	261559.7158	2020
High-middle SDI	Gastritis and duodenitis	Both	256662.4435	245649.7439	267675.1431	2021
High-middle SDI	Gastritis and duodenitis	Both	258617.7407	242406.7041	274828.7774	2022
High-middle SDI	Gastritis and duodenitis	Both	260544.6288	238230.8131	282858.4445	2023
High-middle SDI	Gastritis and duodenitis	Both	262517.5022	233299.2227	291735.7817	2024
High-middle SDI	Gastritis and duodenitis	Both	264498.0767	227626.6363	301369.5171	2025
High-middle SDI	Gastritis and duodenitis	Both	266415.4559	221221.4427	311609.469	2026
High-middle SDI	Gastritis and duodenitis	Both	268233.5169	214102.5792	322364.4546	2027
High-middle SDI	Gastritis and duodenitis	Both	270030.8458	206362.9125	333698.7791	2028
High-middle SDI	Gastritis and duodenitis	Both	271864.955	198052.9876	345676.9224	2029
High-middle SDI	Gastritis and duodenitis	Both	273695.6836	189139.2673	358252.0999	2030
Middle SDI	Gastritis and duodenitis	Male	408780.9393	407012.6856	410549.1931	1990
Middle SDI	Gastritis and duodenitis	Male	408983.519	407219.0034	410748.0345	1991
Middle SDI	Gastritis and duodenitis	Male	407259.5813	405499.2862	409019.8763	1992
Middle SDI	Gastritis and duodenitis	Male	404955.5609	403200.3499	406710.7718	1993
Middle SDI	Gastritis and duodenitis	Male	403671.8258	401919.508	405424.1435	1994
Middle SDI	Gastritis and duodenitis	Male	403781.3242	402028.8323	405533.8161	1995
Middle SDI	Gastritis and duodenitis	Male	402075.1252	400326.4179	403823.8325	1996

Middle SDI	Gastritis and duodenitis	Male	398162.0389	396421.9663	399902.1115	1997
Middle SDI	Gastritis and duodenitis	Male	397233.2629	395495.246	398971.2799	1998
Middle SDI	Gastritis and duodenitis	Male	398205.5775	396465.4198	399945.7352	1999
Middle SDI	Gastritis and duodenitis	Male	403522.3875	401770.5547	405274.2202	2000
Middle SDI	Gastritis and duodenitis	Male	408694.4405	406931.3207	410457.5603	2001
Middle SDI	Gastritis and duodenitis	Male	416549.9342	414769.787	418330.0814	2002
Middle SDI	Gastritis and duodenitis	Male	424493.7488	422696.528	426290.9696	2003
Middle SDI	Gastritis and duodenitis	Male	431338.5018	429526.6938	433150.3098	2004
Middle SDI	Gastritis and duodenitis	Male	433795.9958	431978.998	435612.9935	2005
Middle SDI	Gastritis and duodenitis	Male	433718.6891	431901.876	435535.5023	2006
Middle SDI	Gastritis and duodenitis	Male	430463.8284	428653.9255	432273.7313	2007
Middle SDI	Gastritis and duodenitis	Male	429898.1656	428089.4752	431706.8561	2008
Middle SDI	Gastritis and duodenitis	Male	429594.3172	427786.2806	431402.3538	2009
Middle SDI	Gastritis and duodenitis	Male	430049.1521	428240.1604	431858.1439	2010
Middle SDI	Gastritis and duodenitis	Male	432191.4876	430377.9612	434005.014	2011
Middle SDI	Gastritis and duodenitis	Male	435318.2583	433498.119	437138.3976	2012
Middle SDI	Gastritis and duodenitis	Male	438380.3801	436553.7892	440206.9709	2013
Middle SDI	Gastritis and duodenitis	Male	439512.3637	437683.3807	441341.3467	2014
Middle SDI	Gastritis and duodenitis	Male	442256.6108	440421.8285	444091.3931	2015
Middle SDI	Gastritis and duodenitis	Male	446961.3287	445116.6676	448805.9899	2016
Middle SDI	Gastritis and duodenitis	Male	449493.8022	447643.8531	451343.7513	2017
Middle SDI	Gastritis and duodenitis	Male	445000.349	443159.3242	446841.3739	2018
Middle SDI	Gastritis and duodenitis	Male	442675.4498	440835.1136	444515.786	2019
Middle SDI	Gastritis and duodenitis	Male	581715.9649	561711.5073	601720.4225	2020
Middle SDI	Gastritis and duodenitis	Male	582030.9441	554090.3307	609971.5575	2021
Middle SDI	Gastritis and duodenitis	Male	581811.3776	543533.8196	620088.9357	2022
Middle SDI	Gastritis and duodenitis	Male	581339.2732	530930.2676	631748.2788	2023
Middle SDI	Gastritis and duodenitis	Male	580944.978	516917.6729	644972.283	2024
Middle SDI	Gastritis and duodenitis	Male	580506.1898	501580.2945	659432.0851	2025
Middle SDI	Gastritis and duodenitis	Male	579805.2861	484904.0868	674706.4855	2026
Middle SDI	Gastritis and duodenitis	Male	578625.1784	466846.6703	690403.6864	2027
Middle SDI	Gastritis and duodenitis	Male	577171.6179	447684.17	706659.0659	2028
Middle SDI	Gastritis and duodenitis	Male	575707.2014	427685.3812	723729.0216	2029
Middle SDI	Gastritis and duodenitis	Male	574145.7747	406831.1822	741460.3672	2030
Middle SDI	Gastritis and duodenitis	Female	378115.1941	376415.2446	379815.1436	1990
Middle SDI	Gastritis and duodenitis	Female	378104.7016	376409.9006	379799.5026	1991
Middle SDI	Gastritis and duodenitis	Female	378674.5956	376978.975	380370.2162	1992
Middle SDI	Gastritis and duodenitis	Female	376937.8968	375246.2689	378629.5246	1993
Middle SDI	Gastritis and duodenitis	Female	378485.0199	376789.984	380180.0558	1994
Middle SDI	Gastritis and duodenitis	Female	378623.5961	376928.3142	380318.878	1995
Middle SDI	Gastritis and duodenitis	Female	378256.1025	376561.6916	379950.5133	1996
Middle SDI	Gastritis and duodenitis	Female	380094.734	378396.1801	381793.2879	1997
Middle SDI	Gastritis and duodenitis	Female	382479.5095	380775.5764	384183.4425	1998
Middle SDI	Gastritis and duodenitis	Female	386677.5929	384964.2433	388390.9425	1999
Middle SDI	Gastritis and duodenitis	Female	394543.0733	392812.205	396273.9416	2000
Middle SDI	Gastritis and duodenitis	Female	401376.1387	399630.186	403122.0914	2001
Middle SDI	Gastritis and duodenitis	Female	410626.4931	408860.3072	412392.6791	2002
Middle SDI	Gastritis and duodenitis	Female	420915.4822	419127.032	422703.9323	2003
Middle SDI	Gastritis and duodenitis	Female	429414.7292	427608.0834	431221.375	2004
Middle SDI	Gastritis and duodenitis	Female	435036.3851	433217.8331	436854.9372	2005
Middle SDI	Gastritis and duodenitis	Female	438077.8054	436252.8792	439902.7315	2006
Middle SDI	Gastritis and duodenitis	Female	437673.7625	435849.7357	439497.7892	2007
Middle SDI	Gastritis and duodenitis	Female	439218.8606	437391.6114	441046.1099	2008
Middle SDI	Gastritis and duodenitis	Female	441412.9146	439581.0751	443244.7541	2009
Middle SDI	Gastritis and duodenitis	Female	443926.6189	442089.5229	445763.7149	2010
Middle SDI	Gastritis and duodenitis	Female	447051.6957	445208.0786	448895.3128	2011
Middle SDI	Gastritis and duodenitis	Female	450218.9551	448368.7521	452069.1581	2012
Middle SDI	Gastritis and duodenitis	Female	454721.0554	452861.5318	456580.579	2013
Middle SDI	Gastritis and duodenitis	Female	459155.4093	457286.7338	461024.0848	2014
Middle SDI	Gastritis and duodenitis	Female	464563.0335	462683.2301	466442.8369	2015
Middle SDI	Gastritis and duodenitis	Female	473394.3017	471496.4757	475292.1278	2016
Middle SDI	Gastritis and duodenitis	Female	482562.1538	480645.8395	484478.4682	2017

Middle SDI	Gastritis and duodenitis	Female	486632.9914	484708.1478	488557.835	2018
Middle SDI	Gastritis and duodenitis	Female	489479.1731	487543.9851	491414.3611	2019
Middle SDI	Gastritis and duodenitis	Female	638220.0991	617479.3099	658960.8883	2020
Middle SDI	Gastritis and duodenitis	Female	645691.3883	615671.1602	675711.6164	2021
Middle SDI	Gastritis and duodenitis	Female	652635.7762	610442.3932	694829.1592	2022
Middle SDI	Gastritis and duodenitis	Female	659306.4877	602651.5347	715961.4408	2023
Middle SDI	Gastritis and duodenitis	Female	666076.5633	592947.7494	739205.3771	2024
Middle SDI	Gastritis and duodenitis	Female	672804.6698	581364.5553	764244.7842	2025
Middle SDI	Gastritis and duodenitis	Female	679286.3431	567869.0873	790703.5989	2026
Middle SDI	Gastritis and duodenitis	Female	685357.6822	552433.2464	818282.118	2027
Middle SDI	Gastritis and duodenitis	Female	691245.4243	535317.85	847172.9986	2028
Middle SDI	Gastritis and duodenitis	Female	697216.1794	516760.7049	877671.6538	2029
Middle SDI	Gastritis and duodenitis	Female	703128.6498	496667.9234	909589.3762	2030
Middle SDI	Gastritis and duodenitis	Both	786896.1334	783427.9302	790364.3366	1990
Middle SDI	Gastritis and duodenitis	Both	787088.2205	783628.904	790547.5371	1991
Middle SDI	Gastritis and duodenitis	Both	785934.1769	782478.2612	789390.0926	1992
Middle SDI	Gastritis and duodenitis	Both	781893.4576	778446.6188	785340.2964	1993
Middle SDI	Gastritis and duodenitis	Both	782156.8457	778709.492	785604.1993	1994
Middle SDI	Gastritis and duodenitis	Both	782404.9203	778957.1465	785852.694	1995
Middle SDI	Gastritis and duodenitis	Both	780331.2277	776888.1095	783774.3458	1996
Middle SDI	Gastritis and duodenitis	Both	778256.7729	774818.1464	781695.3995	1997
Middle SDI	Gastritis and duodenitis	Both	779712.7724	776270.8223	783154.7224	1998
Middle SDI	Gastritis and duodenitis	Both	784883.1705	781429.6632	788336.6777	1999
Middle SDI	Gastritis and duodenitis	Both	798065.4608	794582.7597	801548.1618	2000
Middle SDI	Gastritis and duodenitis	Both	810070.5792	806561.5067	813579.6517	2001
Middle SDI	Gastritis and duodenitis	Both	827176.4274	823630.0942	830722.7605	2002
Middle SDI	Gastritis and duodenitis	Both	845409.2309	841823.56	848994.9019	2003
Middle SDI	Gastritis and duodenitis	Both	860753.231	857134.7772	864371.6848	2004
Middle SDI	Gastritis and duodenitis	Both	868832.3809	865196.8311	872467.9307	2005
Middle SDI	Gastritis and duodenitis	Both	871796.4945	868154.7551	875438.2338	2006
Middle SDI	Gastritis and duodenitis	Both	868137.5909	864503.6613	871771.5205	2007
Middle SDI	Gastritis and duodenitis	Both	869117.0263	865481.0866	872752.966	2008
Middle SDI	Gastritis and duodenitis	Both	871007.2318	867367.3557	874647.1078	2009
Middle SDI	Gastritis and duodenitis	Both	873975.771	870329.6833	877621.8588	2010
Middle SDI	Gastritis and duodenitis	Both	879243.1833	875586.0398	882900.3268	2011
Middle SDI	Gastritis and duodenitis	Both	885537.2134	881866.8712	889207.5557	2012
Middle SDI	Gastritis and duodenitis	Both	893101.4355	889415.321	896787.5499	2013
Middle SDI	Gastritis and duodenitis	Both	898667.773	894970.1144	902365.4315	2014
Middle SDI	Gastritis and duodenitis	Both	906819.6443	903105.0586	910534.23	2015
Middle SDI	Gastritis and duodenitis	Both	920355.6305	916613.1433	924098.1177	2016
Middle SDI	Gastritis and duodenitis	Both	932055.956	928289.6925	935822.2195	2017
Middle SDI	Gastritis and duodenitis	Both	931633.3404	927867.4719	935399.2089	2018
Middle SDI	Gastritis and duodenitis	Both	932154.623	928379.0988	935930.1471	2019
Middle SDI	Gastritis and duodenitis	Both	1219936.064	1179190.817	1260681.311	2020
Middle SDI	Gastritis and duodenitis	Both	1227722.332	1169761.491	1285683.174	2021
Middle SDI	Gastritis and duodenitis	Both	1234447.154	1153976.213	1314918.095	2022
Middle SDI	Gastritis and duodenitis	Both	1240645.761	1133581.802	1347709.72	2023
Middle SDI	Gastritis and duodenitis	Both	1247021.541	1109865.422	1384177.66	2024
Middle SDI	Gastritis and duodenitis	Both	1253310.86	1082944.85	1423676.869	2025
Middle SDI	Gastritis and duodenitis	Both	1259091.629	1052773.174	1465410.084	2026
Middle SDI	Gastritis and duodenitis	Both	1263982.861	1019279.917	1508685.804	2027
Middle SDI	Gastritis and duodenitis	Both	1268417.042	983002.02	1553832.064	2028
Middle SDI	Gastritis and duodenitis	Both	1272923.381	944446.0862	1601400.675	2029
Middle SDI	Gastritis and duodenitis	Both	1277274.425	903499.1056	1651049.743	2030
Low-middle SDI	Gastritis and duodenitis	Male	263071.7923	261655.3	264488.2846	1990
Low-middle SDI	Gastritis and duodenitis	Male	265943.2903	264522.9433	267363.6374	1991
Low-middle SDI	Gastritis and duodenitis	Male	267796.0416	266371.4739	269220.6094	1992
Low-middle SDI	Gastritis and duodenitis	Male	268632.6245	267205.907	270059.342	1993
Low-middle SDI	Gastritis and duodenitis	Male	269222.9092	267794.6569	270651.1615	1994
Low-middle SDI	Gastritis and duodenitis	Male	269606.8889	268177.6627	271036.1151	1995
Low-middle SDI	Gastritis and duodenitis	Male	270096.605	268666.1157	271527.0942	1996
Low-middle SDI	Gastritis and duodenitis	Male	272236.6064	270800.4225	273672.7903	1997

Low-middle SDI	Gastritis and duodenitis	Male	273370.5673	271931.3779	274809.7567	1998
Low-middle SDI	Gastritis and duodenitis	Male	274167.8211	272726.5131	275609.129	1999
Low-middle SDI	Gastritis and duodenitis	Male	275572.3515	274127.3236	277017.3795	2000
Low-middle SDI	Gastritis and duodenitis	Male	277018.2016	275569.3492	278467.054	2001
Low-middle SDI	Gastritis and duodenitis	Male	278496.2354	277043.4769	279948.9939	2002
Low-middle SDI	Gastritis and duodenitis	Male	279054.5727	277600.3479	280508.7974	2003
Low-middle SDI	Gastritis and duodenitis	Male	280174.5894	278717.4209	281631.758	2004
Low-middle SDI	Gastritis and duodenitis	Male	282068.3602	280606.2093	283530.5111	2005
Low-middle SDI	Gastritis and duodenitis	Male	284733.8844	283264.771	286202.9977	2006
Low-middle SDI	Gastritis and duodenitis	Male	287206.3212	285730.7884	288681.8541	2007
Low-middle SDI	Gastritis and duodenitis	Male	292121.1967	290632.9398	293609.4537	2008
Low-middle SDI	Gastritis and duodenitis	Male	295021.4932	293525.7693	296517.2172	2009
Low-middle SDI	Gastritis and duodenitis	Male	297479.4174	295977.4017	298981.433	2010
Low-middle SDI	Gastritis and duodenitis	Male	300298.9586	298789.7629	301808.1542	2011
Low-middle SDI	Gastritis and duodenitis	Male	302549.6503	301034.7455	304064.5551	2012
Low-middle SDI	Gastritis and duodenitis	Male	305903.1927	304379.8165	307426.569	2013
Low-middle SDI	Gastritis and duodenitis	Male	308524.9991	306995.0094	310054.9889	2014
Low-middle SDI	Gastritis and duodenitis	Male	312955.4204	311414.32	314496.5209	2015
Low-middle SDI	Gastritis and duodenitis	Male	321064.6544	319503.4592	322625.8497	2016
Low-middle SDI	Gastritis and duodenitis	Male	328564.9143	326985.3142	330144.5144	2017
Low-middle SDI	Gastritis and duodenitis	Male	328635.1435	327054.6942	330215.5928	2018
Low-middle SDI	Gastritis and duodenitis	Male	328495.1797	326911.3492	330079.0102	2019
Low-middle SDI	Gastritis and duodenitis	Male	423064.8243	409261.3087	436868.3399	2020
Low-middle SDI	Gastritis and duodenitis	Male	427650.8529	409376.0667	445925.6391	2021
Low-middle SDI	Gastritis and duodenitis	Male	432198.5323	408012.8781	456384.1865	2022
Low-middle SDI	Gastritis and duodenitis	Male	436758.7709	405478.7102	468038.8316	2023
Low-middle SDI	Gastritis and duodenitis	Male	441419.6893	402010.167	480829.2117	2024
Low-middle SDI	Gastritis and duodenitis	Male	446146.1544	397666.9515	494625.3574	2025
Low-middle SDI	Gastritis and duodenitis	Male	450873.5357	392478.0326	509269.0388	2026
Low-middle SDI	Gastritis and duodenitis	Male	455530.2001	386433.6466	524626.7537	2027
Low-middle SDI	Gastritis and duodenitis	Male	460164.1555	379602.0476	540726.2634	2028
Low-middle SDI	Gastritis and duodenitis	Male	464858.7758	372057.308	557660.2435	2029
Low-middle SDI	Gastritis and duodenitis	Male	469581.3499	363771.924	575390.7758	2030
Low-middle SDI	Gastritis and duodenitis	Female	242415.5347	241056.5399	243774.5295	1990
Low-middle SDI	Gastritis and duodenitis	Female	244090.7284	242731.4868	245449.9701	1991
Low-middle SDI	Gastritis and duodenitis	Female	246416.1388	245051.1863	247781.0913	1992
Low-middle SDI	Gastritis and duodenitis	Female	247898.1992	246529.1832	249267.2151	1993
Low-middle SDI	Gastritis and duodenitis	Female	250407.3561	249031.3777	251783.3345	1994
Low-middle SDI	Gastritis and duodenitis	Female	252640.1116	251257.9844	254022.2387	1995
Low-middle SDI	Gastritis and duodenitis	Female	255431.1526	254041.3533	256820.9519	1996
Low-middle SDI	Gastritis and duodenitis	Female	259897.3427	258495.2922	261299.3931	1997
Low-middle SDI	Gastritis and duodenitis	Female	262655.1313	261245.5752	264064.6874	1998
Low-middle SDI	Gastritis and duodenitis	Female	265561.2122	264143.7851	266978.6393	1999
Low-middle SDI	Gastritis and duodenitis	Female	269657.3463	268228.884	271085.8085	2000
Low-middle SDI	Gastritis and duodenitis	Female	273171.8631	271733.9951	274609.7312	2001
Low-middle SDI	Gastritis and duodenitis	Female	276551.4435	275104.5749	277998.3122	2002
Low-middle SDI	Gastritis and duodenitis	Female	279710.8002	278255.5602	281166.0401	2003
Low-middle SDI	Gastritis and duodenitis	Female	283144.1449	281679.8639	284608.4259	2004
Low-middle SDI	Gastritis and duodenitis	Female	287756.12	286279.7881	289232.4519	2005
Low-middle SDI	Gastritis and duodenitis	Female	293505.1603	292013.9964	294996.3242	2006
Low-middle SDI	Gastritis and duodenitis	Female	300986.0756	299475.8326	302496.3186	2007
Low-middle SDI	Gastritis and duodenitis	Female	310830.6872	309295.6458	312365.7286	2008
Low-middle SDI	Gastritis and duodenitis	Female	319531.6399	317974.9753	321088.3044	2009
Low-middle SDI	Gastritis and duodenitis	Female	327465.0017	325888.881	329041.1224	2010
Low-middle SDI	Gastritis and duodenitis	Female	333089.8862	331500.1374	334679.635	2011
Low-middle SDI	Gastritis and duodenitis	Female	336462.8322	334864.9655	338060.699	2012
Low-middle SDI	Gastritis and duodenitis	Female	342060.1268	340448.8685	343671.385	2013
Low-middle SDI	Gastritis and duodenitis	Female	346913.0459	345290.2486	348535.8433	2014
Low-middle SDI	Gastritis and duodenitis	Female	354178.1609	352538.2339	355818.088	2015
Low-middle SDI	Gastritis and duodenitis	Female	364472.0893	362808.2097	366135.969	2016
Low-middle SDI	Gastritis and duodenitis	Female	375010.1338	373322.0568	376698.2109	2017
Low-middle SDI	Gastritis and duodenitis	Female	378811.3484	377113.9906	380508.7063	2018

Low-middle SDI	Gastritis and duodenitis	Female	380945.1229	379239.1234	382651.1223	2019
Low-middle SDI	Gastritis and duodenitis	Female	485856.9307	470591.4335	501122.4279	2020
Low-middle SDI	Gastritis and duodenitis	Female	493287.4492	472744.4671	513830.4314	2021
Low-middle SDI	Gastritis and duodenitis	Female	500559.9664	473027.9764	528091.9564	2022
Low-middle SDI	Gastritis and duodenitis	Female	507799.7531	471857.6378	543741.8685	2023
Low-middle SDI	Gastritis and duodenitis	Female	515128.9132	469518.5676	560739.2589	2024
Low-middle SDI	Gastritis and duodenitis	Female	522487.8387	466053.2451	578922.4324	2025
Low-middle SDI	Gastritis and duodenitis	Female	529779.8467	461457.5883	598102.1051	2026
Low-middle SDI	Gastritis and duodenitis	Female	536944.4161	455731.0162	618157.8159	2027
Low-middle SDI	Gastritis and duodenitis	Female	544089.2695	448993.1236	639185.4154	2028
Low-middle SDI	Gastritis and duodenitis	Female	551307.3804	441325.3754	661289.3855	2029
Low-middle SDI	Gastritis and duodenitis	Female	558521.38	432663.3728	684379.3872	2030
Low-middle SDI	Gastritis and duodenitis	Both	505487.327	502711.84	508262.8141	1990
Low-middle SDI	Gastritis and duodenitis	Both	510034.0188	507254.4301	512813.6074	1991
Low-middle SDI	Gastritis and duodenitis	Both	514212.1804	511422.6602	517001.7007	1992
Low-middle SDI	Gastritis and duodenitis	Both	516530.8237	513735.0901	519326.5572	1993
Low-middle SDI	Gastritis and duodenitis	Both	519630.2653	516826.0346	522434.4959	1994
Low-middle SDI	Gastritis and duodenitis	Both	522247.0005	519435.6472	525058.3538	1995
Low-middle SDI	Gastritis and duodenitis	Both	525527.7576	522707.469	528348.0462	1996
Low-middle SDI	Gastritis and duodenitis	Both	532133.949	529295.7147	534972.1834	1997
Low-middle SDI	Gastritis and duodenitis	Both	536025.6986	533176.9531	538874.4442	1998
Low-middle SDI	Gastritis and duodenitis	Both	539729.0332	536870.2982	542587.7683	1999
Low-middle SDI	Gastritis and duodenitis	Both	545229.6978	542356.2076	548103.1879	2000
Low-middle SDI	Gastritis and duodenitis	Both	550190.0647	547303.3443	553076.7851	2001
Low-middle SDI	Gastritis and duodenitis	Both	555047.679	552148.0518	557947.3061	2002
Low-middle SDI	Gastritis and duodenitis	Both	558765.3728	555855.9081	561674.8376	2003
Low-middle SDI	Gastritis and duodenitis	Both	563318.7343	560397.2848	566240.1838	2004
Low-middle SDI	Gastritis and duodenitis	Both	569824.4802	566885.9974	572762.963	2005
Low-middle SDI	Gastritis and duodenitis	Both	578239.0447	575278.7674	581199.322	2006
Low-middle SDI	Gastritis and duodenitis	Both	588192.3968	585206.6209	591178.1727	2007
Low-middle SDI	Gastritis and duodenitis	Both	602951.8839	599928.5856	605975.1822	2008
Low-middle SDI	Gastritis and duodenitis	Both	614553.1331	611500.7446	617605.5216	2009
Low-middle SDI	Gastritis and duodenitis	Both	624944.4191	621866.2827	628022.5554	2010
Low-middle SDI	Gastritis and duodenitis	Both	633388.8447	630289.9003	636487.7892	2011
Low-middle SDI	Gastritis and duodenitis	Both	639012.4826	635899.711	642125.2541	2012
Low-middle SDI	Gastritis and duodenitis	Both	647963.3195	644828.685	651097.954	2013
Low-middle SDI	Gastritis and duodenitis	Both	655438.0451	652285.2579	658590.8323	2014
Low-middle SDI	Gastritis and duodenitis	Both	667133.5814	663952.5539	670314.6088	2015
Low-middle SDI	Gastritis and duodenitis	Both	685536.7437	682311.6688	688761.8186	2016
Low-middle SDI	Gastritis and duodenitis	Both	703575.0481	700307.371	706842.7253	2017
Low-middle SDI	Gastritis and duodenitis	Both	707446.492	704168.6848	710724.2991	2018
Low-middle SDI	Gastritis and duodenitis	Both	709440.3026	706150.4727	712730.1325	2019
Low-middle SDI	Gastritis and duodenitis	Both	908921.755	879852.7422	937990.7678	2020
Low-middle SDI	Gastritis and duodenitis	Both	920938.3021	882120.5337	959756.0705	2021
Low-middle SDI	Gastritis and duodenitis	Both	932758.4986	881040.8545	984476.1428	2022
Low-middle SDI	Gastritis and duodenitis	Both	944558.524	877336.348	1011780.7	2023
Low-middle SDI	Gastritis and duodenitis	Both	956548.6025	871528.7345	1041568.471	2024
Low-middle SDI	Gastritis and duodenitis	Both	968633.9932	863720.1965	1073547.79	2025
Low-middle SDI	Gastritis and duodenitis	Both	980653.3823	853935.6208	1107371.144	2026
Low-middle SDI	Gastritis and duodenitis	Both	992474.6162	842164.6628	1142784.57	2027
Low-middle SDI	Gastritis and duodenitis	Both	1004253.425	828595.1711	1179911.679	2028
Low-middle SDI	Gastritis and duodenitis	Both	1016166.156	813382.6834	1218949.629	2029
Low-middle SDI	Gastritis and duodenitis	Both	1028102.73	796435.2967	1259770.163	2030
Low SDI	Gastritis and duodenitis	Male	165550.159	164436.2005	166664.1175	1990
Low SDI	Gastritis and duodenitis	Male	168481.0068	167376.1399	169585.8736	1991
Low SDI	Gastritis and duodenitis	Male	171366.5603	170252.9226	172480.1981	1992
Low SDI	Gastritis and duodenitis	Male	174133.3096	173010.5419	175256.0772	1993
Low SDI	Gastritis and duodenitis	Male	177045.9204	175913.7399	178178.1009	1994
Low SDI	Gastritis and duodenitis	Male	179701.4745	178560.6288	180842.3202	1995
Low SDI	Gastritis and duodenitis	Male	182996.9382	181845.2541	184148.6222	1996
Low SDI	Gastritis and duodenitis	Male	185824.0224	184663.0681	186984.9767	1997
Low SDI	Gastritis and duodenitis	Male	189108.6117	187936.889	190280.3345	1998

Low SDI	Gastritis and duodenitis	Male	191950.1521	190769.1323	193131.172	1999
Low SDI	Gastritis and duodenitis	Male	193644.0888	192457.6859	194830.4918	2000
Low SDI	Gastritis and duodenitis	Male	194089.9641	192902.2824	195277.6458	2001
Low SDI	Gastritis and duodenitis	Male	195117.487	193926.6089	196308.3652	2002
Low SDI	Gastritis and duodenitis	Male	196753.2571	195557.1605	197949.3537	2003
Low SDI	Gastritis and duodenitis	Male	198327.3101	197126.2355	199528.3848	2004
Low SDI	Gastritis and duodenitis	Male	200023.0002	198816.5317	201229.4687	2005
Low SDI	Gastritis and duodenitis	Male	204110.8354	202891.5283	205330.1426	2006
Low SDI	Gastritis and duodenitis	Male	208851.5241	207617.5048	210085.5435	2007
Low SDI	Gastritis and duodenitis	Male	212167.7183	210923.4866	213411.95	2008
Low SDI	Gastritis and duodenitis	Male	215927.4196	214671.7335	217183.1056	2009
Low SDI	Gastritis and duodenitis	Male	217740.8357	216479.6876	219001.9838	2010
Low SDI	Gastritis and duodenitis	Male	220852.8659	219582.3461	222123.3857	2011
Low SDI	Gastritis and duodenitis	Male	224388.1414	223107.1045	225669.1783	2012
Low SDI	Gastritis and duodenitis	Male	228835.9482	227541.8295	230130.0669	2013
Low SDI	Gastritis and duodenitis	Male	232809.6806	231503.9521	234115.4091	2014
Low SDI	Gastritis and duodenitis	Male	237497.162	236177.731	238816.5929	2015
Low SDI	Gastritis and duodenitis	Male	244173.3369	242834.4393	245512.2345	2016
Low SDI	Gastritis and duodenitis	Male	249971.3394	248615.7987	251326.88	2017
Low SDI	Gastritis and duodenitis	Male	253471.9318	252105.8955	254837.9681	2018
Low SDI	Gastritis and duodenitis	Male	256556.8413	255164.9961	257948.6866	2019
Low SDI	Gastritis and duodenitis	Male	220166.7144	215377.6714	224955.7573	2020
Low SDI	Gastritis and duodenitis	Male	224370.5983	216926.3521	231814.8444	2021
Low SDI	Gastritis and duodenitis	Male	228594.6363	217669.4519	239519.8208	2022
Low SDI	Gastritis and duodenitis	Male	232894.5904	217810.7117	247978.4692	2023
Low SDI	Gastritis and duodenitis	Male	237293.5406	217428.5832	257158.4981	2024
Low SDI	Gastritis and duodenitis	Male	241773.4115	216524.1799	267022.6432	2025
Low SDI	Gastritis and duodenitis	Male	246336.9517	215122.4219	277551.4815	2026
Low SDI	Gastritis and duodenitis	Male	250931.9758	213188.3977	288675.554	2027
Low SDI	Gastritis and duodenitis	Male	255564.5792	210729.1701	300399.9883	2028
Low SDI	Gastritis and duodenitis	Male	260251.3679	207749.2932	312753.4426	2029
Low SDI	Gastritis and duodenitis	Male	264982.376	204226.1022	325738.6498	2030
Low SDI	Gastritis and duodenitis	Female	105754.2987	104868.0959	106640.5015	1990
Low SDI	Gastritis and duodenitis	Female	107538.9882	106661.4448	108416.5316	1991
Low SDI	Gastritis and duodenitis	Female	109412.0487	108528.225	110295.8723	1992
Low SDI	Gastritis and duodenitis	Female	111060.5545	110169.8457	111951.2633	1993
Low SDI	Gastritis and duodenitis	Female	112962.4339	112063.9402	113860.9275	1994
Low SDI	Gastritis and duodenitis	Female	115148.7658	114241.3242	116056.2073	1995
Low SDI	Gastritis and duodenitis	Female	117741.3819	116823.2996	118659.4641	1996
Low SDI	Gastritis and duodenitis	Female	120154.7965	119226.8497	121082.7433	1997
Low SDI	Gastritis and duodenitis	Female	122761.8591	121823.3129	123700.4053	1998
Low SDI	Gastritis and duodenitis	Female	125057.7166	124109.9218	126005.5114	1999
Low SDI	Gastritis and duodenitis	Female	126851.6239	125896.7098	127806.538	2000
Low SDI	Gastritis and duodenitis	Female	128690.5976	127728.4529	129652.7424	2001
Low SDI	Gastritis and duodenitis	Female	130657.7901	129687.9373	131627.6429	2002
Low SDI	Gastritis and duodenitis	Female	132793.921	131815.743	133772.099	2003
Low SDI	Gastritis and duodenitis	Female	135038.6943	134051.8331	136025.5555	2004
Low SDI	Gastritis and duodenitis	Female	137664.6453	136667.7166	138661.5741	2005
Low SDI	Gastritis and duodenitis	Female	140756.291	139747.7117	141764.8702	2006
Low SDI	Gastritis and duodenitis	Female	144836.8802	143813.1135	145860.6469	2007
Low SDI	Gastritis and duodenitis	Female	149707.8973	148666.1691	150749.6255	2008
Low SDI	Gastritis and duodenitis	Female	154412.466	153353.6084	155471.3236	2009
Low SDI	Gastritis and duodenitis	Female	158630.2724	157556.3266	159704.2182	2010
Low SDI	Gastritis and duodenitis	Female	162355.524	161268.4928	163442.5552	2011
Low SDI	Gastritis and duodenitis	Female	166066.5439	164966.6129	167166.4749	2012
Low SDI	Gastritis and duodenitis	Female	169851.7841	168738.8114	170964.7568	2013
Low SDI	Gastritis and duodenitis	Female	174192.5346	173064.7362	175320.333	2014
Low SDI	Gastritis and duodenitis	Female	178509.3901	177366.9582	179651.822	2015
Low SDI	Gastritis and duodenitis	Female	184571.6126	183408.9345	185734.2908	2016
Low SDI	Gastritis and duodenitis	Female	191281.3854	190096.7823	192465.9886	2017
Low SDI	Gastritis and duodenitis	Female	196512.5983	195309.9916	197715.205	2018
Low SDI	Gastritis and duodenitis	Female	201329.5632	200098.0105	202561.1159	2019

Low SDI	Gastritis and duodenitis	Female	174360.4849	170394.9908	178325.9789	2020
Low SDI	Gastritis and duodenitis	Female	179587.6612	173703.4964	185471.826	2021
Low SDI	Gastritis and duodenitis	Female	184895.5865	176450.6634	193340.5097	2022
Low SDI	Gastritis and duodenitis	Female	190309.3891	178747.6544	201871.1239	2023
Low SDI	Gastritis and duodenitis	Female	195857.0694	180653.5096	211060.6291	2024
Low SDI	Gastritis and duodenitis	Female	201533.1126	182170.0992	220896.1259	2025
Low SDI	Gastritis and duodenitis	Female	207310.7898	183278.0778	231343.5019	2026
Low SDI	Gastritis and duodenitis	Female	213149.4739	183938.435	242360.5128	2027
Low SDI	Gastritis and duodenitis	Female	219073.5451	184163.4559	253983.6344	2028
Low SDI	Gastritis and duodenitis	Female	225110.7857	183960.6404	266260.931	2029
Low SDI	Gastritis and duodenitis	Female	231247.3283	183299.6492	279195.0074	2030
Low SDI	Gastritis and duodenitis	Both	271304.4577	269304.2965	273304.619	1990
Low SDI	Gastritis and duodenitis	Both	276019.995	274037.5847	278002.4052	1991
Low SDI	Gastritis and duodenitis	Both	280778.609	278781.1476	282776.0704	1992
Low SDI	Gastritis and duodenitis	Both	285193.8641	283180.3876	287207.3406	1993
Low SDI	Gastritis and duodenitis	Both	290008.3543	287977.6802	292039.0285	1994
Low SDI	Gastritis and duodenitis	Both	294850.2403	292801.953	296898.5276	1995
Low SDI	Gastritis and duodenitis	Both	300738.32	298668.5537	302808.0863	1996
Low SDI	Gastritis and duodenitis	Both	305978.8189	303889.9178	308067.72	1997
Low SDI	Gastritis and duodenitis	Both	311870.4708	309760.2019	313980.7398	1998
Low SDI	Gastritis and duodenitis	Both	317007.8687	314879.054	319136.6834	1999
Low SDI	Gastritis and duodenitis	Both	320495.7127	318354.3957	322637.0298	2000
Low SDI	Gastritis and duodenitis	Both	322780.5617	320630.7353	324930.3882	2001
Low SDI	Gastritis and duodenitis	Both	325775.2771	323614.5462	327936.0081	2002
Low SDI	Gastritis and duodenitis	Both	329547.1781	327372.9035	331721.4527	2003
Low SDI	Gastritis and duodenitis	Both	333366.0044	331178.0686	335553.9402	2004
Low SDI	Gastritis and duodenitis	Both	337687.6455	335484.2483	339891.0428	2005
Low SDI	Gastritis and duodenitis	Both	344867.1264	342639.24	347095.0128	2006
Low SDI	Gastritis and duodenitis	Both	353688.4044	351430.6183	355946.1904	2007
Low SDI	Gastritis and duodenitis	Both	361875.6156	359589.6557	364161.5755	2008
Low SDI	Gastritis and duodenitis	Both	370339.8856	368025.3419	372654.4293	2009
Low SDI	Gastritis and duodenitis	Both	376371.1081	374036.0143	378706.202	2010
Low SDI	Gastritis and duodenitis	Both	383208.3899	380850.8389	385565.941	2011
Low SDI	Gastritis and duodenitis	Both	390454.6853	388073.7175	392835.6532	2012
Low SDI	Gastritis and duodenitis	Both	398687.7323	396280.6409	401094.8237	2013
Low SDI	Gastritis and duodenitis	Both	407002.2152	404568.6882	409435.7421	2014
Low SDI	Gastritis and duodenitis	Both	416006.5521	413544.6893	418468.4149	2015
Low SDI	Gastritis and duodenitis	Both	428744.9495	426243.3738	431246.5252	2016
Low SDI	Gastritis and duodenitis	Both	441252.7248	438712.581	443792.8685	2017
Low SDI	Gastritis and duodenitis	Both	449984.5301	447415.8872	452553.1731	2018
Low SDI	Gastritis and duodenitis	Both	457886.4046	455263.0066	460509.8026	2019
Low SDI	Gastritis and duodenitis	Both	394527.1993	385772.6622	403281.7363	2020
Low SDI	Gastritis and duodenitis	Both	403958.2595	390629.8486	417286.6704	2021
Low SDI	Gastritis and duodenitis	Both	413490.2228	394120.1152	432860.3304	2022
Low SDI	Gastritis and duodenitis	Both	423203.9796	396558.3661	449849.5931	2023
Low SDI	Gastritis and duodenitis	Both	433150.61	398082.0928	468219.1273	2024
Low SDI	Gastritis and duodenitis	Both	443306.5241	398694.2791	487918.7691	2025
Low SDI	Gastritis and duodenitis	Both	453647.7415	398400.4997	508894.9834	2026
Low SDI	Gastritis and duodenitis	Both	464081.4498	397126.8326	531036.0669	2027
Low SDI	Gastritis and duodenitis	Both	474638.1243	394892.626	554383.6227	2028
Low SDI	Gastritis and duodenitis	Both	485362.1537	391709.9336	579014.3737	2029
Low SDI	Gastritis and duodenitis	Both	496229.7043	387525.7514	604933.6572	2030
Global	Gastroesophageal reflux disease	Male	1622855.585	1619350.203	1626360.968	1990
Global	Gastroesophageal reflux disease	Male	1654820.076	1651333.767	1658306.385	1991
Global	Gastroesophageal reflux disease	Male	1687430.897	1683909.392	1690952.403	1992
Global	Gastroesophageal reflux disease	Male	1720438.111	1716882.635	1723993.587	1993
Global	Gastroesophageal reflux disease	Male	1754256.585	1750665.419	1757847.751	1994
Global	Gastroesophageal reflux disease	Male	1789286.739	1785658.595	1792914.884	1995
Global	Gastroesophageal reflux disease	Male	1826478.021	1822811.033	1830145.01	1996
Global	Gastroesophageal reflux disease	Male	1865395.732	1861688.533	1869102.93	1997
Global	Gastroesophageal reflux disease	Male	1904916.741	1901169.12	1908664.363	1998
Global	Gastroesophageal reflux disease	Male	1944629.886	1940841.998	1948417.773	1999

Global	Gastroesophageal reflux disease	Male	1983970.178	1980142.832	1987797.525	2000
Global	Gastroesophageal reflux disease	Male	2022799.898	2018934.086	2026665.711	2001
Global	Gastroesophageal reflux disease	Male	2061218.451	2057314.978	2065121.924	2002
Global	Gastroesophageal reflux disease	Male	2099762.238	2095821.342	2103703.135	2003
Global	Gastroesophageal reflux disease	Male	2139105.862	2135127.067	2143084.658	2004
Global	Gastroesophageal reflux disease	Male	2179842.439	2175824.737	2183860.142	2005
Global	Gastroesophageal reflux disease	Male	2220012.517	2215956.813	2224068.22	2006
Global	Gastroesophageal reflux disease	Male	2258617.67	2254525.829	2262709.511	2007
Global	Gastroesophageal reflux disease	Male	2296801.385	2292674.106	2300928.664	2008
Global	Gastroesophageal reflux disease	Male	2336678.795	2332514.793	2340842.798	2009
Global	Gastroesophageal reflux disease	Male	2381022.675	2376818.21	2385227.14	2010
Global	Gastroesophageal reflux disease	Male	2430331.812	2426082.793	2434580.831	2011
Global	Gastroesophageal reflux disease	Male	2482107.63	2477812.317	2486402.944	2012
Global	Gastroesophageal reflux disease	Male	2534853.381	2530511.432	2539195.331	2013
Global	Gastroesophageal reflux disease	Male	2587701.821	2583313.629	2592090.014	2014
Global	Gastroesophageal reflux disease	Male	2640028.895	2635595.277	2644462.512	2015
Global	Gastroesophageal reflux disease	Male	2704043.548	2699554.893	2708532.202	2016
Global	Gastroesophageal reflux disease	Male	2767508.645	2762964.831	2772052.459	2017
Global	Gastroesophageal reflux disease	Male	2819200.483	2814613.176	2823787.789	2018
Global	Gastroesophageal reflux disease	Male	2872593.098	2867915.876	2877270.321	2019
Global	Gastroesophageal reflux disease	Male	2943650.453	2917236.046	2970064.86	2020
Global	Gastroesophageal reflux disease	Male	2997913.689	2949974.496	3045852.883	2021
Global	Gastroesophageal reflux disease	Male	3052092.643	2976687.183	3127498.104	2022
Global	Gastroesophageal reflux disease	Male	3106122.197	2998285.36	3213959.034	2023
Global	Gastroesophageal reflux disease	Male	3160397.897	3015423.025	3305372.769	2024
Global	Gastroesophageal reflux disease	Male	3214867.519	3027969.859	3401765.179	2025
Global	Gastroesophageal reflux disease	Male	3269546.379	3036254.411	3502838.347	2026
Global	Gastroesophageal reflux disease	Male	3323994.828	3040044.961	3607944.694	2027
Global	Gastroesophageal reflux disease	Male	3378121.54	3039352.613	3716890.466	2028
Global	Gastroesophageal reflux disease	Male	3432347.522	3034501.444	3830193.6	2029
Global	Gastroesophageal reflux disease	Male	3486539.682	3025144.275	3947935.089	2030
Global	Gastroesophageal reflux disease	Female	1779391.942	1775718.83	1783065.055	1990
Global	Gastroesophageal reflux disease	Female	1814854.237	1811197.302	1818511.173	1991
Global	Gastroesophageal reflux disease	Female	1850894.057	1847200.087	1854588.028	1992
Global	Gastroesophageal reflux disease	Female	1887347.416	1883617.548	1891077.284	1993
Global	Gastroesophageal reflux disease	Female	1924744.912	1920977.313	1928512.511	1994
Global	Gastroesophageal reflux disease	Female	1963261.462	1959455.036	1967067.887	1995
Global	Gastroesophageal reflux disease	Female	2004459.897	2000612.359	2008307.435	1996
Global	Gastroesophageal reflux disease	Female	2048495.92	2044604.938	2052386.902	1997
Global	Gastroesophageal reflux disease	Female	2093769.984	2089834.85	2097705.118	1998
Global	Gastroesophageal reflux disease	Female	2139390.735	2135411.561	2143369.908	1999
Global	Gastroesophageal reflux disease	Female	2184165.922	2180143.943	2188187.9	2000
Global	Gastroesophageal reflux disease	Female	2225463.734	2221402.761	2229524.707	2001
Global	Gastroesophageal reflux disease	Female	2263018.828	2258922.751	2267114.905	2002
Global	Gastroesophageal reflux disease	Female	2298938.976	2294809.555	2303068.398	2003
Global	Gastroesophageal reflux disease	Female	2336286.749	2332122.875	2340450.623	2004
Global	Gastroesophageal reflux disease	Female	2377437.123	2373235.636	2381638.61	2005
Global	Gastroesophageal reflux disease	Female	2420661.812	2416421.176	2424902.448	2006
Global	Gastroesophageal reflux disease	Female	2463083.956	2458805.297	2467362.616	2007
Global	Gastroesophageal reflux disease	Female	2506114	2501797.123	2510430.877	2008
Global	Gastroesophageal reflux disease	Female	2551558.085	2547201.154	2555915.017	2009
Global	Gastroesophageal reflux disease	Female	2602342.18	2597940.914	2606743.445	2010
Global	Gastroesophageal reflux disease	Female	2657752.901	2653303.765	2662202.037	2011
Global	Gastroesophageal reflux disease	Female	2715193.06	2710694.836	2719691.284	2012
Global	Gastroesophageal reflux disease	Female	2773614.745	2769067.155	2778162.336	2013
Global	Gastroesophageal reflux disease	Female	2831959.013	2827362.612	2836555.414	2014
Global	Gastroesophageal reflux disease	Female	2889767.391	2885122.976	2894411.805	2015
Global	Gastroesophageal reflux disease	Female	2965167.565	2960461.385	2969873.745	2016
Global	Gastroesophageal reflux disease	Female	3040288.531	3035520.175	3045056.887	2017
Global	Gastroesophageal reflux disease	Female	3097470.751	3092656.503	3102285	2018
Global	Gastroesophageal reflux disease	Female	3156046.893	3151142.023	3160951.762	2019
Global	Gastroesophageal reflux disease	Female	3234389.576	3205103.832	3263675.319	2020

Global	Gastroesophageal reflux disease	Female	3294153.627	3240308.394	3347998.861	2021
Global	Gastroesophageal reflux disease	Female	3353757.703	3268609.31	3438906.096	2022
Global	Gastroesophageal reflux disease	Female	3412855.24	3290763.705	3534946.774	2023
Global	Gastroesophageal reflux disease	Female	3471869.192	3307501.721	3636236.663	2024
Global	Gastroesophageal reflux disease	Female	3530936.896	3318885.187	3742988.606	2025
Global	Gastroesophageal reflux disease	Female	3590344.747	3325521.1	3855168.393	2026
Global	Gastroesophageal reflux disease	Female	3649489.595	3327043.626	3971935.563	2027
Global	Gastroesophageal reflux disease	Female	3708055.466	3323278.894	4092832.037	2028
Global	Gastroesophageal reflux disease	Female	3766449.682	3314552.4	4218346.963	2029
Global	Gastroesophageal reflux disease	Female	3824664.315	3300624.104	4348704.527	2030
Global	Gastroesophageal reflux disease	Both	3402247.527	3395069.032	3409426.022	1990
Global	Gastroesophageal reflux disease	Both	3469674.313	3462531.068	3476817.558	1991
Global	Gastroesophageal reflux disease	Both	3538324.955	3531109.479	3545540.43	1992
Global	Gastroesophageal reflux disease	Both	3607785.527	3600500.183	3615070.871	1993
Global	Gastroesophageal reflux disease	Both	3679001.497	3671642.732	3686360.262	1994
Global	Gastroesophageal reflux disease	Both	3752548.201	3745113.632	3759982.771	1995
Global	Gastroesophageal reflux disease	Both	3830937.918	3823423.392	3838452.445	1996
Global	Gastroesophageal reflux disease	Both	3913891.652	3906293.471	3921489.832	1997
Global	Gastroesophageal reflux disease	Both	3998686.726	3991003.97	4006369.481	1998
Global	Gastroesophageal reflux disease	Both	4084020.62	4076253.559	4091787.681	1999
Global	Gastroesophageal reflux disease	Both	4168136.1	4160286.775	4175985.425	2000
Global	Gastroesophageal reflux disease	Both	4248263.632	4240336.847	4256190.418	2001
Global	Gastroesophageal reflux disease	Both	4324237.279	4316237.729	4332236.829	2002
Global	Gastroesophageal reflux disease	Both	4398701.214	4390630.896	4406771.532	2003
Global	Gastroesophageal reflux disease	Both	4475392.611	4467249.942	4483535.281	2004
Global	Gastroesophageal reflux disease	Both	4557279.562	4549060.373	4565498.752	2005
Global	Gastroesophageal reflux disease	Both	4640674.329	4632377.988	4648970.669	2006
Global	Gastroesophageal reflux disease	Both	4721701.626	4713331.126	4730072.126	2007
Global	Gastroesophageal reflux disease	Both	4802915.385	4794471.229	4811359.541	2008
Global	Gastroesophageal reflux disease	Both	4888236.88	4879715.946	4896757.814	2009
Global	Gastroesophageal reflux disease	Both	4983364.855	4974759.124	4991970.585	2010
Global	Gastroesophageal reflux disease	Both	5088084.713	5079386.559	5096782.868	2011
Global	Gastroesophageal reflux disease	Both	5197300.69	5188507.153	5206094.227	2012
Global	Gastroesophageal reflux disease	Both	5308468.127	5299578.587	5317357.666	2013
Global	Gastroesophageal reflux disease	Both	5419660.834	5410676.241	5428645.428	2014
Global	Gastroesophageal reflux disease	Both	5529796.286	5520718.254	5538874.318	2015
Global	Gastroesophageal reflux disease	Both	5669211.112	5660016.278	5678405.947	2016
Global	Gastroesophageal reflux disease	Both	5807797.176	5798485.006	5817109.346	2017
Global	Gastroesophageal reflux disease	Both	5916671.234	5907269.679	5926072.789	2018
Global	Gastroesophageal reflux disease	Both	6028639.991	6019057.899	6038222.083	2019
Global	Gastroesophageal reflux disease	Both	6178040.029	6122339.878	6233740.179	2020
Global	Gastroesophageal reflux disease	Both	6292067.317	6190282.89	6393851.743	2021
Global	Gastroesophageal reflux disease	Both	6405850.346	6245296.493	6566404.199	2022
Global	Gastroesophageal reflux disease	Both	6518977.437	6289049.065	6748905.808	2023
Global	Gastroesophageal reflux disease	Both	6632267.089	6322924.746	6941609.432	2024
Global	Gastroesophageal reflux disease	Both	6745804.415	6346855.046	7144753.785	2025
Global	Gastroesophageal reflux disease	Both	6859891.126	6361775.512	7358006.739	2026
Global	Gastroesophageal reflux disease	Both	6973484.423	6367088.588	7579880.257	2027
Global	Gastroesophageal reflux disease	Both	7086177.005	6362631.507	7809722.503	2028
Global	Gastroesophageal reflux disease	Both	7198797.204	6349053.845	8048540.563	2029
Global	Gastroesophageal reflux disease	Both	7311203.997	6325768.379	8296639.615	2030
High SDI	Gastroesophageal reflux disease	Male	283379.7642	281933.401	284826.1275	1990
High SDI	Gastroesophageal reflux disease	Male	286624.6392	285225.0169	288024.2615	1991
High SDI	Gastroesophageal reflux disease	Male	289970.7549	288562.6915	291378.8183	1992
High SDI	Gastroesophageal reflux disease	Male	293306.4592	291891.0664	294721.852	1993
High SDI	Gastroesophageal reflux disease	Male	296718.0864	295294.4615	298141.7112	1994
High SDI	Gastroesophageal reflux disease	Male	300377.6163	298944.6231	301810.6095	1995
High SDI	Gastroesophageal reflux disease	Male	304191.7031	302748.9371	305634.4691	1996
High SDI	Gastroesophageal reflux disease	Male	308287.718	306834.527	309740.9091	1997
High SDI	Gastroesophageal reflux disease	Male	312471.9858	311008.212	313935.7596	1998
High SDI	Gastroesophageal reflux disease	Male	316600.4648	315126.3007	318074.6289	1999
High SDI	Gastroesophageal reflux disease	Male	320621.3175	319137.0772	322105.5579	2000

High SDI	Gastroesophageal reflux disease	Male	323892.5379	322400.2459	325384.83	2001
High SDI	Gastroesophageal reflux disease	Male	326173.0459	324675.2952	327670.7965	2002
High SDI	Gastroesophageal reflux disease	Male	327817.3721	326315.6978	329319.0465	2003
High SDI	Gastroesophageal reflux disease	Male	329312.7688	327807.4891	330818.0485	2004
High SDI	Gastroesophageal reflux disease	Male	331105.6622	329596.0488	332615.2756	2005
High SDI	Gastroesophageal reflux disease	Male	332930.5342	331416.4962	334444.5722	2006
High SDI	Gastroesophageal reflux disease	Male	334773.2509	333254.7916	336291.7103	2007
High SDI	Gastroesophageal reflux disease	Male	337138.2795	335614.0349	338662.5241	2008
High SDI	Gastroesophageal reflux disease	Male	340250.6302	338718.725	341782.5353	2009
High SDI	Gastroesophageal reflux disease	Male	344379.5152	342837.7145	345921.3159	2010
High SDI	Gastroesophageal reflux disease	Male	350149.4076	348593.9993	351704.8159	2011
High SDI	Gastroesophageal reflux disease	Male	357129.8985	355558.0561	358701.7409	2012
High SDI	Gastroesophageal reflux disease	Male	364440.6413	362851.5981	366029.6845	2013
High SDI	Gastroesophageal reflux disease	Male	371283.003	369677.9567	372888.0494	2014
High SDI	Gastroesophageal reflux disease	Male	376850.9788	375233.0935	378468.8641	2015
High SDI	Gastroesophageal reflux disease	Male	381508.3507	379879.2701	383137.4313	2016
High SDI	Gastroesophageal reflux disease	Male	386014.1606	384373.2056	387655.1156	2017
High SDI	Gastroesophageal reflux disease	Male	391520.2332	389866.5887	393173.8777	2018
High SDI	Gastroesophageal reflux disease	Male	398380.8975	396657.8493	400103.9457	2019
High SDI	Gastroesophageal reflux disease	Male	374335.5049	369540.1943	379130.8154	2020
High SDI	Gastroesophageal reflux disease	Male	379288.3218	370893.0355	387683.6081	2021
High SDI	Gastroesophageal reflux disease	Male	384177.3753	371201.3848	397153.3657	2022
High SDI	Gastroesophageal reflux disease	Male	389007.4257	370653.1729	407361.6785	2023
High SDI	Gastroesophageal reflux disease	Male	393777.0269	369318.7572	418235.2966	2024
High SDI	Gastroesophageal reflux disease	Male	398480.8355	367223.3416	429738.3294	2025
High SDI	Gastroesophageal reflux disease	Male	403135.2872	364424.7287	441845.8457	2026
High SDI	Gastroesophageal reflux disease	Male	407713.0127	360926.9444	454499.081	2027
High SDI	Gastroesophageal reflux disease	Male	412225.1956	356759.1949	467691.1962	2028
High SDI	Gastroesophageal reflux disease	Male	416658.8103	351917.8601	481399.7606	2029
High SDI	Gastroesophageal reflux disease	Male	420996.2317	346387.7093	495604.7541	2030
High SDI	Gastroesophageal reflux disease	Female	330472.3283	328902.3096	332042.3469	1990
High SDI	Gastroesophageal reflux disease	Female	333832.8562	332300.0414	335365.671	1991
High SDI	Gastroesophageal reflux disease	Female	337198.6801	335657.9477	338739.4124	1992
High SDI	Gastroesophageal reflux disease	Female	340512.858	338965.3071	342060.4089	1993
High SDI	Gastroesophageal reflux disease	Female	343850.2979	342295.2376	345405.3581	1994
High SDI	Gastroesophageal reflux disease	Female	347274.2546	345711.0648	348837.4443	1995
High SDI	Gastroesophageal reflux disease	Female	350657.9166	349086.6464	352229.1867	1996
High SDI	Gastroesophageal reflux disease	Female	354002.501	352423.2586	355581.7433	1997
High SDI	Gastroesophageal reflux disease	Female	357246.2181	355659.2329	358833.2032	1998
High SDI	Gastroesophageal reflux disease	Female	360475.1225	358880.4215	362069.8235	1999
High SDI	Gastroesophageal reflux disease	Female	363701.4398	362098.9601	365303.9195	2000
High SDI	Gastroesophageal reflux disease	Female	365628.1507	364021.1298	367235.1717	2001
High SDI	Gastroesophageal reflux disease	Female	365568.5659	363962.0058	367175.1261	2002
High SDI	Gastroesophageal reflux disease	Female	364398.0001	362794.4729	366001.5273	2003
High SDI	Gastroesophageal reflux disease	Female	363313.9679	361713.0675	364914.8683	2004
High SDI	Gastroesophageal reflux disease	Female	363331.2788	361730.421	364932.1366	2005
High SDI	Gastroesophageal reflux disease	Female	364123.3781	362520.6622	365726.0941	2006
High SDI	Gastroesophageal reflux disease	Female	365110.0891	363504.9845	366715.1937	2007
High SDI	Gastroesophageal reflux disease	Female	366739.3899	365130.3492	368348.4305	2008
High SDI	Gastroesophageal reflux disease	Female	369386.0732	367770.7464	371001.3999	2009
High SDI	Gastroesophageal reflux disease	Female	373675.7845	372050.5717	375300.9974	2010
High SDI	Gastroesophageal reflux disease	Female	379939.931	378300.5029	381579.3591	2011
High SDI	Gastroesophageal reflux disease	Female	387686.7614	386029.8243	389343.6984	2012
High SDI	Gastroesophageal reflux disease	Female	395820.7677	394145.4829	397496.0525	2013
High SDI	Gastroesophageal reflux disease	Female	403284.7166	401592.6849	404976.7482	2014
High SDI	Gastroesophageal reflux disease	Female	409125.5463	407420.5427	410830.55	2015
High SDI	Gastroesophageal reflux disease	Female	413740.2175	412024.5909	415455.844	2016
High SDI	Gastroesophageal reflux disease	Female	418135.2124	416408.6368	419861.7881	2017
High SDI	Gastroesophageal reflux disease	Female	423817.7524	422078.5243	425556.9805	2018
High SDI	Gastroesophageal reflux disease	Female	431040.5955	429241.6129	432839.5781	2019
High SDI	Gastroesophageal reflux disease	Female	410311.7083	404584.591	416038.8256	2020
High SDI	Gastroesophageal reflux disease	Female	415553.1864	405543.0997	425563.2731	2021

High SDI	Gastroesophageal reflux disease	Female	420735.7787	405274.8819	436196.6755	2022
High SDI	Gastroesophageal reflux disease	Female	425846.923	403987.8056	447706.0405	2023
High SDI	Gastroesophageal reflux disease	Female	430862.6607	401747.5171	459977.8043	2024
High SDI	Gastroesophageal reflux disease	Female	435790.1117	398600.6497	472979.5736	2025
High SDI	Gastroesophageal reflux disease	Female	440676.7318	394641.4309	486712.0327	2026
High SDI	Gastroesophageal reflux disease	Female	445491.4905	389875.7342	501107.2469	2027
High SDI	Gastroesophageal reflux disease	Female	450227.2191	384320.4778	516133.9604	2028
High SDI	Gastroesophageal reflux disease	Female	454846.3063	377955.0222	531737.5903	2029
High SDI	Gastroesophageal reflux disease	Female	459336.9742	370773.1558	547900.7926	2030
High SDI	Gastroesophageal reflux disease	Both	613852.0925	610835.7106	616868.4743	1990
High SDI	Gastroesophageal reflux disease	Both	620457.4954	617525.0583	623389.9325	1991
High SDI	Gastroesophageal reflux disease	Both	627169.4349	624220.6392	630118.2307	1992
High SDI	Gastroesophageal reflux disease	Both	633819.3172	630856.3735	636782.2609	1993
High SDI	Gastroesophageal reflux disease	Both	640568.3842	637589.6992	643547.0693	1994
High SDI	Gastroesophageal reflux disease	Both	647651.8709	644655.6879	650648.0538	1995
High SDI	Gastroesophageal reflux disease	Both	654849.6197	651835.5835	657863.6558	1996
High SDI	Gastroesophageal reflux disease	Both	662290.219	659257.7856	665322.6524	1997
High SDI	Gastroesophageal reflux disease	Both	669718.2039	666667.445	672768.9627	1998
High SDI	Gastroesophageal reflux disease	Both	677075.5873	674006.7221	680144.4524	1999
High SDI	Gastroesophageal reflux disease	Both	684322.7573	681236.0372	687409.4774	2000
High SDI	Gastroesophageal reflux disease	Both	689520.6886	686421.3756	692620.0017	2001
High SDI	Gastroesophageal reflux disease	Both	691741.6118	688637.301	694845.9226	2002
High SDI	Gastroesophageal reflux disease	Both	692215.3722	689110.1707	695320.5737	2003
High SDI	Gastroesophageal reflux disease	Both	692626.7367	689520.5566	695732.9168	2004
High SDI	Gastroesophageal reflux disease	Both	694436.941	691326.4698	697547.4122	2005
High SDI	Gastroesophageal reflux disease	Both	697053.9123	693937.1584	700170.6663	2006
High SDI	Gastroesophageal reflux disease	Both	699883.34	696759.7761	703006.904	2007
High SDI	Gastroesophageal reflux disease	Both	703877.6694	700744.3841	707010.9546	2008
High SDI	Gastroesophageal reflux disease	Both	709636.7033	706489.4714	712783.9352	2009
High SDI	Gastroesophageal reflux disease	Both	718055.2997	714888.2862	721222.3132	2010
High SDI	Gastroesophageal reflux disease	Both	730089.3386	726894.5022	733284.175	2011
High SDI	Gastroesophageal reflux disease	Both	744816.6599	741587.8804	748045.4393	2012
High SDI	Gastroesophageal reflux disease	Both	760261.409	756997.0811	763525.737	2013
High SDI	Gastroesophageal reflux disease	Both	774567.7196	771270.6416	777864.7976	2014
High SDI	Gastroesophageal reflux disease	Both	785976.5251	782653.6362	789299.4141	2015
High SDI	Gastroesophageal reflux disease	Both	795248.5681	791903.861	798593.2753	2016
High SDI	Gastroesophageal reflux disease	Both	804149.373	800781.8424	807516.9036	2017
High SDI	Gastroesophageal reflux disease	Both	815337.9856	811945.1129	818730.8582	2018
High SDI	Gastroesophageal reflux disease	Both	829421.493	825899.4622	832943.5238	2019
High SDI	Gastroesophageal reflux disease	Both	784647.2132	774124.7853	795169.641	2020
High SDI	Gastroesophageal reflux disease	Both	794841.5082	776436.1351	813246.8812	2021
High SDI	Gastroesophageal reflux disease	Both	804913.1539	776476.2667	833350.0412	2022
High SDI	Gastroesophageal reflux disease	Both	814854.3487	774640.9785	855067.7189	2023
High SDI	Gastroesophageal reflux disease	Both	824639.6875	771066.2742	878213.1008	2024
High SDI	Gastroesophageal reflux disease	Both	834270.9472	765823.9913	902717.9031	2025
High SDI	Gastroesophageal reflux disease	Both	843812.019	759066.1597	928557.8783	2026
High SDI	Gastroesophageal reflux disease	Both	853204.5032	750802.6786	955606.3279	2027
High SDI	Gastroesophageal reflux disease	Both	862452.4147	741079.6728	983825.1566	2028
High SDI	Gastroesophageal reflux disease	Both	871505.1166	729872.8823	1013137.351	2029
High SDI	Gastroesophageal reflux disease	Both	880333.2059	717160.8651	1043505.547	2030
High-middle SDI	Gastroesophageal reflux disease	Male	358788.2036	357156.3249	360420.0823	1990
High-middle SDI	Gastroesophageal reflux disease	Male	363807.6805	362217.9162	365397.4449	1991
High-middle SDI	Gastroesophageal reflux disease	Male	368866.7411	367265.524	370467.9581	1992
High-middle SDI	Gastroesophageal reflux disease	Male	373813.5004	372202.2135	375424.7874	1993
High-middle SDI	Gastroesophageal reflux disease	Male	378842.9821	377220.6526	380465.3117	1994
High-middle SDI	Gastroesophageal reflux disease	Male	384066.2429	382431.9932	385700.4925	1995
High-middle SDI	Gastroesophageal reflux disease	Male	389702.7145	388055.6894	391349.7397	1996
High-middle SDI	Gastroesophageal reflux disease	Male	395485.6734	393825.6602	397145.6865	1997
High-middle SDI	Gastroesophageal reflux disease	Male	401270.0412	399597.162	402942.9204	1998
High-middle SDI	Gastroesophageal reflux disease	Male	407097.5652	405411.7418	408783.3885	1999
High-middle SDI	Gastroesophageal reflux disease	Male	412916.3051	411217.6195	414614.9907	2000
High-middle SDI	Gastroesophageal reflux disease	Male	418874.138	417162.4297	420585.8463	2001

High-middle SDI	Gastroesophageal reflux disease	Male	424788.4196	423063.8893	426512.9498	2002
High-middle SDI	Gastroesophageal reflux disease	Male	430715.2786	428977.996	432452.5612	2003
High-middle SDI	Gastroesophageal reflux disease	Male	436725.0323	434974.8366	438475.228	2004
High-middle SDI	Gastroesophageal reflux disease	Male	442918.7357	441155.2811	444682.1902	2005
High-middle SDI	Gastroesophageal reflux disease	Male	448823.951	447048.0283	450599.8738	2006
High-middle SDI	Gastroesophageal reflux disease	Male	453943.6641	452157.1288	455730.1993	2007
High-middle SDI	Gastroesophageal reflux disease	Male	458590.3295	456794.1741	460386.4848	2008
High-middle SDI	Gastroesophageal reflux disease	Male	463344.7065	461538.6537	465150.7592	2009
High-middle SDI	Gastroesophageal reflux disease	Male	468942.1009	467124.4288	470759.773	2010
High-middle SDI	Gastroesophageal reflux disease	Male	475618.2194	473786.8246	477449.6142	2011
High-middle SDI	Gastroesophageal reflux disease	Male	482684.2829	480838.5048	484530.061	2012
High-middle SDI	Gastroesophageal reflux disease	Male	489592.5205	487732.8289	491452.2121	2013
High-middle SDI	Gastroesophageal reflux disease	Male	496322.1236	494448.8279	498195.4192	2014
High-middle SDI	Gastroesophageal reflux disease	Male	503330.6177	501442.8488	505218.3866	2015
High-middle SDI	Gastroesophageal reflux disease	Male	516116.3059	514203.3587	518029.253	2016
High-middle SDI	Gastroesophageal reflux disease	Male	528571.5047	526632.0119	530510.9974	2017
High-middle SDI	Gastroesophageal reflux disease	Male	534364.1066	532413.9198	536314.2933	2018
High-middle SDI	Gastroesophageal reflux disease	Male	539583.9857	537572.8397	541595.1317	2019
High-middle SDI	Gastroesophageal reflux disease	Male	265388.0481	262044.7421	268731.354	2020
High-middle SDI	Gastroesophageal reflux disease	Male	267906.4763	262126.463	273686.4895	2021
High-middle SDI	Gastroesophageal reflux disease	Male	270373.1674	261508.9007	279237.4341	2022
High-middle SDI	Gastroesophageal reflux disease	Male	272682.1265	260236.3671	285127.8859	2023
High-middle SDI	Gastroesophageal reflux disease	Male	274933.7974	258448.5872	291419.0076	2024
High-middle SDI	Gastroesophageal reflux disease	Male	277241.0094	256248.2611	298233.7577	2025
High-middle SDI	Gastroesophageal reflux disease	Male	279650.2129	253726.7483	305573.6775	2026
High-middle SDI	Gastroesophageal reflux disease	Male	281999.4621	250767.9266	313230.9975	2027
High-middle SDI	Gastroesophageal reflux disease	Male	284206.9297	247321.5179	321092.3416	2028
High-middle SDI	Gastroesophageal reflux disease	Male	286363.736	243467.4928	329259.9792	2029
High-middle SDI	Gastroesophageal reflux disease	Male	288551.6057	239253.1068	337850.1046	2030
High-middle SDI	Gastroesophageal reflux disease	Female	444148.8972	442326.1419	445971.6524	1990
High-middle SDI	Gastroesophageal reflux disease	Female	450542.8332	448757.6868	452327.9796	1991
High-middle SDI	Gastroesophageal reflux disease	Female	456996.9462	455198.5317	458795.3606	1992
High-middle SDI	Gastroesophageal reflux disease	Female	463293.329	461483.2382	465103.4197	1993
High-middle SDI	Gastroesophageal reflux disease	Female	469514.7183	467692.1137	471337.323	1994
High-middle SDI	Gastroesophageal reflux disease	Female	475863.668	474028.0146	477699.3213	1995
High-middle SDI	Gastroesophageal reflux disease	Female	483012.5492	481162.3063	484862.7921	1996
High-middle SDI	Gastroesophageal reflux disease	Female	491080.9115	489214.3437	492947.4793	1997
High-middle SDI	Gastroesophageal reflux disease	Female	499522.7356	497639.2664	501406.2048	1998
High-middle SDI	Gastroesophageal reflux disease	Female	507913.3689	506013.1265	509813.6113	1999
High-middle SDI	Gastroesophageal reflux disease	Female	515763.6872	513847.7545	517679.62	2000
High-middle SDI	Gastroesophageal reflux disease	Female	522271.045	520342.3728	524199.7172	2001
High-middle SDI	Gastroesophageal reflux disease	Female	527420.6407	525482.0014	529359.2799	2002
High-middle SDI	Gastroesophageal reflux disease	Female	531706.2847	529759.4085	533653.161	2003
High-middle SDI	Gastroesophageal reflux disease	Female	536146.1581	534190.7544	538101.5618	2004
High-middle SDI	Gastroesophageal reflux disease	Female	541311.292	539345.952	543276.632	2005
High-middle SDI	Gastroesophageal reflux disease	Female	546871.7784	544895.8435	548847.7134	2006
High-middle SDI	Gastroesophageal reflux disease	Female	551942.436	549956.9053	553927.9666	2007
High-middle SDI	Gastroesophageal reflux disease	Female	557028.4027	555033.2609	559023.5445	2008
High-middle SDI	Gastroesophageal reflux disease	Female	562685.4711	560679.6109	564691.3312	2009
High-middle SDI	Gastroesophageal reflux disease	Female	569565.6554	567546.8272	571584.4835	2010
High-middle SDI	Gastroesophageal reflux disease	Female	577556.1144	575522.3545	579589.8742	2011
High-middle SDI	Gastroesophageal reflux disease	Female	585809.3711	583760.3166	587858.4255	2012
High-middle SDI	Gastroesophageal reflux disease	Female	593942.4669	591878.5102	596006.4236	2013
High-middle SDI	Gastroesophageal reflux disease	Female	601909.7185	599831.168	603988.269	2014
High-middle SDI	Gastroesophageal reflux disease	Female	610007.1528	607913.3484	612100.9572	2015
High-middle SDI	Gastroesophageal reflux disease	Female	626306.1167	624183.4971	628428.7363	2016
High-middle SDI	Gastroesophageal reflux disease	Female	642167.6477	640014.5933	644320.7021	2017
High-middle SDI	Gastroesophageal reflux disease	Female	648722.6422	646558.6029	650886.6814	2018
High-middle SDI	Gastroesophageal reflux disease	Female	654406.6643	652185.5592	656627.7694	2019
High-middle SDI	Gastroesophageal reflux disease	Female	337046.5683	332700.7438	341392.3927	2020
High-middle SDI	Gastroesophageal reflux disease	Female	339791.891	332035.7158	347548.0662	2021
High-middle SDI	Gastroesophageal reflux disease	Female	342462.1747	330424.9413	354499.4082	2022

High-middle SDI	Gastroesophageal reflux disease	Female	344874.4537	327888.4542	361860.4532	2023
High-middle SDI	Gastroesophageal reflux disease	Female	347204.6354	324654.5527	369754.7182	2024
High-middle SDI	Gastroesophageal reflux disease	Female	349644.3775	320893.3415	378395.4135	2025
High-middle SDI	Gastroesophageal reflux disease	Female	352262.0242	316733.4987	387790.5496	2026
High-middle SDI	Gastroesophageal reflux disease	Female	354788.2897	311975.7648	397600.8146	2027
High-middle SDI	Gastroesophageal reflux disease	Female	357082.7697	306533.0469	407632.4924	2028
High-middle SDI	Gastroesophageal reflux disease	Female	359307.939	300551.3135	418064.5645	2029
High-middle SDI	Gastroesophageal reflux disease	Female	361605.622	294116.3314	429094.9127	2030
High-middle SDI	Gastroesophageal reflux disease	Both	802937.1007	799482.4668	806391.7347	1990
High-middle SDI	Gastroesophageal reflux disease	Both	814350.5137	810975.6029	817725.4244	1991
High-middle SDI	Gastroesophageal reflux disease	Both	825863.6873	822464.0558	829263.3187	1992
High-middle SDI	Gastroesophageal reflux disease	Both	837106.8294	833685.4517	840528.2071	1993
High-middle SDI	Gastroesophageal reflux disease	Both	848357.7005	844912.7663	851802.6347	1994
High-middle SDI	Gastroesophageal reflux disease	Both	859929.9108	856460.0078	863399.8139	1995
High-middle SDI	Gastroesophageal reflux disease	Both	872715.2637	869217.9956	876212.5317	1996
High-middle SDI	Gastroesophageal reflux disease	Both	886566.5849	883040.0039	890093.1658	1997
High-middle SDI	Gastroesophageal reflux disease	Both	900792.7768	897236.4284	904349.1252	1998
High-middle SDI	Gastroesophageal reflux disease	Both	915010.934	911424.8683	918596.9998	1999
High-middle SDI	Gastroesophageal reflux disease	Both	928679.9923	925065.374	932294.6107	2000
High-middle SDI	Gastroesophageal reflux disease	Both	941145.183	937504.8025	944785.5635	2001
High-middle SDI	Gastroesophageal reflux disease	Both	952209.0602	948545.8907	955872.2298	2002
High-middle SDI	Gastroesophageal reflux disease	Both	962421.5633	958737.4044	966105.7222	2003
High-middle SDI	Gastroesophageal reflux disease	Both	972871.1904	969165.591	976576.7898	2004
High-middle SDI	Gastroesophageal reflux disease	Both	984230.0277	980501.2331	987958.8222	2005
High-middle SDI	Gastroesophageal reflux disease	Both	995695.7295	991943.8718	999447.5872	2006
High-middle SDI	Gastroesophageal reflux disease	Both	1005886.1	1002114.034	1009658.166	2007
High-middle SDI	Gastroesophageal reflux disease	Both	1015618.732	1011827.435	1019410.029	2008
High-middle SDI	Gastroesophageal reflux disease	Both	1026030.178	1022218.265	1029842.09	2009
High-middle SDI	Gastroesophageal reflux disease	Both	1038507.756	1034671.256	1042344.256	2010
High-middle SDI	Gastroesophageal reflux disease	Both	1053174.334	1049309.179	1057039.488	2011
High-middle SDI	Gastroesophageal reflux disease	Both	1068493.654	1064598.821	1072388.486	2012
High-middle SDI	Gastroesophageal reflux disease	Both	1083534.987	1079611.339	1087458.636	2013
High-middle SDI	Gastroesophageal reflux disease	Both	1098231.842	1094279.996	1102183.688	2014
High-middle SDI	Gastroesophageal reflux disease	Both	1113337.771	1109356.197	1117319.344	2015
High-middle SDI	Gastroesophageal reflux disease	Both	1142422.423	1138386.856	1146457.989	2016
High-middle SDI	Gastroesophageal reflux disease	Both	1170739.152	1166646.605	1174831.7	2017
High-middle SDI	Gastroesophageal reflux disease	Both	1183086.749	1178972.523	1187200.975	2018
High-middle SDI	Gastroesophageal reflux disease	Both	1193990.65	1189758.399	1198222.901	2019
High-middle SDI	Gastroesophageal reflux disease	Both	602434.6163	594745.4859	610123.7467	2020
High-middle SDI	Gastroesophageal reflux disease	Both	607698.3672	594162.1788	621234.5557	2021
High-middle SDI	Gastroesophageal reflux disease	Both	612835.3421	591933.842	633736.8422	2022
High-middle SDI	Gastroesophageal reflux disease	Both	617556.5802	588124.8213	646988.3391	2023
High-middle SDI	Gastroesophageal reflux disease	Both	622138.4328	583103.1399	661173.7258	2024
High-middle SDI	Gastroesophageal reflux disease	Both	626885.3869	577141.6027	676629.1712	2025
High-middle SDI	Gastroesophageal reflux disease	Both	631912.237	570460.247	693364.2271	2026
High-middle SDI	Gastroesophageal reflux disease	Both	636787.7517	562743.6914	710831.8121	2027
High-middle SDI	Gastroesophageal reflux disease	Both	641289.6994	553854.5648	728724.834	2028
High-middle SDI	Gastroesophageal reflux disease	Both	645671.675	544018.8063	747324.5437	2029
High-middle SDI	Gastroesophageal reflux disease	Both	650157.2277	533369.4381	766945.0173	2030
Middle SDI	Gastroesophageal reflux disease	Male	437481.835	435679.2798	439284.3902	1990
Middle SDI	Gastroesophageal reflux disease	Male	448041.4537	446274.8613	449808.046	1991
Middle SDI	Gastroesophageal reflux disease	Male	458774.484	456985.7539	460563.2142	1992
Middle SDI	Gastroesophageal reflux disease	Male	469917.7134	468107.3544	471728.0725	1993
Middle SDI	Gastroesophageal reflux disease	Male	481578.6278	479744.9205	483412.3351	1994
Middle SDI	Gastroesophageal reflux disease	Male	493717.8416	491859.5823	495576.101	1995
Middle SDI	Gastroesophageal reflux disease	Male	506564.7505	504680.8645	508448.6366	1996
Middle SDI	Gastroesophageal reflux disease	Male	519866.4477	517956.3586	521776.5369	1997
Middle SDI	Gastroesophageal reflux disease	Male	533421.9871	531485.5659	535358.4082	1998
Middle SDI	Gastroesophageal reflux disease	Male	547042.709	545080.122	549005.2959	1999
Middle SDI	Gastroesophageal reflux disease	Male	560485.7486	558497.6715	562473.8257	2000
Middle SDI	Gastroesophageal reflux disease	Male	574086.1992	572072.6762	576099.7222	2001
Middle SDI	Gastroesophageal reflux disease	Male	588001.3203	585962.0711	590040.5695	2002

Middle SDI	Gastroesophageal reflux disease	Male	602250.5775	600185.3503	604315.8047	2003
Middle SDI	Gastroesophageal reflux disease	Male	616538.3682	614447.3363	618629.4001	2004
Middle SDI	Gastroesophageal reflux disease	Male	630579.649	628463.458	632695.84	2005
Middle SDI	Gastroesophageal reflux disease	Male	643981.0831	641841.2049	646120.9613	2006
Middle SDI	Gastroesophageal reflux disease	Male	656388.6714	654227.1565	658550.1863	2007
Middle SDI	Gastroesophageal reflux disease	Male	667997.4422	665815.8599	670179.0245	2008
Middle SDI	Gastroesophageal reflux disease	Male	679747.6065	677545.8317	681949.3812	2009
Middle SDI	Gastroesophageal reflux disease	Male	693125.9914	690901.4471	695350.5357	2010
Middle SDI	Gastroesophageal reflux disease	Male	707931.4838	705681.9803	710180.9872	2011
Middle SDI	Gastroesophageal reflux disease	Male	723169.0802	720894.1947	725443.9657	2012
Middle SDI	Gastroesophageal reflux disease	Male	738688.3875	736387.9964	740988.7786	2013
Middle SDI	Gastroesophageal reflux disease	Male	754667.1037	752340.6251	756993.5822	2014
Middle SDI	Gastroesophageal reflux disease	Male	770875.441	768522.6868	773228.1951	2015
Middle SDI	Gastroesophageal reflux disease	Male	791794.9996	789408.6801	794181.3191	2016
Middle SDI	Gastroesophageal reflux disease	Male	812514.1415	810093.5449	814934.7381	2017
Middle SDI	Gastroesophageal reflux disease	Male	827827.5327	825383.2345	830271.8309	2018
Middle SDI	Gastroesophageal reflux disease	Male	842574.0697	840055.0497	845093.0896	2019
Middle SDI	Gastroesophageal reflux disease	Male	1074054.658	1062310.428	1085798.887	2020
Middle SDI	Gastroesophageal reflux disease	Male	1090106.788	1069257.822	1110955.753	2021
Middle SDI	Gastroesophageal reflux disease	Male	1105811.036	1073453.474	1138168.598	2022
Middle SDI	Gastroesophageal reflux disease	Male	1121153.696	1075343.906	1166963.486	2023
Middle SDI	Gastroesophageal reflux disease	Male	1136304.427	1075202.334	1197406.519	2024
Middle SDI	Gastroesophageal reflux disease	Male	1151237.09	1072937.056	1229537.124	2025
Middle SDI	Gastroesophageal reflux disease	Male	1165932.884	1068747.594	1263118.175	2026
Middle SDI	Gastroesophageal reflux disease	Male	1180352.032	1062724.329	1297979.736	2027
Middle SDI	Gastroesophageal reflux disease	Male	1194409.404	1054860.362	1333958.446	2028
Middle SDI	Gastroesophageal reflux disease	Male	1208289.749	1045295.531	1371283.966	2029
Middle SDI	Gastroesophageal reflux disease	Male	1221947.774	1033869.889	1410025.659	2030
Middle SDI	Gastroesophageal reflux disease	Female	462668.6899	460814.721	464522.6588	1990
Middle SDI	Gastroesophageal reflux disease	Female	474844.0088	473023.8589	476664.1588	1991
Middle SDI	Gastroesophageal reflux disease	Female	487131.4613	485286.5945	488976.3282	1992
Middle SDI	Gastroesophageal reflux disease	Female	499769.3881	497900.542	501638.2343	1993
Middle SDI	Gastroesophageal reflux disease	Female	512832.635	510938.3906	514726.8794	1994
Middle SDI	Gastroesophageal reflux disease	Female	526293.2978	524372.6614	528213.9342	1995
Middle SDI	Gastroesophageal reflux disease	Female	540232.9235	538285.3348	542180.5122	1996
Middle SDI	Gastroesophageal reflux disease	Female	554514.4289	552539.5761	556489.2817	1997
Middle SDI	Gastroesophageal reflux disease	Female	569130.506	567128.1605	571132.8514	1998
Middle SDI	Gastroesophageal reflux disease	Female	583971.6991	581941.7826	586001.6156	1999
Middle SDI	Gastroesophageal reflux disease	Female	599050.4801	596992.8709	601108.0893	2000
Middle SDI	Gastroesophageal reflux disease	Female	614411.9884	612326.5445	616497.4322	2001
Middle SDI	Gastroesophageal reflux disease	Female	629972.7656	627859.4759	632086.0554	2002
Middle SDI	Gastroesophageal reflux disease	Female	645803.5021	643662.2698	647944.7343	2003
Middle SDI	Gastroesophageal reflux disease	Female	661719.3183	659550.2872	663888.3494	2004
Middle SDI	Gastroesophageal reflux disease	Female	677708.4134	675511.7408	679905.0861	2005
Middle SDI	Gastroesophageal reflux disease	Female	693297.7542	691074.5284	695520.9799	2006
Middle SDI	Gastroesophageal reflux disease	Female	708066.4745	705818.4412	710314.5078	2007
Middle SDI	Gastroesophageal reflux disease	Female	722241.6034	719970.011	724513.1957	2008
Middle SDI	Gastroesophageal reflux disease	Female	736589.5045	734294.2139	738884.7951	2009
Middle SDI	Gastroesophageal reflux disease	Female	752224.1115	749903.231	754544.9921	2010
Middle SDI	Gastroesophageal reflux disease	Female	769089.5842	766741.4099	771437.7585	2011
Middle SDI	Gastroesophageal reflux disease	Female	786248.4206	783872.84	788624.0013	2012
Middle SDI	Gastroesophageal reflux disease	Female	803750.8898	801347.7341	806154.0455	2013
Middle SDI	Gastroesophageal reflux disease	Female	821589.4756	819158.4111	824020.5402	2014
Middle SDI	Gastroesophageal reflux disease	Female	839773.8058	837314.4994	842233.1122	2015
Middle SDI	Gastroesophageal reflux disease	Female	863756.9214	861260.8401	866253.0026	2016
Middle SDI	Gastroesophageal reflux disease	Female	887563.1556	885029.539	890096.7721	2017
Middle SDI	Gastroesophageal reflux disease	Female	904792.024	902232.9617	907351.0863	2018
Middle SDI	Gastroesophageal reflux disease	Female	921353.5221	918718.2341	923988.8101	2019
Middle SDI	Gastroesophageal reflux disease	Female	1167461.154	1154981.961	1179940.347	2020
Middle SDI	Gastroesophageal reflux disease	Female	1186030.696	1163916.938	1208144.454	2021
Middle SDI	Gastroesophageal reflux disease	Female	1204214.563	1169917.447	1238511.678	2022
Middle SDI	Gastroesophageal reflux disease	Female	1221881.923	1173334.074	1270429.772	2023

Middle SDI	Gastroesophageal reflux disease	Female	1239200.772	1174441.542	1303960.002	2024
Middle SDI	Gastroesophageal reflux disease	Female	1256224.37	1173220.448	1339228.292	2025
Middle SDI	Gastroesophageal reflux disease	Female	1273083.275	1170019.9	1376146.651	2026
Middle SDI	Gastroesophageal reflux disease	Female	1289642.726	1164846.6	1414438.852	2027
Middle SDI	Gastroesophageal reflux disease	Female	1305697.575	1157588.851	1453806.299	2028
Middle SDI	Gastroesophageal reflux disease	Female	1321405.258	1148364.335	1494446.18	2029
Middle SDI	Gastroesophageal reflux disease	Female	1336795.482	1137075.829	1536515.134	2030
Middle SDI	Gastroesophageal reflux disease	Both	900150.5249	896494.0008	903807.049	1990
Middle SDI	Gastroesophageal reflux disease	Both	922885.4625	919298.7202	926472.2048	1991
Middle SDI	Gastroesophageal reflux disease	Both	945905.9454	942272.3483	949539.5424	1992
Middle SDI	Gastroesophageal reflux disease	Both	969687.1015	966007.8963	973366.3068	1993
Middle SDI	Gastroesophageal reflux disease	Both	994411.2628	990683.3111	998139.2144	1994
Middle SDI	Gastroesophageal reflux disease	Both	1020011.139	1016232.244	1023790.035	1995
Middle SDI	Gastroesophageal reflux disease	Both	1046797.674	1042966.199	1050629.149	1996
Middle SDI	Gastroesophageal reflux disease	Both	1074380.877	1070495.935	1078265.819	1997
Middle SDI	Gastroesophageal reflux disease	Both	1102552.493	1098613.726	1106491.26	1998
Middle SDI	Gastroesophageal reflux disease	Both	1131014.408	1127021.905	1135006.912	1999
Middle SDI	Gastroesophageal reflux disease	Both	1159536.229	1155490.542	1163581.915	2000
Middle SDI	Gastroesophageal reflux disease	Both	1188498.188	1184399.221	1192597.154	2001
Middle SDI	Gastroesophageal reflux disease	Both	1217974.086	1213821.547	1222126.625	2002
Middle SDI	Gastroesophageal reflux disease	Both	1248054.08	1243847.62	1252260.539	2003
Middle SDI	Gastroesophageal reflux disease	Both	1278257.687	1273997.624	1282517.75	2004
Middle SDI	Gastroesophageal reflux disease	Both	1308288.062	1303975.199	1312600.926	2005
Middle SDI	Gastroesophageal reflux disease	Both	1337278.837	1332915.733	1341641.941	2006
Middle SDI	Gastroesophageal reflux disease	Both	1364455.146	1360045.598	1368864.694	2007
Middle SDI	Gastroesophageal reflux disease	Both	1390239.046	1385785.871	1394692.22	2008
Middle SDI	Gastroesophageal reflux disease	Both	1416337.111	1411840.046	1420834.176	2009
Middle SDI	Gastroesophageal reflux disease	Both	1445350.103	1440804.678	1449895.528	2010
Middle SDI	Gastroesophageal reflux disease	Both	1477021.068	1472423.39	1481618.746	2011
Middle SDI	Gastroesophageal reflux disease	Both	1509417.501	1504767.035	1514067.967	2012
Middle SDI	Gastroesophageal reflux disease	Both	1542439.277	1537735.731	1547142.824	2013
Middle SDI	Gastroesophageal reflux disease	Both	1576256.579	1571499.036	1581014.122	2014
Middle SDI	Gastroesophageal reflux disease	Both	1610649.247	1605837.186	1615461.307	2015
Middle SDI	Gastroesophageal reflux disease	Both	1655551.921	1650669.52	1660434.322	2016
Middle SDI	Gastroesophageal reflux disease	Both	1700077.297	1695123.084	1705031.51	2017
Middle SDI	Gastroesophageal reflux disease	Both	1732619.557	1727616.196	1737622.917	2018
Middle SDI	Gastroesophageal reflux disease	Both	1763927.592	1758773.284	1769081.9	2019
Middle SDI	Gastroesophageal reflux disease	Both	2241515.812	2217292.39	2265739.234	2020
Middle SDI	Gastroesophageal reflux disease	Both	2276137.484	2233174.76	2319100.208	2021
Middle SDI	Gastroesophageal reflux disease	Both	2310025.599	2243370.921	2376680.277	2022
Middle SDI	Gastroesophageal reflux disease	Both	2343035.619	2248677.979	2437393.258	2023
Middle SDI	Gastroesophageal reflux disease	Both	2375505.199	2249643.876	2501366.522	2024
Middle SDI	Gastroesophageal reflux disease	Both	2407461.46	2246157.503	2568765.416	2025
Middle SDI	Gastroesophageal reflux disease	Both	2439016.16	2238767.494	2639264.825	2026
Middle SDI	Gastroesophageal reflux disease	Both	2469994.758	2227570.929	2712418.587	2027
Middle SDI	Gastroesophageal reflux disease	Both	2500106.979	2212449.213	2787764.745	2028
Middle SDI	Gastroesophageal reflux disease	Both	2529695.007	2193659.866	2865730.147	2029
Middle SDI	Gastroesophageal reflux disease	Both	2558743.255	2170945.718	2946540.793	2030
Low-middle SDI	Gastroesophageal reflux disease	Male	383247.2195	381566.9566	384927.4825	1990
Low-middle SDI	Gastroesophageal reflux disease	Male	392370.7233	390729.9722	394011.4743	1991
Low-middle SDI	Gastroesophageal reflux disease	Male	401521.3845	399860.942	403181.8269	1992
Low-middle SDI	Gastroesophageal reflux disease	Male	410748.6466	409068.9618	412428.3315	1993
Low-middle SDI	Gastroesophageal reflux disease	Male	420032.7374	418333.494	421731.9809	1994
Low-middle SDI	Gastroesophageal reflux disease	Male	429441.6444	427722.1264	431161.1624	1995
Low-middle SDI	Gastroesophageal reflux disease	Male	439489.6012	437748.5497	441230.6527	1996
Low-middle SDI	Gastroesophageal reflux disease	Male	450266.8394	448502.9428	452030.736	1997
Low-middle SDI	Gastroesophageal reflux disease	Male	461373.8974	459586.6561	463161.1388	1998
Low-middle SDI	Gastroesophageal reflux disease	Male	472548.3944	470737.8556	474358.9333	1999
Low-middle SDI	Gastroesophageal reflux disease	Male	483427.6844	481594.7686	485260.6002	2000
Low-middle SDI	Gastroesophageal reflux disease	Male	493811.9534	491958.1596	495665.7471	2001
Low-middle SDI	Gastroesophageal reflux disease	Male	504074.9674	502200.8813	505949.0535	2002
Low-middle SDI	Gastroesophageal reflux disease	Male	514532.8092	512638.178	516427.4403	2003

Low-middle SDI	Gastroesophageal reflux disease	Male	525609.5724	523693.2481	527525.8966	2004
Low-middle SDI	Gastroesophageal reflux disease	Male	537491.8893	535552.4946	539431.284	2005
Low-middle SDI	Gastroesophageal reflux disease	Male	549709.9622	547747.1364	551672.788	2006
Low-middle SDI	Gastroesophageal reflux disease	Male	562020.9816	560034.8554	564007.1079	2007
Low-middle SDI	Gastroesophageal reflux disease	Male	574377.0404	572367.8213	576386.2596	2008
Low-middle SDI	Gastroesophageal reflux disease	Male	587137.1395	585104.3249	589169.954	2009
Low-middle SDI	Gastroesophageal reflux disease	Male	600539.386	598482.0292	602596.7428	2010
Low-middle SDI	Gastroesophageal reflux disease	Male	614337.5309	612255.1655	616419.8962	2011
Low-middle SDI	Gastroesophageal reflux disease	Male	628298.147	626190.8057	630405.4884	2012
Low-middle SDI	Gastroesophageal reflux disease	Male	642521.8016	640389.3188	644654.2844	2013
Low-middle SDI	Gastroesophageal reflux disease	Male	657033.2449	654875.3419	659191.1479	2014
Low-middle SDI	Gastroesophageal reflux disease	Male	672007.9677	669824.0205	674191.9149	2015
Low-middle SDI	Gastroesophageal reflux disease	Male	687876.4081	685664.667	690088.1492	2016
Low-middle SDI	Gastroesophageal reflux disease	Male	703444.1306	701204.635	705683.6262	2017
Low-middle SDI	Gastroesophageal reflux disease	Male	718731.7815	716466.3544	720997.2085	2018
Low-middle SDI	Gastroesophageal reflux disease	Male	734491.2611	732145.1247	736837.3975	2019
Low-middle SDI	Gastroesophageal reflux disease	Male	958925.1522	948881.5506	968968.7538	2020
Low-middle SDI	Gastroesophageal reflux disease	Male	981059.9114	963752.0432	998367.7797	2021
Low-middle SDI	Gastroesophageal reflux disease	Male	1003327.446	976634.8403	1030020.051	2022
Low-middle SDI	Gastroesophageal reflux disease	Male	1025784.311	987904.2204	1063664.401	2023
Low-middle SDI	Gastroesophageal reflux disease	Male	1048535.978	997748.3056	1099323.651	2024
Low-middle SDI	Gastroesophageal reflux disease	Male	1071481.304	1006064.383	1136898.226	2025
Low-middle SDI	Gastroesophageal reflux disease	Male	1094564.758	1012870.351	1176259.165	2026
Low-middle SDI	Gastroesophageal reflux disease	Male	1117643.491	1018067.594	1217219.388	2027
Low-middle SDI	Gastroesophageal reflux disease	Male	1140793.291	1021730.829	1259855.753	2028
Low-middle SDI	Gastroesophageal reflux disease	Male	1164125.242	1023923.979	1304326.505	2029
Low-middle SDI	Gastroesophageal reflux disease	Male	1187502.917	1024461.97	1350543.864	2030
Low-middle SDI	Gastroesophageal reflux disease	Female	377453.3338	375782.7242	379123.9433	1990
Low-middle SDI	Gastroesophageal reflux disease	Female	386912.7982	385277.0529	388548.5436	1991
Low-middle SDI	Gastroesophageal reflux disease	Female	396536.6665	394879.7936	398193.5395	1992
Low-middle SDI	Gastroesophageal reflux disease	Female	406365.8653	404688.3069	408043.4237	1993
Low-middle SDI	Gastroesophageal reflux disease	Female	416492.0925	414792.9555	418191.2295	1994
Low-middle SDI	Gastroesophageal reflux disease	Female	426896.5597	425174.8604	428618.259	1995
Low-middle SDI	Gastroesophageal reflux disease	Female	438484.068	436737.4461	440230.6899	1996
Low-middle SDI	Gastroesophageal reflux disease	Female	451464.3125	449690.1789	453238.446	1997
Low-middle SDI	Gastroesophageal reflux disease	Female	465063.3824	463260.6912	466866.0737	1998
Low-middle SDI	Gastroesophageal reflux disease	Female	478716.866	476885.6995	480548.0324	1999
Low-middle SDI	Gastroesophageal reflux disease	Female	491480.8524	489623.5567	493338.1481	2000
Low-middle SDI	Gastroesophageal reflux disease	Female	502934.7845	501054.7918	504814.7772	2001
Low-middle SDI	Gastroesophageal reflux disease	Female	513483.8046	511583.3386	515384.2705	2002
Low-middle SDI	Gastroesophageal reflux disease	Female	523955.6254	522034.8552	525876.3957	2003
Low-middle SDI	Gastroesophageal reflux disease	Female	535266.6132	533323.8818	537209.3447	2004
Low-middle SDI	Gastroesophageal reflux disease	Female	547957.4733	545990.3579	549924.5887	2005
Low-middle SDI	Gastroesophageal reflux disease	Female	561758.476	559765.2322	563751.7199	2006
Low-middle SDI	Gastroesophageal reflux disease	Female	575763.9001	573744.4878	577783.3124	2007
Low-middle SDI	Gastroesophageal reflux disease	Female	589992.2459	587946.6199	592037.872	2008
Low-middle SDI	Gastroesophageal reflux disease	Female	604583.7151	602511.5195	606655.9106	2009
Low-middle SDI	Gastroesophageal reflux disease	Female	619934.9681	617835.1236	622034.8127	2010
Low-middle SDI	Gastroesophageal reflux disease	Female	635225.814	633098.7501	637352.8779	2011
Low-middle SDI	Gastroesophageal reflux disease	Female	650210.3113	648056.9131	652363.7095	2012
Low-middle SDI	Gastroesophageal reflux disease	Female	665234.2504	663054.7688	667413.732	2013
Low-middle SDI	Gastroesophageal reflux disease	Female	680666.749	678460.724	682872.774	2014
Low-middle SDI	Gastroesophageal reflux disease	Female	697009.4455	694775.572	699243.319	2015
Low-middle SDI	Gastroesophageal reflux disease	Female	716331.1387	714064.4694	718597.808	2016
Low-middle SDI	Gastroesophageal reflux disease	Female	735732.4032	733432.0757	738032.7307	2017
Low-middle SDI	Gastroesophageal reflux disease	Female	752783.6053	750455.2405	755111.9701	2018
Low-middle SDI	Gastroesophageal reflux disease	Female	770190.6037	767783.8896	772597.3179	2019
Low-middle SDI	Gastroesophageal reflux disease	Female	993808.0879	982845.0585	1004771.117	2020
Low-middle SDI	Gastroesophageal reflux disease	Female	1017279.639	998133.1343	1036426.143	2021
Low-middle SDI	Gastroesophageal reflux disease	Female	1040882.923	1011158.767	1070607.08	2022
Low-middle SDI	Gastroesophageal reflux disease	Female	1064580.475	1022241.23	1106919.721	2023
Low-middle SDI	Gastroesophageal reflux disease	Female	1088498.098	1031603.355	1145392.84	2024

Low-middle SDI	Gastroesophageal reflux disease	Female	1112611.936	1039222.343	1186001.53	2025
Low-middle SDI	Gastroesophageal reflux disease	Female	1136933.872	1045176.043	1228691.702	2026
Low-middle SDI	Gastroesophageal reflux disease	Female	1161268.186	1049314.841	1273221.531	2027
Low-middle SDI	Gastroesophageal reflux disease	Female	1185601.9	1051633.217	1319570.583	2028
Low-middle SDI	Gastroesophageal reflux disease	Female	1210061.993	1052211.118	1367912.869	2029
Low-middle SDI	Gastroesophageal reflux disease	Female	1234581.956	1050925.69	1418238.223	2030
Low-middle SDI	Gastroesophageal reflux disease	Both	760700.5533	757349.6808	764051.4258	1990
Low-middle SDI	Gastroesophageal reflux disease	Both	779283.5215	776007.0251	782560.0179	1991
Low-middle SDI	Gastroesophageal reflux disease	Both	798058.051	794740.7356	801375.3664	1992
Low-middle SDI	Gastroesophageal reflux disease	Both	817114.5119	813757.2687	820471.7552	1993
Low-middle SDI	Gastroesophageal reflux disease	Both	836524.83	833126.4496	839923.2104	1994
Low-middle SDI	Gastroesophageal reflux disease	Both	856338.2041	852896.9868	859779.4214	1995
Low-middle SDI	Gastroesophageal reflux disease	Both	877973.6692	874485.9958	881461.3426	1996
Low-middle SDI	Gastroesophageal reflux disease	Both	901731.1519	898193.1217	905269.182	1997
Low-middle SDI	Gastroesophageal reflux disease	Both	926437.2799	922847.3473	930027.2125	1998
Low-middle SDI	Gastroesophageal reflux disease	Both	951265.2604	947623.5551	954906.9657	1999
Low-middle SDI	Gastroesophageal reflux disease	Both	974908.5368	971218.3253	978598.7483	2000
Low-middle SDI	Gastroesophageal reflux disease	Both	996746.7379	993012.9514	1000480.524	2001
Low-middle SDI	Gastroesophageal reflux disease	Both	1017558.772	1013784.22	1021333.324	2002
Low-middle SDI	Gastroesophageal reflux disease	Both	1038488.435	1034673.033	1042303.836	2003
Low-middle SDI	Gastroesophageal reflux disease	Both	1060876.186	1057017.13	1064735.241	2004
Low-middle SDI	Gastroesophageal reflux disease	Both	1085449.363	1081542.852	1089355.873	2005
Low-middle SDI	Gastroesophageal reflux disease	Both	1111468.438	1107512.369	1115424.508	2006
Low-middle SDI	Gastroesophageal reflux disease	Both	1137784.882	1133779.343	1141790.42	2007
Low-middle SDI	Gastroesophageal reflux disease	Both	1164369.286	1160314.441	1168424.132	2008
Low-middle SDI	Gastroesophageal reflux disease	Both	1191720.855	1187615.844	1195825.865	2009
Low-middle SDI	Gastroesophageal reflux disease	Both	1220474.354	1216317.153	1224631.555	2010
Low-middle SDI	Gastroesophageal reflux disease	Both	1249563.345	1245353.916	1253772.774	2011
Low-middle SDI	Gastroesophageal reflux disease	Both	1278508.458	1274247.719	1282769.198	2012
Low-middle SDI	Gastroesophageal reflux disease	Both	1307756.052	1303444.088	1312068.016	2013
Low-middle SDI	Gastroesophageal reflux disease	Both	1337699.994	1333336.066	1342063.922	2014
Low-middle SDI	Gastroesophageal reflux disease	Both	1369017.413	1364599.593	1373435.234	2015
Low-middle SDI	Gastroesophageal reflux disease	Both	1404207.547	1399729.136	1408685.957	2016
Low-middle SDI	Gastroesophageal reflux disease	Both	1439176.534	1434636.711	1443716.357	2017
Low-middle SDI	Gastroesophageal reflux disease	Both	1471515.387	1466921.595	1476109.179	2018
Low-middle SDI	Gastroesophageal reflux disease	Both	1504681.865	1499929.014	1509434.715	2019
Low-middle SDI	Gastroesophageal reflux disease	Both	1952733.24	1931726.609	1973739.871	2020
Low-middle SDI	Gastroesophageal reflux disease	Both	1998339.55	1961885.177	2034793.923	2021
Low-middle SDI	Gastroesophageal reflux disease	Both	2044210.369	1987793.607	2100627.131	2022
Low-middle SDI	Gastroesophageal reflux disease	Both	2090364.786	2010145.45	2170584.122	2023
Low-middle SDI	Gastroesophageal reflux disease	Both	2137034.076	2029351.661	2244716.492	2024
Low-middle SDI	Gastroesophageal reflux disease	Both	2184093.241	2045286.725	2322899.756	2025
Low-middle SDI	Gastroesophageal reflux disease	Both	2231498.63	2058046.394	2404950.867	2026
Low-middle SDI	Gastroesophageal reflux disease	Both	2278911.677	2067382.435	2490440.918	2027
Low-middle SDI	Gastroesophageal reflux disease	Both	2326395.191	2073364.046	2579426.336	2028
Low-middle SDI	Gastroesophageal reflux disease	Both	2374187.235	2076135.097	2672239.374	2029
Low-middle SDI	Gastroesophageal reflux disease	Both	2422084.873	2075387.659	2768782.087	2030
Low SDI	Gastroesophageal reflux disease	Male	158911.8913	157847.5733	159976.2093	1990
Low SDI	Gastroesophageal reflux disease	Male	163003.7249	161983.7733	164023.6764	1991
Low SDI	Gastroesophageal reflux disease	Male	167301.1444	166268.6603	168333.6284	1992
Low SDI	Gastroesophageal reflux disease	Male	171597.5541	170551.1153	172643.993	1993
Low SDI	Gastroesophageal reflux disease	Male	176011.5346	174951.1502	177071.9189	1994
Low SDI	Gastroesophageal reflux disease	Male	180620.1532	179544.7757	181695.5307	1995
Low SDI	Gastroesophageal reflux disease	Male	185485.5637	184394.2951	186576.8323	1996
Low SDI	Gastroesophageal reflux disease	Male	190383.8103	189276.6578	191490.9628	1997
Low SDI	Gastroesophageal reflux disease	Male	195185.2861	194062.7492	196307.823	1998
Low SDI	Gastroesophageal reflux disease	Male	200068.6724	198930.6787	201206.6662	1999
Low SDI	Gastroesophageal reflux disease	Male	205182.7858	204028.7894	206336.7822	2000
Low SDI	Gastroesophageal reflux disease	Male	210830.2272	209658.7756	212001.6787	2001
Low SDI	Gastroesophageal reflux disease	Male	216927.8593	215737.8069	218117.9118	2002
Low SDI	Gastroesophageal reflux disease	Male	223187.8162	221978.9328	224396.6996	2003
Low SDI	Gastroesophageal reflux disease	Male	229634.9842	228406.9727	230862.9958	2004

Low SDI	Gastroesophageal reflux disease	Male	236222.8463	234975.5664	237470.1262	2005
Low SDI	Gastroesophageal reflux disease	Male	242936.7855	241670.1685	244203.4025	2006
Low SDI	Gastroesophageal reflux disease	Male	249966.1222	248679.5329	251252.7115	2007
Low SDI	Gastroesophageal reflux disease	Male	257275.0544	255967.9821	258582.1267	2008
Low SDI	Gastroesophageal reflux disease	Male	264845.6261	263517.6417	266173.6105	2009
Low SDI	Gastroesophageal reflux disease	Male	272633.9805	271284.7913	273983.1697	2010
Low SDI	Gastroesophageal reflux disease	Male	280743.5159	279372.5756	282114.4562	2011
Low SDI	Gastroesophageal reflux disease	Male	289169.3551	287776.146	290562.5642	2012
Low SDI	Gastroesophageal reflux disease	Male	297847.1807	296431.3608	299263.0007	2013
Low SDI	Gastroesophageal reflux disease	Male	306682.8173	305244.2667	308121.3679	2014
Low SDI	Gastroesophageal reflux disease	Male	315785.2137	314323.4165	317247.011	2015
Low SDI	Gastroesophageal reflux disease	Male	325002.2047	323516.628	326487.7814	2016
Low SDI	Gastroesophageal reflux disease	Male	334737.004	333226.3754	336247.6325	2017
Low SDI	Gastroesophageal reflux disease	Male	345119.2874	343582.5813	346655.9934	2018
Low SDI	Gastroesophageal reflux disease	Male	355910.7576	354291.1589	357530.3562	2019
Low SDI	Gastroesophageal reflux disease	Male	309942.1086	306280.5363	313603.6809	2020
Low SDI	Gastroesophageal reflux disease	Male	320182.4895	314063.3523	326301.6266	2021
Low SDI	Gastroesophageal reflux disease	Male	330667.5411	321301.7094	340033.3728	2022
Low SDI	Gastroesophageal reflux disease	Male	341477.4407	328168.2484	354786.633	2023
Low SDI	Gastroesophageal reflux disease	Male	352678.3406	334741.2894	370615.3919	2024
Low SDI	Gastroesophageal reflux disease	Male	364229.6372	340965.0098	387494.2646	2025
Low SDI	Gastroesophageal reflux disease	Male	376078.1648	346788.2892	405368.0404	2026
Low SDI	Gastroesophageal reflux disease	Male	388149.1897	352130.6462	424167.7332	2027
Low SDI	Gastroesophageal reflux disease	Male	400507.5094	357030.3332	443984.6855	2028
Low SDI	Gastroesophageal reflux disease	Male	413220.5643	361512.6284	464928.5003	2029
Low SDI	Gastroesophageal reflux disease	Male	426245.6233	365496.1092	486995.1374	2030
Low SDI	Gastroesophageal reflux disease	Female	163506.607	162426.2257	164586.9883	1990
Low SDI	Gastroesophageal reflux disease	Female	167613.2666	166577.5555	168648.9776	1991
Low SDI	Gastroesophageal reflux disease	Female	171885.1571	170837.1298	172933.1843	1992
Low SDI	Gastroesophageal reflux disease	Female	176183.6323	175121.8827	177245.3819	1993
Low SDI	Gastroesophageal reflux disease	Female	180798.1982	179722.0466	181874.3497	1994
Low SDI	Gastroesophageal reflux disease	Female	185735.2649	184643.2597	186827.2701	1995
Low SDI	Gastroesophageal reflux disease	Female	190935.6445	189826.9094	192044.3797	1996
Low SDI	Gastroesophageal reflux disease	Female	196230.0619	195104.4342	197355.6897	1997
Low SDI	Gastroesophageal reflux disease	Female	201502.2518	200360.0138	202644.4898	1998
Low SDI	Gastroesophageal reflux disease	Female	206894.0149	205735.0048	208053.0251	1999
Low SDI	Gastroesophageal reflux disease	Female	212572.5699	211396.1094	213749.0303	2000
Low SDI	Gastroesophageal reflux disease	Female	218639.2875	217444.4358	219834.1391	2001
Low SDI	Gastroesophageal reflux disease	Female	225094.3491	223880.1865	226308.5118	2002
Low SDI	Gastroesophageal reflux disease	Female	231688.4913	230454.8826	232922.1	2003
Low SDI	Gastroesophageal reflux disease	Female	238484.416	237231.0486	239737.7833	2004
Low SDI	Gastroesophageal reflux disease	Female	245543.7511	244270.1506	246817.3515	2005
Low SDI	Gastroesophageal reflux disease	Female	252832.0317	251537.8603	254126.2031	2006
Low SDI	Gastroesophageal reflux disease	Female	260504.7618	259189.2429	261820.2806	2007
Low SDI	Gastroesophageal reflux disease	Female	268486.1499	267148.7484	269823.5515	2008
Low SDI	Gastroesophageal reflux disease	Female	276750.9635	275391.2413	278110.6856	2009
Low SDI	Gastroesophageal reflux disease	Female	285294.1052	283911.659	286676.5515	2010
Low SDI	Gastroesophageal reflux disease	Female	294172.9342	292767.235	295578.6333	2011
Low SDI	Gastroesophageal reflux disease	Female	303391.1034	301961.6252	304820.5816	2012
Low SDI	Gastroesophageal reflux disease	Female	312901.8967	311448.2469	314355.5464	2013
Low SDI	Gastroesophageal reflux disease	Female	322617.4773	321139.4656	324095.4889	2014
Low SDI	Gastroesophageal reflux disease	Female	332633.9724	331131.0652	334136.8797	2015
Low SDI	Gastroesophageal reflux disease	Female	343092.9718	341563.8647	344622.0788	2016
Low SDI	Gastroesophageal reflux disease	Female	354092.3042	352535.7242	355648.8842	2017
Low SDI	Gastroesophageal reflux disease	Female	365483.5025	363899.2644	367067.7407	2018
Low SDI	Gastroesophageal reflux disease	Female	377248.6282	375579.8305	378917.4259	2019
Low SDI	Gastroesophageal reflux disease	Female	333143.6057	329242.4617	337044.7497	2020
Low SDI	Gastroesophageal reflux disease	Female	344514.9985	337967.1312	351062.8658	2021
Low SDI	Gastroesophageal reflux disease	Female	356124.9039	346076.8371	366172.9707	2022
Low SDI	Gastroesophageal reflux disease	Female	368027.2875	353725.1022	382329.4727	2023
Low SDI	Gastroesophageal reflux disease	Female	380275.7665	360980.1958	399571.3372	2024
Low SDI	Gastroesophageal reflux disease	Female	392857.3581	367812.6775	417902.0386	2025

Low SDI	Gastroesophageal reflux disease	Female	405738.7792	374190.8358	437286.7225	2026
Low SDI	Gastroesophageal reflux disease	Female	418850.6608	380038.6559	457662.6657	2027
Low SDI	Gastroesophageal reflux disease	Female	432238.5269	385376.0069	479101.0469	2028
Low SDI	Gastroesophageal reflux disease	Female	445953.1353	390212.5242	501693.7464	2029
Low SDI	Gastroesophageal reflux disease	Female	459976.0352	394486.8454	525465.225	2030
Low SDI	Gastroesophageal reflux disease	Both	322418.4983	320273.7989	324563.1976	1990
Low SDI	Gastroesophageal reflux disease	Both	330616.9914	328561.3289	332672.654	1991
Low SDI	Gastroesophageal reflux disease	Both	339186.3014	337105.7901	341266.8128	1992
Low SDI	Gastroesophageal reflux disease	Both	347781.1864	345672.9979	349889.3749	1993
Low SDI	Gastroesophageal reflux disease	Both	356809.7327	354673.1968	358946.2686	1994
Low SDI	Gastroesophageal reflux disease	Both	366355.4181	364188.0353	368522.8009	1995
Low SDI	Gastroesophageal reflux disease	Both	376421.2083	374221.2046	378621.212	1996
Low SDI	Gastroesophageal reflux disease	Both	386613.8722	384381.0919	388846.6525	1997
Low SDI	Gastroesophageal reflux disease	Both	396687.5379	394422.763	398952.3128	1998
Low SDI	Gastroesophageal reflux disease	Both	406962.6874	404665.6835	409259.6913	1999
Low SDI	Gastroesophageal reflux disease	Both	417755.3557	415424.8988	420085.8125	2000
Low SDI	Gastroesophageal reflux disease	Both	429469.5146	427103.2115	431835.8178	2001
Low SDI	Gastroesophageal reflux disease	Both	442022.2085	439617.9934	444426.4236	2002
Low SDI	Gastroesophageal reflux disease	Both	454876.3075	452433.8155	457318.7996	2003
Low SDI	Gastroesophageal reflux disease	Both	468119.4002	465638.0213	470600.7792	2004
Low SDI	Gastroesophageal reflux disease	Both	481766.5974	479245.717	484287.4777	2005
Low SDI	Gastroesophageal reflux disease	Both	495768.8173	493208.0288	498329.6057	2006
Low SDI	Gastroesophageal reflux disease	Both	510470.884	507868.7758	513072.9921	2007
Low SDI	Gastroesophageal reflux disease	Both	525761.2043	523116.7304	528405.6781	2008
Low SDI	Gastroesophageal reflux disease	Both	541596.5896	538908.883	544284.2961	2009
Low SDI	Gastroesophageal reflux disease	Both	557928.0857	555196.4503	560659.7212	2010
Low SDI	Gastroesophageal reflux disease	Both	574916.4501	572139.8106	577693.0895	2011
Low SDI	Gastroesophageal reflux disease	Both	592560.4585	589737.7712	595383.1459	2012
Low SDI	Gastroesophageal reflux disease	Both	610749.0774	607879.6077	613618.5471	2013
Low SDI	Gastroesophageal reflux disease	Both	629300.2946	626383.7324	632216.8568	2014
Low SDI	Gastroesophageal reflux disease	Both	648419.1862	645454.4817	651383.8907	2015
Low SDI	Gastroesophageal reflux disease	Both	668095.1765	665080.4928	671109.8603	2016
Low SDI	Gastroesophageal reflux disease	Both	688829.3082	685762.0996	691896.5167	2017
Low SDI	Gastroesophageal reflux disease	Both	710602.7899	707481.8457	713723.7341	2018
Low SDI	Gastroesophageal reflux disease	Both	733159.3858	729870.9894	736447.7822	2019
Low SDI	Gastroesophageal reflux disease	Both	643085.7143	635522.9981	650648.4305	2020
Low SDI	Gastroesophageal reflux disease	Both	664697.488	652030.4836	677364.4924	2021
Low SDI	Gastroesophageal reflux disease	Both	686792.445	667378.5465	706206.3435	2022
Low SDI	Gastroesophageal reflux disease	Both	709504.7281	681893.3506	737116.1057	2023
Low SDI	Gastroesophageal reflux disease	Both	732954.1071	695721.4852	770186.7291	2024
Low SDI	Gastroesophageal reflux disease	Both	757086.9952	708777.6873	805396.3032	2025
Low SDI	Gastroesophageal reflux disease	Both	781816.944	720979.125	842654.7629	2026
Low SDI	Gastroesophageal reflux disease	Both	806999.8505	732169.3021	881830.3988	2027
Low SDI	Gastroesophageal reflux disease	Both	832746.0363	742406.3401	923085.7325	2028
Low SDI	Gastroesophageal reflux disease	Both	859173.6996	751725.1526	966622.2467	2029
Low SDI	Gastroesophageal reflux disease	Both	886221.6585	759982.9546	1012460.362	2030

ɔy global and SDI regions.

Val	Upper	Lower
5067028.188	5594765.382	4526963.76
5092272.485	5625008.92	4609605.704
5123756.476	5636200.847	4620422.284
5144546.382	5656711.11	4614238
5141071.759	5584625.194	4630881.968
5065939.68	5520187.025	4561901.18
4976486.775	5416525.349	4508533.142
4927461.773	5347093.254	4475191.587
4844114.272	5248484.362	4447527.407
4772455.373	5144539.986	4336110.284
4703587.792	5077334.957	4301345.75
4604644.531	4960707.757	4217382.534
4519021.696	4915463.73	4167087.621
4413350.671	4797326.148	4083956.456
4296726.782	4726089.021	3995789.404
4254205.279	4717765.864	3971580.594
4130276.664	4574301.21	3838968.261
4043549.945	4500949.459	3772923.058
4005920.058	4477996.328	3735547.355
3889153.473	4353555.753	3637238.48
3807241.848	4249017.706	3569129.622
3732731.066	4185580.063	3498790.811
3667697.145	4162898.836	3439406.951
3628968.422	4127559.243	3409070.797
3557271.706	3933039.071	3347471.164
3538121.214	3889949.603	3320124.178
3536184.113	3901687.697	3280862.762
3515917.502	3842136.42	3252606.38
3488211.277	3949537.211	3214448.097
3468024.991	3888099.588	3147439.86
NA	NA	NA
NA	NA	NA
NA	NA	NA
NA	NA	NA
NA	NA	NA
NA	NA	NA
NA	NA	NA
NA	NA	NA
NA	NA	NA
NA	NA	NA
NA	NA	NA
3129035.802	3670228.887	2691251.13
3134488.5	3692821.1	2702829.277
3179321.613	3700857.201	2730521.981
3161672.067	3645712.498	2763506.559
3149189.36	3645590.694	2770353.826
3118920.053	3574306.121	2752782.463
3097406.745	3526862.563	2735510.043
3136451.027	3615178.505	2768069.046
3109991.874	3571298.538	2750723.392
3085639.758	3487298.688	2727946.749
3071632.206	3494063.35	2720947.43
3044249.093	3438489.777	2723687.096
3010731.758	3394003.476	2698642.904
2963984.072	3342703.47	2669554.929
2892180.256	3214689.877	2635386.986
2881026.185	3215678.635	2618874.381
2824423.915	3131381.533	2565991.835
2758836.535	3044941.396	2504078.137
2716911.694	2986863.645	2491594.225

2637397.534	2895208.147	2413960.864
2610629.103	2850459.682	2388421.768
2579472.86	2794407.082	2360732.665
2532565.612	2757245.369	2320475.814
2532650.028	2748667.816	2306744.133
2507064.847	2738766.27	2282239.014
2519673.02	2723709.565	2300871.4
2537019.691	2770404.639	2304983.253
2559867.067	2801783.875	2315416.336
2560047.571	2830908.82	2309259.875
2561484.864	2851924.087	2289099.967
NA	NA	NA
NA	NA	NA
NA	NA	NA
NA	NA	NA
NA	NA	NA
NA	NA	NA
NA	NA	NA
NA	NA	NA
NA	NA	NA
NA	NA	NA
NA	NA	NA
8196063.99	8965447.061	7581034.837
8226760.985	8988020.481	7602251.789
8303078.088	8995029.208	7710292.929
8306218.448	8971850.882	7735198.046
8290261.119	8904961.825	7724575.557
8184859.734	8789455.69	7651995.818
8073893.52	8665961.487	7546832.803
8063912.8	8642472.201	7525511.127
7954106.145	8552776.427	7438942.822
7858095.132	8424827.043	7349946.904
7775219.998	8354673.883	7277303.404
7648893.624	8213422.047	7152053.006
7529753.455	8099480.422	7066590.534
7377334.743	7968656.299	6936614.891
7188907.037	7777395	6771289.143
7135231.465	7698004.198	6716108.83
6954700.579	7569953.402	6536028.163
6802386.48	7405475.81	6386715.13
6722831.752	7319295.247	6304013.956
6526551.007	7134010.357	6130158.022
6417870.951	7005881.185	6027907.443
6312203.927	6950017.722	5931869.934
6200262.757	6786422.25	5836319.446
6161618.45	6784897.348	5786157.79
6064336.553	6583705.944	5709753.753
6057794.234	6615477.889	5708124.58
6073203.804	6580016.189	5674482.253
6075784.569	6591991.919	5645386.473
6048258.848	6634810.889	5586886.185
6029509.855	6641773.454	5586597.711
NA	NA	NA
NA	NA	NA
NA	NA	NA
NA	NA	NA
NA	NA	NA
NA	NA	NA
NA	NA	NA
NA	NA	NA
NA	NA	NA
NA	NA	NA

NA	NA	NA
428727.2381	448255.3065	411183.2435
420234.1295	438844.429	402789.0425
413078.5549	431301.3675	394741.1365
409833.8384	426858.286	391889.6951
401896.0503	419477.3394	384416.4691
394675.1368	411543.0305	377664.4071
380029.3777	396154.3048	363381.7326
365491.9296	380886.2614	349650.3524
354180.9555	369314.6472	339556.6396
341328.7641	355715.6524	326729.9823
325827.2223	340434.9817	311568.1288
313748.01	328510.1118	299116.1841
305625.3355	320555.2158	291722.6396
298142.0182	312907.4923	284087.1477
286845.9761	302105.1115	273102.4991
281244.6	296417.8498	267431.9462
273234.6756	288216.6479	259162.081
266659.6285	281947.1535	252581.4014
261022.9178	276470.2374	246950.6668
255291.6905	270402.3798	240596.3984
249997.8725	265160.3965	235714.8675
246250.3262	262165.3148	231794.501
242275.7627	258269.5665	227892.7472
239714.5894	255431.3193	224663.6265
236606.128	252557.507	221209.1405
237837.2001	253537.096	222085.9379
239024.8982	255347.0814	222513.6884
239274.1969	256268.5386	221744.2038
242089.8544	260772.8593	224547.6058
243368.9255	262054.8177	224984.7198
NA	NA	NA
NA	NA	NA
NA	NA	NA
NA	NA	NA
NA	NA	NA
NA	NA	NA
NA	NA	NA
NA	NA	NA
NA	NA	NA
NA	NA	NA
NA	NA	NA
314131.3194	330635.3384	290515.3666
310051.0231	326647.0823	286939.3739
305834.4609	321657.8145	282200.5469
305919.3343	322912.8132	281352.0707
300207.9598	317300.5806	275268.7765
297086.5226	314109.6289	272550.4415
288925.2352	305261.3568	264202.7293
280235.2388	296489.8345	256015.5644
271062.3679	286858.882	247967.6757
261422.1378	277525.5441	239215.6725
249325.7038	265238.4258	227768.2026
240157.3986	256658.859	218983.9223
233392.2201	250270.3788	212567.9877
227428.4515	243753.5197	207072.9972
218267.6054	234847.9609	197751.9244
212459.8643	228991.9463	191883.5591
205891.0277	223069.838	185797.9859
200685.6966	217359.458	180386.1667
196554.7846	213631.3158	176390.7921
191378.3723	208878.2279	171172.1914

187156.9797	204723.1518	166881.9703
184993.1391	202239.9697	164413.2173
182366.8222	199693.4531	161865.9551
180235.2369	197362.609	159676.653
177334.5083	194686.0482	157114.182
178303.1501	195394.65	158047.3789
180156.6373	197331.2661	158585.1563
181433.3976	198758.4216	159608.0009
185031.5218	203152.4655	162510.7949
187952.1734	206716.4583	165181.4217
NA	NA	NA
NA	NA	NA
NA	NA	NA
NA	NA	NA
NA	NA	NA
NA	NA	NA
NA	NA	NA
NA	NA	NA
NA	NA	NA
NA	NA	NA
NA	NA	NA
742858.5575	772001.7768	704263.9458
730285.1526	758746.272	692586.4913
718913.0158	747337.8161	680822.5064
715753.1727	744691.7045	676746.244
702104.0101	730670.6528	662790.6157
691761.6595	720274.0119	652529.046
668954.6129	697742.6156	630054.2858
645727.1684	673980.674	607422.2895
625243.3233	652722.8157	589030.4538
602750.9019	629803.3311	567512.6461
575152.9261	602176.3217	540428.906
553905.4086	581475.1599	520378.1595
539017.5557	566665.6248	505806.4928
525570.4697	553173.6864	493218.5883
505113.5816	533414.6244	472119.1043
493704.4643	521613.79	460072.5392
479125.7033	507376.6947	446083.4807
467345.325	496412.0533	434443.7437
457577.7024	486876.1572	424321.9349
446670.0628	475964.4383	414181.4698
437154.8522	466254.4985	404317.7828
431243.4653	461286.3457	398020.0541
424642.5848	454205.3994	391274.4337
419949.8263	449963.4391	386533.759
413940.6363	444622.0137	380274.2638
416140.3502	447223.7132	382164.1723
419181.5356	451359.6513	383015.7309
420707.5946	453785.3617	383321.5581
427121.3762	461497.354	389001.1317
431321.0989	465696.6842	391452.2512
NA	NA	NA
NA	NA	NA
NA	NA	NA
NA	NA	NA
NA	NA	NA
NA	NA	NA
NA	NA	NA
NA	NA	NA
NA	NA	NA
NA	NA	NA
NA	NA	NA

834274.9586	917925.8398	764728.4566
839407.6353	930045.9915	772199.9768
869264.7616	949780.1334	806131.9351
911400.5264	985016.0656	848035.0474
933334.4705	1011115.855	872430.8383
914391.7533	986439.7134	853567.4797
866370.5316	928909.3146	815150.145
821583.3709	877860.0571	775788.5787
793731.9882	848695.7851	752921.9779
799411.9687	851452.557	758672.2061
796865.8553	848819.8392	753158.4091
780259.6155	827452.2945	736493.1495
771652.5426	822468.9062	732152.7998
767060.8042	813694.7107	725955.8177
755095.2433	802371.6851	717367.8257
756978.6487	804455.4135	719509.5992
708929.0456	748352.4287	678052.4174
682871.603	718931.2742	653540.6912
675126.3187	711042.3547	645447.469
648231.6915	683326.564	620229.4958
641816.9458	678440.6042	612607.1031
619554.2881	658479.15	589770.3806
609657.0527	647377.6126	578945.2574
599680.285	636233.4721	567026.7134
597171.3033	632929.2612	563044.2288
605321.3111	644028.5662	568479.2585
600351.4188	640567.4143	561602.842
580324.2315	623551.8898	540578.7167
567929.5828	621216.2023	522762.6855
563802.6968	619840.6649	508223.3415
NA	NA	NA
NA	NA	NA
NA	NA	NA
NA	NA	NA
NA	NA	NA
NA	NA	NA
NA	NA	NA
NA	NA	NA
NA	NA	NA
NA	NA	NA
NA	NA	NA
370247.848	413169.1525	336373.9272
368952.9837	413578.9745	336159.761
371460.9278	411126.8095	341798.8755
380353.239	418804.4783	351420.1296
385181.1067	418406.719	357039.5494
377426.232	409774.8062	351109.3879
364473.3547	395020.84	340902.7212
354634.2649	381804.3853	332003.4208
346557.7946	372715.89	322568.823
347581.0878	373417.9607	325289.1371
348189.1039	373456.2393	326358.2717
347426.1869	371513.0978	326858.4567
349246.0774	374294.259	327958.4385
350891.2723	375356.6802	328309.8472
347786.6036	370607.2317	326398.1545
348900.6198	372327.7852	326386.1149
335795.6743	356906.188	314848.4828
329338.2623	349396.2456	307802.2738
326040.7067	344935.4264	305088.0975
320158.5148	339589.3184	298863.5642
319150.8436	338717.6001	299005.4873

315082.4941	334083.4738	293388.6204
312744.6027	331743.797	289363.2138
312262.1288	332297.6767	289795.67
313158.5584	332752.1768	292075.3086
318678.5103	339670.1778	294770.5916
319186.9711	340692.0252	294656.4645
316091.9501	339481.769	289916.0238
313262.3039	340070.8282	284965.6638
312211.6531	344333.1292	279658.6225
NA	NA	NA
NA	NA	NA
NA	NA	NA
NA	NA	NA
NA	NA	NA
NA	NA	NA
NA	NA	NA
NA	NA	NA
NA	NA	NA
NA	NA	NA
NA	NA	NA
1204522.807	1303365.976	1118917.817
1208360.619	1316556.81	1129664.731
1240725.689	1334275.567	1164501.703
1291753.765	1381804.739	1218537.014
1318515.577	1402934.325	1240584.514
1291817.985	1369228.096	1219456.514
1230843.886	1299879.168	1166549.389
1176217.636	1242826.236	1118417.779
1140289.783	1208425.894	1084490.123
1146993.056	1211447.049	1095072.368
1145054.959	1202423.489	1087598.677
1127685.802	1187422.423	1072553.17
1120898.62	1185316.924	1067789.019
1117952.077	1174898.544	1066444.136
1102881.847	1161196.822	1049466.327
1105879.268	1166415.511	1055766.77
1044724.72	1099896.899	999114.7382
1012209.865	1062956.747	968848.6005
1001167.025	1050123.674	958655.2678
968390.2064	1016879.976	925825.4178
960967.7894	1007179.182	917622.7243
934636.7823	987935.4681	889060.7469
922401.6554	973113.5837	875650.0083
911942.4138	959282.4275	865030.0921
910329.8617	960001.3856	864850.1401
923999.8214	971400.1742	875760.3847
919538.3899	969417.9816	871112.1097
896416.1817	949554.8739	842898.9388
881191.8867	945564.4716	820104.1497
876014.3499	942390.2934	810891.5116
NA	NA	NA
NA	NA	NA
NA	NA	NA
NA	NA	NA
NA	NA	NA
NA	NA	NA
NA	NA	NA
NA	NA	NA
NA	NA	NA
NA	NA	NA
NA	NA	NA
1219599.848	1419979.817	1049371.583

1221410.78	1420970.065	1076396.476
1219347.184	1397668.408	1085115.768
1210215.771	1381917.76	1080438.852
1201045.262	1374696.426	1075008.851
1186948.023	1372194.86	1068083.019
1171995.239	1339784.56	1068462.948
1145761.142	1300245.047	1049843.232
1121131.65	1265335.02	1033479.929
1105041.477	1243880.928	1018160.219
1096516.546	1230967.916	1010853.132
1082525.771	1216204.406	998393.9201
1079988.012	1233496.483	1003673.933
1073566.927	1211021.859	995075.9679
1066119.398	1225562.018	992035.5162
1054600.16	1241921.207	983236.4378
1027063.241	1203178.071	954174.9908
1005266.239	1169903.425	939418.1459
1001400.397	1164048.4	936231.0064
986146.5929	1148394.622	922899.0698
972307.1466	1127213.251	909869.9268
960270.2242	1112328.7	895922.205
943232.4565	1090746.502	875151.1162
929759.9693	1073117.659	861125.5011
911038.8356	1038655.721	848304.0243
898221.1978	1006731.279	832248.3684
896980.8709	1017912.508	825101.4826
887466.1107	995617.2196	811401.7401
875392.7074	1005531.829	788218.7108
869825.0055	1007284.099	782951.6359
NA	NA	NA
NA	NA	NA
NA	NA	NA
NA	NA	NA
NA	NA	NA
NA	NA	NA
NA	NA	NA
NA	NA	NA
NA	NA	NA
NA	NA	NA
NA	NA	NA
716501.0471	851456.8843	635624.9734
711821.6842	848307.0057	635431.1198
718842.8525	854654.4517	646093.932
706788.0925	831058.8149	639782.4249
703034.1584	823144.2991	638936.408
692261.6248	804132.7314	630324.7761
676526.6468	775296.3364	616164.7098
671054.2968	768759.3535	618828.5965
664164.5947	758866.1628	612319.2685
659977.0895	745455.8829	607221.1103
659249.7082	743380.8439	606576.8099
653590.5583	731611.0374	604115.7008
653016.1271	729127.0546	603745.3826
648751.5155	720522.5944	600030.1444
641808.0542	707137.2508	596049.5557
632571.2843	696211.7968	586141.651
616986.1277	678005.1001	571893.5051
601775.0899	657142.5592	557688.7409
593448.2117	647826.617	553721.7648
583430.7895	633286.1388	544453.7982
577669.9733	628679.5693	539347.4602
570847.2116	616874.3281	527208.4834

562038.7532	611525.1008	522952.1413
560599.7433	604560.6713	518944.2989
551732.5766	600188.8445	507819.2739
545592.3289	590069.5598	500939.6682
548387.4304	600457.997	501322.8395
552086.1212	610424.8212	497275.8687
553459.1216	615859.2697	494368.5061
556215.6944	624982.3183	486830.8218
NA	NA	NA
NA	NA	NA
NA	NA	NA
NA	NA	NA
NA	NA	NA
NA	NA	NA
NA	NA	NA
NA	NA	NA
NA	NA	NA
NA	NA	NA
NA	NA	NA
1936100.895	2177436.41	1750648.423
1933232.464	2187579.028	1758921.848
1938190.037	2173669.979	1777669.131
1917003.864	2139320.201	1765892.458
1904079.421	2122869.546	1748512.175
1879209.648	2091808.843	1735252.588
1848521.885	2028460.923	1720491.406
1816815.439	2007526.643	1694112.172
1785296.244	1958095.473	1667282.899
1765018.567	1924011.657	1654728.111
1755766.254	1929494.356	1643681.35
1736116.33	1905076.866	1622061.524
1733004.139	1915019.425	1621039.198
1722318.442	1887933.607	1616158.499
1707927.452	1906303.15	1609129.554
1687171.444	1889563.206	1591305.729
1644049.369	1844548.767	1546484.239
1607041.329	1790988.106	1515573.544
1594848.609	1787708.012	1504578.02
1569577.382	1766031.579	1484425.373
1549977.12	1733717.554	1463571.459
1531117.436	1713407.969	1441950.585
1505271.21	1682410.824	1421771.111
1490359.713	1669179.68	1398361.84
1462771.412	1615398.759	1374190.978
1443813.527	1563866.458	1356614.99
1445368.301	1593166.43	1345097.288
1439552.232	1583735.532	1338473.281
1428851.829	1589189.886	1311732.562
1426040.7	1583778.119	1313180.137
NA	NA	NA
NA	NA	NA
NA	NA	NA
NA	NA	NA
NA	NA	NA
NA	NA	NA
NA	NA	NA
NA	NA	NA
NA	NA	NA
NA	NA	NA
NA	NA	NA
1950474.228	2191570.414	1655648.761
1968093.905	2223687.729	1673973.25

1972043.368	2226585.692	1668437.543
1957562.909	2194788.18	1665981.135
1941674.995	2161512.87	1647500.666
1907294.859	2122129.342	1641989.442
1891065.167	2098039.624	1623102.023
1917554.581	2120970.2	1660574.146
1894163.081	2100127.417	1667551.344
1844709.247	2033771.082	1592719.106
1801907.894	1973057.409	1592269.889
1756463.702	1925282.488	1531404.91
1702600.096	1874441.248	1529717.447
1621039.299	1775377.705	1459202.31
1549756.852	1716984.727	1411424.544
1535635.683	1717942.637	1409423.516
1503181.087	1674901.196	1375224.68
1460842.1	1650893.817	1335558.274
1450449.797	1678060.474	1322313.494
1388256.534	1589772.437	1273188.564
1345413.496	1542325.975	1235889.164
1315367.678	1536929.056	1210339.488
1286253.354	1517138.334	1172822.264
1266872.82	1495049.516	1157687.317
1229151.3	1430412.317	1121060.782
1209249.779	1391321.232	1103850.415
1213775.039	1421908.619	1085546.393
1219261.226	1397024.099	1090098.178
1213332.117	1438471.049	1060802.974
1204516.852	1424664.43	1036432.275
NA	NA	NA
NA	NA	NA
NA	NA	NA
NA	NA	NA
NA	NA	NA
NA	NA	NA
NA	NA	NA
NA	NA	NA
NA	NA	NA
NA	NA	NA
NA	NA	NA
1265287.58	1531748.901	1029881.047
1273189.879	1542419.205	1034434.239
1301324.832	1571188.096	1037712.226
1285745.52	1528447.354	1057631.341
1276278.774	1523855.49	1076915.589
1267315.99	1492488.459	1064598.095
1279244.691	1501815.32	1076810.088
1334926.635	1596271.339	1120696.614
1326437.464	1577126.336	1117858.752
1311519.084	1527718.073	1118759.88
1307339.771	1532136.348	1116527.61
1289970.382	1500039.667	1106056.043
1260526.575	1457270.604	1091825.014
1224511.009	1421651.542	1071552.978
1175193.364	1349655.718	1039668.164
1175456.736	1349850.057	1035632.466
1159532.758	1316198.88	1020327.6
1129788.245	1276781.088	993419.832
1105528.49	1246796.141	985110.3136
1052403.405	1176657.77	940566.2358
1043388.004	1161912.545	926072.186
1033093.109	1155273.171	912931.8745
998028.9143	1112021.846	891165.2798

1004074.908	1124124.837	895381.0884
981048.8559	1099106.225	873297.1396
993805.2962	1110861.963	885909.004
1003544.208	1135548.593	889536.9134
1018712.453	1153434.372	888881.7759
1015375.568	1178123.683	883021.2929
1012357.122	1187305.574	859758.5628
NA	NA	NA
NA	NA	NA
NA	NA	NA
NA	NA	NA
NA	NA	NA
NA	NA	NA
NA	NA	NA
NA	NA	NA
NA	NA	NA
NA	NA	NA
NA	NA	NA
3215761.809	3578487.768	2881445.852
3241283.784	3597580.578	2896648.724
3273368.2	3625064.241	2938067.136
3243308.429	3565183.894	2925010.1
3217953.768	3529022.558	2901779.523
3174610.849	3470027.976	2894279.138
3170309.858	3470492.294	2886934.934
3252481.216	3579013.137	2917631.398
3220600.545	3532130.665	2920504.453
3156228.331	3441416.406	2859683.871
3109247.664	3397809.174	2832505.557
3046434.084	3321638.279	2786083.534
2963126.671	3245455.531	2717706.461
2845550.308	3113424.604	2618211.681
2724950.217	2957986.438	2525688.703
2711092.42	2991429.048	2504825.001
2662713.845	2953164.259	2464582.436
2590630.345	2875878.21	2395282.937
2555978.287	2844815.196	2350254.922
2440659.939	2707330.061	2259550.79
2388801.5	2662054.141	2202941.81
2348460.787	2624243.689	2168649.488
2284282.269	2584362.435	2108210.405
2270947.728	2564720.257	2092527.317
2210200.156	2477910.823	2036078.594
2203055.075	2447665.701	2031232.763
2217319.247	2508937.914	2028025.979
2237973.678	2517251.89	2031125.677
2228707.685	2560109.352	2004751.391
2216873.974	2550605.822	1988317.047
NA	NA	NA
NA	NA	NA
NA	NA	NA
NA	NA	NA
NA	NA	NA
NA	NA	NA
NA	NA	NA
NA	NA	NA
NA	NA	NA
NA	NA	NA
NA	NA	NA
631463.4015	751765.6788	513039.2669
640651.2132	763644.1485	511014.7624
647535.1577	773274.7091	511797.6306

653033.9099	777515.9627	526958.4763
660638.1529	781245.4088	531596.7361
660164.8107	785160.4045	531272.3807
664571.8959	791164.1311	535726.4971
674627.0298	800129.8285	546174.0244
678471.6406	808495.767	543091.7438
679538.5576	809857.1305	548435.7573
680098.9326	810895.9752	548008.8207
669318.6929	799620.7934	536639.7726
656877.0243	785319.4316	534923.4796
651273.3234	783175.0718	532723.5602
636647.48	768043.6712	522063.7277
623494.224	749265.1513	506637.6488
615631.4096	735281.3641	508219.9814
625672.9079	751869.9598	518224.0992
615685.3339	738224.8431	510343.6071
609007.439	727873.8363	509837.8348
595513.2735	706580.2806	499469.6937
589104.0251	699369.121	495640.7317
584095.3455	686613.2681	492524.654
590748	688118.1244	505090.1187
581089.2762	673277.4694	498171.6282
585256.5552	677942.2109	496209.8783
583805.6906	685726.3323	493091.4364
587332.7851	684973.0532	492140.1146
587209.9801	686383.1526	496817.6467
584260.9927	682744.6123	492551.9201
NA	NA	NA
NA	NA	NA
NA	NA	NA
NA	NA	NA
NA	NA	NA
NA	NA	NA
NA	NA	NA
NA	NA	NA
NA	NA	NA
NA	NA	NA
NA	NA	NA
461451.5222	572626.5757	340964.1946
469056.7476	586989.6755	346693.6144
480427.6256	608511.2664	356913.4201
481421.4208	594138.3697	359692.713
483050.4658	594428.3062	363802.5146
483399.2214	600323.0868	365523.2422
486809.7135	595425.2005	375049.6829
494177.2119	606025.5985	386155.9922
500343.4741	616208.8982	386300.1683
503714.586	615484.8777	392478.5102
506117.4716	618033.0374	396106.0946
511704.2119	623956.6006	402518.1983
513168.1199	621342.1185	405715.1863
511015.0568	614864.9306	412968.9253
507737.221	606307.1101	413611.0968
510251.1636	609823.4442	415566.5314
504843.3062	599676.1318	413948.3262
495884.5162	579236.4482	411703.5084
493986.6629	578488.764	414695.5921
488692.3455	570623.4397	406339.3571
481939.2504	559801.0943	405114.9147
474143.3448	549285.7271	400389.6926
476073.7019	551924.4735	402559.0135
474165.0461	547173.6262	398930.3279

482470.9279	556374.0166	406442.8235
481961.6852	552194.2775	409256.9438
484406.3512	557370.4805	412187.4425
490191.7774	562894.46	418641.8531
491562.8758	563789.7549	417643.6514
491392.0868	568959.1177	419082.1873
NA	NA	NA
NA	NA	NA
NA	NA	NA
NA	NA	NA
NA	NA	NA
NA	NA	NA
NA	NA	NA
NA	NA	NA
NA	NA	NA
NA	NA	NA
NA	NA	NA
1092914.924	1263157.475	922628.8344
1109707.961	1279686.274	929923.2159
1127962.783	1292313.914	935282.0776
1134455.331	1298578.755	949897.897
1143688.619	1306831.076	968268.6273
1143564.032	1309462.175	970252.9332
1151381.609	1320143.16	985952.6881
1168804.242	1334024.327	1000932.172
1178815.115	1345414.329	1007044.508
1183253.144	1350374.748	1018764.086
1186216.404	1346798.079	1014250.839
1181022.905	1347051.85	1007417.665
1170045.144	1337629.495	1004531.711
1162288.38	1338325.18	998221.3349
1144384.701	1315200.739	986469.7928
1133745.388	1307358.972	984101.7983
1120474.716	1281658.921	976445.9345
1121557.424	1286377.354	981344.3508
1109671.997	1278633.247	968824.5405
1097699.784	1256479.489	963372.609
1077452.524	1236172.919	943752.8455
1063247.37	1206045.602	931256.0349
1060169.047	1202866.673	932392.7423
1064913.046	1209055.031	934384.1207
1063560.204	1203575.776	934076.8077
1067218.24	1204231.075	938092.2528
1068212.042	1208042.876	940659.987
1077524.563	1220140.318	941572.6989
1078772.856	1221949.81	946966.3877
1075653.08	1219387.174	948157.2632
NA	NA	NA
NA	NA	NA
NA	NA	NA
NA	NA	NA
NA	NA	NA
NA	NA	NA
NA	NA	NA
NA	NA	NA
NA	NA	NA
NA	NA	NA
NA	NA	NA
1144978.942	1532205.527	851868.6365
1150902.242	1533210.538	859749.6188
1154043.387	1530830.309	865756.9089
1155662.532	1522950.684	872407.5352

1158411.191	1517194.738	881265.2339
1161232.335	1520680.59	886868.7067
1160763.686	1515255.906	887690.025
1158808.088	1514884.101	893302.0309
1161139.709	1510530.668	894934.9473
1166904.272	1515425.026	903874.0211
1178238.863	1518928.963	911275.5961
1186553.005	1537967.414	918306.5299
1199351.588	1554062.652	938228.5517
1211780.484	1558881.44	947510.4254
1222257.605	1566573.476	963338.8595
1228285.536	1573307.414	970619.0101
1230654.368	1583894.25	964302.4312
1229083.589	1573646.974	964975.3561
1232997.754	1582899.806	966591.181
1235745.182	1574595.666	962691.7503
1240063.751	1598080.998	968910.1142
1248945.517	1616050.504	970320.9539
1259680.528	1623378.455	970826.0887
1273060.983	1642722.833	976953.38
1282932.164	1655099.634	981944.1011
1298128.954	1684991.411	1002214.047
1322149.768	1713732.977	1011081.333
1341254.801	1744260.469	1012821.413
1341481.078	1748876.089	1010231.746
1343976.82	1756754.059	1010421.735
NA	NA	NA
NA	NA	NA
NA	NA	NA
NA	NA	NA
NA	NA	NA
NA	NA	NA
NA	NA	NA
NA	NA	NA
NA	NA	NA
NA	NA	NA
NA	NA	NA
1070707.12	1429028.847	785451.7569
1071710.402	1447724.195	776875.3505
1074345.225	1463877.55	777652.5973
1074779.526	1444314.564	779241.0531
1080716.375	1452556.475	783004.6581
1085663.411	1472474.227	793876.0568
1091104.911	1466986.131	791339.6112
1100651.548	1488247.485	800918.6138
1109454.096	1504097.741	805861.2347
1121222.034	1521080.907	814224.6138
1137370.473	1547858.733	829953.6466
1151543.319	1556394.859	834118.3025
1168957.247	1587388.368	850544.6055
1187523.187	1602853.74	873744.4268
1203962.999	1627540.407	886422.4713
1218253.226	1639069.711	894855.4516
1229604.211	1660252.293	902348.921
1239001.43	1675521.482	912236.1055
1254099.652	1702903.585	915167.3061
1269471.395	1730975.774	928126.3681
1285597.424	1758061.917	934390.6934
1300577.924	1779647.943	947879.7924
1313545.996	1794825.384	950949.3994
1330957.047	1824861.572	956769.8326
1348770.964	1849858.061	966039.2568

1371013.109	1878470.231	989850.6916
1404233.655	1932204.431	1005266.228
1438835.663	1983900.278	1038093.65
1456794.858	2013693.282	1037637.588
1470912.869	2032509.904	1046258.776
NA	NA	NA
NA	NA	NA
NA	NA	NA
NA	NA	NA
NA	NA	NA
NA	NA	NA
NA	NA	NA
NA	NA	NA
NA	NA	NA
NA	NA	NA
NA	NA	NA
2215686.063	2918865.254	1692074.286
2222612.644	2948209.583	1702590.939
2228388.612	2951549.914	1712919.075
2230442.057	2952999.85	1703321.595
2239127.566	2975548.557	1715172.066
2246895.747	2980668.265	1732659.894
2251868.597	2986054.365	1732010.689
2259459.636	3013354.619	1746228.358
2270593.805	3040942.959	1747834.855
2288126.305	3028251.245	1758479.404
2315609.336	3084276.684	1767771.57
2338096.324	3107051.28	1792978.144
2368308.835	3151364.919	1814621.946
2399303.67	3179385.977	1842395.313
2426220.604	3206137.847	1868491.716
2446538.762	3216784.493	1877172.658
2460258.579	3265330.787	1885906.3
2468085.019	3268278.559	1892379.217
2487097.406	3289724.137	1896262.761
2505216.577	3320458.984	1908946.04
2525661.175	3359126.216	1927143.982
2549523.441	3392770.414	1930079.253
2573226.525	3433401.928	1946409.128
2604018.031	3480942.556	1971256.446
2631703.128	3529770.174	1984918.035
2669142.064	3593086.165	2017810.794
2726383.423	3651012.422	2044716.747
2780090.464	3747465.327	2077344.341
2798275.936	3772166.589	2088394.09
2814889.689	3794394.811	2087791.298
NA	NA	NA
NA	NA	NA
NA	NA	NA
NA	NA	NA
NA	NA	NA
NA	NA	NA
NA	NA	NA
NA	NA	NA
NA	NA	NA
NA	NA	NA
NA	NA	NA
87044.70454	120407.4191	62410.35147
86492.91796	119659.8253	61608.06791
85742.79079	118995.883	61432.55417
85409.23126	118263.8689	61156.58337
85284.90336	117823.1217	60967.26491

85165.92305	117754.5999	60601.03293
84586.74887	116464.0131	60317.72227
84288.27455	116843.3337	59738.5624
84123.19902	117379.6207	59672.42427
83884.4826	117160.6937	58972.20315
84115.51933	117523.6463	58991.25629
83943.78113	117888.3311	58628.16049
83993.05001	117177.9946	58677.31108
83784.13889	116981.7606	58559.88348
83135.60489	116117.0841	57922.86624
83056.79231	115741.7226	57570.87849
82973.1537	115702.0859	57880.7975
82775.71813	114712.0189	57912.09282
82728.4375	114603.2127	57801.47808
82874.95977	114269.6638	57848.09047
83328.97876	114811.6923	58597.9833
84010.80279	116042.1924	58835.55977
84893.81233	117061.3449	59194.1258
86065.19769	119011.1102	60365.8095
87013.83594	120169.3559	60553.86737
88254.93859	121869.3473	61289.69984
89720.47491	124002.4721	62427.64341
90977.8087	126011.2039	63202.05169
92165.01675	127666.9767	64285.75809
93247.73392	129398.1461	64678.13986
NA	NA	NA
NA	NA	NA
NA	NA	NA
NA	NA	NA
NA	NA	NA
NA	NA	NA
NA	NA	NA
NA	NA	NA
NA	NA	NA
NA	NA	NA
NA	NA	NA
120479.1876	172242.5554	81658.78031
118585.9288	169449.0723	80354.80435
116945.9982	166269.2539	79447.91125
115917.8977	163698.8423	78462.41632
115358.5579	163140.3736	78424.82049
115587.5942	162434.5964	78620.21204
115895.8015	163842.3053	78954.94518
116171.2349	164043.459	78771.77242
116576.6775	164044.2262	79115.77135
116907.3174	165688.337	78952.44516
116664.0456	164216.371	78942.92965
116136.4396	164930.1233	77925.71371
115294.1523	164041.3279	77179.61283
114397.2574	162452.7579	76716.42713
113357.3311	161145.1354	75660.25032
113141.9049	159341.5003	76056.84828
113075.6214	160760.065	76177.76313
113109.6211	160615.2793	76079.05937
113430.7573	161072.3914	76555.32071
113860.0073	160015.6394	76413.70434
114718.1879	161601.3423	77679.07552
116074.059	163035.8381	78541.33183
117629.9772	165633.0623	79388.06387
119166.6758	167514.2744	79866.29213
120704.8729	169877.4458	80739.49245
122574.2017	172285.2463	81833.17146

124505.8397	175058.9361	82757.50478
126299.1245	178755.7597	85147.74258
128153.857	182228.0825	85244.06883
130125.0363	185424.3723	86301.42935
NA	NA	NA
NA	NA	NA
NA	NA	NA
NA	NA	NA
NA	NA	NA
NA	NA	NA
NA	NA	NA
NA	NA	NA
NA	NA	NA
NA	NA	NA
NA	NA	NA
207523.8921	292352.0553	142999.5643
205078.8467	288106.7333	141751.2406
202688.789	284133.0474	140435.8629
201327.129	281570.9015	139902.9511
200643.4613	280571.9445	139779.507
200753.5173	279480.9426	139928.1923
200482.5504	280953.2499	139049.0665
200459.5095	280780.5339	138561.2107
200699.8765	281237.2329	138540.4607
200791.8	282898.0697	138312.5484
200779.565	280977.4045	137691.9718
200080.2207	282126.5443	137550.9617
199287.2023	280169.5722	136497.6209
198181.3963	278437.9188	135572.5502
196492.936	276777.3069	134255.0886
196198.6972	275209.9761	134280.9489
196048.7751	275356.9829	134168.7757
195885.3392	274487.5129	134014.6411
196159.1948	275037.5253	134741.8651
196734.9671	274807.0966	134739.1197
198047.1666	276502.5262	136100.2902
200084.8618	279974.8341	137340.6942
202523.7896	283723.5923	138493.0334
205231.8735	288557.7734	140263.9576
207718.7088	290704.4575	142035.4887
210829.1403	294925.5135	143991.9487
214226.3146	298972.96	146230.2431
217276.9332	304426.0251	147189.6006
220318.8737	309084.2513	150198.6574
223372.7702	314252.7492	152044.0092
NA	NA	NA
NA	NA	NA
NA	NA	NA
NA	NA	NA
NA	NA	NA
NA	NA	NA
NA	NA	NA
NA	NA	NA
NA	NA	NA
NA	NA	NA
NA	NA	NA
220089.7125	298962.6413	159618.0714
220450.7336	301642.8074	160152.1277
221331.7429	298371.3205	162792.8414
222019.9264	299429.125	163904.1016
222658.346	299230.8539	166727.7407
222464.8574	298776.6371	165509.8286

220477.3886	298018.4622	163623.9314
217827.5963	292288.4065	162628.0684
216759.0909	289277.1147	161230.5408
218119.6572	293353.1067	163700.2037
220707.0763	292336.7866	165439.065
222246.5791	296364.034	166853.4769
224647.8636	299907.222	169002.4551
227025.1215	300445.4176	173048.2212
228623.4084	302516.7937	175587.6149
228787.1957	303749.4224	174464.0203
224571.3526	299307.2903	169908.7255
219166.3466	292423.8417	166957.4096
215360.5774	287072.4205	162930.5454
211488.5579	281782.8547	158872.5964
210875.8082	281671.8938	159084.4049
210955.347	282650.5171	157871.3574
211964.513	283783.5617	158062.5675
213180.067	287254.6581	159191.675
214494.6673	287205.5769	159499.4324
216626.3955	291550.468	160497.8564
219411.8893	296656.4319	160980.0802
221159.4779	297270.2282	162178.4537
221427.529	299033.2075	159954.2099
222439.3545	299038.207	162277.5309
NA	NA	NA
NA	NA	NA
NA	NA	NA
NA	NA	NA
NA	NA	NA
NA	NA	NA
NA	NA	NA
NA	NA	NA
NA	NA	NA
NA	NA	NA
NA	NA	NA
223462.5858	310681.9944	154851.6195
222895.2685	310998.655	155997.5352
222332.4195	308364.7298	155682.3865
222493.1577	309290.8388	155105.2872
222992.5125	312614.4358	156706.1592
223166.5897	310812.7225	156655.5447
223286.478	313098.6497	154962.6065
223812.0574	315250.1371	155137.5735
224445.4111	315564.4867	155594.2901
226486.645	319231.5622	155234.0175
229052.7967	323300.8163	158447.7781
231585.4312	325012.068	160148.9043
235236.5483	328535.7254	164840.4633
239060.7817	334458.4841	166672.561
242348.0778	340109.452	168836.2496
244010.8932	341278.7794	168533.5479
243642.7319	341546.6482	169231.8328
241898.8417	337792.4987	167916.5104
240219.5578	335028.2457	165463.4257
239466.6519	337868.416	165530.7454
240044.6661	335638.5088	165448.2888
241258.5599	340568.4468	164362.1848
242550.2041	342432.0789	167037.6502
244528.3354	343015.9068	167316.6981
247185.61	347883.8057	167815.5779
250641.0442	352837.7119	171212.3561
256549.423	363478.8161	173342.6428

262628.4336	374359.2485	177375.9959
265809.4215	377851.1856	179811.2553
268400.0794	383721.98	181272.7543
NA	NA	NA
NA	NA	NA
NA	NA	NA
NA	NA	NA
NA	NA	NA
NA	NA	NA
NA	NA	NA
NA	NA	NA
NA	NA	NA
NA	NA	NA
NA	NA	NA
443552.2983	605499.0291	323310.2123
443346.0021	604206.2466	324270.0734
443664.1624	603554.3973	321769.2041
444513.0841	605262.2097	323089.2325
445650.8585	602028.958	326685.5689
445631.4471	606243.7152	326700.473
443763.8666	603237.5047	323428.4915
441639.6536	605163.4491	320762.7393
441204.502	604016.8051	317020.771
444606.3022	607462.3059	322579.0145
449759.8731	614746.9827	324008.5664
453832.0103	618314.4088	327740.3296
459884.4119	626710.0137	332622.0932
466085.9032	633771.4953	339601.9874
470971.4862	639012.4643	345671.2911
472798.089	640836.5063	343463.0801
468214.0845	636952.2071	341135.1859
461065.1883	629792.6623	335892.6668
455580.1353	619122.2117	329132.2758
450955.2099	616853.9459	326822.3546
450920.4743	617345.223	326370.4814
452213.907	619419.0042	324239.3076
454514.7171	623292.3072	327148.2562
457708.4025	627529.5511	328478.4226
461680.2772	635820.0153	329430.9458
467267.4397	642924.8306	334001.1915
475961.3123	655364.5888	336987.5141
483787.9115	666124.1816	341713.8122
487236.9505	675581.5687	342977.9788
490839.4339	680127.3761	346059.2482
NA	NA	NA
NA	NA	NA
NA	NA	NA
NA	NA	NA
NA	NA	NA
NA	NA	NA
NA	NA	NA
NA	NA	NA
NA	NA	NA
NA	NA	NA
NA	NA	NA
408773.0496	558056.4793	296342.2203
409002.5601	549118.8544	296219.0023
407266.971	546438.0152	300420.9894
404940.9221	537696.5627	296715.513
403663.612	539207.3569	297331.0215
403818.7366	533052.7741	302356.9605
402116.6034	529706.6051	301236.3551

398140.9709	527713.2494	299249.7918
397189.3224	515120.9569	301220.1843
398141.1854	521622.8766	305449.6454
403503.2905	524270.4095	308069.8324
408674.3544	527286.1122	318543.1311
416560.0764	535954.7923	321859.7929
424519.8247	547210.9598	333190.7677
431402.8098	553023.1771	340736.3793
433867.0627	554893.2107	344718.6826
433738.6584	552081.8972	343537.113
430408.9983	544896.1617	343727.5733
429869.7203	546203.6446	339375.2495
429589.033	544146.7048	339837.4793
430046.7223	547615.9355	340521.59
432177.5191	545993.3727	336714.4708
435307.0841	556663.626	336489.4243
438388.5825	558496.4054	339718.7275
439495.7355	563953.1244	338384.1184
442231.2702	566095.1912	339450.9028
446987.086	572139.3094	337010.1971
449566.8753	579485.3898	337380.7667
444985.4404	571786.131	334786.2725
442645.2895	572399.3706	325852.3598
NA	NA	NA
NA	NA	NA
NA	NA	NA
NA	NA	NA
NA	NA	NA
NA	NA	NA
NA	NA	NA
NA	NA	NA
NA	NA	NA
NA	NA	NA
NA	NA	NA
378116.7145	501183.4883	274952.7582
378106.859	506988.4321	276414.0571
378698.1597	505043.5072	274053.0649
376906.5371	504389.4371	272989.2425
378509.8611	506611.1324	274524.7497
378658.1217	507317.9618	275401.0793
378248.2715	507459.1048	274320.2833
380086.6271	512299.9229	275735.8475
382438.9333	514646.9761	277943.1059
386616.5024	519134.344	281095.084
394544.9172	537304.6714	287684.6609
401361.4393	538189.1405	292883.3607
410612.0604	549500.5411	302852.0809
420946.6625	560749.1734	311106.239
429477.0055	572696.9774	318135.4196
435107.5609	582313.8438	319154.7352
438112.3391	588373.7251	324656.1351
437627.092	586074.3908	322541.1977
439171.9008	594207.5935	323341.5294
441400.6403	592449.8675	325432.7249
443935.3786	601023.1408	325520.8144
447059.1482	604382.9396	327661.0963
450211.2091	608697.3726	328375.1948
454722.271	619300.4428	330281.5865
459140.8558	623607.1098	330755.5808
464518.5807	637342.5917	337718.6879
473390.6225	646351.2458	337729.1181
482627.0214	658526.8144	346242.1621

486652.0497	668368.7379	351143.2758
489449.311	672861.716	350725.0287
NA	NA	NA
NA	NA	NA
NA	NA	NA
NA	NA	NA
NA	NA	NA
NA	NA	NA
NA	NA	NA
NA	NA	NA
NA	NA	NA
NA	NA	NA
NA	NA	NA
786889.7642	1051074.997	593578.264
787109.4191	1045379.973	599533.2072
785965.1308	1041992.751	595967.3291
781847.4591	1040566.678	590321.7769
782173.4731	1036555.553	595978.6613
782476.8583	1036451.257	597445.0689
780364.875	1039212.018	592966.0724
778227.598	1033368.293	595725.8851
779628.2557	1031109.041	592146.4023
784757.6878	1036714.352	599352.3599
798048.2077	1057826.238	608338.9794
810035.7937	1064458.142	618809.6007
827172.1367	1090443.467	632167.0173
845466.4872	1105750.675	653061.5651
860879.8153	1119756.087	662622.3046
868974.6236	1123883.203	672522.6028
871850.9975	1133720.623	672060.0611
868036.0903	1126199.554	671971.0344
869041.6211	1133883.505	671196.711
870989.6733	1132177.435	673956.651
873982.1009	1142624.836	677106.6596
879236.6673	1152070.684	676566.3429
885518.2932	1167093.358	676092.526
893110.8535	1178362.941	683910.6987
898636.5912	1186374.226	685050.0877
906749.8509	1197083.205	693959.9438
920377.7085	1215581.08	693141.0813
932193.8967	1237104.385	699672.2229
931637.4902	1234942.156	694171.8371
932094.6005	1237307.784	696949.9837
NA	NA	NA
NA	NA	NA
NA	NA	NA
NA	NA	NA
NA	NA	NA
NA	NA	NA
NA	NA	NA
NA	NA	NA
NA	NA	NA
NA	NA	NA
NA	NA	NA
263042.9866	372811.0973	181246.5375
265959.6304	383866.0979	184172.1013
267822.0542	388024.7669	184652.1056
268648.4783	386261.1607	190147.9539
269230.1659	380628.3276	190805.4564
269595.7172	375844.6457	192528.4664
270057.4095	370175.1156	195186.3267
272235.3518	373113.0264	198396.1343

273379.9928	374450.998	199552.1154
274158.025	370010.9521	201222.2312
275569.4807	372848.039	202386.2797
277030.4196	374127.4861	202445.333
278517.812	373216.7524	207119.7946
279054.9417	369981.1854	208303.3675
280159.1861	371516.8231	213165.4408
282055.7092	376958.7882	212040.103
284724.6709	378136.6762	216068.822
287174.2596	379868.5134	219018.5494
292159.7228	387906.8306	221868.3058
295072.5891	389254.0281	224250.8014
297499.0879	392304.8635	227789.9015
300293.3694	398413.6553	228843.682
302499.8747	401443.2257	230361.2931
305873.4872	405529.7696	231650.0629
308485.3797	408603.657	232524.7042
312907.1921	415263.484	237497.216
321107.3367	424789.8705	242547.9573
328697.6405	442072.3402	248060.367
328648.476	435011.0121	247400.3097
328417.9103	440679.5065	245680.0032
NA	NA	NA
NA	NA	NA
NA	NA	NA
NA	NA	NA
NA	NA	NA
NA	NA	NA
NA	NA	NA
NA	NA	NA
NA	NA	NA
NA	NA	NA
NA	NA	NA
242418.2316	324637.1609	172887.2222
244092.9439	328503.3123	174275.0218
246434.4673	331978.9962	177081.9673
247895.6609	332833.7285	177089.4329
250408.0952	338758.3463	180547.0701
252627.0445	339596.2856	182290.6805
255412.9932	344026.6154	184373.1365
259910.6675	347779.2302	188056.7193
262642.9206	349975.4625	190581.5159
265536.4956	358363.7844	193112.693
269667.8334	361801.7508	195590.4897
273189.5627	365818.5742	199427.8763
276566.1906	371331.5457	200839.0917
279714.6044	376030.2341	205041.8484
283146.8717	379566.8831	206812.3658
287747.6521	387392.1945	211783.5521
293457.1611	394591.5904	215838.4065
300932.1913	405349.519	221479.6303
310859.1336	419741.9347	228535.2804
319584.2285	433978.8109	234490.3391
327524.6493	446585.1872	240783.7315
333127.1197	449823.3663	243991.2951
336413.0607	458554.52	246955.9642
342023.5501	464854.2408	251037.7547
346856.916	473071.3027	256091.1483
354132.1061	481888.8507	259720.5149
364492.3493	498255.5003	266073.9671
375128.2539	511224.4074	274369.3046
378839.3215	520178.0195	274851.8083

380874.0042	521249.3746	274897.1379
NA	NA	NA
NA	NA	NA
NA	NA	NA
NA	NA	NA
NA	NA	NA
NA	NA	NA
NA	NA	NA
NA	NA	NA
NA	NA	NA
NA	NA	NA
NA	NA	NA
505461.2182	664081.6266	376876.9621
510052.5743	671126.3919	380436.7569
514256.5215	674824.1434	383461.5652
516544.1392	683061.4412	389104.188
519638.2612	680780.5431	391329.3868
522222.7616	684012.8492	395622.0518
525470.4027	691322.7131	398257.5558
532146.0193	698238.5681	404202.1101
536022.9134	704209.3167	407531.4989
539694.5207	710071.5384	410623.8222
545237.314	721985.2239	415720.831
550219.9824	720862.4363	421675.153
555084.0027	729743.118	424310.9856
558769.5461	732849.6066	429148.4143
563306.0578	739947.5864	434733.4402
569803.3613	751336.0028	439561.5673
578181.832	760044.7215	446541.7219
588106.4509	782187.6454	451892.2813
603018.8564	801701.2975	462868.555
614656.8176	816911.8627	470071.3247
625023.7372	832245.0935	478202.962
633420.4891	848188.1987	482034.4738
638912.9353	857331.0532	486018.095
647897.0373	868722.5454	494942.8388
655342.2957	881785.8285	497023.2846
667039.2982	897041.7854	507454.3836
685599.686	922820.2234	517737.7917
703825.8944	949489.8384	532274.5247
707487.7975	957215.2028	534059.8158
709291.9145	956708.7909	530069.9533
NA	NA	NA
NA	NA	NA
NA	NA	NA
NA	NA	NA
NA	NA	NA
NA	NA	NA
NA	NA	NA
NA	NA	NA
NA	NA	NA
NA	NA	NA
NA	NA	NA
165538.1696	231665.0561	107243.0515
168498.5135	235210.766	108549.3656
171372.0816	237589.5679	111423.7203
174127.2931	241689.5899	113545.342
177050.6304	244806.0657	114553.6911
179658.2329	249114.3422	117388.0114
182988.8004	253402.1857	118624.0035
185770.6652	258532.4197	121018.1848
189134.7786	262310.109	124158.3714

192034.2102	263744.4456	124997.1035
193769.2489	264794.6259	127196.6973
194073.1354	267225.6158	129166.442
195042.5109	268143.3705	129900.2358
196791.8119	269160.2974	132586.7424
198321.5838	269190.6935	135326.0244
199894.0423	269794.8194	136931.1815
204015.148	274532.9584	138923.2723
208918.8829	280441.0151	142919.6965
212230.6171	280754.2581	146750.7606
216065.6726	286427.4594	150085.4871
217659.4344	290075.7727	152738.9193
220849.3309	293767.8483	154339.1174
224346.3531	298680.3172	158987.7205
228873.824	302623.7457	161338.5083
232752.0763	305377.9764	164552.0241
237407.1017	314187.1668	168452.978
244203.7163	325717.7779	173554.1047
250114.782	335075.665	178174.7786
253505.1136	337523.2947	181882.1442
256467.2828	341839.6906	182989.0088
NA	NA	NA
NA	NA	NA
NA	NA	NA
NA	NA	NA
NA	NA	NA
NA	NA	NA
NA	NA	NA
NA	NA	NA
NA	NA	NA
NA	NA	NA
NA	NA	NA
105751.5854	143952.0532	74892.36055
107543.2682	145890.3993	75995.90815
109439.3063	148510.6269	78030.32798
111062.8445	151050.4448	79184.22155
112937.0913	152618.0363	80583.68026
115106.2319	155364.3	82869.3088
117735.0507	158951.8314	84517.25788
120135.1639	162317.4601	86973.38787
122803.1127	166156.5578	88414.72603
125115.6732	168679.279	90715.02384
126871.5339	171748.3685	91700.97285
128689.1317	174281.1394	92967.41657
130655.2154	175676.4878	94077.73804
132797.2061	178942.4941	95496.04763
135014.0325	181842.2462	96796.48451
137615.0951	184030.8561	99690.20148
140679.5725	188075.6909	102139.6073
144785.7678	194769.3268	104988.1762
149759.7817	200814.8436	108783.2867
154492.3317	208321.2548	112376.7691
158699.3754	214704.1153	115539.9683
162375.5944	219320.7308	118613.3113
166049.1525	223896.0057	120575.6128
169814.8728	230034.2874	124022.1685
174170.7561	237355.3189	126692.7487
178422.5219	244017.3465	129646.1532
184552.8214	253468.4756	134218.6081
191388.7816	262105.5403	138692.2979
196562.2647	269540.7513	142159.3053
201276.4408	275635.3175	145261.6955

NA	NA	NA
NA	NA	NA
NA	NA	NA
NA	NA	NA
NA	NA	NA
NA	NA	NA
NA	NA	NA
NA	NA	NA
NA	NA	NA
NA	NA	NA
NA	NA	NA
271289.7551	351599.5993	196972.2039
276041.7817	357713.3146	200554.7974
280811.3879	362288.523	202629.8012
285190.1376	367704.2809	206595.6477
289987.7217	372085.3399	209987.6029
294764.4648	380878.5871	213266.4095
300723.8511	387902.0623	217250.3956
305905.8291	395585.558	221519.4191
311937.8914	403469.0448	225198.0115
317149.8833	411387.3305	229090.6593
320640.7828	415442.9599	231462.7267
322762.2671	415963.6766	234598.4409
325697.7264	421525.796	237193.3986
329589.018	424403.0778	240502.7703
333335.6163	432838.7044	243084.5256
337509.1374	435314.4533	248245.545
344694.7206	445275.2805	254928.9371
353704.6507	458739.7101	261174.1883
361990.3988	467359.5461	269142.1952
370558.0043	477999.5016	274217.1302
376358.8098	489749.8492	279001.8703
383224.9253	498114.5521	286200.9615
390395.5056	509011.5421	290284.3341
398688.6968	519618.2014	298356.6591
406922.8324	530092.1043	303487.3249
415829.6236	542923.1742	309846.821
428756.5377	560789.8897	319640.1266
441503.5636	583659.5181	329530.3561
450067.3782	592468.7412	334694.3526
457743.7236	606262.7963	340744.7134
NA	NA	NA
NA	NA	NA
NA	NA	NA
NA	NA	NA
NA	NA	NA
NA	NA	NA
NA	NA	NA
NA	NA	NA
NA	NA	NA
NA	NA	NA
NA	NA	NA
1622894.544	2902319.269	839407.2213
1654802.652	2961667.944	855237.3743
1687413.673	3022165.763	871507.0304
1720443.959	3091096.991	888868.6188
1754253.634	3154712.528	907154.9329
1789273.811	3219405.448	926025.481
1826445.471	3285324.014	945764.5012
1865382.976	3349795.755	964350.4914
1904924.279	3416241.09	986258.2466
1944648.864	3484464.722	1008372.262

1984027.172	3551200.851	1027230.512
2022822.254	3616213.369	1048327.032
2061198.125	3679186.517	1066558.024
2099717.095	3748301.381	1086376.743
2139030.041	3816112.239	1106631.629
2179915.925	3888661.619	1128690.734
2220141.076	3965269.355	1149790.255
2258652.458	4036000.968	1168956.347
2296751.528	4111993.273	1189294.917
2336568.47	4178168.131	1208450.593
2380948.39	4253778.981	1232470.229
2430339.404	4339745.366	1257445.329
2482161.209	4435778.58	1282284.415
2534953.811	4527716.247	1311042.532
2587755.077	4623446.56	1335796.237
2639664.337	4719758.641	1362022.072
2704074.408	4847170.148	1394803.437
2767879.809	4987476.058	1427041.792
2819123.779	5082765.559	1450268.19
2872510.151	5183408.245	1479428.025
NA	NA	NA
NA	NA	NA
NA	NA	NA
NA	NA	NA
NA	NA	NA
NA	NA	NA
NA	NA	NA
NA	NA	NA
NA	NA	NA
NA	NA	NA
NA	NA	NA
1779408.483	3181334.574	922963.5105
1814845.025	3245852.835	942161.2217
1850887.234	3309765.426	959521.7169
1887359.877	3375832.828	979193.1086
1924776.435	3440568.021	998381.4891
1963242.121	3507282.892	1018702.925
2004395.367	3585285.031	1040160.871
2048459.679	3662044.173	1062869.675
2093754.944	3745080.244	1086561.539
2139402.381	3824607.514	1109028.743
2184276.261	3902457.048	1131203.046
2225559.439	3975200.606	1154657.939
2263024.401	4040424.788	1174331.721
2298867.916	4102677.091	1193018.302
2336171.09	4164755.076	1211087.148
2377450.11	4241761.214	1233650.86
2420786.479	4314034.355	1258049.083
2463112.968	4383102.562	1280877.005
2506075.458	4466903.584	1302998.501
2551465.762	4546263.962	1324456.104
2602297.883	4638461.862	1349856.673
2657772.04	4737343.035	1379391.171
2715241.531	4837856.238	1407192.68
2773722.42	4947274.795	1436481.774
2832027.695	5044508.499	1465120.468
2889265.893	5151765.115	1494303.092
2965198.283	5283872.071	1532539.024
3040806.976	5421813.681	1570553.336
3097383.178	5527695.62	1598869.313
3155917.726	5632673.005	1623853.192
NA	NA	NA

NA	NA	NA
NA	NA	NA
NA	NA	NA
NA	NA	NA
NA	NA	NA
NA	NA	NA
NA	NA	NA
NA	NA	NA
NA	NA	NA
NA	NA	NA
3402303.026	6093057.849	1762370.732
3469647.678	6215479.844	1797398.596
3538300.907	6342172.009	1831028.747
3607803.836	6466584.859	1868061.727
3679030.07	6594838.999	1905145.802
3752515.932	6727735.746	1944794.185
3830840.839	6871804.751	1984529.065
3913842.655	7011490.573	2027288.826
3998679.223	7164962.027	2073118.105
4084051.246	7308883.949	2117521.289
4168303.433	7455216.231	2158590.999
4248381.693	7598951.032	2200474.846
4324222.526	7740242.704	2240509.224
4398585.011	7871430.645	2279081.028
4475201.131	8006465.141	2319225.542
4557366.036	8152653.321	2363488.581
4640927.555	8295498.243	2408303.128
4721765.425	8426635.334	2449833.352
4802826.986	8563839.675	2495425.755
4888034.232	8705859.032	2537039.579
4983246.274	8857874.851	2582874.1
5088111.443	9049033.933	2639025.689
5197402.74	9248893.623	2689423.93
5308676.231	9450519.286	2747457.509
5419782.773	9645934.086	2800834.836
5528930.229	9842961.849	2856186.309
5669272.691	10120325.93	2924680.69
5808686.785	10410832.94	2993702.37
5916506.957	10610356.3	3048526.933
6028427.877	10815633.89	3103987.903
NA	NA	NA
NA	NA	NA
NA	NA	NA
NA	NA	NA
NA	NA	NA
NA	NA	NA
NA	NA	NA
NA	NA	NA
NA	NA	NA
NA	NA	NA
NA	NA	NA
283403.8876	513334.5456	143507.336
286615.3157	519558.1751	144619.1628
289966.2443	524730.6658	146042.9188
293307.0113	531985.2561	147666.5892
296710.6653	538988.5891	148788.6032
300366.687	545385.7937	150876.8025
304163.0629	552233.2351	152565.9641
308269.4923	558518.6131	155140.4279
312456.2901	565655.0574	157900.8894
316591.5472	574347.8211	160742.2708
320679.284	581359.1759	162762.4088

323969.564	586448.8344	163820.5063
326195.2234	588493.1422	165246.6751
327789.8724	591654.3523	166815.7194
329239.8851	593809.7245	167090.191
331124.8632	597462.0487	167604.9758
333022.388	600427.3321	168760.5902
334816.7389	605424.6702	169834.838
337118.2515	609797.8989	170911.0099
340162.3717	616071.0887	172037.7723
344244.6316	624470.8276	174217.3159
350083.7714	636126.8765	177491.3303
357141.713	647484.2791	181100.5282
364516.4779	661221.6746	184967.1531
371428.9175	673895.715	188571.3862
376940.6467	683504.6963	191493.2153
381505.8623	692590.3149	193591.3708
385864.2473	700165.1739	195849.189
391362.2792	708030.2379	198555.8424
398538.5266	716927.4802	201468.2597
NA	NA	NA
NA	NA	NA
NA	NA	NA
NA	NA	NA
NA	NA	NA
NA	NA	NA
NA	NA	NA
NA	NA	NA
NA	NA	NA
NA	NA	NA
NA	NA	NA
330478.3422	596156.0332	170237.5219
333831.3385	603492.7744	171341.8731
337195.424	609222.884	172630.3087
340506.9045	612911.6126	173834.7468
343846.6322	621079.5079	174972.2218
347273.3129	626162.9581	176623.6824
350657.7403	631947.8319	178681.8661
353991.0572	636095.8811	181043.1165
357198.6693	643932.2954	182712.3804
360413.9661	649538.0601	184989.4928
363771.9112	656776.7509	187039.2603
365773.9686	660684.1787	187612.9919
365634.1975	661300.1713	186666.4952
364356.4876	660030.7791	185366.6913
363195.5878	658176.3525	184108.9191
363293.0281	657854.1834	184331.4422
364196.9736	659558.0555	184516.1327
365156.695	660216.1933	184957.9067
366742.9216	663679.9173	186086.4623
369320.1272	666315.7123	188127.8033
373566.0836	673502.5649	190343.8311
379869.1415	686100.0935	193427.0686
387685.561	700699.7273	196587.4659
395895.5194	713664.4109	200254.7475
403421.7727	725543.6456	203993.4739
409217.4827	733252.671	206651.7158
413734.0281	742457.3067	208611.712
417992.7261	749990.7316	210826.2171
423684.7348	761857.202	214105.2187
431175.2794	776366.7142	218106.4896
NA	NA	NA
NA	NA	NA

NA	NA	NA
NA	NA	NA
NA	NA	NA
NA	NA	NA
NA	NA	NA
NA	NA	NA
NA	NA	NA
NA	NA	NA
NA	NA	NA
613882.2298	1109140.414	313802.6244
620446.6542	1121443.859	316083.4356
627161.6683	1133949.131	318675.7863
633813.9158	1143001.656	321810.9406
640557.2976	1154663.2	324276.2874
647639.9998	1167426.368	327222.7444
654820.8032	1182110.096	331913.1118
662260.5496	1195918.608	336409.5871
669654.9594	1210810.084	341043.5656
677005.5133	1225038.189	345781.0061
684451.1951	1242130.931	349712.3564
689743.5326	1251012.136	351542.7961
691829.4209	1254583.51	352220.9684
692146.36	1253505.525	351536.4742
692435.4729	1252489.382	351468.622
694417.8913	1256416.322	352244.7985
697219.3616	1259451.286	353831.7718
699973.434	1264893.983	355082.687
703861.173	1272559.207	357350.9889
709482.4989	1282581.434	360273.0312
717810.7153	1297477.755	364602.3976
729952.9129	1318448.873	370610.559
744827.274	1344754.989	377710.3949
760411.9972	1373279.243	385216.7777
774850.6902	1401465.425	392661.0491
786158.1294	1419831.983	398845.0227
795239.8905	1439641.778	403014.5466
803856.9734	1457791.094	406695.2709
815047.014	1473019.97	413086.4922
829713.806	1492911.455	420692.7198
NA	NA	NA
NA	NA	NA
NA	NA	NA
NA	NA	NA
NA	NA	NA
NA	NA	NA
NA	NA	NA
NA	NA	NA
NA	NA	NA
NA	NA	NA
NA	NA	NA
358815.2461	647522.8386	184265.796
363795.6775	657464.9095	187378.2566
368840.5867	666553.6512	189389.9218
373803.1285	674458.3866	191959.7195
378845.1273	684342.6932	194635.3576
384091.7273	692647.1796	197085.9822
389703.2225	702915.6719	199691.4395
395468.2006	712124.627	202040.3786
401268.2399	722556.9098	204614.9116
407102.4901	732264.7565	208402.3296
412948.0402	740608.8277	210964.0527
418880.0551	754220.7353	213780.3443

424755.5431	764870.969	217098.7878
430681.4044	775687.3504	220058.4362
436691.3319	787764.8916	223232.3695
442979.159	800563.252	226599.8672
448906.5888	808258.0107	229340.9548
453956.0095	815622.4193	232357.9156
458566.5074	825781.963	234774.3943
463282.7614	831580.7916	237495.7141
468912.5411	841363.1037	239835.1831
475631.8094	855355.3869	243514.5413
482737.61	868658.013	246886.7999
489694.1704	882998.5194	250560.4415
496320.6088	896021.9594	253725.4042
502779.1868	907662.2128	257654.7016
516145.5529	934420.6016	263086.8039
529176.7014	959857.0621	269600.5867
534354.1045	968773.5408	272031.9375
539377.5232	978388.6653	274383.7016
NA	NA	NA
NA	NA	NA
NA	NA	NA
NA	NA	NA
NA	NA	NA
NA	NA	NA
NA	NA	NA
NA	NA	NA
NA	NA	NA
NA	NA	NA
NA	NA	NA
444154.889	797825.7613	229451.7253
450532.8282	808731.1908	233012.4736
456980.6508	819884.6502	236414.5842
463310.8888	831756.259	239599.519
469558.1286	842051.0712	242217.9664
475869.9078	854809.0307	245284.1824
482940.6706	869742.3024	247880.7619
491019.6206	883035.8958	252296.6634
499507.7213	898943.8061	256479.024
507947.7337	911764.9969	260199.9646
515869.6968	924495.6794	263690.2589
522322.5805	934955.8601	266542.3853
527406.3498	944507.7862	270433.481
531646.7621	950770.6308	272879.1815
536088.8949	958748.9031	275510.9124
541333.3178	972393.4107	278676.9062
546944.1225	983069.998	282243.4199
551938.8785	994735.3759	285192.9969
557000.3148	1000960.854	288385.5775
562633.9032	1012645.561	291489.9371
569554.7388	1025784.713	294869.4434
577576.4183	1039057.241	299149.2955
585857.4643	1055938.6	303647.3152
594053.887	1074268.39	307570.1932
601948.749	1087930.65	310649.2839
609397.6891	1102327.884	313853.6353
626332.399	1128344.743	321418.7802
642815.8432	1156385.669	329164.1376
648690.9633	1166730.176	332321.6075
654198.1181	1177112.023	334840.9277
NA	NA	NA
NA	NA	NA
NA	NA	NA

NA	NA	NA
NA	NA	NA
NA	NA	NA
NA	NA	NA
NA	NA	NA
NA	NA	NA
NA	NA	NA
NA	NA	NA
802970.1351	1445577.618	414860.2413
814328.5057	1466461.246	419854.0913
825821.2374	1486554.457	425420.3659
837114.0173	1505889.679	430944.93
848403.2558	1526393.764	436794.3835
859961.6351	1550625.64	442370.1646
872643.8931	1573152.396	447572.2014
886487.8212	1595267.876	454431.8453
900775.9612	1623079.448	461332.9786
915050.2238	1648340.004	469016.5168
928817.737	1669422.256	474457.899
941202.6355	1691522.999	480309.0887
952161.8929	1709520.361	487634.788
962328.1665	1725396.78	492979.6179
972780.2268	1743525.574	498556.0439
984312.4768	1766150.468	504739.1151
995850.7113	1782128.694	511544.1117
1005894.888	1802911.001	517550.9124
1015566.822	1817671.919	523159.9718
1025916.665	1838196.919	529162.5652
1038467.28	1866863.227	535857.3663
1053208.228	1894347.468	542828.2162
1068595.074	1924454.72	551187.1618
1083748.057	1953240.451	557239.8992
1098269.358	1979371.388	564945.9431
1112176.876	2002166.317	570976.559
1142477.952	2057036.562	585453.3554
1171992.545	2118761.374	600349.4975
1183045.068	2136344.739	605979.1772
1193575.641	2157746.521	610302.6814
NA	NA	NA
NA	NA	NA
NA	NA	NA
NA	NA	NA
NA	NA	NA
NA	NA	NA
NA	NA	NA
NA	NA	NA
NA	NA	NA
NA	NA	NA
NA	NA	NA
437539.3132	781519.1227	227007.3802
448022.2036	802311.3835	232672.1422
458745.6725	819821.7728	238873.0841
469907.2572	842685.9015	244625.1563
481564.7374	863351.051	250418.4768
493706.8835	883240.3124	255422.6931
506554.9706	907854.4754	263162.0152
519861.3453	931266.5518	269708.425
533440.8147	953818.4491	277304.6122
547074.7082	977009.2175	284936.4096
560508.6443	997211.1987	291576.5033
574065.9555	1020574.618	298223.1835
587954.4594	1046956.209	305439.0977

602217.2476	1069964.833	312600.2323
616519.5238	1097501.383	319738.3911
630676.2674	1121751.126	326709.3612
644097.3802	1148671.901	334146.6015
656399.8773	1172715.12	340919.5038
667941.8586	1194828.274	346728.9569
679646.3423	1214930.757	353048.2746
693081.6404	1243475.483	359567.4445
707954.8732	1268780.201	368069.7436
723221.2018	1298087.61	375361.3193
738752.209	1323830.294	383538.1526
754674.8978	1351498.503	391306.6052
770494.2192	1380955.515	398961.5111
791834.3076	1415613.379	408914.4545
812938.1488	1459298.157	418704.2359
827821.2175	1485681.956	426506.2353
842416.6166	1513859.611	434144.3314
NA	NA	NA
NA	NA	NA
NA	NA	NA
NA	NA	NA
NA	NA	NA
NA	NA	NA
NA	NA	NA
NA	NA	NA
NA	NA	NA
NA	NA	NA
NA	NA	NA
462708.6721	827034.7929	241326.2952
474827.9886	849439.5789	246919.3105
487103.3195	871310.9889	253057.8177
499758.3904	896073.7058	259526.1694
512834.6549	919201.6893	265877.8685
526302.283	942917.9162	273258.7942
540229.6854	967574.825	280115.0129
554505.0509	990793.2296	287518.3395
569134.9917	1017124.748	295005.1167
583981.8087	1040863.642	302061.9447
599072.6909	1067607.856	310082.5202
614405.8821	1097055.84	317624.2974
629939.5156	1127776.915	326182.0284
645769.1392	1155424.681	333962.8312
661694.3138	1183005.707	342528.3622
677793.1287	1210971.016	350649.7261
693402.0269	1237751.041	358160.7992
708071.5793	1263486.596	366174.026
722188.1096	1286529.146	373807.3008
736512.671	1310390.207	382501.155
752190.8048	1337930.744	390972.2629
769116.3909	1368509.331	399389.9888
786291.3638	1397818.578	407149.5041
803819.6713	1432453.011	414975.9472
821584.8134	1461690.013	422996.1428
839340.4555	1493929.651	432124.5507
863798.0319	1544339.888	444613.0151
888050.806	1591178.513	457274.3785
904781.0815	1622838.695	468345.9047
921177.4203	1651665.177	476253.5913
NA	NA	NA
NA	NA	NA
NA	NA	NA
NA	NA	NA

NA	NA	NA
NA	NA	NA
NA	NA	NA
NA	NA	NA
NA	NA	NA
NA	NA	NA
NA	NA	NA
900247.9853	1613341.72	468333.6754
922850.1922	1655923.406	480151.5349
945848.992	1693908.87	491901.9384
969665.6476	1739675.666	504701.0746
994399.3923	1782552.74	518240.9796
1020009.167	1825702.594	530214.499
1046784.656	1876606.73	544968.8919
1074366.396	1922931.267	558851.6686
1102575.806	1970943.197	572288.0515
1131056.517	2017872.859	587494.7719
1159581.335	2064819.055	602503.1443
1188471.838	2117292.045	616785.542
1217893.975	2176105.544	632084.4289
1247986.387	2230394.893	646563.0636
1278213.838	2287979.799	662594.5061
1308469.396	2343561.535	677359.0873
1337499.407	2394377.374	692307.4007
1364471.457	2441741.931	707093.5298
1390129.968	2486224.418	719267.6284
1416159.013	2531286.197	732446.3143
1445272.445	2582999.338	747454.3617
1477071.264	2638780.807	763677.6421
1509512.566	2695906.188	779008.6234
1542571.88	2755883.236	796482.7815
1576259.711	2813188.516	812624.4856
1609834.675	2873610.156	829021.8875
1655632.339	2954499.645	851062.6388
1700988.955	3050476.67	872429.0831
1732602.299	3108543.568	890286.8339
1763594.037	3164463.591	907808.8766
NA	NA	NA
NA	NA	NA
NA	NA	NA
NA	NA	NA
NA	NA	NA
NA	NA	NA
NA	NA	NA
NA	NA	NA
NA	NA	NA
NA	NA	NA
NA	NA	NA
383247.4852	685270.4527	198264.7976
392371.7043	701244.29	203606.2006
401530.8809	717216.4711	208089.62
410776.4309	735611.456	212962.521
420042.8022	753207.7056	218248.3957
429398.026	771457.7789	223077.8794
439418.7473	788188.2343	228011.5146
450241.1756	804943.3802	234766.3987
461395.8729	823867.2635	241053.8374
472602.464	842830.6977	246880.6341
483495.1034	860448.5756	252316.4194
493823.2952	879061.8683	258697.3487
504073.3731	893125.0698	264155.6518
514499.1919	912816.7951	270395.6554

525546.8703	930513.1602	275976.0635
537497.2738	954489.0206	282827.4915
549747.6385	975714.8644	289117.3817
562049.0565	996620.1177	295286.1197
574361.2793	1019767.304	301337.4661
587103.7751	1042727.022	307124.9863
600535.9444	1067437.401	314573.318
614360.482	1092633.098	321376.3655
628312.6064	1119140.994	328737.2015
642537.4241	1143685.714	336210.9445
657006.762	1169768.954	342874.2037
671934.067	1197815.166	351512.3491
687901.2484	1224375.855	358333.5784
703511.1013	1261834.411	364698.035
718728.7143	1289324.695	374233.2478
734465.3572	1319354.539	382769.0018
NA	NA	NA
NA	NA	NA
NA	NA	NA
NA	NA	NA
NA	NA	NA
NA	NA	NA
NA	NA	NA
NA	NA	NA
NA	NA	NA
NA	NA	NA
NA	NA	NA
377452.8223	669289.7439	197407.9502
386915.263	687088.2826	202541.2137
396557.7969	704964.7923	207684.0862
406399.6696	721824.1022	213178.766
416521.5193	739989.2164	218178.106
426826.0707	756769.9124	223548.8638
438367.3528	776972.4973	229873.087
451418.814	798517.0317	236083.405
465080.7875	822775.3382	243867.7991
478805.6492	842779.678	250909.9989
491594.114	865452.3883	256783.5156
502995.4226	888724.42	263632.7973
513479.9953	906278.0785	269238.4869
523900.3354	925914.3866	274839.0683
535166.3326	943771.2431	280754.5473
547913.7071	968442.6945	287643.5833
561809.716	993076.9598	295099.8507
575793.317	1019984.055	302292.3315
589984.583	1047299.85	309760.1526
604540.8435	1072684.798	317764.9088
619963.4174	1096931.648	325649.1055
635281.8754	1125882.728	334174.5977
650245.2314	1154655.804	341590.3321
665244.2423	1184196.98	349008.5315
680615.9328	1210954.651	356999.0161
696799.908	1239769.343	364488.0574
716354.4739	1269912.881	375338.5379
735946.8925	1302288.067	384339.2984
752783.1617	1328054.994	392785.4544
770123.1197	1359762.274	401702.1625
NA	NA	NA
NA	NA	NA
NA	NA	NA
NA	NA	NA
NA	NA	NA

NA	NA	NA
NA	NA	NA
NA	NA	NA
NA	NA	NA
NA	NA	NA
NA	NA	NA
760700.3074	1354386.251	398399.5682
779286.9673	1388182.159	408590.2675
798088.6778	1422056.371	417795.1607
817176.1006	1456484.389	428679.8959
836564.3215	1491203.249	438427.155
856224.0967	1526856.891	449132.2801
877786.1001	1564912.765	461003.6195
901659.9897	1603235.792	473043.3903
926476.6604	1646515.275	486368.0108
951408.1132	1688168.319	498184.4659
975089.2175	1726549.927	510719.947
996818.7177	1768432.173	523625.2175
1017553.368	1807405.339	533418.2308
1038399.527	1845636.265	545344.9076
1060713.203	1881999.351	556737.2984
1085410.981	1923812.915	570742.8878
1111557.354	1971154.831	584749.964
1137842.374	2018067.816	597413.1245
1164345.862	2065527.129	611864.9054
1191644.619	2112526.234	626285.1998
1220499.362	2158671.745	642269.6183
1249642.357	2216009.954	656322.7026
1278557.838	2269844.275	670632.0359
1307781.666	2324553.156	686531.4811
1337622.695	2373332.521	700871.8321
1368733.975	2432312.861	717522.0725
1404255.722	2496309.599	733669.4509
1439457.994	2556873.151	750645.766
1471511.876	2615727.115	767802.3375
1504588.477	2678871.955	784025.1075
NA	NA	NA
NA	NA	NA
NA	NA	NA
NA	NA	NA
NA	NA	NA
NA	NA	NA
NA	NA	NA
NA	NA	NA
NA	NA	NA
NA	NA	NA
NA	NA	NA
158909.9386	281582.4352	82721.99899
162994.6114	288297.785	84806.7699
167304.2081	295323.851	87037.43292
171602.0795	303488.303	89383.80062
176019.7293	311484.1842	91721.73038
180615.7887	319694.6164	94078.3005
185484.628	329693.0198	96588.62339
190394.8777	337504.1268	99044.97023
195186.9914	345841.6738	101772.4683
200072.3469	353633.9163	104456.6779
205160.1404	363823.7991	107042.5803
210814.9191	373139.6455	109939.4486
216916.9374	383630.3668	113059.7364
223192.0857	394115.0041	116728.4992
229661.1226	404438.6496	120102.4706

236234.6508	414032.4179	123390.7234
242931.8258	427142.891	127349.5529
249964.3526	438162.4011	130475.9507
257267.5888	452246.9866	134226.5937
264849.8768	465708.2042	138293.5051
272627.6432	478478.7843	142321.268
280741.4063	492367.9436	146624.2606
289158.054	506479.2694	151069.7767
297841.2518	523903.964	155130.0146
306688.4898	539893.0367	159905.8559
315856.2303	555372.9213	164524.5034
325000.2658	573855.1566	168967.1539
334673.6814	592867.914	174062.9056
345110.8078	611234.119	179194.2131
355933.7011	630338.3117	184699.2284
NA	NA	NA
NA	NA	NA
NA	NA	NA
NA	NA	NA
NA	NA	NA
NA	NA	NA
NA	NA	NA
NA	NA	NA
NA	NA	NA
NA	NA	NA
NA	NA	NA
163507.9022	290816.6262	85809.12246
167603.2084	298209.4983	88214.3357
171888.9059	306414.0107	90257.36372
176197.6928	312702.9475	92445.50525
180803.6172	322220.1465	95141.20467
185731.3285	330889.3476	97488.28875
190931.9379	339937.621	100366.2961
196227.159	349344.3151	102948.6007
201503.684	357768.5589	105783.3216
206891.9019	367817.1691	108543.3464
212572.9504	377905.9247	111397.5209
218631.6632	389167.617	114944.7426
225097.9579	401023.8441	118441.7981
231692.3465	412037.7941	121986.0053
238487.1824	424890.1331	125413.4131
245543.2553	438562.2438	129231.4781
252825.6256	450890.9788	133037.7827
260510.681	464991.9025	137451.6104
268485.4493	478372.3402	141281.3533
276753.8397	494013.3326	145770.3212
285291.7123	508415.0591	150014.8121
294172.0524	524986.2802	154974.077
303380.2338	542003.3186	159764.8474
312902.1431	559471.8979	165047.3861
322623.8444	576612.3819	169600.207
332650.044	592346.2253	174980.2525
343089.1782	612338.5637	180757.5439
354078.2541	630472.1106	185941.5545
365486.2227	651566.7518	191630.1885
377251.3672	671042.7175	198467.7786
NA	NA	NA
NA	NA	NA
NA	NA	NA
NA	NA	NA
NA	NA	NA
NA	NA	NA

NA	NA	NA
NA	NA	NA
NA	NA	NA
NA	NA	NA
NA	NA	NA
322417.8409	571645.0769	169181.7515
330597.8198	585436.5765	173544.2232
339193.114	601415.4075	178083.7868
347799.7723	616065.2447	182127.2706
356823.3465	633633.4772	187068.8693
366347.1173	648859.5473	191994.6967
376416.5659	667123.9258	197046.3306
386622.0366	684864.8896	202525.9823
396690.6753	701979.4497	207908.465
406964.2488	721370.76	212948.0686
417733.0908	738751.0445	218393.9275
429446.5824	759119.107	225242.5886
442014.8952	781440.3129	232528.0781
454884.4322	804017.7314	239164.4999
468148.305	828578.1023	246307.4097
481777.9061	852183.9731	253553.547
495757.4513	876147.2902	261123.6829
510475.0337	902821.7435	269156.1921
525753.0382	930619.3267	276928.7763
541603.7165	959239.0077	285188.9518
557919.3555	986751.4172	293345.7015
574913.4586	1017354.224	302958.2969
592538.2878	1048792.161	312129.878
610743.3949	1082374.871	321708.0065
629312.3342	1114592.076	331499.8728
648506.2743	1148159.072	341024.2177
668089.444	1185086.314	351062.7743
688751.9355	1221636.228	360391.7678
710597.0305	1262244.826	371904.9743
733185.0683	1299600.801	384712.0772
NA	NA	NA
NA	NA	NA
NA	NA	NA
NA	NA	NA
NA	NA	NA
NA	NA	NA
NA	NA	NA
NA	NA	NA
NA	NA	NA
NA	NA	NA
NA	NA	NA
