# Supplementary material for: The global, regional, and national patterns of change in the burden of nonmalignant upper gastrointestinal diseases from 1990 to 2019 and the forecast for the next decade
Source: Int J Surg. 2024 Jul 3;111(1):80–92. doi: 10.1097/JS9.0000000000001902 (PMC11745775; doi:10.1097/JS9.0000000000001902)
Supplement: Supplementary file 11 [file js9-111-0080-s011.pdf]

Table S10. Age-standardized DALYs rates of PUD, GD, and GERD from 1990 to 2019 and projected to 2030, cate

Location	Cause	Sex	Pred_val	Pred_low	Pred_up	Year
Global	Peptic ulcer disease	Male	245.452687	245.2262973	245.6790767	1990
Global	Peptic ulcer disease	Male	241.2771801	241.0559523	241.4984078	1991
Global	Peptic ulcer disease	Male	237.4526326	237.2360447	237.6692205	1992
Global	Peptic ulcer disease	Male	233.382536	233.1704323	233.5946397	1993
Global	Peptic ulcer disease	Male	228.1545085	227.9473767	228.3616403	1994
Global	Peptic ulcer disease	Male	220.3124832	220.1111955	220.5137708	1995
Global	Peptic ulcer disease	Male	212.0560918	211.8608577	212.2513259	1996
Global	Peptic ulcer disease	Male	205.4118789	205.2221059	205.6016518	1997
Global	Peptic ulcer disease	Male	197.5318208	197.3479599	197.7156817	1998
Global	Peptic ulcer disease	Male	189.984056	189.8061138	190.1619981	1999
Global	Peptic ulcer disease	Male	183.2156027	183.0430455	183.38816	2000
Global	Peptic ulcer disease	Male	175.6709243	175.5039423	175.8379062	2001
Global	Peptic ulcer disease	Male	168.8355994	168.6738162	168.9973825	2002
Global	Peptic ulcer disease	Male	161.5047418	161.3483543	161.6611294	2003
Global	Peptic ulcer disease	Male	153.6553732	153.5048047	153.8059417	2004
Global	Peptic ulcer disease	Male	148.7288039	148.5825991	148.8750088	2005
Global	Peptic ulcer disease	Male	141.0807106	140.940234	141.2211871	2006
Global	Peptic ulcer disease	Male	134.8380572	134.7026671	134.9734472	2007
Global	Peptic ulcer disease	Male	130.5230329	130.3916289	130.654437	2008
Global	Peptic ulcer disease	Male	124.0007545	123.8743064	124.1272027	2009
Global	Peptic ulcer disease	Male	118.7729502	118.6507781	118.8951224	2010
Global	Peptic ulcer disease	Male	113.8988043	113.7807003	114.0169082	2011
Global	Peptic ulcer disease	Male	109.4405001	109.3262351	109.5547651	2012
Global	Peptic ulcer disease	Male	106.0369009	105.9257494	106.1480525	2013
Global	Peptic ulcer disease	Male	101.7791222	101.6715236	101.8867208	2014
Global	Peptic ulcer disease	Male	98.97842645	98.87368045	99.08317245	2015
Global	Peptic ulcer disease	Male	96.73212829	96.62989719	96.8343594	2016
Global	Peptic ulcer disease	Male	94.09589171	93.99631869	94.19546473	2017
Global	Peptic ulcer disease	Male	91.4026572	91.30566234	91.49965207	2018
Global	Peptic ulcer disease	Male	88.97158868	88.87689743	89.06627994	2019
Global	Peptic ulcer disease	Male	86.8920689	84.46550581	89.31863199	2020
Global	Peptic ulcer disease	Male	84.69076048	81.37334738	88.00817357	2021
Global	Peptic ulcer disease	Male	82.56125819	78.11604243	87.00647395	2022
Global	Peptic ulcer disease	Male	80.49895036	74.77622139	86.22167933	2023
Global	Peptic ulcer disease	Male	78.49630918	71.3957498	85.59686855	2024
Global	Peptic ulcer disease	Male	76.55129518	68.00294347	85.09964688	2025
Global	Peptic ulcer disease	Male	74.66545945	64.62252741	84.70839149	2026
Global	Peptic ulcer disease	Male	72.8412587	61.27230027	84.41021713	2027
Global	Peptic ulcer disease	Male	71.0753229	57.9600686	84.19057719	2028
Global	Peptic ulcer disease	Male	69.36029076	54.68742226	84.03315927	2029
Global	Peptic ulcer disease	Male	67.69228139	51.45764091	83.92692187	2030
Global	Peptic ulcer disease	Female	137.3330076	137.1783721	137.4876432	1990
Global	Peptic ulcer disease	Female	134.7699115	134.6185901	134.921233	1991
Global	Peptic ulcer disease	Female	134.0102227	133.8610248	134.1594205	1992
Global	Peptic ulcer disease	Female	130.9035232	130.7575298	131.0495165	1993
Global	Peptic ulcer disease	Female	127.9746408	127.8317599	128.1175216	1994
Global	Peptic ulcer disease	Female	124.3377895	124.1983873	124.4771916	1995
Global	Peptic ulcer disease	Female	121.1072142	120.9710713	121.2433571	1996
Global	Peptic ulcer disease	Female	119.9818758	119.8479319	120.1158196	1997
Global	Peptic ulcer disease	Female	116.4818237	116.3513105	116.6123369	1998
Global	Peptic ulcer disease	Female	113.1134319	112.9862552	113.2406085	1999
Global	Peptic ulcer disease	Female	110.2666894	110.142485	110.3908939	2000
Global	Peptic ulcer disease	Female	107.1005509	106.9794501	107.2216516	2001
Global	Peptic ulcer disease	Female	103.9060369	103.7879863	104.0240875	2002
Global	Peptic ulcer disease	Female	100.2949858	100.1802107	100.409761	2003
Global	Peptic ulcer disease	Female	95.82987699	95.71892683	95.94082715	2004

Global	Peptic ulcer disease	Female	93.44333813	93.33499861	93.55167764	2005
Global	Peptic ulcer disease	Female	89.6369005	89.53200957	89.74179143	2006
Global	Peptic ulcer disease	Female	85.64575248	85.54442603	85.74707894	2007
Global	Peptic ulcer disease	Female	82.47565293	82.37738645	82.5739194	2008
Global	Peptic ulcer disease	Female	78.30850381	78.21386023	78.40314738	2009
Global	Peptic ulcer disease	Female	75.82340122	75.73132117	75.91548127	2010
Global	Peptic ulcer disease	Female	73.28841973	73.19891176	73.3779277	2011
Global	Peptic ulcer disease	Female	70.37650011	70.28979347	70.46320675	2012
Global	Peptic ulcer disease	Female	68.79155937	68.70682528	68.87629345	2013
Global	Peptic ulcer disease	Female	66.5602028	66.47780628	66.64259932	2014
Global	Peptic ulcer disease	Female	65.41536865	65.33457421	65.4961631	2015
Global	Peptic ulcer disease	Female	64.41332815	64.33402288	64.49263343	2016
Global	Peptic ulcer disease	Female	63.54368878	63.46578562	63.62159194	2017
Global	Peptic ulcer disease	Female	62.17003377	62.0937809	62.24628663	2018
Global	Peptic ulcer disease	Female	60.87219378	60.79745513	60.94693242	2019
Global	Peptic ulcer disease	Female	59.82711672	57.77802224	61.87621119	2020
Global	Peptic ulcer disease	Female	58.78420947	56.03184553	61.5365734	2021
Global	Peptic ulcer disease	Female	57.76074771	54.10943532	61.4120601	2022
Global	Peptic ulcer disease	Female	56.75334899	52.07047172	61.43622625	2023
Global	Peptic ulcer disease	Female	55.76513299	49.95447617	61.5757898	2024
Global	Peptic ulcer disease	Female	54.80045598	47.78826813	61.81264384	2025
Global	Peptic ulcer disease	Female	53.85580507	45.58685145	62.12475868	2026
Global	Peptic ulcer disease	Female	52.92797248	43.36015681	62.49578814	2027
Global	Peptic ulcer disease	Female	52.01420039	41.11496393	62.91343685	2028
Global	Peptic ulcer disease	Female	51.11755086	38.86026988	63.37483184	2029
Global	Peptic ulcer disease	Female	50.24178795	36.60420306	63.87937284	2030
Global	Peptic ulcer disease	Both	189.0923629	187.997929	190.1867968	1990
Global	Peptic ulcer disease	Both	185.8227135	184.750842	186.8945851	1991
Global	Peptic ulcer disease	Both	183.6371151	182.5838066	184.6904237	1992
Global	Peptic ulcer disease	Both	180.1500147	179.1185282	181.1815011	1993
Global	Peptic ulcer disease	Both	176.1445751	175.1359308	177.1532195	1994
Global	Peptic ulcer disease	Both	170.4757903	169.493182	171.4583987	1995
Global	Peptic ulcer disease	Both	164.804205	163.8483321	165.7600779	1996
Global	Peptic ulcer disease	Both	161.0051798	160.0700303	161.9403292	1997
Global	Peptic ulcer disease	Both	155.3985476	154.4887439	156.3083514	1998
Global	Peptic ulcer disease	Both	150.086129	149.2008909	150.9713672	1999
Global	Peptic ulcer disease	Both	145.3629277	144.5009412	146.2249141	2000
Global	Peptic ulcer disease	Both	140.0870082	139.2496705	140.924346	2001
Global	Peptic ulcer disease	Both	135.1568937	134.3434394	135.9703479	2002
Global	Peptic ulcer disease	Both	129.7514043	128.962733	130.5400757	2003
Global	Peptic ulcer disease	Both	123.6670654	122.9045114	124.4296195	2004
Global	Peptic ulcer disease	Both	120.0697007	119.3261652	120.8132363	2005
Global	Peptic ulcer disease	Both	114.4432982	113.7248923	115.1617042	2006
Global	Peptic ulcer disease	Both	109.4034828	108.709138	110.0978277	2007
Global	Peptic ulcer disease	Both	105.691669	105.0169186	106.3664194	2008
Global	Peptic ulcer disease	Both	100.3494012	99.69885654	100.9999458	2009
Global	Peptic ulcer disease	Both	96.51467751	95.88373649	97.14561853	2010
Global	Peptic ulcer disease	Both	92.85388343	92.24171983	93.46604703	2011
Global	Peptic ulcer disease	Both	89.19497267	88.60168709	89.78825825	2012
Global	Peptic ulcer disease	Both	86.70211402	86.12289339	87.28133466	2013
Global	Peptic ulcer disease	Both	83.48901098	82.92625532	84.05176664	2014
Global	Peptic ulcer disease	Both	81.56720301	81.01728048	82.11712553	2015
Global	Peptic ulcer disease	Both	79.98002615	79.44130912	80.51874317	2016
Global	Peptic ulcer disease	Both	78.26977674	77.74213659	78.79741689	2017
Global	Peptic ulcer disease	Both	76.27450552	75.75825218	76.79075886	2018
Global	Peptic ulcer disease	Both	74.44097845	73.93585527	74.94610163	2019
Global	Peptic ulcer disease	Both	73.00356117	67.12645637	78.88066597	2020
Global	Peptic ulcer disease	Both	71.41599406	65.27258403	77.55940409	2021

Global	Peptic ulcer disease	Both	69.87788101	63.24310852	76.51265351	2022
Global	Peptic ulcer disease	Both	68.38735581	61.04408438	75.73062725	2023
Global	Peptic ulcer disease	Both	66.94419724	58.70312114	75.18527334	2024
Global	Peptic ulcer disease	Both	65.55054023	56.25606187	74.84501859	2025
Global	Peptic ulcer disease	Both	64.20608948	53.73668838	74.67549058	2026
Global	Peptic ulcer disease	Both	62.91148016	51.17212416	74.65083616	2027
Global	Peptic ulcer disease	Both	61.66488148	48.580059	74.74970396	2028
Global	Peptic ulcer disease	Both	60.46530452	45.97282691	74.95778214	2029
Global	Peptic ulcer disease	Both	59.31349377	43.36014029	75.26684724	2030
High SDI	Peptic ulcer disease	Male	99.10344207	98.80356185	99.40332229	1990
High SDI	Peptic ulcer disease	Male	95.42431168	95.13625686	95.71236649	1991
High SDI	Peptic ulcer disease	Male	92.0643328	91.78470265	92.34396295	1992
High SDI	Peptic ulcer disease	Male	89.63233722	89.35935928	89.90531517	1993
High SDI	Peptic ulcer disease	Male	86.26733233	86.0023638	86.53230086	1994
High SDI	Peptic ulcer disease	Male	83.08564748	82.82838308	83.34291188	1995
High SDI	Peptic ulcer disease	Male	78.54641588	78.2989063	78.79392545	1996
High SDI	Peptic ulcer disease	Male	74.04052172	73.80295042	74.27809301	1997
High SDI	Peptic ulcer disease	Male	70.27252509	70.04364745	70.50140272	1998
High SDI	Peptic ulcer disease	Male	66.33583251	66.11594322	66.55572181	1999
High SDI	Peptic ulcer disease	Male	61.989621	61.77964859	62.19959341	2000
High SDI	Peptic ulcer disease	Male	58.4527425	58.25119927	58.65428573	2001
High SDI	Peptic ulcer disease	Male	55.69771062	55.50332803	55.8920932	2002
High SDI	Peptic ulcer disease	Male	53.13678719	52.94915574	53.32441864	2003
High SDI	Peptic ulcer disease	Male	50.02312197	49.84328786	50.20295607	2004
High SDI	Peptic ulcer disease	Male	47.92150568	47.74763381	48.09537755	2005
High SDI	Peptic ulcer disease	Male	45.55286917	45.38534394	45.72039439	2006
High SDI	Peptic ulcer disease	Male	43.44236866	43.28082809	43.60390923	2007
High SDI	Peptic ulcer disease	Male	41.55010419	41.39405949	41.7061489	2008
High SDI	Peptic ulcer disease	Male	39.73120961	39.58042109	39.88199813	2009
High SDI	Peptic ulcer disease	Male	38.03717224	37.89137043	38.18297404	2010
High SDI	Peptic ulcer disease	Male	36.67086362	36.52921255	36.8125147	2011
High SDI	Peptic ulcer disease	Male	35.29416611	35.15675273	35.43157949	2012
High SDI	Peptic ulcer disease	Male	34.16439924	34.03062808	34.2981704	2013
High SDI	Peptic ulcer disease	Male	33.01942628	32.88923926	33.14961329	2014
High SDI	Peptic ulcer disease	Male	32.46452577	32.33667442	32.59237712	2015
High SDI	Peptic ulcer disease	Male	31.97103772	31.84520589	32.09686955	2016
High SDI	Peptic ulcer disease	Male	31.36265704	31.23910481	31.48620927	2017
High SDI	Peptic ulcer disease	Male	31.05596686	30.93398355	31.17795016	2018
High SDI	Peptic ulcer disease	Male	30.59986608	30.47760418	30.72212798	2019
High SDI	Peptic ulcer disease	Male	30.79742493	29.87279158	31.72205827	2020
High SDI	Peptic ulcer disease	Male	30.45505814	29.07788664	31.83222964	2021
High SDI	Peptic ulcer disease	Male	30.11944056	28.17188326	32.06699787	2022
High SDI	Peptic ulcer disease	Male	29.79498364	27.19436751	32.39559978	2023
High SDI	Peptic ulcer disease	Male	29.48218706	26.16345035	32.80092377	2024
High SDI	Peptic ulcer disease	Male	29.18208884	25.09022143	33.27395626	2025
High SDI	Peptic ulcer disease	Male	28.88498838	23.9754813	33.79449546	2026
High SDI	Peptic ulcer disease	Male	28.58985561	22.82518121	34.35453	2027
High SDI	Peptic ulcer disease	Male	28.3018067	21.64816305	34.95545035	2028
High SDI	Peptic ulcer disease	Male	28.02336369	20.44922503	35.59750236	2029
High SDI	Peptic ulcer disease	Male	27.75581631	19.23121634	36.28041628	2030
High SDI	Peptic ulcer disease	Female	51.37036814	51.18859109	51.55214518	1990
High SDI	Peptic ulcer disease	Female	49.75133225	49.57670756	49.92595695	1991
High SDI	Peptic ulcer disease	Female	48.1453841	47.97541118	48.31535703	1992
High SDI	Peptic ulcer disease	Female	47.2370121	47.07014973	47.40387447	1993
High SDI	Peptic ulcer disease	Female	45.53529584	45.37287543	45.69771625	1994
High SDI	Peptic ulcer disease	Female	44.18415126	44.0255667	44.34273582	1995
High SDI	Peptic ulcer disease	Female	42.22757234	42.07385559	42.38128909	1996
High SDI	Peptic ulcer disease	Female	40.25475735	40.10597752	40.40353718	1997

High SDI	Peptic ulcer disease	Female	38.2630615	38.11924007	38.40688292	1998
High SDI	Peptic ulcer disease	Female	36.26994927	36.13107361	36.40882493	1999
High SDI	Peptic ulcer disease	Female	34.03645724	33.90303478	34.1698797	2000
High SDI	Peptic ulcer disease	Female	32.27152257	32.14261045	32.4004347	2001
High SDI	Peptic ulcer disease	Female	30.80435258	30.67957537	30.9291298	2002
High SDI	Peptic ulcer disease	Female	29.50322738	29.38214044	29.62431431	2003
High SDI	Peptic ulcer disease	Female	27.86374306	27.74706187	27.98042426	2004
High SDI	Peptic ulcer disease	Female	26.61809242	26.50501541	26.73116943	2005
High SDI	Peptic ulcer disease	Female	25.34789726	25.23846019	25.45733434	2006
High SDI	Peptic ulcer disease	Female	24.25469986	24.14861093	24.3607888	2007
High SDI	Peptic ulcer disease	Female	23.30471095	23.20163717	23.40778474	2008
High SDI	Peptic ulcer disease	Female	22.29770676	22.19769838	22.39771513	2009
High SDI	Peptic ulcer disease	Female	21.39727066	21.3001121	21.49442923	2010
High SDI	Peptic ulcer disease	Female	20.75129904	20.65632361	20.84627447	2011
High SDI	Peptic ulcer disease	Female	20.06451684	19.97191236	20.15712132	2012
High SDI	Peptic ulcer disease	Female	19.49029095	19.39965992	19.58092197	2013
High SDI	Peptic ulcer disease	Female	18.88177305	18.79311009	18.97043602	2014
High SDI	Peptic ulcer disease	Female	18.62425143	18.53674571	18.71175714	2015
High SDI	Peptic ulcer disease	Female	18.50464266	18.4178234	18.59146192	2016
High SDI	Peptic ulcer disease	Female	18.3201853	18.23428537	18.40608524	2017
High SDI	Peptic ulcer disease	Female	18.29182903	18.20641732	18.37724075	2018
High SDI	Peptic ulcer disease	Female	18.21398871	18.12711761	18.30085981	2019
High SDI	Peptic ulcer disease	Female	18.39952956	17.84403464	18.95502449	2020
High SDI	Peptic ulcer disease	Female	18.35356542	17.53051629	19.17661456	2021
High SDI	Peptic ulcer disease	Female	18.31375907	17.15007354	19.47744459	2022
High SDI	Peptic ulcer disease	Female	18.28277765	16.72362925	19.84192605	2023
High SDI	Peptic ulcer disease	Female	18.26102789	16.260076	20.26197977	2024
High SDI	Peptic ulcer disease	Female	18.2502659	15.76543245	20.73509936	2025
High SDI	Peptic ulcer disease	Female	18.24359022	15.23820889	21.24897155	2026
High SDI	Peptic ulcer disease	Female	18.2386243	14.67952921	21.79771939	2027
High SDI	Peptic ulcer disease	Female	18.23939219	14.09440704	22.38437733	2028
High SDI	Peptic ulcer disease	Female	18.24849718	13.48532688	23.01166748	2029
High SDI	Peptic ulcer disease	Female	18.26770394	12.85347108	23.6819368	2030
High SDI	Peptic ulcer disease	Both	72.83833065	71.53270401	74.1439573	1990
High SDI	Peptic ulcer disease	Both	70.34347549	69.07359604	71.61335495	1991
High SDI	Peptic ulcer disease	Both	67.99675223	66.76075543	69.23274902	1992
High SDI	Peptic ulcer disease	Both	66.45922998	65.24879078	67.66966919	1993
High SDI	Peptic ulcer disease	Both	64.05735523	62.87898018	65.23573029	1994
High SDI	Peptic ulcer disease	Both	61.91560297	60.76744883	63.06375712	1995
High SDI	Peptic ulcer disease	Both	58.83480631	57.72502848	59.94458414	1996
High SDI	Peptic ulcer disease	Both	55.76266431	54.69123306	56.83409555	1997
High SDI	Peptic ulcer disease	Both	52.98371873	51.94872365	54.01871382	1998
High SDI	Peptic ulcer disease	Both	50.13913449	49.14096224	51.13730673	1999
High SDI	Peptic ulcer disease	Both	46.9893574	46.03051393	47.94820086	2000
High SDI	Peptic ulcer disease	Both	44.44716136	43.52139429	45.37292844	2001
High SDI	Peptic ulcer disease	Both	42.41530687	41.51875884	43.3118549	2002
High SDI	Peptic ulcer disease	Both	40.56565161	39.6957736	41.43552961	2003
High SDI	Peptic ulcer disease	Both	38.26946984	37.43084434	39.10809534	2004
High SDI	Peptic ulcer disease	Both	36.64847114	35.83431976	37.46262253	2005
High SDI	Peptic ulcer disease	Both	34.88549693	34.09720212	35.67379175	2006
High SDI	Peptic ulcer disease	Both	33.33256399	32.56839252	34.09673547	2007
High SDI	Peptic ulcer disease	Both	31.94981167	31.20803352	32.69158981	2008
High SDI	Peptic ulcer disease	Both	30.56613116	29.84649312	31.2857692	2009
High SDI	Peptic ulcer disease	Both	29.2934926	28.59491526	29.99206994	2010
High SDI	Peptic ulcer disease	Both	28.31447526	27.63267932	28.9962712	2011
High SDI	Peptic ulcer disease	Both	27.30723833	26.64282666	27.97165001	2012
High SDI	Peptic ulcer disease	Both	26.47964213	25.830041	27.12924327	2013
High SDI	Peptic ulcer disease	Both	25.62575137	24.9907423	26.26076043	2014

High SDI	Peptic ulcer disease	Both	25.24203075	24.61638735	25.86767415	2015
High SDI	Peptic ulcer disease	Both	24.96039116	24.34197858	25.57880375	2016
High SDI	Peptic ulcer disease	Both	24.58462547	23.97472197	25.19452898	2017
High SDI	Peptic ulcer disease	Both	24.43543559	23.83170828	25.0391629	2018
High SDI	Peptic ulcer disease	Both	24.18826486	23.59078845	24.78574128	2019
High SDI	Peptic ulcer disease	Both	24.37304	22.6818279	26.0642521	2020
High SDI	Peptic ulcer disease	Both	24.19712989	22.32766386	26.06659591	2021
High SDI	Peptic ulcer disease	Both	24.02967219	21.86761936	26.19172502	2022
High SDI	Peptic ulcer disease	Both	23.87530413	21.31587215	26.43473611	2023
High SDI	Peptic ulcer disease	Both	23.73579643	20.68974226	26.78185061	2024
High SDI	Peptic ulcer disease	Both	23.6134517	20.00509841	27.221805	2025
High SDI	Peptic ulcer disease	Both	23.50077637	19.26776564	27.73378709	2026
High SDI	Peptic ulcer disease	Both	23.39663123	18.48519698	28.30806549	2027
High SDI	Peptic ulcer disease	Both	23.30644957	17.66661386	28.94628527	2028
High SDI	Peptic ulcer disease	Both	23.23384304	16.8166683	29.65101779	2029
High SDI	Peptic ulcer disease	Both	23.18132911	15.93727912	30.42537911	2030
High-middle SDI	Peptic ulcer disease	Male	168.7822852	168.3973347	169.1672356	1990
High-middle SDI	Peptic ulcer disease	Male	166.1119031	165.736936	166.4868702	1991
High-middle SDI	Peptic ulcer disease	Male	167.8429485	167.4719641	168.213933	1992
High-middle SDI	Peptic ulcer disease	Male	171.7350151	171.3653319	172.1046983	1993
High-middle SDI	Peptic ulcer disease	Male	171.9195763	171.554479	172.2846737	1994
High-middle SDI	Peptic ulcer disease	Male	165.3620721	165.0075339	165.7166103	1995
High-middle SDI	Peptic ulcer disease	Male	154.0310515	153.6920368	154.3700662	1996
High-middle SDI	Peptic ulcer disease	Male	143.5454806	143.2213779	143.8695832	1997
High-middle SDI	Peptic ulcer disease	Male	136.112865	135.8003523	136.4253778	1998
High-middle SDI	Peptic ulcer disease	Male	133.908449	133.6024488	134.2144493	1999
High-middle SDI	Peptic ulcer disease	Male	130.5497105	130.251273	130.848148	2000
High-middle SDI	Peptic ulcer disease	Male	125.2727684	124.9835706	125.5619663	2001
High-middle SDI	Peptic ulcer disease	Male	121.4903463	121.2085296	121.772163	2002
High-middle SDI	Peptic ulcer disease	Male	118.3837032	118.1084921	118.6589143	2003
High-middle SDI	Peptic ulcer disease	Male	114.1878322	113.9205967	114.4550678	2004
High-middle SDI	Peptic ulcer disease	Male	111.8934101	111.6322655	112.1545547	2005
High-middle SDI	Peptic ulcer disease	Male	102.7845874	102.5372127	103.031962	2006
High-middle SDI	Peptic ulcer disease	Male	96.98564258	96.74839414	97.22289101	2007
High-middle SDI	Peptic ulcer disease	Male	93.8563096	93.62586365	94.08675554	2008
High-middle SDI	Peptic ulcer disease	Male	88.48054959	88.25919623	88.70190295	2009
High-middle SDI	Peptic ulcer disease	Male	85.77188566	85.55658101	85.98719031	2010
High-middle SDI	Peptic ulcer disease	Male	81.16689238	80.95993532	81.37384944	2011
High-middle SDI	Peptic ulcer disease	Male	78.20762796	78.0070165	78.40823942	2012
High-middle SDI	Peptic ulcer disease	Male	75.33263615	75.1381482	75.5271241	2013
High-middle SDI	Peptic ulcer disease	Male	73.39638034	73.2068485	73.58591219	2014
High-middle SDI	Peptic ulcer disease	Male	72.75408932	72.56769867	72.94047997	2015
High-middle SDI	Peptic ulcer disease	Male	70.65725258	70.47574645	70.83875871	2016
High-middle SDI	Peptic ulcer disease	Male	67.0022041	66.8274274	67.17698081	2017
High-middle SDI	Peptic ulcer disease	Male	64.32961461	64.16016515	64.49906407	2018
High-middle SDI	Peptic ulcer disease	Male	62.62556112	62.45933062	62.79179163	2019
High-middle SDI	Peptic ulcer disease	Male	62.25533454	59.19389493	65.31677415	2020
High-middle SDI	Peptic ulcer disease	Male	60.5428975	55.65779623	65.42799878	2021
High-middle SDI	Peptic ulcer disease	Male	58.89030025	51.77975527	66.00084524	2022
High-middle SDI	Peptic ulcer disease	Male	57.33795252	47.7546075	66.92129754	2023
High-middle SDI	Peptic ulcer disease	Male	55.84717422	43.62112053	68.0732279	2024
High-middle SDI	Peptic ulcer disease	Male	54.35706768	39.37635473	69.33778062	2025
High-middle SDI	Peptic ulcer disease	Male	52.83668626	35.04910814	70.62426437	2026
High-middle SDI	Peptic ulcer disease	Male	51.3639363	30.73175028	71.99612232	2027
High-middle SDI	Peptic ulcer disease	Male	49.98227587	26.46917569	73.49537605	2028
High-middle SDI	Peptic ulcer disease	Male	48.66105331	22.2509037	75.07120293	2029
High-middle SDI	Peptic ulcer disease	Male	47.34399361	18.05991505	76.62807216	2030
High-middle SDI	Peptic ulcer disease	Female	62.71452443	62.51019422	62.91885465	1990

High-middle SDI	Peptic ulcer disease	Female	61.36225975	61.16349986	61.56101964	1991
High-middle SDI	Peptic ulcer disease	Female	60.70156031	60.50595966	60.89716096	1992
High-middle SDI	Peptic ulcer disease	Female	61.13676401	60.94217334	61.33135467	1993
High-middle SDI	Peptic ulcer disease	Female	60.89231027	60.69972478	61.08489575	1994
High-middle SDI	Peptic ulcer disease	Female	58.70867074	58.52107313	58.89626835	1995
High-middle SDI	Peptic ulcer disease	Female	55.69635466	55.51539512	55.87731419	1996
High-middle SDI	Peptic ulcer disease	Female	53.22299342	53.04774426	53.39824258	1997
High-middle SDI	Peptic ulcer disease	Female	51.07140762	50.90125122	51.24156402	1998
High-middle SDI	Peptic ulcer disease	Female	50.20049063	50.03338008	50.36760118	1999
High-middle SDI	Peptic ulcer disease	Female	49.31325365	49.14909767	49.47740964	2000
High-middle SDI	Peptic ulcer disease	Female	48.21083224	48.05014643	48.37151804	2001
High-middle SDI	Peptic ulcer disease	Female	47.48794502	47.33009779	47.64579224	2002
High-middle SDI	Peptic ulcer disease	Female	46.7450482	46.59003482	46.90006158	2003
High-middle SDI	Peptic ulcer disease	Female	45.37801872	45.22688893	45.52914851	2004
High-middle SDI	Peptic ulcer disease	Female	44.563077	44.41483863	44.71131538	2005
High-middle SDI	Peptic ulcer disease	Female	41.97541633	41.83322525	42.1176074	2006
High-middle SDI	Peptic ulcer disease	Female	40.27473144	40.13708267	40.41238021	2007
High-middle SDI	Peptic ulcer disease	Female	39.00874538	38.8747939	39.14269686	2008
High-middle SDI	Peptic ulcer disease	Female	37.4891408	37.35925502	37.61902657	2009
High-middle SDI	Peptic ulcer disease	Female	36.55648143	36.42957092	36.68339193	2010
High-middle SDI	Peptic ulcer disease	Female	35.26555451	35.14234689	35.38876213	2011
High-middle SDI	Peptic ulcer disease	Female	34.20309249	34.08317105	34.32301392	2012
High-middle SDI	Peptic ulcer disease	Female	33.40260133	33.28535018	33.51985248	2013
High-middle SDI	Peptic ulcer disease	Female	32.73647799	32.62164091	32.85131508	2014
High-middle SDI	Peptic ulcer disease	Female	32.58074933	32.46727876	32.69421989	2015
High-middle SDI	Peptic ulcer disease	Female	31.9043171	31.7931561	32.0154781	2016
High-middle SDI	Peptic ulcer disease	Female	30.91804661	30.80969363	31.0263996	2017
High-middle SDI	Peptic ulcer disease	Female	30.00854756	29.902697	30.11439811	2018
High-middle SDI	Peptic ulcer disease	Female	29.28248967	29.17796409	29.38701525	2019
High-middle SDI	Peptic ulcer disease	Female	29.42553392	28.23176708	30.61930076	2020
High-middle SDI	Peptic ulcer disease	Female	28.81749075	27.11396722	30.52101428	2021
High-middle SDI	Peptic ulcer disease	Female	28.21789944	25.87221022	30.56358867	2022
High-middle SDI	Peptic ulcer disease	Female	27.63801881	24.56396552	30.7120721	2023
High-middle SDI	Peptic ulcer disease	Female	27.07532669	23.21159414	30.93905924	2024
High-middle SDI	Peptic ulcer disease	Female	26.51742979	21.8196393	31.21522027	2025
High-middle SDI	Peptic ulcer disease	Female	25.95375656	20.39332302	31.51419011	2026
High-middle SDI	Peptic ulcer disease	Female	25.39693784	18.95308326	31.84079241	2027
High-middle SDI	Peptic ulcer disease	Female	24.85855098	17.51408596	32.20301599	2028
High-middle SDI	Peptic ulcer disease	Female	24.33740583	16.07920033	32.59561133	2029
High-middle SDI	Peptic ulcer disease	Female	23.82139286	14.64384325	32.99894247	2030
High-middle SDI	Peptic ulcer disease	Both	111.0013886	109.376751	112.6260262	1990
High-middle SDI	Peptic ulcer disease	Both	109.2099454	107.6186878	110.8012029	1991
High-middle SDI	Peptic ulcer disease	Both	109.8922771	108.3176295	111.4669247	1992
High-middle SDI	Peptic ulcer disease	Both	112.2336198	110.6630355	113.8042042	1993
High-middle SDI	Peptic ulcer disease	Both	112.3843971	110.8288154	113.9399788	1994
High-middle SDI	Peptic ulcer disease	Both	108.1783229	106.6624095	109.6942362	1995
High-middle SDI	Peptic ulcer disease	Both	101.2456498	99.78997114	102.7013284	1996
High-middle SDI	Peptic ulcer disease	Both	95.03874546	93.63759947	96.43989146	1997
High-middle SDI	Peptic ulcer disease	Both	90.44955719	89.09204964	91.80706475	1998
High-middle SDI	Peptic ulcer disease	Both	89.09016903	87.7566671	90.42367096	1999
High-middle SDI	Peptic ulcer disease	Both	87.13684266	85.83074581	88.44293952	2000
High-middle SDI	Peptic ulcer disease	Both	84.09874547	82.82726542	85.37022553	2001
High-middle SDI	Peptic ulcer disease	Both	81.97014545	80.72822994	83.21206096	2002
High-middle SDI	Peptic ulcer disease	Both	80.15919976	78.94414619	81.37425332	2003
High-middle SDI	Peptic ulcer disease	Both	77.48695095	76.30354366	78.67035823	2004
High-middle SDI	Peptic ulcer disease	Both	76.05022119	74.89031458	77.2101278	2005
High-middle SDI	Peptic ulcer disease	Both	70.42944981	69.32293399	71.53596563	2006
High-middle SDI	Peptic ulcer disease	Both	66.83716333	65.7701992	67.90412745	2007

High-middle SDI	Peptic ulcer disease	Both	64.72989539	63.69176096	65.76802983	2008
High-middle SDI	Peptic ulcer disease	Both	61.37626824	60.37420209	62.37833438	2009
High-middle SDI	Peptic ulcer disease	Both	59.61518479	58.63854413	60.59182545	2010
High-middle SDI	Peptic ulcer disease	Both	56.75737673	55.81424915	57.70050432	2011
High-middle SDI	Peptic ulcer disease	Both	54.80620394	53.88956012	55.72284776	2012
High-middle SDI	Peptic ulcer disease	Both	53.05985951	52.1662582	53.95346082	2013
High-middle SDI	Peptic ulcer disease	Both	51.85185705	50.97747188	52.72624223	2014
High-middle SDI	Peptic ulcer disease	Both	51.51861881	50.65628381	52.38095382	2015
High-middle SDI	Peptic ulcer disease	Both	50.17960523	49.33780633	51.02140413	2016
High-middle SDI	Peptic ulcer disease	Both	47.95921538	47.14295874	48.77547201	2017
High-middle SDI	Peptic ulcer disease	Both	46.26334717	45.46719351	47.05950082	2018
High-middle SDI	Peptic ulcer disease	Both	45.10159178	44.32144478	45.88173878	2019
High-middle SDI	Peptic ulcer disease	Both	44.71305911	40.20474425	49.22137398	2020
High-middle SDI	Peptic ulcer disease	Both	43.64538	38.58250927	48.70825072	2021
High-middle SDI	Peptic ulcer disease	Both	42.6213825	36.63162703	48.61113796	2022
High-middle SDI	Peptic ulcer disease	Both	41.67377677	34.45331471	48.89423883	2023
High-middle SDI	Peptic ulcer disease	Both	40.78974172	32.10485179	49.47463166	2024
High-middle SDI	Peptic ulcer disease	Both	39.93651282	29.60806331	50.26496232	2025
High-middle SDI	Peptic ulcer disease	Both	39.09365734	26.98720283	51.20011184	2026
High-middle SDI	Peptic ulcer disease	Both	38.3072383	24.29812556	52.31635104	2027
High-middle SDI	Peptic ulcer disease	Both	37.60989361	21.56569839	53.65408883	2028
High-middle SDI	Peptic ulcer disease	Both	36.99155293	18.77291241	55.21019345	2029
High-middle SDI	Peptic ulcer disease	Both	36.4206123	15.88691169	56.9543129	2030
Middle SDI	Peptic ulcer disease	Male	211.6437423	211.2201823	212.0673024	1990
Middle SDI	Peptic ulcer disease	Male	206.7385595	206.3277127	207.1494062	1991
Middle SDI	Peptic ulcer disease	Male	201.4768567	201.0774023	201.8763112	1992
Middle SDI	Peptic ulcer disease	Male	195.2350812	194.8474025	195.6227599	1993
Middle SDI	Peptic ulcer disease	Male	189.1136829	188.7373015	189.4900644	1994
Middle SDI	Peptic ulcer disease	Male	182.7260634	182.3606477	183.0914792	1995
Middle SDI	Peptic ulcer disease	Male	176.2518606	175.8974358	176.6062853	1996
Middle SDI	Peptic ulcer disease	Male	168.177649	167.8360482	168.5192498	1997
Middle SDI	Peptic ulcer disease	Male	160.5411907	160.2121569	160.8702246	1998
Middle SDI	Peptic ulcer disease	Male	153.9612139	153.643929	154.2784989	1999
Middle SDI	Peptic ulcer disease	Male	149.0832472	148.7756519	149.3908426	2000
Middle SDI	Peptic ulcer disease	Male	143.7580954	143.4601729	144.0560179	2001
Middle SDI	Peptic ulcer disease	Male	139.9022581	139.6126722	140.1918439	2002
Middle SDI	Peptic ulcer disease	Male	135.5580414	135.2775167	135.838566	2003
Middle SDI	Peptic ulcer disease	Male	131.221686	130.9498727	131.4934993	2004
Middle SDI	Peptic ulcer disease	Male	126.5546361	126.2917009	126.8175713	2005
Middle SDI	Peptic ulcer disease	Male	119.8005856	119.5492897	120.0518815	2006
Middle SDI	Peptic ulcer disease	Male	113.986848	113.7461561	114.2275398	2007
Middle SDI	Peptic ulcer disease	Male	110.6009899	110.3678086	110.8341711	2008
Middle SDI	Peptic ulcer disease	Male	106.3408596	106.1156111	106.566108	2009
Middle SDI	Peptic ulcer disease	Male	102.3939822	102.1761136	102.6118507	2010
Middle SDI	Peptic ulcer disease	Male	98.58771239	98.37731368	98.7981111	2011
Middle SDI	Peptic ulcer disease	Male	94.49722905	94.29435877	94.70009934	2012
Middle SDI	Peptic ulcer disease	Male	91.0105605	90.81421366	91.20690733	2013
Middle SDI	Peptic ulcer disease	Male	87.04780881	86.85868444	87.23693318	2014
Middle SDI	Peptic ulcer disease	Male	83.72153552	83.53892636	83.90414467	2015
Middle SDI	Peptic ulcer disease	Male	81.46459664	81.28740403	81.64178926	2016
Middle SDI	Peptic ulcer disease	Male	78.58288051	78.41161134	78.75414967	2017
Middle SDI	Peptic ulcer disease	Male	75.62415017	75.45864531	75.78965503	2018
Middle SDI	Peptic ulcer disease	Male	73.29571702	73.13489383	73.45654021	2019
Middle SDI	Peptic ulcer disease	Male	72.14045472	69.60149225	74.67941718	2020
Middle SDI	Peptic ulcer disease	Male	69.98424655	66.80397932	73.16451377	2021
Middle SDI	Peptic ulcer disease	Male	67.88238519	63.87709021	71.88768016	2022
Middle SDI	Peptic ulcer disease	Male	65.83791144	60.88984883	70.78597404	2023
Middle SDI	Peptic ulcer disease	Male	63.84994339	57.88423982	69.81564696	2024

Middle SDI	Peptic ulcer disease	Male	61.91564425	54.8859513	68.94533721	2025
Middle SDI	Peptic ulcer disease	Male	60.03079016	51.9143061	68.14727421	2026
Middle SDI	Peptic ulcer disease	Male	58.19535526	48.98447055	67.40623997	2027
Middle SDI	Peptic ulcer disease	Male	56.41173168	46.10825588	66.71520747	2028
Middle SDI	Peptic ulcer disease	Male	54.67835668	43.29078904	66.06592432	2029
Middle SDI	Peptic ulcer disease	Male	52.99189314	40.53471087	65.44907541	2030
Middle SDI	Peptic ulcer disease	Female	124.4247222	124.1164494	124.732995	1990
Middle SDI	Peptic ulcer disease	Female	120.6191403	120.3205247	120.9177559	1991
Middle SDI	Peptic ulcer disease	Female	118.9120465	118.6198295	119.2042636	1992
Middle SDI	Peptic ulcer disease	Female	114.5324898	114.2492697	114.8157098	1993
Middle SDI	Peptic ulcer disease	Female	111.0643842	110.7893428	111.3394257	1994
Middle SDI	Peptic ulcer disease	Female	106.7690052	106.5027646	107.0352459	1995
Middle SDI	Peptic ulcer disease	Female	102.0263614	101.7694552	102.2832677	1996
Middle SDI	Peptic ulcer disease	Female	98.58289647	98.33416182	98.83163112	1997
Middle SDI	Peptic ulcer disease	Female	94.87934183	94.63918337	95.1195003	1998
Middle SDI	Peptic ulcer disease	Female	91.75921021	91.52660568	91.99181473	1999
Middle SDI	Peptic ulcer disease	Female	89.36588999	89.1395226	89.59225738	2000
Middle SDI	Peptic ulcer disease	Female	86.5078877	86.28814295	86.72763245	2001
Middle SDI	Peptic ulcer disease	Female	84.52591751	84.31159407	84.74024096	2002
Middle SDI	Peptic ulcer disease	Female	81.95573542	81.74771546	82.16375538	2003
Middle SDI	Peptic ulcer disease	Female	78.88812462	78.68716046	79.08908877	2004
Middle SDI	Peptic ulcer disease	Female	75.72624393	75.53228658	75.92020127	2005
Middle SDI	Peptic ulcer disease	Female	71.90922261	71.72316629	72.09527893	2006
Middle SDI	Peptic ulcer disease	Female	68.16457385	67.9864595	68.34268821	2007
Middle SDI	Peptic ulcer disease	Female	65.3126051	65.14118164	65.48402856	2008
Middle SDI	Peptic ulcer disease	Female	62.34756879	62.18294017	62.51219741	2009
Middle SDI	Peptic ulcer disease	Female	60.00301578	59.84411524	60.16191631	2010
Middle SDI	Peptic ulcer disease	Female	57.65410575	57.50086886	57.80734264	2011
Middle SDI	Peptic ulcer disease	Female	55.21049644	55.06296012	55.35803276	2012
Middle SDI	Peptic ulcer disease	Female	53.57204553	53.4289933	53.71509777	2013
Middle SDI	Peptic ulcer disease	Female	51.29141937	51.1536521	51.42918663	2014
Middle SDI	Peptic ulcer disease	Female	49.27885864	49.14601462	49.41170266	2015
Middle SDI	Peptic ulcer disease	Female	48.05287143	47.92389732	48.18184555	2016
Middle SDI	Peptic ulcer disease	Female	46.96287789	46.83746298	47.0882928	2017
Middle SDI	Peptic ulcer disease	Female	45.76219869	45.64028886	45.88410852	2018
Middle SDI	Peptic ulcer disease	Female	44.67954926	44.56071249	44.79838602	2019
Middle SDI	Peptic ulcer disease	Female	44.0897783	42.21976602	45.95979058	2020
Middle SDI	Peptic ulcer disease	Female	43.12168588	40.74995884	45.49341292	2021
Middle SDI	Peptic ulcer disease	Female	42.17113967	39.14819339	45.19408595	2022
Middle SDI	Peptic ulcer disease	Female	41.23615644	37.4606456	45.01166729	2023
Middle SDI	Peptic ulcer disease	Female	40.31621013	35.71849674	44.91392352	2024
Middle SDI	Peptic ulcer disease	Female	39.40956798	33.94147085	44.8776651	2025
Middle SDI	Peptic ulcer disease	Female	38.51692542	32.14778557	44.88606526	2026
Middle SDI	Peptic ulcer disease	Female	37.64200995	30.35201515	44.93200476	2027
Middle SDI	Peptic ulcer disease	Female	36.78318505	28.56025589	45.0061142	2028
Middle SDI	Peptic ulcer disease	Female	35.93978566	26.77710344	45.10246788	2029
Middle SDI	Peptic ulcer disease	Female	35.10918402	25.00508376	45.21328429	2030
Middle SDI	Peptic ulcer disease	Both	167.6428036	165.5898308	169.6957765	1990
Middle SDI	Peptic ulcer disease	Both	163.2774751	161.2823707	165.2725795	1991
Middle SDI	Peptic ulcer disease	Both	159.813641	157.8674051	161.7598769	1992
Middle SDI	Peptic ulcer disease	Both	154.5047862	152.6178024	156.39177	1993
Middle SDI	Peptic ulcer disease	Both	149.6913807	147.8580406	151.5247208	1994
Middle SDI	Peptic ulcer disease	Both	144.3245298	142.5461896	146.10287	1995
Middle SDI	Peptic ulcer disease	Both	138.674623	136.9543626	140.3948833	1996
Middle SDI	Peptic ulcer disease	Both	132.9038498	131.2413082	134.5663915	1997
Middle SDI	Peptic ulcer disease	Both	127.2151064	125.6100147	128.8201981	1998
Middle SDI	Peptic ulcer disease	Both	122.387191	120.8331753	123.9412066	1999
Middle SDI	Peptic ulcer disease	Both	118.7735879	117.2631569	120.2840188	2000

Middle SDI	Peptic ulcer disease	Both	114.6890786	113.2243523	116.1538048	2001
Middle SDI	Peptic ulcer disease	Both	111.7831903	110.3579576	113.208423	2002
Middle SDI	Peptic ulcer disease	Both	108.3317226	106.9495316	109.7139137	2003
Middle SDI	Peptic ulcer disease	Both	104.5995826	103.2602888	105.9388763	2004
Middle SDI	Peptic ulcer disease	Both	100.6668971	99.37042638	101.9633679	2005
Middle SDI	Peptic ulcer disease	Both	95.4211383	94.17736684	96.66490975	2006
Middle SDI	Peptic ulcer disease	Both	90.65002928	89.45639832	91.84366025	2007
Middle SDI	Peptic ulcer disease	Both	87.49854582	86.34297249	88.65411915	2008
Middle SDI	Peptic ulcer disease	Both	83.8357624	82.7200169	84.9515079	2009
Middle SDI	Peptic ulcer disease	Both	80.65819228	79.57861001	81.73777455	2010
Middle SDI	Peptic ulcer disease	Both	77.57533566	76.53193401	78.61873731	2011
Middle SDI	Peptic ulcer disease	Both	74.29617923	73.28951022	75.30284824	2012
Middle SDI	Peptic ulcer disease	Both	71.73326249	70.75698515	72.70953983	2013
Middle SDI	Peptic ulcer disease	Both	68.6286579	67.68719413	69.57012166	2014
Middle SDI	Peptic ulcer disease	Both	65.96155632	65.05245999	66.87065265	2015
Middle SDI	Peptic ulcer disease	Both	64.22664397	63.34284216	65.11044579	2016
Middle SDI	Peptic ulcer disease	Both	62.26844515	61.40949373	63.12739657	2017
Middle SDI	Peptic ulcer disease	Both	60.21723283	59.38268859	61.05177708	2018
Middle SDI	Peptic ulcer disease	Both	58.52172654	57.70970726	59.33374583	2019
Middle SDI	Peptic ulcer disease	Both	57.78376263	51.85342338	63.71410188	2020
Middle SDI	Peptic ulcer disease	Both	56.23744	50.18403892	62.29084108	2021
Middle SDI	Peptic ulcer disease	Both	54.73209564	48.40678982	61.05740145	2022
Middle SDI	Peptic ulcer disease	Both	53.26995005	46.52340084	60.01649927	2023
Middle SDI	Peptic ulcer disease	Both	51.85143519	44.54664588	59.1562245	2024
Middle SDI	Peptic ulcer disease	Both	50.47453326	42.49453271	58.45453381	2025
Middle SDI	Peptic ulcer disease	Both	49.13786784	40.39059968	57.88513601	2026
Middle SDI	Peptic ulcer disease	Both	47.84372998	38.25781717	57.4296428	2027
Middle SDI	Peptic ulcer disease	Both	46.59308165	36.11325525	57.07290804	2028
Middle SDI	Peptic ulcer disease	Both	45.3853268	33.96813148	56.80252212	2029
Middle SDI	Peptic ulcer disease	Both	44.21754355	31.82906043	56.60602666	2030
Low-middle SDI	Peptic ulcer disease	Male	530.2685871	529.460588	531.0765861	1990
Low-middle SDI	Peptic ulcer disease	Male	523.2697028	522.4787666	524.060639	1991
Low-middle SDI	Peptic ulcer disease	Male	513.3212811	512.5472941	514.0952681	1992
Low-middle SDI	Peptic ulcer disease	Male	499.2781017	498.5231448	500.0330587	1993
Low-middle SDI	Peptic ulcer disease	Male	484.5609809	483.8258191	485.2961427	1994
Low-middle SDI	Peptic ulcer disease	Male	466.1988754	465.4854906	466.9122603	1995
Low-middle SDI	Peptic ulcer disease	Male	453.48589	452.7898091	454.1819709	1996
Low-middle SDI	Peptic ulcer disease	Male	450.4489236	449.7629203	451.134927	1997
Low-middle SDI	Peptic ulcer disease	Male	435.0515383	434.3845567	435.71852	1998
Low-middle SDI	Peptic ulcer disease	Male	412.7079789	412.0666173	413.3493404	1999
Low-middle SDI	Peptic ulcer disease	Male	395.4412166	394.8198817	396.0625515	2000
Low-middle SDI	Peptic ulcer disease	Male	378.3788603	377.7771623	378.9805582	2001
Low-middle SDI	Peptic ulcer disease	Male	359.938011	359.3570454	360.5189766	2002
Low-middle SDI	Peptic ulcer disease	Male	336.7219591	336.1654631	337.2784552	2003
Low-middle SDI	Peptic ulcer disease	Male	313.576076	313.0461123	314.1060396	2004
Low-middle SDI	Peptic ulcer disease	Male	303.5350616	303.0202097	304.0499134	2005
Low-middle SDI	Peptic ulcer disease	Male	289.9396206	289.4431949	290.4360463	2006
Low-middle SDI	Peptic ulcer disease	Male	274.6963105	274.2201102	275.1725107	2007
Low-middle SDI	Peptic ulcer disease	Male	265.9402981	265.4787098	266.4018865	2008
Low-middle SDI	Peptic ulcer disease	Male	248.315171	247.8758974	248.7544447	2009
Low-middle SDI	Peptic ulcer disease	Male	234.6922719	234.272173	235.1123708	2010
Low-middle SDI	Peptic ulcer disease	Male	223.4995261	223.0965546	223.9024977	2011
Low-middle SDI	Peptic ulcer disease	Male	213.3210563	212.9336248	213.7084878	2012
Low-middle SDI	Peptic ulcer disease	Male	206.4822208	206.1042566	206.860185	2013
Low-middle SDI	Peptic ulcer disease	Male	196.6959891	196.3303629	197.0616153	2014
Low-middle SDI	Peptic ulcer disease	Male	188.7304213	188.3779493	189.0828932	2015
Low-middle SDI	Peptic ulcer disease	Male	184.3440298	184.0016537	184.6864059	2016
Low-middle SDI	Peptic ulcer disease	Male	180.5382572	180.2045301	180.8719843	2017

Low-middle SDI	Peptic ulcer disease	Male	175.2328065	174.9084121	175.557201	2018
Low-middle SDI	Peptic ulcer disease	Male	169.5907327	169.275647	169.9058184	2019
Low-middle SDI	Peptic ulcer disease	Male	165.6285135	158.9524048	172.3046222	2020
Low-middle SDI	Peptic ulcer disease	Male	161.5585041	152.0122428	171.1047655	2021
Low-middle SDI	Peptic ulcer disease	Male	157.5762751	144.415738	170.7368123	2022
Low-middle SDI	Peptic ulcer disease	Male	153.652575	136.4202999	170.8848501	2023
Low-middle SDI	Peptic ulcer disease	Male	149.7945049	128.1873918	171.401618	2024
Low-middle SDI	Peptic ulcer disease	Male	146.0317693	119.8375833	172.2259553	2025
Low-middle SDI	Peptic ulcer disease	Male	142.3724533	111.4497557	173.295151	2026
Low-middle SDI	Peptic ulcer disease	Male	138.7930187	103.0562668	174.5297706	2027
Low-middle SDI	Peptic ulcer disease	Male	135.2715358	94.67995871	175.8631129	2028
Low-middle SDI	Peptic ulcer disease	Male	131.812253	86.35484583	177.2696602	2029
Low-middle SDI	Peptic ulcer disease	Male	128.4338454	78.11798189	178.7497088	2030
Low-middle SDI	Peptic ulcer disease	Female	348.9620594	348.2903547	349.633764	1990
Low-middle SDI	Peptic ulcer disease	Female	343.0947761	342.4390365	343.7505157	1991
Low-middle SDI	Peptic ulcer disease	Female	342.7098595	342.0642495	343.3554695	1992
Low-middle SDI	Peptic ulcer disease	Female	331.7535058	331.1262664	332.3807453	1993
Low-middle SDI	Peptic ulcer disease	Female	322.0658974	321.4566004	322.6751944	1994
Low-middle SDI	Peptic ulcer disease	Female	312.5616835	311.9694744	313.1538925	1995
Low-middle SDI	Peptic ulcer disease	Female	308.2483129	307.6685363	308.8280896	1996
Low-middle SDI	Peptic ulcer disease	Female	312.2751124	311.7016181	312.8486067	1997
Low-middle SDI	Peptic ulcer disease	Female	303.1859972	302.6282678	303.7437266	1998
Low-middle SDI	Peptic ulcer disease	Female	292.2758231	291.7354035	292.8162427	1999
Low-middle SDI	Peptic ulcer disease	Female	284.5473881	284.02094	285.0738362	2000
Low-middle SDI	Peptic ulcer disease	Female	275.6355047	275.1231283	276.1478811	2001
Low-middle SDI	Peptic ulcer disease	Female	265.2220764	264.7244726	265.7196801	2002
Low-middle SDI	Peptic ulcer disease	Female	252.4351622	251.9556384	252.9146861	2003
Low-middle SDI	Peptic ulcer disease	Female	236.7491744	236.2907871	237.2075617	2004
Low-middle SDI	Peptic ulcer disease	Female	231.058928	230.6125821	231.5052739	2005
Low-middle SDI	Peptic ulcer disease	Female	222.5034997	222.0718552	222.9351442	2006
Low-middle SDI	Peptic ulcer disease	Female	211.9549009	211.539575	212.3702268	2007
Low-middle SDI	Peptic ulcer disease	Female	202.1877928	201.7883176	202.587268	2008
Low-middle SDI	Peptic ulcer disease	Female	186.3972529	186.0212471	186.7732586	2009
Low-middle SDI	Peptic ulcer disease	Female	178.606849	178.2467114	178.9669866	2010
Low-middle SDI	Peptic ulcer disease	Female	171.7472623	171.4008202	172.0937043	2011
Low-middle SDI	Peptic ulcer disease	Female	161.7712542	161.4407195	162.101789	2012
Low-middle SDI	Peptic ulcer disease	Female	158.6628244	158.3405284	158.9851203	2013
Low-middle SDI	Peptic ulcer disease	Female	151.4643813	151.1540228	151.7747398	2014
Low-middle SDI	Peptic ulcer disease	Female	149.2613665	148.9586495	149.5640835	2015
Low-middle SDI	Peptic ulcer disease	Female	146.3461551	146.0517395	146.6405706	2016
Low-middle SDI	Peptic ulcer disease	Female	144.4377504	144.15015	144.7253509	2017
Low-middle SDI	Peptic ulcer disease	Female	140.1347385	139.8557632	140.4137138	2018
Low-middle SDI	Peptic ulcer disease	Female	136.0173888	135.746459	136.2883186	2019
Low-middle SDI	Peptic ulcer disease	Female	133.8745832	127.5512736	140.1978927	2020
Low-middle SDI	Peptic ulcer disease	Female	131.0488959	122.4450408	139.652751	2021
Low-middle SDI	Peptic ulcer disease	Female	128.249195	116.7311169	139.7672731	2022
Low-middle SDI	Peptic ulcer disease	Female	125.4649947	110.6239239	140.3060655	2023
Low-middle SDI	Peptic ulcer disease	Female	122.7123185	104.2691784	141.1554587	2024
Low-middle SDI	Peptic ulcer disease	Female	120.0165049	97.77046367	142.2625462	2025
Low-middle SDI	Peptic ulcer disease	Female	117.3668944	91.17970386	143.5540849	2026
Low-middle SDI	Peptic ulcer disease	Female	114.7468574	84.52862901	144.9650858	2027
Low-middle SDI	Peptic ulcer disease	Female	112.1473944	77.84544147	146.4493472	2028
Low-middle SDI	Peptic ulcer disease	Female	109.5813491	71.16641493	147.9962832	2029
Low-middle SDI	Peptic ulcer disease	Female	107.0681665	64.52568848	149.6106445	2030
Low-middle SDI	Peptic ulcer disease	Both	441.6882492	437.4514838	445.9250146	1990
Low-middle SDI	Peptic ulcer disease	Both	435.180851	431.0338549	439.3278471	1991
Low-middle SDI	Peptic ulcer disease	Both	429.8394017	425.7723807	433.9064226	1992
Low-middle SDI	Peptic ulcer disease	Both	417.1971224	413.2407132	421.1535316	1993

Low-middle SDI	Peptic ulcer disease	Both	404.8028888	400.9557303	408.6500474	1994
Low-middle SDI	Peptic ulcer disease	Both	390.6741147	386.9379611	394.4102684	1995
Low-middle SDI	Peptic ulcer disease	Both	381.9792545	378.3328646	385.6256444	1996
Low-middle SDI	Peptic ulcer disease	Both	382.2994582	378.7020569	385.8968595	1997
Low-middle SDI	Peptic ulcer disease	Both	369.9058184	366.4065817	373.4050552	1998
Low-middle SDI	Peptic ulcer disease	Both	353.2089153	349.8256448	356.5921858	1999
Low-middle SDI	Peptic ulcer disease	Both	340.5952433	337.311057	343.8794296	2000
Low-middle SDI	Peptic ulcer disease	Both	327.4903453	324.3081479	330.6725428	2001
Low-middle SDI	Peptic ulcer disease	Both	312.9754522	309.9019625	316.0489419	2002
Low-middle SDI	Peptic ulcer disease	Both	294.8619391	291.9137547	297.8101236	2003
Low-middle SDI	Peptic ulcer disease	Both	275.3800346	272.5618454	278.1982239	2004
Low-middle SDI	Peptic ulcer disease	Both	267.4598711	264.7183838	270.2013584	2005
Low-middle SDI	Peptic ulcer disease	Both	256.346871	253.6990124	258.9947296	2006
Low-middle SDI	Peptic ulcer disease	Both	243.4442557	240.9006802	245.9878312	2007
Low-middle SDI	Peptic ulcer disease	Both	234.1167935	231.6591233	236.5744637	2008
Low-middle SDI	Peptic ulcer disease	Both	217.2568343	214.9250675	219.5886011	2009
Low-middle SDI	Peptic ulcer disease	Both	206.4699999	204.2352002	208.7047995	2010
Low-middle SDI	Peptic ulcer disease	Both	197.4318301	195.2833177	199.5803425	2011
Low-middle SDI	Peptic ulcer disease	Both	187.2980429	185.2410188	189.355067	2012
Low-middle SDI	Peptic ulcer disease	Both	182.2073696	180.2020742	184.2126651	2013
Low-middle SDI	Peptic ulcer disease	Both	173.6345565	171.7000205	175.5690925	2014
Low-middle SDI	Peptic ulcer disease	Both	168.6202902	166.7465085	170.4940719	2015
Low-middle SDI	Peptic ulcer disease	Both	164.9939943	163.1713002	166.8166885	2016
Low-middle SDI	Peptic ulcer disease	Both	162.1425287	160.362538	163.9225193	2017
Low-middle SDI	Peptic ulcer disease	Both	157.3349967	155.6029776	159.0670159	2018
Low-middle SDI	Peptic ulcer disease	Both	152.4780086	150.7963599	154.1596573	2019
Low-middle SDI	Peptic ulcer disease	Both	149.8664177	133.9179824	165.8148531	2020
Low-middle SDI	Peptic ulcer disease	Both	146.4650734	129.540774	163.3893729	2021
Low-middle SDI	Peptic ulcer disease	Both	143.1478242	124.4605736	161.8350747	2022
Low-middle SDI	Peptic ulcer disease	Both	139.8996265	118.7298604	161.0693926	2023
Low-middle SDI	Peptic ulcer disease	Both	136.738944	112.4913988	160.9864892	2024
Low-middle SDI	Peptic ulcer disease	Both	133.7016045	105.90221	161.5009991	2025
Low-middle SDI	Peptic ulcer disease	Both	130.7924807	99.0749287	162.5100326	2026
Low-middle SDI	Peptic ulcer disease	Both	127.9972275	92.07580125	163.9186537	2027
Low-middle SDI	Peptic ulcer disease	Both	125.3031442	84.94723193	165.6590564	2028
Low-middle SDI	Peptic ulcer disease	Both	122.7216482	77.72828657	167.7150099	2029
Low-middle SDI	Peptic ulcer disease	Both	120.2767571	70.44988709	170.1036272	2030
Low SDI	Peptic ulcer disease	Male	392.9224803	391.8526869	393.9922736	1990
Low SDI	Peptic ulcer disease	Male	389.719952	388.6765877	390.7633162	1991
Low SDI	Peptic ulcer disease	Male	384.7484107	383.7251517	385.7716697	1992
Low SDI	Peptic ulcer disease	Male	379.4367564	378.4322832	380.4412295	1993
Low SDI	Peptic ulcer disease	Male	375.2584718	374.2711909	376.2457526	1994
Low SDI	Peptic ulcer disease	Male	366.5675416	365.6024042	367.5326789	1995
Low SDI	Peptic ulcer disease	Male	360.7905564	359.843197	361.7379157	1996
Low SDI	Peptic ulcer disease	Male	358.2890557	357.3542264	359.2238849	1997
Low SDI	Peptic ulcer disease	Male	351.9622416	351.0448619	352.8796213	1998
Low SDI	Peptic ulcer disease	Male	343.567551	342.6716517	344.4634503	1999
Low SDI	Peptic ulcer disease	Male	336.4504946	335.57181	337.3291791	2000
Low SDI	Peptic ulcer disease	Male	323.3746951	322.522489	324.2269012	2001
Low SDI	Peptic ulcer disease	Male	309.2560322	308.4322051	310.0798594	2002
Low SDI	Peptic ulcer disease	Male	298.2771519	297.4766881	299.0776156	2003
Low SDI	Peptic ulcer disease	Male	282.4856777	281.7175313	283.2538241	2004
Low SDI	Peptic ulcer disease	Male	269.2461073	268.5048839	269.9873307	2005
Low SDI	Peptic ulcer disease	Male	257.7787071	257.0637159	258.4936983	2006
Low SDI	Peptic ulcer disease	Male	254.6592277	253.9586769	255.3597785	2007
Low SDI	Peptic ulcer disease	Male	243.1709798	242.49573	243.8462296	2008
Low SDI	Peptic ulcer disease	Male	233.6757525	233.0238816	234.3276233	2009
Low SDI	Peptic ulcer disease	Male	221.7632459	221.138659	222.3878327	2010

Low SDI	Peptic ulcer disease	Male	213.0360445	212.4332513	213.6388378	2011
Low SDI	Peptic ulcer disease	Male	205.1372411	204.5550138	205.7194685	2012
Low SDI	Peptic ulcer disease	Male	201.8593167	201.289389	202.4292444	2013
Low SDI	Peptic ulcer disease	Male	192.5322888	191.984766	193.0798116	2014
Low SDI	Peptic ulcer disease	Male	188.6209606	188.0874048	189.1545163	2015
Low SDI	Peptic ulcer disease	Male	182.1124924	181.597543	182.6274417	2016
Low SDI	Peptic ulcer disease	Male	177.7635166	177.2631106	178.2639226	2017
Low SDI	Peptic ulcer disease	Male	172.6003345	172.114501	173.0861681	2018
Low SDI	Peptic ulcer disease	Male	166.954019	166.4796455	167.4283924	2019
Low SDI	Peptic ulcer disease	Male	160.5418874	155.9533588	165.1304161	2020
Low SDI	Peptic ulcer disease	Male	155.5254414	148.8209345	162.2299484	2021
Low SDI	Peptic ulcer disease	Male	150.7284896	141.4209841	160.035995	2022
Low SDI	Peptic ulcer disease	Male	146.111553	133.9164663	158.3066397	2023
Low SDI	Peptic ulcer disease	Male	141.6446863	126.3846526	156.90472	2024
Low SDI	Peptic ulcer disease	Male	137.3345579	118.8949515	155.7741644	2025
Low SDI	Peptic ulcer disease	Male	133.2042469	111.5165179	154.891976	2026
Low SDI	Peptic ulcer disease	Male	129.2562824	104.2828856	154.2296792	2027
Low SDI	Peptic ulcer disease	Male	125.4579149	97.18859099	153.7272387	2028
Low SDI	Peptic ulcer disease	Male	121.7811551	90.22763295	153.3346773	2029
Low SDI	Peptic ulcer disease	Male	118.2248209	83.41193555	153.0377063	2030
Low SDI	Peptic ulcer disease	Female	288.4971663	287.5336225	289.4607101	1990
Low SDI	Peptic ulcer disease	Female	286.295636	285.3545821	287.2366898	1991
Low SDI	Peptic ulcer disease	Female	286.3071931	285.3793005	287.2350858	1992
Low SDI	Peptic ulcer disease	Female	280.6736567	279.7658112	281.5815023	1993
Low SDI	Peptic ulcer disease	Female	275.5526786	274.6649575	276.4403997	1994
Low SDI	Peptic ulcer disease	Female	269.6106314	268.7433475	270.4779153	1995
Low SDI	Peptic ulcer disease	Female	265.6411599	264.7910255	266.4912943	1996
Low SDI	Peptic ulcer disease	Female	264.1080863	263.270613	264.9455597	1997
Low SDI	Peptic ulcer disease	Female	261.7314735	260.9075385	262.5554084	1998
Low SDI	Peptic ulcer disease	Female	256.8769556	256.0714514	257.6824599	1999
Low SDI	Peptic ulcer disease	Female	251.6587301	250.8712226	252.4462376	2000
Low SDI	Peptic ulcer disease	Female	248.3339625	247.5610941	249.1068309	2001
Low SDI	Peptic ulcer disease	Female	243.1102426	242.3545977	243.8658875	2002
Low SDI	Peptic ulcer disease	Female	235.3406654	234.6074153	236.0739156	2003
Low SDI	Peptic ulcer disease	Female	226.6888728	225.97958	227.3981656	2004
Low SDI	Peptic ulcer disease	Female	221.993495	221.3003252	222.6866647	2005
Low SDI	Peptic ulcer disease	Female	212.8192138	212.1496987	213.4887289	2006
Low SDI	Peptic ulcer disease	Female	202.4914609	201.8480307	203.134891	2007
Low SDI	Peptic ulcer disease	Female	196.3571697	195.7325954	196.9817439	2008
Low SDI	Peptic ulcer disease	Female	187.8547334	187.2555846	188.4538822	2009
Low SDI	Peptic ulcer disease	Female	179.2398842	178.6657793	179.813989	2010
Low SDI	Peptic ulcer disease	Female	171.5034248	170.9511655	172.0556842	2011
Low SDI	Peptic ulcer disease	Female	167.8621618	167.3244298	168.3998939	2012
Low SDI	Peptic ulcer disease	Female	162.6006407	162.0789718	163.1223095	2013
Low SDI	Peptic ulcer disease	Female	161.0842761	160.5731388	161.5954133	2014
Low SDI	Peptic ulcer disease	Female	156.333155	155.8380059	156.8283041	2015
Low SDI	Peptic ulcer disease	Female	152.2592305	151.7791061	152.7393548	2016
Low SDI	Peptic ulcer disease	Female	149.8716028	149.4028843	150.3403213	2017
Low SDI	Peptic ulcer disease	Female	146.4051412	145.9484222	146.8618603	2018
Low SDI	Peptic ulcer disease	Female	142.5563361	142.1102067	143.0024655	2019
Low SDI	Peptic ulcer disease	Female	138.2957041	133.7123324	142.8790758	2020
Low SDI	Peptic ulcer disease	Female	134.9703871	128.5973108	141.3434633	2021
Low SDI	Peptic ulcer disease	Female	131.7475756	123.1217892	140.3733621	2022
Low SDI	Peptic ulcer disease	Female	128.6095517	117.4381653	139.7809382	2023
Low SDI	Peptic ulcer disease	Female	125.5453459	111.6293655	139.4613263	2024
Low SDI	Peptic ulcer disease	Female	122.540403	105.7403892	139.3404168	2025
Low SDI	Peptic ulcer disease	Female	119.6076347	99.82997468	139.3852947	2026
Low SDI	Peptic ulcer disease	Female	116.7539875	93.93636652	139.5716084	2027

Low SDI	Peptic ulcer disease	Female	113.9647604	88.0701633	139.8593575	2028
Low SDI	Peptic ulcer disease	Female	111.2336864	82.24306525	140.2243075	2029
Low SDI	Peptic ulcer disease	Female	108.5511771	76.46221462	140.6401396	2030
Low SDI	Peptic ulcer disease	Both	342.0246156	336.1559143	347.8933169	1990
Low SDI	Peptic ulcer disease	Both	339.283837	333.5144161	345.0532579	1991
Low SDI	Peptic ulcer disease	Both	336.7455825	331.0683872	342.4227777	1992
Low SDI	Peptic ulcer disease	Both	331.2312086	325.6681789	336.7942383	1993
Low SDI	Peptic ulcer disease	Both	326.5047793	321.0520665	331.9574921	1994
Low SDI	Peptic ulcer disease	Both	319.0957773	313.7698909	324.4216637	1995
Low SDI	Peptic ulcer disease	Both	314.1589533	308.940959	319.3769477	1996
Low SDI	Peptic ulcer disease	Both	312.0906533	306.9542804	317.2270262	1997
Low SDI	Peptic ulcer disease	Both	307.673861	302.6319195	312.7158025	1998
Low SDI	Peptic ulcer disease	Both	300.9877412	296.0601593	305.9153231	1999
Low SDI	Peptic ulcer disease	Both	294.7860394	289.9649163	299.6071625	2000
Low SDI	Peptic ulcer disease	Both	286.5536976	281.857177	291.2502183	2001
Low SDI	Peptic ulcer disease	Both	276.846702	272.2869003	281.4065038	2002
Low SDI	Peptic ulcer disease	Both	267.3863606	262.9592459	271.8134753	2003
Low SDI	Peptic ulcer disease	Both	255.1362395	250.8661507	259.4063284	2004
Low SDI	Peptic ulcer disease	Both	246.1484259	242.006588	250.2902638	2005
Low SDI	Peptic ulcer disease	Both	235.8097911	231.8104426	239.8091396	2006
Low SDI	Peptic ulcer disease	Both	229.0460282	225.1652822	232.9267742	2007
Low SDI	Peptic ulcer disease	Both	220.1892216	216.4356816	223.9427617	2008
Low SDI	Peptic ulcer disease	Both	211.112516	207.4982471	214.7267849	2009
Low SDI	Peptic ulcer disease	Both	200.8088592	197.3426992	204.2750192	2010
Low SDI	Peptic ulcer disease	Both	192.5333926	189.1937799	195.8730053	2011
Low SDI	Peptic ulcer disease	Both	186.7578915	183.5222531	189.9935298	2012
Low SDI	Peptic ulcer disease	Both	182.4693498	179.3173154	185.6213841	2013
Low SDI	Peptic ulcer disease	Both	177.0894366	174.0307177	180.1481555	2014
Low SDI	Peptic ulcer disease	Both	172.7180788	169.7484115	175.6877461	2015
Low SDI	Peptic ulcer disease	Both	167.4271219	164.5527921	170.3014516	2016
Low SDI	Peptic ulcer disease	Both	164.069075	161.2704928	166.8676571	2017
Low SDI	Peptic ulcer disease	Both	159.7655983	157.0454541	162.4857426	2018
Low SDI	Peptic ulcer disease	Both	155.0171951	152.3785064	157.6558837	2019
Low SDI	Peptic ulcer disease	Both	149.5461923	138.0506727	161.0417118	2020
Low SDI	Peptic ulcer disease	Both	145.3958937	133.1737565	157.6180308	2021
Low SDI	Peptic ulcer disease	Both	141.4272588	127.8874059	154.9671117	2022
Low SDI	Peptic ulcer disease	Both	137.6117198	122.2352746	152.988165	2023
Low SDI	Peptic ulcer disease	Both	133.9314675	116.306257	151.556678	2024
Low SDI	Peptic ulcer disease	Both	130.3867465	110.1989404	150.5745526	2025
Low SDI	Peptic ulcer disease	Both	126.9993238	104.0187099	149.9799378	2026
Low SDI	Peptic ulcer disease	Both	123.7773209	97.83278919	149.7218526	2027
Low SDI	Peptic ulcer disease	Both	120.6971072	91.66238975	149.7318246	2028
Low SDI	Peptic ulcer disease	Both	117.7417172	85.51933296	149.9641013	2029
Low SDI	Peptic ulcer disease	Both	114.9076537	79.41701844	150.398289	2030
Global	Gastritis and duodenitis	Male	51.4148138	51.31683274	51.51279485	1990
Global	Gastritis and duodenitis	Male	50.64790512	50.55234902	50.74346123	1991
Global	Gastritis and duodenitis	Male	49.76455155	49.6710015	49.85810161	1992
Global	Gastritis and duodenitis	Male	48.8594825	48.76782814	48.95113686	1993
Global	Gastritis and duodenitis	Male	48.02655054	47.93666715	48.11643392	1994
Global	Gastritis and duodenitis	Male	47.23484013	47.14663468	47.32304559	1995
Global	Gastritis and duodenitis	Male	46.30895433	46.22254598	46.39536268	1996
Global	Gastritis and duodenitis	Male	45.31285312	45.22832912	45.39737711	1997
Global	Gastritis and duodenitis	Male	44.51256418	44.42969443	44.59543393	1998
Global	Gastritis and duodenitis	Male	43.85781028	43.77643589	43.93918467	1999
Global	Gastritis and duodenitis	Male	43.45505758	43.37489225	43.5352229	2000
Global	Gastritis and duodenitis	Male	42.95146349	42.87257821	43.03034877	2001
Global	Gastritis and duodenitis	Male	42.58369994	42.50599132	42.66140857	2002
Global	Gastritis and duodenitis	Male	42.20462906	42.1280896	42.28116851	2003

Global	Gastritis and duodenitis	Male	41.74311182	41.66782029	41.81840335	2004
Global	Gastritis and duodenitis	Male	41.12201281	41.04811946	41.19590615	2005
Global	Gastritis and duodenitis	Male	40.32635672	40.25409937	40.39861406	2006
Global	Gastritis and duodenitis	Male	39.39533132	39.32484047	39.46582218	2007
Global	Gastritis and duodenitis	Male	38.69389474	38.62488189	38.76290758	2008
Global	Gastritis and duodenitis	Male	37.99899304	37.93140588	38.06658021	2009
Global	Gastritis and duodenitis	Male	37.38178347	37.3155058	37.44806115	2010
Global	Gastritis and duodenitis	Male	36.90218515	36.83708228	36.96728802	2011
Global	Gastritis and duodenitis	Male	36.47864475	36.41465673	36.54263276	2012
Global	Gastritis and duodenitis	Male	36.14706509	36.08406676	36.21006343	2013
Global	Gastritis and duodenitis	Male	35.73032649	35.66835967	35.79229331	2014
Global	Gastritis and duodenitis	Male	35.47086458	35.40974895	35.53198022	2015
Global	Gastritis and duodenitis	Male	35.43322493	35.37276685	35.49368302	2016
Global	Gastritis and duodenitis	Male	35.26293133	35.2032357	35.32262696	2017
Global	Gastritis and duodenitis	Male	34.62537175	34.56678355	34.68395995	2018
Global	Gastritis and duodenitis	Male	34.07793065	34.02011392	34.13574737	2019
Global	Gastritis and duodenitis	Male	33.78764083	33.00803539	34.56724628	2020
Global	Gastritis and duodenitis	Male	33.39376044	32.28162961	34.50589128	2021
Global	Gastritis and duodenitis	Male	33.00880018	31.47416645	34.5434339	2022
Global	Gastritis and duodenitis	Male	32.63152184	30.61171846	34.65132522	2023
Global	Gastritis and duodenitis	Male	32.26278844	29.70951085	34.81606602	2024
Global	Gastritis and duodenitis	Male	31.90443565	28.77777962	35.03109168	2025
Global	Gastritis and duodenitis	Male	31.55663383	27.82425303	35.28901463	2026
Global	Gastritis and duodenitis	Male	31.21808531	26.85273614	35.58343449	2027
Global	Gastritis and duodenitis	Male	30.88715265	25.86500444	35.90930087	2028
Global	Gastritis and duodenitis	Male	30.5637139	24.86297001	36.26445779	2029
Global	Gastritis and duodenitis	Male	30.24873613	23.84902203	36.64845023	2030
Global	Gastritis and duodenitis	Female	45.19593051	45.10940275	45.28245827	1990
Global	Gastritis and duodenitis	Female	44.38366976	44.29902191	44.46831761	1991
Global	Gastritis and duodenitis	Female	43.66336887	43.58029819	43.74643954	1992
Global	Gastritis and duodenitis	Female	42.89276712	42.81123871	42.97429552	1993
Global	Gastritis and duodenitis	Female	42.32511258	42.24492867	42.40529649	1994
Global	Gastritis and duodenitis	Female	41.7265126	41.64767127	41.80535393	1995
Global	Gastritis and duodenitis	Female	41.15360685	41.07608205	41.23113165	1996
Global	Gastritis and duodenitis	Female	40.72529078	40.64895105	40.8016305	1997
Global	Gastritis and duodenitis	Female	40.27097928	40.19582799	40.34613057	1998
Global	Gastritis and duodenitis	Female	39.93514868	39.86104771	40.00924965	1999
Global	Gastritis and duodenitis	Female	39.75101927	39.67780312	39.82423542	2000
Global	Gastritis and duodenitis	Female	39.48371852	39.41147572	39.55596131	2001
Global	Gastritis and duodenitis	Female	39.33538517	39.26399176	39.40677857	2002
Global	Gastritis and duodenitis	Female	39.20961746	39.13904272	39.2801922	2003
Global	Gastritis and duodenitis	Female	38.98151025	38.91185949	39.051161	2004
Global	Gastritis and duodenitis	Female	38.66826038	38.5996012	38.73691955	2005
Global	Gastritis and duodenitis	Female	38.23607296	38.16853126	38.30361465	2006
Global	Gastritis and duodenitis	Female	37.72652573	37.66017958	37.79287188	2007
Global	Gastritis and duodenitis	Female	37.40400332	37.33865247	37.46935417	2008
Global	Gastritis and duodenitis	Female	37.10698393	37.04256973	37.17139814	2009
Global	Gastritis and duodenitis	Female	36.84422239	36.78067602	36.90776877	2010
Global	Gastritis and duodenitis	Female	36.5545956	36.49192289	36.61726832	2011
Global	Gastritis and duodenitis	Female	36.20659319	36.14483856	36.26834782	2012
Global	Gastritis and duodenitis	Female	35.98275707	35.92179194	36.0437222	2013
Global	Gastritis and duodenitis	Female	35.77008064	35.70987315	35.83028813	2014
Global	Gastritis and duodenitis	Female	35.67403166	35.61445468	35.73360864	2015
Global	Gastritis and duodenitis	Female	35.84805529	35.7888749	35.90723568	2016
Global	Gastritis and duodenitis	Female	36.04397977	35.98516972	36.10278983	2017
Global	Gastritis and duodenitis	Female	35.83397568	35.77582779	35.89212356	2018
Global	Gastritis and duodenitis	Female	35.53740423	35.47988099	35.59492747	2019
Global	Gastritis and duodenitis	Female	35.61932147	34.61609696	36.62254598	2020

Global	Gastritis and duodenitis	Female	35.56142682	34.20624317	36.91661048	2021
Global	Gastritis and duodenitis	Female	35.49923851	33.68701336	37.31146366	2022
Global	Gastritis and duodenitis	Female	35.43360491	33.0847974	37.78241243	2023
Global	Gastritis and duodenitis	Female	35.36730773	32.41693504	38.31768043	2024
Global	Gastritis and duodenitis	Female	35.30140246	31.69346087	38.90934405	2025
Global	Gastritis and duodenitis	Female	35.23202961	30.91890001	39.5451592	2026
Global	Gastritis and duodenitis	Female	35.15764243	30.09731795	40.21796691	2027
Global	Gastritis and duodenitis	Female	35.07856834	29.23258141	40.92455527	2028
Global	Gastritis and duodenitis	Female	34.9970637	28.32889599	41.66523141	2029
Global	Gastritis and duodenitis	Female	34.91420561	27.38903935	42.43937187	2030
Global	Gastritis and duodenitis	Both	48.15083909	47.60359704	48.69808114	1990
Global	Gastritis and duodenitis	Both	47.36518841	46.82848875	47.90188807	1991
Global	Gastritis and duodenitis	Both	46.57182564	46.04529838	47.0983529	1992
Global	Gastritis and duodenitis	Both	45.74470602	45.22816457	46.26124748	1993
Global	Gastritis and duodenitis	Both	45.04896788	44.54148489	45.55645086	1994
Global	Gastritis and duodenitis	Both	44.35922638	43.8604941	44.85795866	1995
Global	Gastritis and duodenitis	Both	43.61640075	43.12678416	44.10601733	1996
Global	Gastritis and duodenitis	Both	42.91668335	42.4357888	43.39757789	1997
Global	Gastritis and duodenitis	Both	42.2968056	41.82401831	42.7695929	1998
Global	Gastritis and duodenitis	Both	41.8127695	41.34718527	42.27835373	1999
Global	Gastritis and duodenitis	Both	41.52466749	41.06511402	41.98422096	2000
Global	Gastritis and duodenitis	Both	41.1400407	40.68697266	41.59310875	2001
Global	Gastritis and duodenitis	Both	40.88915403	40.44187667	41.33643139	2002
Global	Gastritis and duodenitis	Both	40.64449629	40.20283734	41.08615524	2003
Global	Gastritis and duodenitis	Both	40.30405587	39.86851427	40.73959746	2004
Global	Gastritis and duodenitis	Both	39.8392453	39.4105313	40.2679593	2005
Global	Gastritis and duodenitis	Both	39.22966509	38.80875015	39.65058004	2006
Global	Gastritis and duodenitis	Both	38.51048096	38.0978787	38.92308321	2007
Global	Gastritis and duodenitis	Both	37.9949328	37.58925145	38.40061414	2008
Global	Gastritis and duodenitis	Both	37.49649153	37.09749596	37.8954871	2009
Global	Gastritis and duodenitis	Both	37.0559787	36.66335979	37.44859762	2010
Global	Gastritis and duodenitis	Both	36.67346562	36.28695222	37.05997902	2011
Global	Gastritis and duodenitis	Both	36.28881545	35.90831013	36.66932077	2012
Global	Gastritis and duodenitis	Both	36.01445085	35.63910767	36.38979402	2013
Global	Gastritis and duodenitis	Both	35.7053113	35.33514375	36.07547885	2014
Global	Gastritis and duodenitis	Both	35.53094254	35.16518133	35.89670375	2015
Global	Gastritis and duodenitis	Both	35.60462615	35.24201507	35.96723723	2016
Global	Gastritis and duodenitis	Both	35.62685271	35.26756269	35.98614273	2017
Global	Gastritis and duodenitis	Both	35.21452409	34.860511	35.56853718	2018
Global	Gastritis and duodenitis	Both	34.79880685	34.44984836	35.14776533	2019
Global	Gastritis and duodenitis	Both	34.72680017	32.32902853	37.12457181	2020
Global	Gastritis and duodenitis	Both	34.50969966	31.96107796	37.05832136	2021
Global	Gastritis and duodenitis	Both	34.29704154	31.50205831	37.09202476	2022
Global	Gastritis and duodenitis	Both	34.08924288	30.95195641	37.22652935	2023
Global	Gastritis and duodenitis	Both	33.8885611	30.32010547	37.45701674	2024
Global	Gastritis and duodenitis	Both	33.69714787	29.6188218	37.77547395	2025
Global	Gastritis and duodenitis	Both	33.51371339	28.85912541	38.16830136	2026
Global	Gastritis and duodenitis	Both	33.33745041	28.04993282	38.624968	2027
Global	Gastritis and duodenitis	Both	33.16813159	27.19786692	39.13839626	2028
Global	Gastritis and duodenitis	Both	33.00729446	26.30867864	39.70591027	2029
Global	Gastritis and duodenitis	Both	32.8566396	25.38686539	40.32641381	2030
High SDI	Gastritis and duodenitis	Male	19.90966813	19.78177473	20.03756153	1990
High SDI	Gastritis and duodenitis	Male	19.4902045	19.37034987	19.61005912	1991
High SDI	Gastritis and duodenitis	Male	19.04059881	18.92388871	19.1573089	1992
High SDI	Gastritis and duodenitis	Male	18.67970067	18.56506731	18.79433404	1993
High SDI	Gastritis and duodenitis	Male	18.3667205	18.25394782	18.47949318	1994
High SDI	Gastritis and duodenitis	Male	18.05660366	17.94566554	18.16754179	1995
High SDI	Gastritis and duodenitis	Male	17.67812105	17.56925051	17.7869916	1996

High SDI	Gastritis and duodenitis	Male	17.34526799	17.23836241	17.45217358	1997
High SDI	Gastritis and duodenitis	Male	17.03730789	16.93217612	17.14243965	1998
High SDI	Gastritis and duodenitis	Male	16.72876616	16.62531649	16.83221582	1999
High SDI	Gastritis and duodenitis	Male	16.50151523	16.39947816	16.60355229	2000
High SDI	Gastritis and duodenitis	Male	16.22394656	16.12347771	16.32441542	2001
High SDI	Gastritis and duodenitis	Male	15.98215311	15.88315308	16.08115314	2002
High SDI	Gastritis and duodenitis	Male	15.70765734	15.61020558	15.80510911	2003
High SDI	Gastritis and duodenitis	Male	15.37582541	15.28010314	15.47154767	2004
High SDI	Gastritis and duodenitis	Male	15.12585169	15.03155386	15.22014953	2005
High SDI	Gastritis and duodenitis	Male	14.8801888	14.78730165	14.97307594	2006
High SDI	Gastritis and duodenitis	Male	14.61616639	14.52480832	14.70752447	2007
High SDI	Gastritis and duodenitis	Male	14.3748	14.28488888	14.46471112	2008
High SDI	Gastritis and duodenitis	Male	14.18109892	14.09240085	14.26979698	2009
High SDI	Gastritis and duodenitis	Male	14.04530462	13.95754195	14.13306729	2010
High SDI	Gastritis and duodenitis	Male	13.95944202	13.87238975	14.04649429	2011
High SDI	Gastritis and duodenitis	Male	13.90295932	13.8165118	13.98940685	2012
High SDI	Gastritis and duodenitis	Male	13.88799523	13.80197963	13.97401083	2013
High SDI	Gastritis and duodenitis	Male	13.85472518	13.76914113	13.94030922	2014
High SDI	Gastritis and duodenitis	Male	13.85506939	13.76977798	13.94036081	2015
High SDI	Gastritis and duodenitis	Male	13.88788824	13.80277906	13.97299743	2016
High SDI	Gastritis and duodenitis	Male	13.90254613	13.81766106	13.98743121	2017
High SDI	Gastritis and duodenitis	Male	13.91159608	13.82642152	13.99677064	2018
High SDI	Gastritis and duodenitis	Male	13.92305232	13.834105	14.01199964	2019
High SDI	Gastritis and duodenitis	Male	14.0225252	13.67902733	14.36602307	2020
High SDI	Gastritis and duodenitis	Male	14.05169257	13.5614756	14.54190955	2021
High SDI	Gastritis and duodenitis	Male	14.0806807	13.40309838	14.75826301	2022
High SDI	Gastritis and duodenitis	Male	14.11012259	13.21386665	15.00637853	2023
High SDI	Gastritis and duodenitis	Male	14.14131448	12.99984261	15.28278635	2024
High SDI	Gastritis and duodenitis	Male	14.17482797	12.76432132	15.58533461	2025
High SDI	Gastritis and duodenitis	Male	14.20880211	12.50799966	15.90960455	2026
High SDI	Gastritis and duodenitis	Male	14.24244429	12.23176468	16.2531239	2027
High SDI	Gastritis and duodenitis	Male	14.2763544	11.93714772	16.61556109	2028
High SDI	Gastritis and duodenitis	Male	14.31185871	11.62582401	16.99789341	2029
High SDI	Gastritis and duodenitis	Male	14.3495644	11.29864743	17.40048136	2030
High SDI	Gastritis and duodenitis	Female	24.32439993	24.18665831	24.46214154	1990
High SDI	Gastritis and duodenitis	Female	23.64273655	23.5098979	23.7755752	1991
High SDI	Gastritis and duodenitis	Female	23.00830926	22.87844114	23.13817737	1992
High SDI	Gastritis and duodenitis	Female	22.5021072	22.37445114	22.62976325	1993
High SDI	Gastritis and duodenitis	Female	22.0996793	21.97386966	22.22548895	1994
High SDI	Gastritis and duodenitis	Female	21.84343593	21.71900922	21.96786264	1995
High SDI	Gastritis and duodenitis	Female	21.62824476	21.50504879	21.75144073	1996
High SDI	Gastritis and duodenitis	Female	21.4106669	21.28878202	21.53255178	1997
High SDI	Gastritis and duodenitis	Female	21.2140189	21.09334857	21.33468924	1998
High SDI	Gastritis and duodenitis	Female	21.01287684	20.89337151	21.13238218	1999
High SDI	Gastritis and duodenitis	Female	20.76031814	20.64200772	20.87862856	2000
High SDI	Gastritis and duodenitis	Female	20.47291035	20.35585042	20.58997029	2001
High SDI	Gastritis and duodenitis	Female	20.13290652	20.01728838	20.24852467	2002
High SDI	Gastritis and duodenitis	Female	19.79283846	19.67862967	19.90704724	2003
High SDI	Gastritis and duodenitis	Female	19.43376188	19.32100373	19.54652003	2004
High SDI	Gastritis and duodenitis	Female	19.18216775	19.07056221	19.29377329	2005
High SDI	Gastritis and duodenitis	Female	18.96201228	18.85145808	19.07256647	2006
High SDI	Gastritis and duodenitis	Female	18.7599997	18.65045869	18.86954071	2007
High SDI	Gastritis and duodenitis	Female	18.59487358	18.48622841	18.70351874	2008
High SDI	Gastritis and duodenitis	Female	18.46436282	18.35645802	18.57226762	2009
High SDI	Gastritis and duodenitis	Female	18.39630686	18.28892652	18.5036872	2010
High SDI	Gastritis and duodenitis	Female	18.40457754	18.2974833	18.51167179	2011
High SDI	Gastritis and duodenitis	Female	18.43630461	18.32943534	18.54317389	2012
High SDI	Gastritis and duodenitis	Female	18.47582732	18.36911338	18.58254126	2013

High SDI	Gastritis and duodenitis	Female	18.52010668	18.41350821	18.62670516	2014
High SDI	Gastritis and duodenitis	Female	18.59035485	18.48379626	18.69691345	2015
High SDI	Gastritis and duodenitis	Female	18.68277075	18.57618106	18.78936044	2016
High SDI	Gastritis and duodenitis	Female	18.76553268	18.65889299	18.87217236	2017
High SDI	Gastritis and duodenitis	Female	18.8536936	18.74664216	18.96074503	2018
High SDI	Gastritis and duodenitis	Female	18.96834278	18.85924309	19.07744246	2019
High SDI	Gastritis and duodenitis	Female	19.03222291	18.43088544	19.63356039	2020
High SDI	Gastritis and duodenitis	Female	19.13089975	18.31510138	19.94669813	2021
High SDI	Gastritis and duodenitis	Female	19.22898427	18.13326703	20.32470151	2022
High SDI	Gastritis and duodenitis	Female	19.32710551	17.89987219	20.75433883	2023
High SDI	Gastritis and duodenitis	Female	19.42616656	17.6237783	21.22855483	2024
High SDI	Gastritis and duodenitis	Female	19.52623149	17.30981665	21.74264633	2025
High SDI	Gastritis and duodenitis	Female	19.62555566	16.96003914	22.29107218	2026
High SDI	Gastritis and duodenitis	Female	19.72351568	16.57630578	22.87072559	2027
High SDI	Gastritis and duodenitis	Female	19.82064365	16.16065357	23.48063374	2028
High SDI	Gastritis and duodenitis	Female	19.9178176	15.71481729	24.12081791	2029
High SDI	Gastritis and duodenitis	Female	20.01523504	15.23971404	24.79075604	2030
High SDI	Gastritis and duodenitis	Both	22.1288059	21.34379757	22.91381424	1990
High SDI	Gastritis and duodenitis	Both	21.57654652	20.80667226	22.34642078	1991
High SDI	Gastritis and duodenitis	Both	21.03706477	20.28157119	21.79255836	1992
High SDI	Gastritis and duodenitis	Both	20.60611293	19.86272897	21.34949689	1993
High SDI	Gastritis and duodenitis	Both	20.25216461	19.5193467	20.98498252	1994
High SDI	Gastritis and duodenitis	Both	19.97689461	19.25339481	20.70039441	1995
High SDI	Gastritis and duodenitis	Both	19.68841497	18.97491051	20.40191942	1996
High SDI	Gastritis and duodenitis	Both	19.41878794	18.71574289	20.12183299	1997
High SDI	Gastritis and duodenitis	Both	19.16881509	18.47580123	19.86182894	1998
High SDI	Gastritis and duodenitis	Both	18.91552088	18.23219123	19.59885053	1999
High SDI	Gastritis and duodenitis	Both	18.66898133	17.99489951	19.34306314	2000
High SDI	Gastritis and duodenitis	Both	18.38025291	17.71541832	19.04508749	2001
High SDI	Gastritis and duodenitis	Both	18.08265278	17.42706743	18.73823814	2002
High SDI	Gastritis and duodenitis	Both	17.76896471	17.12265581	18.41527361	2003
High SDI	Gastritis and duodenitis	Both	17.4185683	16.7820881	18.0550485	2004
High SDI	Gastritis and duodenitis	Both	17.16531286	16.53686558	17.79376015	2005
High SDI	Gastritis and duodenitis	Both	16.92862284	16.30784091	17.54940476	2006
High SDI	Gastritis and duodenitis	Both	16.69069948	16.07772354	17.30367541	2007
High SDI	Gastritis and duodenitis	Both	16.48190625	15.87606206	17.08775044	2008
High SDI	Gastritis and duodenitis	Both	16.3150916	15.7154168	16.9147664	2009
High SDI	Gastritis and duodenitis	Both	16.21061587	15.61559614	16.80563561	2010
High SDI	Gastritis and duodenitis	Both	16.17089993	15.57920948	16.76259038	2011
High SDI	Gastritis and duodenitis	Both	16.15744617	15.56858133	16.74631101	2012
High SDI	Gastritis and duodenitis	Both	16.16743362	15.58087662	16.75399062	2013
High SDI	Gastritis and duodenitis	Both	16.17098512	15.58674659	16.75522364	2014
High SDI	Gastritis and duodenitis	Both	16.2048837	15.62228784	16.78747957	2015
High SDI	Gastritis and duodenitis	Both	16.2649554	15.68355653	16.84635428	2016
High SDI	Gastritis and duodenitis	Both	16.31108195	15.73093521	16.89122869	2017
High SDI	Gastritis and duodenitis	Both	16.35779208	15.77810993	16.93747424	2018
High SDI	Gastritis and duodenitis	Both	16.41978743	15.83921136	17.0003635	2019
High SDI	Gastritis and duodenitis	Both	16.53109362	15.22763901	17.83454822	2020
High SDI	Gastritis and duodenitis	Both	16.5961439	15.20857879	17.98370902	2021
High SDI	Gastritis and duodenitis	Both	16.66203878	15.14124823	18.18282933	2022
High SDI	Gastritis and duodenitis	Both	16.7297786	15.02391768	18.43563952	2023
High SDI	Gastritis and duodenitis	Both	16.80085463	14.85949083	18.74221842	2024
High SDI	Gastritis and duodenitis	Both	16.87597723	14.65220607	19.09974839	2025
High SDI	Gastritis and duodenitis	Both	16.95369989	14.40534971	19.50205006	2026
High SDI	Gastritis and duodenitis	Both	17.03369118	14.12247121	19.94491114	2027
High SDI	Gastritis and duodenitis	Both	17.11692885	13.80720287	20.42665484	2028
High SDI	Gastritis and duodenitis	Both	17.20495785	13.4625878	20.9473279	2029
High SDI	Gastritis and duodenitis	Both	17.29862825	13.09034951	21.50690698	2030

High-middle SDI	Gastritis and duodenitis	Male	42.53894672	42.35891258	42.71898086	1990
High-middle SDI	Gastritis and duodenitis	Male	41.81900744	41.64812516	41.98988971	1991
High-middle SDI	Gastritis and duodenitis	Male	41.18509268	41.0176537	41.35253166	1992
High-middle SDI	Gastritis and duodenitis	Male	40.56243604	40.39817209	40.72669999	1993
High-middle SDI	Gastritis and duodenitis	Male	39.96754767	39.80608004	40.12901529	1994
High-middle SDI	Gastritis and duodenitis	Male	39.27535159	39.11662593	39.43407726	1995
High-middle SDI	Gastritis and duodenitis	Male	38.27292481	38.11769424	38.42815537	1996
High-middle SDI	Gastritis and duodenitis	Male	37.18042974	37.02890937	37.3319501	1997
High-middle SDI	Gastritis and duodenitis	Male	36.37480566	36.226227	36.52338431	1998
High-middle SDI	Gastritis and duodenitis	Male	35.95751408	35.81100448	36.10402369	1999
High-middle SDI	Gastritis and duodenitis	Male	35.75950307	35.61451578	35.90449035	2000
High-middle SDI	Gastritis and duodenitis	Male	35.40455725	35.26153602	35.54757848	2001
High-middle SDI	Gastritis and duodenitis	Male	35.16071776	35.01947285	35.30196267	2002
High-middle SDI	Gastritis and duodenitis	Male	34.91860838	34.77900715	35.05820961	2003
High-middle SDI	Gastritis and duodenitis	Male	34.55374871	34.41601718	34.69148023	2004
High-middle SDI	Gastritis and duodenitis	Male	33.956159	33.82079414	34.09152387	2005
High-middle SDI	Gastritis and duodenitis	Male	32.72657025	32.59519224	32.85794825	2006
High-middle SDI	Gastritis and duodenitis	Male	31.33330048	31.20634181	31.46025916	2007
High-middle SDI	Gastritis and duodenitis	Male	30.1956972	30.07247125	30.31892315	2008
High-middle SDI	Gastritis and duodenitis	Male	29.12498368	29.00524234	29.24472502	2009
High-middle SDI	Gastritis and duodenitis	Male	28.48712133	28.36985993	28.60438274	2010
High-middle SDI	Gastritis and duodenitis	Male	27.94971379	27.83474531	28.06468227	2011
High-middle SDI	Gastritis and duodenitis	Male	27.53169488	27.4187689	27.64462086	2012
High-middle SDI	Gastritis and duodenitis	Male	27.16505874	27.05397449	27.27614299	2013
High-middle SDI	Gastritis and duodenitis	Male	26.83175179	26.72237694	26.94112664	2014
High-middle SDI	Gastritis and duodenitis	Male	26.60743459	26.49941324	26.71545594	2015
High-middle SDI	Gastritis and duodenitis	Male	26.45127334	26.34442804	26.55811864	2016
High-middle SDI	Gastritis and duodenitis	Male	26.19569487	26.09015801	26.30123172	2017
High-middle SDI	Gastritis and duodenitis	Male	25.80617442	25.7021733	25.91017554	2018
High-middle SDI	Gastritis and duodenitis	Male	25.50437209	25.39793119	25.61081299	2019
High-middle SDI	Gastritis and duodenitis	Male	25.41919856	24.78272981	26.05566731	2020
High-middle SDI	Gastritis and duodenitis	Male	25.15841602	24.0983309	26.21850115	2021
High-middle SDI	Gastritis and duodenitis	Male	24.90221148	23.32073907	26.48368388	2022
High-middle SDI	Gastritis and duodenitis	Male	24.65318539	22.48024456	26.82612623	2023
High-middle SDI	Gastritis and duodenitis	Male	24.41100598	21.58936995	27.23264202	2024
High-middle SDI	Gastritis and duodenitis	Male	24.17388001	20.65374843	27.69401159	2025
High-middle SDI	Gastritis and duodenitis	Male	23.93838018	19.67880238	28.19795798	2026
High-middle SDI	Gastritis and duodenitis	Male	23.70657171	18.67232415	28.74081926	2027
High-middle SDI	Gastritis and duodenitis	Male	23.48094984	17.64040943	29.32149026	2028
High-middle SDI	Gastritis and duodenitis	Male	23.26118828	16.58518754	29.93718901	2029
High-middle SDI	Gastritis and duodenitis	Male	23.04556379	15.50685983	30.58426775	2030
High-middle SDI	Gastritis and duodenitis	Female	37.87775244	37.72168432	38.03382057	1990
High-middle SDI	Gastritis and duodenitis	Female	37.22800014	37.07726304	37.37873724	1991
High-middle SDI	Gastritis and duodenitis	Female	36.58693757	36.43881952	36.73505561	1992
High-middle SDI	Gastritis and duodenitis	Female	36.08483031	35.93884209	36.23081853	1993
High-middle SDI	Gastritis and duodenitis	Female	35.65425352	35.51014352	35.79836352	1994
High-middle SDI	Gastritis and duodenitis	Female	35.18133049	35.0390717	35.32358928	1995
High-middle SDI	Gastritis and duodenitis	Female	34.68004261	34.53977581	34.82030941	1996
High-middle SDI	Gastritis and duodenitis	Female	34.22149028	34.08319398	34.35978657	1997
High-middle SDI	Gastritis and duodenitis	Female	33.79070456	33.65426267	33.92714644	1998
High-middle SDI	Gastritis and duodenitis	Female	33.55470284	33.41967092	33.68973476	1999
High-middle SDI	Gastritis and duodenitis	Female	33.40362542	33.269734	33.53751684	2000
High-middle SDI	Gastritis and duodenitis	Female	33.23964394	33.10698651	33.37230136	2001
High-middle SDI	Gastritis and duodenitis	Female	33.20351593	33.07191512	33.33511674	2002
High-middle SDI	Gastritis and duodenitis	Female	33.17463032	33.04403324	33.30522739	2003
High-middle SDI	Gastritis and duodenitis	Female	33.06117892	32.93172242	33.19063542	2004
High-middle SDI	Gastritis and duodenitis	Female	32.74113452	32.61317204	32.86909699	2005
High-middle SDI	Gastritis and duodenitis	Female	32.15755605	32.03169184	32.28342026	2006

High-middle SDI	Gastritis and duodenitis	Female	31.40043944	31.27708541	31.52379348	2007
High-middle SDI	Gastritis and duodenitis	Female	30.66706598	30.54612398	30.78800799	2008
High-middle SDI	Gastritis and duodenitis	Female	30.069518	29.95062711	30.18840889	2009
High-middle SDI	Gastritis and duodenitis	Female	29.64486368	29.5275899	29.76213746	2010
High-middle SDI	Gastritis and duodenitis	Female	29.30752167	29.19167286	29.42337048	2011
High-middle SDI	Gastritis and duodenitis	Female	28.98283669	28.86841186	29.09726152	2012
High-middle SDI	Gastritis and duodenitis	Female	28.74119423	28.62798255	28.8544059	2013
High-middle SDI	Gastritis and duodenitis	Female	28.58949585	28.47723314	28.70175857	2014
High-middle SDI	Gastritis and duodenitis	Female	28.53656055	28.42496603	28.64815507	2015
High-middle SDI	Gastritis and duodenitis	Female	28.73791971	28.62646537	28.84937405	2016
High-middle SDI	Gastritis and duodenitis	Female	28.95297942	28.84160911	29.06434973	2017
High-middle SDI	Gastritis and duodenitis	Female	28.88941681	28.77851693	29.00031668	2018
High-middle SDI	Gastritis and duodenitis	Female	28.77390491	28.6614928	28.88631701	2019
High-middle SDI	Gastritis and duodenitis	Female	28.89813691	28.10384694	29.69242688	2020
High-middle SDI	Gastritis and duodenitis	Female	28.93236068	27.70642544	30.15829593	2021
High-middle SDI	Gastritis and duodenitis	Female	28.95990787	27.18546765	30.73434809	2022
High-middle SDI	Gastritis and duodenitis	Female	28.98102342	26.57030902	31.39173782	2023
High-middle SDI	Gastritis and duodenitis	Female	29.00153747	25.87942255	32.12365238	2024
High-middle SDI	Gastritis and duodenitis	Female	29.02379175	25.1216124	32.92597111	2025
High-middle SDI	Gastritis and duodenitis	Female	29.04421865	24.30229647	33.78614084	2026
High-middle SDI	Gastritis and duodenitis	Female	29.05755521	23.42282472	34.69228571	2027
High-middle SDI	Gastritis and duodenitis	Female	29.06339644	22.4867167	35.64007618	2028
High-middle SDI	Gastritis and duodenitis	Female	29.06699592	21.49995306	36.63403878	2029
High-middle SDI	Gastritis and duodenitis	Female	29.07090708	20.46522012	37.67659404	2030
High-middle SDI	Gastritis and duodenitis	Both	39.65389802	38.6799872	40.62780884	1990
High-middle SDI	Gastritis and duodenitis	Both	38.9905587	38.03512176	39.94599564	1991
High-middle SDI	Gastritis and duodenitis	Both	38.37316724	37.43419199	39.31214248	1992
High-middle SDI	Gastritis and duodenitis	Both	37.83921775	36.91462198	38.76381352	1993
High-middle SDI	Gastritis and duodenitis	Both	37.34455019	36.43326877	38.25583161	1994
High-middle SDI	Gastritis and duodenitis	Both	36.77729547	35.87911827	37.67547267	1995
High-middle SDI	Gastritis and duodenitis	Both	36.04954018	35.16692861	36.93215175	1996
High-middle SDI	Gastritis and duodenitis	Both	35.30350584	34.43614427	36.17086742	1997
High-middle SDI	Gastritis and duodenitis	Both	34.70337086	33.84911846	35.55762326	1998
High-middle SDI	Gastritis and duodenitis	Both	34.39705105	33.5522049	35.24189721	1999
High-middle SDI	Gastritis and duodenitis	Both	34.23581595	33.39815885	35.07347304	2000
High-middle SDI	Gastritis and duodenitis	Both	33.98865437	33.1595606	34.81774814	2001
High-middle SDI	Gastritis and duodenitis	Both	33.8660616	33.04413295	34.68799025	2002
High-middle SDI	Gastritis and duodenitis	Both	33.74840226	32.93309856	34.56370597	2003
High-middle SDI	Gastritis and duodenitis	Both	33.52428941	32.7170285	34.33155031	2004
High-middle SDI	Gastritis and duodenitis	Both	33.07992679	32.28356271	33.87629086	2005
High-middle SDI	Gastritis and duodenitis	Both	32.19988967	31.420666	32.97911333	2006
High-middle SDI	Gastritis and duodenitis	Both	31.14836975	30.38870593	31.90803358	2007
High-middle SDI	Gastritis and duodenitis	Both	30.2259425	29.48397764	30.96790737	2008
High-middle SDI	Gastritis and duodenitis	Both	29.40311352	28.67716803	30.129059	2009
High-middle SDI	Gastritis and duodenitis	Both	28.87694458	28.16302897	29.59086019	2010
High-middle SDI	Gastritis and duodenitis	Both	28.44494542	27.74212806	29.14776279	2011
High-middle SDI	Gastritis and duodenitis	Both	28.07498074	27.38246754	28.76749395	2012
High-middle SDI	Gastritis and duodenitis	Both	27.77712629	27.09340676	28.46084581	2013
High-middle SDI	Gastritis and duodenitis	Both	27.5445862	26.86847975	28.22069264	2014
High-middle SDI	Gastritis and duodenitis	Both	27.41254223	26.74238008	28.08270439	2015
High-middle SDI	Gastritis and duodenitis	Both	27.44365065	26.77772371	28.10957759	2016
High-middle SDI	Gastritis and duodenitis	Both	27.43780354	26.77610187	28.09950522	2017
High-middle SDI	Gastritis and duodenitis	Both	27.2265456	26.57063731	27.8824539	2018
High-middle SDI	Gastritis and duodenitis	Both	27.02710405	26.37552494	27.67868316	2019
High-middle SDI	Gastritis and duodenitis	Both	27.08210216	25.36884147	28.79536285	2020
High-middle SDI	Gastritis and duodenitis	Both	26.98715092	25.04781314	28.92648869	2021
High-middle SDI	Gastritis and duodenitis	Both	26.89369568	24.58811552	29.19927584	2022
High-middle SDI	Gastritis and duodenitis	Both	26.80443288	24.00839823	29.60046753	2023

High-middle SDI	Gastritis and duodenitis	Both	26.72366697	23.33243686	30.11489709	2024
High-middle SDI	Gastritis and duodenitis	Both	26.65298685	22.57713165	30.72884204	2025
High-middle SDI	Gastritis and duodenitis	Both	26.58938418	21.75460974	31.42415862	2026
High-middle SDI	Gastritis and duodenitis	Both	26.53225815	20.87390562	32.19061067	2027
High-middle SDI	Gastritis and duodenitis	Both	26.4839188	19.94225153	33.02558607	2028
High-middle SDI	Gastritis and duodenitis	Both	26.44823725	18.96444275	33.93203176	2029
High-middle SDI	Gastritis and duodenitis	Both	26.42696515	17.94127494	34.91265535	2030
Middle SDI	Gastritis and duodenitis	Male	65.44612606	65.2260838	65.66616831	1990
Middle SDI	Gastritis and duodenitis	Male	63.91573739	63.70314274	64.12833205	1991
Middle SDI	Gastritis and duodenitis	Male	62.15127935	61.94487851	62.3576802	1992
Middle SDI	Gastritis and duodenitis	Male	60.38820994	60.18758509	60.5888348	1993
Middle SDI	Gastritis and duodenitis	Male	58.84346567	58.64799853	59.03893281	1994
Middle SDI	Gastritis and duodenitis	Male	57.56951126	57.37861482	57.7604077	1995
Middle SDI	Gastritis and duodenitis	Male	56.07024709	55.88418287	56.2563113	1996
Middle SDI	Gastritis and duodenitis	Male	54.24350397	54.06293358	54.42407435	1997
Middle SDI	Gastritis and duodenitis	Male	52.86682859	52.69096698	53.04269019	1998
Middle SDI	Gastritis and duodenitis	Male	51.80832676	51.63646707	51.98018645	1999
Middle SDI	Gastritis and duodenitis	Male	51.38888829	51.2197972	51.55797937	2000
Middle SDI	Gastritis and duodenitis	Male	50.95099554	50.78462372	51.11736736	2001
Middle SDI	Gastritis and duodenitis	Male	50.77898649	50.61505635	50.94291663	2002
Middle SDI	Gastritis and duodenitis	Male	50.59422939	50.43282606	50.75563272	2003
Middle SDI	Gastritis and duodenitis	Male	50.30545416	50.146535	50.46437333	2004
Middle SDI	Gastritis and duodenitis	Male	49.48855232	49.33295333	49.64415131	2005
Middle SDI	Gastritis and duodenitis	Male	48.23904699	48.08786044	48.39023353	2006
Middle SDI	Gastritis and duodenitis	Male	46.67532272	46.52899103	46.8216544	2007
Middle SDI	Gastritis and duodenitis	Male	45.53707661	45.39464904	45.67950418	2008
Middle SDI	Gastritis and duodenitis	Male	44.49725516	44.35844596	44.63606436	2009
Middle SDI	Gastritis and duodenitis	Male	43.56336667	43.42789402	43.69883931	2010
Middle SDI	Gastritis and duodenitis	Male	42.75293756	42.6207032	42.88517192	2011
Middle SDI	Gastritis and duodenitis	Male	42.05819753	41.92895012	42.18744494	2012
Middle SDI	Gastritis and duodenitis	Male	41.39003389	41.26361837	41.51644941	2013
Middle SDI	Gastritis and duodenitis	Male	40.55242935	40.42906591	40.67579278	2014
Middle SDI	Gastritis and duodenitis	Male	39.89669579	39.77592858	40.01746299	2015
Middle SDI	Gastritis and duodenitis	Male	39.38669246	39.26834886	39.50503605	2016
Middle SDI	Gastritis and duodenitis	Male	38.69729383	38.58162472	38.81296293	2017
Middle SDI	Gastritis and duodenitis	Male	37.4479754	37.33568412	37.56026668	2018
Middle SDI	Gastritis and duodenitis	Male	36.4640134	36.35387757	36.57414924	2019
Middle SDI	Gastritis and duodenitis	Male	36.3242074	35.11297827	37.53543654	2020
Middle SDI	Gastritis and duodenitis	Male	35.59800334	33.91728739	37.2787193	2021
Middle SDI	Gastritis and duodenitis	Male	34.88500444	32.61125755	37.15875133	2022
Middle SDI	Gastritis and duodenitis	Male	34.18535139	31.23771758	37.1329852	2023
Middle SDI	Gastritis and duodenitis	Male	33.50121018	29.8223608	37.18005956	2024
Middle SDI	Gastritis and duodenitis	Male	32.83447295	28.38143594	37.28750995	2025
Middle SDI	Gastritis and duodenitis	Male	32.18336486	26.92515839	37.44157133	2026
Middle SDI	Gastritis and duodenitis	Male	31.54600553	25.46010234	37.63190872	2027
Middle SDI	Gastritis and duodenitis	Male	30.92175426	23.99145343	37.85205509	2028
Middle SDI	Gastritis and duodenitis	Male	30.31163314	22.52403677	38.09922951	2029
Middle SDI	Gastritis and duodenitis	Male	29.71698542	21.06209492	38.37187591	2030
Middle SDI	Gastritis and duodenitis	Female	58.60893957	58.41248529	58.80539385	1990
Middle SDI	Gastritis and duodenitis	Female	57.25113751	57.06111085	57.44116417	1991
Middle SDI	Gastritis and duodenitis	Female	56.03237069	55.84702664	56.21771475	1992
Middle SDI	Gastritis and duodenitis	Female	54.54999371	54.36948402	54.7305034	1993
Middle SDI	Gastritis and duodenitis	Female	53.43009879	53.25392365	53.60627393	1994
Middle SDI	Gastritis and duodenitis	Female	52.18543224	52.01360265	52.35726182	1995
Middle SDI	Gastritis and duodenitis	Female	50.92079018	50.75331768	51.08826268	1996
Middle SDI	Gastritis and duodenitis	Female	49.98394795	49.82021919	50.14767671	1997
Middle SDI	Gastritis and duodenitis	Female	49.09436244	48.93432577	49.25439912	1998
Middle SDI	Gastritis and duodenitis	Female	48.51526723	48.3582076	48.67232687	1999

Middle SDI	Gastritis and duodenitis	Female	48.44082306	48.28566433	48.5959818	2000
Middle SDI	Gastritis and duodenitis	Female	48.20619392	48.05322734	48.3591605	2001
Middle SDI	Gastritis and duodenitis	Female	48.29879142	48.14743706	48.45014579	2002
Middle SDI	Gastritis and duodenitis	Female	48.45764025	48.30781509	48.6074654	2003
Middle SDI	Gastritis and duodenitis	Female	48.31223731	48.16451118	48.45996344	2004
Middle SDI	Gastritis and duodenitis	Female	47.79428534	47.64929507	47.93927561	2005
Middle SDI	Gastritis and duodenitis	Female	46.89124675	46.74979774	47.03269576	2006
Middle SDI	Gastritis and duodenitis	Female	45.60078399	45.46350365	45.73806434	2007
Middle SDI	Gastritis and duodenitis	Female	44.57928319	44.44563979	44.71292659	2008
Middle SDI	Gastritis and duodenitis	Female	43.69218264	43.56180243	43.82256286	2009
Middle SDI	Gastritis and duodenitis	Female	42.8794839	42.75209024	43.00687756	2010
Middle SDI	Gastritis and duodenitis	Female	42.13501642	42.0104833	42.25954954	2011
Middle SDI	Gastritis and duodenitis	Female	41.4129177	41.29115005	41.53468535	2012
Middle SDI	Gastritis and duodenitis	Female	40.85024364	40.73090649	40.9695808	2013
Middle SDI	Gastritis and duodenitis	Female	40.28388229	40.16691947	40.4008451	2014
Middle SDI	Gastritis and duodenitis	Female	39.79926302	39.68446925	39.91405678	2015
Middle SDI	Gastritis and duodenitis	Female	39.58135063	39.46833259	39.69436868	2016
Middle SDI	Gastritis and duodenitis	Female	39.40557659	39.29419556	39.51695761	2017
Middle SDI	Gastritis and duodenitis	Female	38.84313489	38.73380661	38.95246318	2018
Middle SDI	Gastritis and duodenitis	Female	38.22090628	38.11299999	38.32881258	2019
Middle SDI	Gastritis and duodenitis	Female	38.11588116	36.91312034	39.31864199	2020
Middle SDI	Gastritis and duodenitis	Female	37.73250594	36.00465447	39.4603574	2021
Middle SDI	Gastritis and duodenitis	Female	37.35255571	34.95772538	39.74738604	2022
Middle SDI	Gastritis and duodenitis	Female	36.97632352	33.81461075	40.13803628	2023
Middle SDI	Gastritis and duodenitis	Female	36.6057548	32.59953123	40.61197836	2024
Middle SDI	Gastritis and duodenitis	Female	36.24121837	31.32640297	41.15603377	2025
Middle SDI	Gastritis and duodenitis	Female	35.88104475	30.00495786	41.75713165	2026
Middle SDI	Gastritis and duodenitis	Female	35.52445145	28.64241663	42.40648627	2027
Middle SDI	Gastritis and duodenitis	Female	35.17128215	27.24428828	43.09827601	2028
Middle SDI	Gastritis and duodenitis	Female	34.82277027	25.81560525	43.82993529	2029
Middle SDI	Gastritis and duodenitis	Female	34.47889009	24.35969899	44.59808119	2030
Middle SDI	Gastritis and duodenitis	Both	61.97969686	60.75629667	63.20309705	1990
Middle SDI	Gastritis and duodenitis	Both	60.53325788	59.34059457	61.72592119	1991
Middle SDI	Gastritis and duodenitis	Both	59.04255754	57.87998839	60.2051267	1992
Middle SDI	Gastritis and duodenitis	Both	57.42080653	56.28882753	58.55278553	1993
Middle SDI	Gastritis and duodenitis	Both	56.08089605	54.97591795	57.18587415	1994
Middle SDI	Gastritis and duodenitis	Both	54.81592811	53.73646632	55.8953899	1995
Middle SDI	Gastritis and duodenitis	Both	53.42576478	52.3729599	54.47856966	1996
Middle SDI	Gastritis and duodenitis	Both	52.04883476	51.02207366	53.07559586	1997
Middle SDI	Gastritis and duodenitis	Both	50.91593187	49.91286444	51.9189993	1998
Middle SDI	Gastritis and duodenitis	Both	50.1031382	49.11978997	51.08648643	1999
Middle SDI	Gastritis and duodenitis	Both	49.86840814	48.89804742	50.83876885	2000
Middle SDI	Gastritis and duodenitis	Both	49.53614408	48.57938881	50.49289935	2001
Middle SDI	Gastritis and duodenitis	Both	49.50787808	48.56253815	50.45321801	2002
Middle SDI	Gastritis and duodenitis	Both	49.50918795	48.57509206	50.44328384	2003
Middle SDI	Gastritis and duodenitis	Both	49.29379372	48.37268402	50.21490342	2004
Middle SDI	Gastritis and duodenitis	Both	48.62243259	47.71844128	49.52642389	2005
Middle SDI	Gastritis and duodenitis	Both	47.54307369	46.66118442	48.42496295	2006
Middle SDI	Gastritis and duodenitis	Both	46.10432816	45.24771961	46.96093671	2007
Middle SDI	Gastritis and duodenitis	Both	45.00793814	44.17263581	45.84324048	2008
Middle SDI	Gastritis and duodenitis	Both	44.03253595	43.2165783	44.8484936	2009
Middle SDI	Gastritis and duodenitis	Both	43.15061238	42.35272931	43.94849545	2010
Middle SDI	Gastritis and duodenitis	Both	42.36900735	41.58864607	43.14936862	2011
Middle SDI	Gastritis and duodenitis	Both	41.65465745	40.89083498	42.41847993	2012
Middle SDI	Gastritis and duodenitis	Both	41.03884522	40.28997804	41.7877124	2013
Middle SDI	Gastritis and duodenitis	Both	40.34010894	39.60641815	41.07379973	2014
Middle SDI	Gastritis and duodenitis	Both	39.76617957	39.04624393	40.4861152	2015
Middle SDI	Gastritis and duodenitis	Both	39.40298468	38.69489827	40.1110711	2016

Middle SDI	Gastritis and duodenitis	Both	38.98068986	38.2843716	39.67700812	2017
Middle SDI	Gastritis and duodenitis	Both	38.09023586	37.40925888	38.77121284	2018
Middle SDI	Gastritis and duodenitis	Both	37.29201293	36.62493889	37.95908697	2019
Middle SDI	Gastritis and duodenitis	Both	37.18799058	34.08317149	40.29280966	2020
Middle SDI	Gastritis and duodenitis	Both	36.64018193	33.33495768	39.94540618	2021
Middle SDI	Gastritis and duodenitis	Both	36.10557195	32.45495675	39.75618715	2022
Middle SDI	Gastritis and duodenitis	Both	35.58511785	31.4503228	39.71991291	2023
Middle SDI	Gastritis and duodenitis	Both	35.08190393	30.34202123	39.82178663	2024
Middle SDI	Gastritis and duodenitis	Both	34.5982229	29.1526159	40.0438299	2025
Middle SDI	Gastritis and duodenitis	Both	34.13321215	27.90108936	40.36533493	2026
Middle SDI	Gastritis and duodenitis	Both	33.68634697	26.60168594	40.77100801	2027
Middle SDI	Gastritis and duodenitis	Both	33.25777582	25.26453577	41.25101588	2028
Middle SDI	Gastritis and duodenitis	Both	32.84934674	23.89736659	41.8013269	2029
Middle SDI	Gastritis and duodenitis	Both	32.46261421	22.50522232	42.4200061	2030
Low-middle SDI	Gastritis and duodenitis	Male	63.82701491	63.56505855	64.08897127	1990
Low-middle SDI	Gastritis and duodenitis	Male	63.22872417	62.97328505	63.4841633	1991
Low-middle SDI	Gastritis and duodenitis	Male	62.37231312	62.12204935	62.6225769	1992
Low-middle SDI	Gastritis and duodenitis	Male	61.277816	61.03271399	61.522918	1993
Low-middle SDI	Gastritis and duodenitis	Male	60.10945411	59.86955592	60.34935229	1994
Low-middle SDI	Gastritis and duodenitis	Male	58.9595685	58.72467473	59.19446227	1995
Low-middle SDI	Gastritis and duodenitis	Male	57.87867543	57.64854506	58.1088058	1996
Low-middle SDI	Gastritis and duodenitis	Male	57.14143945	56.91529272	57.36758619	1997
Low-middle SDI	Gastritis and duodenitis	Male	56.1844625	55.96263531	56.4062897	1998
Low-middle SDI	Gastritis and duodenitis	Male	55.12878843	54.9115533	55.34602355	1999
Low-middle SDI	Gastritis and duodenitis	Male	54.36574541	54.15216222	54.5793286	2000
Low-middle SDI	Gastritis and duodenitis	Male	53.65320415	53.4430921	53.8633162	2001
Low-middle SDI	Gastritis and duodenitis	Male	52.90908773	52.70257625	53.11559921	2002
Low-middle SDI	Gastritis and duodenitis	Male	51.99364132	51.7911104	52.19617224	2003
Low-middle SDI	Gastritis and duodenitis	Male	51.05101976	50.85279956	51.24923996	2004
Low-middle SDI	Gastritis and duodenitis	Male	50.31782407	50.12333033	50.51231782	2005
Low-middle SDI	Gastritis and duodenitis	Male	49.57489247	49.38454985	49.7652351	2006
Low-middle SDI	Gastritis and duodenitis	Male	48.67633663	48.49068349	48.86198978	2007
Low-middle SDI	Gastritis and duodenitis	Male	48.23396616	48.05193493	48.41599739	2008
Low-middle SDI	Gastritis and duodenitis	Male	47.47861083	47.30073775	47.65648391	2009
Low-middle SDI	Gastritis and duodenitis	Male	46.74206868	46.56819382	46.91594353	2010
Low-middle SDI	Gastritis and duodenitis	Male	46.12026564	45.9500668	46.29046448	2011
Low-middle SDI	Gastritis and duodenitis	Male	45.45433997	45.28774558	45.62093436	2012
Low-middle SDI	Gastritis and duodenitis	Male	45.02645038	44.86266165	45.19023911	2013
Low-middle SDI	Gastritis and duodenitis	Male	44.50731558	44.3463178	44.66831337	2014
Low-middle SDI	Gastritis and duodenitis	Male	44.22564775	44.06703429	44.38426122	2015
Low-middle SDI	Gastritis and duodenitis	Male	44.34970448	44.1929802	44.50642875	2016
Low-middle SDI	Gastritis and duodenitis	Male	44.37910678	44.22438327	44.53383029	2017
Low-middle SDI	Gastritis and duodenitis	Male	43.38018129	43.22911999	43.53124258	2018
Low-middle SDI	Gastritis and duodenitis	Male	42.41767117	42.26930087	42.56604147	2019
Low-middle SDI	Gastritis and duodenitis	Male	42.16996942	40.8099958	43.52994304	2020
Low-middle SDI	Gastritis and duodenitis	Male	41.66316634	39.89592254	43.43041014	2021
Low-middle SDI	Gastritis and duodenitis	Male	41.16559568	38.87287588	43.45831549	2022
Low-middle SDI	Gastritis and duodenitis	Male	40.67391687	37.77008904	43.5777447	2023
Low-middle SDI	Gastritis and duodenitis	Male	40.19064924	36.61033466	43.77096382	2024
Low-middle SDI	Gastritis and duodenitis	Male	39.72020673	35.41098526	44.02942821	2025
Low-middle SDI	Gastritis and duodenitis	Male	39.26232665	34.18328601	44.34136728	2026
Low-middle SDI	Gastritis and duodenitis	Male	38.81379022	32.93180677	44.69577367	2027
Low-middle SDI	Gastritis and duodenitis	Male	38.37139376	31.65850792	45.08427961	2028
Low-middle SDI	Gastritis and duodenitis	Male	37.93668578	30.3676353	45.50573625	2029
Low-middle SDI	Gastritis and duodenitis	Male	37.51267746	29.06398635	45.96136857	2030
Low-middle SDI	Gastritis and duodenitis	Female	59.45461919	59.1994243	59.70981408	1990
Low-middle SDI	Gastritis and duodenitis	Female	58.57901337	58.33144417	58.82658258	1991
Low-middle SDI	Gastritis and duodenitis	Female	57.87784753	57.63534081	58.12035425	1992

Low-middle SDI	Gastritis and duodenitis	Female	56.99385281	56.75626055	57.23144506	1993
Low-middle SDI	Gastritis and duodenitis	Female	56.26471214	56.03180135	56.49762293	1994
Low-middle SDI	Gastritis and duodenitis	Female	55.46515611	55.23695156	55.69336066	1995
Low-middle SDI	Gastritis and duodenitis	Female	54.82548729	54.60151713	55.04945746	1996
Low-middle SDI	Gastritis and duodenitis	Female	54.49129203	54.27098122	54.71160284	1997
Low-middle SDI	Gastritis and duodenitis	Female	53.82195383	53.60578151	54.03812615	1998
Low-middle SDI	Gastritis and duodenitis	Female	53.22544854	53.01304315	53.43785394	1999
Low-middle SDI	Gastritis and duodenitis	Female	52.92045361	52.71100389	53.12990334	2000
Low-middle SDI	Gastritis and duodenitis	Female	52.50601746	52.2997594	52.71227553	2001
Low-middle SDI	Gastritis and duodenitis	Female	52.14904041	51.94566144	52.35241939	2002
Low-middle SDI	Gastritis and duodenitis	Female	51.6903519	51.49011627	51.89058753	2003
Low-middle SDI	Gastritis and duodenitis	Female	51.17902982	50.98220806	51.37585157	2004
Low-middle SDI	Gastritis and duodenitis	Female	50.80291339	50.60946789	50.9963589	2005
Low-middle SDI	Gastritis and duodenitis	Female	50.39950846	50.21006695	50.58894997	2006
Low-middle SDI	Gastritis and duodenitis	Female	50.13467096	49.94912366	50.32021826	2007
Low-middle SDI	Gastritis and duodenitis	Female	50.22467546	50.042209	50.40714192	2008
Low-middle SDI	Gastritis and duodenitis	Female	50.10305479	49.9240572	50.28205239	2009
Low-middle SDI	Gastritis and duodenitis	Female	49.96651672	49.79075716	50.14227627	2010
Low-middle SDI	Gastritis and duodenitis	Female	49.5827447	49.41040784	49.75508156	2011
Low-middle SDI	Gastritis and duodenitis	Female	48.89703244	48.72849335	49.06557154	2012
Low-middle SDI	Gastritis and duodenitis	Female	48.61043302	48.444695	48.77617103	2013
Low-middle SDI	Gastritis and duodenitis	Female	48.22834393	48.06543981	48.39124805	2014
Low-middle SDI	Gastritis and duodenitis	Female	48.16420584	48.00354238	48.32486929	2015
Low-middle SDI	Gastritis and duodenitis	Female	48.41905862	48.26020506	48.57791217	2016
Low-middle SDI	Gastritis and duodenitis	Female	48.70320509	48.54599498	48.8604152	2017
Low-middle SDI	Gastritis and duodenitis	Female	48.0764226	47.92218591	48.2306593	2018
Low-middle SDI	Gastritis and duodenitis	Female	47.27908265	47.12726843	47.43089687	2019
Low-middle SDI	Gastritis and duodenitis	Female	47.11627966	45.65677155	48.57578777	2020
Low-middle SDI	Gastritis and duodenitis	Female	46.79062742	44.85812405	48.7231308	2021
Low-middle SDI	Gastritis and duodenitis	Female	46.46313052	43.92011163	49.0061494	2022
Low-middle SDI	Gastritis and duodenitis	Female	46.13288149	42.87764482	49.38811817	2023
Low-middle SDI	Gastritis and duodenitis	Female	45.8023317	41.75515103	49.84951237	2024
Low-middle SDI	Gastritis and duodenitis	Female	45.47398184	40.56919636	50.37876732	2025
Low-middle SDI	Gastritis and duodenitis	Female	45.14492889	39.32883634	50.96102144	2026
Low-middle SDI	Gastritis and duodenitis	Female	44.81300271	38.04021382	51.58579159	2027
Low-middle SDI	Gastritis and duodenitis	Female	44.47718256	36.70806825	52.24629687	2028
Low-middle SDI	Gastritis and duodenitis	Female	44.13945147	35.33804077	52.94086218	2029
Low-middle SDI	Gastritis and duodenitis	Female	43.80225598	33.93543539	53.66907657	2030
Low-middle SDI	Gastritis and duodenitis	Both	61.80143254	60.24941855	63.35344652	1990
Low-middle SDI	Gastritis and duodenitis	Both	61.06106667	59.53891417	62.58321918	1991
Low-middle SDI	Gastritis and duodenitis	Both	60.27472561	58.78192026	61.76753097	1992
Low-middle SDI	Gastritis and duodenitis	Both	59.2787078	57.81665407	60.74076152	1993
Low-middle SDI	Gastritis and duodenitis	Both	58.32060888	56.88824148	59.75297629	1994
Low-middle SDI	Gastritis and duodenitis	Both	57.33888302	55.93557934	58.7421867	1995
Low-middle SDI	Gastritis and duodenitis	Both	56.4726569	55.09672863	57.84858517	1996
Low-middle SDI	Gastritis and duodenitis	Both	55.93061411	54.5780885	57.28313972	1997
Low-middle SDI	Gastritis and duodenitis	Both	55.11214485	53.78471968	56.43957003	1998
Low-middle SDI	Gastritis and duodenitis	Both	54.2846449	52.98175455	55.58753526	1999
Low-middle SDI	Gastritis and duodenitis	Both	53.74703509	52.46426848	55.0298017	2000
Low-middle SDI	Gastritis and duodenitis	Both	53.17818712	51.9157066	54.44066765	2001
Low-middle SDI	Gastritis and duodenitis	Both	52.62575682	51.38319757	53.86831607	2002
Low-middle SDI	Gastritis and duodenitis	Both	51.93782514	50.71671933	53.15893094	2003
Low-middle SDI	Gastritis and duodenitis	Both	51.21199295	50.01316359	52.41082231	2004
Low-middle SDI	Gastritis and duodenitis	Both	50.6539929	49.47615416	51.83183164	2005
Low-middle SDI	Gastritis and duodenitis	Both	50.07259013	48.91775834	51.22742192	2006
Low-middle SDI	Gastritis and duodenitis	Both	49.48577667	48.35431497	50.61723838	2007
Low-middle SDI	Gastritis and duodenitis	Both	49.30410579	48.19061026	50.41760132	2008
Low-middle SDI	Gastritis and duodenitis	Both	48.85888829	47.76644301	49.95133357	2009

Low-middle SDI	Gastritis and duodenitis	Both	48.41919475	47.34805883	49.49033066	2010
Low-middle SDI	Gastritis and duodenitis	Both	47.9163668	46.866972	48.96576159	2011
Low-middle SDI	Gastritis and duodenitis	Both	47.23778032	46.21146076	48.26409989	2012
Low-middle SDI	Gastritis and duodenitis	Both	46.87902375	45.87032564	47.88772185	2013
Low-middle SDI	Gastritis and duodenitis	Both	46.42854201	45.43718666	47.41989736	2014
Low-middle SDI	Gastritis and duodenitis	Both	46.25748901	45.28088298	47.23409505	2015
Low-middle SDI	Gastritis and duodenitis	Both	46.44960856	45.48451936	47.41469776	2016
Low-middle SDI	Gastritis and duodenitis	Both	46.61072975	45.65690529	47.5645542	2017
Low-middle SDI	Gastritis and duodenitis	Both	45.80298813	44.86975571	46.73622056	2018
Low-middle SDI	Gastritis and duodenitis	Both	44.92567507	44.01270571	45.83864443	2019
Low-middle SDI	Gastritis and duodenitis	Both	44.75178922	40.77023069	48.73334776	2020
Low-middle SDI	Gastritis and duodenitis	Both	44.34009914	40.19717527	48.48302301	2021
Low-middle SDI	Gastritis and duodenitis	Both	43.9356264	39.52081723	48.35043556	2022
Low-middle SDI	Gastritis and duodenitis	Both	43.53616695	38.73560512	48.33672878	2023
Low-middle SDI	Gastritis and duodenitis	Both	43.14473104	37.84885808	48.44060401	2024
Low-middle SDI	Gastritis and duodenitis	Both	42.76603036	36.87458215	48.65747858	2025
Low-middle SDI	Gastritis and duodenitis	Both	42.39935329	35.82632196	48.97238463	2026
Low-middle SDI	Gastritis and duodenitis	Both	42.04281662	34.71500581	49.37062743	2027
Low-middle SDI	Gastritis and duodenitis	Both	41.69453859	33.54899336	49.84008383	2028
Low-middle SDI	Gastritis and duodenitis	Both	41.35670006	32.33713256	50.37626755	2029
Low-middle SDI	Gastritis and duodenitis	Both	41.03300984	31.08762978	50.97838989	2030
Low SDI	Gastritis and duodenitis	Male	85.4488192	85.0152368	85.8824016	1990
Low SDI	Gastritis and duodenitis	Male	85.03707526	84.62738211	85.4467684	1991
Low SDI	Gastritis and duodenitis	Male	84.48384803	84.08128625	84.8864098	1992
Low SDI	Gastritis and duodenitis	Male	83.90757307	83.51094206	84.30420408	1993
Low SDI	Gastritis and duodenitis	Male	83.42157487	83.0304807	83.81266904	1994
Low SDI	Gastritis and duodenitis	Male	82.79706455	82.41144669	83.18268241	1995
Low SDI	Gastritis and duodenitis	Male	82.34549073	81.96515655	82.7258249	1996
Low SDI	Gastritis and duodenitis	Male	81.66635719	81.29163034	82.04108403	1997
Low SDI	Gastritis and duodenitis	Male	81.17315359	80.80340511	81.54290207	1998
Low SDI	Gastritis and duodenitis	Male	80.4113888	80.04728386	80.77549374	1999
Low SDI	Gastritis and duodenitis	Male	79.15267574	78.79528007	79.51007142	2000
Low SDI	Gastritis and duodenitis	Male	77.23614743	76.8875577	77.58473715	2001
Low SDI	Gastritis and duodenitis	Male	75.39824761	75.05838795	75.73810726	2002
Low SDI	Gastritis and duodenitis	Male	73.944466	73.6121787	74.27675331	2003
Low SDI	Gastritis and duodenitis	Male	72.3546715	72.03043425	72.67890875	2004
Low SDI	Gastritis and duodenitis	Male	70.8194692	70.50301875	71.13591964	2005
Low SDI	Gastritis and duodenitis	Male	69.93493913	69.62499925	70.244879	2006
Low SDI	Gastritis and duodenitis	Male	69.47774994	69.17293434	69.78256553	2007
Low SDI	Gastritis and duodenitis	Male	68.66284647	68.3638006	68.96189234	2008
Low SDI	Gastritis and duodenitis	Male	67.97605768	67.68248991	68.26962545	2009
Low SDI	Gastritis and duodenitis	Male	66.73110025	66.44431007	67.01789042	2010
Low SDI	Gastritis and duodenitis	Male	66.05282573	65.77106375	66.3345877	2011
Low SDI	Gastritis and duodenitis	Male	65.29814523	65.02180838	65.57448209	2012
Low SDI	Gastritis and duodenitis	Male	64.80350244	64.53192156	65.07508331	2013
Low SDI	Gastritis and duodenitis	Male	64.13102665	63.86464395	64.39740934	2014
Low SDI	Gastritis and duodenitis	Male	63.64591297	63.3840923	63.90773364	2015
Low SDI	Gastritis and duodenitis	Male	63.48566742	63.22795385	63.743381	2016
Low SDI	Gastritis and duodenitis	Male	63.21273592	62.95911471	63.46635714	2017
Low SDI	Gastritis and duodenitis	Male	62.44796388	62.19903123	62.69689653	2018
Low SDI	Gastritis and duodenitis	Male	61.59697516	61.3452883	61.84866201	2019
Low SDI	Gastritis and duodenitis	Male	60.97855537	59.69714554	62.25996521	2020
Low SDI	Gastritis and duodenitis	Male	60.30056889	58.33459368	62.2665441	2021
Low SDI	Gastritis and duodenitis	Male	59.63966501	56.81957063	62.45975939	2022
Low SDI	Gastritis and duodenitis	Male	58.98970685	55.19807388	62.78133981	2023
Low SDI	Gastritis and duodenitis	Male	58.34403409	53.48903151	63.19903668	2024
Low SDI	Gastritis and duodenitis	Male	57.70496419	51.70896669	63.70096169	2025
Low SDI	Gastritis and duodenitis	Male	57.0849461	49.88335181	64.2865404	2026

Low SDI	Gastritis and duodenitis	Male	56.48353444	48.02134488	64.94572399	2027
Low SDI	Gastritis and duodenitis	Male	55.89414356	46.12375188	65.66453524	2028
Low SDI	Gastritis and duodenitis	Male	55.30988396	44.18902256	66.43074536	2029
Low SDI	Gastritis and duodenitis	Male	54.73052348	42.22020097	67.24084599	2030
Low SDI	Gastritis and duodenitis	Female	61.73625056	61.34555456	62.12694656	1990
Low SDI	Gastritis and duodenitis	Female	61.30168	60.93328142	61.67007858	1991
Low SDI	Gastritis and duodenitis	Female	60.90367945	60.5425013	61.26485761	1992
Low SDI	Gastritis and duodenitis	Female	60.41320136	60.05751837	60.76888435	1993
Low SDI	Gastritis and duodenitis	Female	59.98320101	59.6329234	60.33347861	1994
Low SDI	Gastritis and duodenitis	Female	59.61250364	59.267385	59.95762227	1995
Low SDI	Gastritis and duodenitis	Female	59.39908338	59.05852489	59.73964187	1996
Low SDI	Gastritis and duodenitis	Female	59.08798515	58.75209559	59.4238747	1997
Low SDI	Gastritis and duodenitis	Female	58.87872132	58.54697751	59.21046514	1998
Low SDI	Gastritis and duodenitis	Female	58.47327295	58.14632818	58.80021773	1999
Low SDI	Gastritis and duodenitis	Female	57.82242533	57.50096573	58.14388493	2000
Low SDI	Gastritis and duodenitis	Female	57.14425767	56.8284596	57.46005575	2001
Low SDI	Gastritis and duodenitis	Female	56.50351835	56.19314227	56.81389443	2002
Low SDI	Gastritis and duodenitis	Female	55.90580151	55.60074216	56.21086087	2003
Low SDI	Gastritis and duodenitis	Female	55.31097386	55.01126603	55.61068169	2004
Low SDI	Gastritis and duodenitis	Female	54.85144773	54.55659399	55.14630147	2005
Low SDI	Gastritis and duodenitis	Female	54.45021111	54.16031084	54.74011139	2006
Low SDI	Gastritis and duodenitis	Female	54.26013858	53.97487439	54.54540277	2007
Low SDI	Gastritis and duodenitis	Female	54.30937165	54.02794331	54.59079999	2008
Low SDI	Gastritis and duodenitis	Female	54.23247632	53.95528197	54.50967066	2009
Low SDI	Gastritis and duodenitis	Female	54.02396535	53.75115645	54.29677426	2010
Low SDI	Gastritis and duodenitis	Female	53.67091907	53.40287221	53.93896593	2011
Low SDI	Gastritis and duodenitis	Female	53.30521796	53.04183712	53.56859879	2012
Low SDI	Gastritis and duodenitis	Female	52.93270969	52.67391037	53.19150902	2013
Low SDI	Gastritis and duodenitis	Female	52.7398859	52.48504476	52.99472704	2014
Low SDI	Gastritis and duodenitis	Female	52.50585692	52.25497316	52.75674067	2015
Low SDI	Gastritis and duodenitis	Female	52.63028673	52.38282084	52.87775262	2016
Low SDI	Gastritis and duodenitis	Female	52.88538112	52.64093108	53.12983117	2017
Low SDI	Gastritis and duodenitis	Female	52.72551154	52.48438056	52.96664252	2018
Low SDI	Gastritis and duodenitis	Female	52.48873951	52.24540699	52.73207203	2019
Low SDI	Gastritis and duodenitis	Female	52.51177901	51.34177822	53.68177981	2020
Low SDI	Gastritis and duodenitis	Female	52.40719242	50.70600529	54.10837955	2021
Low SDI	Gastritis and duodenitis	Female	52.29778614	49.92003045	54.67554184	2022
Low SDI	Gastritis and duodenitis	Female	52.1821718	49.01986791	55.34447568	2023
Low SDI	Gastritis and duodenitis	Female	52.0602877	48.02507888	56.09549652	2024
Low SDI	Gastritis and duodenitis	Female	51.93227225	46.94752397	56.91702053	2025
Low SDI	Gastritis and duodenitis	Female	51.79651884	45.79605874	57.79697894	2026
Low SDI	Gastritis and duodenitis	Female	51.65171202	44.57679907	58.72662497	2027
Low SDI	Gastritis and duodenitis	Female	51.49574445	43.2932067	59.69828221	2028
Low SDI	Gastritis and duodenitis	Female	51.3281362	41.94877546	60.70749694	2029
Low SDI	Gastritis and duodenitis	Female	51.14981469	40.54762884	61.75200055	2030
Low SDI	Gastritis and duodenitis	Both	73.80189495	71.3896808	76.2141091	1990
Low SDI	Gastritis and duodenitis	Both	73.36809243	70.99593801	75.74024684	1991
Low SDI	Gastritis and duodenitis	Both	72.88850424	70.55264592	75.22436255	1992
Low SDI	Gastritis and duodenitis	Both	72.35563356	70.05494632	74.65632081	1993
Low SDI	Gastritis and duodenitis	Both	71.89833307	69.63156365	74.16510249	1994
Low SDI	Gastritis and duodenitis	Both	71.39849764	69.16549932	73.63149596	1995
Low SDI	Gastritis and duodenitis	Both	71.06885006	68.86730156	73.27039855	1996
Low SDI	Gastritis and duodenitis	Both	70.57716908	68.40812175	72.74621641	1997
Low SDI	Gastritis and duodenitis	Both	70.23244829	68.09274226	72.37215431	1998
Low SDI	Gastritis and duodenitis	Both	69.65342521	67.54676897	71.76008144	1999
Low SDI	Gastritis and duodenitis	Both	68.70450509	66.6356083	70.77340188	2000
Low SDI	Gastritis and duodenitis	Both	67.40889315	65.38404295	69.43374335	2001
Low SDI	Gastritis and duodenitis	Both	66.17209419	64.19069976	68.15348861	2002

Low SDI	Gastritis and duodenitis	Both	65.1443885	63.20297938	67.08579762	2003
Low SDI	Gastritis and duodenitis	Both	64.05127675	62.15095803	65.95159547	2004
Low SDI	Gastritis and duodenitis	Both	63.04774777	61.18653568	64.90895986	2005
Low SDI	Gastritis and duodenitis	Both	62.39901915	60.57277338	64.22526492	2006
Low SDI	Gastritis and duodenitis	Both	62.06702125	60.27104318	63.86299931	2007
Low SDI	Gastritis and duodenitis	Both	61.67559553	59.90943735	63.4417537	2008
Low SDI	Gastritis and duodenitis	Both	61.28683659	59.55136199	63.0223112	2009
Low SDI	Gastritis and duodenitis	Both	60.55682266	58.85659218	62.25705315	2010
Low SDI	Gastritis and duodenitis	Both	60.03736232	58.36955822	61.70516642	2011
Low SDI	Gastritis and duodenitis	Both	59.47637676	57.84169183	61.11106169	2012
Low SDI	Gastritis and duodenitis	Both	59.04119496	57.43688979	60.64550013	2013
Low SDI	Gastritis and duodenitis	Both	58.6077358	57.03318726	60.18228434	2014
Low SDI	Gastritis and duodenitis	Both	58.24617995	56.69972866	59.79263124	2015
Low SDI	Gastritis and duodenitis	Both	58.228994	56.70723109	59.75075691	2016
Low SDI	Gastritis and duodenitis	Both	58.21913006	56.72100534	59.71725477	2017
Low SDI	Gastritis and duodenitis	Both	57.75362615	56.28393884	59.22331346	2018
Low SDI	Gastritis and duodenitis	Both	57.20574102	55.76349808	58.64798396	2019
Low SDI	Gastritis and duodenitis	Both	56.8563173	53.7413302	59.9713044	2020
Low SDI	Gastritis and duodenitis	Both	56.46406425	53.05708288	59.87104562	2021
Low SDI	Gastritis and duodenitis	Both	56.08373978	52.19691186	59.9705677	2022
Low SDI	Gastritis and duodenitis	Both	55.71180448	51.17412512	60.24948384	2023
Low SDI	Gastritis and duodenitis	Both	55.34462437	50.01048839	60.67876035	2024
Low SDI	Gastritis and duodenitis	Both	54.98578634	48.73170224	61.23987043	2025
Low SDI	Gastritis and duodenitis	Both	54.64249769	47.36639338	61.918602	2026
Low SDI	Gastritis and duodenitis	Both	54.31556618	45.93119175	62.6999406	2027
Low SDI	Gastritis and duodenitis	Both	54.0006416	44.43372751	63.56755568	2028
Low SDI	Gastritis and duodenitis	Both	53.69403467	42.87758678	64.51048255	2029
Low SDI	Gastritis and duodenitis	Both	53.39747144	41.26804392	65.52689895	2030
Global	Gastroesophageal reflux disease	Male	69.09406319	68.98731746	69.20080892	1990
Global	Gastroesophageal reflux disease	Male	68.9397449	68.83831392	69.04117588	1991
Global	Gastroesophageal reflux disease	Male	68.81564149	68.71544801	68.91583498	1992
Global	Gastroesophageal reflux disease	Male	68.71756988	68.61861123	68.81652853	1993
Global	Gastroesophageal reflux disease	Male	68.65328933	68.55540806	68.7511706	1994
Global	Gastroesophageal reflux disease	Male	68.64441293	68.54748029	68.74134557	1995
Global	Gastroesophageal reflux disease	Male	68.6915188	68.59548672	68.78755088	1996
Global	Gastroesophageal reflux disease	Male	68.77981712	68.68465123	68.87498301	1997
Global	Gastroesophageal reflux disease	Male	68.87777041	68.78343759	68.97210323	1998
Global	Gastroesophageal reflux disease	Male	68.96609229	68.87258187	69.05960272	1999
Global	Gastroesophageal reflux disease	Male	69.0310716	68.93836396	69.12377924	2000
Global	Gastroesophageal reflux disease	Male	69.04818171	68.95632213	69.14004128	2001
Global	Gastroesophageal reflux disease	Male	69.02221562	68.93124289	69.11318836	2002
Global	Gastroesophageal reflux disease	Male	68.97994499	68.88985632	69.07003366	2003
Global	Gastroesophageal reflux disease	Male	68.9418324	68.85261542	69.03104937	2004
Global	Gastroesophageal reflux disease	Male	68.9366508	68.84824926	69.02505234	2005
Global	Gastroesophageal reflux disease	Male	68.87279133	68.78525744	68.96032523	2006
Global	Gastroesophageal reflux disease	Male	68.71611094	68.62952392	68.80269797	2007
Global	Gastroesophageal reflux disease	Male	68.54920171	68.46354406	68.63485937	2008
Global	Gastroesophageal reflux disease	Male	68.45704499	68.37223095	68.54185902	2009
Global	Gastroesophageal reflux disease	Male	68.50811368	68.42400912	68.59221824	2010
Global	Gastroesophageal reflux disease	Male	68.68564908	68.60216394	68.76913423	2011
Global	Gastroesophageal reflux disease	Male	68.91623804	68.8333331	68.99914298	2012
Global	Gastroesophageal reflux disease	Male	69.16767734	69.0853204	69.25003429	2013
Global	Gastroesophageal reflux disease	Male	69.41857736	69.33674752	69.5004072	2014
Global	Gastroesophageal reflux disease	Male	69.65626093	69.57493122	69.73759064	2015
Global	Gastroesophageal reflux disease	Male	70.1855942	70.10457	70.26661841	2016
Global	Gastroesophageal reflux disease	Male	70.68757586	70.60681376	70.76833797	2017
Global	Gastroesophageal reflux disease	Male	70.89039301	70.81010332	70.97068271	2018
Global	Gastroesophageal reflux disease	Male	71.13795274	71.05630229	71.21960319	2019

Global	Gastroesophageal reflux disease	Male	71.40790413	70.77389724	72.04191101	2020
Global	Gastroesophageal reflux disease	Male	71.66776865	70.52539978	72.81013752	2021
Global	Gastroesophageal reflux disease	Male	71.92575379	70.15103833	73.70046924	2022
Global	Gastroesophageal reflux disease	Male	72.17457848	69.67043514	74.67872182	2023
Global	Gastroesophageal reflux disease	Male	72.42232907	69.1013052	75.74335294	2024
Global	Gastroesophageal reflux disease	Male	72.6731211	68.44914694	76.89709527	2025
Global	Gastroesophageal reflux disease	Male	72.92870433	67.72575314	78.13165551	2026
Global	Gastroesophageal reflux disease	Male	73.17888587	66.92821946	79.42955228	2027
Global	Gastroesophageal reflux disease	Male	73.41771937	66.05560051	80.77983822	2028
Global	Gastroesophageal reflux disease	Male	73.65326965	65.1163554	82.1901839	2029
Global	Gastroesophageal reflux disease	Male	73.88941921	64.11131365	83.66752477	2030
Global	Gastroesophageal reflux disease	Female	74.27234058	74.16382862	74.38085255	1990
Global	Gastroesophageal reflux disease	Female	74.20387828	74.09984802	74.30790855	1991
Global	Gastroesophageal reflux disease	Female	74.15105462	74.04810545	74.25400379	1992
Global	Gastroesophageal reflux disease	Female	74.10469044	74.00285999	74.20652088	1993
Global	Gastroesophageal reflux disease	Female	74.07022211	73.96940823	74.17103599	1994
Global	Gastroesophageal reflux disease	Female	74.05970071	73.95983108	74.15957034	1995
Global	Gastroesophageal reflux disease	Female	74.10924567	74.01028873	74.20820262	1996
Global	Gastroesophageal reflux disease	Female	74.22841134	74.13032607	74.32649661	1997
Global	Gastroesophageal reflux disease	Female	74.36321014	74.26597437	74.46044591	1998
Global	Gastroesophageal reflux disease	Female	74.4805964	74.38421051	74.57698228	1999
Global	Gastroesophageal reflux disease	Female	74.54708803	74.45155425	74.64262181	2000
Global	Gastroesophageal reflux disease	Female	74.46394116	74.36937379	74.55850853	2001
Global	Gastroesophageal reflux disease	Female	74.23705187	74.14355075	74.33055299	2002
Global	Gastroesophageal reflux disease	Female	73.94482337	73.85241369	74.03723305	2003
Global	Gastroesophageal reflux disease	Female	73.68513482	73.59377605	73.77649359	2004
Global	Gastroesophageal reflux disease	Female	73.5384535	73.44803763	73.62886938	2005
Global	Gastroesophageal reflux disease	Female	73.42761362	73.33811776	73.51710948	2006
Global	Gastroesophageal reflux disease	Female	73.26286495	73.17432725	73.35140266	2007
Global	Gastroesophageal reflux disease	Female	73.11733905	73.02972333	73.20495477	2008
Global	Gastroesophageal reflux disease	Female	73.0539665	72.96718564	73.14074736	2009
Global	Gastroesophageal reflux disease	Female	73.14114849	73.05507428	73.22722269	2010
Global	Gastroesophageal reflux disease	Female	73.34333869	73.25788803	73.42878934	2011
Global	Gastroesophageal reflux disease	Female	73.58783919	73.5029759	73.67270248	2012
Global	Gastroesophageal reflux disease	Female	73.84885186	73.76454699	73.93315673	2013
Global	Gastroesophageal reflux disease	Female	74.10605749	74.02228465	74.18983034	2014
Global	Gastroesophageal reflux disease	Female	74.35335206	74.27007746	74.43662666	2015
Global	Gastroesophageal reflux disease	Female	75.03987458	74.9568284	75.12292077	2016
Global	Gastroesophageal reflux disease	Female	75.6983216	75.61546	75.78118321	2017
Global	Gastroesophageal reflux disease	Female	75.90684694	75.82445309	75.9892408	2018
Global	Gastroesophageal reflux disease	Female	76.15690389	76.073411	76.24039678	2019
Global	Gastroesophageal reflux disease	Female	76.3907228	75.7035968	77.07784879	2020
Global	Gastroesophageal reflux disease	Female	76.64624781	75.39580856	77.89668705	2021
Global	Gastroesophageal reflux disease	Female	76.89899388	74.94808606	78.8499017	2022
Global	Gastroesophageal reflux disease	Female	77.13826201	74.3797291	79.89679491	2023
Global	Gastroesophageal reflux disease	Female	77.37431174	73.71195668	81.0366668	2024
Global	Gastroesophageal reflux disease	Female	77.61312048	72.95262773	82.27361324	2025
Global	Gastroesophageal reflux disease	Female	77.86039662	72.11788889	83.60290434	2026
Global	Gastroesophageal reflux disease	Female	78.10198492	71.20173881	85.00223102	2027
Global	Gastroesophageal reflux disease	Female	78.32960071	70.2017266	86.45747481	2028
Global	Gastroesophageal reflux disease	Female	78.55280732	69.12810007	87.97751457	2029
Global	Gastroesophageal reflux disease	Female	78.77587326	67.98215179	89.56959474	2030
Global	Gastroesophageal reflux disease	Both	71.69835448	71.11784962	72.27885934	1990
Global	Gastroesophageal reflux disease	Both	71.584733	71.01222678	72.15723921	1991
Global	Gastroesophageal reflux disease	Both	71.49430638	70.92849113	72.06012163	1992
Global	Gastroesophageal reflux disease	Both	71.42035211	70.86079406	71.97991016	1993
Global	Gastroesophageal reflux disease	Both	71.36980898	70.81624215	71.92337581	1994
Global	Gastroesophageal reflux disease	Both	71.35949446	70.8115823	71.90740661	1995

Global	Gastroesophageal reflux disease	Both	71.40773563	70.86515879	71.95031247	1996
Global	Gastroesophageal reflux disease	Both	71.51190087	70.97433305	72.04946869	1997
Global	Gastroesophageal reflux disease	Both	71.62889646	71.09619853	72.16159439	1998
Global	Gastroesophageal reflux disease	Both	71.73250835	71.20471711	72.26029958	1999
Global	Gastroesophageal reflux disease	Both	71.79896417	71.27613105	72.3217973	2000
Global	Gastroesophageal reflux disease	Both	71.76625996	71.24875139	72.28376852	2001
Global	Gastroesophageal reflux disease	Both	71.63945688	71.12758181	72.15133195	2002
Global	Gastroesophageal reflux disease	Both	71.47144829	70.96525118	71.97764539	2003
Global	Gastroesophageal reflux disease	Both	71.32181147	70.8211995	71.82242344	2004
Global	Gastroesophageal reflux disease	Both	71.24550162	70.7500932	71.74091005	2005
Global	Gastroesophageal reflux disease	Both	71.15801445	70.66785622	71.64817268	2006
Global	Gastroesophageal reflux disease	Both	70.99734577	70.51271679	71.48197474	2007
Global	Gastroesophageal reflux disease	Both	70.84146886	70.36220228	71.32073543	2008
Global	Gastroesophageal reflux disease	Both	70.76443231	70.29007816	71.23878647	2009
Global	Gastroesophageal reflux disease	Both	70.83442381	70.36428302	71.3045646	2010
Global	Gastroesophageal reflux disease	Both	71.02497884	70.55857057	71.4913871	2011
Global	Gastroesophageal reflux disease	Both	71.26302832	70.80014459	71.72591204	2012
Global	Gastroesophageal reflux disease	Both	71.51969724	71.060164	71.97923048	2013
Global	Gastroesophageal reflux disease	Both	71.77420239	71.31788953	72.23051526	2014
Global	Gastroesophageal reflux disease	Both	72.01699392	71.56370931	72.47027854	2015
Global	Gastroesophageal reflux disease	Both	72.62603894	72.17463359	73.0774443	2016
Global	Gastroesophageal reflux disease	Both	73.20722339	72.757724	73.65672278	2017
Global	Gastroesophageal reflux disease	Both	73.41235823	72.96577852	73.85893794	2018
Global	Gastroesophageal reflux disease	Both	73.66019284	73.21573417	74.10465152	2019
Global	Gastroesophageal reflux disease	Both	73.91679836	72.73189347	75.10170325	2020
Global	Gastroesophageal reflux disease	Both	74.1762077	72.62008197	75.73233343	2021
Global	Gastroesophageal reflux disease	Both	74.43493076	72.31771688	76.55214464	2022
Global	Gastroesophageal reflux disease	Both	74.68461052	71.86248802	77.50673302	2023
Global	Gastroesophageal reflux disease	Both	74.93514166	71.29131355	78.57896976	2024
Global	Gastroesophageal reflux disease	Both	75.19243834	70.62121589	79.7636608	2025
Global	Gastroesophageal reflux disease	Both	75.46091648	69.87283294	81.04900003	2026
Global	Gastroesophageal reflux disease	Both	75.72900278	69.0445954	82.41341015	2027
Global	Gastroesophageal reflux disease	Both	75.99019857	68.13596752	83.84442962	2028
Global	Gastroesophageal reflux disease	Both	76.25412703	67.15685431	85.35139975	2029
Global	Gastroesophageal reflux disease	Both	76.52590976	66.10883492	86.94298459	2030
High SDI	Gastroesophageal reflux disease	Male	62.93849167	62.71592599	63.16105735	1990
High SDI	Gastroesophageal reflux disease	Male	62.78329826	62.5809696	62.98562692	1991
High SDI	Gastroesophageal reflux disease	Male	62.65358165	62.45279308	62.85437021	1992
High SDI	Gastroesophageal reflux disease	Male	62.54641772	62.34737527	62.74546017	1993
High SDI	Gastroesophageal reflux disease	Male	62.4752472	62.27753783	62.67295657	1994
High SDI	Gastroesophageal reflux disease	Male	62.4573252	62.26059651	62.65405389	1995
High SDI	Gastroesophageal reflux disease	Male	62.48573345	62.2898234	62.6816435	1996
High SDI	Gastroesophageal reflux disease	Male	62.56468934	62.36952091	62.75985776	1997
High SDI	Gastroesophageal reflux disease	Male	62.65698565	62.46248807	62.85148323	1998
High SDI	Gastroesophageal reflux disease	Male	62.7375675	62.54371663	62.93141837	1999
High SDI	Gastroesophageal reflux disease	Male	62.76481256	62.57169102	62.95793411	2000
High SDI	Gastroesophageal reflux disease	Male	62.63408813	62.44201418	62.82616208	2001
High SDI	Gastroesophageal reflux disease	Male	62.30713739	62.11652351	62.49775126	2002
High SDI	Gastroesophageal reflux disease	Male	61.86045356	61.67146858	62.04943854	2003
High SDI	Gastroesophageal reflux disease	Male	61.36910389	61.1818099	61.55639789	2004
High SDI	Gastroesophageal reflux disease	Male	60.91625255	60.73057347	61.10193164	2005
High SDI	Gastroesophageal reflux disease	Male	60.44939276	60.26537633	60.63340919	2006
High SDI	Gastroesophageal reflux disease	Male	59.93907759	59.75689242	60.12126277	2007
High SDI	Gastroesophageal reflux disease	Male	59.48478279	59.30434153	59.66522405	2008
High SDI	Gastroesophageal reflux disease	Male	59.1933191	59.01424441	59.3723938	2009
High SDI	Gastroesophageal reflux disease	Male	59.14444909	58.96623879	59.3226594	2010
High SDI	Gastroesophageal reflux disease	Male	59.39104326	59.21318934	59.56889718	2011
High SDI	Gastroesophageal reflux disease	Male	59.82168589	59.64384894	59.99952284	2012

High SDI	Gastroesophageal reflux disease	Male	60.29678609	60.1188189	60.47475328	2013
High SDI	Gastroesophageal reflux disease	Male	60.69101297	60.51299435	60.86903158	2014
High SDI	Gastroesophageal reflux disease	Male	60.88650557	60.70873006	61.06428108	2015
High SDI	Gastroesophageal reflux disease	Male	60.96626542	60.78873632	61.14379453	2016
High SDI	Gastroesophageal reflux disease	Male	61.04252545	60.86492006	61.22013084	2017
High SDI	Gastroesophageal reflux disease	Male	61.30548148	61.12771514	61.48324782	2018
High SDI	Gastroesophageal reflux disease	Male	61.81446719	61.62379319	62.00514118	2019
High SDI	Gastroesophageal reflux disease	Male	62.12275862	61.34986848	62.89564875	2020
High SDI	Gastroesophageal reflux disease	Male	62.51774719	61.14688703	63.88860735	2021
High SDI	Gastroesophageal reflux disease	Male	62.9163463	60.79956481	65.03312778	2022
High SDI	Gastroesophageal reflux disease	Male	63.31934763	60.33751732	66.30117794	2023
High SDI	Gastroesophageal reflux disease	Male	63.72536315	59.77134957	67.67937674	2024
High SDI	Gastroesophageal reflux disease	Male	64.13056077	59.10304544	69.15807611	2025
High SDI	Gastroesophageal reflux disease	Male	64.53680891	58.34184825	70.73176956	2026
High SDI	Gastroesophageal reflux disease	Male	64.94449124	57.49320088	72.39578161	2027
High SDI	Gastroesophageal reflux disease	Male	65.35477844	56.56142471	74.14813216	2028
High SDI	Gastroesophageal reflux disease	Male	65.76713762	55.54741477	75.98686047	2029
High SDI	Gastroesophageal reflux disease	Male	66.17853989	54.44860613	77.90847366	2030
High SDI	Gastroesophageal reflux disease	Female	67.77012813	67.54359106	67.99666521	1990
High SDI	Gastroesophageal reflux disease	Female	67.65877056	67.44652267	67.87101846	1991
High SDI	Gastroesophageal reflux disease	Female	67.54386901	67.3329581	67.75477993	1992
High SDI	Gastroesophageal reflux disease	Female	67.43545922	67.22606364	67.64485479	1993
High SDI	Gastroesophageal reflux disease	Female	67.34915302	67.14096903	67.55733701	1994
High SDI	Gastroesophageal reflux disease	Female	67.2840614	67.07685179	67.49127101	1995
High SDI	Gastroesophageal reflux disease	Female	67.22831542	67.02197898	67.43465186	1996
High SDI	Gastroesophageal reflux disease	Female	67.15574717	66.95033953	67.3611548	1997
High SDI	Gastroesophageal reflux disease	Female	67.06521515	66.86070029	67.26973	1998
High SDI	Gastroesophageal reflux disease	Female	66.97176807	66.76810814	67.17542801	1999
High SDI	Gastroesophageal reflux disease	Female	66.86179431	66.65895156	67.06463706	2000
High SDI	Gastroesophageal reflux disease	Female	66.49470434	66.29320446	66.69620423	2001
High SDI	Gastroesophageal reflux disease	Female	65.74715648	65.54783957	65.94647339	2002
High SDI	Gastroesophageal reflux disease	Female	64.80059687	64.60378174	64.99741199	2003
High SDI	Gastroesophageal reflux disease	Female	63.87109345	63.67667402	64.06551287	2004
High SDI	Gastroesophageal reflux disease	Female	63.14714353	62.95471122	63.33957584	2005
High SDI	Gastroesophageal reflux disease	Female	62.57501037	62.38425023	62.7657705	2006
High SDI	Gastroesophageal reflux disease	Female	62.02451665	61.83540422	62.21362908	2007
High SDI	Gastroesophageal reflux disease	Female	61.58187725	61.3942029	61.7695516	2008
High SDI	Gastroesophageal reflux disease	Female	61.32076796	61.13419254	61.50734338	2009
High SDI	Gastroesophageal reflux disease	Female	61.34743904	61.16145966	61.53341843	2010
High SDI	Gastroesophageal reflux disease	Female	61.7135643	61.52760212	61.89952647	2011
High SDI	Gastroesophageal reflux disease	Female	62.31101317	62.12464996	62.49737639	2012
High SDI	Gastroesophageal reflux disease	Female	62.96727371	62.78032585	63.15422157	2013
High SDI	Gastroesophageal reflux disease	Female	63.5124265	63.32501395	63.69983904	2014
High SDI	Gastroesophageal reflux disease	Female	63.79979538	63.61230079	63.98728997	2015
High SDI	Gastroesophageal reflux disease	Female	63.91702193	63.72958156	64.1044623	2016
High SDI	Gastroesophageal reflux disease	Female	64.01667297	63.82904928	64.20429666	2017
High SDI	Gastroesophageal reflux disease	Female	64.37551858	64.18736221	64.56367496	2018
High SDI	Gastroesophageal reflux disease	Female	65.04441606	64.84574551	65.2430866	2019
High SDI	Gastroesophageal reflux disease	Female	65.20387187	64.31066045	66.09708329	2020
High SDI	Gastroesophageal reflux disease	Female	65.64208395	64.07047215	67.21369574	2021
High SDI	Gastroesophageal reflux disease	Female	66.08587356	63.66359971	68.5081474	2022
High SDI	Gastroesophageal reflux disease	Female	66.53474267	63.12373584	69.94574949	2023
High SDI	Gastroesophageal reflux disease	Female	66.98488968	62.46148536	71.508294	2024
High SDI	Gastroesophageal reflux disease	Female	67.4310406	61.67880344	73.18327776	2025
High SDI	Gastroesophageal reflux disease	Female	67.88048319	60.79084055	74.97012584	2026
High SDI	Gastroesophageal reflux disease	Female	68.33389989	59.8037648	76.86403499	2027
High SDI	Gastroesophageal reflux disease	Female	68.79135927	58.7211828	78.86153574	2028
High SDI	Gastroesophageal reflux disease	Female	69.24952682	57.54181579	80.95723785	2029

High SDI	Gastroesophageal reflux disease	Female	69.70342422	56.26179874	83.14504971	2030
High SDI	Gastroesophageal reflux disease	Both	65.39965625	64.24497462	66.55433788	1990
High SDI	Gastroesophageal reflux disease	Both	65.26286792	64.12003655	66.40569928	1991
High SDI	Gastroesophageal reflux disease	Both	65.1369283	64.00251456	66.27134203	1992
High SDI	Gastroesophageal reflux disease	Both	65.02535476	63.89854661	66.15216292	1993
High SDI	Gastroesophageal reflux disease	Both	64.94269397	63.82263136	66.06275657	1994
High SDI	Gastroesophageal reflux disease	Both	64.8967569	63.78280372	66.01071008	1995
High SDI	Gastroesophageal reflux disease	Both	64.87764982	63.76913168	65.98616797	1996
High SDI	Gastroesophageal reflux disease	Both	64.87462505	63.77139545	65.97785465	1997
High SDI	Gastroesophageal reflux disease	Both	64.86905542	63.77085346	65.96725738	1998
High SDI	Gastroesophageal reflux disease	Both	64.85722529	63.76391176	65.95053882	1999
High SDI	Gastroesophageal reflux disease	Both	64.81147811	63.72342652	65.8995297	2000
High SDI	Gastroesophageal reflux disease	Both	64.55885903	63.47796738	65.63975068	2001
High SDI	Gastroesophageal reflux disease	Both	64.01799845	62.94718966	65.08880725	2002
High SDI	Gastroesophageal reflux disease	Both	63.31833196	62.25881203	64.37785189	2003
High SDI	Gastroesophageal reflux disease	Both	62.60577212	61.55754136	63.65400288	2004
High SDI	Gastroesophageal reflux disease	Both	62.01624682	60.97817722	63.05431642	2005
High SDI	Gastroesophageal reflux disease	Both	61.4968963	60.46835347	62.52543913	2006
High SDI	Gastroesophageal reflux disease	Both	60.96750786	59.94900715	61.98600858	2007
High SDI	Gastroesophageal reflux disease	Both	60.52042842	59.5112578	61.52959905	2008
High SDI	Gastroesophageal reflux disease	Both	60.245655	59.24387566	61.24743434	2009
High SDI	Gastroesophageal reflux disease	Both	60.23535716	59.23806052	61.2326538	2010
High SDI	Gastroesophageal reflux disease	Both	60.54166284	59.54586369	61.53746198	2011
High SDI	Gastroesophageal reflux disease	Both	61.05486666	60.05881149	62.05092183	2012
High SDI	Gastroesophageal reflux disease	Both	61.61930095	60.62226685	62.61633505	2013
High SDI	Gastroesophageal reflux disease	Both	62.08746362	61.08997235	63.08495489	2014
High SDI	Gastroesophageal reflux disease	Both	62.32773005	61.33137956	63.32408053	2015
High SDI	Gastroesophageal reflux disease	Both	62.42555059	61.43112116	63.41998003	2016
High SDI	Gastroesophageal reflux disease	Both	62.51272407	61.51986318	63.50558495	2017
High SDI	Gastroesophageal reflux disease	Both	62.82036399	61.8265847	63.81414328	2018
High SDI	Gastroesophageal reflux disease	Both	63.40340335	62.4031628	64.40364391	2019
High SDI	Gastroesophageal reflux disease	Both	63.65631379	62.00287083	65.30975674	2020
High SDI	Gastroesophageal reflux disease	Both	64.07584285	62.00725048	66.14443522	2021
High SDI	Gastroesophageal reflux disease	Both	64.50275521	61.79056455	67.21494588	2022
High SDI	Gastroesophageal reflux disease	Both	64.93839665	61.39739271	68.47940058	2023
High SDI	Gastroesophageal reflux disease	Both	65.38146627	60.85912567	69.90380688	2024
High SDI	Gastroesophageal reflux disease	Both	65.82868074	60.19157476	71.46578673	2025
High SDI	Gastroesophageal reflux disease	Both	66.28564683	59.41518619	73.15610748	2026
High SDI	Gastroesophageal reflux disease	Both	66.75401667	58.54019647	74.96783686	2027
High SDI	Gastroesophageal reflux disease	Both	67.23572763	57.57291708	76.89853818	2028
High SDI	Gastroesophageal reflux disease	Both	67.73030156	56.51425154	78.94635158	2029
High SDI	Gastroesophageal reflux disease	Both	68.23525525	55.36131245	81.10919804	2030
High-middle SDI	Gastroesophageal reflux disease	Male	63.92164	63.7174023	64.1258777	1990
High-middle SDI	Gastroesophageal reflux disease	Male	63.64404741	63.45788675	63.83020807	1991
High-middle SDI	Gastroesophageal reflux disease	Male	63.39803425	63.21414349	63.58192501	1992
High-middle SDI	Gastroesophageal reflux disease	Male	63.17363667	62.99205248	63.35522087	1993
High-middle SDI	Gastroesophageal reflux disease	Male	62.97497376	62.79528073	63.15466678	1994
High-middle SDI	Gastroesophageal reflux disease	Male	62.8165187	62.63837724	62.99466017	1995
High-middle SDI	Gastroesophageal reflux disease	Male	62.67599053	62.49941202	62.85256903	1996
High-middle SDI	Gastroesophageal reflux disease	Male	62.53750537	62.36249768	62.71251305	1997
High-middle SDI	Gastroesophageal reflux disease	Male	62.39068927	62.21720439	62.56417416	1998
High-middle SDI	Gastroesophageal reflux disease	Male	62.23343859	62.06144662	62.40543057	1999
High-middle SDI	Gastroesophageal reflux disease	Male	62.08304546	61.91244796	62.25364296	2000
High-middle SDI	Gastroesophageal reflux disease	Male	61.93204861	61.76289639	62.10120083	2001
High-middle SDI	Gastroesophageal reflux disease	Male	61.7706805	61.6030014	61.93835959	2002
High-middle SDI	Gastroesophageal reflux disease	Male	61.61605797	61.44978859	61.78232735	2003
High-middle SDI	Gastroesophageal reflux disease	Male	61.47355669	61.30863548	61.63847791	2004
High-middle SDI	Gastroesophageal reflux disease	Male	61.36112901	61.19744481	61.5248132	2005

High-middle SDI	Gastroesophageal reflux disease	Male	61.18675213	61.02445114	61.34905313	2006
High-middle SDI	Gastroesophageal reflux disease	Male	60.90480749	60.74407945	61.06553553	2007
High-middle SDI	Gastroesophageal reflux disease	Male	60.58527698	60.42610045	60.74445352	2008
High-middle SDI	Gastroesophageal reflux disease	Male	60.30390792	60.14614989	60.46166595	2009
High-middle SDI	Gastroesophageal reflux disease	Male	60.15521972	59.99860873	60.31183071	2010
High-middle SDI	Gastroesophageal reflux disease	Male	60.13135791	59.97572023	60.28699559	2011
High-middle SDI	Gastroesophageal reflux disease	Male	60.15923991	60.00448224	60.31399758	2012
High-middle SDI	Gastroesophageal reflux disease	Male	60.21117251	60.05716598	60.36517905	2013
High-middle SDI	Gastroesophageal reflux disease	Male	60.28379735	60.13038334	60.43721135	2014
High-middle SDI	Gastroesophageal reflux disease	Male	60.42989903	60.27680188	60.58299618	2015
High-middle SDI	Gastroesophageal reflux disease	Male	61.24288678	61.08923717	61.39653639	2016
High-middle SDI	Gastroesophageal reflux disease	Male	62.03498271	61.88032907	62.18963636	2017
High-middle SDI	Gastroesophageal reflux disease	Male	62.1161402	61.96182576	62.27045465	2018
High-middle SDI	Gastroesophageal reflux disease	Male	62.15993745	61.9965664	62.32330851	2019
High-middle SDI	Gastroesophageal reflux disease	Male	62.29849864	61.55149471	63.04550256	2020
High-middle SDI	Gastroesophageal reflux disease	Male	62.43881601	61.11332057	63.76431145	2021
High-middle SDI	Gastroesophageal reflux disease	Male	62.5778797	60.54026473	64.61549467	2022
High-middle SDI	Gastroesophageal reflux disease	Male	62.69274983	59.84116294	65.54433671	2023
High-middle SDI	Gastroesophageal reflux disease	Male	62.80149377	59.04312101	66.55986654	2024
High-middle SDI	Gastroesophageal reflux disease	Male	62.92296239	58.16403332	67.68189147	2025
High-middle SDI	Gastroesophageal reflux disease	Male	63.06460075	57.22289325	68.90630825	2026
High-middle SDI	Gastroesophageal reflux disease	Male	63.201032	56.20466162	70.19740239	2027
High-middle SDI	Gastroesophageal reflux disease	Male	63.31316119	55.09800179	71.5283206	2028
High-middle SDI	Gastroesophageal reflux disease	Male	63.41755361	53.91827386	72.91683337	2029
High-middle SDI	Gastroesophageal reflux disease	Male	63.53009405	52.6751947	74.3849934	2030
High-middle SDI	Gastroesophageal reflux disease	Female	74.12402209	73.91110562	74.33693856	1990
High-middle SDI	Gastroesophageal reflux disease	Female	73.95201033	73.75351374	74.15050693	1991
High-middle SDI	Gastroesophageal reflux disease	Female	73.80176385	73.60505873	73.99846897	1992
High-middle SDI	Gastroesophageal reflux disease	Female	73.64412805	73.44941795	73.83883814	1993
High-middle SDI	Gastroesophageal reflux disease	Female	73.46233743	73.26933105	73.65534381	1994
High-middle SDI	Gastroesophageal reflux disease	Female	73.27878446	73.08733346	73.47023546	1995
High-middle SDI	Gastroesophageal reflux disease	Female	73.15917076	72.9692479	73.34909361	1996
High-middle SDI	Gastroesophageal reflux disease	Female	73.15738694	72.96885183	73.34592204	1997
High-middle SDI	Gastroesophageal reflux disease	Female	73.18992467	73.00269922	73.37715012	1998
High-middle SDI	Gastroesophageal reflux disease	Female	73.17987264	72.99397565	73.36576963	1999
High-middle SDI	Gastroesophageal reflux disease	Female	73.07728187	72.89276735	73.26179638	2000
High-middle SDI	Gastroesophageal reflux disease	Female	72.76487334	72.58211946	72.94762721	2001
High-middle SDI	Gastroesophageal reflux disease	Female	72.284869	72.10411686	72.46562114	2002
High-middle SDI	Gastroesophageal reflux disease	Female	71.71578112	71.53709414	71.89446811	2003
High-middle SDI	Gastroesophageal reflux disease	Female	71.17998883	71.00326183	71.35671584	2004
High-middle SDI	Gastroesophageal reflux disease	Female	70.75439941	70.5793942	70.92940462	2005
High-middle SDI	Gastroesophageal reflux disease	Female	70.37995566	70.2066097	70.55330163	2006
High-middle SDI	Gastroesophageal reflux disease	Female	69.96362266	69.79197768	70.13526763	2007
High-middle SDI	Gastroesophageal reflux disease	Female	69.58168998	69.41162351	69.75175646	2008
High-middle SDI	Gastroesophageal reflux disease	Female	69.29702823	69.12831399	69.46574247	2009
High-middle SDI	Gastroesophageal reflux disease	Female	69.19116719	69.02346612	69.35886826	2010
High-middle SDI	Gastroesophageal reflux disease	Female	69.22517816	69.05827658	69.39207974	2011
High-middle SDI	Gastroesophageal reflux disease	Female	69.31147749	69.14526541	69.47768957	2012
High-middle SDI	Gastroesophageal reflux disease	Female	69.41925002	69.25360882	69.58489122	2013
High-middle SDI	Gastroesophageal reflux disease	Female	69.54416581	69.37893057	69.70940105	2014
High-middle SDI	Gastroesophageal reflux disease	Female	69.72383931	69.55872842	69.88895021	2015
High-middle SDI	Gastroesophageal reflux disease	Female	70.80444808	70.63843761	70.97045855	2016
High-middle SDI	Gastroesophageal reflux disease	Female	71.84452644	71.67716059	72.01189228	2017
High-middle SDI	Gastroesophageal reflux disease	Female	71.92722073	71.76001194	72.09442952	2018
High-middle SDI	Gastroesophageal reflux disease	Female	71.95456089	71.77968639	72.12943539	2019
High-middle SDI	Gastroesophageal reflux disease	Female	72.05479007	71.15495173	72.9546284	2020
High-middle SDI	Gastroesophageal reflux disease	Female	72.19404871	70.56235105	73.82574637	2021
High-middle SDI	Gastroesophageal reflux disease	Female	72.32746087	69.79561283	74.8593089	2022

High-middle SDI	Gastroesophageal reflux disease	Female	72.42896165	68.86888628	75.98903703	2023
High-middle SDI	Gastroesophageal reflux disease	Female	72.52615253	67.82107077	77.2312343	2024
High-middle SDI	Gastroesophageal reflux disease	Female	72.64073393	66.67162232	78.60984554	2025
High-middle SDI	Gastroesophageal reflux disease	Female	72.77782567	65.44065533	80.11499601	2026
High-middle SDI	Gastroesophageal reflux disease	Female	72.90375277	64.10840836	81.69909719	2027
High-middle SDI	Gastroesophageal reflux disease	Female	72.99955076	62.66618528	83.33291624	2028
High-middle SDI	Gastroesophageal reflux disease	Female	73.09012121	61.13701784	85.04322458	2029
High-middle SDI	Gastroesophageal reflux disease	Female	73.19197488	59.52920991	86.85473986	2030
High-middle SDI	Gastroesophageal reflux disease	Both	69.28162141	68.17411359	70.38912923	1990
High-middle SDI	Gastroesophageal reflux disease	Both	69.04807455	67.9566232	70.1395259	1991
High-middle SDI	Gastroesophageal reflux disease	Both	68.84236505	67.76282934	69.92190076	1992
High-middle SDI	Gastroesophageal reflux disease	Both	68.64493972	67.57627498	69.71360446	1993
High-middle SDI	Gastroesophageal reflux disease	Both	68.44873967	67.39061042	69.50686892	1994
High-middle SDI	Gastroesophageal reflux disease	Both	68.27275958	67.22446768	69.32105147	1995
High-middle SDI	Gastroesophageal reflux disease	Both	68.13970321	67.10121499	69.17819142	1996
High-middle SDI	Gastroesophageal reflux disease	Both	68.0698455	67.04038023	69.09931077	1997
High-middle SDI	Gastroesophageal reflux disease	Both	68.01409921	66.99334367	69.03485476	1998
High-middle SDI	Gastroesophageal reflux disease	Both	67.93068744	66.91886584	68.94250903	1999
High-middle SDI	Gastroesophageal reflux disease	Both	67.80231666	66.79935492	68.8052784	2000
High-middle SDI	Gastroesophageal reflux disease	Both	67.56490835	66.57185698	68.55795971	2001
High-middle SDI	Gastroesophageal reflux disease	Both	67.23603235	66.25337452	68.21869017	2002
High-middle SDI	Gastroesophageal reflux disease	Both	66.86480703	65.89242235	67.8371917	2003
High-middle SDI	Gastroesophageal reflux disease	Both	66.51573133	65.55334074	67.47812192	2004
High-middle SDI	Gastroesophageal reflux disease	Both	66.2378932	65.28454019	67.1912462	2005
High-middle SDI	Gastroesophageal reflux disease	Both	65.95402731	65.00999866	66.89805597	2006
High-middle SDI	Gastroesophageal reflux disease	Both	65.59555039	64.66128166	66.52981912	2007
High-middle SDI	Gastroesophageal reflux disease	Both	65.23647698	64.31146378	66.16149017	2008
High-middle SDI	Gastroesophageal reflux disease	Both	64.94590431	64.02919502	65.8626136	2009
High-middle SDI	Gastroesophageal reflux disease	Both	64.81257432	63.90231686	65.72283178	2010
High-middle SDI	Gastroesophageal reflux disease	Both	64.81146987	63.90683474	65.716105	2011
High-middle SDI	Gastroesophageal reflux disease	Both	64.86207972	63.96259778	65.76156166	2012
High-middle SDI	Gastroesophageal reflux disease	Both	64.93564481	64.04063273	65.83065689	2013
High-middle SDI	Gastroesophageal reflux disease	Both	65.02766232	64.13633759	65.91898705	2014
High-middle SDI	Gastroesophageal reflux disease	Both	65.18380183	64.29450506	66.07309859	2015
High-middle SDI	Gastroesophageal reflux disease	Both	66.1282055	65.23604468	67.02036633	2016
High-middle SDI	Gastroesophageal reflux disease	Both	67.04210111	66.14646791	67.93773431	2017
High-middle SDI	Gastroesophageal reflux disease	Both	67.11664381	66.22307749	68.01021014	2018
High-middle SDI	Gastroesophageal reflux disease	Both	67.14428681	66.24978136	68.03879226	2019
High-middle SDI	Gastroesophageal reflux disease	Both	67.35201417	65.64076942	69.06325891	2020
High-middle SDI	Gastroesophageal reflux disease	Both	67.48706035	65.36142355	69.61269716	2021
High-middle SDI	Gastroesophageal reflux disease	Both	67.62197969	64.85740507	70.3865543	2022
High-middle SDI	Gastroesophageal reflux disease	Both	67.73241551	64.15327614	71.31155488	2023
High-middle SDI	Gastroesophageal reflux disease	Both	67.84220359	63.30344987	72.38095731	2024
High-middle SDI	Gastroesophageal reflux disease	Both	67.97345426	62.34205985	73.60484867	2025
High-middle SDI	Gastroesophageal reflux disease	Both	68.13421871	61.3000642	74.96837322	2026
High-middle SDI	Gastroesophageal reflux disease	Both	68.29551947	60.16434707	76.42669187	2027
High-middle SDI	Gastroesophageal reflux disease	Both	68.43864007	58.9259278	77.95135234	2028
High-middle SDI	Gastroesophageal reflux disease	Both	68.58563485	57.60467543	79.56659427	2029
High-middle SDI	Gastroesophageal reflux disease	Both	68.75476528	56.21063901	81.29889155	2030
Middle SDI	Gastroesophageal reflux disease	Male	59.98426536	59.80672934	60.16180137	1990
Middle SDI	Gastroesophageal reflux disease	Male	59.82704553	59.66627071	59.98782035	1991
Middle SDI	Gastroesophageal reflux disease	Male	59.71161604	59.55336152	59.86987057	1992
Middle SDI	Gastroesophageal reflux disease	Male	59.64109009	59.48535016	59.79683002	1993
Middle SDI	Gastroesophageal reflux disease	Male	59.63260547	59.47895189	59.78625904	1994
Middle SDI	Gastroesophageal reflux disease	Male	59.71837921	59.56635131	59.87040712	1995
Middle SDI	Gastroesophageal reflux disease	Male	59.88516268	59.73456172	60.03576363	1996
Middle SDI	Gastroesophageal reflux disease	Male	60.08769218	59.93839718	60.23698717	1997
Middle SDI	Gastroesophageal reflux disease	Male	60.29378988	60.14577327	60.44180649	1998

Middle SDI	Gastroesophageal reflux disease	Male	60.49492041	60.34814964	60.64169119	1999
Middle SDI	Gastroesophageal reflux disease	Male	60.68690005	60.54126961	60.83253048	2000
Middle SDI	Gastroesophageal reflux disease	Male	60.87276794	60.72827589	61.01725999	2001
Middle SDI	Gastroesophageal reflux disease	Male	61.0588763	60.915509	61.20224359	2002
Middle SDI	Gastroesophageal reflux disease	Male	61.2430657	61.10083636	61.38529504	2003
Middle SDI	Gastroesophageal reflux disease	Male	61.42155402	61.2804416	61.56266643	2004
Middle SDI	Gastroesophageal reflux disease	Male	61.58892308	61.44883299	61.72901316	2005
Middle SDI	Gastroesophageal reflux disease	Male	61.64458213	61.50569669	61.78346757	2006
Middle SDI	Gastroesophageal reflux disease	Male	61.5581031	61.42063462	61.69557158	2007
Middle SDI	Gastroesophageal reflux disease	Male	61.44023772	61.30416493	61.57631051	2008
Middle SDI	Gastroesophageal reflux disease	Male	61.39939393	61.26452431	61.53426355	2009
Middle SDI	Gastroesophageal reflux disease	Male	61.51685139	61.38291277	61.65079001	2010
Middle SDI	Gastroesophageal reflux disease	Male	61.75677064	61.62359153	61.88994975	2011
Middle SDI	Gastroesophageal reflux disease	Male	62.04272068	61.91022107	62.17522028	2012
Middle SDI	Gastroesophageal reflux disease	Male	62.34440915	62.21256528	62.47625303	2013
Middle SDI	Gastroesophageal reflux disease	Male	62.68170223	62.55042662	62.81297784	2014
Middle SDI	Gastroesophageal reflux disease	Male	63.05141661	62.92058898	63.18224424	2015
Middle SDI	Gastroesophageal reflux disease	Male	63.78182076	63.65099676	63.91264476	2016
Middle SDI	Gastroesophageal reflux disease	Male	64.46552451	64.33457255	64.59647646	2017
Middle SDI	Gastroesophageal reflux disease	Male	64.72718114	64.59678939	64.8575729	2018
Middle SDI	Gastroesophageal reflux disease	Male	64.96080536	64.82436828	65.09724243	2019
Middle SDI	Gastroesophageal reflux disease	Male	65.25087184	64.55114876	65.95059491	2020
Middle SDI	Gastroesophageal reflux disease	Male	65.5335607	64.28794326	66.77917815	2021
Middle SDI	Gastroesophageal reflux disease	Male	65.80697857	63.88636067	67.72759648	2022
Middle SDI	Gastroesophageal reflux disease	Male	66.06136439	63.36560001	68.75712878	2023
Middle SDI	Gastroesophageal reflux disease	Male	66.31497814	62.75160251	69.87835378	2024
Middle SDI	Gastroesophageal reflux disease	Male	66.57761705	62.05147202	71.10376208	2025
Middle SDI	Gastroesophageal reflux disease	Male	66.84486993	61.27469422	72.41504564	2026
Middle SDI	Gastroesophageal reflux disease	Male	67.10009078	60.41438789	73.78579367	2027
Middle SDI	Gastroesophageal reflux disease	Male	67.33520914	59.46863947	75.20177881	2028
Middle SDI	Gastroesophageal reflux disease	Male	67.56672497	58.45205441	76.68139553	2029
Middle SDI	Gastroesophageal reflux disease	Male	67.80300863	57.36618729	78.23982998	2030
Middle SDI	Gastroesophageal reflux disease	Female	64.45489007	64.27148936	64.63829078	1990
Middle SDI	Gastroesophageal reflux disease	Female	64.43630588	64.26883241	64.60377935	1991
Middle SDI	Gastroesophageal reflux disease	Female	64.44088537	64.27549228	64.60627847	1992
Middle SDI	Gastroesophageal reflux disease	Female	64.46368007	64.30053113	64.62682901	1993
Middle SDI	Gastroesophageal reflux disease	Female	64.52131316	64.36010091	64.68252541	1994
Middle SDI	Gastroesophageal reflux disease	Female	64.63007004	64.47048044	64.78965963	1995
Middle SDI	Gastroesophageal reflux disease	Female	64.76893887	64.61091034	64.9269674	1996
Middle SDI	Gastroesophageal reflux disease	Female	64.91241007	64.75589969	65.06892044	1997
Middle SDI	Gastroesophageal reflux disease	Female	65.04882154	64.89384928	65.2037938	1998
Middle SDI	Gastroesophageal reflux disease	Female	65.18502783	65.03156245	65.3384932	1999
Middle SDI	Gastroesophageal reflux disease	Female	65.34053058	65.188442	65.49261915	2000
Middle SDI	Gastroesophageal reflux disease	Female	65.49579695	65.34508897	65.64650494	2001
Middle SDI	Gastroesophageal reflux disease	Female	65.64285392	65.49351531	65.79219254	2002
Middle SDI	Gastroesophageal reflux disease	Female	65.78502013	65.63706128	65.93297898	2003
Middle SDI	Gastroesophageal reflux disease	Female	65.92850073	65.78187506	66.0751264	2004
Middle SDI	Gastroesophageal reflux disease	Female	66.08660695	65.94119525	66.23201865	2005
Middle SDI	Gastroesophageal reflux disease	Female	66.15923839	66.01518103	66.30329575	2006
Middle SDI	Gastroesophageal reflux disease	Female	66.11020588	65.96766596	66.25274581	2007
Middle SDI	Gastroesophageal reflux disease	Female	66.03011325	65.88907905	66.17114745	2008
Middle SDI	Gastroesophageal reflux disease	Female	66.01456541	65.8748559	66.15427491	2009
Middle SDI	Gastroesophageal reflux disease	Female	66.12518599	65.9865643	66.26380768	2010
Middle SDI	Gastroesophageal reflux disease	Female	66.33775696	66.20008258	66.47543134	2011
Middle SDI	Gastroesophageal reflux disease	Female	66.57993787	66.44314707	66.71672868	2012
Middle SDI	Gastroesophageal reflux disease	Female	66.84431135	66.70835932	66.98026338	2013
Middle SDI	Gastroesophageal reflux disease	Female	67.14434504	67.00911613	67.27957396	2014
Middle SDI	Gastroesophageal reflux disease	Female	67.49077251	67.35612072	67.6254243	2015

Middle SDI	Gastroesophageal reflux disease	Female	68.28043638	68.14584773	68.41502503	2016
Middle SDI	Gastroesophageal reflux disease	Female	69.02633556	68.89166393	69.1610072	2017
Middle SDI	Gastroesophageal reflux disease	Female	69.26980561	69.1357798	69.40383142	2018
Middle SDI	Gastroesophageal reflux disease	Female	69.48512476	69.345594	69.62465552	2019
Middle SDI	Gastroesophageal reflux disease	Female	69.7254586	68.99266696	70.45825025	2020
Middle SDI	Gastroesophageal reflux disease	Female	69.99480544	68.69675419	71.29285669	2021
Middle SDI	Gastroesophageal reflux disease	Female	70.25509617	68.25863996	72.25155239	2022
Middle SDI	Gastroesophageal reflux disease	Female	70.49130065	67.6936333	73.288968	2023
Middle SDI	Gastroesophageal reflux disease	Female	70.72209137	67.0285088	74.41567393	2024
Middle SDI	Gastroesophageal reflux disease	Female	70.96022906	66.27338841	75.64706972	2025
Middle SDI	Gastroesophageal reflux disease	Female	71.20950432	65.44601632	76.97299233	2026
Middle SDI	Gastroesophageal reflux disease	Female	71.44806794	64.53495033	78.36118555	2027
Middle SDI	Gastroesophageal reflux disease	Female	71.66256743	63.53372919	79.79140567	2028
Middle SDI	Gastroesophageal reflux disease	Female	71.8689082	62.45665146	81.28116494	2029
Middle SDI	Gastroesophageal reflux disease	Female	72.07737075	61.30709017	82.84765134	2030
Middle SDI	Gastroesophageal reflux disease	Both	62.17462254	61.19241415	63.15683092	1990
Middle SDI	Gastroesophageal reflux disease	Both	62.08661183	61.12215	63.05107367	1991
Middle SDI	Gastroesophageal reflux disease	Both	62.03114188	61.08030985	62.98197391	1992
Middle SDI	Gastroesophageal reflux disease	Both	62.00757489	61.06948758	62.94566221	1993
Middle SDI	Gastroesophageal reflux disease	Both	62.03275462	61.10671161	62.95879763	1994
Middle SDI	Gastroesophageal reflux disease	Both	62.13158362	61.21647157	63.04669567	1995
Middle SDI	Gastroesophageal reflux disease	Both	62.28659728	61.38151045	63.19168411	1996
Middle SDI	Gastroesophageal reflux disease	Both	62.46204629	61.56646147	63.35763112	1997
Middle SDI	Gastroesophageal reflux disease	Both	62.63590755	61.74983614	63.52197895	1998
Middle SDI	Gastroesophageal reflux disease	Both	62.807092	61.93057355	63.68361045	1999
Middle SDI	Gastroesophageal reflux disease	Both	62.98363765	62.11621189	63.85106341	2000
Middle SDI	Gastroesophageal reflux disease	Both	63.15706689	62.29850022	64.01563356	2001
Middle SDI	Gastroesophageal reflux disease	Both	63.32626378	62.47638519	64.17614237	2002
Middle SDI	Gastroesophageal reflux disease	Both	63.49184725	62.65061641	64.3330781	2003
Middle SDI	Gastroesophageal reflux disease	Both	63.65497876	62.82226373	64.48769378	2004
Middle SDI	Gastroesophageal reflux disease	Both	63.82001345	62.99530737	64.64471952	2005
Middle SDI	Gastroesophageal reflux disease	Both	63.88635951	63.07046208	64.70225693	2006
Middle SDI	Gastroesophageal reflux disease	Both	63.82058985	63.01450412	64.62667558	2007
Middle SDI	Gastroesophageal reflux disease	Both	63.72385318	62.92743239	64.52027397	2008
Middle SDI	Gastroesophageal reflux disease	Both	63.69796799	62.91017594	64.48576004	2009
Middle SDI	Gastroesophageal reflux disease	Both	63.81421662	63.0334974	64.59493584	2010
Middle SDI	Gastroesophageal reflux disease	Both	64.04248251	63.26797563	64.81698939	2011
Middle SDI	Gastroesophageal reflux disease	Both	64.30828722	63.53960956	65.07696488	2012
Middle SDI	Gastroesophageal reflux disease	Both	64.59278425	63.82970753	65.35586096	2013
Middle SDI	Gastroesophageal reflux disease	Both	64.91273196	64.15467621	65.6707877	2014
Middle SDI	Gastroesophageal reflux disease	Both	65.27200131	64.51798299	66.02601963	2015
Middle SDI	Gastroesophageal reflux disease	Both	66.03331729	65.28111803	66.78551654	2016
Middle SDI	Gastroesophageal reflux disease	Both	66.7492057	65.99897432	67.49943709	2017
Middle SDI	Gastroesophageal reflux disease	Both	67.00233801	66.25660312	67.74807289	2018
Middle SDI	Gastroesophageal reflux disease	Both	67.22725807	66.48393926	67.97057688	2019
Middle SDI	Gastroesophageal reflux disease	Both	67.48812516	66.14004801	68.83620232	2020
Middle SDI	Gastroesophageal reflux disease	Both	67.76686645	66.04421718	69.48951573	2021
Middle SDI	Gastroesophageal reflux disease	Both	68.03826215	65.75153303	70.32499127	2022
Middle SDI	Gastroesophageal reflux disease	Both	68.29085354	65.29295931	71.28874776	2023
Middle SDI	Gastroesophageal reflux disease	Both	68.54390708	64.71156917	72.37624499	2024
Middle SDI	Gastroesophageal reflux disease	Both	68.80958643	64.02682677	73.59234609	2025
Middle SDI	Gastroesophageal reflux disease	Both	69.08822324	63.26074525	74.91570123	2026
Middle SDI	Gastroesophageal reflux disease	Both	69.36135076	62.40754206	76.31515946	2027
Middle SDI	Gastroesophageal reflux disease	Both	69.61912478	61.46412293	77.77412663	2028
Middle SDI	Gastroesophageal reflux disease	Both	69.87873527	60.445118	79.31235255	2029
Middle SDI	Gastroesophageal reflux disease	Both	70.15074978	59.3532494	80.94825017	2030
Low-middle SDI	Gastroesophageal reflux disease	Male	89.36394278	89.08520721	89.64267834	1990
Low-middle SDI	Gastroesophageal reflux disease	Male	89.3036682	89.05208835	89.55524804	1991

Low-middle SDI	Gastroesophageal reflux disease	Male	89.24486145	88.99678301	89.49293989	1992
Low-middle SDI	Gastroesophageal reflux disease	Male	89.18722615	88.94251174	89.43194056	1993
Low-middle SDI	Gastroesophageal reflux disease	Male	89.13250535	88.89079159	89.37421912	1994
Low-middle SDI	Gastroesophageal reflux disease	Male	89.10449857	88.86530393	89.34369321	1995
Low-middle SDI	Gastroesophageal reflux disease	Male	89.15534294	88.91845656	89.39222932	1996
Low-middle SDI	Gastroesophageal reflux disease	Male	89.3091134	89.07429099	89.54393581	1997
Low-middle SDI	Gastroesophageal reflux disease	Male	89.5005055	89.26759116	89.73341984	1998
Low-middle SDI	Gastroesophageal reflux disease	Male	89.6585843	89.42756993	89.88959868	1999
Low-middle SDI	Gastroesophageal reflux disease	Male	89.73466565	89.5056133	89.963718	2000
Low-middle SDI	Gastroesophageal reflux disease	Male	89.69917185	89.47231188	89.92603181	2001
Low-middle SDI	Gastroesophageal reflux disease	Male	89.61861857	89.39401131	89.84322583	2002
Low-middle SDI	Gastroesophageal reflux disease	Male	89.52130296	89.29894707	89.74365884	2003
Low-middle SDI	Gastroesophageal reflux disease	Male	89.45302384	89.23287089	89.67317678	2004
Low-middle SDI	Gastroesophageal reflux disease	Male	89.45695294	89.23887802	89.67502786	2005
Low-middle SDI	Gastroesophageal reflux disease	Male	89.46101944	89.24506158	89.6769773	2006
Low-middle SDI	Gastroesophageal reflux disease	Male	89.43648458	89.22267991	89.65028925	2007
Low-middle SDI	Gastroesophageal reflux disease	Male	89.39279633	89.1811501	89.60444255	2008
Low-middle SDI	Gastroesophageal reflux disease	Male	89.37476871	89.16522081	89.58431661	2009
Low-middle SDI	Gastroesophageal reflux disease	Male	89.40577485	89.19823103	89.61331867	2010
Low-middle SDI	Gastroesophageal reflux disease	Male	89.44586726	89.24030359	89.65143094	2011
Low-middle SDI	Gastroesophageal reflux disease	Male	89.47208972	89.26851256	89.67566687	2012
Low-middle SDI	Gastroesophageal reflux disease	Male	89.50138654	89.29977616	89.70299693	2013
Low-middle SDI	Gastroesophageal reflux disease	Male	89.53550069	89.33582051	89.73518087	2014
Low-middle SDI	Gastroesophageal reflux disease	Male	89.59696565	89.39912602	89.79480529	2015
Low-middle SDI	Gastroesophageal reflux disease	Male	89.74897928	89.55272994	89.94522863	2016
Low-middle SDI	Gastroesophageal reflux disease	Male	89.87235454	89.67751684	90.06719224	2017
Low-middle SDI	Gastroesophageal reflux disease	Male	89.92329966	89.73017635	90.11642298	2018
Low-middle SDI	Gastroesophageal reflux disease	Male	89.9791541	89.77676475	90.18154346	2019
Low-middle SDI	Gastroesophageal reflux disease	Male	90.02817065	89.1098839	90.94645739	2020
Low-middle SDI	Gastroesophageal reflux disease	Male	90.08275146	88.50777307	91.65772985	2021
Low-middle SDI	Gastroesophageal reflux disease	Male	90.1369783	87.74824095	92.52571566	2022
Low-middle SDI	Gastroesophageal reflux disease	Male	90.18799919	86.86408651	93.51191188	2023
Low-middle SDI	Gastroesophageal reflux disease	Male	90.23712831	85.8712265	94.60303013	2024
Low-middle SDI	Gastroesophageal reflux disease	Male	90.28672041	84.778344	95.79509682	2025
Low-middle SDI	Gastroesophageal reflux disease	Male	90.3401867	83.60075543	97.07961797	2026
Low-middle SDI	Gastroesophageal reflux disease	Male	90.39347527	82.34273927	98.44421127	2027
Low-middle SDI	Gastroesophageal reflux disease	Male	90.44296659	81.00616866	99.87976453	2028
Low-middle SDI	Gastroesophageal reflux disease	Male	90.48990032	79.59416726	101.3856334	2029
Low-middle SDI	Gastroesophageal reflux disease	Male	90.53698531	78.10881741	102.9651532	2030
Low-middle SDI	Gastroesophageal reflux disease	Female	90.31848468	90.03436302	90.60260635	1990
Low-middle SDI	Gastroesophageal reflux disease	Female	90.31483301	90.05668327	90.57298275	1991
Low-middle SDI	Gastroesophageal reflux disease	Female	90.31207951	90.05715961	90.5669994	1992
Low-middle SDI	Gastroesophageal reflux disease	Female	90.30952431	90.05791314	90.56113547	1993
Low-middle SDI	Gastroesophageal reflux disease	Female	90.31755432	90.06894218	90.56616645	1994
Low-middle SDI	Gastroesophageal reflux disease	Female	90.35044935	90.10443426	90.59646443	1995
Low-middle SDI	Gastroesophageal reflux disease	Female	90.54158817	90.29790868	90.78526765	1996
Low-middle SDI	Gastroesophageal reflux disease	Female	90.93030933	90.68864263	91.17197603	1997
Low-middle SDI	Gastroesophageal reflux disease	Female	91.38281899	91.142984	91.62265398	1998
Low-middle SDI	Gastroesophageal reflux disease	Female	91.78517891	91.54717759	92.02318022	1999
Low-middle SDI	Gastroesophageal reflux disease	Female	91.98722437	91.75132001	92.22312873	2000
Low-middle SDI	Gastroesophageal reflux disease	Female	91.92532308	91.69202558	92.15862057	2001
Low-middle SDI	Gastroesophageal reflux disease	Female	91.69110716	91.46064833	91.92156598	2002
Low-middle SDI	Gastroesophageal reflux disease	Female	91.40269101	91.17507214	91.63030988	2003
Low-middle SDI	Gastroesophageal reflux disease	Female	91.1822733	90.95736281	91.4071838	2004
Low-middle SDI	Gastroesophageal reflux disease	Female	91.1237131	90.90126803	91.34615818	2005
Low-middle SDI	Gastroesophageal reflux disease	Female	91.17126839	90.95120076	91.39133601	2006
Low-middle SDI	Gastroesophageal reflux disease	Female	91.19699451	90.97932054	91.41466849	2007
Low-middle SDI	Gastroesophageal reflux disease	Female	91.21166377	90.99638313	91.42694442	2008

Low-middle SDI	Gastroesophageal reflux disease	Female	91.2259519	91.01304023	91.43886357	2009
Low-middle SDI	Gastroesophageal reflux disease	Female	91.29667996	91.08602367	91.50733626	2010
Low-middle SDI	Gastroesophageal reflux disease	Female	91.31365367	91.10528976	91.52201758	2011
Low-middle SDI	Gastroesophageal reflux disease	Female	91.2551352	91.04912361	91.46114678	2012
Low-middle SDI	Gastroesophageal reflux disease	Female	91.17220157	90.96853586	91.37586727	2013
Low-middle SDI	Gastroesophageal reflux disease	Female	91.12052562	90.91910755	91.3219437	2014
Low-middle SDI	Gastroesophageal reflux disease	Female	91.17016012	90.97079555	91.36952469	2015
Low-middle SDI	Gastroesophageal reflux disease	Female	91.56363975	91.36581997	91.76145952	2016
Low-middle SDI	Gastroesophageal reflux disease	Female	91.95546346	91.75891806	92.15200887	2017
Low-middle SDI	Gastroesophageal reflux disease	Female	92.03359095	91.83892191	92.22825999	2018
Low-middle SDI	Gastroesophageal reflux disease	Female	92.10716588	91.9048452	92.30948657	2019
Low-middle SDI	Gastroesophageal reflux disease	Female	92.14943069	91.1552971	93.14356427	2020
Low-middle SDI	Gastroesophageal reflux disease	Female	92.24383268	90.52047441	93.96719095	2021
Low-middle SDI	Gastroesophageal reflux disease	Female	92.33664598	89.70808047	94.96521148	2022
Low-middle SDI	Gastroesophageal reflux disease	Female	92.41958256	88.74979161	96.08937352	2023
Low-middle SDI	Gastroesophageal reflux disease	Female	92.49729343	87.66688514	97.32770173	2024
Low-middle SDI	Gastroesophageal reflux disease	Female	92.57693078	86.47383096	98.6800306	2025
Low-middle SDI	Gastroesophageal reflux disease	Female	92.66564684	85.18975411	100.1415396	2026
Low-middle SDI	Gastroesophageal reflux disease	Female	92.7533472	83.81376575	101.6929287	2027
Low-middle SDI	Gastroesophageal reflux disease	Female	92.83105611	82.34359355	103.3185187	2028
Low-middle SDI	Gastroesophageal reflux disease	Female	92.90310415	80.78589382	105.0203145	2029
Low-middle SDI	Gastroesophageal reflux disease	Female	92.97648646	79.1469762	106.8059967	2030
Low-middle SDI	Gastroesophageal reflux disease	Both	89.82958004	88.29123303	91.36792705	1990
Low-middle SDI	Gastroesophageal reflux disease	Both	89.79715575	88.28470537	91.30960613	1991
Low-middle SDI	Gastroesophageal reflux disease	Both	89.76629712	88.27418487	91.25840936	1992
Low-middle SDI	Gastroesophageal reflux disease	Both	89.73620118	88.26336236	91.20904	1993
Low-middle SDI	Gastroesophageal reflux disease	Both	89.71294078	88.25864577	91.1672358	1994
Low-middle SDI	Gastroesophageal reflux disease	Both	89.71558033	88.27858577	91.1525749	1995
Low-middle SDI	Gastroesophageal reflux disease	Both	89.83644917	88.41550301	91.25739533	1996
Low-middle SDI	Gastroesophageal reflux disease	Both	90.10742326	88.70100829	91.51383823	1997
Low-middle SDI	Gastroesophageal reflux disease	Both	90.42929216	89.03641509	91.82216922	1998
Low-middle SDI	Gastroesophageal reflux disease	Both	90.7097912	89.33060041	92.08898198	1999
Low-middle SDI	Gastroesophageal reflux disease	Both	90.84946501	89.48481823	92.2141118	2000
Low-middle SDI	Gastroesophageal reflux disease	Both	90.80148141	89.45301431	92.14994852	2001
Low-middle SDI	Gastroesophageal reflux disease	Both	90.64486116	89.3132214	91.97650093	2002
Low-middle SDI	Gastroesophageal reflux disease	Both	90.45238699	89.13780909	91.76696489	2003
Low-middle SDI	Gastroesophageal reflux disease	Both	90.30808091	89.01043039	91.60573143	2004
Low-middle SDI	Gastroesophageal reflux disease	Both	90.28081494	88.99904541	91.56258447	2005
Low-middle SDI	Gastroesophageal reflux disease	Both	90.30676485	89.04046475	91.57306495	2006
Low-middle SDI	Gastroesophageal reflux disease	Both	90.30764528	89.05685048	91.55844008	2007
Low-middle SDI	Gastroesophageal reflux disease	Both	90.2937184	89.05833541	91.52910139	2008
Low-middle SDI	Gastroesophageal reflux disease	Both	90.29247214	89.07235329	91.51259099	2009
Low-middle SDI	Gastroesophageal reflux disease	Both	90.34383223	89.1385757	91.54908876	2010
Low-middle SDI	Gastroesophageal reflux disease	Both	90.37287344	89.18267828	91.56306859	2011
Low-middle SDI	Gastroesophageal reflux disease	Both	90.35705258	89.18207638	91.53202878	2012
Low-middle SDI	Gastroesophageal reflux disease	Both	90.33046287	89.17025634	91.49066939	2013
Low-middle SDI	Gastroesophageal reflux disease	Both	90.32183382	89.1754756	91.46819204	2014
Low-middle SDI	Gastroesophageal reflux disease	Both	90.37768854	89.24414233	91.51123474	2015
Low-middle SDI	Gastroesophageal reflux disease	Both	90.65165219	89.52954226	91.77376213	2016
Low-middle SDI	Gastroesophageal reflux disease	Both	90.91068049	89.79948897	92.021872	2017
Low-middle SDI	Gastroesophageal reflux disease	Both	90.97604795	89.8764262	92.07566971	2018
Low-middle SDI	Gastroesophageal reflux disease	Both	91.04142183	89.95069586	92.1321478	2019
Low-middle SDI	Gastroesophageal reflux disease	Both	91.08606197	89.20947711	92.96264684	2020
Low-middle SDI	Gastroesophageal reflux disease	Both	91.1633522	88.84402394	93.48268045	2021
Low-middle SDI	Gastroesophageal reflux disease	Both	91.2421443	88.23927806	94.24501054	2022
Low-middle SDI	Gastroesophageal reflux disease	Both	91.31773406	87.44137123	95.19409688	2023
Low-middle SDI	Gastroesophageal reflux disease	Both	91.39402177	86.49095793	96.29708561	2024
Low-middle SDI	Gastroesophageal reflux disease	Both	91.47676537	85.41417213	97.53935862	2025

Low-middle SDI	Gastroesophageal reflux disease	Both	91.57207618	84.23785503	98.90629733	2026
Low-middle SDI	Gastroesophageal reflux disease	Both	91.6737229	82.96895826	100.3784876	2027
Low-middle SDI	Gastroesophageal reflux disease	Both	91.7763066	81.61031845	101.9422948	2028
Low-middle SDI	Gastroesophageal reflux disease	Both	91.8836009	80.16827763	103.5989242	2029
Low-middle SDI	Gastroesophageal reflux disease	Both	92.00154051	78.647781	105.3553	2030
Low SDI	Gastroesophageal reflux disease	Male	90.81740234	90.39255844	91.24224624	1990
Low SDI	Gastroesophageal reflux disease	Male	90.83153558	90.46669801	91.19637316	1991
Low SDI	Gastroesophageal reflux disease	Male	90.85170018	90.49360907	91.2097913	1992
Low SDI	Gastroesophageal reflux disease	Male	90.85939673	90.50600251	91.21279095	1993
Low SDI	Gastroesophageal reflux disease	Male	90.85699024	90.50824015	91.20574034	1994
Low SDI	Gastroesophageal reflux disease	Male	90.83193405	90.48717919	91.17668891	1995
Low SDI	Gastroesophageal reflux disease	Male	90.80768217	90.46664205	91.1487223	1996
Low SDI	Gastroesophageal reflux disease	Male	90.7806036	90.44300297	91.11820424	1997
Low SDI	Gastroesophageal reflux disease	Male	90.74766661	90.41329012	91.08204309	1998
Low SDI	Gastroesophageal reflux disease	Male	90.72830776	90.39701514	91.05960039	1999
Low SDI	Gastroesophageal reflux disease	Male	90.71119613	90.38294998	91.03944228	2000
Low SDI	Gastroesophageal reflux disease	Male	90.74218043	90.41700924	91.06735162	2001
Low SDI	Gastroesophageal reflux disease	Male	90.80640238	90.48428384	91.12852092	2002
Low SDI	Gastroesophageal reflux disease	Male	90.89286086	90.57369185	91.21202987	2003
Low SDI	Gastroesophageal reflux disease	Male	90.97698635	90.66075761	91.29321508	2004
Low SDI	Gastroesophageal reflux disease	Male	91.01735573	90.70417005	91.33054142	2005
Low SDI	Gastroesophageal reflux disease	Male	91.02794577	90.71791737	91.33797418	2006
Low SDI	Gastroesophageal reflux disease	Male	91.03766166	90.73081597	91.34450734	2007
Low SDI	Gastroesophageal reflux disease	Male	91.04217116	90.73852841	91.34581391	2008
Low SDI	Gastroesophageal reflux disease	Male	91.0508171	90.75036107	91.35127314	2009
Low SDI	Gastroesophageal reflux disease	Male	91.05103442	90.75375032	91.34831852	2010
Low SDI	Gastroesophageal reflux disease	Male	91.05287205	90.7587835	91.34696059	2011
Low SDI	Gastroesophageal reflux disease	Male	91.05026714	90.75939226	91.34114202	2012
Low SDI	Gastroesophageal reflux disease	Male	91.05736557	90.76964692	91.34508423	2013
Low SDI	Gastroesophageal reflux disease	Male	91.04953528	90.76493471	91.33413585	2014
Low SDI	Gastroesophageal reflux disease	Male	91.03060044	90.74902159	91.31217929	2015
Low SDI	Gastroesophageal reflux disease	Male	90.8911954	90.61261353	91.16977728	2016
Low SDI	Gastroesophageal reflux disease	Male	90.75147959	90.47578392	91.02717525	2017
Low SDI	Gastroesophageal reflux disease	Male	90.7004107	90.42749689	90.97332451	2018
Low SDI	Gastroesophageal reflux disease	Male	90.66527895	90.37022761	90.9603303	2019
Low SDI	Gastroesophageal reflux disease	Male	90.61921313	89.60903797	91.62938828	2020
Low SDI	Gastroesophageal reflux disease	Male	90.56382582	88.86871266	92.25893899	2021
Low SDI	Gastroesophageal reflux disease	Male	90.50861224	87.96837551	93.04884896	2022
Low SDI	Gastroesophageal reflux disease	Male	90.45451842	86.94550536	93.96353147	2023
Low SDI	Gastroesophageal reflux disease	Male	90.4003304	85.81498905	94.98567174	2024
Low SDI	Gastroesophageal reflux disease	Male	90.34436383	84.58367173	96.10505593	2025
Low SDI	Gastroesophageal reflux disease	Male	90.28737468	83.26407506	97.31067429	2026
Low SDI	Gastroesophageal reflux disease	Male	90.23088304	81.865673	98.59609307	2027
Low SDI	Gastroesophageal reflux disease	Male	90.17542093	80.39414371	99.95669815	2028
Low SDI	Gastroesophageal reflux disease	Male	90.11985577	78.85094161	101.3887699	2029
Low SDI	Gastroesophageal reflux disease	Male	90.06281068	77.23583498	102.8897864	2030
Low SDI	Gastroesophageal reflux disease	Female	93.55717238	93.12561088	93.98873388	1990
Low SDI	Gastroesophageal reflux disease	Female	93.58248231	93.21159373	93.9533709	1991
Low SDI	Gastroesophageal reflux disease	Female	93.61595037	93.2512463	93.98065444	1992
Low SDI	Gastroesophageal reflux disease	Female	93.64477136	93.28440669	94.00513602	1993
Low SDI	Gastroesophageal reflux disease	Female	93.65230553	93.29647048	94.00814057	1994
Low SDI	Gastroesophageal reflux disease	Female	93.64786965	93.29601637	93.99972292	1995
Low SDI	Gastroesophageal reflux disease	Female	93.64295774	93.29486916	93.99104633	1996
Low SDI	Gastroesophageal reflux disease	Female	93.64169388	93.29713067	93.98625709	1997
Low SDI	Gastroesophageal reflux disease	Female	93.64647173	93.30522379	93.98771967	1998
Low SDI	Gastroesophageal reflux disease	Female	93.65435776	93.31634601	93.99236952	1999
Low SDI	Gastroesophageal reflux disease	Female	93.67336787	93.33856287	94.00817287	2000
Low SDI	Gastroesophageal reflux disease	Female	93.69463457	93.36315223	94.02611692	2001

Low SDI	Gastroesophageal reflux disease	Female	93.73789078	93.40970729	94.06607427	2002
Low SDI	Gastroesophageal reflux disease	Female	93.77869868	93.45377525	94.10362212	2003
Low SDI	Gastroesophageal reflux disease	Female	93.81725468	93.49557988	94.13892949	2004
Low SDI	Gastroesophageal reflux disease	Female	93.84901611	93.53061889	94.16741332	2005
Low SDI	Gastroesophageal reflux disease	Female	93.8719022	93.55685847	94.18694594	2006
Low SDI	Gastroesophageal reflux disease	Female	93.90504437	93.59336558	94.21672315	2007
Low SDI	Gastroesophageal reflux disease	Female	93.92556253	93.61729842	94.23382664	2008
Low SDI	Gastroesophageal reflux disease	Female	93.94437882	93.63952315	94.24923449	2009
Low SDI	Gastroesophageal reflux disease	Female	93.95445258	93.6530078	94.25589736	2010
Low SDI	Gastroesophageal reflux disease	Female	93.96524287	93.66722936	94.26325638	2011
Low SDI	Gastroesophageal reflux disease	Female	93.97282743	93.67825316	94.2674017	2012
Low SDI	Gastroesophageal reflux disease	Female	93.98610931	93.6949285	94.27729012	2013
Low SDI	Gastroesophageal reflux disease	Female	93.98937928	93.70153623	94.27722233	2014
Low SDI	Gastroesophageal reflux disease	Female	93.98634008	93.70172707	94.27095309	2015
Low SDI	Gastroesophageal reflux disease	Female	93.94715282	93.66558233	94.22872331	2016
Low SDI	Gastroesophageal reflux disease	Female	93.90884694	93.6301811	94.18751278	2017
Low SDI	Gastroesophageal reflux disease	Female	93.8884031	93.61271854	94.16408766	2018
Low SDI	Gastroesophageal reflux disease	Female	93.86486839	93.56774006	94.16199672	2019
Low SDI	Gastroesophageal reflux disease	Female	93.83875055	92.80070421	94.87679688	2020
Low SDI	Gastroesophageal reflux disease	Female	93.81251823	92.06532051	95.55971594	2021
Low SDI	Gastroesophageal reflux disease	Female	93.78603571	91.16319055	96.40888088	2022
Low SDI	Gastroesophageal reflux disease	Female	93.75840949	90.13119687	97.38562211	2023
Low SDI	Gastroesophageal reflux disease	Female	93.72912856	88.98545723	98.4727999	2024
Low SDI	Gastroesophageal reflux disease	Female	93.69858039	87.73502058	99.6621402	2025
Low SDI	Gastroesophageal reflux disease	Female	93.6677642	86.39292572	100.9426027	2026
Low SDI	Gastroesophageal reflux disease	Female	93.63671235	84.96747927	102.3059454	2027
Low SDI	Gastroesophageal reflux disease	Female	93.6042651	83.46303419	103.745496	2028
Low SDI	Gastroesophageal reflux disease	Female	93.56987894	81.88174548	105.2580124	2029
Low SDI	Gastroesophageal reflux disease	Female	93.53404948	80.22506362	106.8430353	2030
Low SDI	Gastroesophageal reflux disease	Both	92.19313598	89.8508741	94.53539786	1990
Low SDI	Gastroesophageal reflux disease	Both	92.21214708	89.91098251	94.51331164	1991
Low SDI	Gastroesophageal reflux disease	Both	92.2381789	89.96620661	94.51015119	1992
Low SDI	Gastroesophageal reflux disease	Both	92.25565051	90.01087269	94.50042832	1993
Low SDI	Gastroesophageal reflux disease	Both	92.25832143	90.04058856	94.4760543	1994
Low SDI	Gastroesophageal reflux disease	Both	92.24405187	90.05315177	94.43495197	1995
Low SDI	Gastroesophageal reflux disease	Both	92.22997609	90.06604984	94.39390234	1996
Low SDI	Gastroesophageal reflux disease	Both	92.21648283	90.0784398	94.35452585	1997
Low SDI	Gastroesophageal reflux disease	Both	92.20301456	90.08955797	94.31647115	1998
Low SDI	Gastroesophageal reflux disease	Both	92.19812773	90.10883232	94.28742315	1999
Low SDI	Gastroesophageal reflux disease	Both	92.19985805	90.13449201	94.26522409	2000
Low SDI	Gastroesophageal reflux disease	Both	92.22642241	90.18562404	94.26722078	2001
Low SDI	Gastroesophageal reflux disease	Both	92.28024175	90.26396396	94.29651955	2002
Low SDI	Gastroesophageal reflux disease	Both	92.34416395	90.35187602	94.33645187	2003
Low SDI	Gastroesophageal reflux disease	Both	92.40579482	90.43755667	94.37403297	2004
Low SDI	Gastroesophageal reflux disease	Both	92.44237043	90.49849784	94.38624301	2005
Low SDI	Gastroesophageal reflux disease	Both	92.45959805	90.5406822	94.37851389	2006
Low SDI	Gastroesophageal reflux disease	Both	92.48161709	90.58780693	94.37542725	2007
Low SDI	Gastroesophageal reflux disease	Both	92.49487628	90.62617588	94.36357667	2008
Low SDI	Gastroesophageal reflux disease	Both	92.50933962	90.66571769	94.35296156	2009
Low SDI	Gastroesophageal reflux disease	Both	92.51536142	90.69685328	94.33386957	2010
Low SDI	Gastroesophageal reflux disease	Both	92.52247969	90.72946834	94.31549104	2011
Low SDI	Gastroesophageal reflux disease	Both	92.52580814	90.75832401	94.29329227	2012
Low SDI	Gastroesophageal reflux disease	Both	92.53686875	90.7942407	94.27949681	2013
Low SDI	Gastroesophageal reflux disease	Both	92.53543963	90.81713339	94.25374587	2014
Low SDI	Gastroesophageal reflux disease	Both	92.52527723	90.83096914	94.21958531	2015
Low SDI	Gastroesophageal reflux disease	Both	92.43698146	90.76776378	94.10619913	2016
Low SDI	Gastroesophageal reflux disease	Both	92.34886107	90.70451196	93.99321018	2017
Low SDI	Gastroesophageal reflux disease	Both	92.31366815	90.69268909	93.9346472	2018

Low SDI	Gastroesophageal reflux disease	Both	92.2848159	90.68132791	93.88830388	2019
Low SDI	Gastroesophageal reflux disease	Both	92.26084335	89.91455372	94.60713298	2020
Low SDI	Gastroesophageal reflux disease	Both	92.22357498	89.48716279	94.95998716	2021
Low SDI	Gastroesophageal reflux disease	Both	92.18873652	88.82691258	95.55056046	2022
Low SDI	Gastroesophageal reflux disease	Both	92.15708644	87.97457252	96.33960036	2023
Low SDI	Gastroesophageal reflux disease	Both	92.12859717	86.96581813	97.29137621	2024
Low SDI	Gastroesophageal reflux disease	Both	92.10363324	85.82375183	98.38351465	2025
Low SDI	Gastroesophageal reflux disease	Both	92.08393652	84.57231971	99.59555333	2026
Low SDI	Gastroesophageal reflux disease	Both	92.07110454	83.22737781	100.9148313	2027
Low SDI	Gastroesophageal reflux disease	Both	92.06557273	81.79896302	102.3321824	2028
Low SDI	Gastroesophageal reflux disease	Both	92.06737873	80.29143789	103.8433196	2029
Low SDI	Gastroesophageal reflux disease	Both	92.07716064	78.70649052	105.4478308	2030

egorized by global and SDI regions.

Val	Upper	Lower
245.3650105	269.5075572	218.6067307
241.1818778	265.7180956	218.1230315
237.3549304	259.5675619	214.2451065
233.2884475	255.2184602	210.6874417
228.0632124	247.0806161	205.2544313
220.2248942	239.874491	199.2227383
211.9668455	230.0437873	192.7402276
205.3270249	221.8729415	187.1263816
197.4497598	212.7014663	181.5888527
189.9023649	204.357962	173.5691722
183.1348633	196.6405159	167.6900253
175.5938429	187.8712032	161.5930583
168.7652775	182.6131982	156.2846474
161.4378078	174.1997593	150.0450295
153.5891191	168.2924526	143.3244164
148.670418	163.4104965	138.9734556
141.0190409	154.9414684	131.4746667
134.7756138	149.3933917	126.1812746
130.4670266	145.1856106	121.8649784
123.9433863	137.6730469	116.0613062
118.7162238	131.7034594	111.4714702
113.8447897	127.3044088	106.8484994
109.3897571	123.8095292	102.8630746
105.9942668	119.8672649	99.76174793
101.7357663	112.5501951	95.92690249
98.93431088	107.6757973	92.84536523
96.69081601	106.570343	89.86515737
94.05318897	102.5030576	87.19194111
91.35911111	103.3625247	84.26497252
88.92818348	99.59185663	80.87013697
NA	NA	NA
NA	NA	NA
NA	NA	NA
NA	NA	NA
NA	NA	NA
NA	NA	NA
NA	NA	NA
NA	NA	NA
NA	NA	NA
NA	NA	NA
NA	NA	NA
137.2969747	159.2227764	118.8760902
134.7272974	157.3035762	117.548522
133.9669536	155.0475253	115.5980766
130.857538	149.1636215	115.0634363
127.9292534	146.7989248	113.2315577
124.2938609	141.2771256	110.2289281
121.063323	137.0952394	107.5670784
119.9437004	137.3065573	106.1607617
116.441977	132.7467359	103.4817823
113.0735779	127.6776559	100.4594161
110.2285912	124.4962835	98.27635638
107.0628959	120.5298745	95.66087661
103.8688524	116.6775859	93.39489347
100.2596068	112.7077979	90.5806437
95.79525971	105.9130108	87.31605554

93.41454698	103.9214322	85.09908479
89.60988366	99.16755587	81.37044897
85.61861575	94.20559272	77.81180624
82.44999094	90.71990299	75.60832944
78.27977857	85.80971557	71.63686433
75.79456485	82.67612233	69.30438526
73.25943869	79.29411682	66.86646967
70.34711197	76.5885526	64.44496896
68.76433074	74.73274193	62.56014346
66.52865437	72.65433751	60.51659385
65.38465018	70.65438489	59.64522932
64.38579443	70.20143597	58.4248994
63.51278369	69.47144806	57.40915546
62.13257054	68.48216919	56.02215036
60.83366873	67.77459294	54.42405974
NA	NA	NA
NA	NA	NA
NA	NA	NA
NA	NA	NA
NA	NA	NA
NA	NA	NA
NA	NA	NA
NA	NA	NA
NA	NA	NA
NA	NA	NA
NA	NA	NA
189.0304085	205.6325112	175.5188963
185.7547691	202.0873439	171.9573892
183.5681178	198.2282289	171.0209841
180.0811689	193.593124	168.0389671
176.0773832	188.5167465	163.939603
170.4106294	182.2759354	159.4702218
164.7383198	176.0452247	153.9450647
160.9444237	172.088472	150.2127228
155.3383478	165.9413704	145.4764014
150.0267654	160.4796607	140.6937304
145.3053216	155.6851479	136.4705283
140.0309758	150.2710993	131.3179222
135.1034084	144.9327907	126.9473234
129.7002059	139.4754701	122.0858462
123.6172198	133.4007788	116.4388064
120.0268651	129.4000751	112.8110118
114.4004008	124.2339246	107.49975
109.3607264	118.5522679	102.7488854
105.6527743	114.472423	99.03085976
100.308225	109.328918	94.28590265
96.47333033	104.9703161	90.63377573
92.81348515	101.9364101	87.06248891
89.1555439	97.22788081	83.90474497
86.66670356	95.16146219	81.30920801
83.45080212	90.59938654	78.54255841
81.5294482	88.80929536	76.74638709
79.94539768	86.86071812	74.70390291
78.23279969	84.84852106	72.7086338
76.23369593	83.40459224	70.53497708
74.39955408	81.9455989	68.95514969
NA	NA	NA
NA	NA	NA

NA	NA	NA
NA	NA	NA
NA	NA	NA
NA	NA	NA
NA	NA	NA
NA	NA	NA
NA	NA	NA
NA	NA	NA
NA	NA	NA
99.08699308	103.7438256	94.59663035
95.38268701	99.68621303	91.1746501
92.00769168	96.06667807	87.81050778
89.60125611	93.44919571	85.44009198
86.23067429	90.10709941	82.20675382
83.06346791	86.63883094	79.32073816
78.48833697	81.76421284	74.88380869
73.97672555	77.1830578	70.60823278
70.22556035	73.25399619	67.0504178
66.28084902	69.12064015	63.20037637
61.90799723	64.82732036	58.9902571
58.37559604	61.19301885	55.53721228
55.63923388	58.40451145	53.00159078
53.09758962	55.81449688	50.47284233
49.9532683	52.66104116	47.42484714
47.88077526	50.52154603	45.4229753
45.50954481	48.11689708	43.05188674
43.40253057	45.93453335	41.00040854
41.51539374	44.01268418	39.20188225
39.69550423	42.12744802	37.36712765
37.99894897	40.37025563	35.77622956
36.65134395	39.13198887	34.44581611
35.27270122	37.72129582	33.08179553
34.14818705	36.42489961	31.89165568
32.99370634	35.27030854	30.73256696
32.45098418	34.66451994	30.24501921
31.97377085	34.28889158	29.73454222
31.36085931	33.63193002	29.05056174
31.06026499	33.50382434	28.7689409
30.60416176	33.04147634	28.24545634
NA	NA	NA
NA	NA	NA
NA	NA	NA
NA	NA	NA
NA	NA	NA
NA	NA	NA
NA	NA	NA
NA	NA	NA
NA	NA	NA
NA	NA	NA
NA	NA	NA
51.37800949	54.09527068	47.85252115
49.74433747	52.4118933	46.34196084
48.11551282	50.64895703	44.72181637
47.22358169	49.81932476	43.87692342
45.50086169	48.02901676	42.24699051
44.1668049	46.68757518	41.03897667
42.19346015	44.5841377	39.01053871
40.21574142	42.5071161	37.12345073

38.21888626	40.44269342	35.28206587
36.22195117	38.32265066	33.48716304
33.97516426	36.03686738	31.32716937
32.21776543	34.24656188	29.70156091
30.7521033	32.77166318	28.37309975
29.46817962	31.4320859	27.14221957
27.81645582	29.80051373	25.51423103
26.58454269	28.55396185	24.35686667
25.31136471	27.26675777	23.12634586
24.22580052	26.14647865	22.12919994
23.28623224	25.15717597	21.21434238
22.28284447	24.09155335	20.25071489
21.3746126	23.17200172	19.37513689
20.74189261	22.49444733	18.76659359
20.05142332	21.79070556	18.11824269
19.48096693	21.21282958	17.54319898
18.86689948	20.58166109	17.00991506
18.6193812	20.29570325	16.74501284
18.51647764	20.27991996	16.62937343
18.32727868	20.15751861	16.38078996
18.30051798	20.12970974	16.33474652
18.22021476	20.08271084	16.2240209
NA	NA	NA
NA	NA	NA
NA	NA	NA
NA	NA	NA
NA	NA	NA
NA	NA	NA
NA	NA	NA
NA	NA	NA
NA	NA	NA
NA	NA	NA
NA	NA	NA
72.82770256	75.78522912	69.05420678
70.31514362	73.15965851	66.66491546
67.95038065	70.73991337	64.41728452
66.43417239	69.11496054	62.9995731
64.02189054	66.68016413	60.54295711
61.89515861	64.48341611	58.55595131
58.7902667	61.3404672	55.53936729
55.71315714	58.17578053	52.55372402
52.93704542	55.27822652	49.91136851
50.08693591	52.36775657	47.24424234
46.92002965	49.14966912	44.24493847
44.38483327	46.60933351	41.81602134
42.36151063	44.55832018	39.87946984
40.53044563	42.66941106	38.11267812
38.21510456	40.33139532	35.85498871
36.61508455	38.6908096	34.32955726
34.84959833	36.94407963	32.58560855
33.30267362	35.34872188	31.12630808
31.92683281	33.92490779	29.81668032
30.54359069	32.49897696	28.43940881
29.26357878	31.19373064	27.23988845
28.29816418	30.26156587	26.2560392
27.286028	29.20639926	25.2997794
26.46231602	28.35182447	24.46127299
25.60120301	27.50062235	23.59362776

25.2293337	27.15895405	23.27202113
24.96473679	26.93315741	22.93526759
24.58495268	26.55082224	22.52852283
24.43973042	26.46579511	22.31227284
24.19134906	26.18335216	22.07810719
NA	NA	NA
NA	NA	NA
NA	NA	NA
NA	NA	NA
NA	NA	NA
NA	NA	NA
NA	NA	NA
NA	NA	NA
NA	NA	NA
NA	NA	NA
NA	NA	NA
168.7361947	184.3970369	155.1413958
166.0259062	182.2674873	153.262853
167.7393878	183.0129371	155.5683351
171.6326975	185.5113903	159.6794
171.8279541	185.3465858	160.7466161
165.2839032	177.3898392	154.9583134
153.9485347	164.7779194	144.9099084
143.4573895	152.829721	135.4717758
136.0255655	145.1854332	129.0514604
133.8344882	142.0391125	127.2028872
130.4713741	138.7768961	123.5728719
125.1966176	132.6478971	118.2106078
121.425053	128.8884762	115.1988216
118.3239216	125.3422389	112.1157096
114.1217719	121.1677464	108.1697326
111.8423169	118.7683514	106.2958623
102.7014987	108.3196473	98.17734283
96.90411394	101.8991135	92.44085122
93.79403714	98.93355609	89.73913396
88.42091814	93.23798983	84.46248487
85.72742728	90.73104577	81.73085085
81.11672812	86.22877591	77.09672321
78.17194027	83.0424082	74.20389583
75.295033	79.86047471	71.1993772
73.36067265	77.98894385	69.19192304
72.73270415	77.34246706	68.4324609
70.63438565	75.37041772	66.10921766
66.96747763	71.8923068	62.35499704
64.30236955	70.41435045	59.30207602
62.60847304	68.7831346	56.5375744
NA	NA	NA
NA	NA	NA
NA	NA	NA
NA	NA	NA
NA	NA	NA
NA	NA	NA
NA	NA	NA
NA	NA	NA
NA	NA	NA
NA	NA	NA
NA	NA	NA
62.68069751	69.9586759	56.9652686

61.3068077	68.78942136	55.78937153
60.63617683	67.24239423	55.78571129
61.08794002	67.22772598	56.46173994
60.87009548	66.07180766	56.32171579
58.6874777	63.70066805	54.53211493
55.6531496	60.39034813	52.066856
53.17540952	57.33125512	49.68442631
51.02532443	55.06852173	47.53877471
50.17043153	53.95328709	46.89082119
49.29058835	52.90313775	46.16543639
48.18698454	51.53551281	45.30523753
47.4575221	50.93322223	44.56903686
46.70678365	49.96371291	43.63167436
45.33788685	48.27956097	42.48360078
44.54516475	47.54552556	41.67532179
41.93049362	44.64852599	39.26431172
40.23108514	42.74694577	37.55144068
38.97371714	41.3328462	36.48672925
37.45614005	39.80845523	35.01562282
36.53183775	38.84236556	34.21970393
35.23802828	37.43768829	32.77982942
34.1775501	36.33245158	31.66081325
33.38362712	35.58019044	30.9728921
32.71530744	34.81493777	30.50823103
32.57210462	34.82781952	30.22545917
31.89746518	34.213765	29.49250967
30.90805933	33.28217389	28.42047017
30.00287575	32.6757111	27.23308105
29.28632558	32.49120343	26.12563655
NA	NA	NA
NA	NA	NA
NA	NA	NA
NA	NA	NA
NA	NA	NA
NA	NA	NA
NA	NA	NA
NA	NA	NA
NA	NA	NA
NA	NA	NA
NA	NA	NA
110.9549105	119.7905076	103.0721278
109.1372027	118.7559306	101.9152785
109.810036	117.9950794	103.137891
112.1644772	119.6602734	105.6885734
112.3330651	119.3053571	105.7833404
108.1300497	114.5620106	102.148485
101.1800913	106.8558494	95.85150969
94.96953396	100.2660816	90.28420484
90.38226704	95.65952752	86.04913116
89.04018919	93.94460563	84.89009492
87.09064904	91.46458086	82.78812355
84.0507258	88.47018758	79.92501437
81.92146334	86.51709105	77.88037934
80.11030705	84.23807615	76.35692168
77.43666833	81.4699384	73.59354575
76.02096117	80.25563349	72.49716777
70.37175116	74.05353603	67.15759289
66.78023955	70.14511773	63.8137375

64.6860757	67.9017857	61.83777314
61.33341946	64.31593639	58.51930381
59.58209419	62.47733671	56.86696109
56.71829672	60.01620387	53.84216081
54.77327772	57.77887551	51.96829194
53.02933602	55.82849201	50.24925452
51.82213648	54.78589149	49.25916025
51.50181444	54.21880423	48.76706705
50.16115328	53.05043577	47.47448628
47.93344408	50.82776351	45.0442537
46.24445654	49.65709532	43.10929634
45.09207177	48.5586209	41.80223059
NA	NA	NA
NA	NA	NA
NA	NA	NA
NA	NA	NA
NA	NA	NA
NA	NA	NA
NA	NA	NA
NA	NA	NA
NA	NA	NA
NA	NA	NA
NA	NA	NA
211.5108792	243.9646468	182.8170457
206.5961274	238.3388269	182.6884084
201.3384863	228.7706271	179.4977375
195.100366	220.8560983	174.5413044
188.9972044	214.1823584	169.286128
182.6372655	207.0758446	164.651308
176.1911282	198.9382011	161.2316379
168.117874	188.6075284	154.2639517
160.4756752	178.1667086	147.9981998
153.8980651	170.608253	142.040951
149.0251959	165.3017451	137.972047
143.7085824	160.0139859	133.0179137
139.8693557	158.91989	130.1321558
135.5214082	151.9590414	125.8685581
131.1854473	149.9663317	122.2704725
126.5175347	147.4814625	118.1074956
119.7301013	139.0503921	111.405565
113.8944583	131.1344204	106.5555412
110.522475	126.3499002	103.5730924
106.2703091	122.1229441	99.45999457
102.3305479	117.729708	95.60317907
98.52463372	113.1946285	92.03810472
94.43579057	108.6638781	87.70649296
90.96449573	104.0448369	84.4224593
86.99879891	98.60051781	81.11800497
83.66719196	94.72500008	77.60249055
81.41655025	92.37925397	74.96577225
78.5383141	87.52733562	72.02057623
75.58107222	86.62385468	68.25254713
73.25659977	84.29141811	65.96430169
NA	NA	NA
NA	NA	NA
NA	NA	NA
NA	NA	NA
NA	NA	NA

NA	NA	NA
NA	NA	NA
NA	NA	NA
NA	NA	NA
NA	NA	NA
NA	NA	NA
124.3666576	144.1052685	110.5070936
120.5496319	141.1254077	108.1051623
118.8528425	138.3391295	106.965894
114.46235	130.8464713	103.4595705
111.0021284	126.29672	101.1032742
106.7132716	121.4925601	97.13878313
101.9641728	114.491928	93.00308448
98.51981124	110.7338247	91.00293032
94.81202429	106.8379161	87.58960987
91.69594779	101.6088643	84.4534591
89.31237445	99.15471739	82.27281188
86.45760175	95.59801749	80.19928055
84.481184	93.63451924	78.09837021
81.91339776	90.54448185	75.75071639
78.84959501	86.37014453	73.07017031
75.69219405	82.77717194	70.15184468
71.86908817	78.33381512	66.3818867
68.11800465	73.92461094	63.19923479
65.26986139	70.94342914	60.72257632
62.30725751	67.42547935	57.57413553
59.96694004	64.94601302	55.61777995
57.61959396	62.00259901	53.12642041
55.1778867	59.85989208	51.04032913
53.54911594	57.646492	49.42396156
51.2631005	55.76919548	47.12405629
49.24531542	53.25732823	45.08711309
48.0235719	52.517683	43.76671511
46.93659627	51.77701287	42.29540445
45.73775117	50.8225034	40.80569805
44.65644736	50.11877823	39.05340419
NA	NA	NA
NA	NA	NA
NA	NA	NA
NA	NA	NA
NA	NA	NA
NA	NA	NA
NA	NA	NA
NA	NA	NA
NA	NA	NA
NA	NA	NA
NA	NA	NA
167.5504517	186.280445	151.887532
163.1762131	182.8829109	148.0958803
159.7196977	176.9293547	146.6722094
154.4062151	170.3652872	142.1863756
149.6035509	164.396751	137.2483153
144.2508181	159.4024387	133.1291816
138.6073469	151.0949964	128.9884943
132.8353182	144.7435459	124.2000298
127.1415115	138.1256715	118.9329671
122.317296	132.2712624	114.7593254
118.7121421	129.0453384	111.4729401

114.6330984	124.6252224	107.2195742
111.7373083	123.3120837	104.6725203
108.286475	118.2459689	101.8914959
104.5572542	116.4637418	98.61858467
100.6276465	112.2766509	94.76593178
95.36560722	106.6057077	89.70189126
90.58258974	100.6120904	85.49663139
87.43983752	97.47765808	82.52490721
83.78163636	93.47589896	79.03844013
80.60914832	89.3768128	75.82429204
77.52770843	86.14270652	72.94776501
74.25004292	82.5203547	69.89086027
71.69864798	79.51083119	67.21367117
68.59015521	75.33662511	64.42343538
65.9180785	71.36727897	61.95756979
64.18852049	70.76120187	59.68936036
62.233509	68.22923922	57.75190702
60.18387122	66.78283765	55.21099641
58.49066638	65.10902739	53.83128834
NA	NA	NA
NA	NA	NA
NA	NA	NA
NA	NA	NA
NA	NA	NA
NA	NA	NA
NA	NA	NA
NA	NA	NA
NA	NA	NA
NA	NA	NA
NA	NA	NA
530.0869422	596.2191201	443.3664427
523.0846376	591.4682864	441.1698733
513.1373402	581.8113866	429.4379219
499.1070329	562.7175359	415.7028231
484.401807	540.5697931	408.2828915
466.0253475	517.7218046	398.4852025
453.2945762	503.3878383	387.7259376
450.2925825	497.8854955	388.2796103
434.8970387	481.5519058	380.9080857
412.5206723	454.0007773	354.4792803
395.2513887	431.3182489	347.5679015
378.2119134	413.0256261	328.5934729
359.8009441	394.3511274	323.3496195
336.589729	367.4987869	302.7298217
313.429069	344.237622	285.2460339
303.4300253	336.8240463	278.161247
289.8425226	319.009897	265.1390833
274.5826929	305.9578557	251.6493541
265.8475179	302.3806379	243.2142885
248.187557	280.8088829	228.7662397
234.5444392	266.3023837	216.1910508
223.337219	257.6045663	206.0216097
213.1510031	249.7056894	194.7912964
206.40867	240.0440305	189.7576015
196.6588707	227.4015504	179.5000543
188.6506219	215.4124826	172.6950453
184.2487225	215.2567835	165.2276809
180.4491213	206.0567773	161.858208

175.1596903	206.3344961	153.812095
169.5073443	200.197896	146.6126637
NA	NA	NA
NA	NA	NA
NA	NA	NA
NA	NA	NA
NA	NA	NA
NA	NA	NA
NA	NA	NA
NA	NA	NA
NA	NA	NA
NA	NA	NA
NA	NA	NA
348.8815406	418.3028959	286.9280399
343.0076185	410.1397874	281.8163371
342.6397277	410.2623412	275.6547013
331.6663618	387.7116894	274.7346457
321.9644564	380.7737605	271.6564287
312.4501714	364.3599785	262.2945504
308.1297401	356.4409873	261.6687503
312.1663218	367.8018736	264.1380109
303.0560839	352.5336043	256.9792542
292.1349572	338.3421389	250.1645634
284.415147	328.2122738	243.1670997
275.5144452	314.5777792	237.6805679
265.1137203	302.2522413	230.2529918
252.3299349	287.7985565	220.4957188
236.6397579	267.0549628	209.1297508
230.97802	261.6262958	203.5530298
222.4311867	251.2852205	196.0341389
211.8807031	237.9900166	185.7443785
202.1241073	226.7279309	179.7818523
186.305153	208.154485	166.1566244
178.5164204	198.5713066	158.1201057
171.6624732	191.2625407	151.8493797
161.6707241	179.769636	144.2989311
158.5916842	176.66186	140.8545808
151.3878813	168.8177236	134.5798346
149.1828503	166.7030118	132.5578846
146.2695499	164.7450515	129.1473411
144.3655704	164.1930552	125.747673
140.0604766	161.8485335	121.8455488
135.9412506	159.5620536	115.1891468
NA	NA	NA
NA	NA	NA
NA	NA	NA
NA	NA	NA
NA	NA	NA
NA	NA	NA
NA	NA	NA
NA	NA	NA
NA	NA	NA
NA	NA	NA
NA	NA	NA
441.5516028	486.859011	395.5253448
435.0409023	480.0896858	388.5604044
429.7096	473.1982008	385.1692193
417.0637507	456.9012205	376.7636405

404.6669654	443.2945469	363.9534727
390.5266369	425.025819	354.2898305
381.82035	416.9350329	345.2933833
382.1610667	418.6669336	343.7864704
369.7571916	402.2391968	335.350805
353.0399575	383.3488781	321.7834694
340.4308323	369.6195087	310.837619
327.3433339	354.7656709	300.5039951
312.8488622	340.6824086	287.2615819
294.7381035	321.4944069	271.8357086
275.2483135	297.7155063	255.023302
267.363172	291.6476416	247.7051186
256.2579063	280.7999619	237.6667067
243.3471663	266.3530874	225.0189456
234.0366038	258.0385032	215.6039614
217.1465775	239.2853936	201.2466499
206.351818	229.322537	190.6660631
197.3119676	219.1068627	182.345633
187.1670162	209.4355862	173.5049741
182.1316429	204.0477955	168.0924806
173.5707255	194.131265	160.1917975
168.5374241	187.2117644	155.7997258
164.906446	186.3968911	151.1595692
162.0602008	182.221979	147.2730638
157.2585509	180.3628892	141.3004859
152.3963132	175.1523889	136.7343836
NA	NA	NA
NA	NA	NA
NA	NA	NA
NA	NA	NA
NA	NA	NA
NA	NA	NA
NA	NA	NA
NA	NA	NA
NA	NA	NA
NA	NA	NA
NA	NA	NA
393.0898993	463.5091373	316.9240755
389.8445986	463.0693796	310.6777092
384.7949534	457.8543592	304.1935187
379.4380263	450.4466443	306.4813458
375.2470531	442.185501	299.3682005
366.4339942	437.2383678	296.6238665
360.6613013	428.8109786	288.6567457
358.2691442	422.9899254	288.4460296
351.950677	414.1683009	284.3920072
343.4588421	406.4934441	273.6229393
336.4389406	400.7208926	273.9850719
323.2628121	382.7883154	258.9540927
309.0902778	366.4764135	253.6695365
298.3532878	354.666099	246.8150881
282.3830344	335.9428674	235.2940467
269.1142538	319.0498819	223.6033372
257.5857679	305.9321865	216.6256938
254.6325497	299.9070096	214.1620825
243.1160234	284.9456647	204.9528543
233.6003142	274.4542198	199.6387904
221.570452	257.4107959	188.4697591

212.9362934	247.0965226	181.2774403
205.008781	237.4890393	174.1396918
201.9675287	233.0966176	175.3794387
192.5234519	220.6204038	165.665459
188.654766	218.3220841	162.4304242
182.0545144	212.8613577	156.0711772
177.7455573	206.8579967	151.0816215
172.6596543	201.6887204	147.9595006
167.0448651	195.4811328	142.2599826
NA	NA	NA
NA	NA	NA
NA	NA	NA
NA	NA	NA
NA	NA	NA
NA	NA	NA
NA	NA	NA
NA	NA	NA
NA	NA	NA
NA	NA	NA
NA	NA	NA
288.8057711	362.6981604	217.6175924
286.567391	361.5085982	215.5052891
286.6151734	364.7703087	218.326609
280.8930002	351.3339944	214.1633258
275.7010573	340.5059543	211.4040545
269.7021949	336.7536296	210.0642335
265.7177126	329.6251337	210.0999074
264.218257	325.3000721	209.6922439
261.8485917	323.956218	210.5134371
256.936053	315.0286447	205.0828932
251.6804411	311.9014752	201.5821034
248.4014883	301.6882848	200.3472981
243.1975433	294.5930121	198.3400044
235.3808289	283.6985475	193.4566142
226.6882109	273.5228054	187.0409194
222.0742147	264.573801	184.2010829
212.8960287	253.2696101	175.4419829
202.5243201	237.6661683	168.8125554
196.4610141	230.2646738	165.3912833
187.8796396	217.9823674	158.1774477
179.1851621	207.8729607	153.0344698
171.4048235	199.7351986	146.93067
167.8067204	195.233943	143.8266441
162.5822512	187.0275298	138.4778872
161.1396536	187.1703057	136.8446106
156.3197804	180.6398818	133.6541874
152.2182012	175.742968	129.1919704
149.8717641	171.4940282	129.0691013
146.4451976	167.7999364	125.015583
142.6091546	165.7559522	121.7840108
NA	NA	NA
NA	NA	NA
NA	NA	NA
NA	NA	NA
NA	NA	NA
NA	NA	NA
NA	NA	NA
NA	NA	NA

NA	NA	NA
NA	NA	NA
NA	NA	NA
342.2559116	389.1067371	296.8986613
339.4786461	386.6706061	290.3554326
336.9228992	383.3461811	287.1090248
331.3412104	375.6485408	284.7685998
326.5723376	366.8613508	283.3166246
319.075012	359.6668768	277.0149776
314.1303026	353.891766	274.0444108
312.1299284	353.2106519	271.32205
307.7186197	346.0006226	267.9599538
300.9553972	338.1496222	262.4289155
294.7812723	332.0259849	257.8010924
286.5297153	321.4582117	251.1477608
276.8084581	313.1688561	242.0205533
267.4265003	302.233736	234.3278725
255.0750189	288.7113026	224.1673748
246.117672	277.303294	217.8726099
235.7529176	265.4735722	208.7198231
229.0500619	257.4312059	203.0255907
220.2173518	249.6065078	194.5698314
211.0884422	237.554001	187.3481723
200.6859398	224.4586098	178.5157999
192.421563	216.1289469	170.8385838
186.6585496	209.5793876	166.7494389
182.4995218	205.2440743	162.6471333
177.1037669	198.6856413	158.2306423
172.7166096	194.3156446	153.8967339
167.3731384	188.8776736	148.7531087
164.0577343	184.659644	144.8721274
159.8109127	181.2558319	141.9248953
155.0830521	175.2089835	137.1501063
NA	NA	NA
NA	NA	NA
NA	NA	NA
NA	NA	NA
NA	NA	NA
NA	NA	NA
NA	NA	NA
NA	NA	NA
NA	NA	NA
NA	NA	NA
NA	NA	NA
51.39807551	68.82342454	38.26691666
50.63177303	67.30949441	37.67004784
49.74751666	66.01518914	37.53409622
48.84276678	64.8687479	36.92241314
48.01514335	63.01518783	36.6906238
47.22768455	61.83977794	36.15708321
46.30327016	60.04575852	35.41646683
45.30372234	58.70305734	34.89330631
44.50181883	58.03242453	34.36758273
43.84708377	56.53322637	34.20869673
43.44861797	55.90645664	33.69820355
42.94830223	55.17393375	33.6133854
42.58507481	54.82787245	33.4977933
42.20833208	53.70028208	33.36211091

41.74930179	53.15855903	33.27227404
41.12852386	52.04207544	32.74835058
40.32862412	51.58026384	31.81557193
39.39155163	49.93832952	31.30106088
38.68979352	49.33877457	30.53031341
37.99255475	48.17317321	29.98169733
37.37161421	47.5277135	29.45381882
36.88930356	47.30537776	28.80183767
36.46586356	46.58947921	28.43011318
36.1358981	46.19896789	27.95679236
35.71444882	45.81413505	27.58023803
35.45412169	45.60313802	27.57437896
35.42079896	45.61597111	27.19896083
35.24846999	45.59990322	26.77097025
34.60041686	44.76335955	26.26114248
34.05162222	44.27101888	25.68171954
NA	NA	NA
NA	NA	NA
NA	NA	NA
NA	NA	NA
NA	NA	NA
NA	NA	NA
NA	NA	NA
NA	NA	NA
NA	NA	NA
NA	NA	NA
NA	NA	NA
45.18473301	60.45429259	33.05475344
44.37091797	60.035126	32.12452996
43.65045575	58.73294977	31.77410243
42.87955661	57.60070164	31.1884905
42.31347117	56.60633637	30.80022745
41.71690032	56.03577733	30.57960785
41.1438133	55.24125896	30.04700341
40.71612614	54.85203643	29.91479483
40.26093461	54.32061283	29.45338277
39.92517541	53.86942401	29.21720484
39.74205751	53.59536541	29.03658257
39.47420022	53.04455718	28.8299983
39.32623601	52.98544452	28.81525012
39.20207896	52.74919656	29.00211486
38.974878	52.59406078	28.78272779
38.66147613	51.98374863	28.46888555
38.22748552	51.79292611	28.18907441
37.7158092	50.97296263	27.81910152
37.3936548	50.67008389	27.34148006
37.09668937	50.69947671	27.20250209
36.83367148	50.01896201	26.86044609
36.54360227	49.78313117	26.74031815
36.19501423	49.44784038	26.30497786
35.97146682	49.31350049	26.00629794
35.75754388	49.21995674	25.63590321
35.66136916	48.80659582	25.69144089
35.83755151	49.40016437	25.62481029
36.03398628	49.82356052	26.04704414
35.82049713	49.70340307	25.54222752
35.52211604	49.35228274	25.32571039
NA	NA	NA

NA	NA	NA
NA	NA	NA
NA	NA	NA
NA	NA	NA
NA	NA	NA
NA	NA	NA
NA	NA	NA
NA	NA	NA
NA	NA	NA
NA	NA	NA
48.1375694	63.39447071	36.79032062
47.35110163	62.75603473	36.30796864
46.55706237	61.34412207	36.0624895
45.72977698	60.07373654	35.03842151
45.03670971	59.45921038	34.75283322
44.35010566	58.33912136	34.37526886
43.60772824	57.18482775	33.57841716
42.90713979	56.73980591	33.20263853
42.28634737	55.96463624	32.57507415
41.80247249	54.96065734	32.33570219
41.51652527	54.70131426	32.03313666
41.13222378	54.04622098	31.76499846
40.8830042	53.87574098	31.50809488
40.64035225	53.34731636	31.532804
40.30164874	52.68776573	31.24625138
39.83685156	52.01595595	30.82076369
39.22469639	51.50408538	30.35533242
38.50196561	50.50886137	29.74656432
37.98652188	49.87987121	29.09084084
37.48716268	49.08745582	28.86246881
37.04487785	48.92031385	28.3857239
36.66108815	48.32808513	27.94969245
36.27625723	48.12608931	27.65748128
36.00267416	47.77046427	27.36796085
35.69096336	47.65545961	27.07044215
35.51643251	47.6017703	26.91158187
35.5937196	47.58787098	26.76598539
35.61549034	47.82710345	26.69542229
35.19649681	47.43045754	26.2382505
34.77902247	46.79579393	25.8676389
NA	NA	NA
NA	NA	NA
NA	NA	NA
NA	NA	NA
NA	NA	NA
NA	NA	NA
NA	NA	NA
NA	NA	NA
NA	NA	NA
NA	NA	NA
NA	NA	NA
19.90830984	27.43493735	14.3506943
19.49699622	26.91145762	14.00348318
19.04184823	26.26036179	13.72877296
18.68984532	25.77176931	13.43867345
18.39313659	25.34978851	13.22255482
18.0934832	25.03668536	12.94765323
17.69870362	24.40910396	12.66257991

17.3438081	23.99325942	12.35847871
17.01408107	23.68437771	12.0969287
16.68077991	23.28790262	11.73529837
16.45227943	23.04982212	11.54593748
16.17674992	22.65014218	11.31993679
15.94529856	22.27112687	11.18377945
15.67906527	21.92898073	10.99052156
15.3362743	21.42059538	10.70119884
15.09788145	21.15018881	10.49090485
14.86314052	20.81125382	10.34073113
14.60128606	20.38687458	10.18418333
14.361532	20.02657067	10.0146656
14.16609197	19.81462999	9.886990272
14.03625335	19.58755522	9.789616921
13.95593032	19.39792489	9.735527041
13.90560761	19.4121341	9.62251626
13.89828036	19.35971926	9.645812738
13.86236487	19.42600169	9.625317325
13.86298636	19.36994123	9.611410876
13.89420445	19.50347192	9.541951289
13.9019318	19.58422196	9.554070047
13.91352118	19.67972711	9.650770684
13.93722963	19.85122369	9.612442175
NA	NA	NA
NA	NA	NA
NA	NA	NA
NA	NA	NA
NA	NA	NA
NA	NA	NA
NA	NA	NA
NA	NA	NA
NA	NA	NA
NA	NA	NA
NA	NA	NA
24.35940058	35.3021401	16.33556973
23.66892188	34.25457308	15.84231341
23.02257693	33.09462597	15.43990673
22.50810037	32.22615637	15.02685332
22.09960767	31.66176321	14.69807503
21.84424453	31.23430939	14.6433326
21.62377906	31.02786637	14.52040118
21.38915351	30.67077612	14.26116066
21.17440273	30.50205194	14.1034105
20.95989754	30.19919006	13.91103589
20.70339607	29.75082881	13.74231825
20.42388548	29.50718395	13.55759428
20.09119279	29.06820835	13.26185186
19.75919218	28.57167714	13.074822
19.40316149	27.9326311	12.77267029
19.16111938	27.53810735	12.57206249
18.94785342	27.15468861	12.42938802
18.75112617	26.79165469	12.41813653
18.59097019	26.48877018	12.32032057
18.46110874	26.31587175	12.21155991
18.3944686	26.27109013	12.1791404
18.40596297	26.32042437	12.14490206
18.44028949	26.42607323	12.16440737
18.48029414	26.38783232	12.16077117

18.52265564	26.48089131	12.20625408
18.5936655	26.68704319	12.21333614
18.68325537	26.8044547	12.2536686
18.76303023	27.07661851	12.32941981
18.85442519	27.21770117	12.30633538
18.97964853	27.51414908	12.38833732
NA	NA	NA
NA	NA	NA
NA	NA	NA
NA	NA	NA
NA	NA	NA
NA	NA	NA
NA	NA	NA
NA	NA	NA
NA	NA	NA
NA	NA	NA
NA	NA	NA
22.14589616	31.36442026	15.22251082
21.59156291	30.47707132	14.88952149
21.04249221	29.67856527	14.5185222
20.61049419	29.04375594	14.24908417
20.25977065	28.54193876	14.00674244
19.99010415	28.12271392	13.87607487
19.6923292	27.81794179	13.64140311
19.40441286	27.45683719	13.39484427
19.13408752	27.07025345	13.12239138
18.86204804	26.83931542	12.90175562
18.61393418	26.41717246	12.68512622
18.33132641	26.21443607	12.46958116
18.04332711	25.71745283	12.2513829
17.73823883	25.29599485	12.07157252
17.38539234	24.77654226	11.78660851
17.14294597	24.30053388	11.60648288
16.91436912	24.02254546	11.41839898
16.67943573	23.56492005	11.32854657
16.47292317	23.23318888	11.1723017
16.30553777	23.01742479	10.98515742
16.20371468	22.8079875	11.05482268
16.16807256	22.83654467	10.93914015
16.15858732	22.81365298	10.95918153
16.17236815	22.88992976	10.8767975
16.17415112	22.89050711	10.92452637
16.20866795	22.94852811	10.90661894
16.26658449	23.12604709	10.93536191
16.30786256	23.2693978	10.96666496
16.35729449	23.43448981	10.96334128
16.43079327	23.55535923	10.98366989
NA	NA	NA
NA	NA	NA
NA	NA	NA
NA	NA	NA
NA	NA	NA
NA	NA	NA
NA	NA	NA
NA	NA	NA
NA	NA	NA
NA	NA	NA
NA	NA	NA

42.56173643	57.24636184	31.0952576
41.8177316	57.25487348	30.40979254
41.17065412	54.72306432	30.44269464
40.52952692	54.46646724	29.98060894
39.93098797	53.6198269	30.05960711
39.24324089	52.33348062	29.35712484
38.23232175	50.91342665	28.52320222
37.11990016	49.63703116	27.82981367
36.31149778	48.36948765	27.38864329
35.90828494	47.6240559	27.33938307
35.72176927	46.96744901	27.01443681
35.36369555	46.84499111	26.87413132
35.12919153	46.318599	26.72591402
34.89293166	45.72770385	26.94842789
34.54368839	45.48561919	26.77129461
33.97199322	44.45593731	26.12314793
32.72215131	43.36277048	25.04875465
31.31120833	41.49934666	24.13447725
30.18597327	39.92436367	22.987644
29.10794516	38.43486817	22.06615096
28.48530438	37.72737867	21.62197239
27.94611848	37.01508473	21.24501474
27.52851542	36.44782378	20.63470641
27.15799442	36.3199311	20.45059925
26.81132067	35.87402426	20.04961226
26.59244144	35.75016716	19.90652434
26.44911836	35.42061044	19.60137058
26.19788027	35.0802657	19.50338801
25.79819296	34.66794976	18.80796258
25.50709862	34.22584452	18.6993794
NA	NA	NA
NA	NA	NA
NA	NA	NA
NA	NA	NA
NA	NA	NA
NA	NA	NA
NA	NA	NA
NA	NA	NA
NA	NA	NA
NA	NA	NA
NA	NA	NA
37.86361948	52.61761934	26.17533927
37.20590378	51.90254164	25.89060954
36.55664352	50.8367087	25.53664358
36.05736601	50.26324964	25.17662672
35.63300227	50.01155532	24.94877466
35.16635369	49.18859384	24.61992054
34.65845595	48.75879735	23.97608333
34.20146708	48.22340987	23.60934928
33.76404998	47.71834253	23.30651569
33.53196106	47.22956957	23.06731739
33.38562002	47.36523468	22.84860733
33.21469263	46.62312544	22.90981156
33.17611889	46.84695298	23.02435559
33.15189175	46.38545439	22.87294437
33.04377596	46.47890221	22.88274337
32.72515635	45.8857247	22.55741417
32.13576162	45.12480358	22.31171326

31.37021238	43.96178055	21.69959558
30.63420488	43.26077881	21.00065308
30.04165866	42.40661111	20.6920476
29.6262505	42.17842941	20.33307214
29.29685403	41.54665194	20.00347719
28.97435744	41.12341048	19.91492703
28.7357822	40.79575942	19.49264949
28.58333772	40.43867691	19.56589755
28.53074304	40.23776563	19.4626086
28.74311854	40.84302461	19.27569482
28.96795128	41.1822549	19.61966815
28.89425345	41.34507657	19.47345373
28.7715785	41.19151244	19.45458056
NA	NA	NA
NA	NA	NA
NA	NA	NA
NA	NA	NA
NA	NA	NA
NA	NA	NA
NA	NA	NA
NA	NA	NA
NA	NA	NA
NA	NA	NA
NA	NA	NA
39.64520835	54.14222461	29.06658707
38.96896631	53.13815113	28.63987032
38.34357601	52.11249587	27.95693265
37.80707335	51.53678195	27.67318107
37.31567329	50.46877781	27.4057041
36.75562389	49.967527	27.05069693
36.01990313	48.77811466	26.37247385
35.26689866	48.20883264	25.82937367
34.66192965	47.29821955	25.18913751
34.36543142	46.81314834	25.08907154
34.21431011	46.6956866	24.78801348
33.96020236	46.0292874	24.60730104
33.83911257	46.00208948	24.76675753
33.72760967	45.59651532	24.48145753
33.5130227	45.37891638	24.67015046
33.07957519	44.73961025	24.27773866
32.18727836	43.87635417	23.43826828
31.12252332	42.3680829	22.75358244
30.20241141	40.98755063	21.76695094
29.376951	40.22650728	21.24936697
28.86198187	39.67096712	20.93827556
28.432942	38.93561305	20.45944682
28.06342789	38.4104145	20.2266622
27.76678017	38.17925555	19.96108962
27.52966674	38.15863091	19.77980266
27.40001344	38.01950937	19.56928844
27.44253478	38.12112886	19.41758844
27.44527997	38.02931125	19.32688542
27.22654783	37.93825649	19.12968712
27.02821494	37.71253355	18.94621366
NA	NA	NA
NA	NA	NA
NA	NA	NA
NA	NA	NA

NA	NA	NA
NA	NA	NA
NA	NA	NA
NA	NA	NA
NA	NA	NA
NA	NA	NA
NA	NA	NA
65.39482132	89.83126474	47.28345889
63.86655554	84.95304695	46.30763221
62.09770857	82.28850856	45.98997657
60.33462486	79.43193062	44.19192646
58.80127341	77.3893699	43.53861963
57.5431577	74.455726	43.24663675
56.05572313	72.76773083	42.17244135
54.21812027	70.25755951	41.60391584
52.83007719	67.75682384	40.35598025
51.76960161	66.00281302	39.88112766
51.3692304	66.22346174	39.48783192
50.94635107	64.77911719	40.07826391
50.78744555	64.28743156	40.0078387
50.60296843	63.66232056	40.09378931
50.32410488	62.87086565	40.27728386
49.50598487	61.99025601	39.92652578
48.23657752	60.40134195	38.73096419
46.65461709	57.86802012	37.96476775
45.51804243	56.76548824	36.43452297
44.48228035	55.18617404	35.60186144
43.54723114	54.01034454	34.84356791
42.73171353	52.82349435	33.42740008
42.03706847	52.45600255	32.26419911
41.37282266	51.79087495	31.9643388
40.52725088	51.15661662	31.18648396
39.86757269	50.12088631	30.51949188
39.36062091	49.95370751	30.08385743
38.66914049	48.96574253	29.2316824
37.40323494	47.64598041	28.31502425
36.41878373	46.45098379	27.1202703
NA	NA	NA
NA	NA	NA
NA	NA	NA
NA	NA	NA
NA	NA	NA
NA	NA	NA
NA	NA	NA
NA	NA	NA
NA	NA	NA
NA	NA	NA
NA	NA	NA
58.58358879	76.61363282	43.27103841
57.22064674	76.08735771	42.34858838
56.00069334	73.92450198	41.59463305
54.50770607	71.89468379	40.13826153
53.39591871	70.51722104	39.6061664
52.15563825	69.12830361	38.63912271
50.88378516	67.29691611	37.90951696
49.94574431	66.35241386	36.83638639
49.04852556	65.43217048	36.33435497
48.47002097	63.96254303	36.29695785

48.41535	64.71592867	35.99396256
48.18963283	63.88926	35.72471968
48.28953036	63.94432849	36.09393554
48.46253454	63.34230312	36.29272422
48.32556556	63.71706878	36.39322692
47.80679986	62.52563252	35.83162936
46.89130734	61.54609869	35.22489325
45.58311094	59.63682186	34.30329657
44.55538957	58.93587792	33.48043735
43.67058754	57.53065009	32.69233276
42.86015745	57.07798892	31.93282854
42.11460227	55.95977364	31.51755587
41.39186847	55.18193951	30.59367713
40.83160361	54.68876703	30.1581691
40.26245472	54.2039754	29.53124543
39.77499259	53.95496202	29.33741944
39.56254154	53.42901991	28.73928877
39.39065087	53.58700553	28.67378503
38.8196686	52.9258869	28.39434763
38.19298146	52.25498351	27.66734259
NA	NA	NA
NA	NA	NA
NA	NA	NA
NA	NA	NA
NA	NA	NA
NA	NA	NA
NA	NA	NA
NA	NA	NA
NA	NA	NA
NA	NA	NA
NA	NA	NA
61.94344272	81.99675907	47.85717228
60.49462108	79.01467084	46.73770274
59.00062862	77.19743151	45.58332411
57.37211823	75.25682577	44.00516735
56.04072346	73.24881902	43.36092608
54.7851545	71.35038739	42.63149303
53.394818	69.82624011	41.42338741
52.01190935	68.01457596	40.70268045
50.87017479	66.38744753	39.56156791
50.05713853	65.24033153	39.13107957
49.84215023	64.84672032	39.01119081
49.52160385	64.130124	38.74604167
49.50313519	63.94136137	38.8515298
49.51400549	63.91926038	39.07363124
49.30822724	63.02462485	38.86694873
48.63597738	61.95101212	38.44727806
47.54125608	60.95412201	37.44355402
46.08432464	58.80041554	36.46323755
44.98440211	57.51605165	35.41316927
44.0115905	56.1561346	34.50687078
43.13046478	55.08288029	33.88281586
42.34605365	54.36214272	33.22239659
41.6314937	53.73056334	32.29080514
41.01892297	52.93470246	31.84742669
40.3157644	52.25195665	31.12445258
39.73899347	51.91188985	30.79766274
39.38093402	51.58308713	30.12103026

38.96059751	51.25971457	29.73845341
38.05846949	49.99810014	28.79209979
37.25747643	48.91406321	28.05734007
NA	NA	NA
NA	NA	NA
NA	NA	NA
NA	NA	NA
NA	NA	NA
NA	NA	NA
NA	NA	NA
NA	NA	NA
NA	NA	NA
NA	NA	NA
NA	NA	NA
63.77332949	94.47304125	44.37065364
63.19448071	93.63836404	44.01087869
62.34360113	93.96104643	43.54498893
61.25673802	90.23057194	43.53000326
60.09514217	87.93460886	42.87269935
58.93899888	84.38770331	42.05601553
57.85053008	81.94547644	41.78431533
57.12500794	80.17735184	41.73544271
56.17298775	78.72832927	41.19973641
55.11346116	75.6670904	40.74157769
54.35963999	74.11863617	40.27139795
53.67193529	72.66002867	39.38260729
52.94962089	70.73316044	39.61582491
52.0438709	69.01267466	39.2664315
51.10416692	67.45698783	39.20309886
50.38418987	67.14240665	38.26822201
49.63735973	65.5539611	38.06414304
48.71216864	64.85162658	37.56468181
48.26187348	63.55955671	37.2738513
47.48656502	62.40403641	36.57796334
46.7259555	61.45007087	36.36870945
46.08528406	60.26201527	35.70223849
45.40346581	59.08467726	35.14241264
44.98499865	58.6946353	34.50117024
44.46959783	57.81979376	33.97656564
44.1839608	58.09700426	33.66425428
44.31531139	58.31505748	33.8118727
44.35295568	58.4489496	33.94155249
43.33053671	56.43413971	32.88452073
42.35562192	56.06932625	31.62651
NA	NA	NA
NA	NA	NA
NA	NA	NA
NA	NA	NA
NA	NA	NA
NA	NA	NA
NA	NA	NA
NA	NA	NA
NA	NA	NA
NA	NA	NA
NA	NA	NA
59.42206695	79.06924902	43.34913828
58.54906403	78.38451443	42.33150957
57.85273345	77.38401833	42.28601895

56.96172533	75.75740723	41.82918774
56.23194448	74.45080847	41.29895577
55.42898234	73.46989909	40.54345125
54.79019135	72.44949793	40.36700054
54.45604066	72.39287125	39.93306878
53.77822998	71.0671067	39.72030642
53.18679776	71.2074767	38.94877371
52.90795599	70.36665658	38.98278165
52.51223797	70.07865271	38.76447506
52.17660667	69.19998372	38.53177771
51.73936972	68.31583566	38.43513271
51.24796027	67.64352579	38.23194861
50.87449356	66.90770073	38.03145369
50.44330148	66.76409972	37.76989301
50.15000522	66.3188408	37.66622754
50.23542952	66.62042546	37.64368288
50.09751917	66.86320794	37.13640468
49.94828136	66.90068164	37.25123567
49.55410276	66.22754353	36.97571268
48.84913946	65.59800208	36.6725148
48.56993281	64.7719729	36.36110009
48.19021633	64.32250949	36.23348544
48.12789453	64.48702477	35.89390362
48.39049121	64.76592376	35.90965809
48.68784136	65.10230799	36.22702313
48.04455938	65.04607709	35.35839933
47.2343009	63.62014947	34.89951389
NA	NA	NA
NA	NA	NA
NA	NA	NA
NA	NA	NA
NA	NA	NA
NA	NA	NA
NA	NA	NA
NA	NA	NA
NA	NA	NA
NA	NA	NA
NA	NA	NA
61.75707112	82.09113221	46.48872782
61.02752449	80.78903564	45.95545046
60.24605154	79.37550857	45.61739595
59.24882185	78.73223801	45.02378694
58.29259499	76.59051099	44.4446021
57.30634996	74.67096981	43.63050527
56.43709504	73.23210361	43.15315148
55.89975517	72.76141729	43.02677672
55.07862439	71.69775031	42.42170757
54.25207255	70.50025516	41.95545479
53.73350938	69.98305073	41.6962832
53.18626854	69.3583709	41.34235241
52.6555681	68.11550299	41.02802126
51.98453185	67.22793127	40.76821046
51.27187863	66.50178188	40.18785233
50.72134736	65.51060593	39.69280532
50.12218349	64.80493596	39.39012114
49.50714041	64.666146	38.84322707
49.31967811	64.80999989	38.53328161
48.85667082	64.49051793	38.11411927

48.39931567	63.84106619	37.83516085
47.88286556	63.35061375	37.04108903
47.18699807	62.48033262	36.56875349
46.83664753	61.93317214	36.38038385
46.38933692	61.49187782	35.90277922
46.21770692	61.4472183	35.77327158
46.41772864	61.67831283	35.69621209
46.58998752	62.0825667	35.79274518
45.76234686	60.77294632	35.03872592
44.87236667	59.50434912	34.00308502
NA	NA	NA
NA	NA	NA
NA	NA	NA
NA	NA	NA
NA	NA	NA
NA	NA	NA
NA	NA	NA
NA	NA	NA
NA	NA	NA
NA	NA	NA
NA	NA	NA
85.56812699	123.2630275	52.77386378
85.15707941	121.9999435	52.61818185
84.57256685	121.1248912	52.18495126
83.97117576	120.1837971	52.3775737
83.4785194	118.9521519	51.46404744
82.82437585	118.0877391	51.39019789
82.40395806	116.2859401	51.09955289
81.73559914	115.4543727	51.0625546
81.29316808	115.2213061	50.71452788
80.55375235	112.693799	50.72130915
79.3585011	110.2197287	50.10374567
77.40227118	107.8979641	49.40723967
75.5549666	104.4976306	48.64407155
74.18388288	102.2257933	47.95454644
72.55138482	99.72408401	47.35709785
70.9514872	96.82974947	47.19875314
70.03588547	95.13078429	46.25445362
69.60859496	93.91414811	46.7575446
68.76631279	92.51134149	46.92451104
68.07568855	91.25792902	46.78183815
66.7252963	89.00907257	46.05530006
66.10013139	87.36374411	45.94659173
65.31352824	86.24284761	45.81260004
64.8529531	86.23646738	45.66704658
64.11610981	83.95044325	45.01100098
63.61415234	84.11603883	44.58400819
63.45013642	83.46246663	44.70261923
63.19953052	83.19079516	44.42372435
62.41358175	82.56418066	44.02545079
61.55223499	81.50701817	43.49661566
NA	NA	NA
NA	NA	NA
NA	NA	NA
NA	NA	NA
NA	NA	NA
NA	NA	NA
NA	NA	NA

NA	NA	NA
NA	NA	NA
NA	NA	NA
NA	NA	NA
61.64808635	81.96139339	44.22368023
61.21011481	81.70754527	43.40929686
60.82722108	81.20936078	43.14260975
60.33300987	80.56501847	43.08394864
59.89993552	80.08981803	42.4986495
59.53755122	79.3909561	42.49747532
59.37437834	79.19856433	42.34133787
59.08845804	79.4585678	42.34897879
58.9411214	78.97450531	42.27022834
58.55679858	78.46147874	42.07958735
57.90541129	77.6766595	41.94712956
57.23553996	76.53885192	41.48967254
56.61963817	75.48568779	41.11347344
56.03417661	75.07834633	40.8242128
55.43074827	74.17112573	40.46718292
54.96761244	73.33051656	40.06262219
54.53589732	72.37093153	39.69557439
54.30728494	72.52753037	39.83558819
54.36059571	72.46505492	39.56161398
54.24002334	72.39550305	39.36252469
53.99623518	72.08873458	39.48016641
53.62091763	71.63931093	38.93833898
53.24452872	71.19600316	38.65780463
52.87141423	70.64456062	38.59052205
52.68556048	70.56065794	38.55327729
52.42501696	70.42311227	38.44911045
52.56439194	70.6642564	38.35753463
52.86325785	71.00305704	38.56644466
52.69014729	71.03159282	38.37995602
52.43583027	70.90668658	38.00473831
NA	NA	NA
NA	NA	NA
NA	NA	NA
NA	NA	NA
NA	NA	NA
NA	NA	NA
NA	NA	NA
NA	NA	NA
NA	NA	NA
NA	NA	NA
NA	NA	NA
73.81431812	97.22715036	52.28197822
73.37922864	96.14932302	52.30494322
72.89185804	96.10492282	52.28857348
72.34479816	94.4752464	51.53425359
71.88286157	94.42554266	50.76540278
71.37331046	93.4000207	50.57045142
71.08470398	92.83269006	50.59531773
70.61088592	92.78957052	50.49631059
70.32291881	91.50254993	50.28389441
69.76557151	90.85217664	50.01441189
68.84731275	89.16703508	49.23052607
67.53589838	87.3910888	48.72393594
66.30623697	85.38884526	47.90116805

65.32264375	84.11076838	47.57869425
64.20411984	82.46421934	46.87748941
63.16661311	81.11135282	46.12273251
62.48773713	80.84235083	46.01650499
62.15090411	80.09398171	45.67272775
61.74791039	79.48371426	45.49372729
61.33444303	79.12063301	45.24670549
60.53428282	78.37215584	44.8941919
60.02694178	77.61155223	44.31136864
59.4462307	76.87925003	44.1299219
59.02784244	76.61871783	43.74890771
58.56785956	75.82036414	43.25966451
58.18443368	75.37055056	43.13337126
58.17460563	75.65689164	43.18931049
58.19944299	75.80240715	43.04566565
57.71720605	75.05620681	42.78103938
57.15549013	74.70151549	42.75423688
NA	NA	NA
NA	NA	NA
NA	NA	NA
NA	NA	NA
NA	NA	NA
NA	NA	NA
NA	NA	NA
NA	NA	NA
NA	NA	NA
NA	NA	NA
NA	NA	NA
69.08137863	124.3275217	35.61553169
68.92444219	124.250617	35.54461718
68.80004529	124.0240416	35.4868876
68.70231654	123.9552897	35.47214015
68.63638095	123.7824565	35.43688562
68.62539195	123.6743217	35.39160086
68.66874605	123.773619	35.51427179
68.75385053	123.9772323	35.50831639
68.84891913	124.0275098	35.53122652
68.93499648	124.2550111	35.58274028
69.00047116	124.1950578	35.5728739
69.0164813	124.0404353	35.54146945
68.99002963	123.9287714	35.57126007
68.94830432	123.6070851	35.55065702
68.91042704	123.2833933	35.51854989
68.91153067	123.1560924	35.48760847
68.8503193	123.0813573	35.45399928
68.69162327	122.7930431	35.38600624
68.5234344	122.5613471	35.32387936
68.43053236	122.5174232	35.3054681
68.48403063	122.7254955	35.30600801
68.66570851	122.95174	35.46471887
68.89957365	123.299047	35.56959656
69.15361898	123.7132032	35.74027585
69.40290996	124.0953716	35.84580082
69.62824068	124.6208066	35.9760956
70.16620677	125.7971588	36.2416722
70.67524467	127.0455377	36.48933784
70.86564844	127.4031391	36.58080221
71.11250196	127.7625093	36.71192338

NA	NA	NA
NA	NA	NA
NA	NA	NA
NA	NA	NA
NA	NA	NA
NA	NA	NA
NA	NA	NA
NA	NA	NA
NA	NA	NA
NA	NA	NA
NA	NA	NA
74.25135623	132.563517	38.28079677
74.18248884	132.4070956	38.32040093
74.12987955	132.4187351	38.27631842
74.08395679	132.4980395	38.30799387
74.04964319	132.4126837	38.32810854
74.0364828	132.4570394	38.22529767
74.08345506	132.3890097	38.20144336
74.20230396	132.6649435	38.2991805
74.33631514	132.9351301	38.33075198
74.45340509	133.1477745	38.38527939
74.52277951	133.2203597	38.36796869
74.43950091	132.8466515	38.35995877
74.21080707	132.5378398	38.26699367
73.91740268	131.9179465	38.17964979
73.65717589	131.3496322	38.04408761
73.51484227	131.4000098	38.00186377
73.40732455	131.1049206	37.94783268
73.23985875	130.8870176	37.8910333
73.09258986	130.697701	37.83905284
73.02794177	130.4065706	37.84391027
73.11679284	130.5087478	37.90653744
73.32098174	130.7582599	38.04432226
73.56642925	131.0567354	38.15076433
73.82890866	131.5801751	38.28415372
74.0847297	132.1680565	38.39380407
74.31708818	132.642819	38.52549954
75.01646776	133.7667559	38.81350224
75.68624636	134.9596372	39.05657701
75.87977203	135.3715604	39.24839473
76.1291813	135.7563922	39.34657274
NA	NA	NA
NA	NA	NA
NA	NA	NA
NA	NA	NA
NA	NA	NA
NA	NA	NA
NA	NA	NA
NA	NA	NA
NA	NA	NA
NA	NA	NA
NA	NA	NA
71.68042104	128.6402975	36.95159552
71.56577447	128.4468422	36.93498626
71.47549827	128.3897935	36.88449824
71.40200591	128.267547	36.89127096
71.35086206	128.2198063	36.89649991
71.33824989	128.1982849	36.86615558

71.38358982	128.2037239	36.86145621
71.48609206	128.4103812	36.902496
71.60120508	128.5029767	36.92983002
71.7034365	128.7263417	36.98487076
71.77148786	128.7350814	36.9713214
71.73833207	128.5124289	36.95258536
71.61056458	128.2547057	36.92156128
71.44250046	127.7838588	36.867865
71.29296576	127.3083065	36.78381467
71.22191646	127.032008	36.74705866
71.1372995	126.8330483	36.70407577
70.974109	126.4838078	36.64314813
70.81648229	126.2145498	36.58023466
70.7382657	126.0719938	36.55428576
70.81008746	126.1997907	36.60090235
71.00346572	126.4369356	36.75384373
71.24333796	126.9937119	36.84394755
71.50183284	127.4255307	37.00560574
71.75487569	128.0044679	37.09541165
71.98409721	128.3954798	37.21812205
72.604118	129.8013573	37.51431353
73.1946678	131.3032402	37.77431042
73.38599781	131.6593131	37.9074488
73.63301177	132.0809708	38.02779235
NA	NA	NA
NA	NA	NA
NA	NA	NA
NA	NA	NA
NA	NA	NA
NA	NA	NA
NA	NA	NA
NA	NA	NA
NA	NA	NA
NA	NA	NA
NA	NA	NA
62.91759854	113.6194712	31.86212154
62.75438379	113.2712751	31.66512241
62.62558432	112.6465506	31.53276604
62.52020781	112.9771351	31.4936099
62.44888073	113.3785275	31.39884252
62.43200482	112.9329804	31.42671975
62.45838296	113.1519512	31.34180924
62.54158032	113.4554693	31.44653392
62.63666392	113.8863658	31.60894362
62.72038233	113.8783329	31.76529758
62.76198441	113.7077019	31.96252467
62.63321528	113.3835661	31.76574377
62.29144397	112.8310713	31.56623403
61.83041061	111.826991	31.48056573
61.32651985	110.2960775	31.38496669
60.88906331	108.8447139	31.01729434
60.44070324	108.7459228	30.83545521
59.93164613	108.0441392	30.54121058
59.47996143	107.5566992	30.17219831
59.18710221	106.8876351	30.06656436
59.13165428	106.6741512	30.09323633
59.38585206	107.0826391	30.20431575
59.82174762	107.7700523	30.42147864

60.29795939	108.8074524	30.69949515
60.69383148	110.0249625	30.89588011
60.87348382	110.1574788	30.94910897
60.93506149	110.3951113	31.02926259
60.98579337	110.4412321	31.04170421
61.24073346	110.8652831	31.05585793
61.78857186	111.9486873	31.36283627
NA	NA	NA
NA	NA	NA
NA	NA	NA
NA	NA	NA
NA	NA	NA
NA	NA	NA
NA	NA	NA
NA	NA	NA
NA	NA	NA
NA	NA	NA
NA	NA	NA
67.7254876	121.7034612	34.62849378
67.61710131	121.4603423	34.56018192
67.50443003	121.1370326	34.4665153
67.39877539	121.8390466	34.34596581
67.31733539	121.4343594	34.36529321
67.25901229	121.1718169	34.3051003
67.21043643	121.4697243	34.32334465
67.14243922	121.6775327	34.34108752
67.05232518	121.6032201	34.28331186
66.96474547	121.3676346	34.23323974
66.88811513	121.408032	34.24266831
66.53596579	120.5379228	34.10700321
65.7646252	118.9349996	33.74001004
64.78369869	116.8311802	33.15812254
63.8267036	115.5286369	32.58911017
63.11158365	114.3883986	32.20301215
62.5612738	113.1003485	32.02661652
62.0099858	111.6561746	31.7597748
61.56304684	110.9469126	31.4076091
61.29038472	110.5118348	31.20515691
61.30878221	110.2996505	31.15102843
61.67919115	110.5992535	31.38890124
62.28521903	111.8045798	31.70960337
62.94994367	112.6140789	32.00051263
63.50226949	113.4437178	32.24737645
63.78075366	113.6828323	32.43918785
63.88114556	113.9242328	32.51241068
63.95749004	114.1451996	32.55264907
64.32734454	115.0108324	32.85964539
65.06210589	116.2074722	33.06972633
NA	NA	NA
NA	NA	NA
NA	NA	NA
NA	NA	NA
NA	NA	NA
NA	NA	NA
NA	NA	NA
NA	NA	NA
NA	NA	NA
NA	NA	NA

NA	NA	NA
65.36550378	118.0829942	33.27037179
65.22783533	117.7963123	33.17192032
65.10441054	117.3078881	33.09427107
64.99553753	117.1198797	32.9411882
64.91539664	116.5834487	32.89342955
64.87338574	116.4567964	32.79328473
64.85597963	116.6033425	32.86561552
64.85520094	116.7824155	32.8837746
64.8486265	117.1756192	32.90186173
64.8393467	117.2459983	32.99173812
64.81674402	117.509426	33.09227445
64.57302227	116.8067319	32.92801032
64.01359775	115.6682875	32.61465168
63.29048013	114.3274139	32.24176376
62.55880475	113.0471955	31.80372954
61.98170011	111.6963535	31.4949391
61.48339073	110.9394117	31.25357097
60.95598627	109.9390348	30.93936761
60.51022148	109.4500433	30.66119534
60.23081042	108.9708702	30.53921628
60.21348294	109.1359974	30.61356212
60.52518065	109.400549	30.78750126
61.0443489	110.2833341	31.05635718
61.61255443	111.0956161	31.34397067
62.08412153	111.8184626	31.55241479
62.3113667	111.9519435	31.66316678
62.39137556	112.1309208	31.70369141
62.45398475	112.3746833	31.70532001
62.76193114	113.0536424	31.92583949
63.39508446	114.2579662	32.16770792
NA	NA	NA
NA	NA	NA
NA	NA	NA
NA	NA	NA
NA	NA	NA
NA	NA	NA
NA	NA	NA
NA	NA	NA
NA	NA	NA
NA	NA	NA
NA	NA	NA
63.91484189	114.8624049	32.80715764
63.63075002	114.5104853	32.6125654
63.38307304	114.1046462	32.50672295
63.16071165	113.8645103	32.39503905
62.9611085	113.4858859	32.35003036
62.80334603	113.2413544	32.22608231
62.6520701	112.7376259	32.2048516
62.50428356	112.440722	32.00111303
62.35472631	111.9838888	31.86159662
62.1948753	111.8288178	31.81067303
62.04889124	111.2548392	31.6729077
61.89387652	111.3389281	31.57758295
61.72804952	111.0289286	31.60232443
61.57403307	110.9304098	31.42904358
61.43262455	110.9573056	31.4007099
61.33731856	111.1910549	31.39624901

61.17018517	110.5260507	31.30284232
60.88625293	109.8565454	31.2232612
60.56967782	109.4429581	31.06731015
60.28891798	108.4358529	30.94242943
60.14813003	108.0159619	30.94075498
60.13003737	107.9628099	30.95313284
60.16502964	108.1344745	30.89332901
60.22381951	108.0859113	30.98190624
60.28313455	108.3533498	31.00868788
60.36134199	108.7507493	31.16355915
61.22836705	109.8358743	31.54701288
62.07261864	111.9789506	32.03158659
62.0782306	111.9053034	32.13695801
62.09799642	111.9670002	32.11220658
NA	NA	NA
NA	NA	NA
NA	NA	NA
NA	NA	NA
NA	NA	NA
NA	NA	NA
NA	NA	NA
NA	NA	NA
NA	NA	NA
NA	NA	NA
NA	NA	NA
74.09409271	132.7788363	38.11270952
73.91943263	132.600922	38.04597529
73.76932406	132.0810956	38.0069536
73.6189412	131.6575773	37.87569563
73.44193461	131.0879259	37.75588861
73.25328059	130.8448518	37.64543273
73.12091419	130.791473	37.47998284
73.12221868	131.0129133	37.52572238
73.16377659	131.2240112	37.60302421
73.16223068	131.4145377	37.50225557
73.06910633	130.9700822	37.44970443
72.74609525	130.4044598	37.20349958
72.25734064	129.8076878	37.09275009
71.68358167	128.626009	36.8474275
71.14917469	127.5441839	36.54052383
70.73574856	126.7331649	36.31548348
70.36809533	126.0398822	36.19771624
69.94238927	125.4183269	36.00621047
69.55748365	124.8209553	35.86147503
69.26984443	124.3590596	35.75566714
69.16992132	124.2603634	35.64597449
69.20827459	124.1805543	35.66652139
69.30048213	124.4273578	35.74227202
69.41952361	124.6597773	35.8345688
69.54039215	124.7485623	35.8518843
69.65140045	124.9639192	35.84379228
70.78842063	126.6277529	36.47989536
71.88028875	128.5237241	37.06386834
71.88941364	128.4379428	37.04938086
71.90112401	128.5261853	37.07119747
NA	NA	NA
NA	NA	NA
NA	NA	NA

NA	NA	NA
NA	NA	NA
NA	NA	NA
NA	NA	NA
NA	NA	NA
NA	NA	NA
NA	NA	NA
NA	NA	NA
69.25761629	124.0675383	35.57720396
69.01995743	123.9612856	35.50642612
68.81370938	123.4847957	35.29160012
68.62127587	123.1526524	35.19688583
68.42761266	122.6781473	35.11530147
68.24972051	122.4831253	35.05782603
68.10670029	122.1013428	35.0003593
68.03656241	122.0468192	34.93725038
67.98652814	121.8797372	34.87143076
67.90802153	121.8650675	34.89424532
67.78698667	121.5617589	34.7640475
67.54238622	121.2972408	34.61895804
67.2070763	120.6916053	34.46115152
66.83428457	120.1462451	34.24255483
66.48683901	119.5956496	34.06288926
66.22312294	119.2406965	33.93071373
65.94476238	118.4476521	33.84033114
65.57801016	117.6630015	33.69828999
65.21586855	116.8754817	33.5418192
64.92169804	116.1286428	33.42235263
64.79378093	115.8419688	33.36243738
64.79717692	115.72555	33.37538141
64.85389925	116.036282	33.37864634
64.93624507	116.2788179	33.45725032
65.01996781	116.38252	33.44890897
65.10820598	116.5481815	33.54036317
66.1097758	118.2737369	34.01825073
67.07745699	120.4055128	34.59384888
67.0777037	120.3168133	34.5682485
67.08585838	120.3956922	34.58957869
NA	NA	NA
NA	NA	NA
NA	NA	NA
NA	NA	NA
NA	NA	NA
NA	NA	NA
NA	NA	NA
NA	NA	NA
NA	NA	NA
NA	NA	NA
NA	NA	NA
59.97572469	107.597145	30.80387567
59.8186644	107.3033096	30.74752155
59.70941652	107.3231779	30.71223277
59.64514145	107.0412523	30.72268944
59.6349951	107.216282	30.80350937
59.71805637	107.2879536	30.85438013
59.87360895	107.5586194	30.96270389
60.06063981	108.0854933	31.00632469
60.25406538	108.1007768	31.08944226

60.44527404	108.2835869	31.19352923
60.63375637	108.8748178	31.29808413
60.8159641	108.9063754	31.35796413
61.00119786	109.2145724	31.40633109
61.18978329	109.4352333	31.59968739
61.3729147	109.7106999	31.68768007
61.55830012	110.0615472	31.84471835
61.62255835	110.2707006	31.85685847
61.53394416	110.1708746	31.81094009
61.41787464	110.0939612	31.74095224
61.37815497	110.0112069	31.67742108
61.50615481	110.4265344	31.79601709
61.75557156	110.6289981	31.97228936
62.04653893	111.3339204	32.1039615
62.34992822	111.7587384	32.27625225
62.67961641	112.2835954	32.44314358
63.01352072	112.8360617	32.62746445
63.77369693	114.297149	32.98284245
64.48182916	115.9047287	33.2832399
64.70333475	116.2952418	33.40050758
64.92011473	116.7249321	33.53661852
NA	NA	NA
NA	NA	NA
NA	NA	NA
NA	NA	NA
NA	NA	NA
NA	NA	NA
NA	NA	NA
NA	NA	NA
NA	NA	NA
NA	NA	NA
NA	NA	NA
64.44142497	116.0483362	33.30399247
64.417976	115.6897772	33.30990524
64.42239372	115.6714391	33.34141743
64.44633222	115.7470099	33.43087788
64.5031539	115.4179934	33.48730317
64.61089802	115.4251554	33.50596332
64.74498035	115.6848152	33.53865078
64.88402906	115.8924474	33.63371204
65.01740876	116.3305527	33.68916098
65.15034986	116.523083	33.69982591
65.3076755	116.8938131	33.78094011
65.46226073	116.9471738	33.88435329
65.61028019	117.3639953	33.95804381
65.75547993	117.6528261	34.05210176
65.90151384	118.0313472	34.09060155
66.07277447	118.6769662	34.20659648
66.14909948	118.6456032	34.23204823
66.09312728	118.4853769	34.2067871
66.00948204	118.4126025	34.24876898
65.99211376	118.0449951	34.3798791
66.10796027	118.345341	34.48991914
66.32689499	118.6165371	34.59002657
66.57128277	118.8161303	34.64993826
66.83711526	119.5023547	34.72027267
67.12922924	120.0665062	34.81471703
67.43933947	120.5564945	35.00722337

68.26455655	122.415394	35.40005927
69.04152167	123.8969707	35.80021591
69.24345205	124.2838793	35.92063664
69.4451105	124.4943461	35.95789579
NA	NA	NA
NA	NA	NA
NA	NA	NA
NA	NA	NA
NA	NA	NA
NA	NA	NA
NA	NA	NA
NA	NA	NA
NA	NA	NA
NA	NA	NA
NA	NA	NA
62.16278167	111.6379492	32.00682584
62.07207315	111.4122291	32.004583
62.01922287	111.4459828	32.02769918
61.99913428	111.3918533	32.04455364
62.02299387	111.3958287	32.07662541
62.12029156	111.4242976	32.10553265
62.26803659	111.5572571	32.16014901
62.43445173	111.9328348	32.22513479
62.60114797	112.1519217	32.29129799
62.7661337	112.3486866	32.37377873
62.94217605	112.6740394	32.43651322
63.11372403	112.8804093	32.57143286
63.2831966	113.1825227	32.69083229
63.45262053	113.2652417	32.74758462
63.61922088	113.558997	32.83513065
63.7994779	113.9546281	32.9282496
63.87151342	113.9586415	33.0001877
63.80063598	113.8607749	33.00163286
63.70233031	113.6868391	32.98180262
63.6754308	113.6704563	33.01702052
63.7990975	113.9539701	33.10297866
64.03497234	114.3981177	33.22220076
64.30410314	114.9094288	33.32070643
64.5898573	115.5383157	33.47551068
64.90198935	116.049622	33.54188904
65.22529041	116.730734	33.73149497
66.01954709	118.2238739	34.12506543
66.76343076	119.9946193	34.5049526
66.97583018	120.3364441	34.60607608
67.18562025	120.617656	34.68413383
NA	NA	NA
NA	NA	NA
NA	NA	NA
NA	NA	NA
NA	NA	NA
NA	NA	NA
NA	NA	NA
NA	NA	NA
NA	NA	NA
NA	NA	NA
NA	NA	NA
89.35789245	161.0189478	46.01622623
89.29775012	161.0295401	45.90477989

89.24011484	161.1609344	45.9695085
89.18613997	160.8120557	45.98409247
89.12628639	160.8501685	45.96961257
89.08649219	160.9434534	46.01228558
89.12199201	161.0852968	45.94094665
89.26225017	161.1233771	46.04851454
89.43488363	160.9275613	46.14234237
89.57643125	161.1728753	46.13036273
89.64641054	161.0594413	46.10311545
89.60857444	160.7729922	46.22370019
89.54510875	160.6040725	46.35982446
89.46491426	160.2979421	46.52250885
89.40952464	159.8149848	46.58838309
89.43392583	159.4477501	46.69139044
89.44433794	159.3777467	46.68095246
89.41786072	159.5082344	46.54385494
89.36707778	159.5462911	46.57955358
89.34587001	159.3182775	46.48729999
89.38370715	159.6755669	46.5974071
89.43091771	159.7941019	46.58519928
89.45961717	159.7639119	46.47891354
89.49137468	159.5750903	46.55163526
89.51867846	159.7706051	46.57313517
89.57204254	159.7156096	46.62995239
89.72792453	161.579284	46.46915532
89.84972509	162.1011157	46.45504956
89.88902575	162.1596321	46.38771598
89.93831657	162.4978331	46.36518424
NA	NA	NA
NA	NA	NA
NA	NA	NA
NA	NA	NA
NA	NA	NA
NA	NA	NA
NA	NA	NA
NA	NA	NA
NA	NA	NA
NA	NA	NA
NA	NA	NA
90.32449195	160.1947266	46.71611265
90.31802282	160.2782795	46.77759098
90.31690547	160.5194074	46.71210572
90.31543081	160.2391119	46.70648145
90.31914962	160.227892	46.8306788
90.3285881	160.3535343	46.91765948
90.49866484	160.5705644	46.88088755
90.87418596	161.3565244	47.00920638
91.30629576	162.1829117	47.29867505
91.69483665	163.2086378	47.3432564
91.89083665	163.5627103	47.38834444
91.83157625	163.4583489	47.52651813
91.6136643	163.2278917	47.47664365
91.34732843	162.8079368	47.49152761
91.1424427	163.0031536	47.35551997
91.10430997	162.6547665	47.2764102
91.1655761	162.683983	47.44007462
91.18482444	162.8749285	47.48264997
91.19095785	163.2053107	47.4293706

91.19675585	163.0351858	47.47180268
91.27764775	162.9094705	47.48227391
91.30224542	162.7495408	47.60571466
91.24574866	162.671279	47.4519818
91.16418594	162.6535706	47.39080563
91.10501343	162.3410783	47.33292112
91.13165356	162.5170484	47.3978945
91.54159973	162.320913	47.57747204
91.94424194	163.1513279	47.71788337
91.99220123	162.9497549	47.63535499
92.05413308	163.2941399	47.67125647
NA	NA	NA
NA	NA	NA
NA	NA	NA
NA	NA	NA
NA	NA	NA
NA	NA	NA
NA	NA	NA
NA	NA	NA
NA	NA	NA
NA	NA	NA
NA	NA	NA
89.82812998	161.4172632	46.60181639
89.79443147	161.2491567	46.61294211
89.76504032	161.3432468	46.60511162
89.73731314	161.1430354	46.67010885
89.70939365	161.1133212	46.6003965
89.69459181	161.1333007	46.63038278
89.79745501	160.9925663	46.69225666
90.0550618	161.3769372	46.83620956
90.35728681	162.0916791	47.01740191
90.62246671	162.6979273	47.16193864
90.75599153	163.0346191	47.1530777
90.70830789	163.0281112	47.26174545
90.56872349	162.6841607	47.03391631
90.39615457	162.2670318	47.03772448
90.26620295	161.7935915	46.89815441
90.2594497	161.3609456	46.87325652
90.29536125	161.5673176	46.91172988
90.29189978	161.6827241	46.89922226
90.27001823	161.9114667	46.88821575
90.26279814	161.7455452	46.91612695
90.32250158	161.9093648	46.97401991
90.35894515	161.8759805	47.00020695
90.34541597	161.8407807	46.83784852
90.32096151	161.7602062	46.9118565
90.30539328	161.7591518	46.84766588
90.34559524	161.9811681	46.88702681
90.62935617	162.3828183	47.17222355
90.89238005	163.2483014	47.24760905
90.93645873	163.1324838	47.17934675
90.99246896	163.1305321	47.13591386
NA	NA	NA
NA	NA	NA
NA	NA	NA
NA	NA	NA
NA	NA	NA
NA	NA	NA

NA	NA	NA
NA	NA	NA
NA	NA	NA
NA	NA	NA
NA	NA	NA
90.80775539	162.9426764	46.67643177
90.80898785	163.1223136	46.75395578
90.82660774	163.2096244	46.69700138
90.82884661	162.9691777	46.68392028
90.82429578	163.0370628	46.60434813
90.79139333	163.0454685	46.57413203
90.76901375	162.9928417	46.75244329
90.74528859	162.8762955	46.62926386
90.70629897	162.8318272	46.72120671
90.68684446	162.5458588	46.6919507
90.65635661	162.2805163	46.6855695
90.69027358	162.6426772	46.58626387
90.75987531	162.8184217	46.59654177
90.85715233	162.6777612	46.79866169
90.95018416	162.6191887	46.75233913
90.98811318	162.277236	46.78876963
90.99324398	162.4711737	46.82704758
91.00515254	162.1840206	46.78892544
91.00938778	162.226807	46.89315
91.0220708	162.4027187	46.85970741
91.02003401	162.6060326	46.88560731
91.02311218	162.4707833	46.87843902
91.01939653	162.2989685	46.87149535
91.03202228	162.579857	46.85566645
91.02956802	162.720211	46.89970947
91.0317962	162.5592283	46.78321268
90.86552585	163.0424979	46.73749096
90.70254677	163.0183404	46.67236083
90.66789302	163.2028888	46.73446341
90.64066291	162.7455143	46.74236624
NA	NA	NA
NA	NA	NA
NA	NA	NA
NA	NA	NA
NA	NA	NA
NA	NA	NA
NA	NA	NA
NA	NA	NA
NA	NA	NA
NA	NA	NA
NA	NA	NA
93.53605207	168.5693027	48.57504247
93.55020913	168.4025509	48.57447707
93.5818774	168.2991086	48.66201183
93.61198987	168.4091371	48.65206223
93.61430609	168.3770116	48.78319397
93.60525504	168.6888488	48.58570558
93.60081859	168.4992956	48.61499333
93.5994162	168.7698755	48.6556357
93.60740451	168.4025049	48.61670182
93.61383448	168.3089663	48.63953789
93.63271563	168.2567527	48.71374264
93.6524573	168.4180485	48.71484009

93.70484693	168.3303246	48.64285185
93.75367213	168.8399463	48.83724872
93.79815446	168.5773173	48.83033579
93.82938895	168.5312065	48.94884177
93.84951154	168.5556346	48.8166467
93.88537761	168.386554	48.84874779
93.90192592	168.8302064	48.96125755
93.92033728	168.7633493	48.94070336
93.9259459	168.6400199	49.0148404
93.93926276	168.4696467	48.97594616
93.94507013	168.6715167	48.86676869
93.96205099	168.6627579	48.94084315
93.96831303	168.5655997	48.84977029
93.96978212	168.7980552	48.84161078
93.91298927	169.4927067	48.82034317
93.85961715	169.4133364	48.64550528
93.84405183	169.2783052	48.79027561
93.81829451	169.3138308	48.67308166
NA	NA	NA
NA	NA	NA
NA	NA	NA
NA	NA	NA
NA	NA	NA
NA	NA	NA
NA	NA	NA
NA	NA	NA
NA	NA	NA
NA	NA	NA
NA	NA	NA
92.17538379	165.2433268	47.97770692
92.18260641	165.2939308	47.97087316
92.2065975	165.3840293	47.97776811
92.22197582	165.1997691	47.96532386
92.22099024	165.769494	47.92393606
92.20048035	165.4161376	47.89218556
92.18765356	165.4753949	47.96482337
92.17590727	165.4697586	47.91152191
92.16100288	165.312813	47.98124022
92.15543353	165.3531104	47.95451669
92.15052822	165.2836668	47.96597066
92.17788814	165.3084085	47.9971973
92.23899147	165.4828465	47.95949088
92.31249483	165.6441451	47.88703087
92.38169352	165.8742607	47.91461152
92.41694523	165.7114487	47.93401613
92.43003162	165.6944137	48.01127621
92.45444827	165.5484878	47.89760578
92.46562144	165.9386286	47.95731505
92.48178189	165.8833424	47.91603302
92.48440401	165.6571807	47.96661354
92.49337368	165.6693169	48.04155116
92.49525129	165.5917189	47.93506567
92.51096558	165.7501496	48.08278557
92.51367806	165.8478178	48.12398521
92.51616053	166.0405226	47.97311633
92.40525879	166.2835173	48.10015355
92.2975363	165.9572475	47.98224901
92.27274337	166.0590844	48.03619793

92.24657267	165.7304327	48.0186824
NA	NA	NA
NA	NA	NA
NA	NA	NA
NA	NA	NA
NA	NA	NA
NA	NA	NA
NA	NA	NA
NA	NA	NA
NA	NA	NA
NA	NA	NA
NA	NA	NA
